# Comprehensive comparative morphology and developmental staging of final instar larvae toward metamorphosis in the insect order Odonata

**DOI:** 10.1038/s41598-021-84639-2

**Published:** 2021-03-04

**Authors:** Genta Okude, Takema Fukatsu, Ryo Futahashi

**Affiliations:** 1 Department of Biological Sciences, Graduate School of Science, The University of Tokyo, Tokyo, Japan; 2 Bioproduction Research Institute, National Institute of Advanced Industrial Science and Technology (AIST), Tsukuba, Japan; 3 Graduate School of Life and Environmental Sciences, University of Tsukuba, Tsukuba, Japan

**Keywords:** Entomology, Biological metamorphosis

## Abstract

The order Odonata (dragonflies and damselflies) is among the most ancestral groups of winged insects with drastic morphological changes upon metamorphosis, and thus important for understanding evo-devo aspects of insects. However, basic developmental descriptions of Odonata have been scarce. In an attempt to establish the foundation of developmental and experimental biology of Odonata, we present an unprecedentedly comprehensive survey of dragonflies and damselflies, in total 158 larvae representing 49 species and 14 families, wherein morphological changes of all the final and/or penultimate instar larvae were photographed and monitored everyday. Although their morphology and development were diverse, we consistently identified two visually recognizable morphogenetic events in the final larval instar, namely start of wing expansion and onset of melanization on the wing sheaths, thereby categorizing the final instar into three stages. While the duration of the first stage ranged 4–66 days across diverse Odonata species, the second or third stages exhibited relatively small variation ranging 3–22 days or 1–8 days, respectively, probably reflecting the steady and irreversible metamorphosis process after stage 2. We also described other characteristic morphological changes during the larval development, although they were observed only in some Odonata species and lineages.

## Introduction

Dragonflies and damselflies (order Odonata) are among the most ancestral winged insects^1,2^ and radically change their morphology, habitat, and behavior from aquatic larvae to terrestrial adults without pupal stage^3,4^, which entail drastic changes of gene expression patterns (e.g. opsin genes in compound eyes^5,6^). In Odonata, while adults show an impressive diversity in their body colors and patterns, larvae exhibit a remarkable diversity in their body shape, which reflect their habitats and ecological niches^7,8^. Because mayflies (order Ephemeroptera), which also represent the most ancestral winged insects with Odonata, undergo unique subimago stage between the final instar larva and the adult, elucidation of the developmental progress of the last three stages of Odonata (i.e., penultimate instar larva, final instar larva, and adult) is important for understanding the evolution of insect metamorphosis. Despite the potential biological importance, the basic information on morphological changes of Odonata during larval-adult metamorphosis has been quite fragmentary, mainly because aquatic and carnivorous Odonata larvae are difficult to maintain in the laboratory. The majority of previous studies focused on the morphological changes during the final larval instar of a single species as follows: Coenagrionidae (*Ischnura verticalis*^9^; *Coenagrion hastulatum*^10^; *Pyrrhosoma nymphula*^11^; *Ischnura senegalensis*^12^; hybrids of *Ischnura elegans* and *I. senegalensis*^13^), Aeshnidae (*Anax imperator*^14^; *Aeshna cyanea*^15^; *Aeshna viridis*^16^), Gomphidae (*Asiagomphus pryeri*^17^) and Libellulidae (*Pachydiplax longipennis*^18^; *Pseudothemis zonata*^19^; *Urothemis assignata*^20^). In these studies, the final larval instar was categorized into 3 to 8 developmental stages defined by morphological traits. However, since different characteristics were used as indicators of morphological changes among different species and most papers did not describe the precise timing for each stage, it is very difficult to comparatively analyze these morphological and developmental data from different Odonata species in a common framework.

Hence, in an attempt to establish the foundation of developmental and experimental biology of Odonata, we performed an unprecedentedly comprehensive morphological survey of dragonflies and damselflies. We everyday recorded the morphological changes during final and/or penultimate larval instar using 158 individuals of 49 Japanese Odonata species representing 14 families. From the enormous amount of morphological data based on approximately 5000 photos, the following two morphological changes, namely “the start of wing expansion” and “the onset of melanization on the wing sheaths”, emerged as easily and commonly recognizable developmental indices across the diverse Odonata species. Based on these indicators, we propose that the final larval instar of Odonata could be generally classified into three developmental stages. Other morphological changes in the compound eyes, heads, larval labia, and wing sheaths were also described in detail, although they were observed in more specific Odonata lineages and species.

## Methods

### Terminology

Following the majority of previous publications, we described the final larval instar as F−0 (= Final) and the penultimate larval instar as F−1 (= F minus 1), because the number of larval instars is not fixed in many Odonata species^3,12^. We started daily photographing of each larva on the day of ecdysis unless otherwise mentioned (Table S1), where the “day 1” denotes the day on which photographing started for each instar.

### Sample collection and rearing

**Figure 1:**
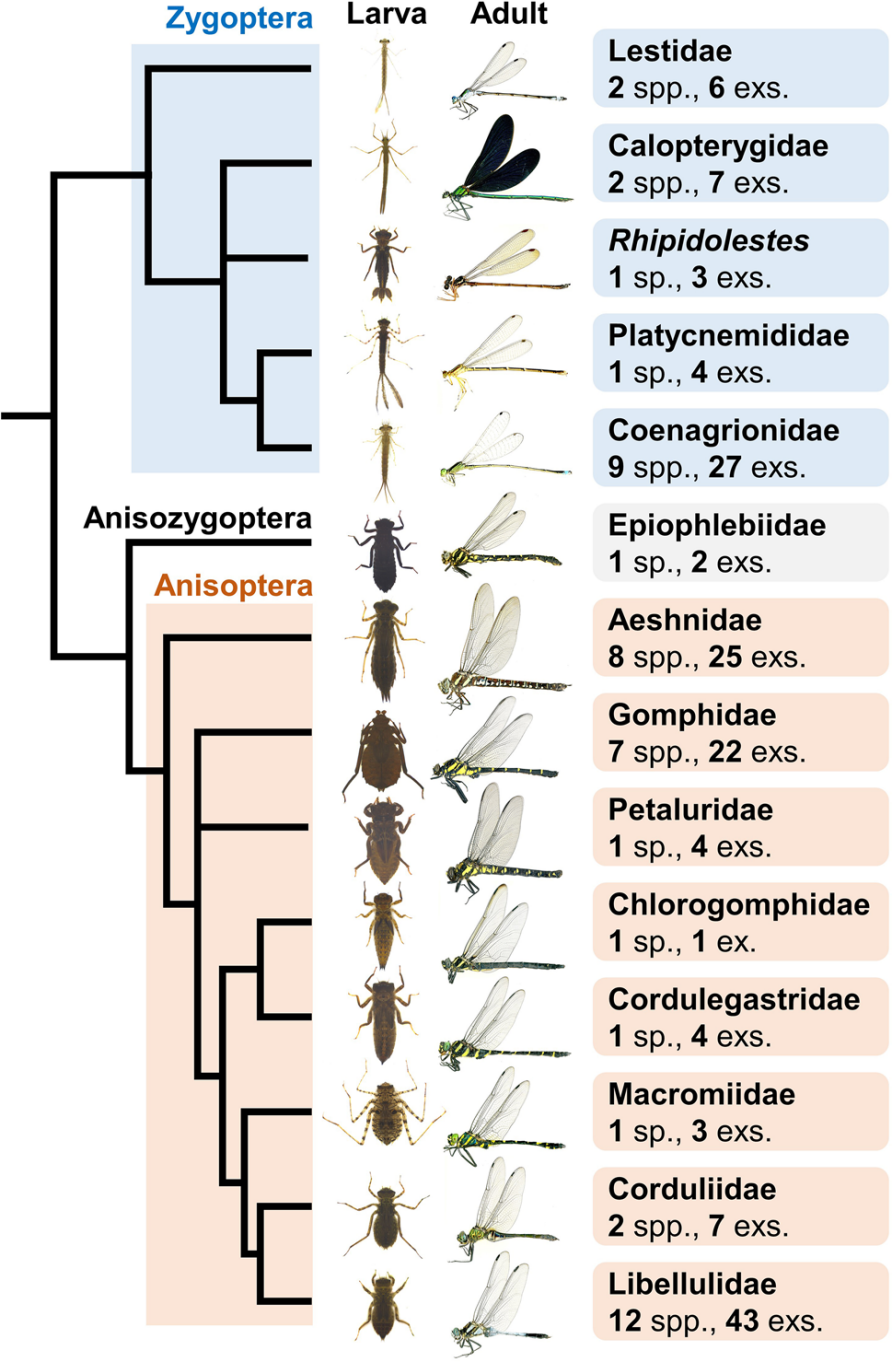
Summary of Odonata species examined in this study. 49 Odonata species (158 individuals) were photographed everyday (see Table 1 and Table S1 in detail). Phylogenetic tree of Odonata was modified from Bybee et al.^4^. The family status of* Rhipidolestes* is currently uncertain (*incertae sedis* group 1^27^). Larva and adult photos of the following 14 species are shown: *Lestes sponsa* (Lestidae), *Calopteryx japonica* (Calopterygidae), *Rhipidolestes hiraoi* (*incertae sedis*), *Pseudocopera annulata* (Platycnemididae), *Ischnura asiatica* (Coenagrionidae), *Epiophlebia superstes *(Epiophlebiidae), *Aeshna crenata* (Aeshnidae), *Sieboldius albardae* (Gomphidae), *Tanypteryx pryeri* (Petaluridae), *Chlorogomphus brunneus* (Chlorogomphidae), *Anotogaster sieboldii *(Cordulegastridae), *Macromia daimoji* (Macromiidae), *Somatochlora uchidai *(Corduliidae), and *Orthetrum albistylum *(Libellulidae).

All the 158 larvae of 49 Odonata species representing 14 families (Fig. 1, listed in Table 1) were collected in the field or reared from eggs that were obtained from adult females in Japan (Table S1). The larvae were individually kept in petri dishes with water in the laboratory. The damselfly larvae (suborder Zygoptera) were fed with *Artemia* brine shrimps, whereas dragonfly larvae (suborders Anisozygoptera and Anisoptera) were fed with *Chironomus* midge larvae. Soon after feeding cessation of the F−0 instar larvae prior to the melanization on the wing sheaths, we transferred them individually to a plastic cage for adult emergence as described previously^12^.

**Table 1:** Interspecific diversity of morphological changes during F−0 instar larvae of 49 species in this study and the additional two species from other references.

Species no.	Family	Species	The number of individuals		Clear emergence of newly formed regions	Compound eyes expansion to dorsal side	Wing sheaths Expansion	Wrinkles on the wings	Pigmentation on the head	Apolyses on compound eyes	Degradation of larval labium	Black markings on the basal wings	Blact dots emergence	Bristles melanization
					Stage 1	Stage 2						Stage 3		
			F−1	F−0	1–1	2–1	2–2	2–3	2–4	2–5	2–6	3–1	3–2	3–3
1	Lestidae	*Lestes sponsa*	1	1	×	△(*2)	○	○	○	×	–	○	×	×
2	Lestidae	*Lestes temporalis*	1	5	×	△(*2)	○	○	○	×	–	○	×	×
3	Calopterygidae	*Mnais costalis*	0	6	×	△(*2)	○	○	×	×	–	○	○	×
4	Calopterygidae	*Calopteryx japonica*	0	1	×	△(*2)	○	○	○	×	–	○	○	○
5	(family status uncertain)	*Rhipidolestes hiraoi*	0	3	×	–	○	○	×	×	–	○	○	×
6	Platycnemididae	*Pseudocopera annulata*	0	4	×	△(*2)	○	○	○	×	–	○	○	○
7	Coenagrionidae	*Paracercion calamorum*	4	7	×	○	○	○	○	×	–	○	○	○
8	Coenagrionidae	*Paracercion hieroglyphicum*	0	3	×	○	○	○	○	×	–	○	○	○
9	Coenagrionidae	*Paracercion sieboldii*	1	3	×	○	○	○	○	×	–	○	○	○
10	Coenagrionidae	*Paracercion melanotum*	0	2	×	○	○	○	○	×	△(*3)	○	○	○
11	Coenagrionidae	*Agriocnemis pygmaea*	0	1	×	○	○	×	○	×	–	○	○	○
12	Coenagrionidae	*Mortonagrion selenion*	0	1	×	○	○	×	○	×	–	○	–	–
13	Coenagrionidae	*Enallagma circulatum*	1	5	×	○	○	○	○	×	–	○	○	○
14	Coenagrionidae	*Ischnura senegalensis*	0	2	×	○	○	×	○	×	×	○	○	○
15	Coenagrionidae	*Ischnura asiatica*	0	3	×	○	○	△(*3)	○	×	–	○	○	○
Ref.1	Coenagrionidae	*Ischnura senegalensis*	3	6	×	○	○	×	○	×	–	○	○	○
Ref.2	Coenagrionidae	*Ischnura elegans* x *I. senegalensis*	4	6	×	○	○	×	○	×	–	○	○	○
16	Epiophlebiidae	*Epiophlebia superstes*	0	2	×	×	○	×	×	×	–	×	×	×
17	Aeshnidae	*Boyeria maclachlani*	0	2	×	×	○	×	×	○	–	×	○	×
18	Aeshnidae	*Planaeschna milnei*	0	3	×	×	○	△(*3)	×	○	–	○	○	×
19	Aeshnidae	*Gynacantha japonica*	1	4	×	×	○	○	×	○	–	×	○	×
20	Aeshnidae	*Aeshna crenata*	0	4	×	×	○	○	×	○	–	○	○	×
21	Aeshnidae	*Aeshna juncea*	0	1	×	×	○	○	×	○	–	○	○	×
22	Aeshnidae	*Anax ephippiger*	4	5	×	×	○	×	×	○	○	×	○	○
23	Aeshnidae	*Anax parthenope*	1	1	×	×	○	△(*3)	×	○	–	×	○	○
24	Aeshnidae	*Anax nigrofasciatus*	1	1	×	×	○	△(*3)	×	○	–	○	○	×
25	Gomphidae	*Sieboldius albardae*	0	3	△(*1)	×	○	×	×	×	–	×	○	×
26	Gomphidae	*Melligomphus viridicostus*	0	3	△(*1)	×	○	○	×	×	–	○	○	×
27	Gomphidae	*Nihonogomphus viridis*	0	1	△(*1)	×	○	○	×	×	–	○	○	×
28	Gomphidae	*Davidius nanus*	0	5	△(*1)	×	○	○	×	×	–	○	○	×
29	Gomphidae	*Sinogomphus flavolimbatus*	0	2	△(*1)	×	○	×	×	×	–	×	×	×
30	Gomphidae	*Stylogomphus suzukii*	0	4	△(*1)	×	○	○	×	×	–	○	○	×
31	Gomphidae	*Asiagomphus melaenops*	0	4	△(*1)	×	○	×	×	×	–	○	○	×
32	Petaluridae	*Tanypteryx pryeri*	0	4	×	×	○	×	×	×	×	×	○	×
33	Chlorogomphidae	*Chlorogomphus brunneus*	0	1	△(*1)	×	○	×	×	×	○	○	○	×
34	Cordulegastridae	*Anotogaster sieboldii*	0	4	△(*1)	×	○	×	×	×	–	○	○	×
35	Macromiidae	*Macromia daimoji*	0	3	△(*1)	×	○	○	×	×	–	○	○	×
36	Corduliidae	*Epitheca bimaculata*	0	1	–	×	○	×	×	×	–	○	○	×
37	Corduliidae	*Somatochlora uchidai*	0	6	△(*1)	×	○	○	×	×	–	○	○	×
38	Libellulidae	*Rhyothemis fuliginosa*	0	7	△(*1)	×	○	○	×	×	–	○	○	○
39	Libellulidae	*Sympetrum darwinianum*	0	3	○	×	○	○	×	○	○	○	○	○
40	Libellulidae	*Sympetrum maculatum*	0	4	○	×	○	○	×	○	○	○	○	○
41	Libellulidae	*Sympetrum infuscatum*	0	3	○	×	○	○	×	○	–	○	○	○
42	Libellulidae	*Sympetrum frequens*	0	1	○	×	○	○	×	○	–	○	○	○
43	Libellulidae	*Sympetrum kunckeli*	0	3	○	×	○	○	×	○	–	○	○	○
44	Libellulidae	*Sympetrum uniforme*	3	3	○	×	○	○	×	○	–	×	○	○
45	Libellulidae	*Pseudothemis zonata*	0	4	△(*1)	×	○	×	×	○	–	×	○	○
46	Libellulidae	*Deielia phaon*	0	1	○	×	○	×	×	×	–	×	○	○
47	Libellulidae	*Acisoma panorpoides*	0	1	△(*1)	×	○	×	×	○	–	○	○	○
48	Libellulidae	*Crocothemis servlia*	1	5	○	×	○	○	×	○	–	○	○	○
49	Libellulidae	*Orthetrum albistylum*	3	5	○	×	○	×	×	×	–	○	○	×

○: Clearly observed.

△(*1): Difficult to determine the start point when the newly formed regions appear.

△(*2): Gradually expand since stage 1.

△(*3): Difficult to detect in some individuals.

×: Undetectable.

Ref. 1: Okude et al.^12^ (*Ischnura senegalensis*, Coenagrionidae). Ref. 2: Okude et al.^13^ (hybrids of *I. elegans* and *I. senegalensis*).

### Species identification

Most Japanese Odonata species were identified by their morphological characters^8,9^, while some species were identified by their nuclear DNA sequences as described below. We extracted DNA from a caudal gill of Zygopteran larvae or a pair of forelegs of newly-emerged Anisopteran adults by using QIAamp DNA Mini Kit (Qiagen). Nuclear ITS1 region was PCR-amplified from the DNA samples using the primers ITS-F0 (5′-GGA AAG ATG GCC AAA CTT GA-3′) and 5.8S-AS1 (5′-GCC GGC CCT CAG CCA G-3′)^21^. PCR-amplified products were subjected to DNA sequencing using BigDye Terminator v3.1 Cycle Sequencing Kit (Applied Biosystems) and 3130xl Genetic Analyzer (Applied Biosystems). Nuclear ITS sequences of all the Japanese Odonata species have been previously deposited in the public database^22^.

### Photographing

F−0 and/or F−1 instar larvae were photographed everyday using a stereoscopic microscope S8APO (Leica Microsystems) with a digital high definition microscope camera MC120HD (Leica), a stereoscopic microscope S9D (Leica Microsystems) with a digital high definition microscope camera MC190HD (Leica), or a stereoscopic microscope SZ-6850T (Relyon) with a digital high definition microscope camera TrueChrome Metrics (Relyon). Most larvae were photographed from the day of ecdysis, whereas some larvae were photographed from the day of collection, when the insects were already F−0 instar (See Table S1). The obtained photographs were adjusted by using software GIMP (GNU Image Manipulation Program) 2.10.6.

## Results and discussion

### Three developmental stages during F−0 instar commonly observed among diverse Odonata species

**Figure 2:**
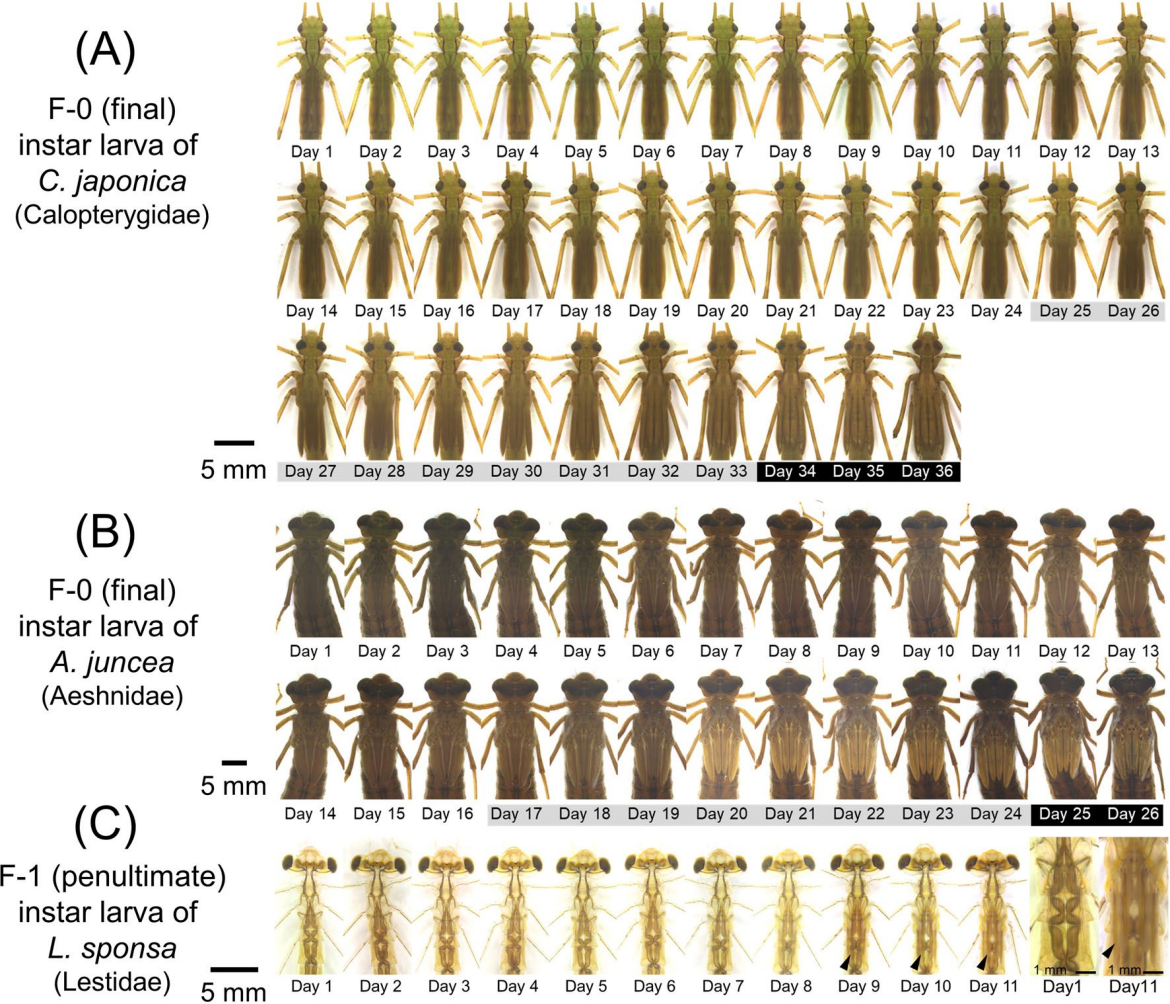
Examples of daily photos obtained in this study. For all photos, see Figs. S2 and S3. The higher quality photos are deposited in the Dryad Digital Repository: 10.5061/dryad.tht76hdz6. (**A**) F−0 instar female larva of *Calopteryx japonica* (Calopterygidae, Zygoptera). Adult emergence was observed on Day 37. (**B**) F−0 instar larva female of *Aeshna juncea* (Aeshnidae, Anisoptera). Adult emergence was observed on Day 27. (**C**) F−1 instar female larva of *Lestes sponsa* (Lestidae, Zygoptera). Ecdysis to F−0 instar was observed on Day 12. Black arrowheads indicate the expansion of the wing sheaths. Day numbers shaded in gray or black indicate stage 2 or 3, respectively.

**Figure 3:**
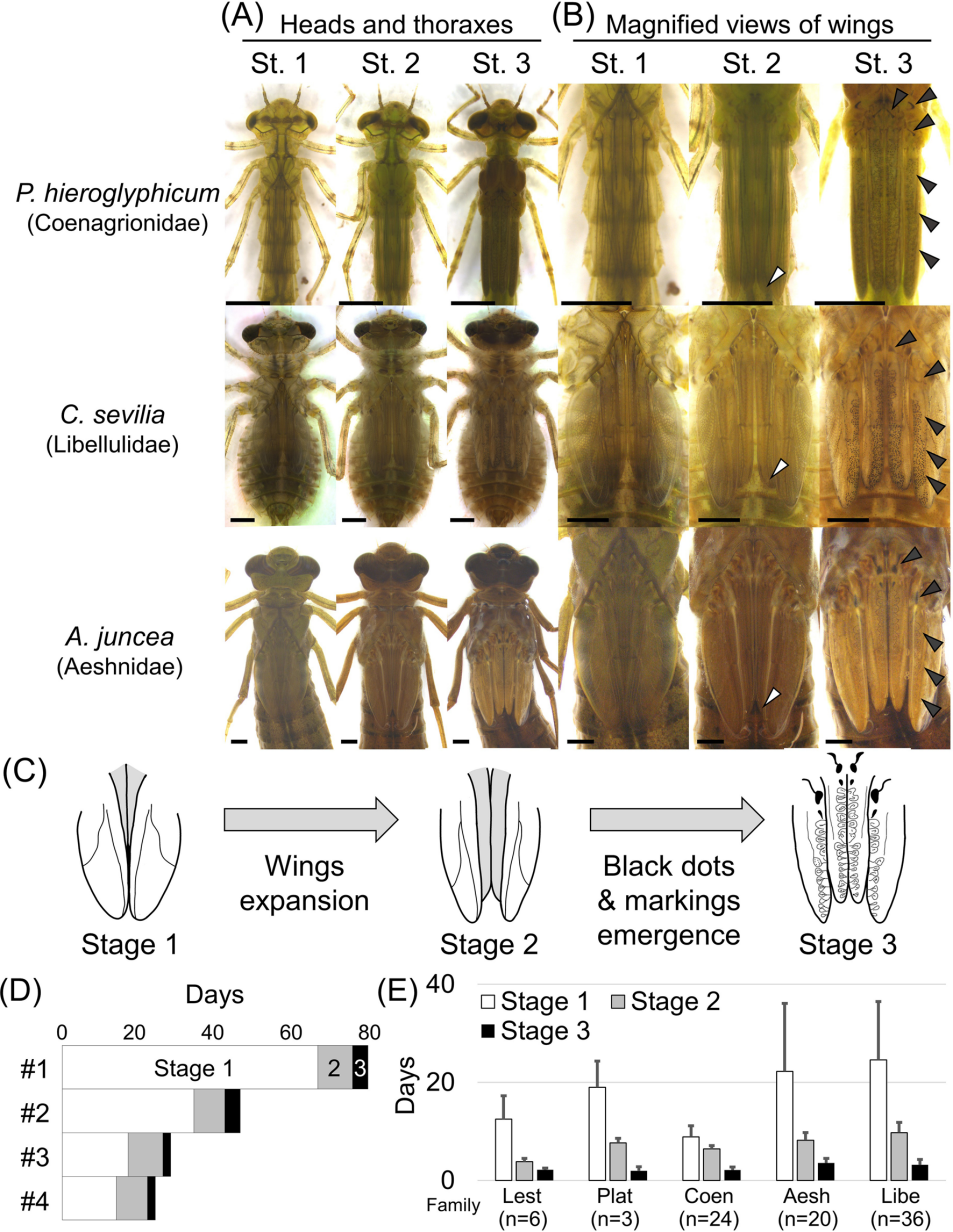
Summary of three developmental stages commonly observed in F−0 instar larvae among diverse Odonata species. (**A**) Dorsal views of heads, thoraces, and wings. (**B**) Magnified views of wing sheaths. White arrowheads indicate the dorsal tips of forewings (defining the onset of stage 2). Black arrowheads indicate the black dots and markings on the wing sheaths (defining the onset of stage 3). All scale bars show 2 mm. All the photos were taken on the first day of each stage. (**C**) Schematic diagrams of three developmental stages. (**D**) Durations of stages 1, 2 and 3 of four individuals of *Aeshna crenata* (Aeshnidae), highlighting varied lengths of stage 1 compared with relatively short and constant lengths of stages 2 and 3 (also see Table S1). (**E**) Durations of stages 1, 2 and 3 of F−0 instar larvae representing different Odonata families. Error bars show standard deviations. *Lest* Lestidae, *Plat* Platycnemididae, *Coen* Coenagrionidae, *Aesh* Aeshnidae, *Libe* Libellulidae.

We took photos of 158 Odonata larvae (150 F−0 instar and/or 22 F−1 instar) of 49 species everyday (Fig. 1; Tables 1, S1), and all the 4979 photos were aligned for comparison of morphological characters within and between the species (Figs. 2; S1, S2, S3). First, we compared daily morphological changes of F−0 instar larvae thoroughly. The morphological indicators used for larval staging were selected on account of the following requirements: (i) the morphological changes can be observed without damaging the larvae, (ii) the changes are discontinuous rather than gradual so that the specific timing of entry to the next stage can be clearly defined, and (iii) the indicators should be applicable to as many species as possible. The most prominent morphological changes commonly observed in almost all the examined species were “the start of wing expansion” and “the onset of melanization on the wing sheaths”. Based on these indicators, the F−0 larval instar could be classified into three developmental stages for most of the examined Odonata species (Figs. 3, S4; Table 1). In stage 1, the wing sheaths are flat without noticeable structures and pigmentation. The tips of the forewings are hidden under the hindwings (Figs. 3B, S4). In stage 2, the wing sheaths begin to expand. We can distinguish stage 2 from stage 1 by the criteria that the tips of the forewings appear from under the hindwings (Figs. 3B, S4, white arrowheads). In stage 3, black dots appear around the anterior edges of the wing sheaths, and black pigmentation also appears at the base of the wing sheaths (Figs. 3B, S4, black arrowheads). To help readers verify our judgment, we provide daily photographs and the duration of each stage for all individuals (Figs. S1, S2, S3; Table S1; higher quality photos are deposited in the Dryad Digital Repository: 10.5061/dryad.tht76hdz6). Among the 49 species we examined, stage 3 was difficult to define for only two species (*Epiophlebia superstes* and *Sinogomphus flavolimbatus*), because their entire body is very dark in color (Tables 1, S1; Fig. S1). It should be noted that two species of the genus *Lestes* (Lestidae) did not exhibit black dots on the wings but could be classified as stage 3 by melanization at the base of the wing sheaths (Tables 1, S1; Fig. S1) While a previous study partly mentioned these criteria for developmental staging of *Pyrrhosoma nymphula*^11^, here we demonstrate that these developmental criteria are applicable to most of the diverse Odonata species (Table 1). Next, we examined morphological changes during F−1 (penultimate) larval instar of Odonata, which were described in a few previous studies for only three species: *Pachydiplax longipennis*^18^, *Aeshna viridis*^16^ and *Ischnura senegalensis*^12^. For all the 22 F−1 instar larvae representing 12 species (Tables 1, S1), the compound eyes gradually tilted during F−1 instar, and the wing sheaths began to expand a few days before ecdysis to F−0 instar (Figs. 2C, S1).

### Variation in the duration of developmental stages during F−0 instar within and between Odonata species

We noticed significant intra-specific variation in the total duration of the F−0 larval instar (Fig. 3D; Table S1). We compared the durations of these developmental stages and identified large variation in the durations of stage 1 within and between the Odonata species (Fig. 3D,E). By contrast, the durations of stage 2 and stage 3 were less variable between and within the species (Figs. 3D,E, S5). Many river-dwelling Odonata species (e.g. Calopterygidae, Gomphidae, Cordulegastridae, Chlorogomphidae, and Macromiidae) are known to overwinter as F−0 instar larvae^14,17^. In these species, notably, while the total durations of F−0 larval instar were generally and conspicuously long (Table S1), the durations of stage 2 and stage 3 were relatively short and similar between species, 3–21 days and 1–8 days respectively (Figs. 3E, S5). The duration of larval instar (especially before entering stage 2 of F−0 instar) under natural conditions may differ from our results because only a limited type of food was supplied in this study. However, we confirmed that the larvae can become adults without food after entering stage 2 of F−0 instar. Overall, the durations after the onset of morphological change (i.e., stage 2 and on) exhibited relatively small variation, as reported in *I. senegalensis*^12^, suggesting that the developmental processes for metamorphosis after stage 2 proceed steadily and irreversibly.

### Morphological changes in compound eyes of damselfly larvae

**Figure 4:**
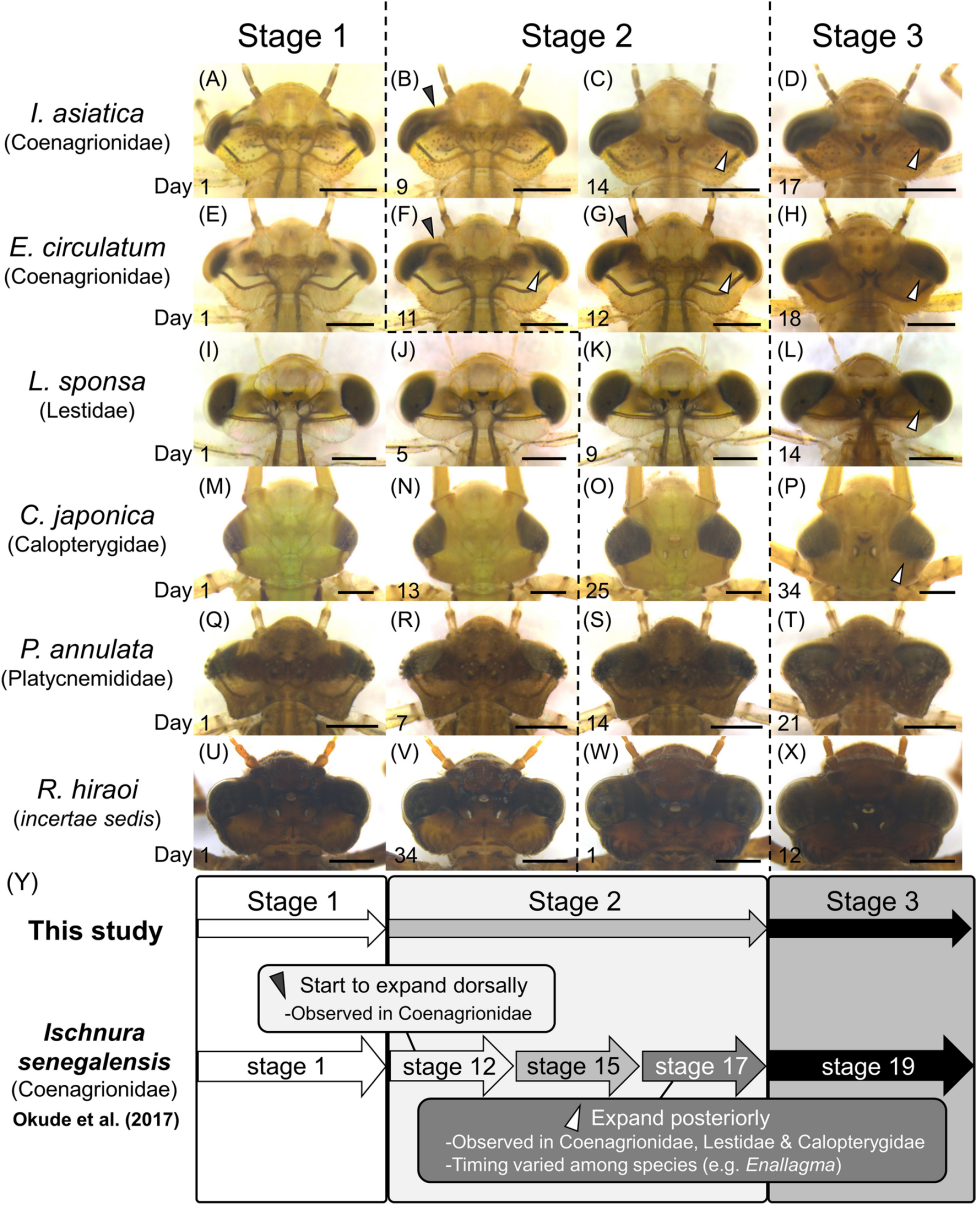
Morphological changes during F−0 instar observed in the compound eyes of damselfly (Zygoptera) larvae. (**A**–**X**) Dorsal views of the heads. All scale bars show 1 mm. Species and family names are provided on the left side. The number shown on the lower left of each image indicates the number of days after ecdysis to F−0 instar (**A**–**T**) and after starting of photographing (**U**–**X**). Black arrowheads indicate the dorsal expansion of the compound eyes, whereas white arrowheads indicate the posterior expansion of the compound eyes. (**A**, **E**, **I**, **M**, **Q**) The first day of stage 1. (**B**, **F**, **K**, **O**, **S**) The first day of stage 2. (**C**) The first day of stage 17^12^ in which posterior expansion begin. (**D**, **H**, **L**, **P**, **T**) The first day of stage 3. (**U**) The first day of photographing in individual No.5–1 (stage 1). (**V**) The 34th day of the photographing in individual No. 5–1 (stage 1). This individual No. 5–1 died before entering stage 2. (**W**) The first day of the photographing in individual No. 5–2 (early stage 2). (**X**) The first day of stage 3 in individual No. 5–2. (**Y**) Schematic diagrams of the comparison of the developmental stages during F−0 larval instar defined in this study and those proposed by Okude et al.^12^.

To our knowledge, Grieve^9^ first mentioned the expansion of the compound eyes during F−0 larval instar in the damselfly *Ischnura verticalis* (Coenagronidae). Recently, in *I. senegalensis* (Coenagrionidae), we categorized F−0 larval instar into five developmental stages that entail the following two-step expansions of the compound eyes: the dorsal expansion (Fig. 4B,a black arrowhead) in the 2nd stage (stage 12) and the posterior expansion (Fig. 4C,D, white arrowheads) in the 4th stage (stage 17) after Okude et al.^12^ (Fig. 4Y). Our comparative survey of diverse damselfly species (Zygoptera) revealed that the two-step expansions of the compound eyes were recognized only in Coenagrionidae species (Fig. 4; Table 1), and the two-step expansions are sometimes observed simultaneously during stage 2 in this study (ex. *Enallagma circulatum*; Fig. 4E–H). We confirmed that stage 1 of Okude et al.^12^ is consistent with stage 1 of this study, whereas stage 19 of Okude et al.^12^ is the same as stage 3 of this study (Fig. 4Y).

In Lestidae (Fig. 4I–L) and Calopterygidae species (Fig. 4M–P), the dorsal expansion of the compound eyes was certainly recognizable, but this expansion proceeded gradually from stage 1 to stage 2 and thus was not suitable as an indicator for developmental staging, whereas the posterior expansion of the compound eyes was faintly observed before stage 3 (Fig. 4L,P, white arrowheads). In Platycnemididae and *Rhipidolestes* (family status is uncertain) species, the expansion of the compound eyes was observed less obviously, which was partly due to the melanization on their body surface and thus not suitable as an indicator for developmental staging (Fig. 4Q–X). Hence, we concluded that the developmental stages 12, 15 and 17 defined by Okude et al.^12^ (Fig. 4Y) are only applicable to some Coenagrionidae species.

### Morphological changes in compound eyes of dragonfly larvae

**Figure 5:**
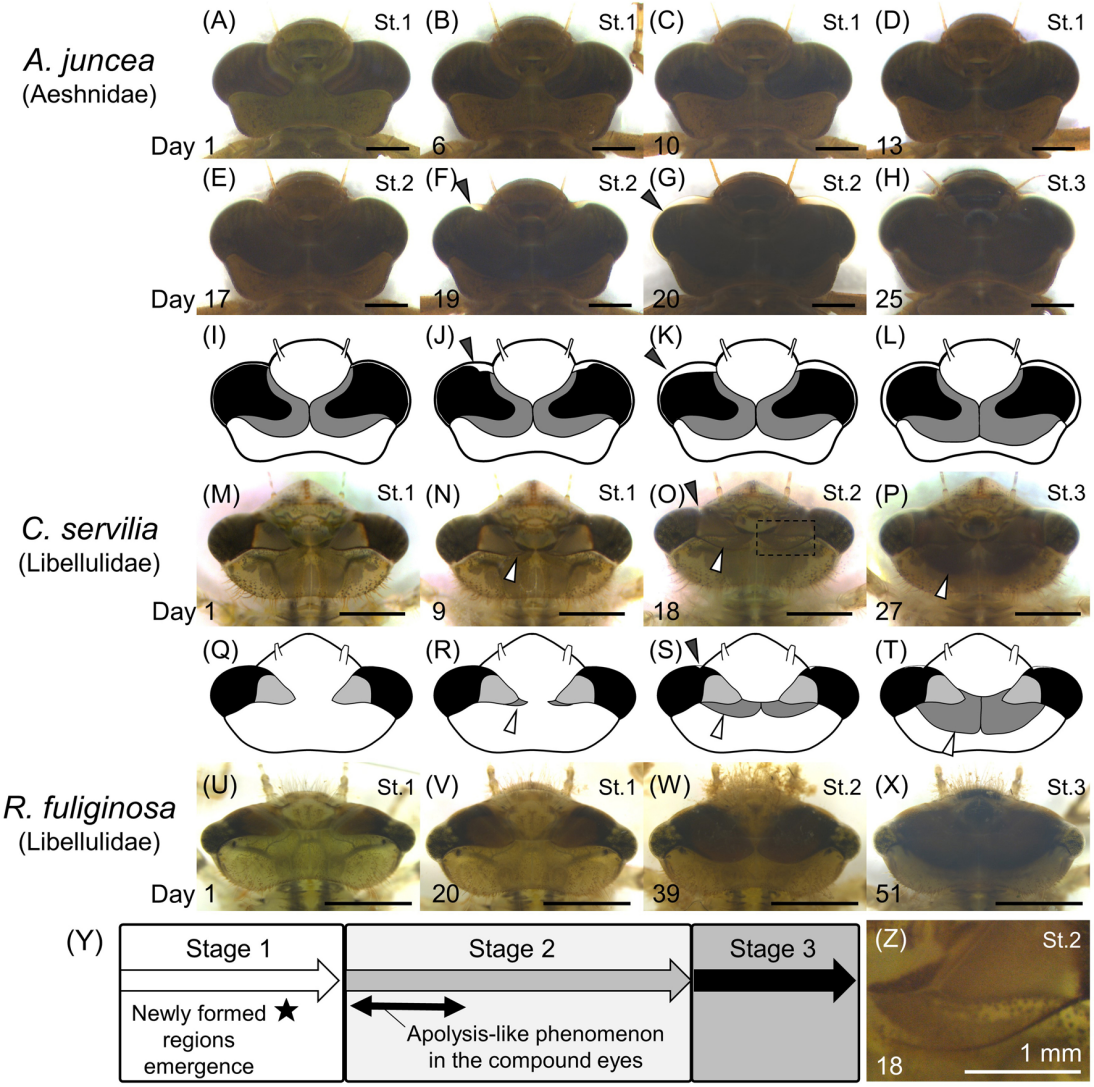
Morphological changes of the compound eyes during F−0 instar in Aeshnidae and Libellulidae larvae. (**A**–**X**) Dorsal views of the heads. All scale bars show 2 mm. Species and family names are provided on the left side. The number shown on the lower left of each image indicates the number of days after ecdysis to F−0 instar. Black arrowheads indicate the progression of the apolysis-like phenomenon in the compound eyes. White arrowheads indicate the newly formed regions of the compound eyes. (**I**–**L**, and **Q**–**T**) Schematic illustration of changes in shape and size of compound eyes. (**I**–**L**) or (**Q**–**T**) correspond to (**E**–**H**) or (**M**–**P**), respectively. (**A**, **M**, **U**) The first day of stage 1. (**E**, **I**, **O**, **S**, **W**) The first day of stage 2. (**H**, **L**, **P**, **T**, **X**) The first day of stage 3. (**N**, **R**) First day when the newly formed regions appeared. (**Y**) Schematic diagrams on the timing of the morphological changes during F−0 larval instar. (**Z**) Magnified view of the newly formed region in (**O**).

Among dragonfly species (Anisozygoptera and Anisoptera), Aeshnidae larvae exhibited the relatively complicated expansion patterns in their compound eyes as previously described^14–16^. During stage 1, the compound eyes expanded gradually toward the midline (Fig. 5A–D). During stage 2, apolysis-like phenomenon was observed from the anterior side to the posterior side of the compound eyes (Fig. 5E–L, black arrowheads; Fig. 5Y). This apolysis-like phenomenon was also observed, though faintly, in the compound eyes of some Libellulidae species (e.g. *C. servilia*) (Figs. 5O, S1; Table 1).

The expansion of the compound eyes was also observed commonly in dragonfly species other than Aeshnidae species (Fig. 5M–X). In some Libellulidae species, newly-formed regions of the compound eyes were clearly distinguishable morphologically (Fig. 5N–P,R–T, white arrowheads, Fig. 5Z). These regions gradually expanded from mid-stage 1 to stage 3 (Fig. 5M–T,Y,Z). Meanwhile, the boundary of the newly formed regions was less obvious in several dragonfly species including Libellulidae (ex. *Rhyothemis fuliginosa*), and thus cannot be regarded as common developmental indicators (Fig. 5U–X; Table 1).

**Figure 6:**
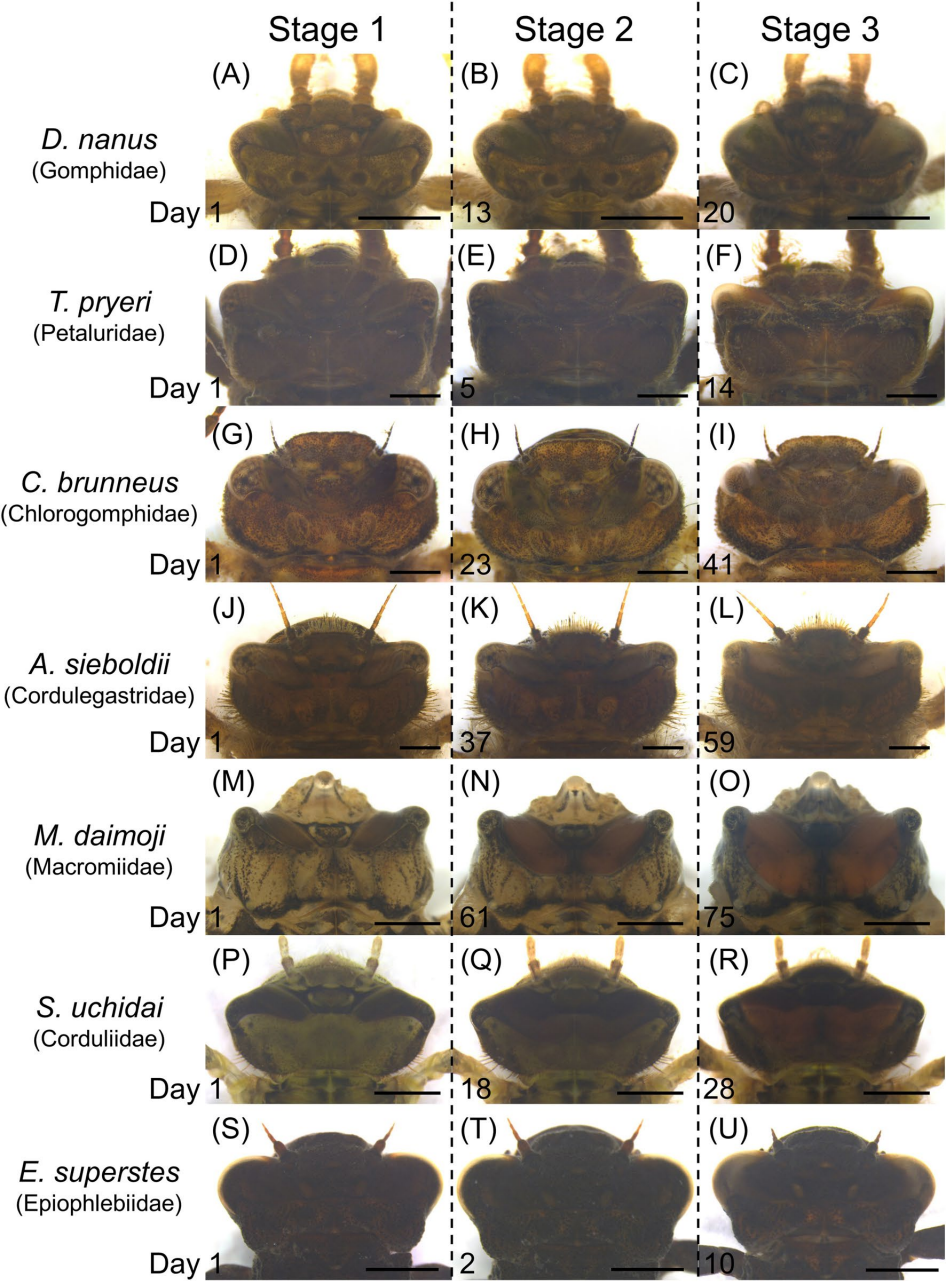
Morphological changes of the compound eyes during F−0 instar in Gomphidae, Petaluridae, Chlorogomphidae, Cordulegastridae, Macromiidae, Corduliidae, and Epiophlebiidae species. (**A**–**U**) Dorsal views of the heads. All scale bars show 2 mm. Species and family names are provided on the left side. The number shown on the lower left of each image indicates the number of days from the day of ecdysis or collection. (**A**, **D**, **G**, **J**, **M**, **S**) The first day of starting photographing. (**P**) The first day of stage 1. (**B**, **E**, **H**, **K**, **N**, **Q**, **T**) The first day of stage 2. (**C**, **F**, **I**, **L**, **O**, **R**) The first day of stage 3. (**U**) The day before adult emergence.

In Gomphidae, Petaluridae, Chlorogomphidae, Cordulegastridae, Macromiidae and Corduliidae species, the posterior expansion patterns of the compound eyes were similar to those in Libellulidae species (Fig. 6). It should be noted that the morphological changes of the compound eyes were difficult to observe in species and/or individuals with dark-colored heads (ex. *Epiophlebia superstes*, Fig. 6S–U), as reported for *Pseudothemis zonata* (Libellulidae)^19^. On the day before adult emergence, the color of the compound eyes resembled that of adult (Fig. 6U).

### Other morphological changes observed in F−0 instar larvae

**Figure 7:**
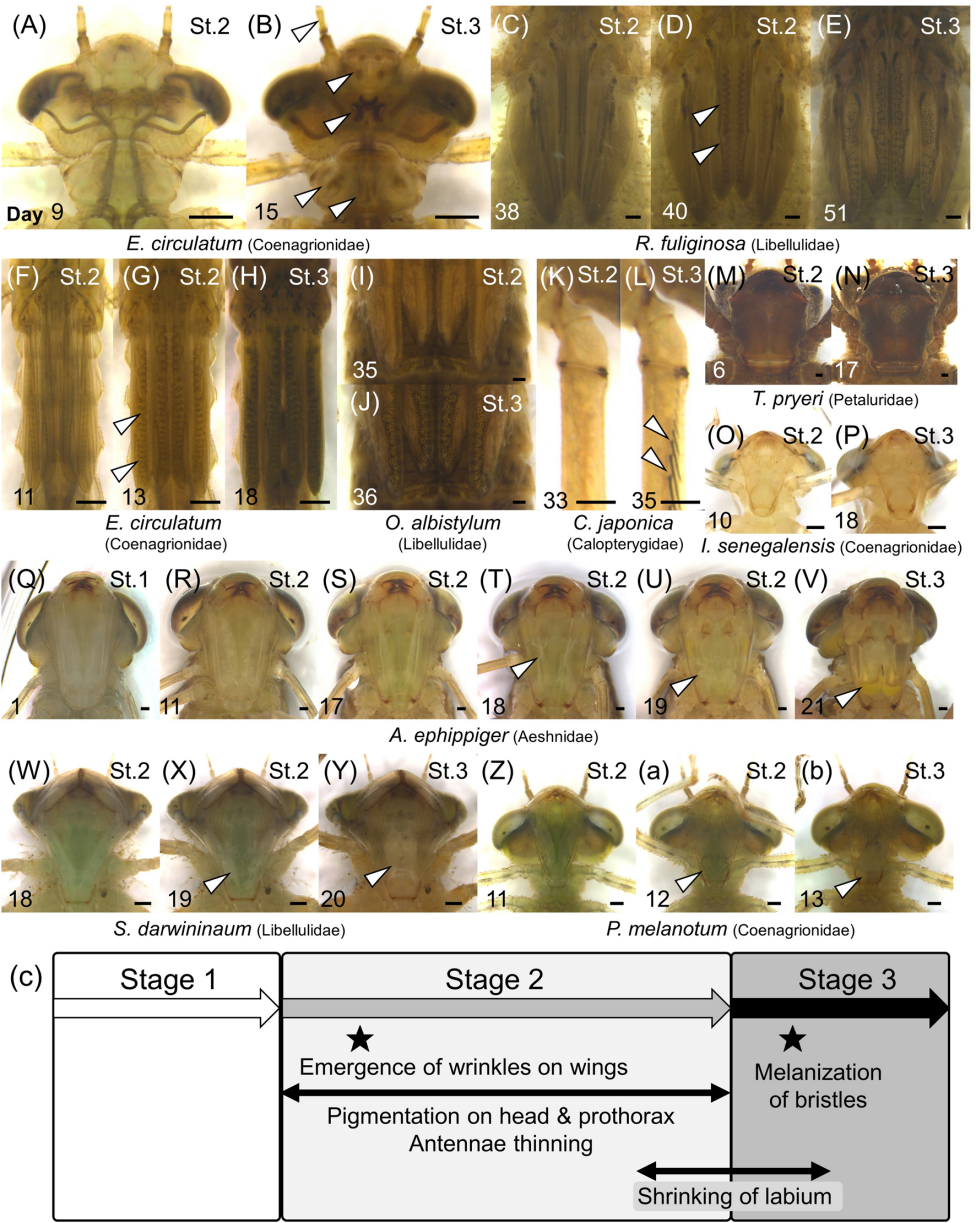
Morphological changes other than compound eyes during F−0 larval instar. The number on each photo indicates the number of days after ecdysis or starting photographing. All scale bars show 0.5 mm. Species and family names are indicated at the bottom. Developmental stage is shown on the upper right of each photo. (**A**, **B**) Dorsal views of the heads. White arrowheads indicate specific pigmentation that appeared during stage 2. (**C**–**J**) Magnified views of wing sheaths. White arrowheads indicate wrinkles in the wing sheaths. (**C**, **F**) The first day of stage 2. (**D**, **G**) The day on which the wrinkles on the wing sheaths appeared. (**E**, **H**) The first day of stage 3. (**K**–**L**) Magnified views of right foreleg. White arrowheads indicate melanized bristles. Adult emergence was observed on Day 37. (**M-b**) Ventral views of the heads. White arrowheads indicate the degraded larval labia. (**Q**) The first day of stage 1. (**M**, **O**, **R**). The first day of stage 2. (**T**, **W**, **Z**) Two days before the onset of stage 3. (**U**, **X**, **a**) One day before the onset of stage 3. (**Y**, **b**) The first day of stage 3. (**N**, **P**, **V**) The last day of stage 3. (**c**) Schematic diagrams on the timing of the morphological changes during F−0 larval instar.

We also summarized other morphological changes that were constantly observed within some specific Odonata lineages (Fig. 7; Table 1). In most damselfly species, specific markings appeared after stage 2 on their heads, prothoraces, around ocelli and mandibles (Fig. 7A,B; Table 1, white arrowheads), and thinning of antennae was observed during stage 2 (Fig. 7A,B, white arrowhead), as reported^12^.

Previous studies regarded the wrinkles on the wing sheaths (described as “accordion-like”^15^) as an indicator of metamorphosis in Odonata^10,11,15,18^. In general, the wrinkles appeared a few days after the onset of stage 2 (Fig. 7C). While the wrinkles on the wings were clearly visible in many Odonata species (Fig. 7D,G, white arrowheads, Table 1), they were less obvious in some species (ex. *Orthetrum albistylum*: Fig. 7I–J). Just before adult emergence at the end of stage 3, the bristles on the body surface were melanized (Fig. 7K–L), although this change was often masked by dark pigmentation of the body surface.

The degradation of larval labium, or shrinkage of tissues within the larval prementum, has been also used as an indicator of metamorphosis in Odonata^10,11,14,16^. We photographed the daily changes in the larval labium of 18 larvae representing 7 species (Table 1; Fig. S3). The degradation of larval labium was more prominent in dragonfly species (Anisoptera) (Fig. 7Q–Y) than in damselfly species (Zygoptera) (Fig. 7O–P,Z-b). We confirmed that the degradation of larval labium occurred from late stage 2 to stage 3 (Fig. 7Q-c, white arrowheads). It should be noted that the changes in larval labium were not prominent in some species like *Tanypteryx pryeri* (Petaluridae, Fig. 7M–N) and *I. senegalensis* (Coenagrionidae, Fig. 7O–P).

## Conclusion and perspective

In this study, we described the daily morphological changes of F−0 and/or F−1 instar larvae, using 158 individuals of 49 Odonata species. Based on the morphology of wing sheaths, we propose that F−0 larval instar could be classified into three developmental stages for most of the examined Odonata species.

The order Odonata is among the most ancestral insects with metamorphosis and are important in studying the origin of insect metamorphosis. However, even the dynamics of insect hormone titers (e.g. ecdysteroids) during F−0 instar has not been described in detail^23,24^, which is ascribed to, at least partly, the lack of developmental descriptions in Odonata. The comprehensive and detailed information provided in this study will enable the precise developmental staging of larval-adult metamorphosis across diverse Odonata species and will advance the comparative analysis of the physiological and molecular mechanisms of metamorphosis with other insects toward understanding the evolution of metamorphosis.

Recently, we established a gene knockdown method by electroporation-mediated RNAi in Odonata^25,26^. Considering that precise larval staging is critical for effective gene knockdown^25,26^, this study will facilitate the identification of appropriate developmental stages for RNAi, thereby facilitating the molecular genetic studies on various topics in Odonata.

## Supplementary Information

Supplementary Legends.

Supplementary Figure S1.

Supplementary Figure S2.

Supplementary Figure S3.

Supplementary Figure S4.

Supplementary Figure S5.

Supplementary Table S1.
