# Supplementary material for: Comprehensive comparative morphology and developmental staging of final instar larvae toward metamorphosis in the insect order Odonata
Source: Sci Rep. 2021 Mar 4;11:5164. doi: 10.1038/s41598-021-84639-2 (PMC7970851; doi:10.1038/s41598-021-84639-2)
Supplement: Supplementary file 1 — Supplementary Legends. [file 41598_2021_84639_MOESM1_ESM.docx]

Fig. S1

All the adjusted photos of F-0 instar larvae taken in this study. Individual data are shown in Table S1.

Fig. S2

All the adjusted photos of F-1 instar larvae taken in this study. Individual data are shown in Table S1.

Fig. S3

All the adjusted photos of the ventral heads focusing on the shrinking larval labium.

Fig. S4

Examples of enlarged views before and after entering each stage. (A) *Calopteryx japonica* (No. 4-1). (B) *Anax parthenope* (No. 23-1).

Fig. S5

Durations of stage 2 and stage 3 of observed larvae. Duration of stage 1 is not included because we started to photograph in the middle of stage 1 for most individuals of river dwelling species. Error bars show standard deviations. Abbreviations: Lest, Lestidae; Calo, Calopterygidae; Plat, Platycnemididae; Coen, Coenagrionidae; Aesh, Aeshnidae; Gomp, Gomphidae; Peta, Petaluridae; Chlo, Chlorogomphidae; Cordule, Cordulegastridae; Macr, Macromiidae; Corduli, Corduliidae; Libe, Libellulidae.

Table S1

Data of all Odonata individuals whose daily photos were taken in this study. (*1) Unidentifiable because we started to photograph from the middle of the stage. (*2) We could not identify the morphological stage due to very dark pigmentation on the body surface.
