# Supplementary material for: Comprehensive comparative morphology and developmental staging of final instar larvae toward metamorphosis in the insect order Odonata
Source: Sci Rep. 2021 Mar 4;11:5164. doi: 10.1038/s41598-021-84639-2 (PMC7970851; doi:10.1038/s41598-021-84639-2)

Comprehensive comparative morphology and developmental staging of final instar larvae toward metamorphosis in the insect order Odonata

Genta Okude, Takema Fukatsu, Ryo Futahashi

Figure S1

All the adjusted photos of F-0 instar larvae taken in this study.
Individual data are shown in Table S1.

1-1 *Lestes sponsa* (1/1)

2 mm

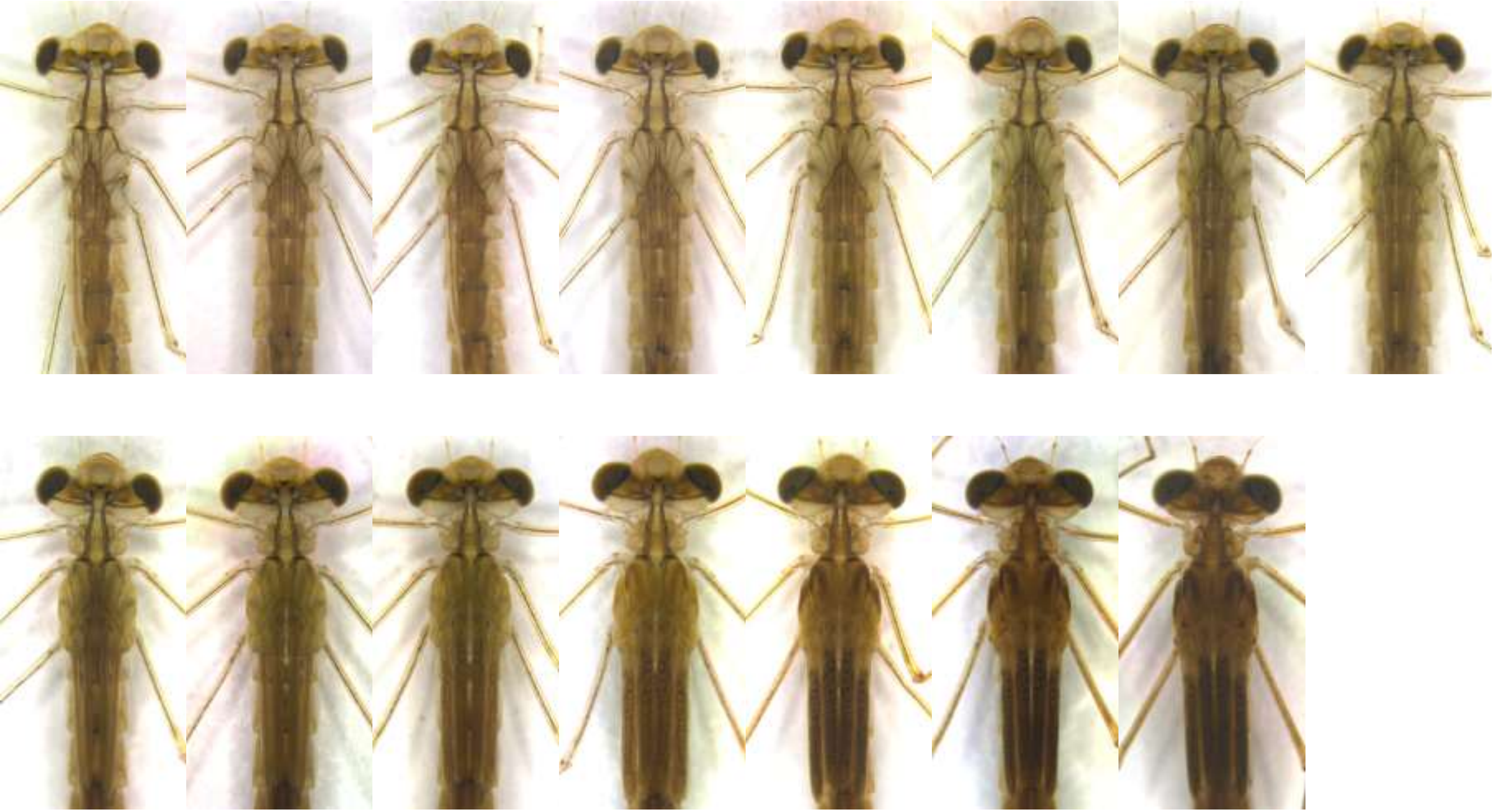

2-1 *Lestes temporalis* (1/1)

2 mm

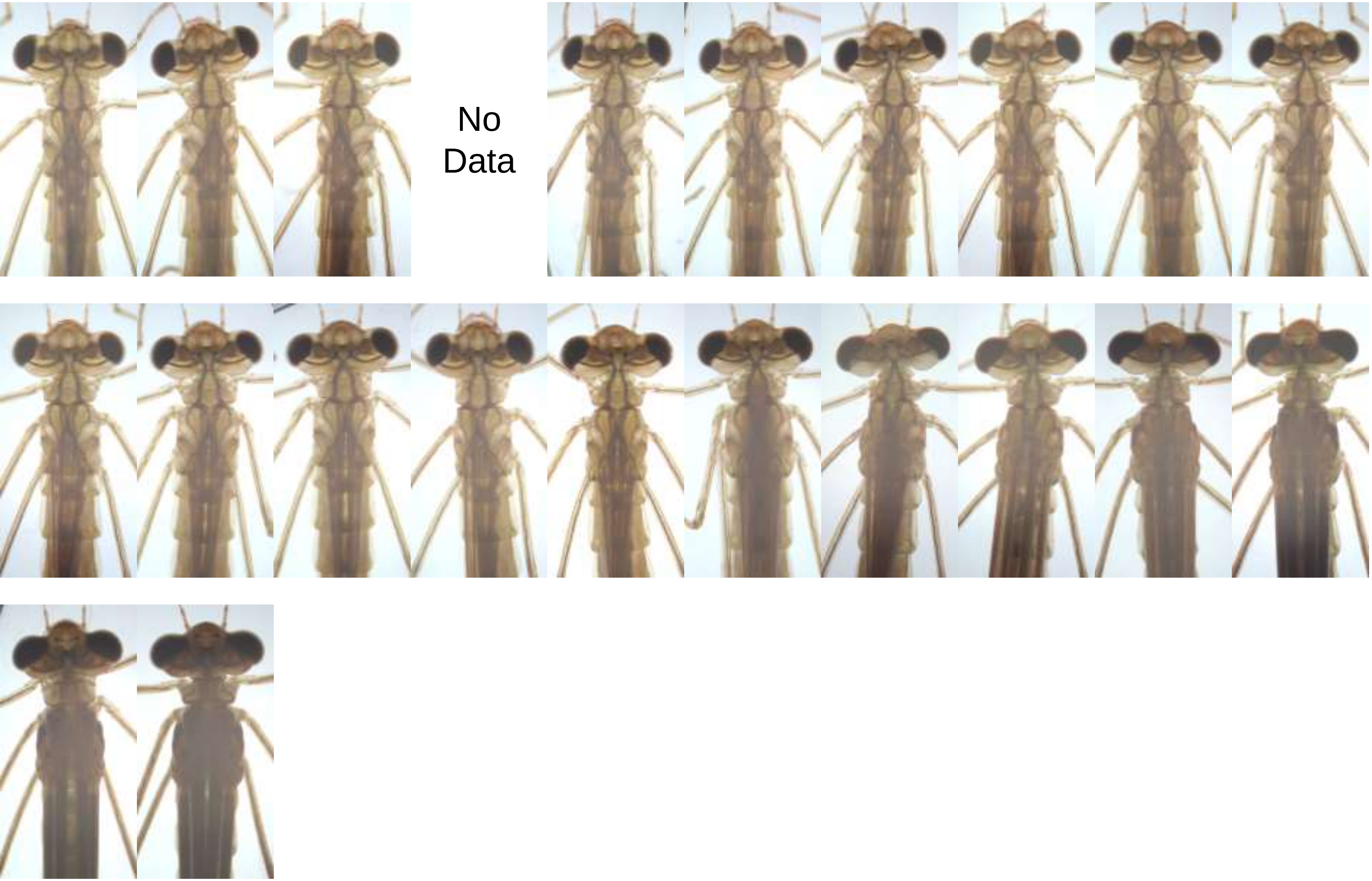

2-2 *Lestes temporalis* (1/1)

2 mm

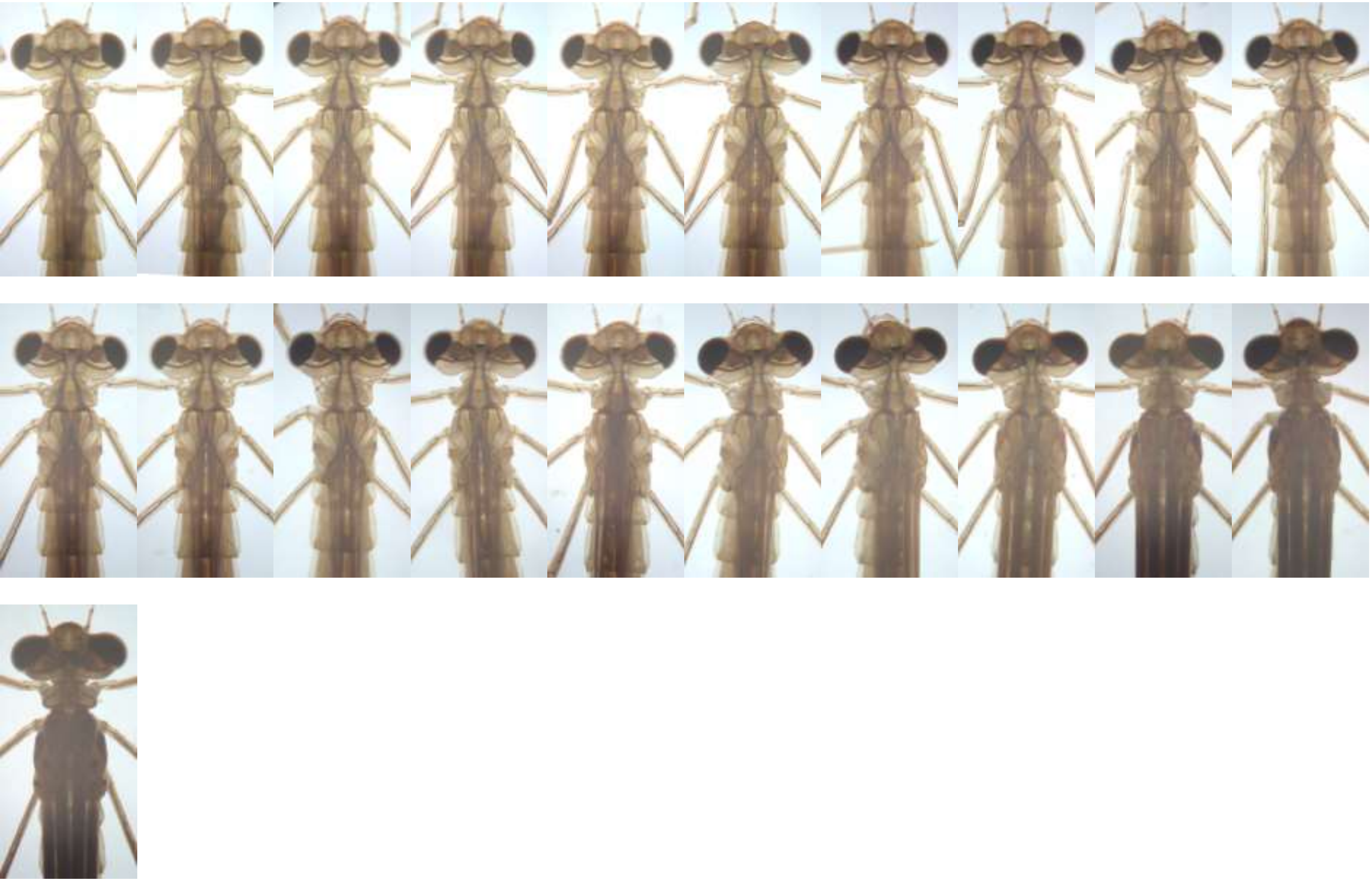

2-3 *Lestes temporalis* (1/1)

2 mm

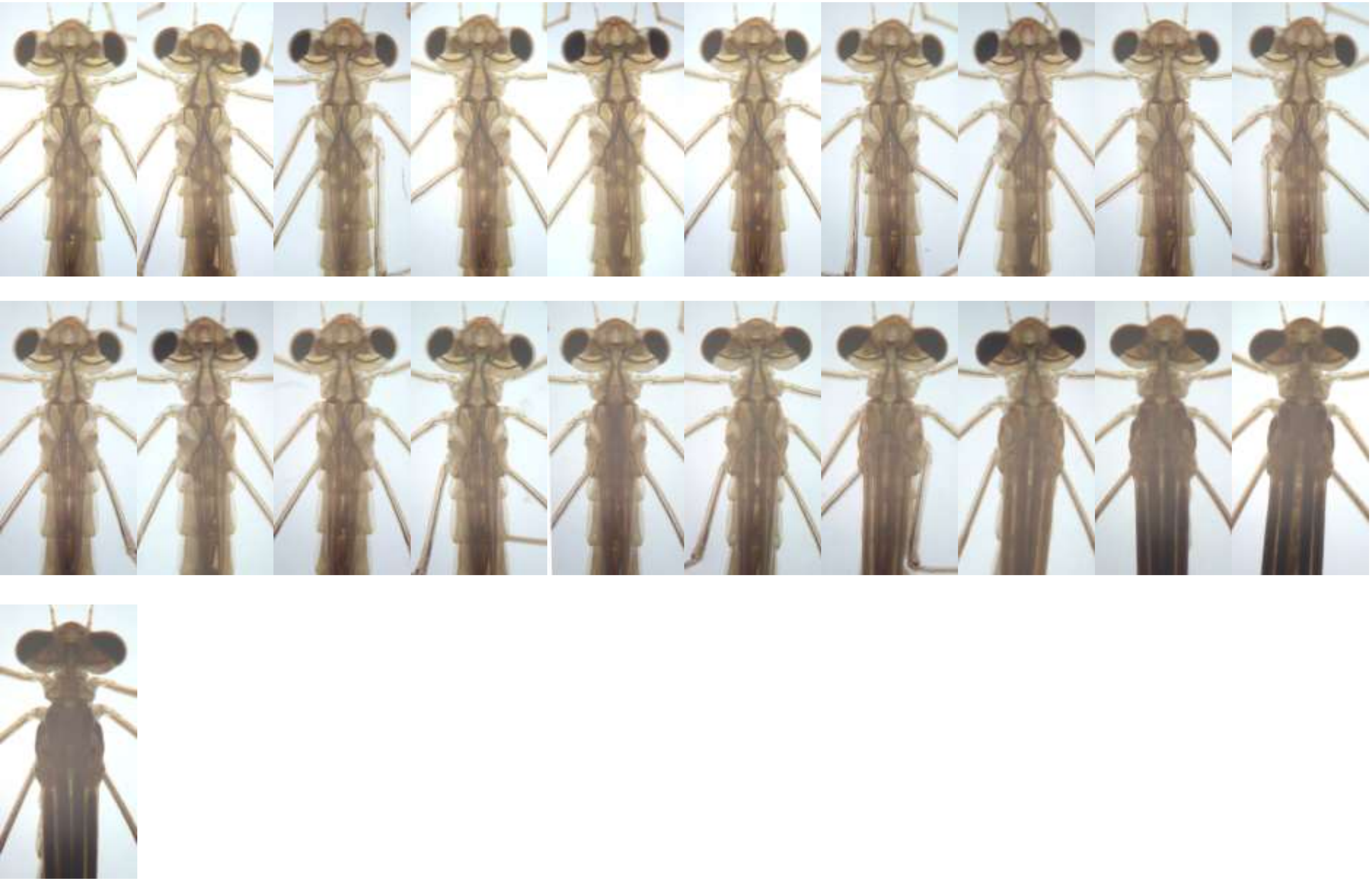

2-4 *Lestes temporalis* (1/1)

2 mm

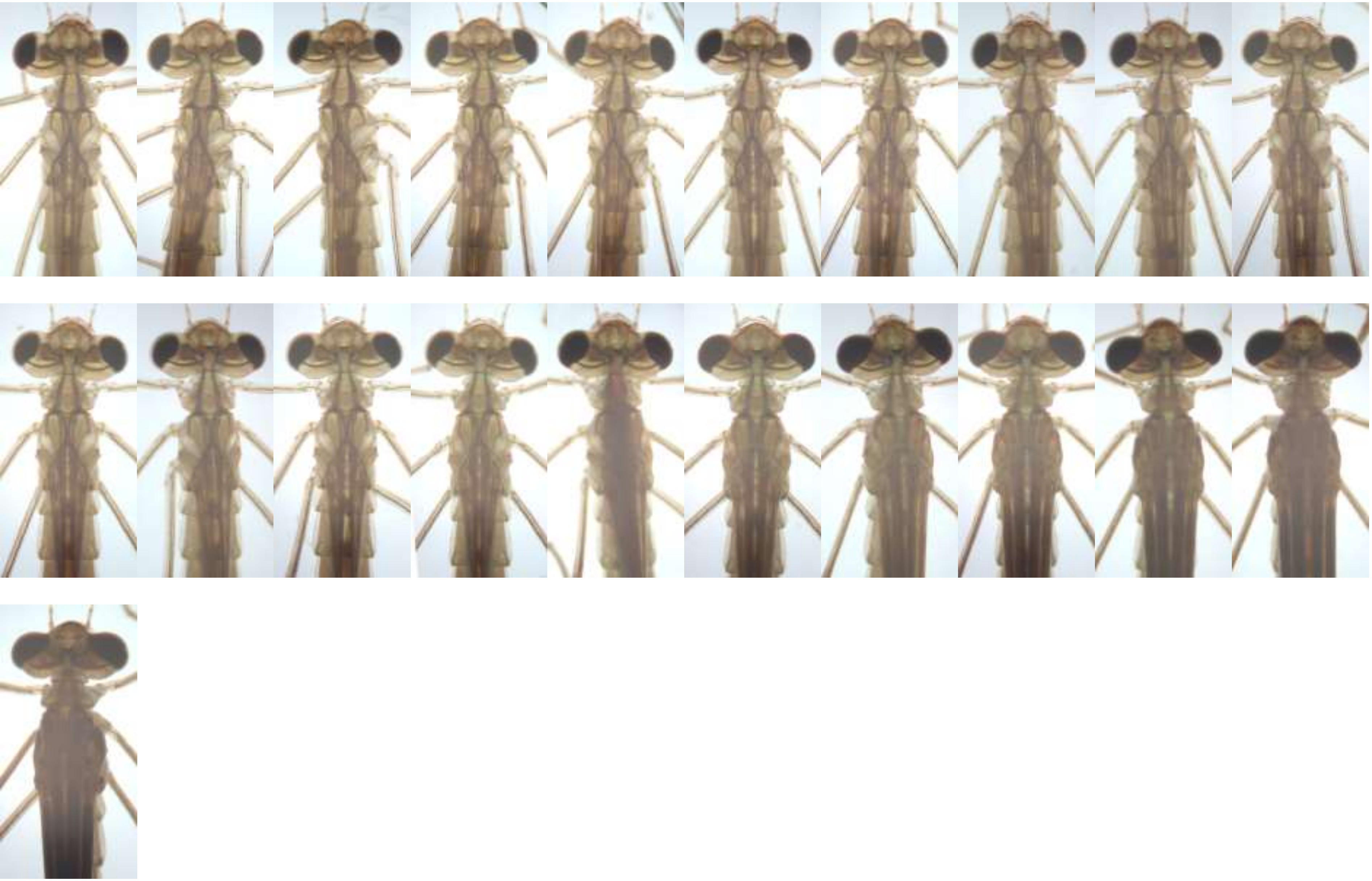

2-5 *Lestes temporalis* (1/1)

2 mm

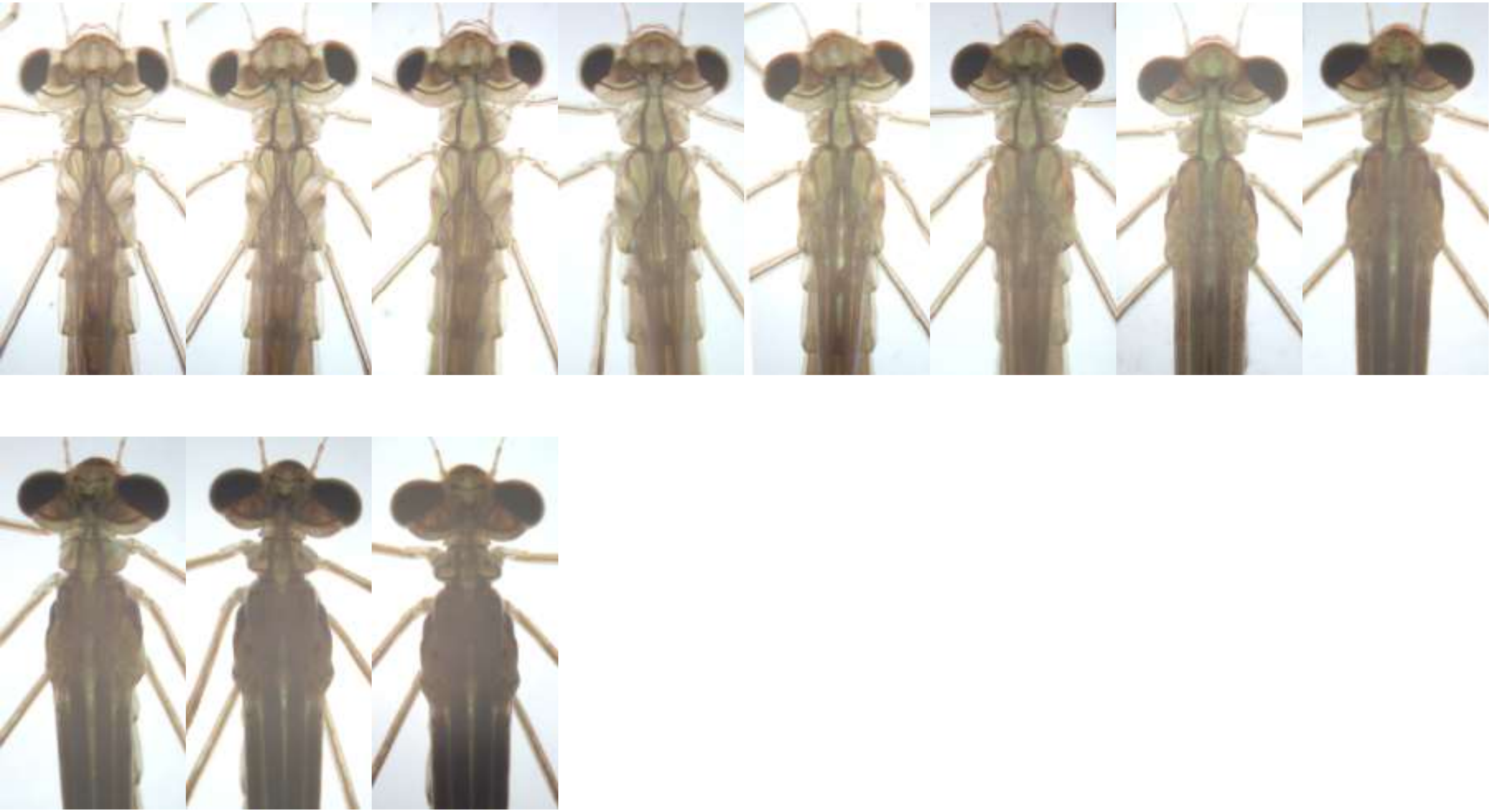

3-1 *Mnais costalis* (1/1)

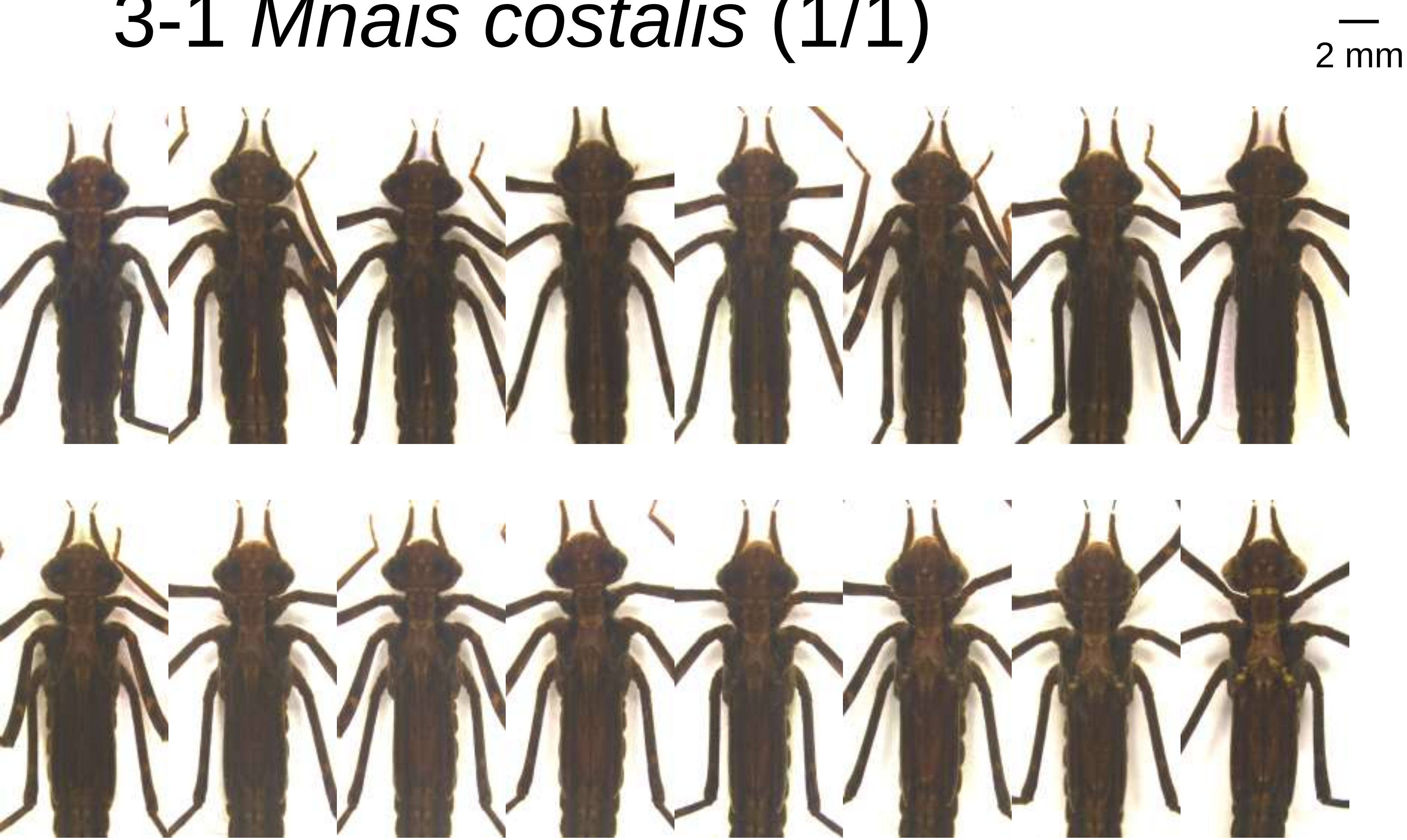

3-2 *Mnais costalis* (1/1)

2 mm

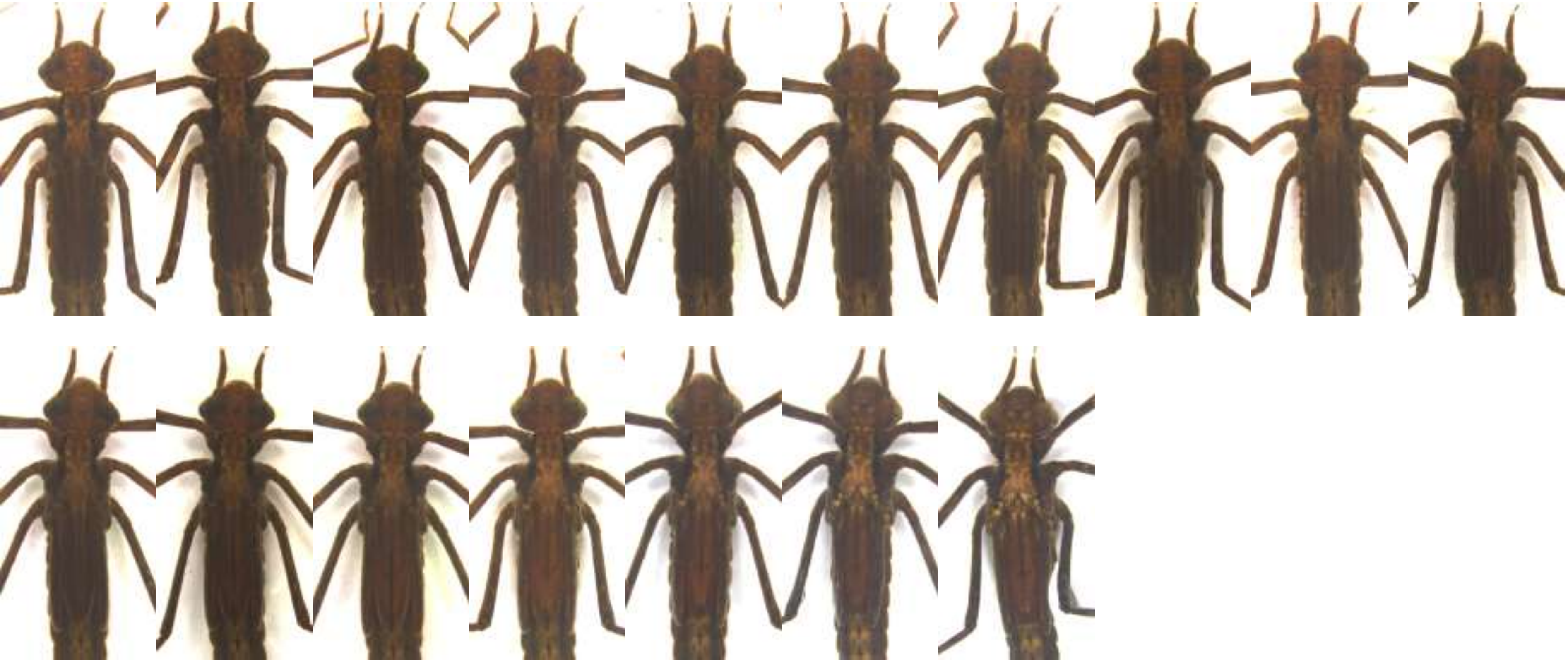

3-3 *Mnais costalis* (1/1)

2 mm

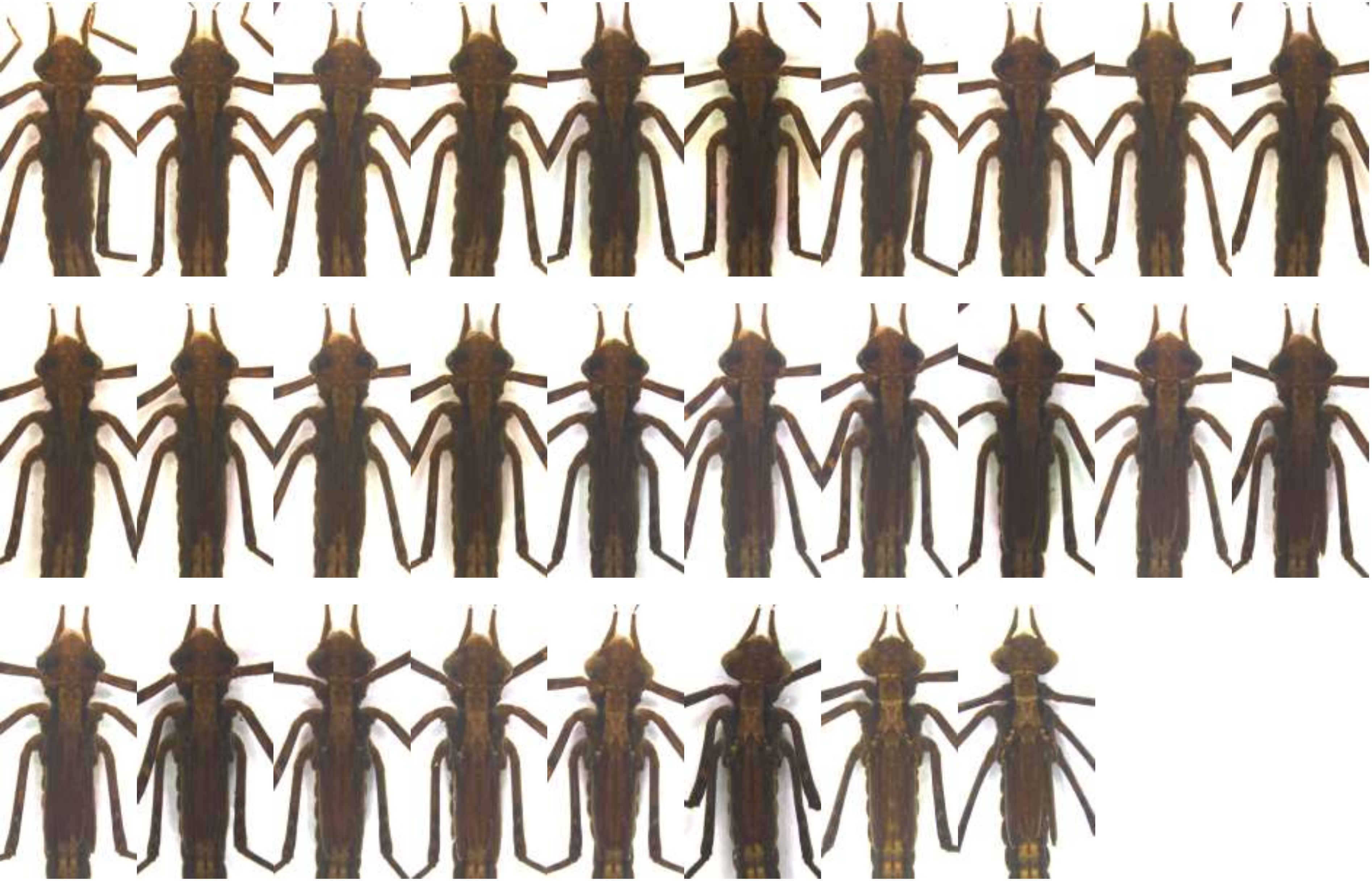

3-4 *Mnais costalis* (1/1)

2 mm

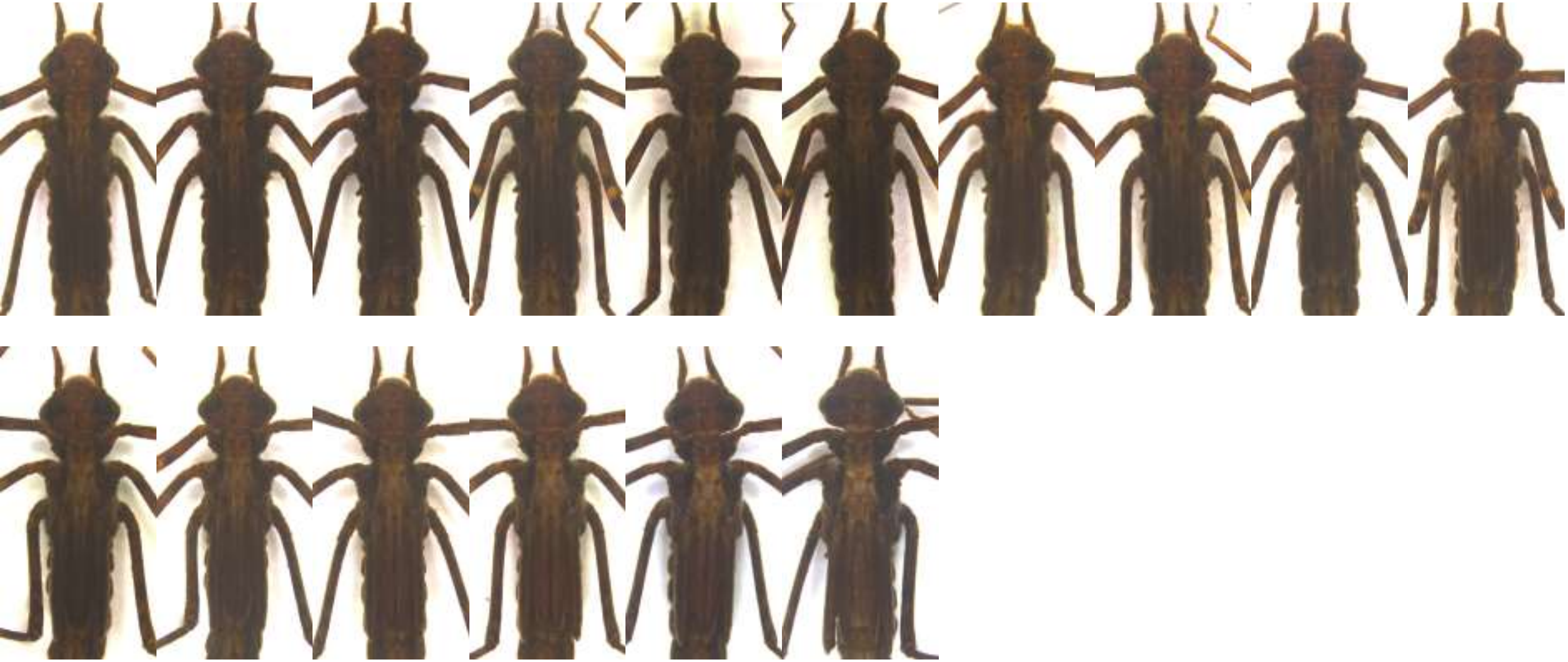

3-5 *Mnais costalis* (1/1)

2 mm

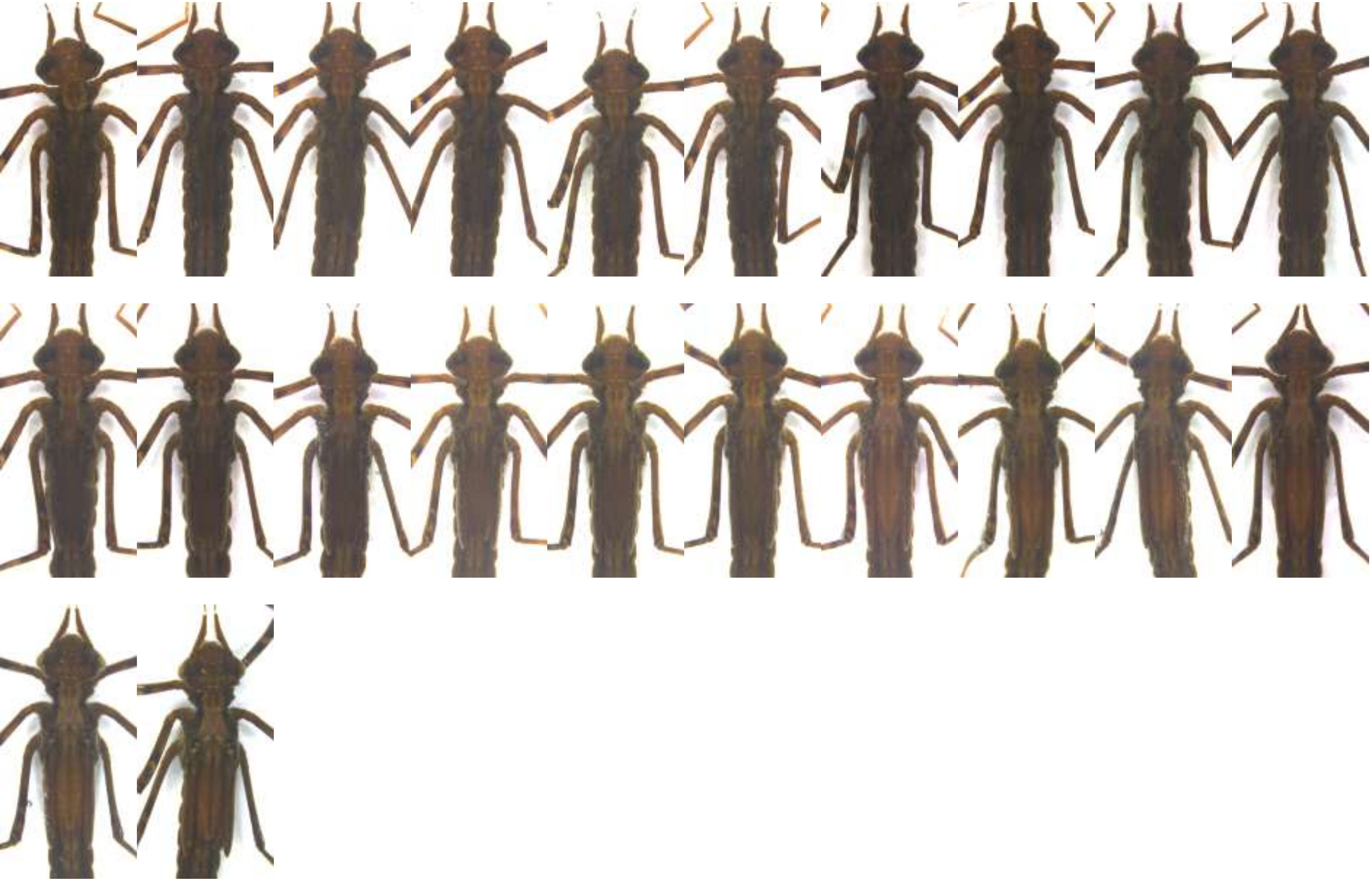

3-6 *Mnais costalis* (1/1)

2 mm

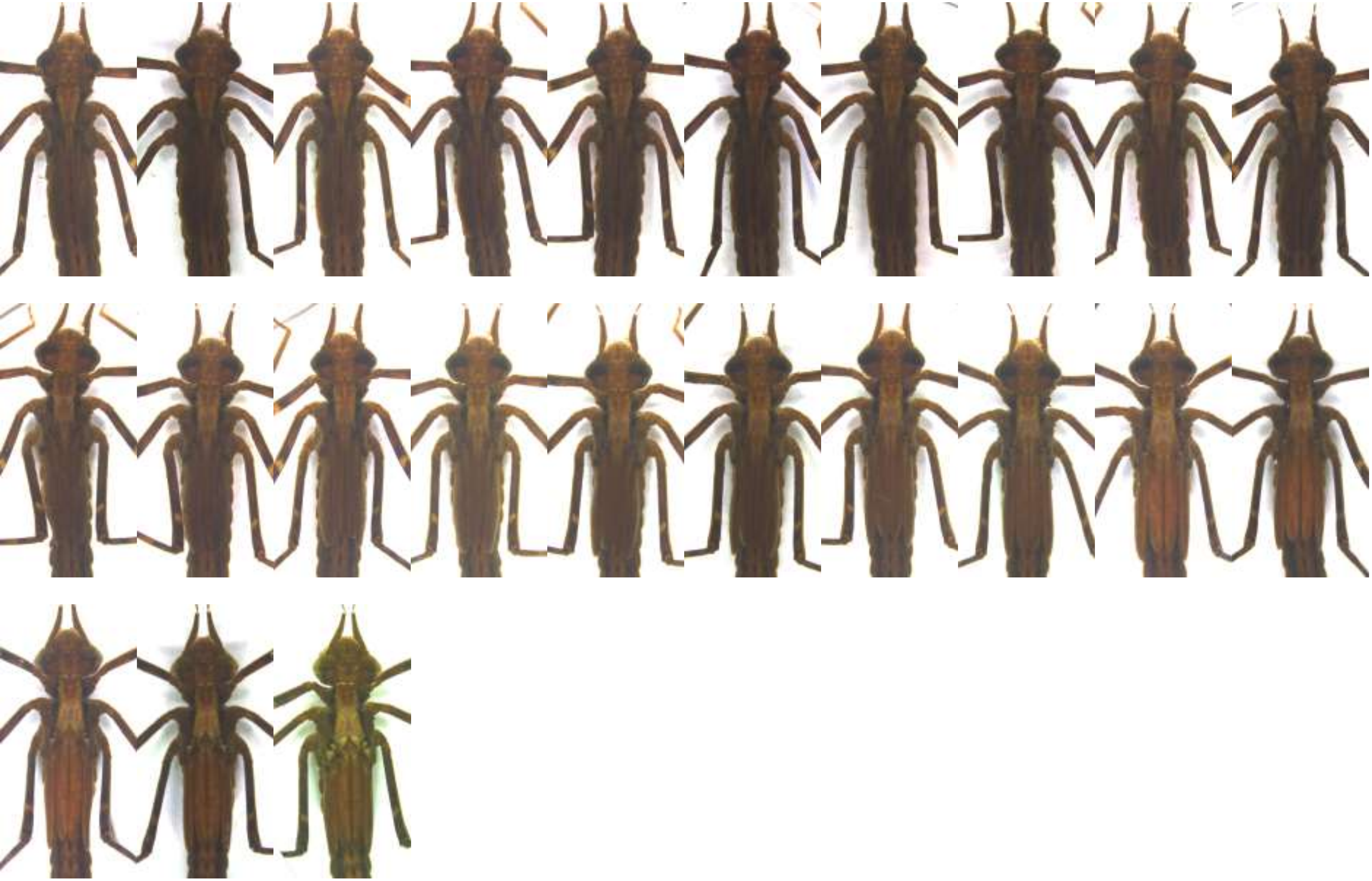

4-1 *Calopteryx japonica* (1/2)

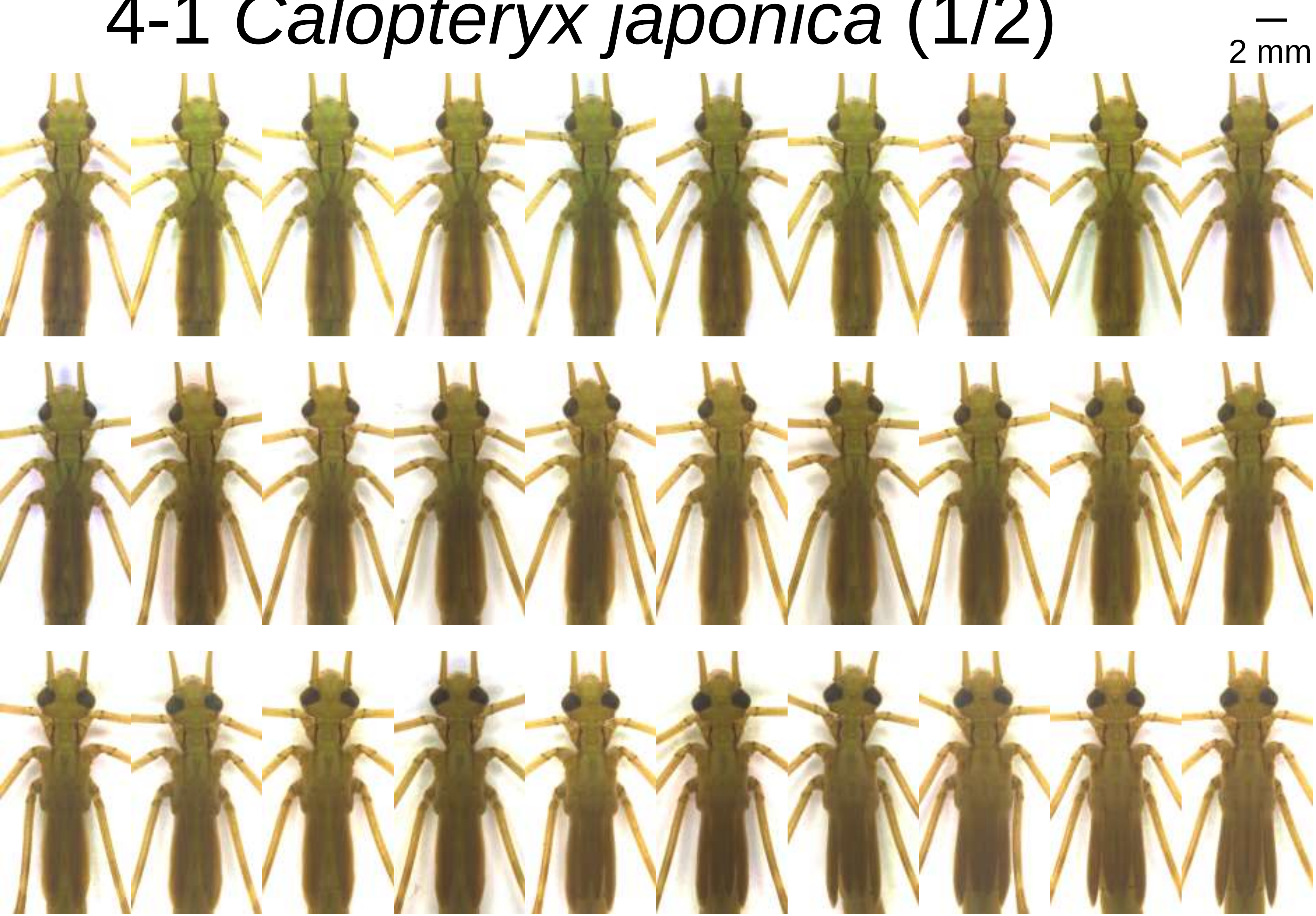

4-1 *Calopteryx japonica* (2/2)

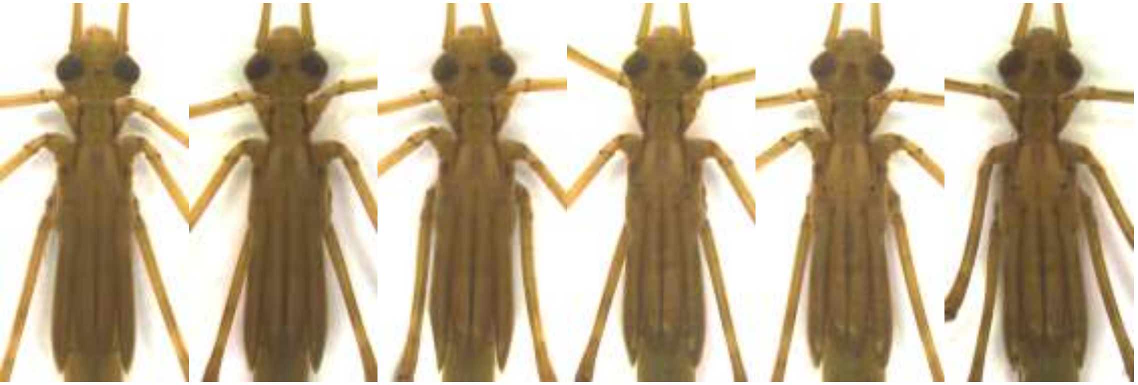

5-1 *Rhipidolestes hiraoi* (1/2)

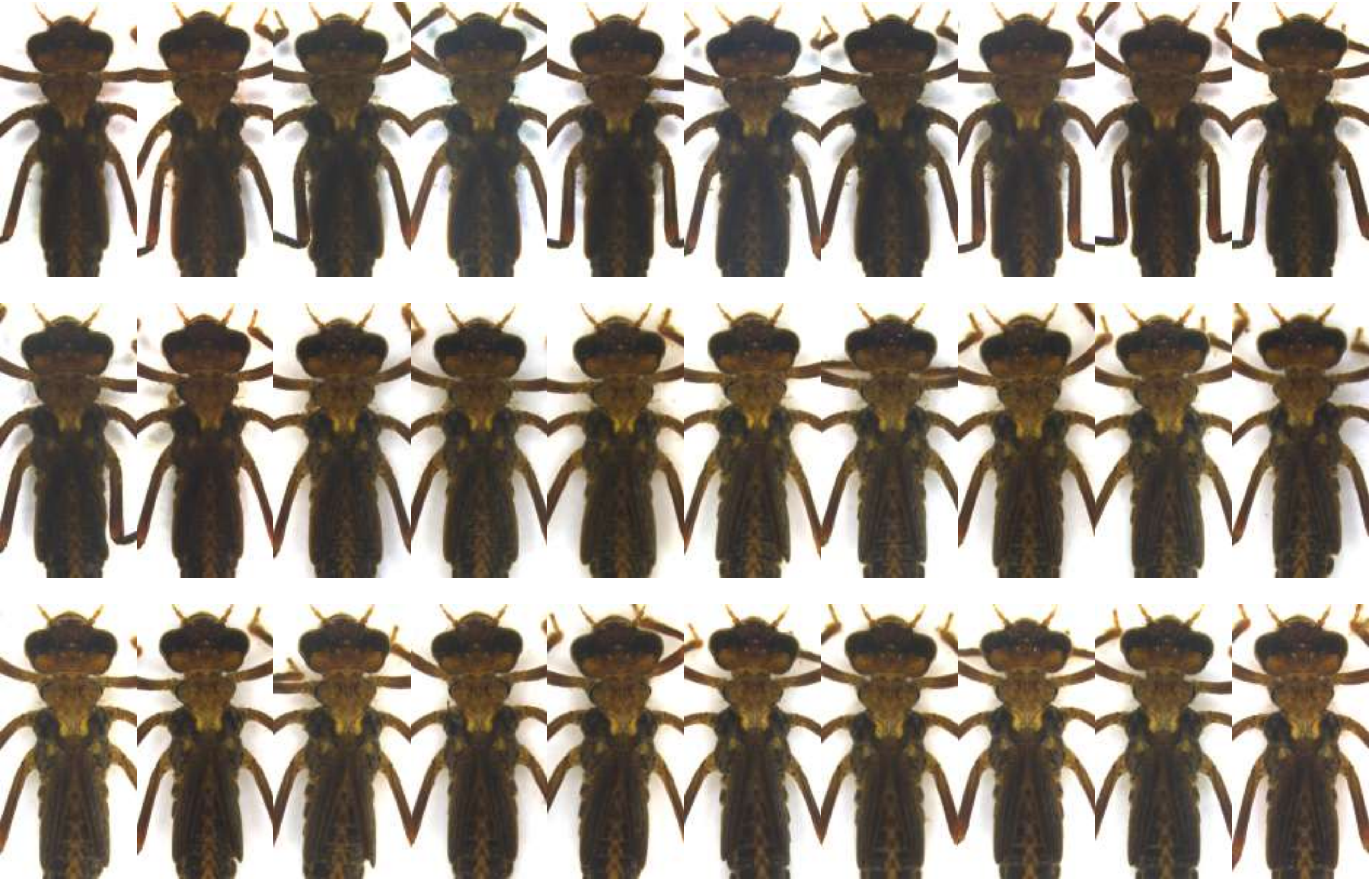

5-1 *Rhipidolestes hiraoi* (2/2)

2 mm

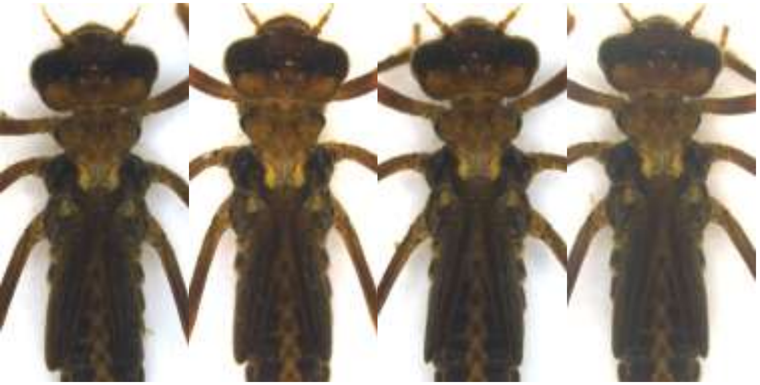

5-2 *Rhipidolestes hiraoi* (1/1)

2 mm

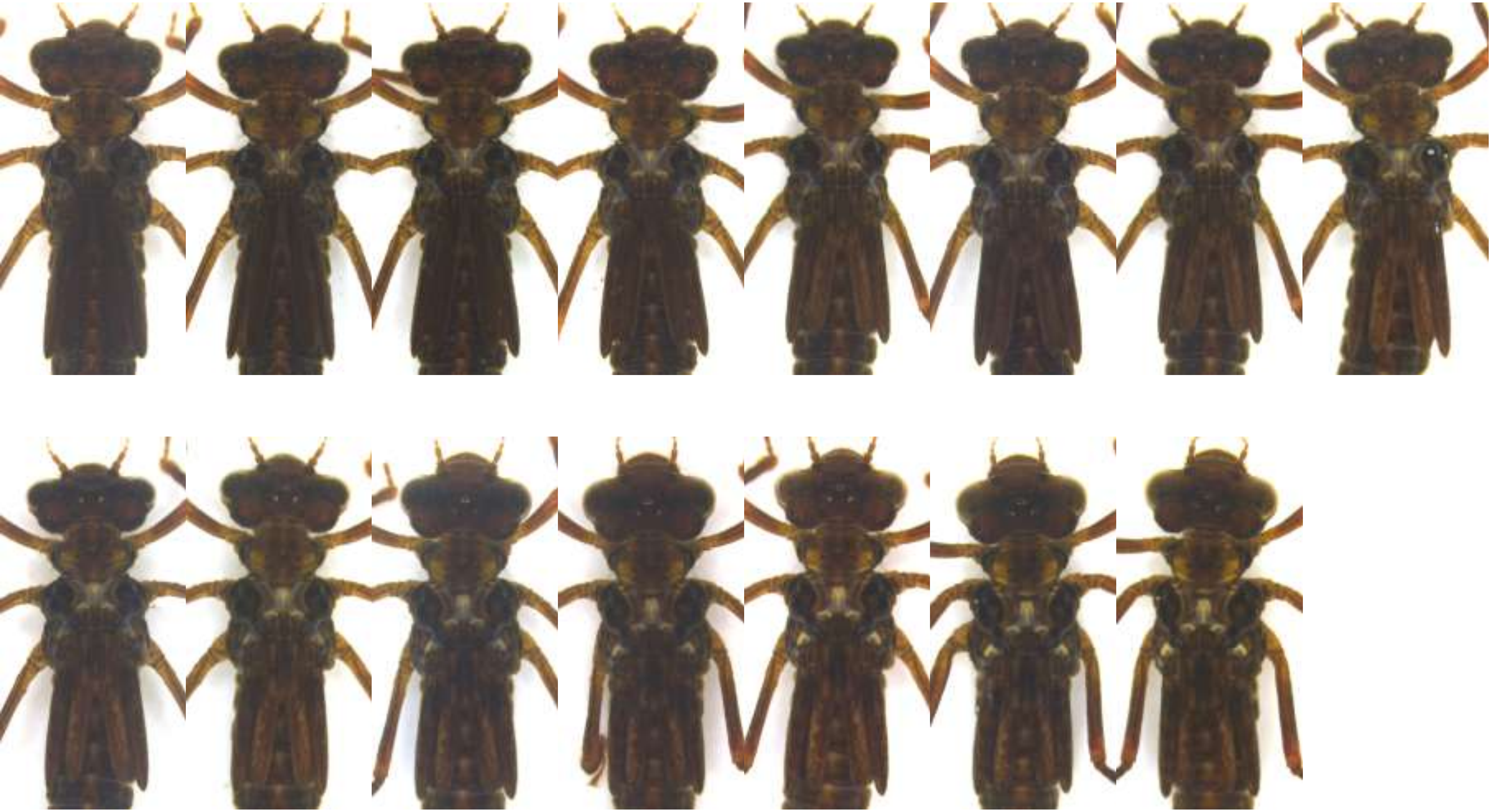

5-3 *Rhipidolestes hiraoi* (1/1)

2 mm

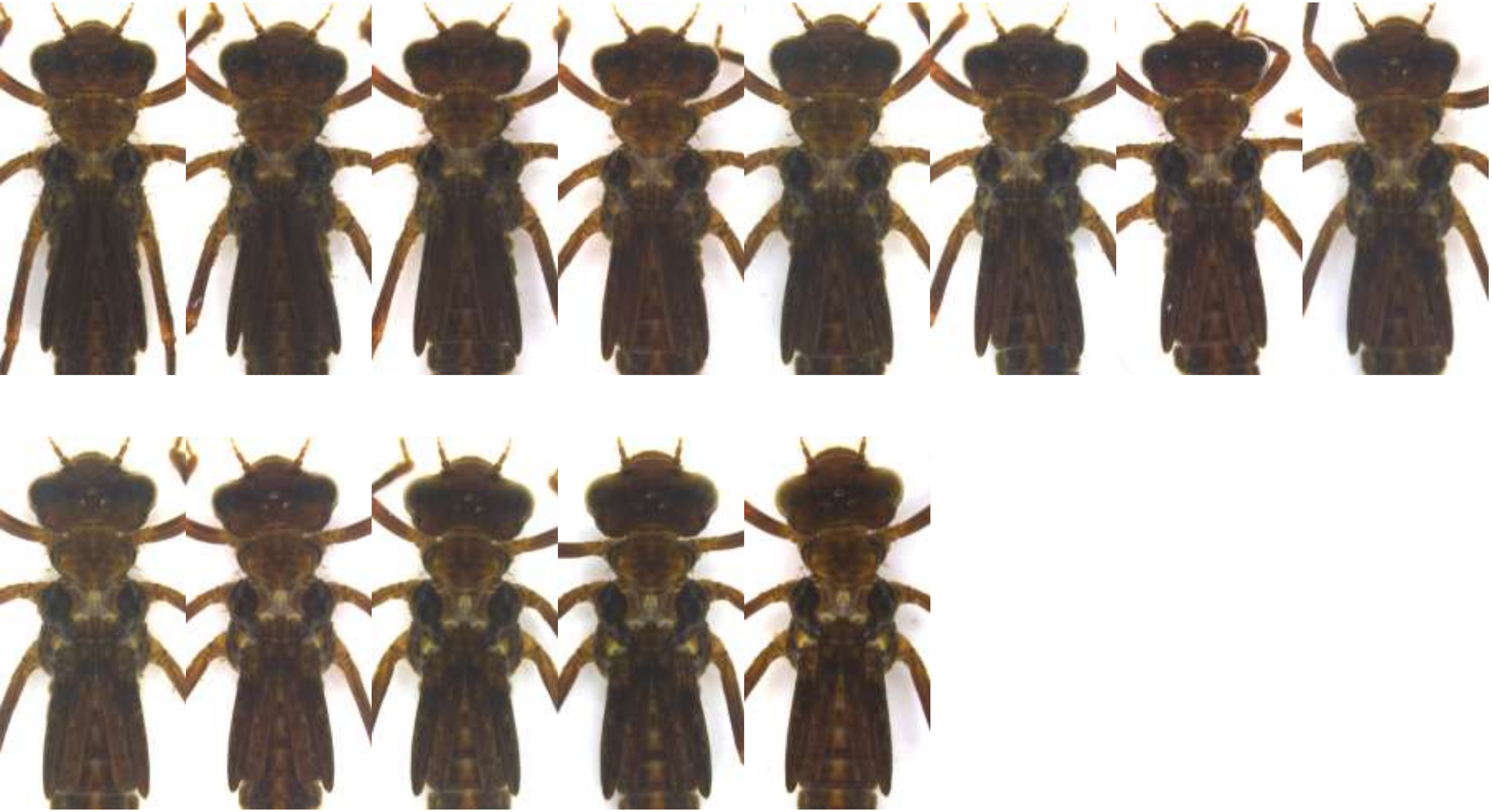

6-1 *Pseudocopera annulata* (1/2)

2 mm

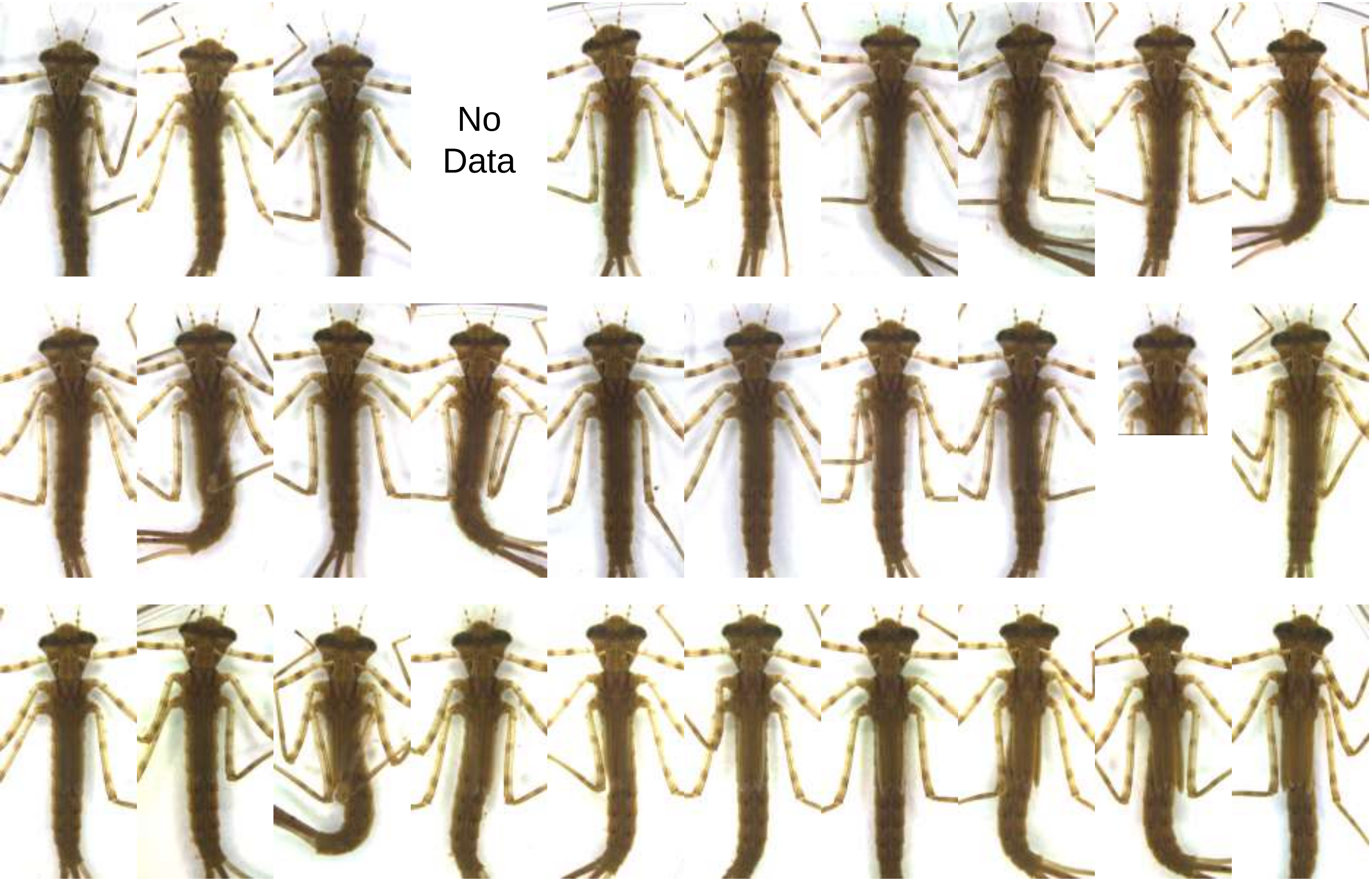

6-1 *Pseudocopera annulata* (2/2)

2 mm

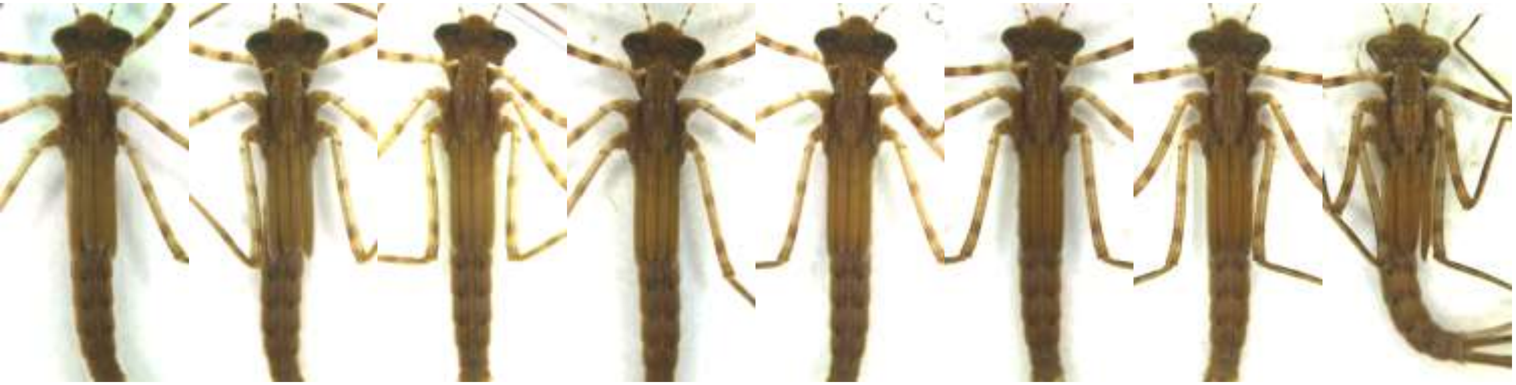

6-2 *Pseudocopera annulata* (1/1)

2 mm

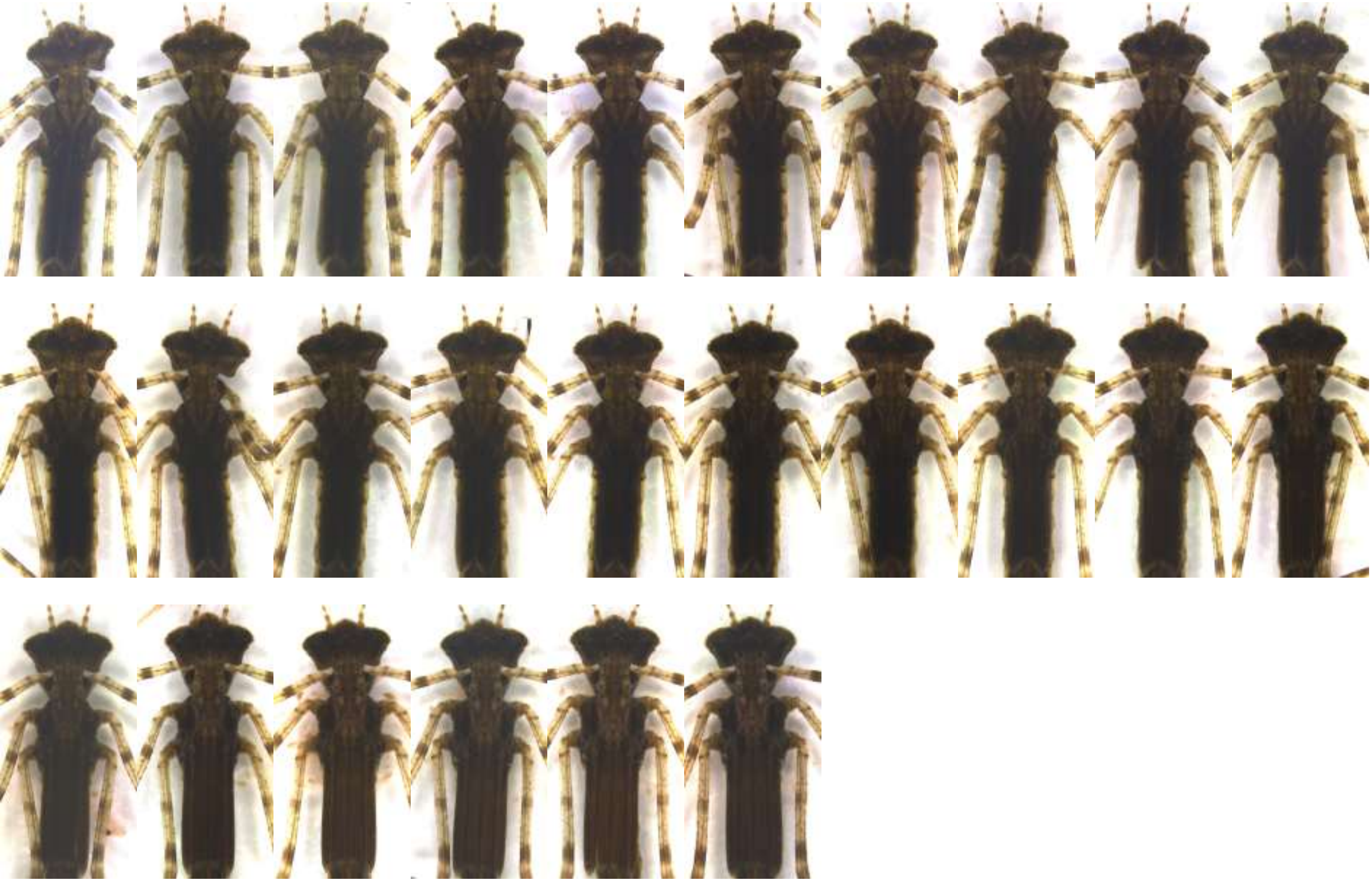

6-3 *Pseudocopera annulata* (1/2)

2 mm

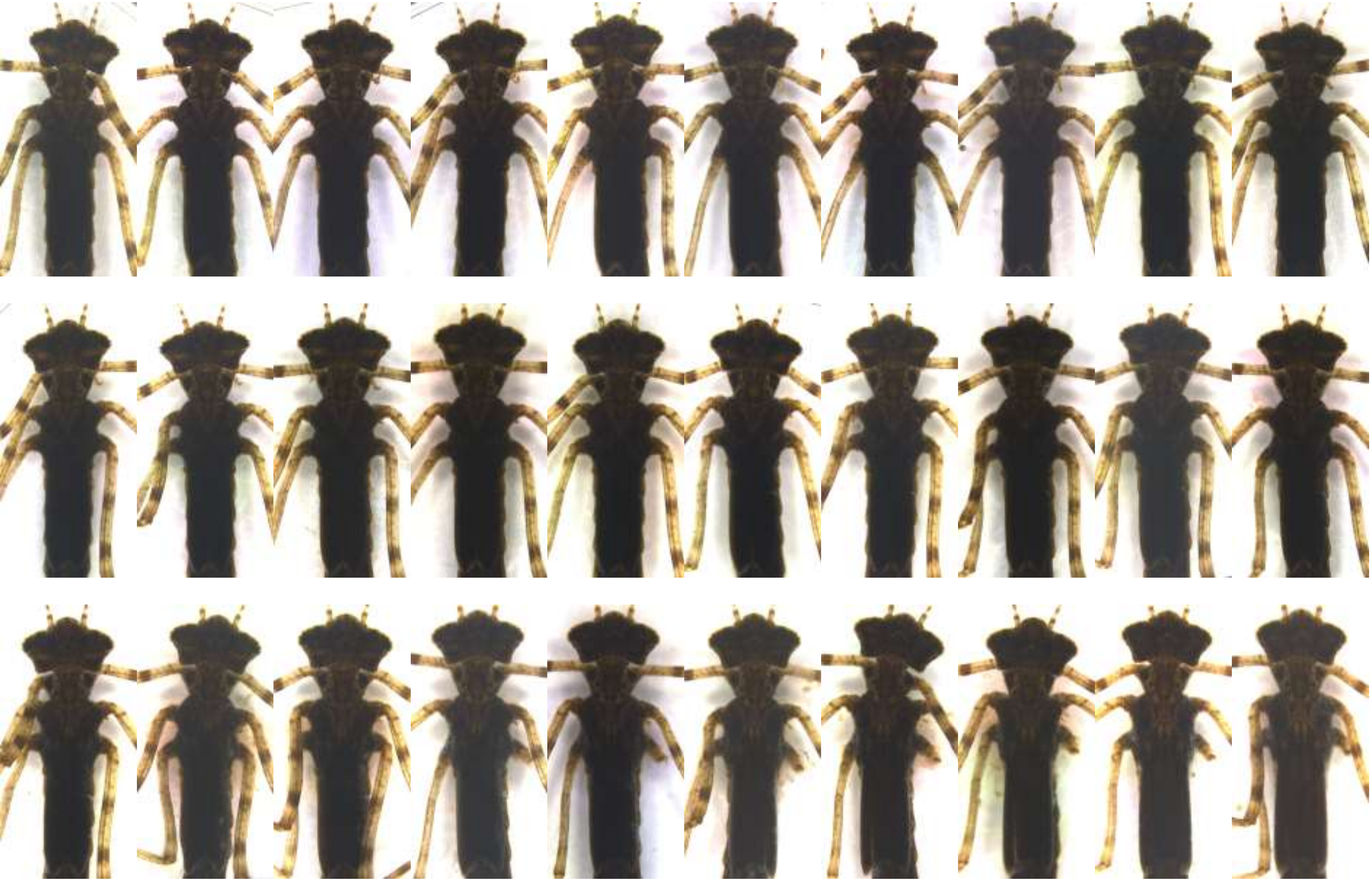

6-3 *Pseudocopera annulata* (2/2)

2 mm

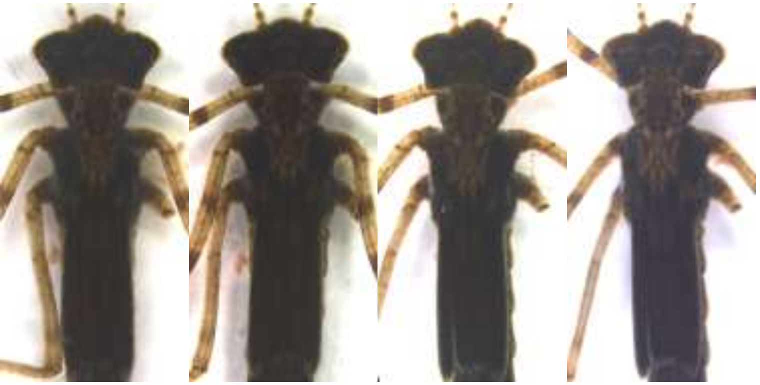

6-4 *Pseudocopera annulata* (1/1)

2 mm

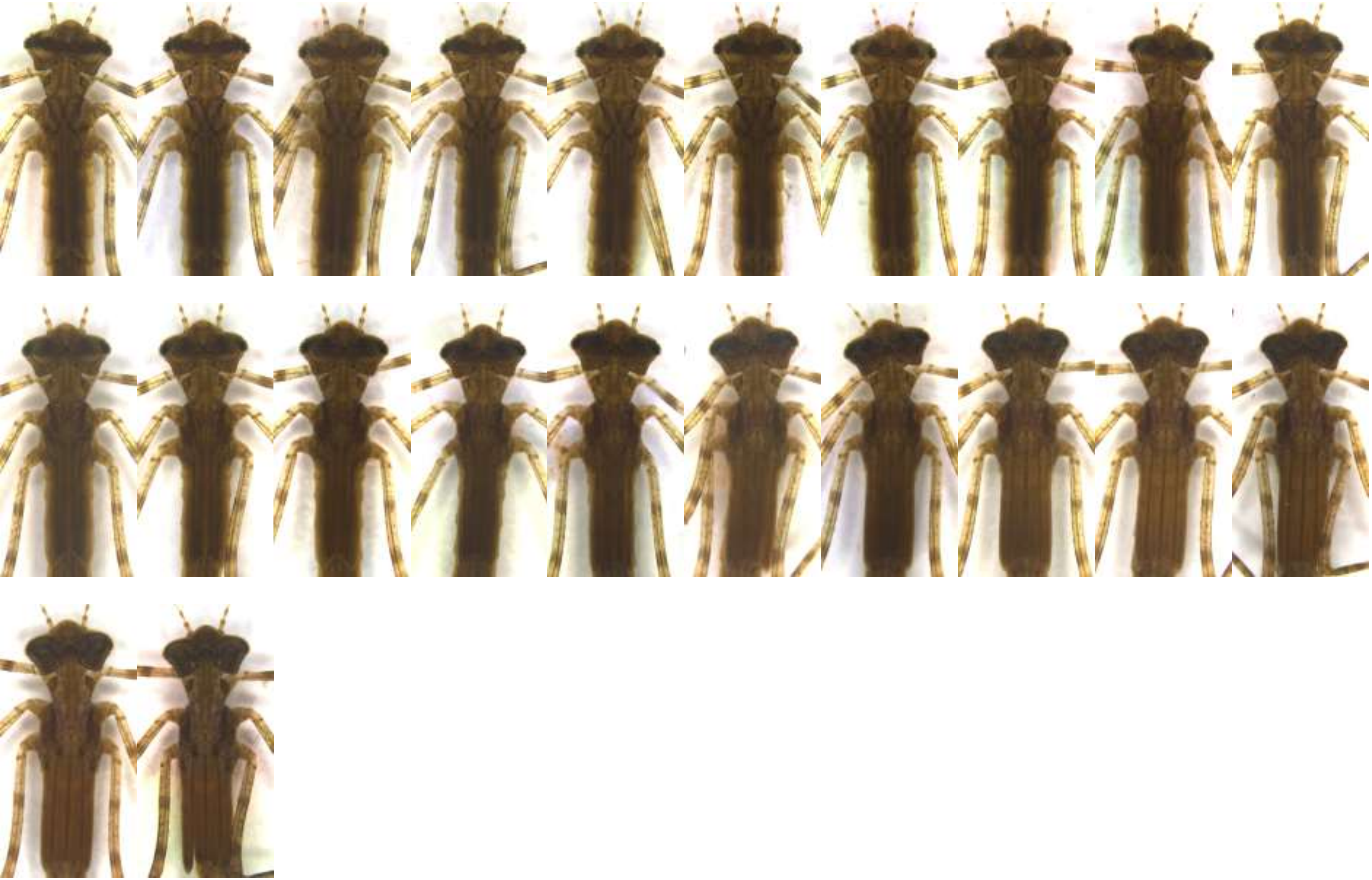

7-1 *Paracercion calamorum* (1/1)

2 mm

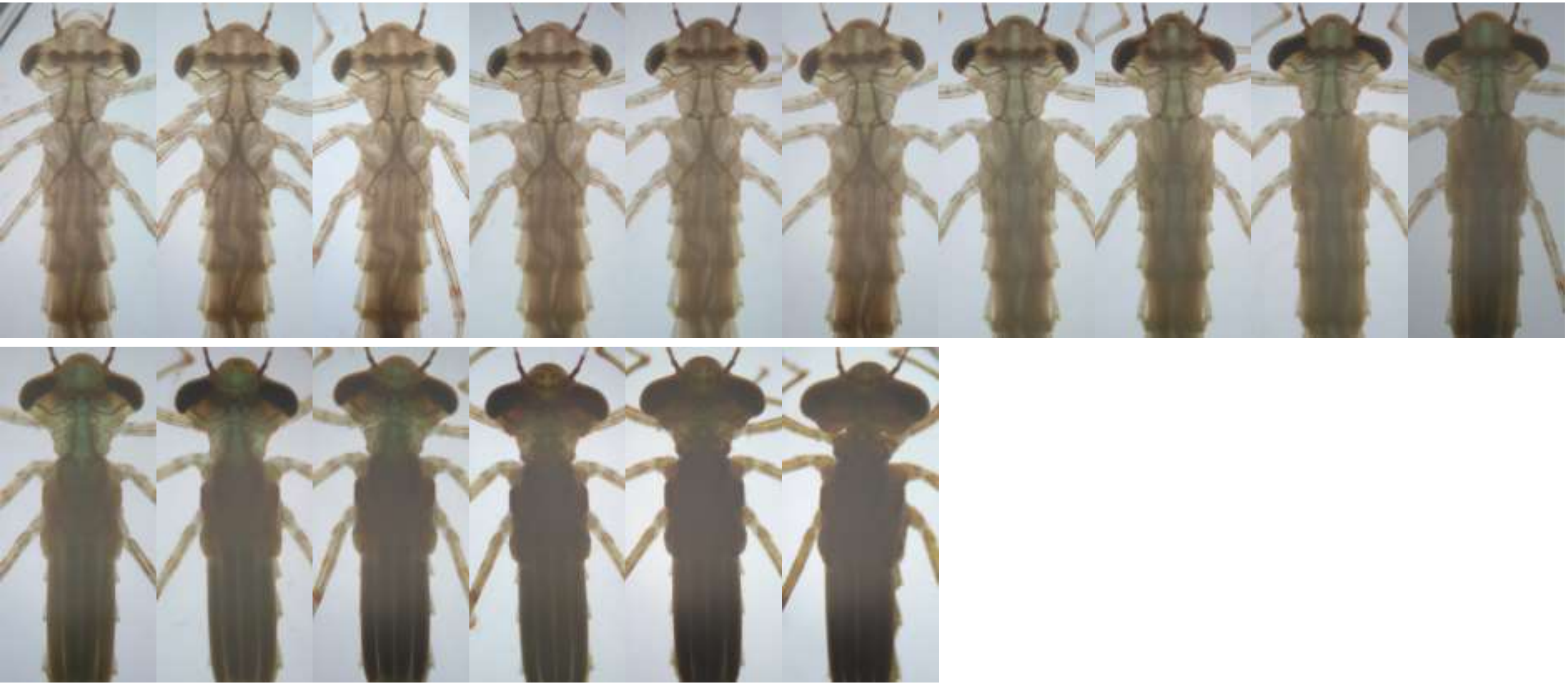

7-2 *Paracercion calamorum* (1/1)

2 mm

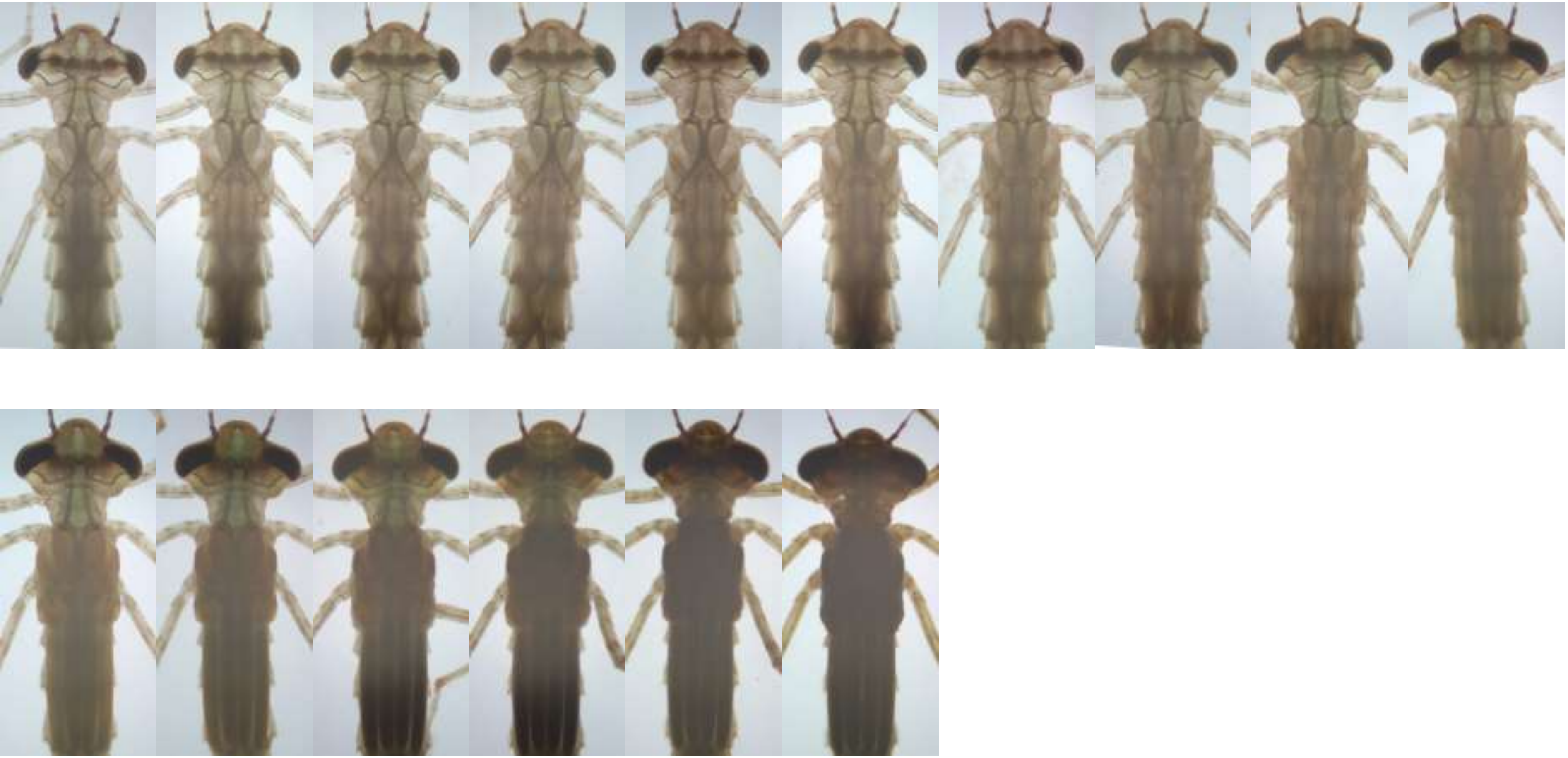

7-3 *Paracercion calamorum* (1/1)

2 mm

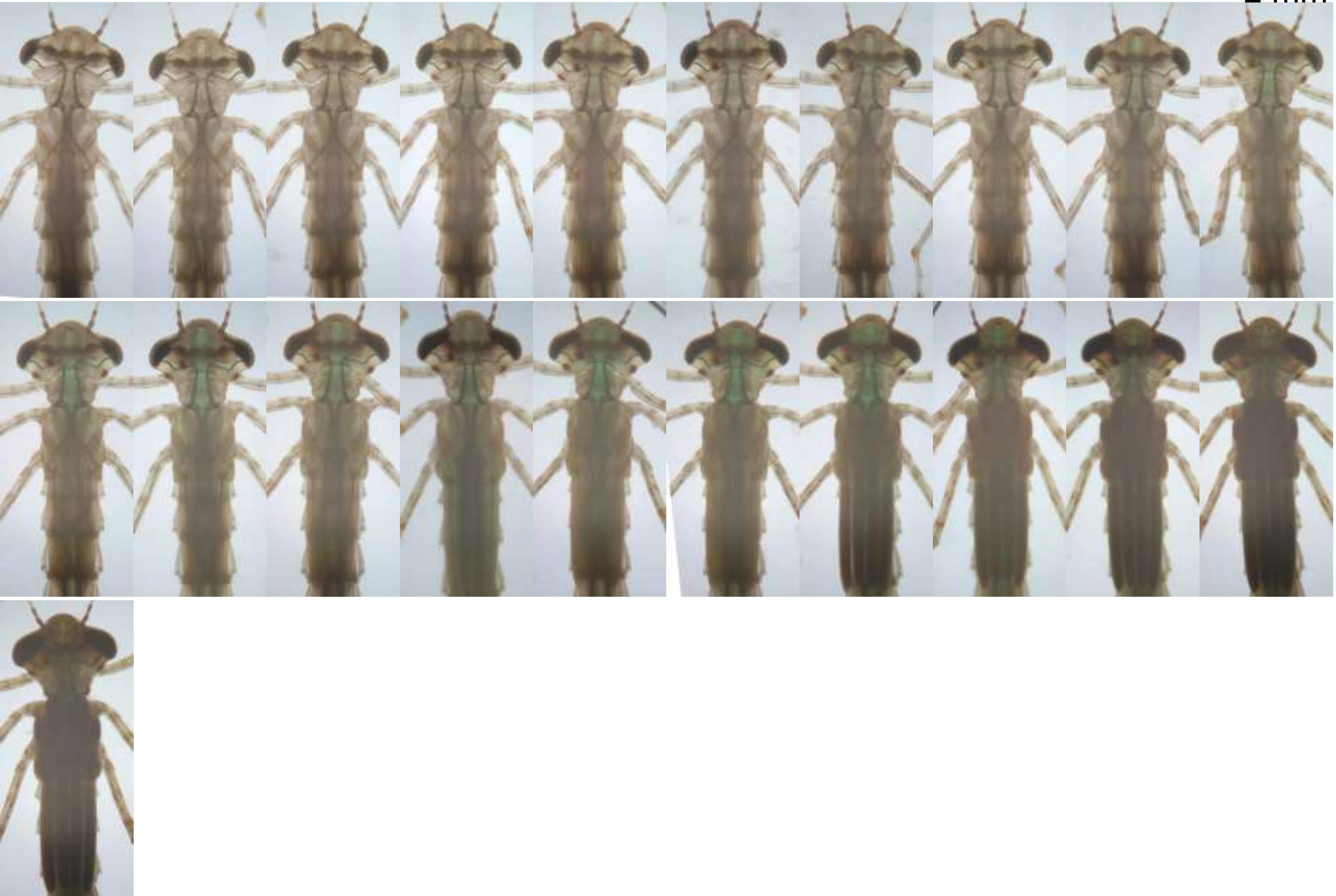

7-4 *Paracercion calamorum* (1/1)

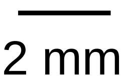

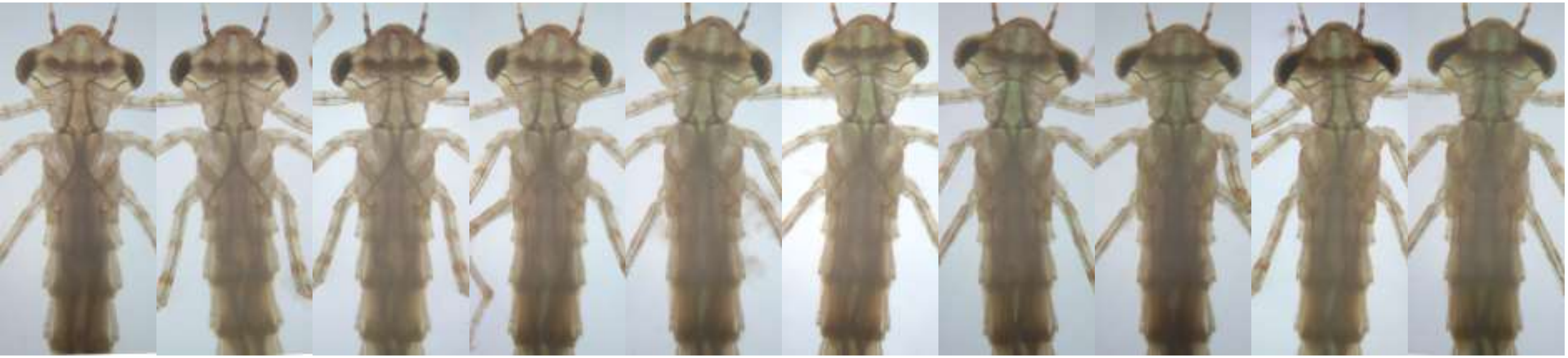

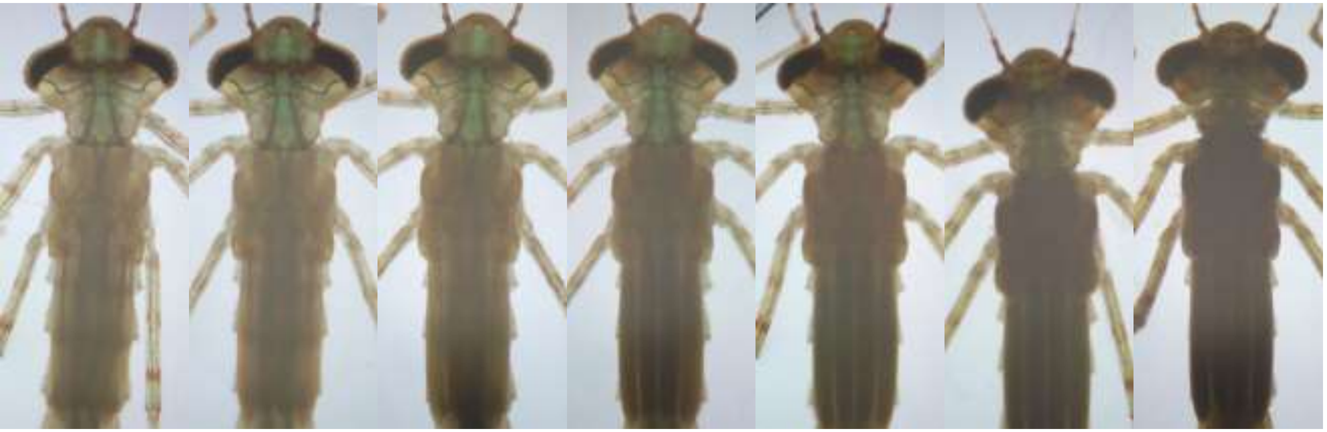

7-5 *Paracercion calamorum* (1/1)

2 mm

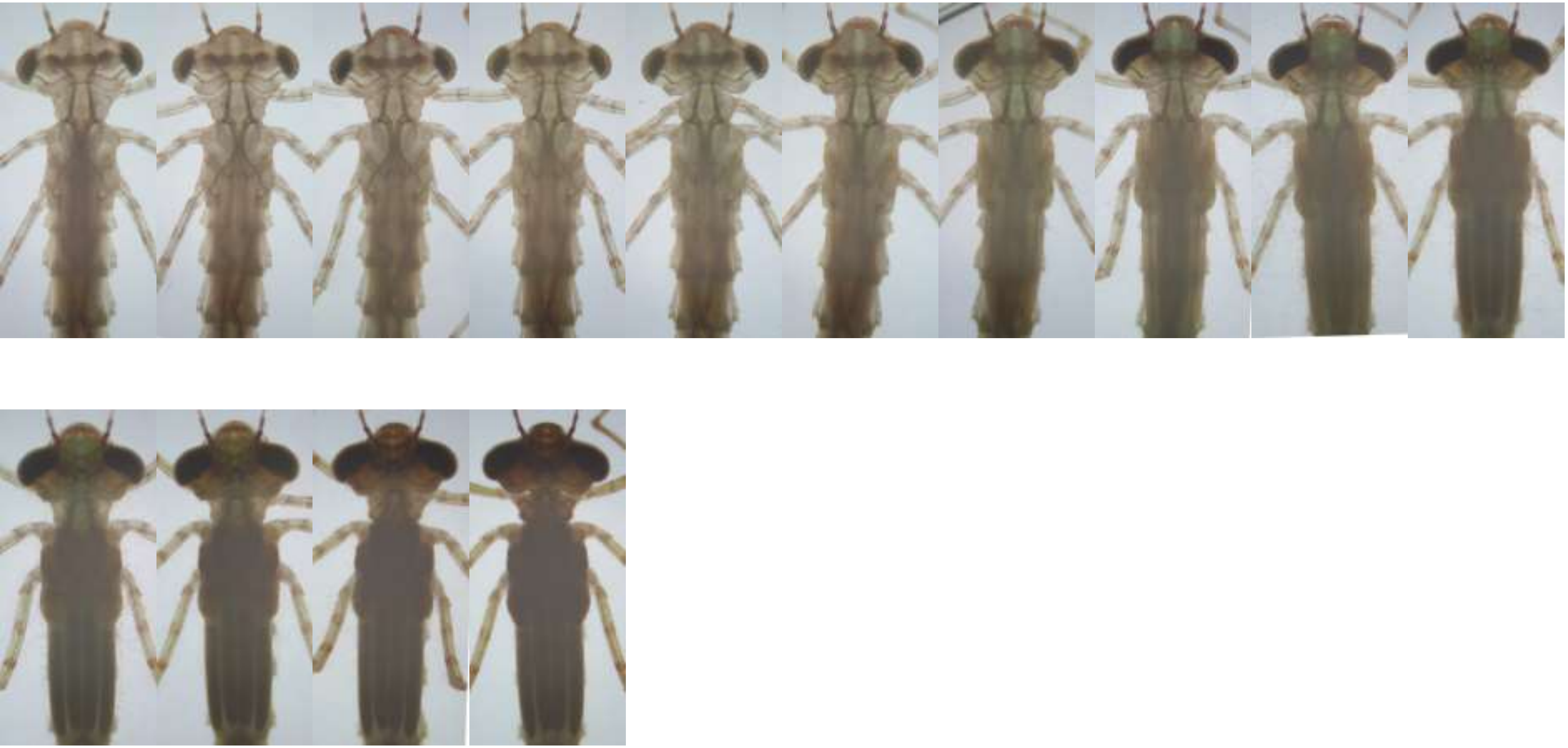

7-6 *Paracercion calamorum* (1/1)

2 mm

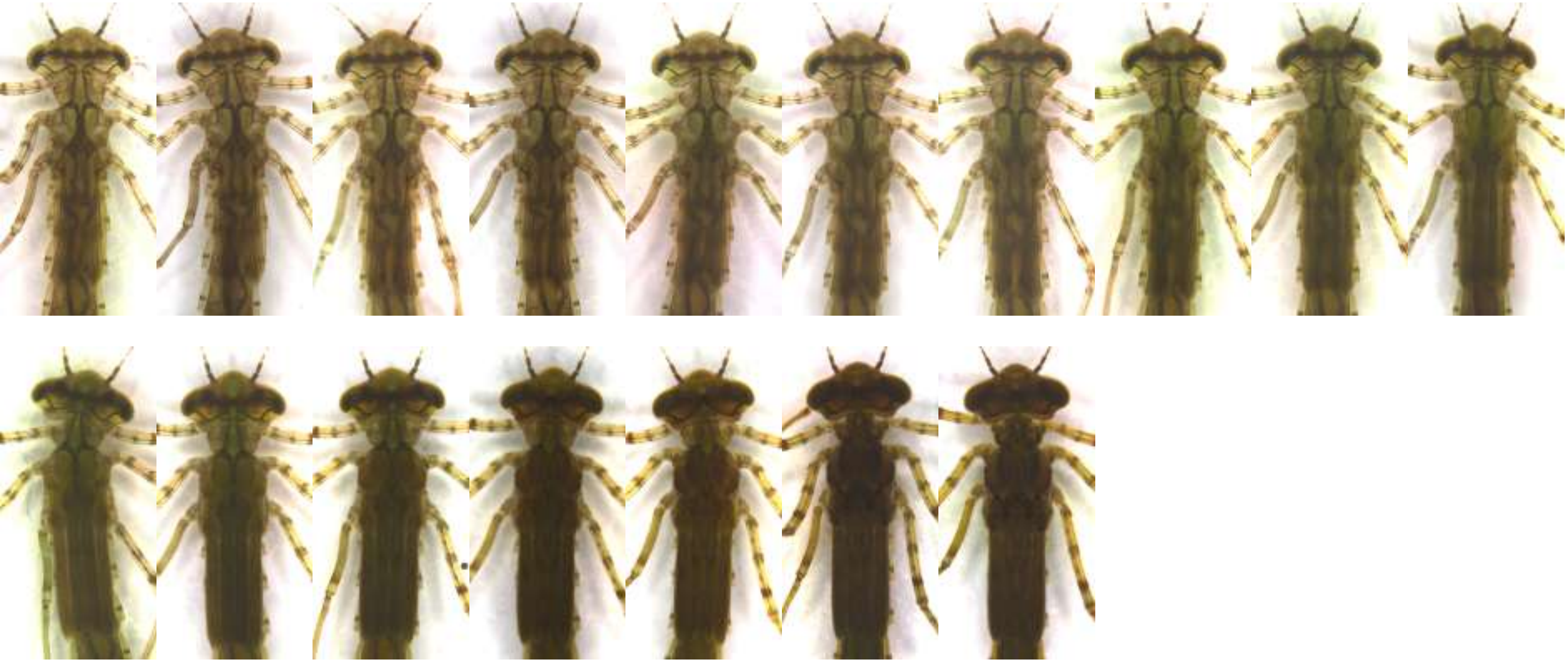

7-7 *Paracercion calamorum* (1/1)

2 mm

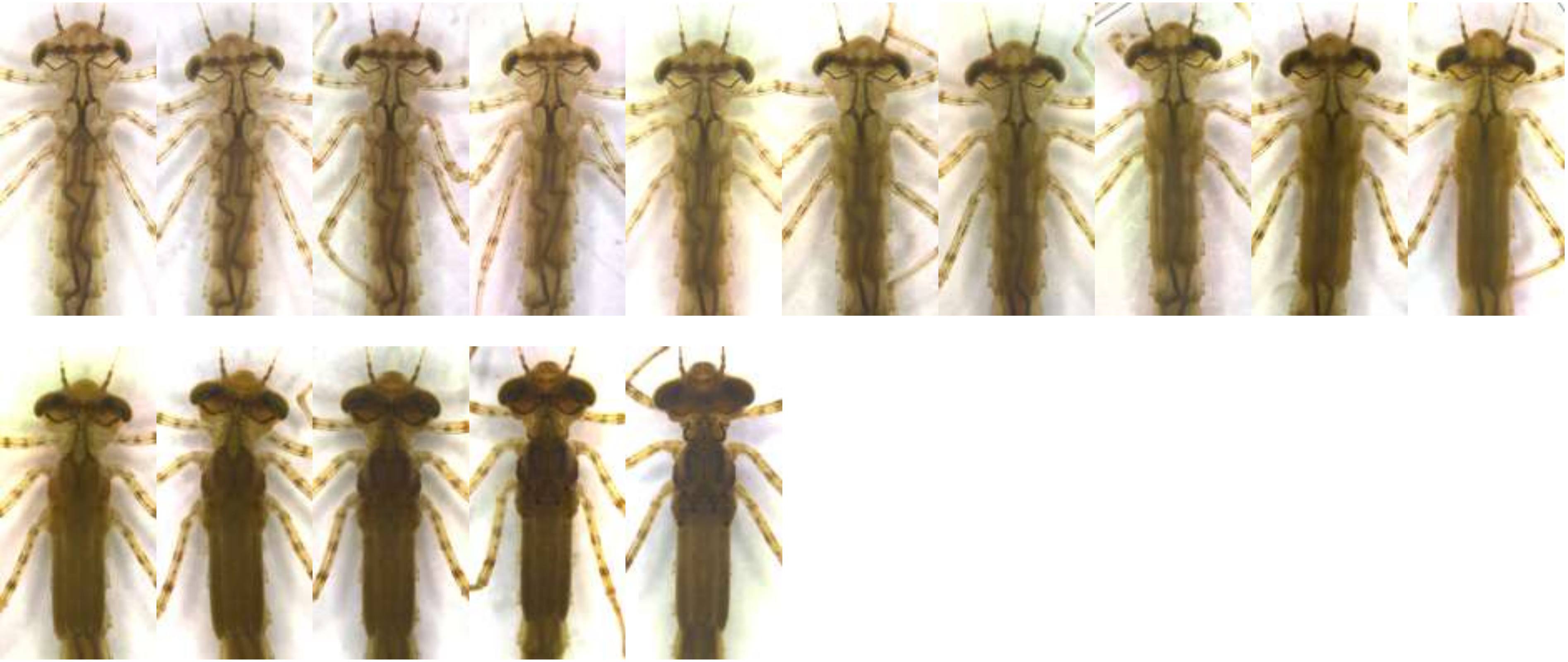

8-1 *Paracercion hieroglyphicum* (1/1)

2 mm

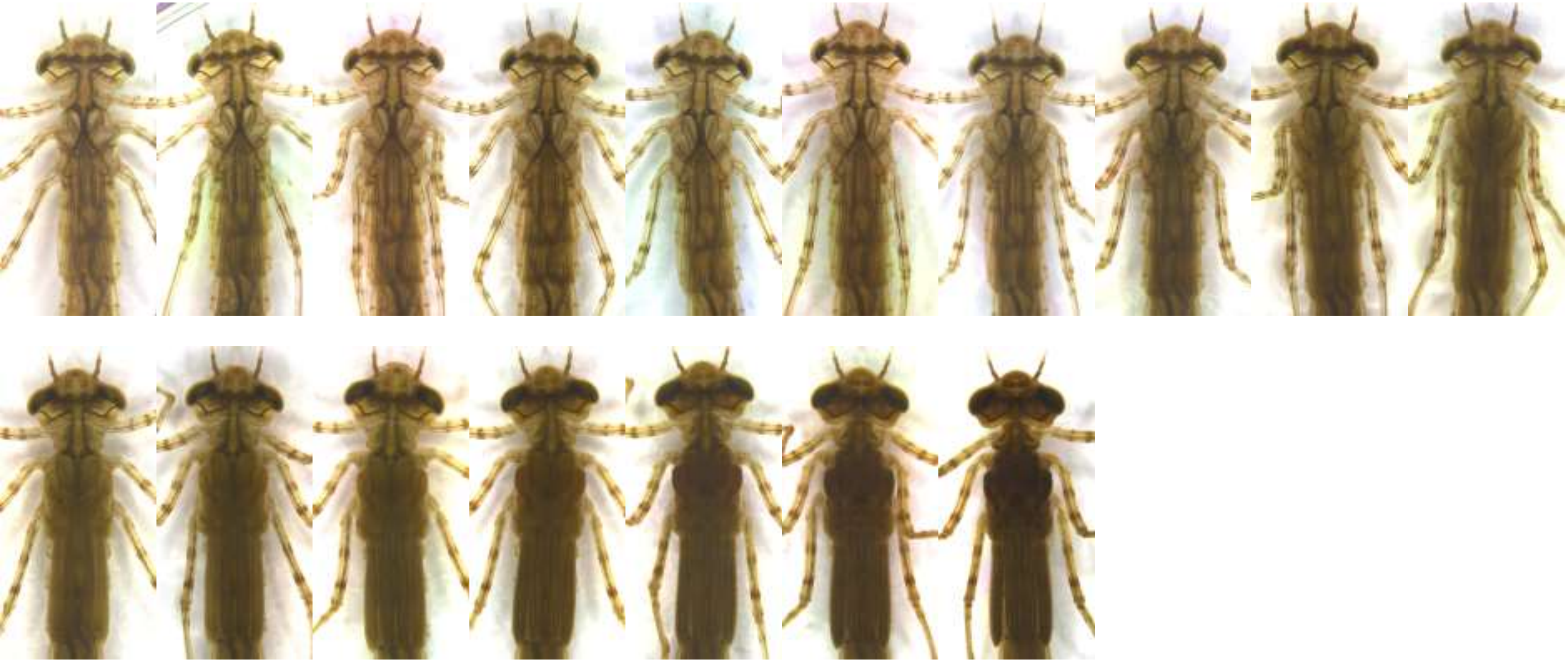

8-2 *Paracercion hieroglyphicum* (1/1)

2 mm

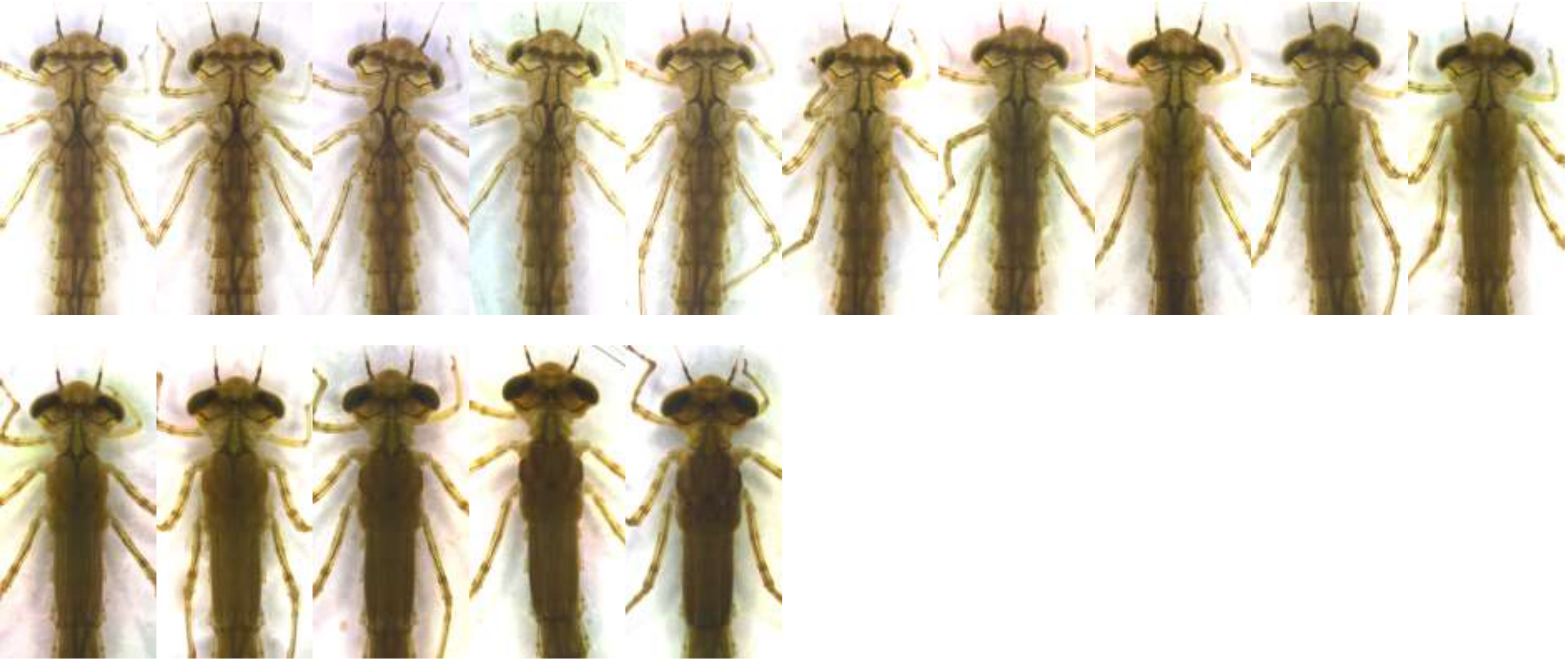

8-3 *Paracercion hieroglyphicum* (1/1)

2 mm

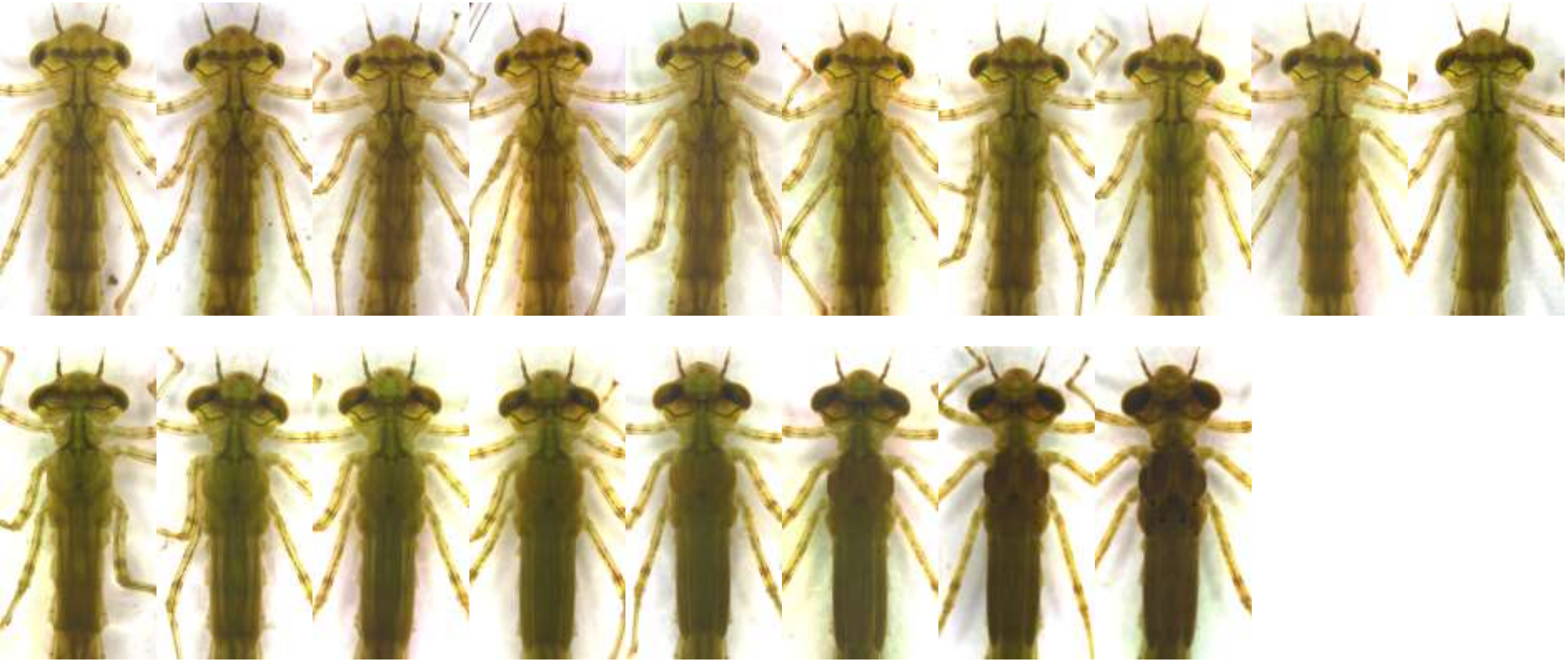

9-1 *Paracercion sieboldii* (1/1)

2 mm

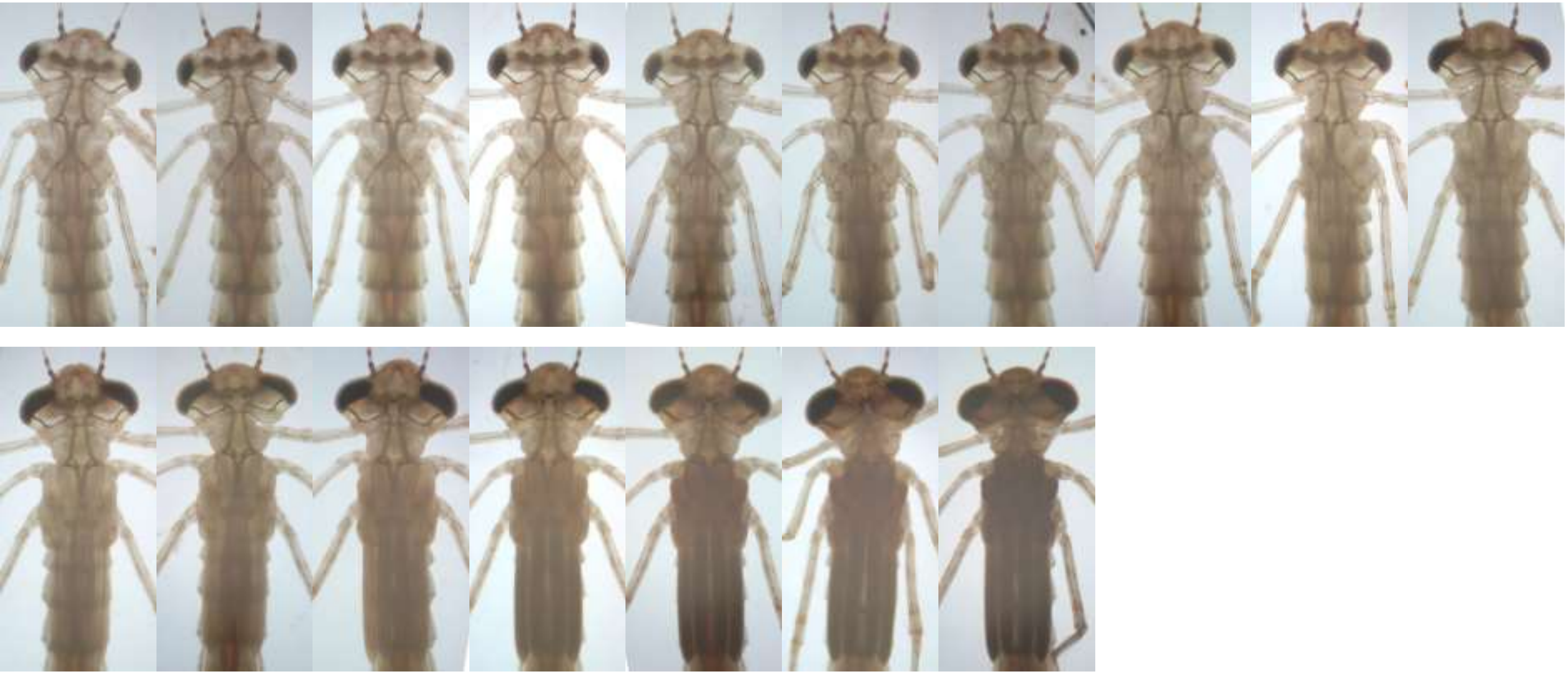

9-2 *Paracercion sieboldii* (1/1)

2 mm

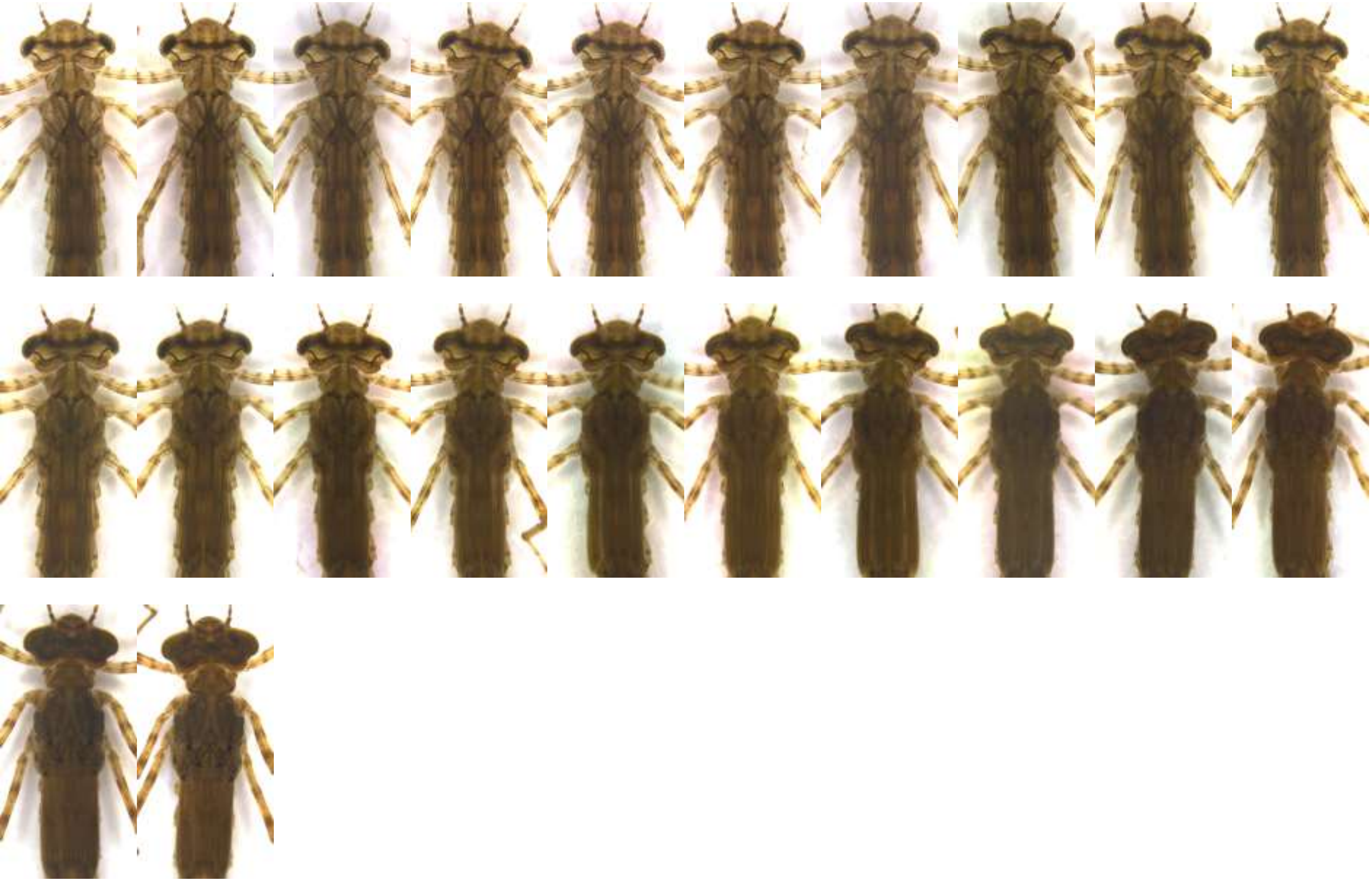

9-3 *Paracercion sieboldii* (1/1)

2 mm

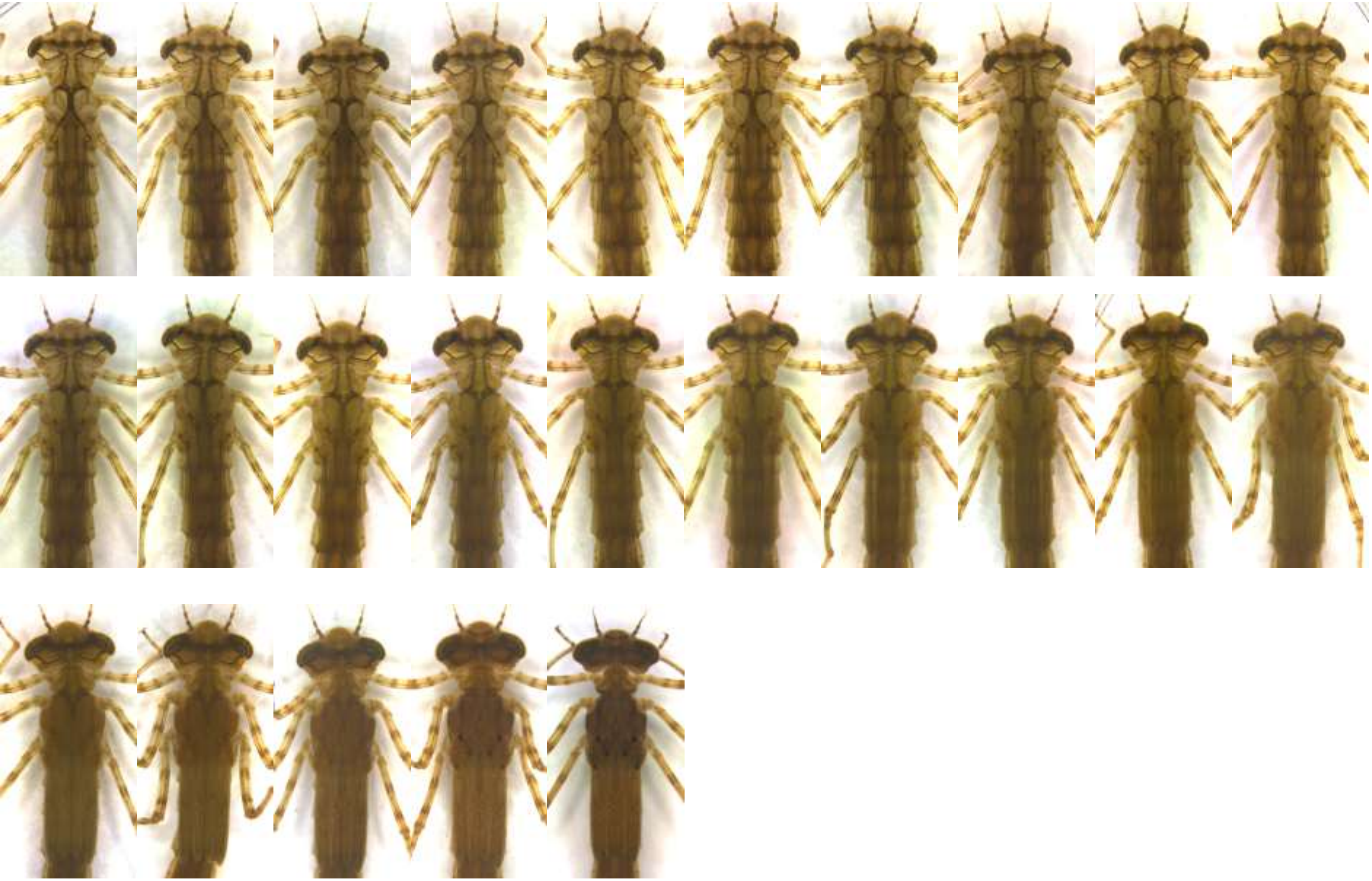

10-1 *Paracercion melanotum* (1/1)

2 mm

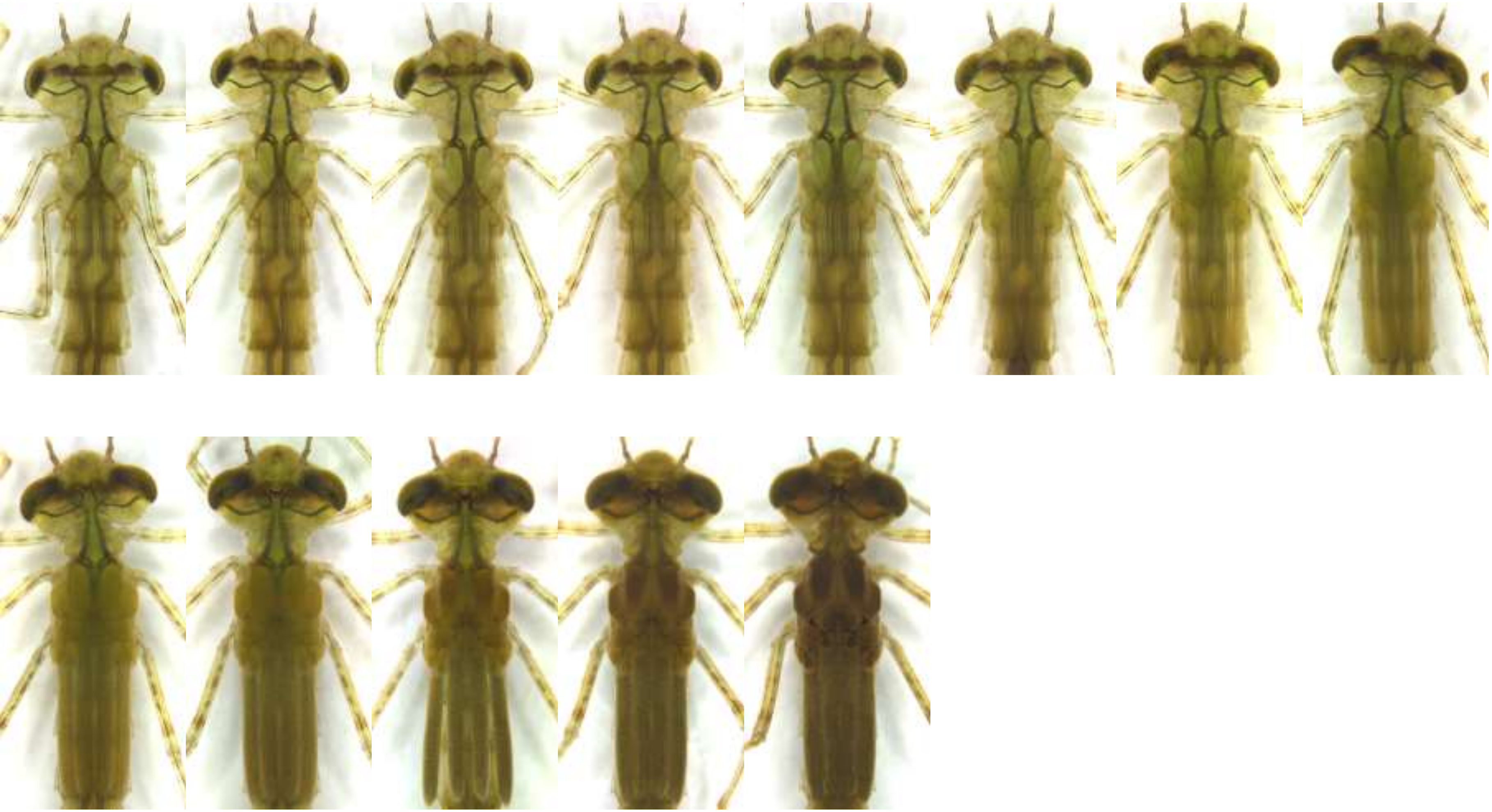

10-2 *Paracercion melanotum* (1/1)

2 mm

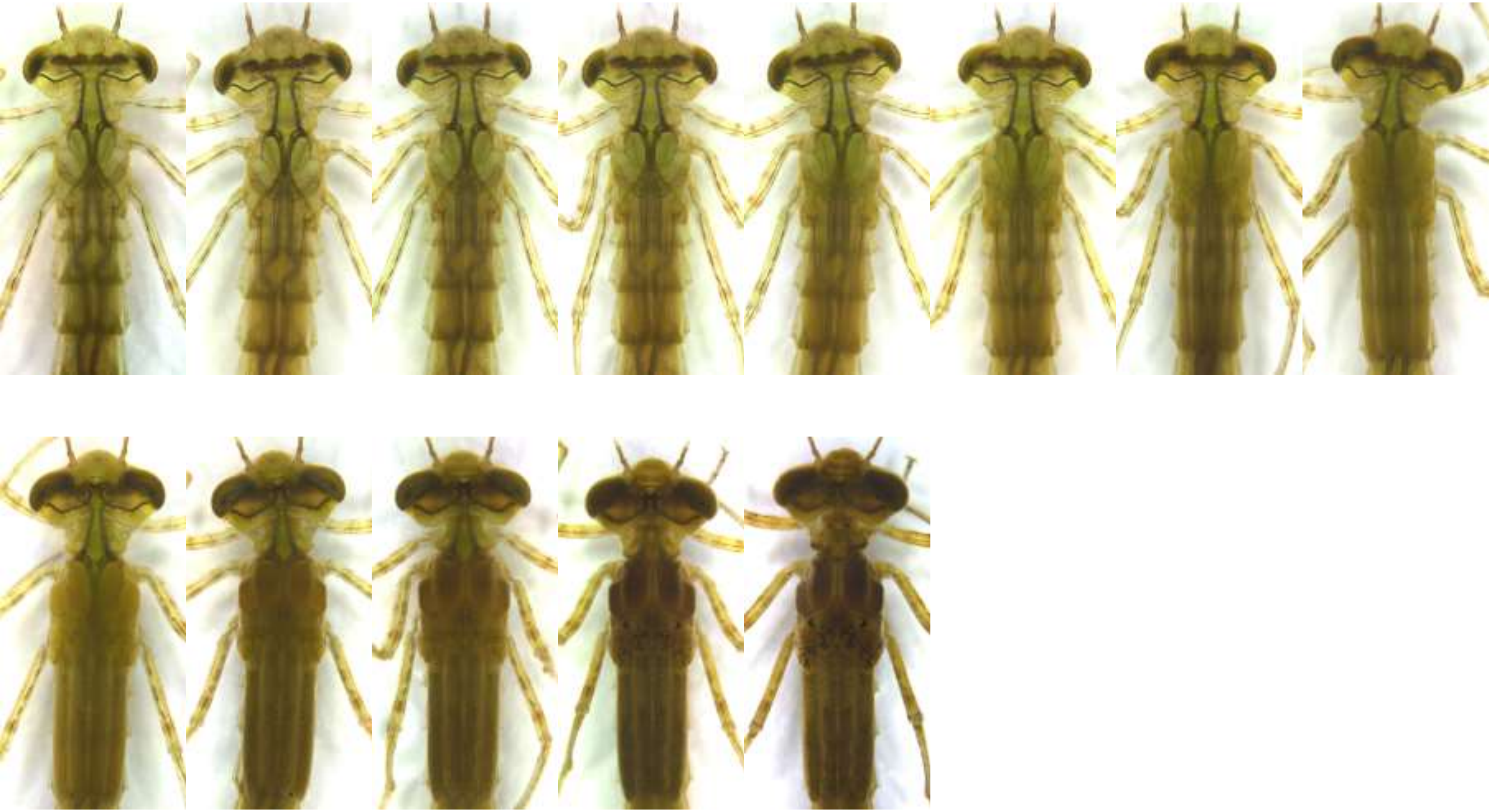

11-1 *Agriocnemis pygmaea* (1/1)

2 mm

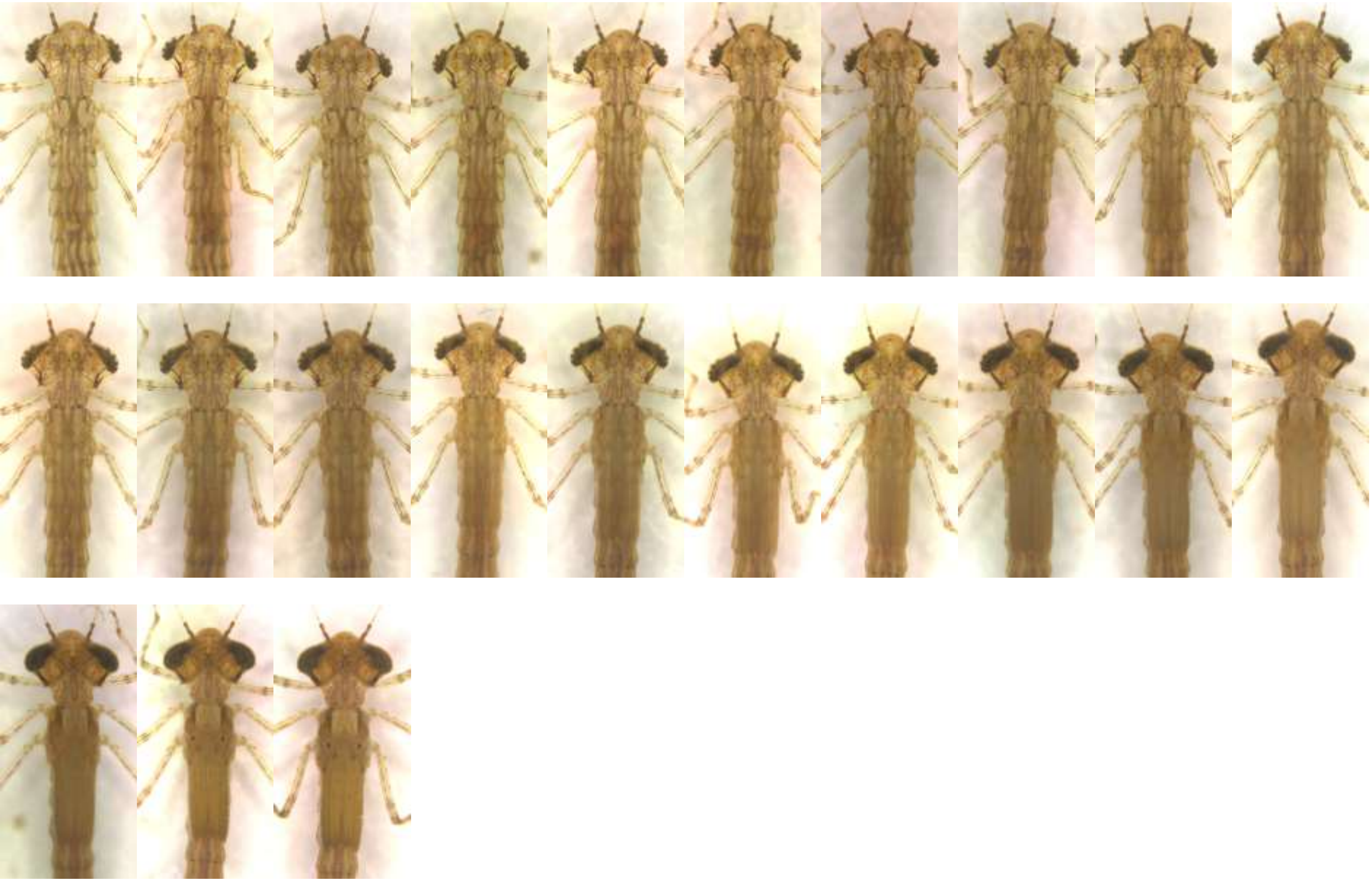

12-1 *Mortonagrion selenion* (1/1)

2 mm

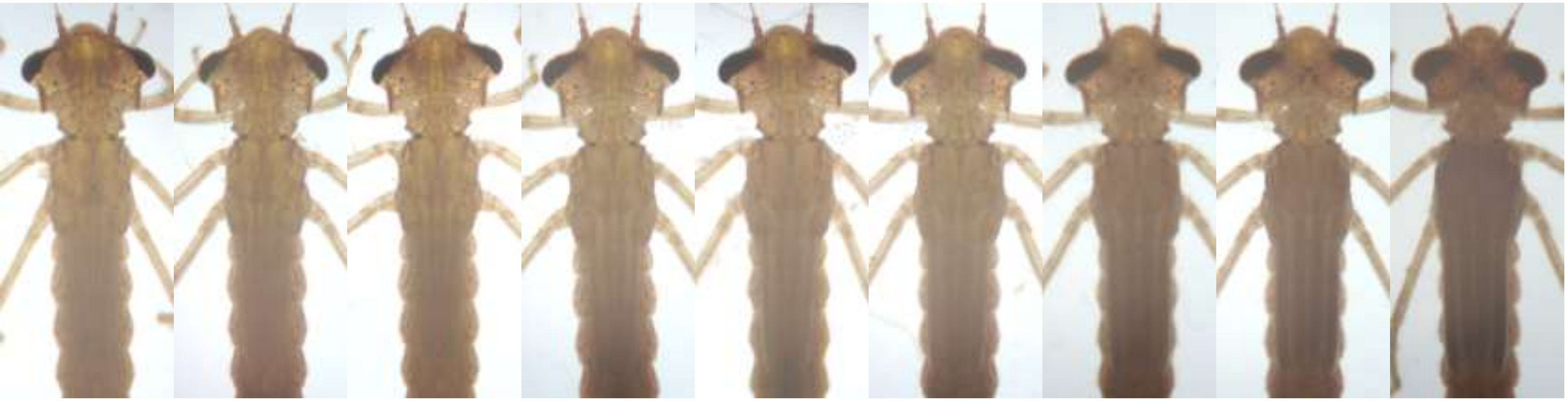

13-1 *Enallagma circulatum* (1/1)

2 mm

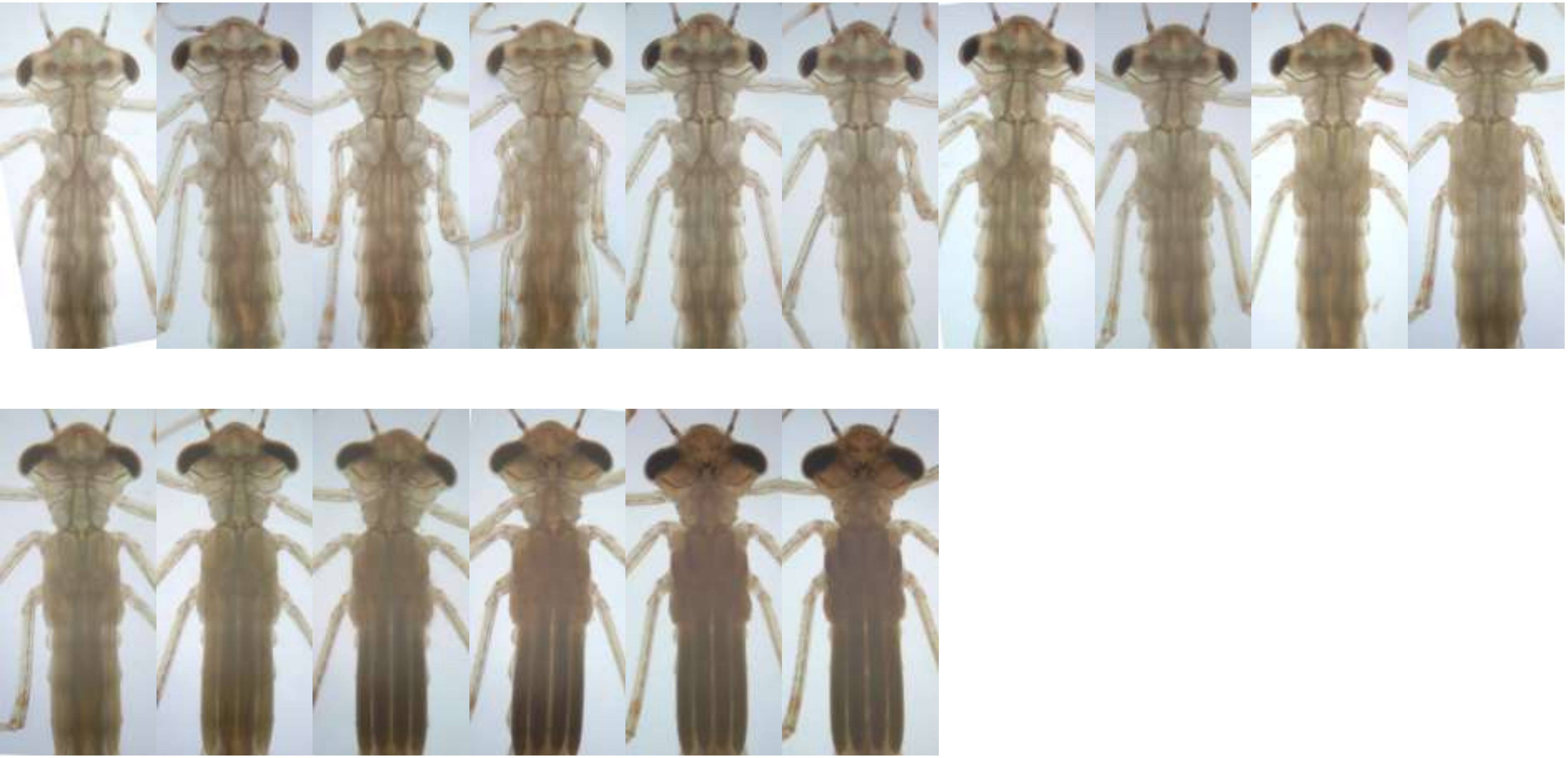

13-2 *Enallagma circulatum* (1/1)

2 mm

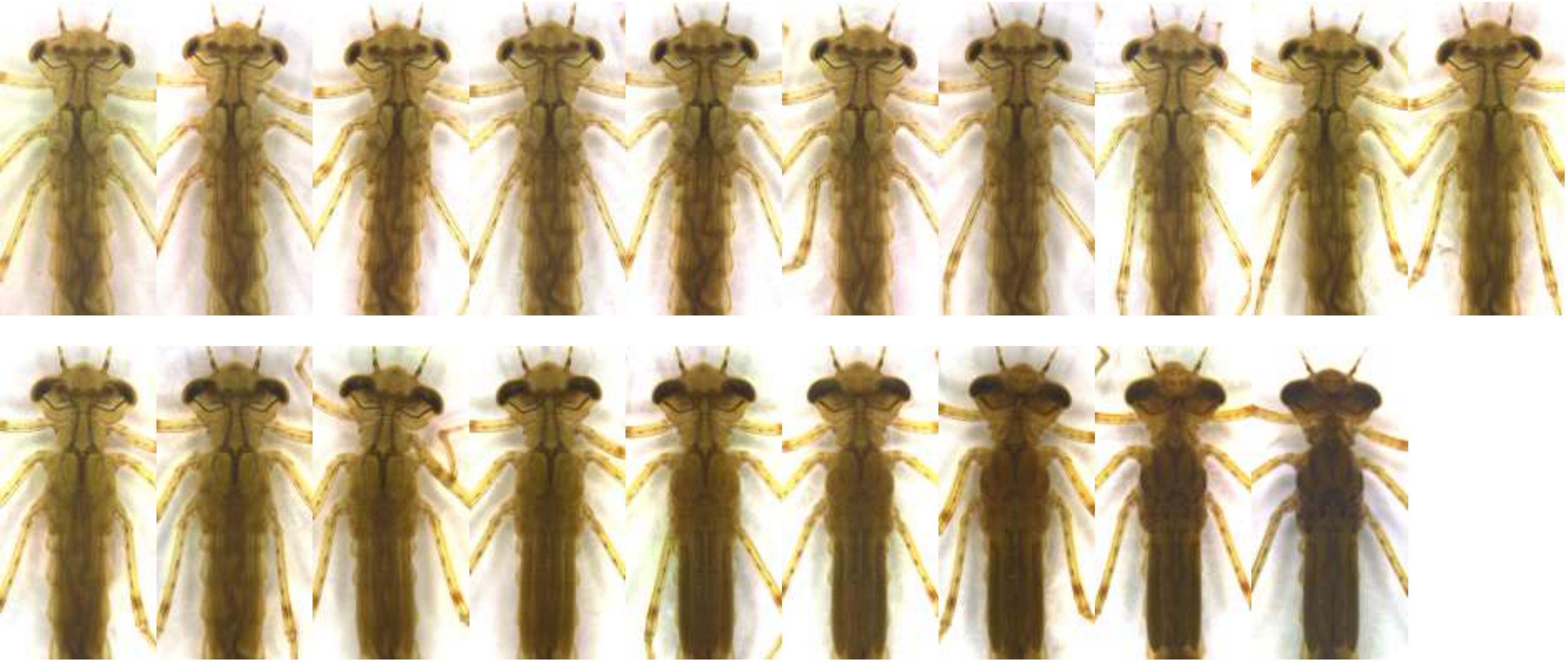

13-3 *Enallagma circulatum* (1/1)

2 mm

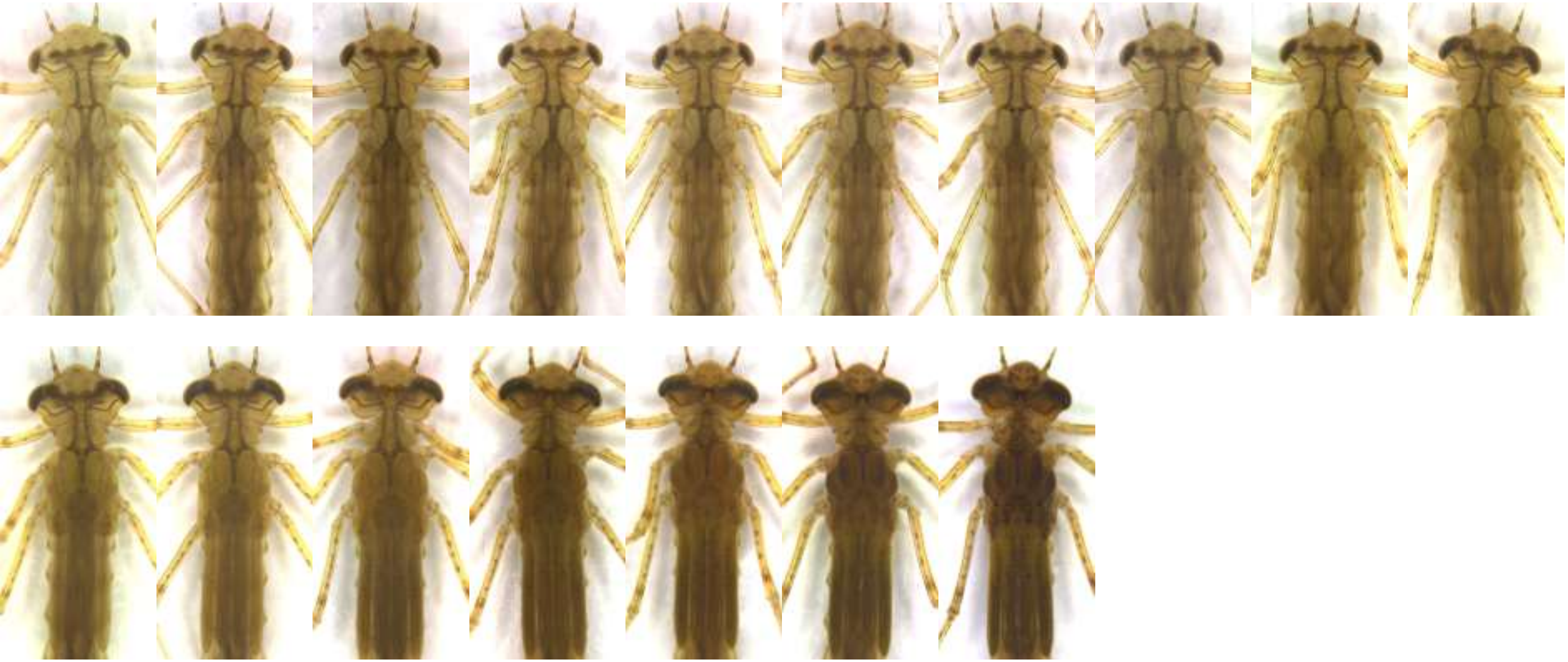

13-4 *Enallagma circulatum* (1/1)

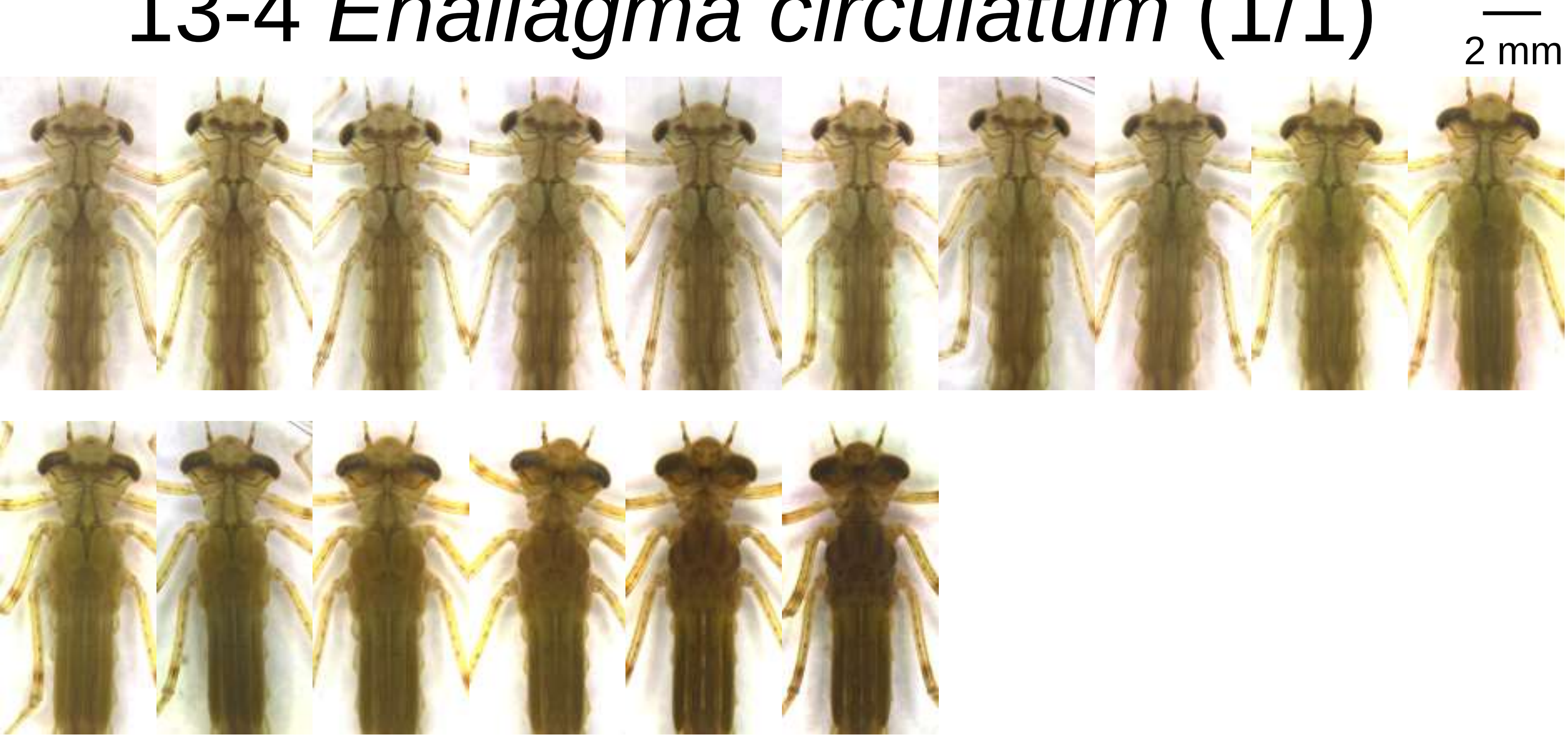

13-5 *Enallagma circulatum* (1/1)

2 mm

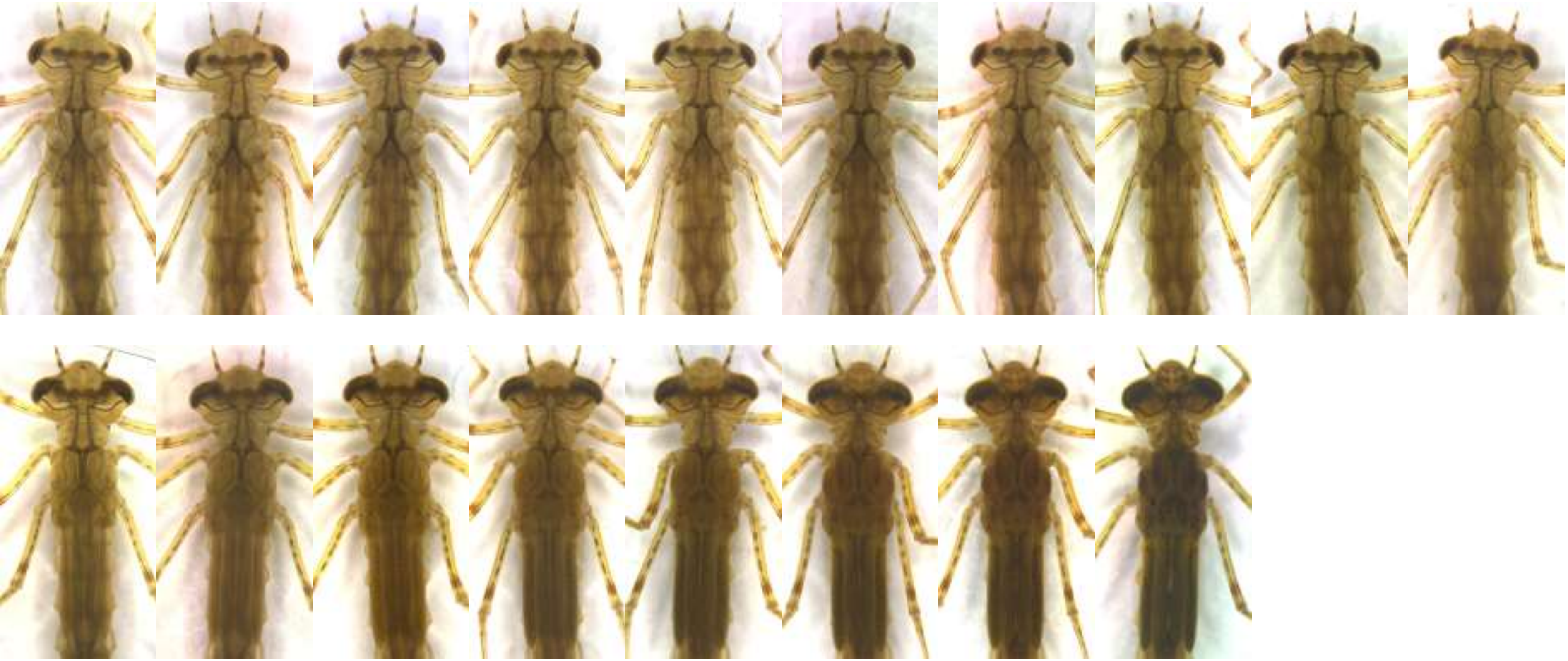

14-1 *Ischnura senegalensis* (1/1)

2 mm

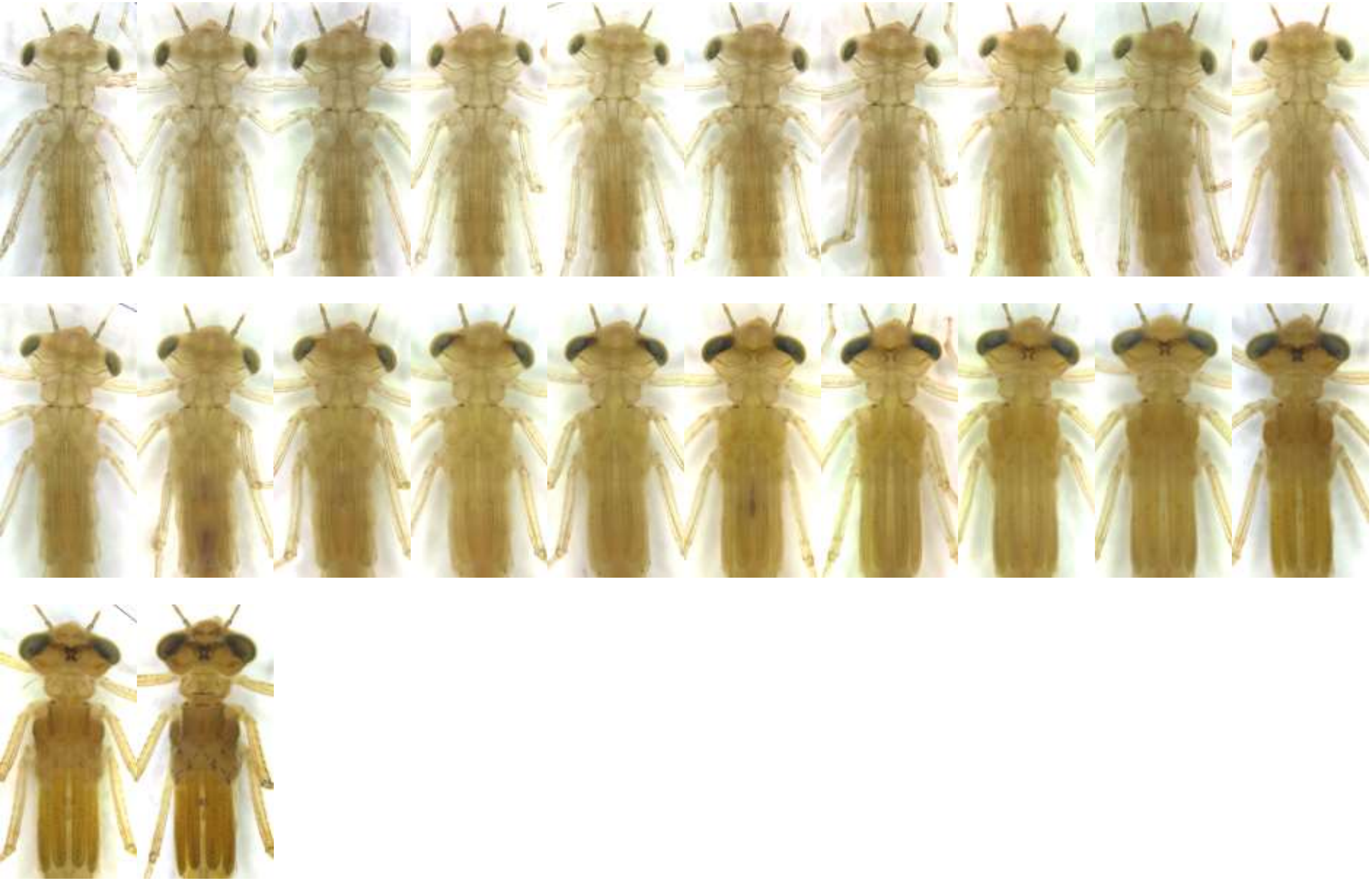

14-2 *Ischnura senegalensis* (1/1)

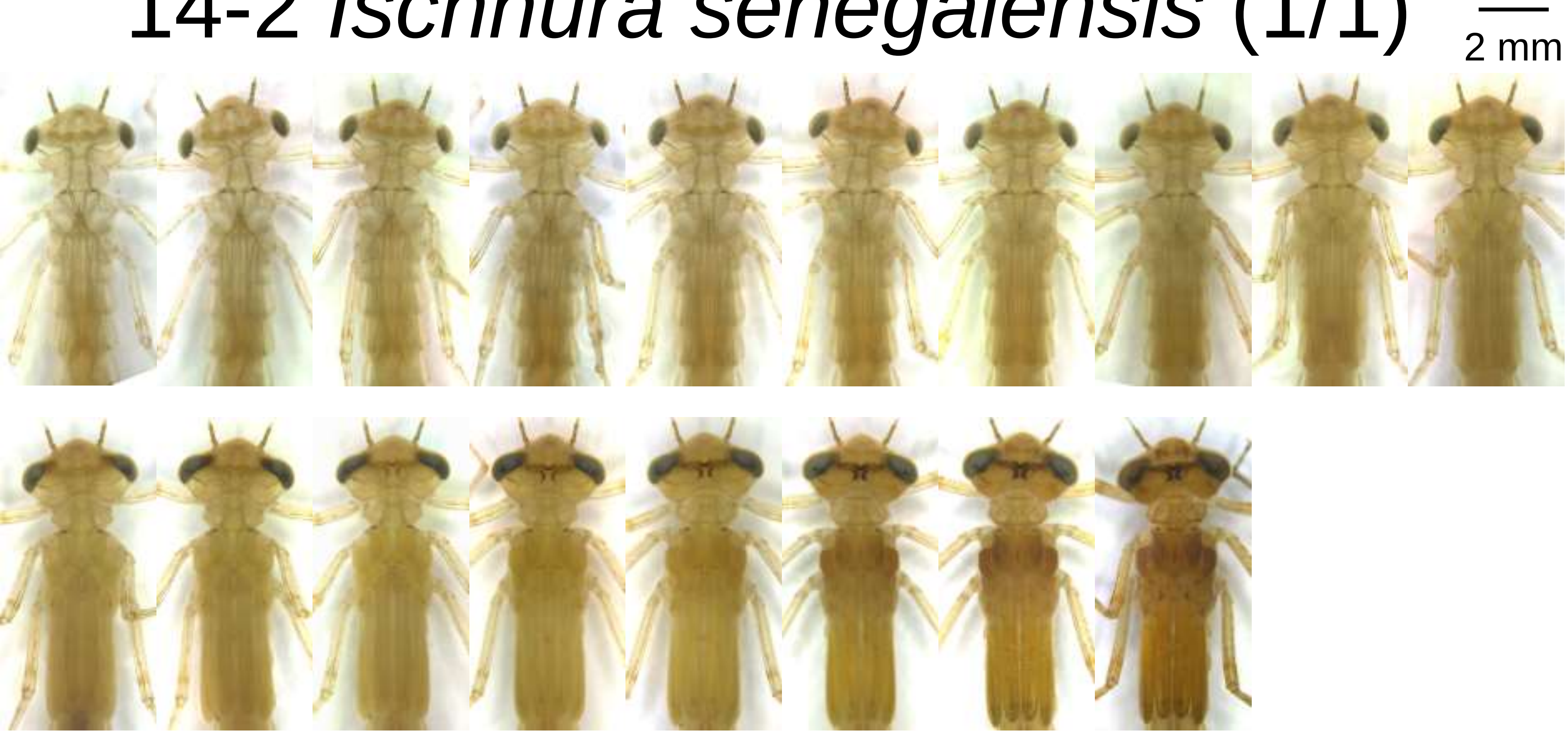

15-1 *Ischnura asiatica* (1/1)

2 mm

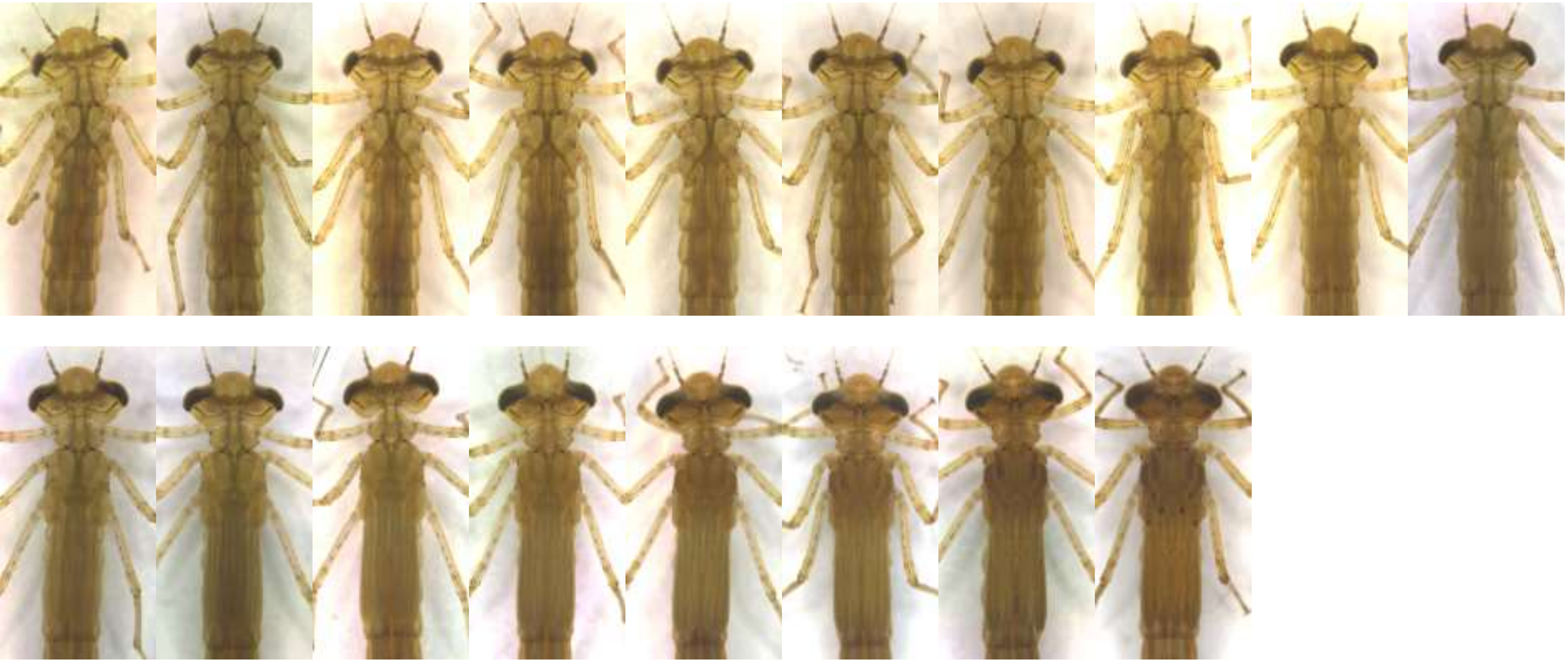

15-2 *Ischnura asiatica* (1/1)

2 mm

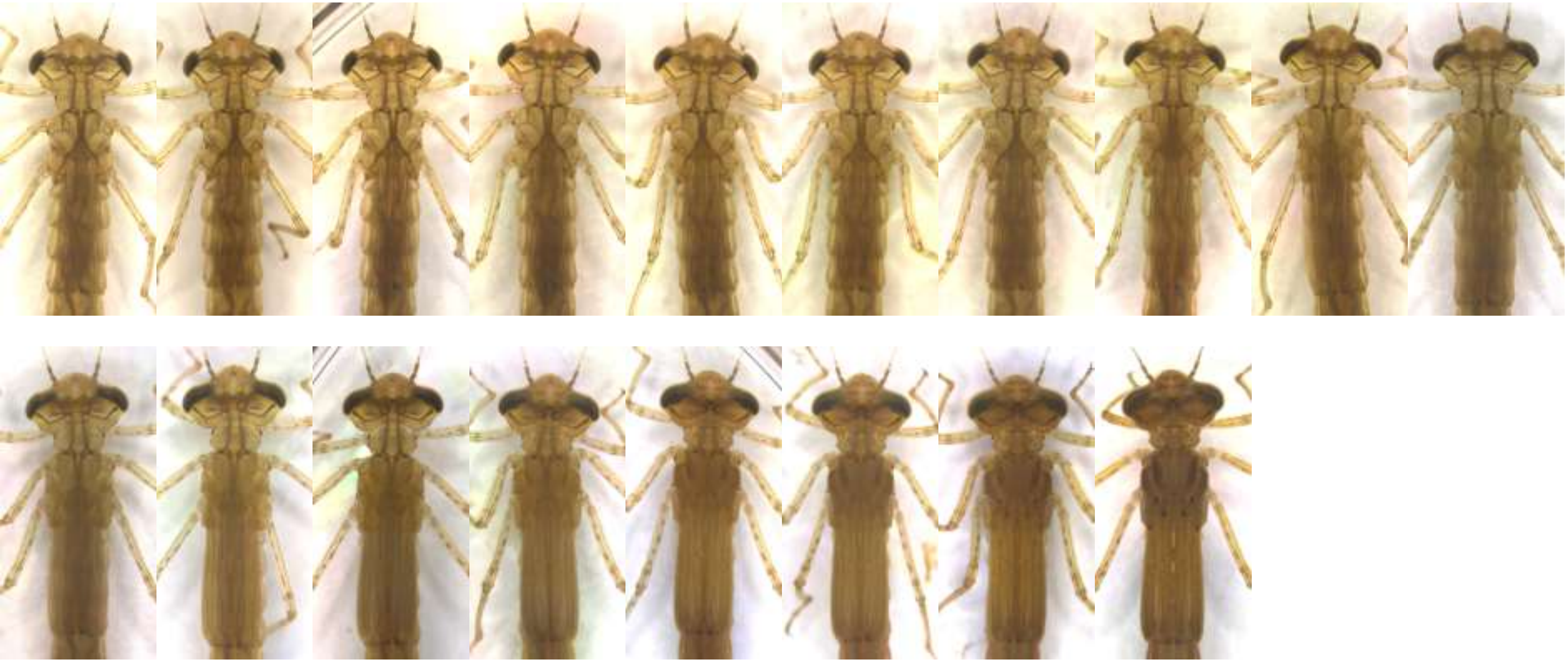

15-3 *Ischnura asiatica* (1/1)

2 mm

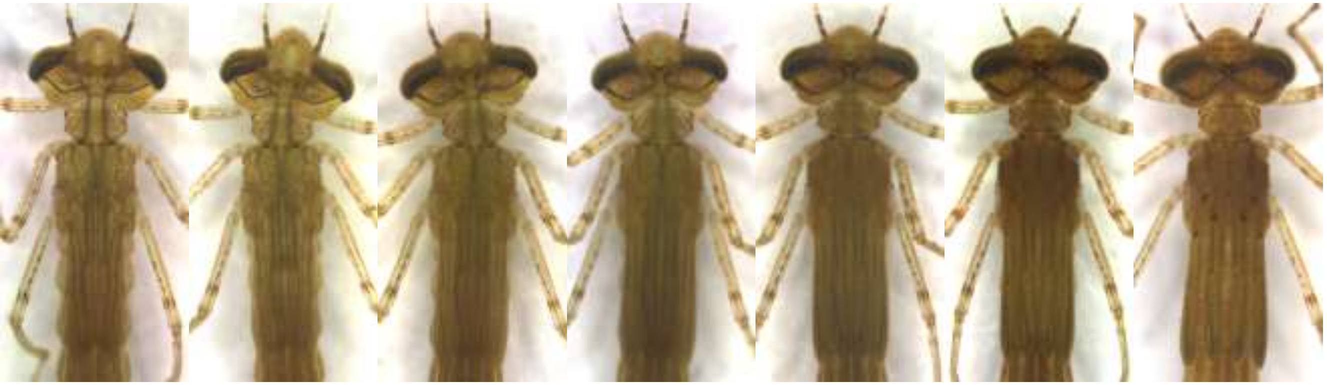

16-1 *Epiophlebia superstes* (1/1)

2 mm

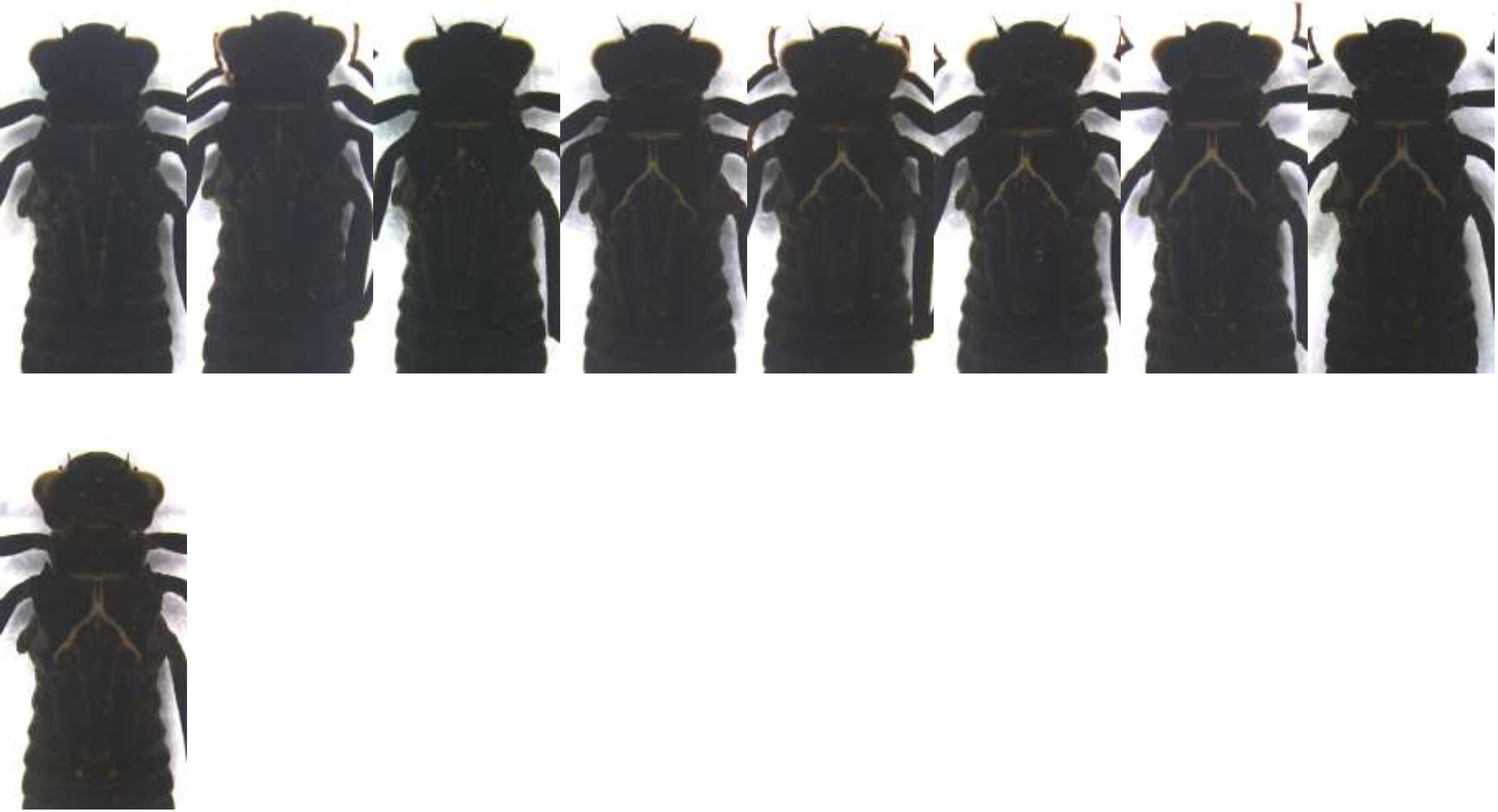

16-2 *Epiophlebia superstes* (1/1)

2 mm

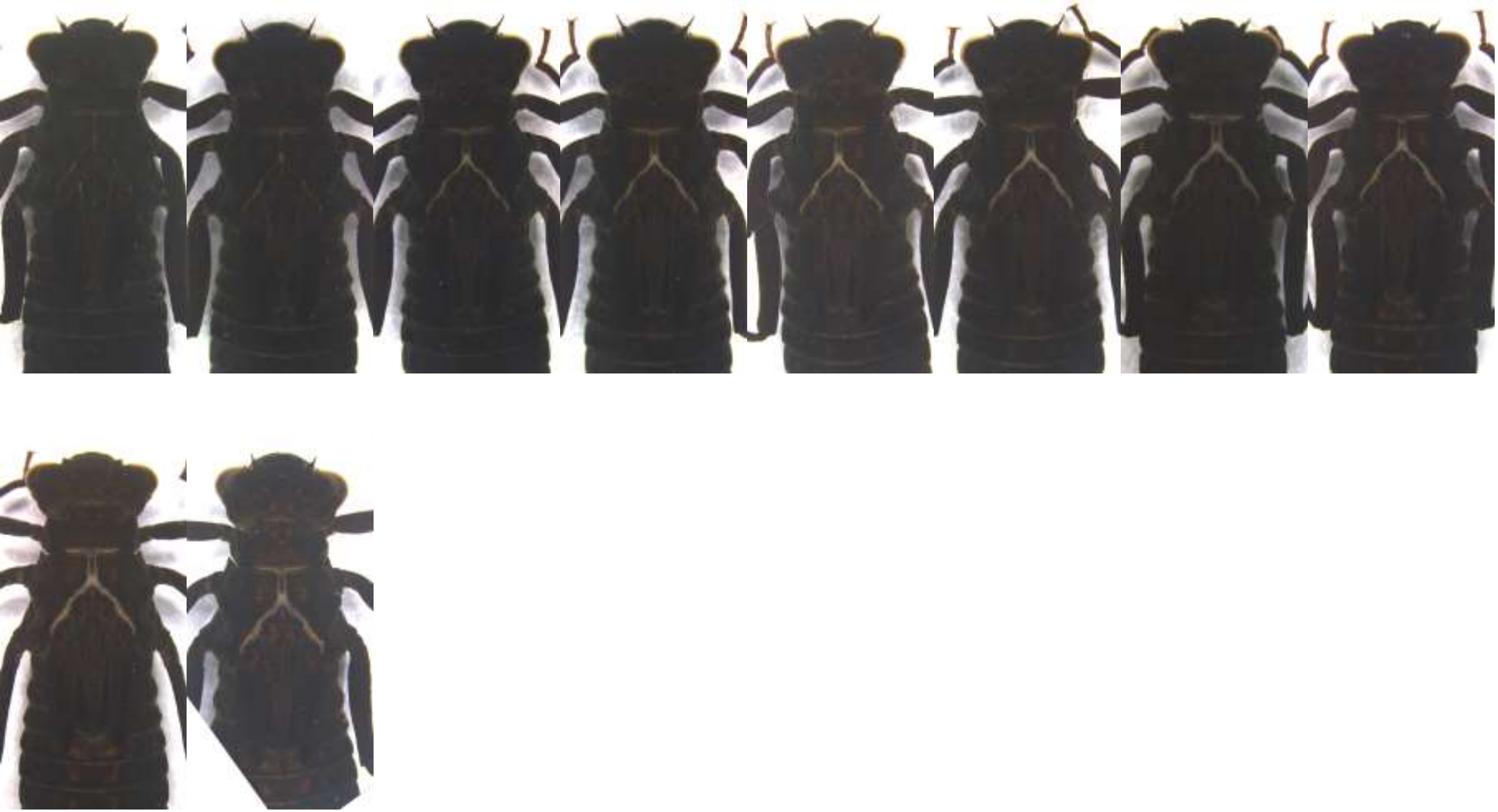

17-1 *Boyeria maclachlani* (1/2)

5 mm

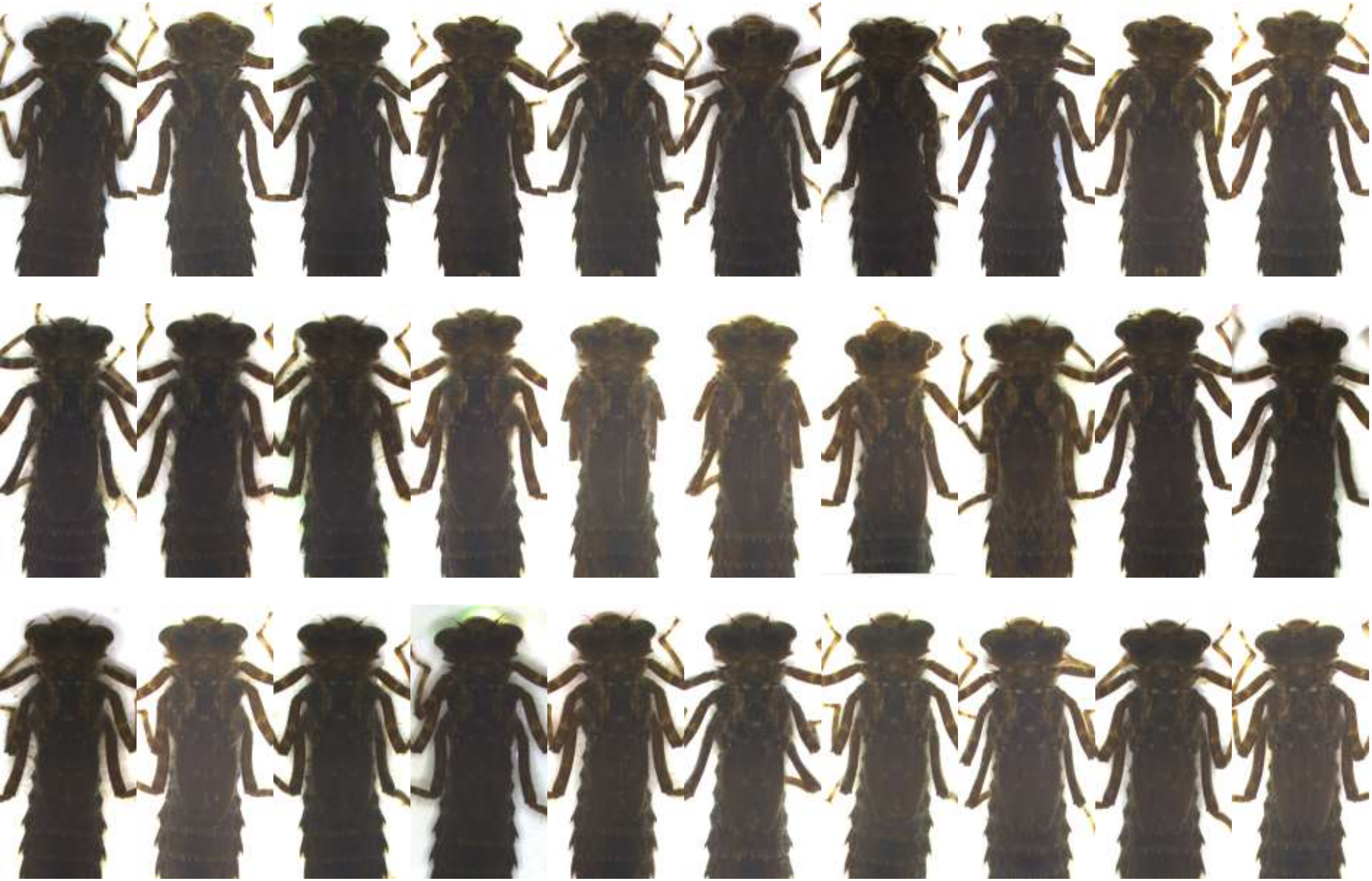

17-1 *Boyeria maclachlani* (2/2)

5 mm

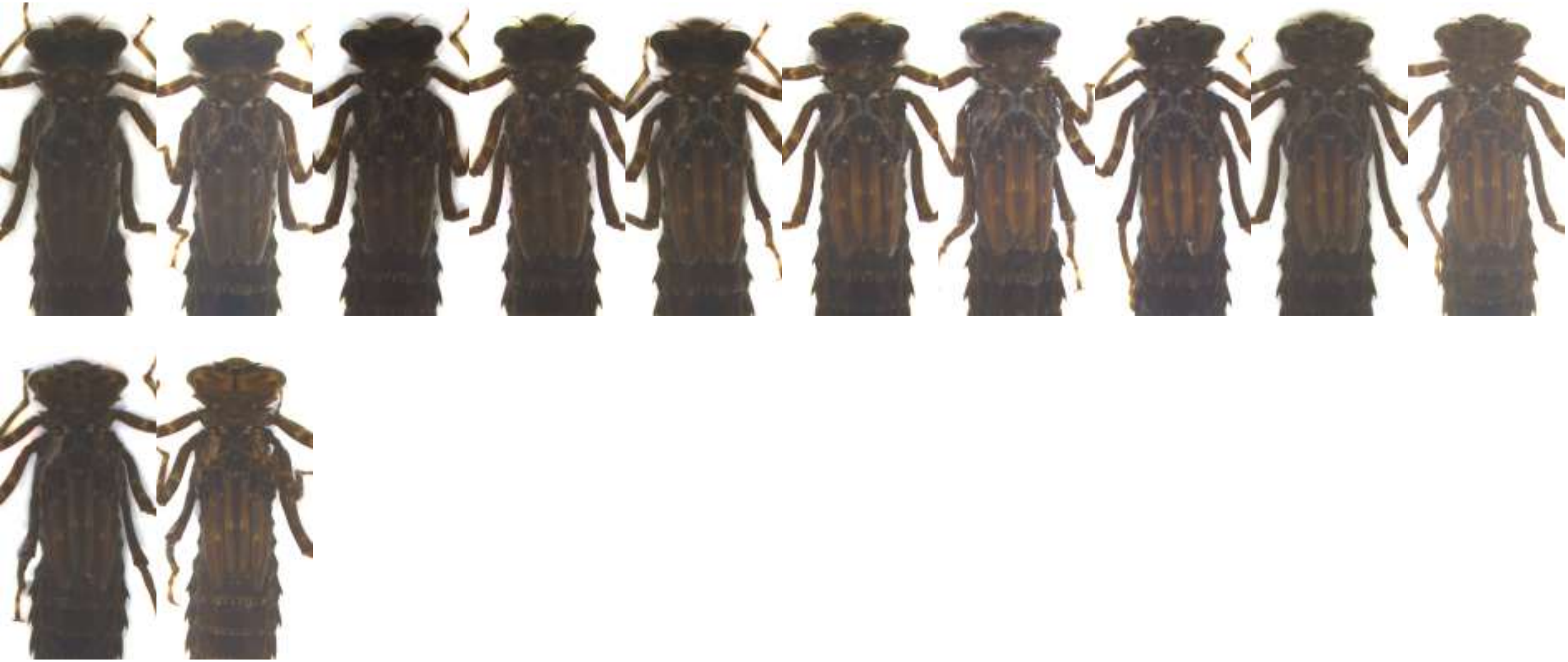

17-2 *Boyeria maclachlani* (1/2)

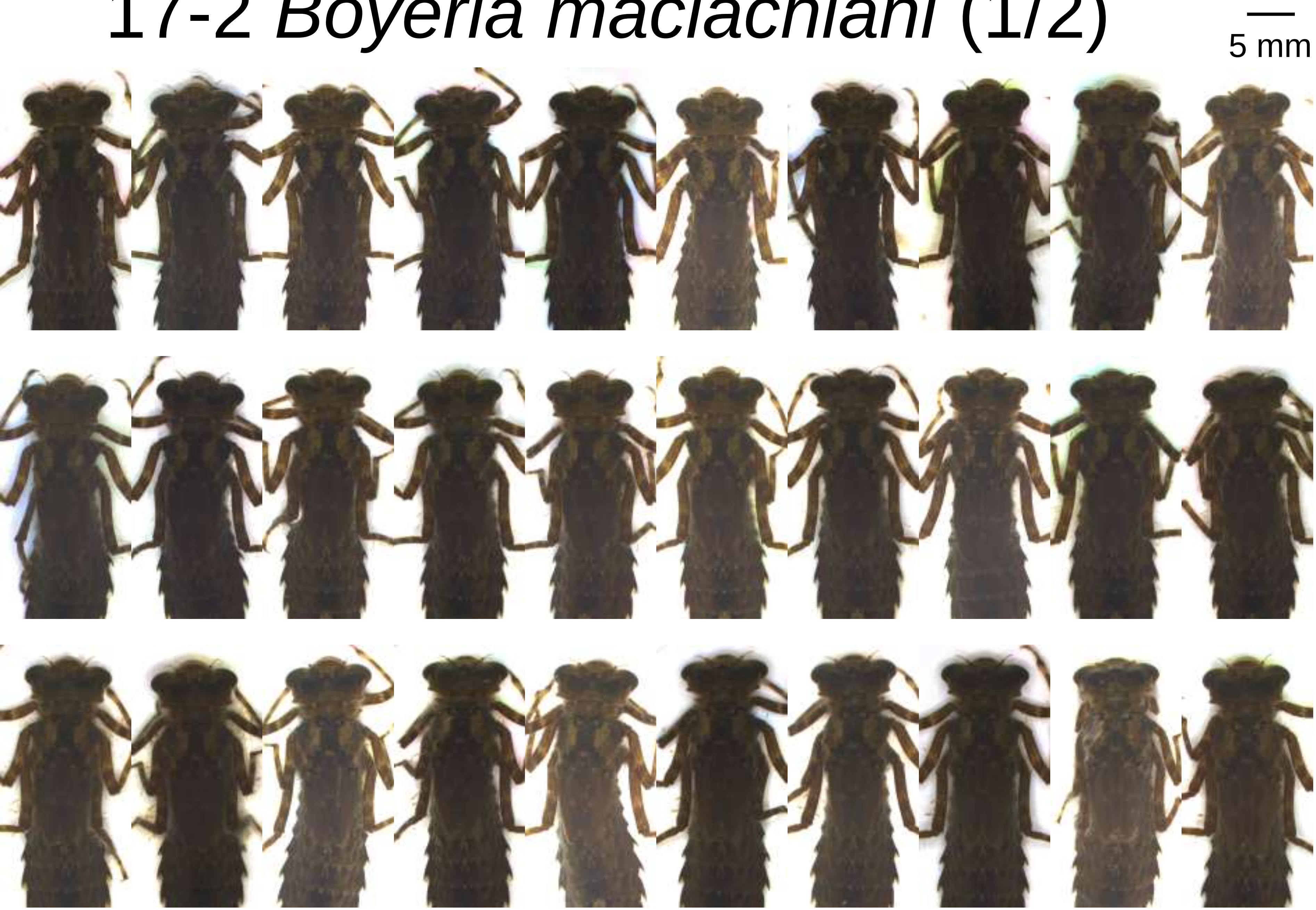

17-2 *Boyeria maclachlani* (2/2)

5 mm

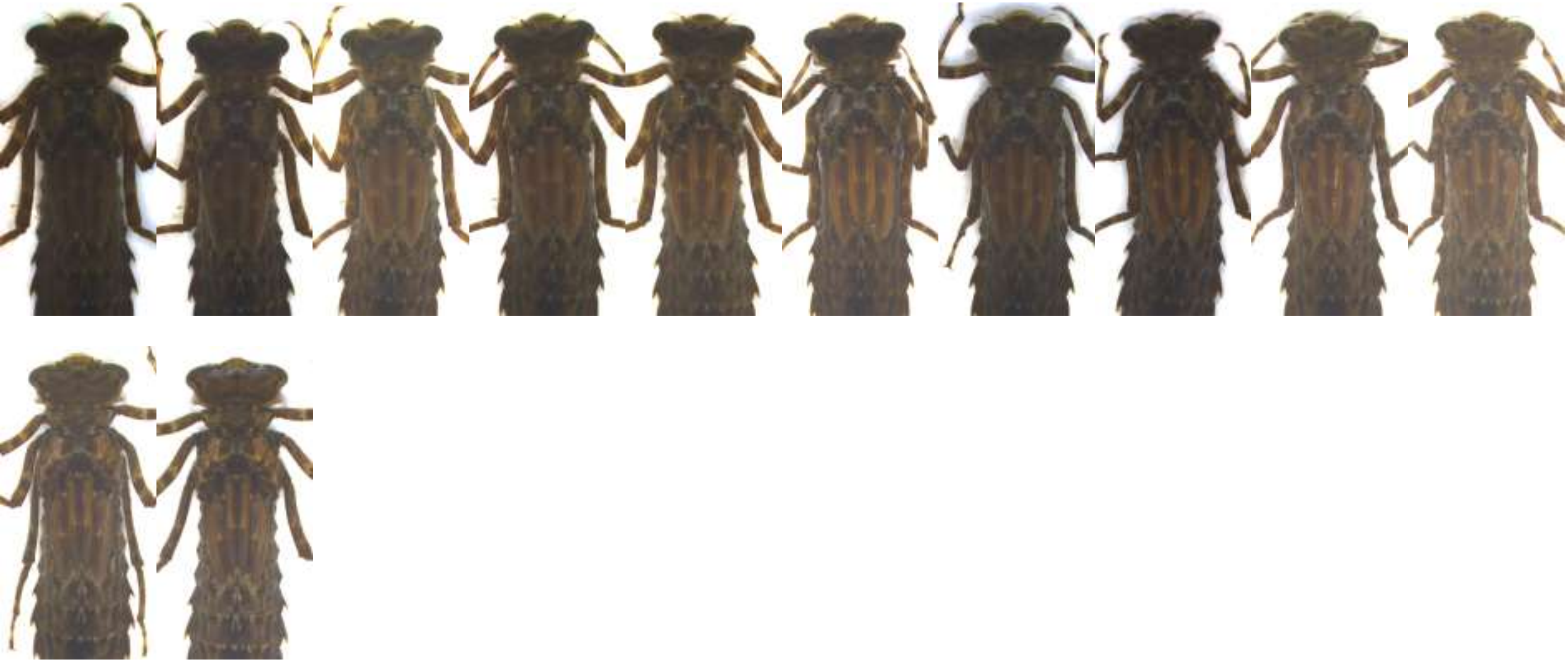

18-1 *Planaeschna milnei* (1/1)

5 mm

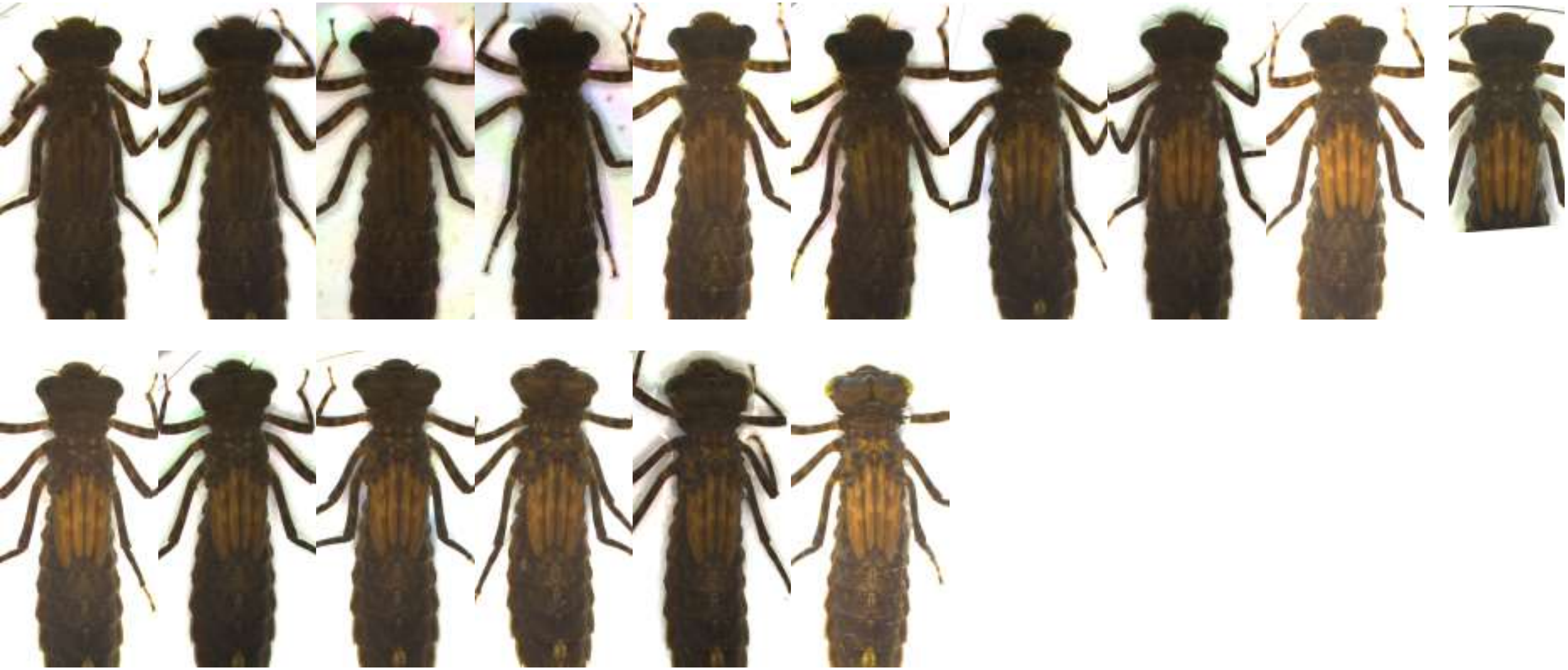

18-2 *Planaeschna milnei* (1/2)

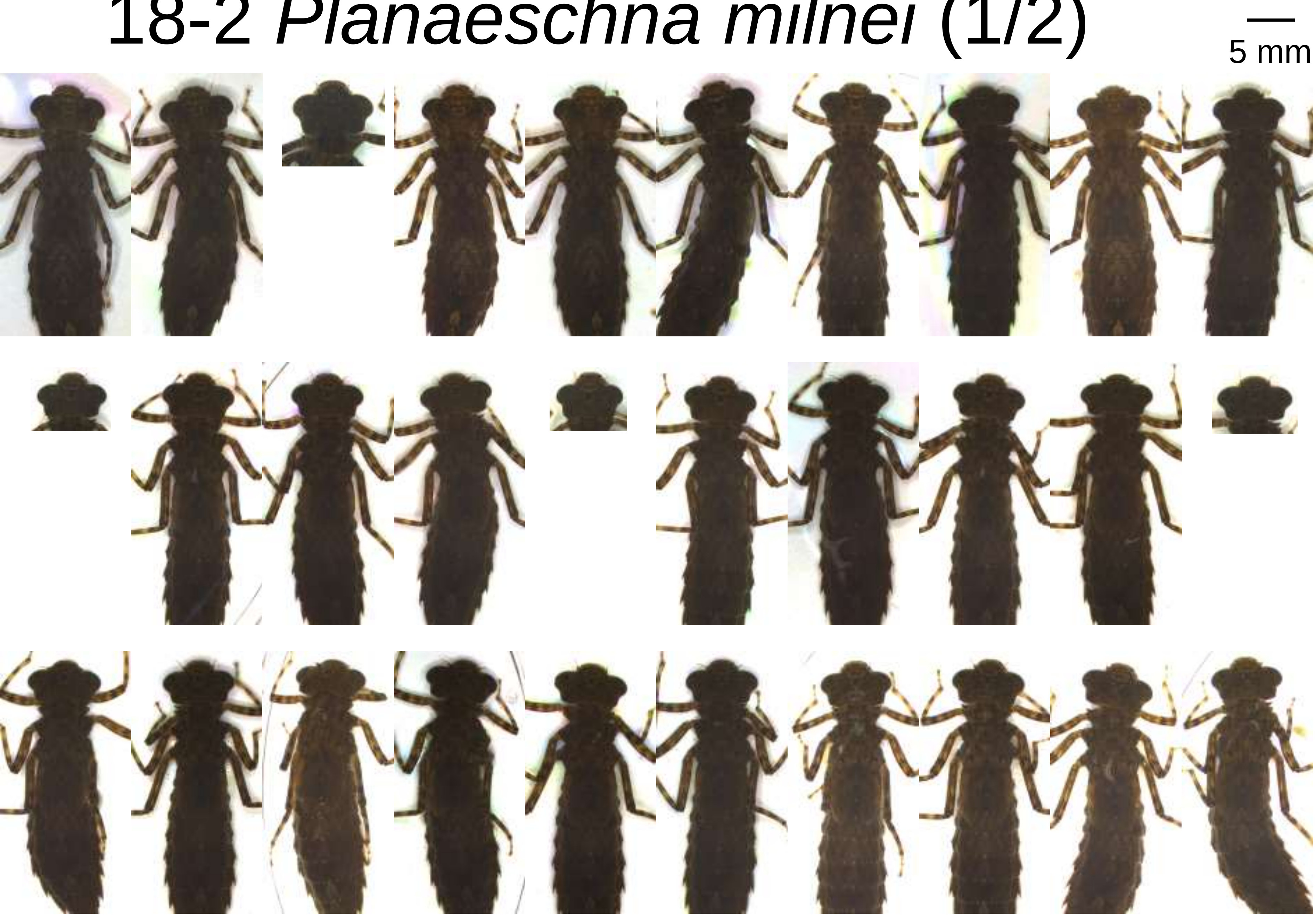

18-2 *Planaeschna milnei* (2/2)

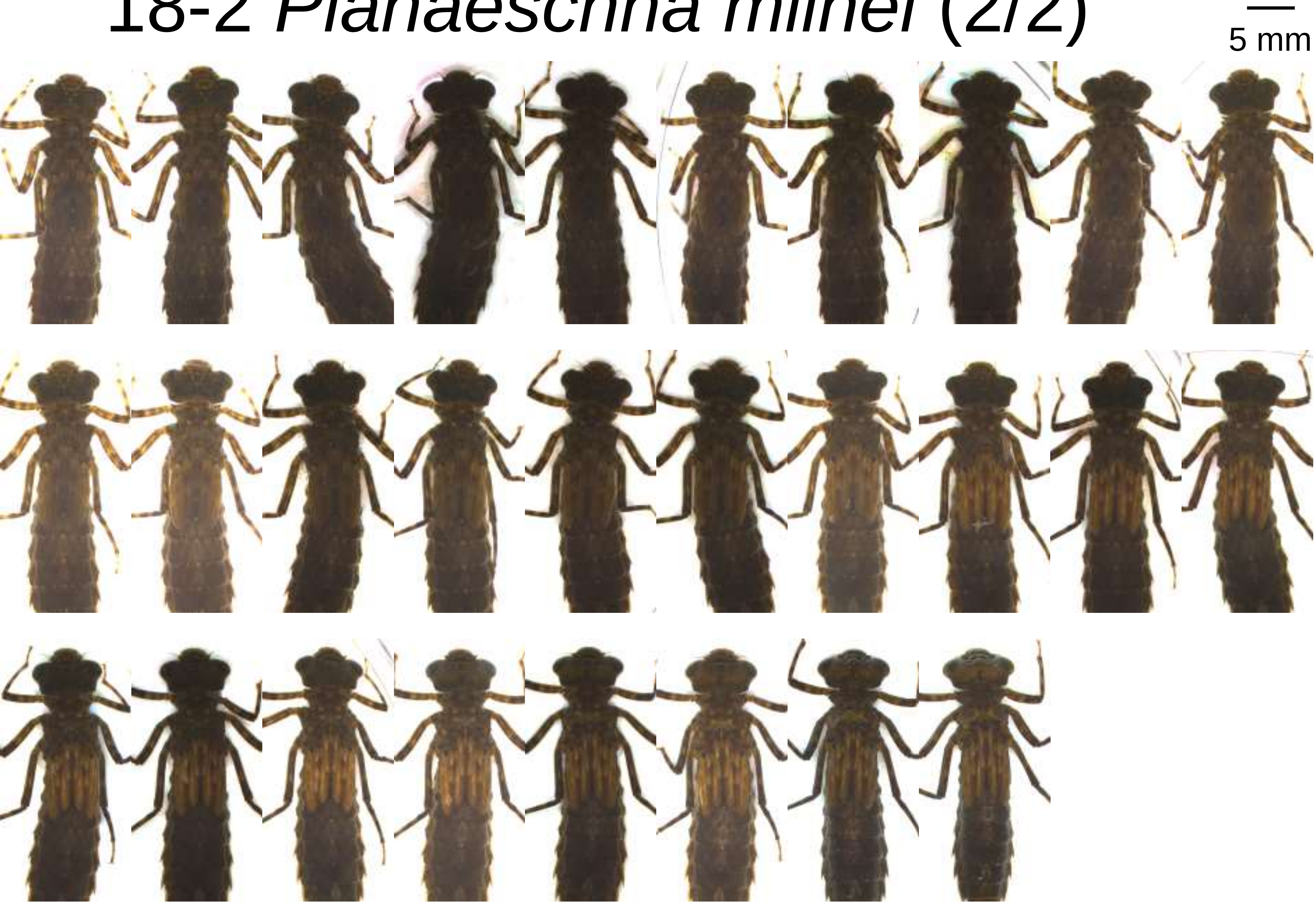

18-3 *Planaeschna milnei* (1/2)

5 mm

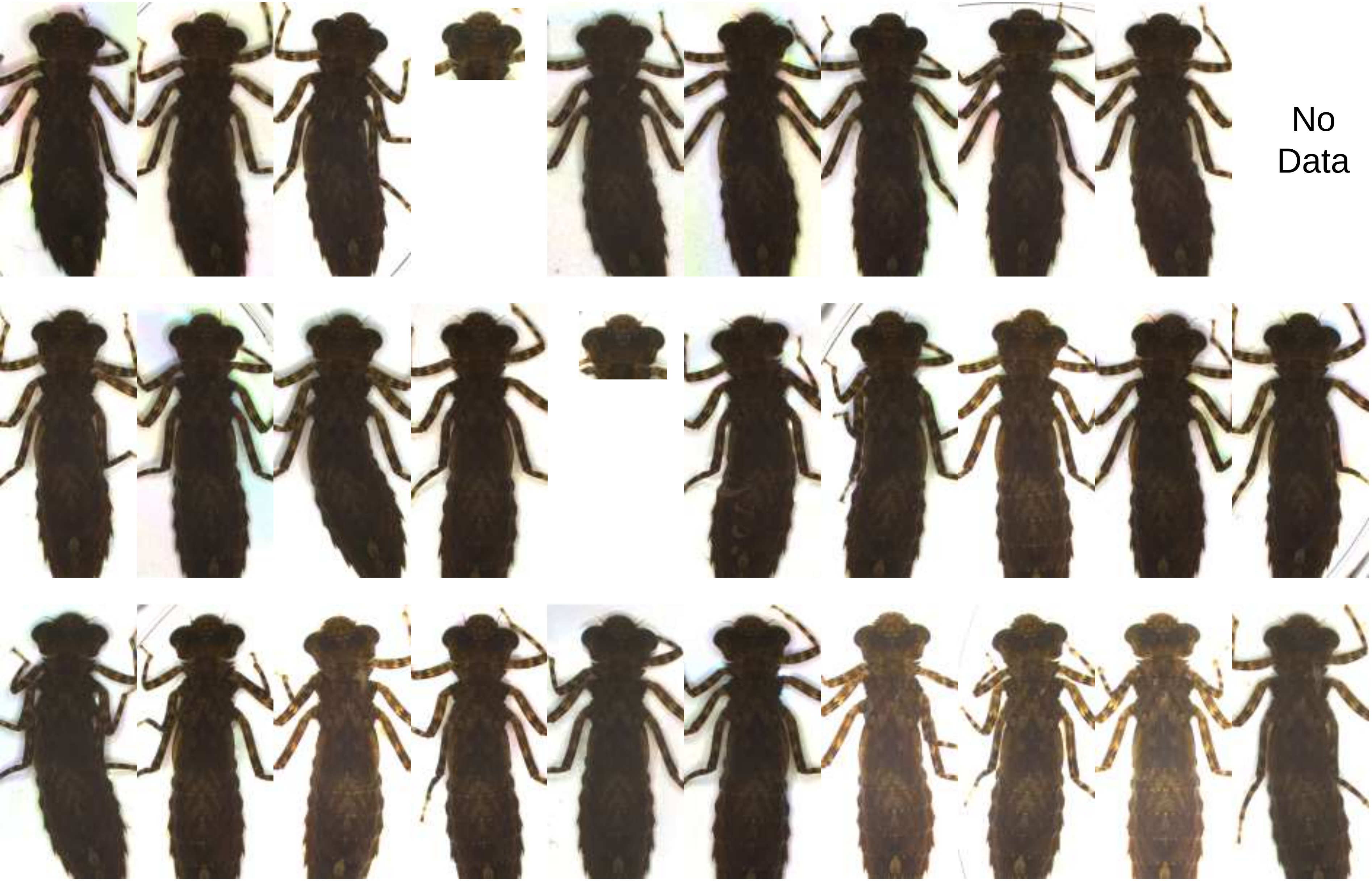

18-3 *Planaeschna milnei* (2/2)

5 mm

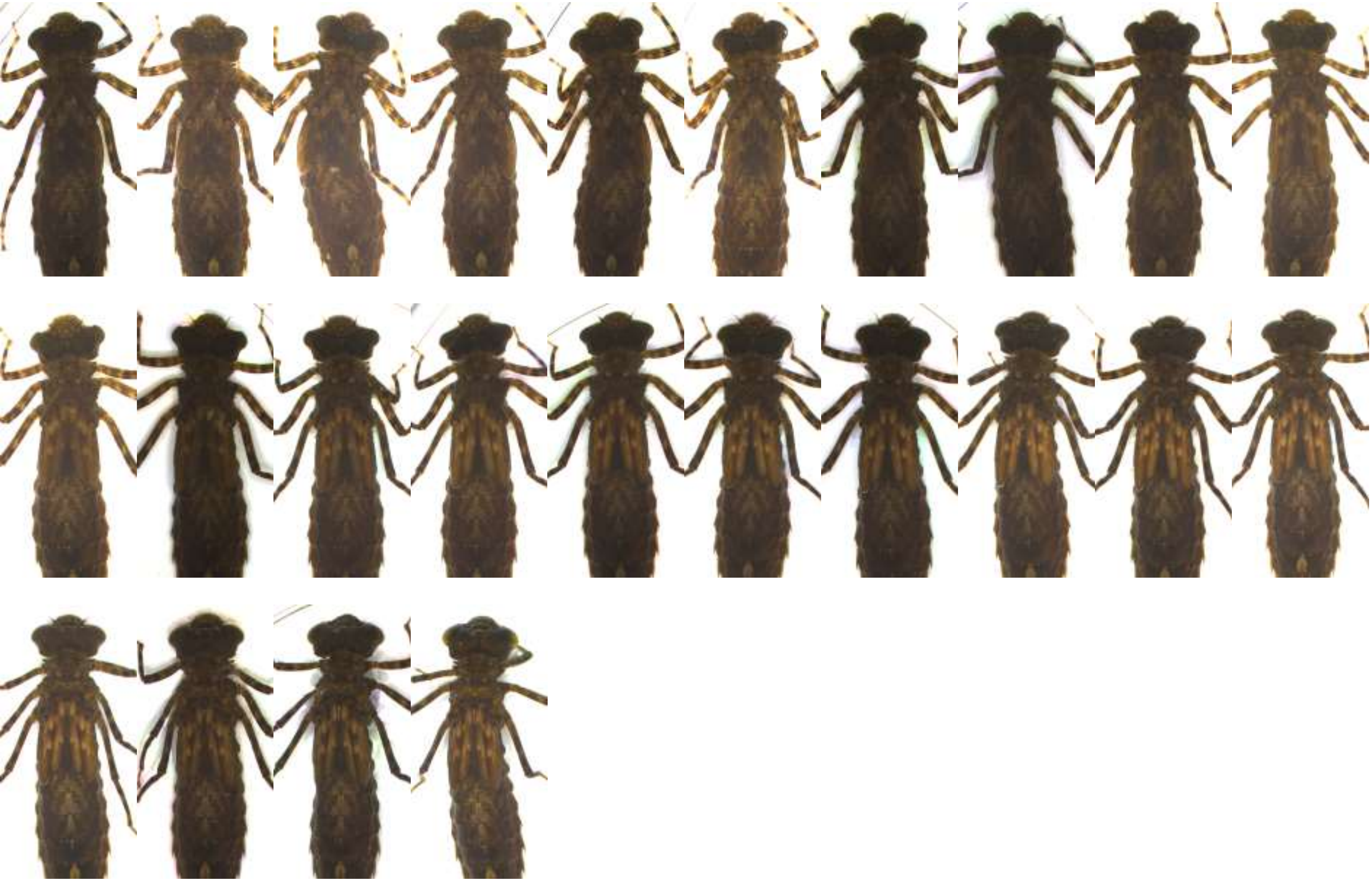

19-1 *Gynacantha japonica* (1/1)

5 mm

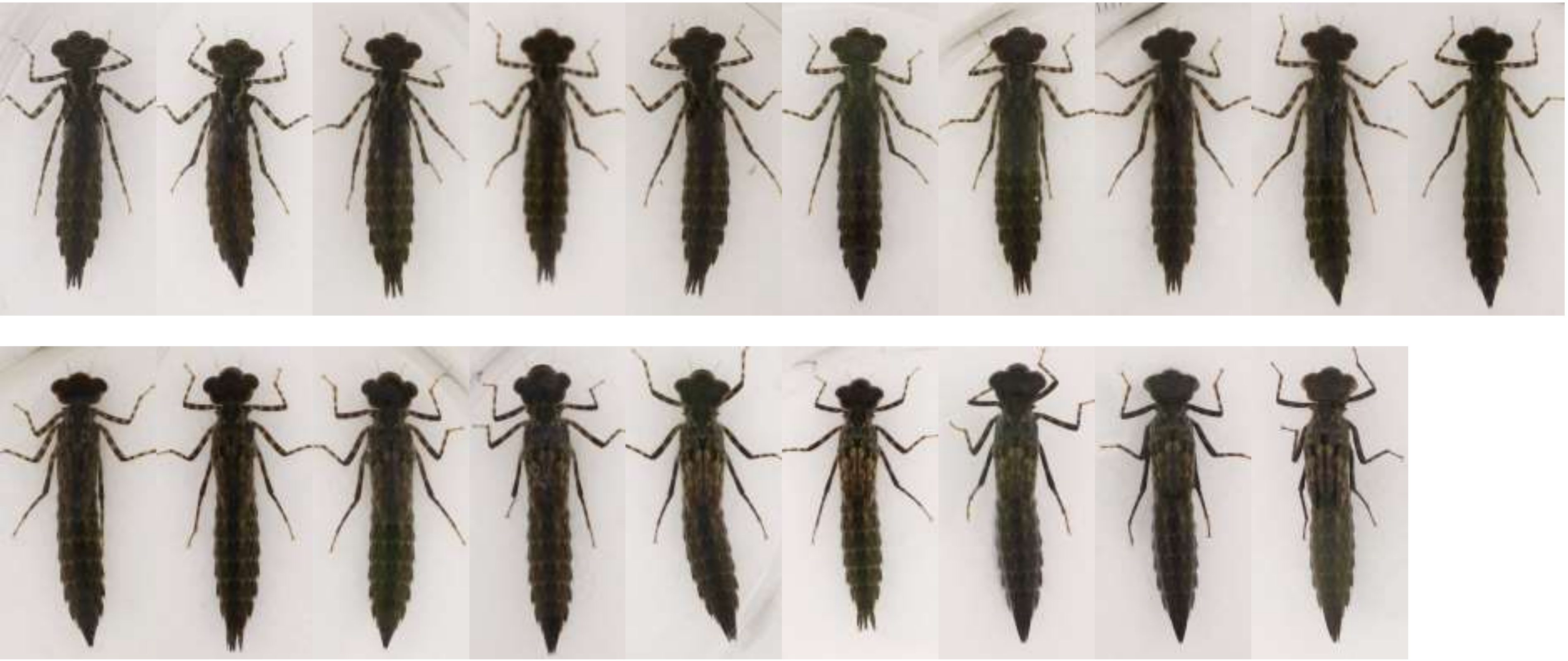

19-2 *Gynacantha japonica* (1/1)

5 mm

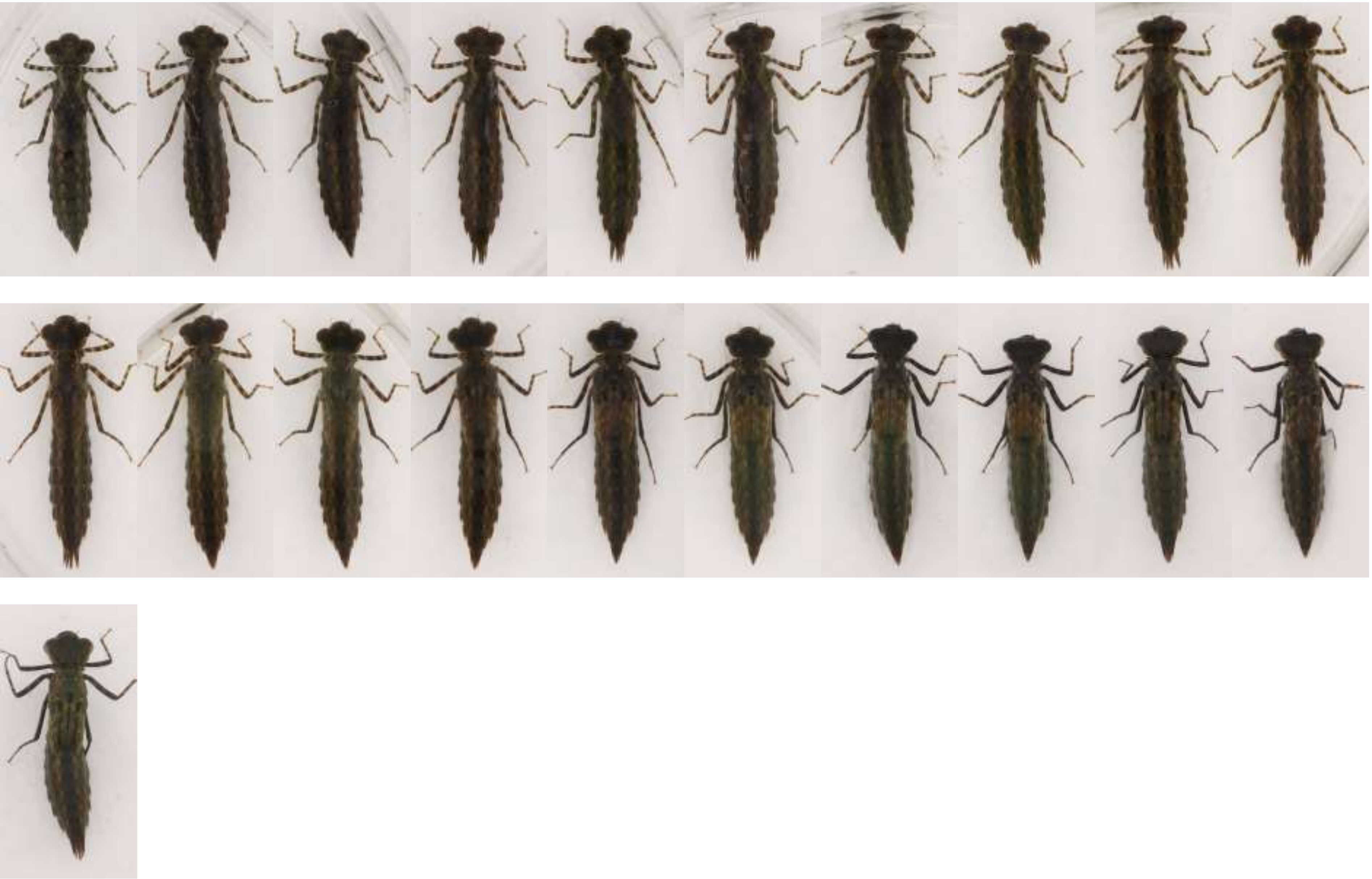

19-4 *Gynacantha japonica* (1/1)

5 mm

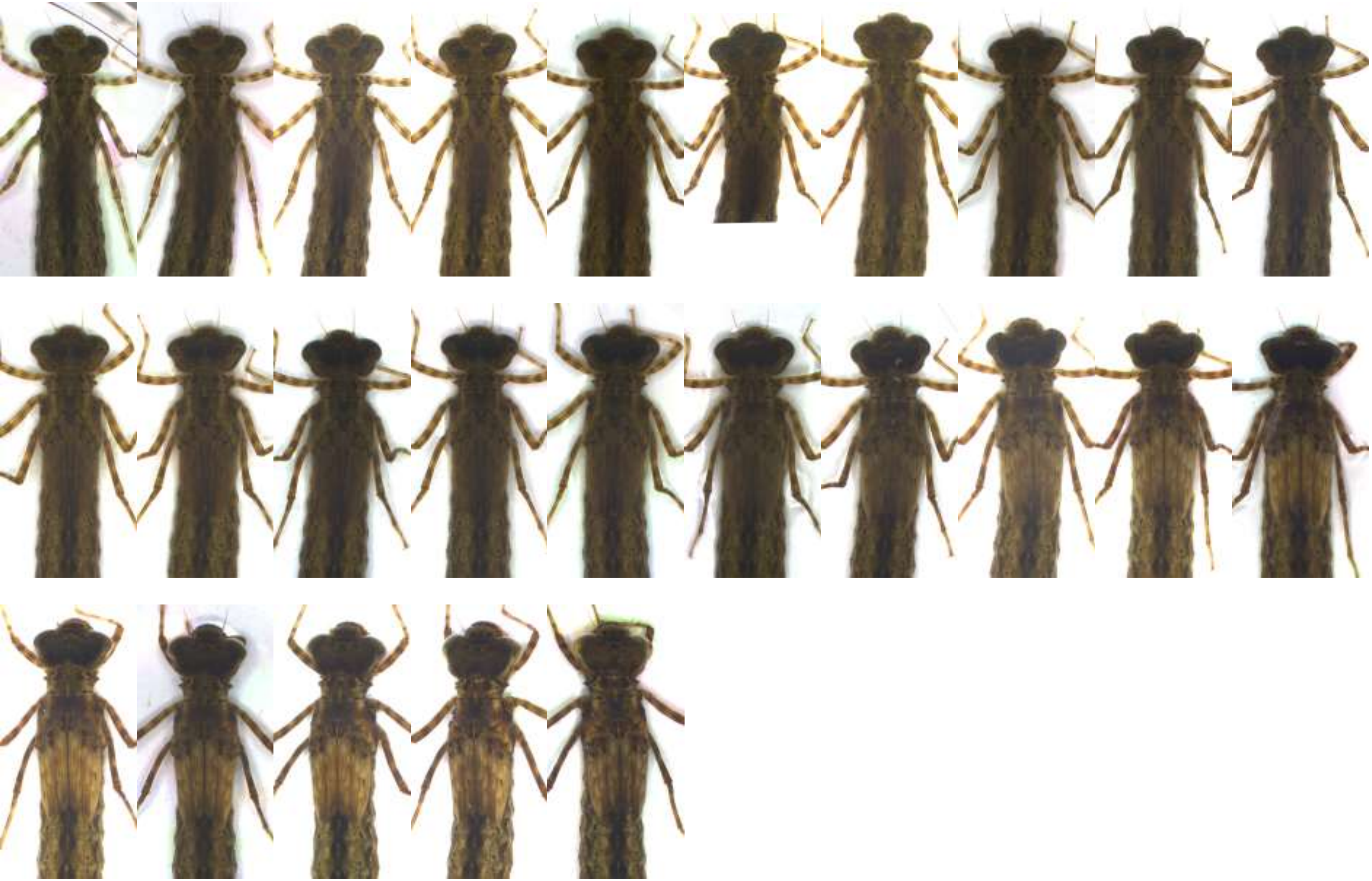

19-5 *Gynacantha japonica* (1/2)

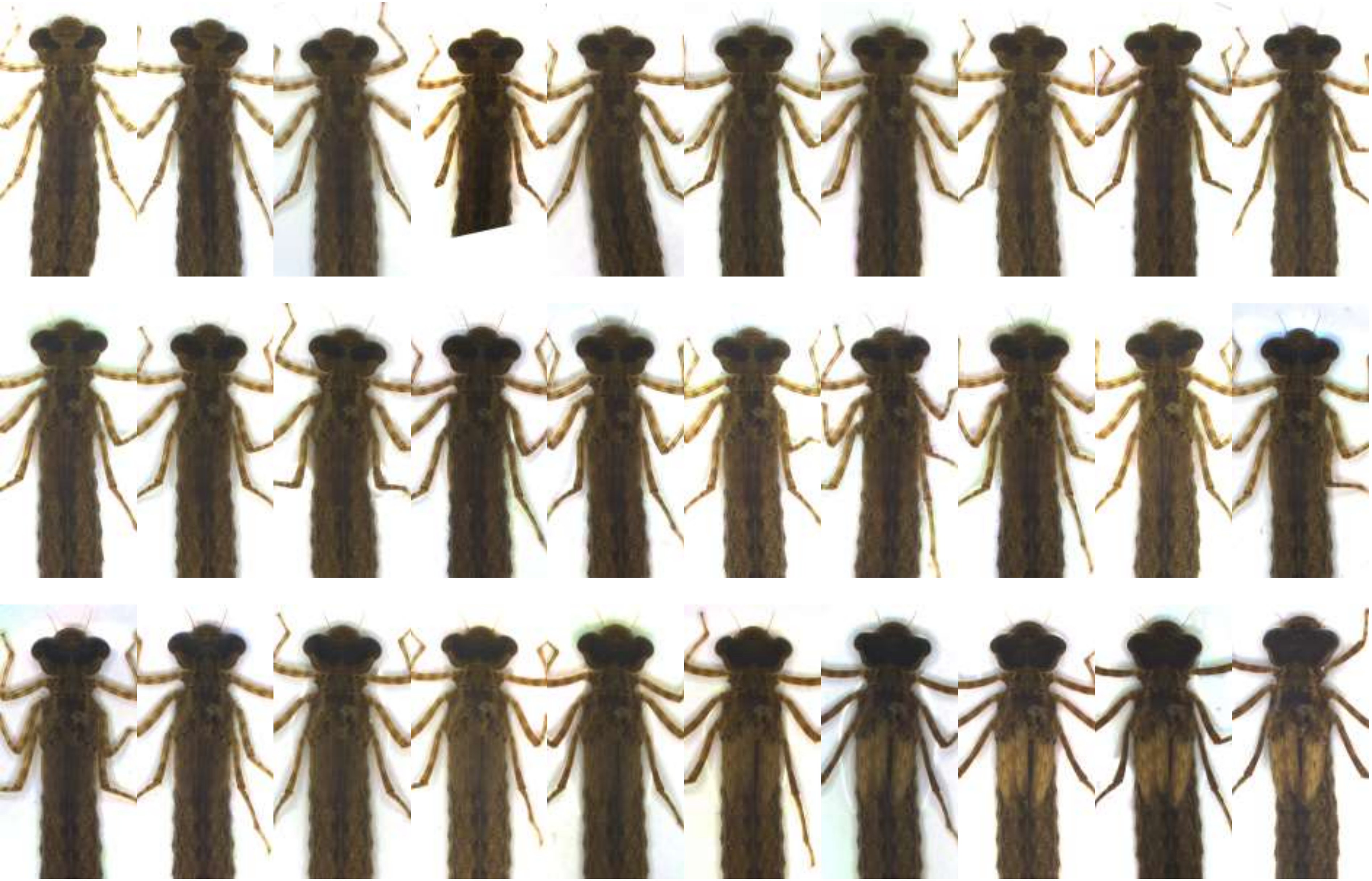

19-5 *Gynacantha japonica* (2/2)

5 mm

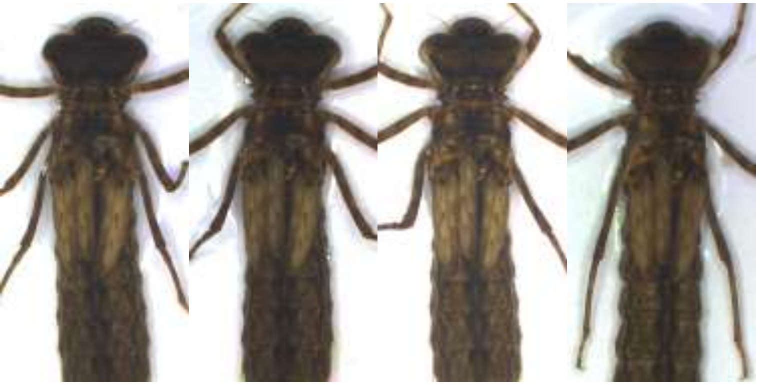

20-1 *Aeshna crenata* (1/3)

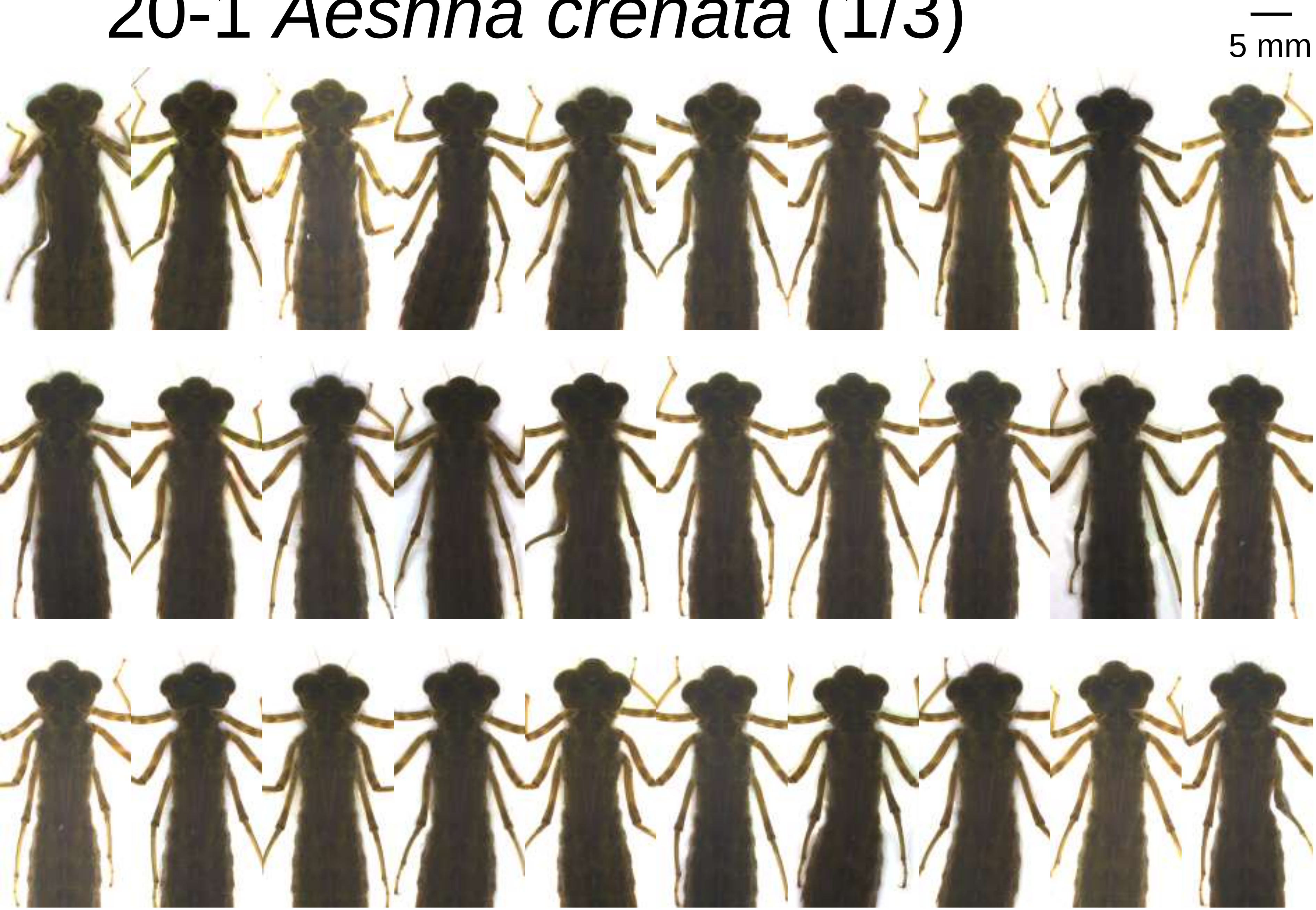

20-1 *Aeshna crenata* (2/3)

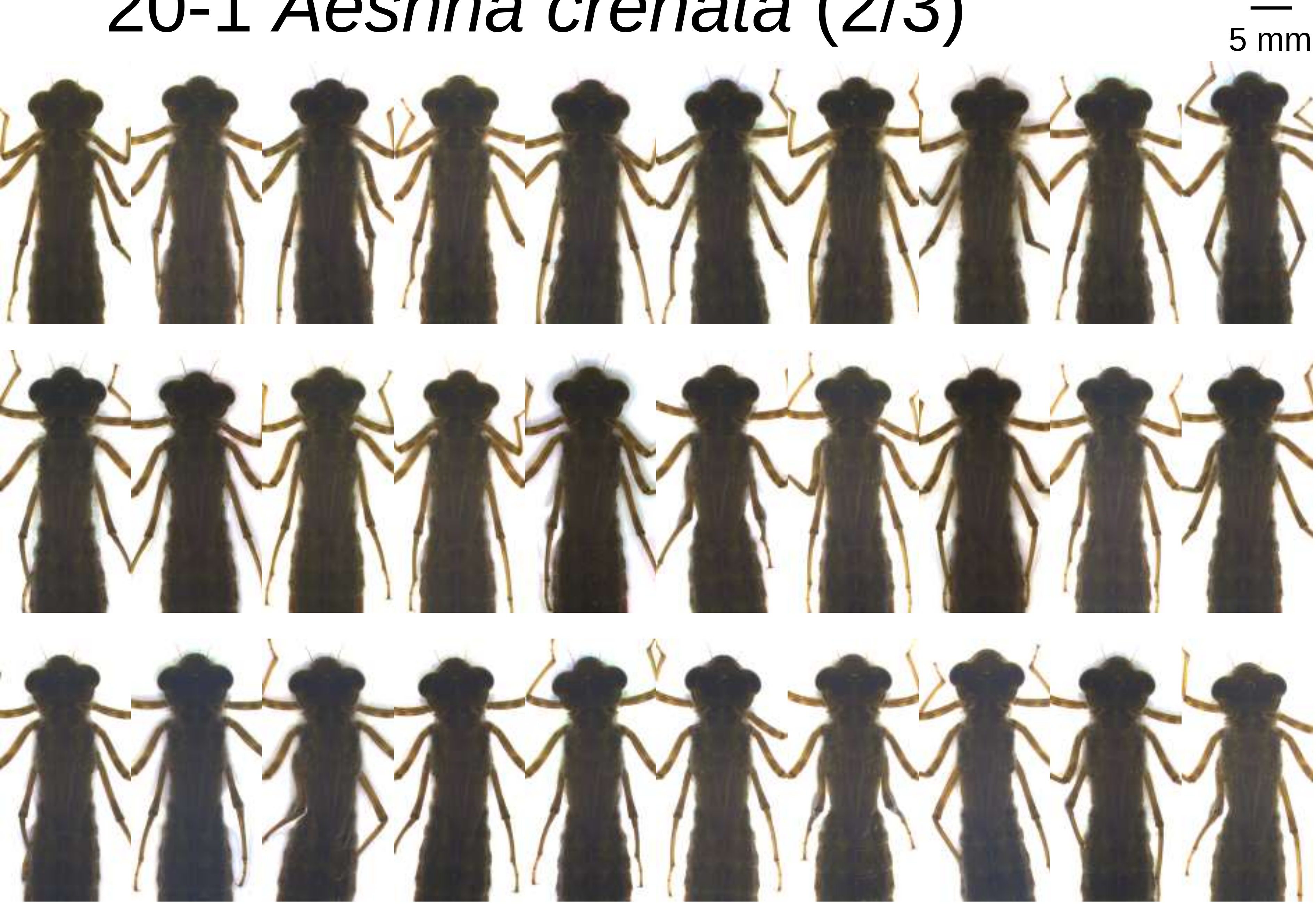

20-1 *Aeshna crenata* (3/3)

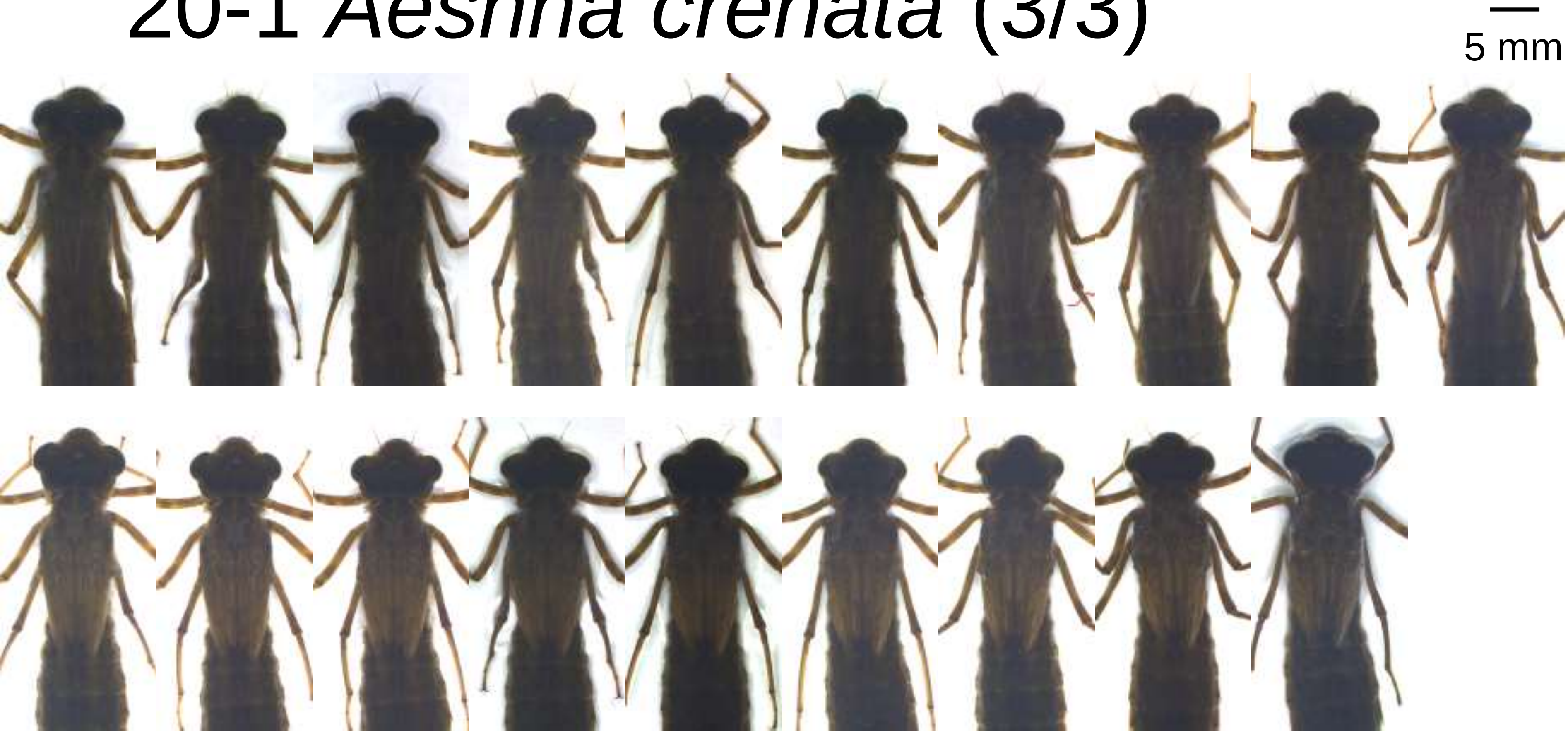

20-2 *Aeshna crenata* (1/2)

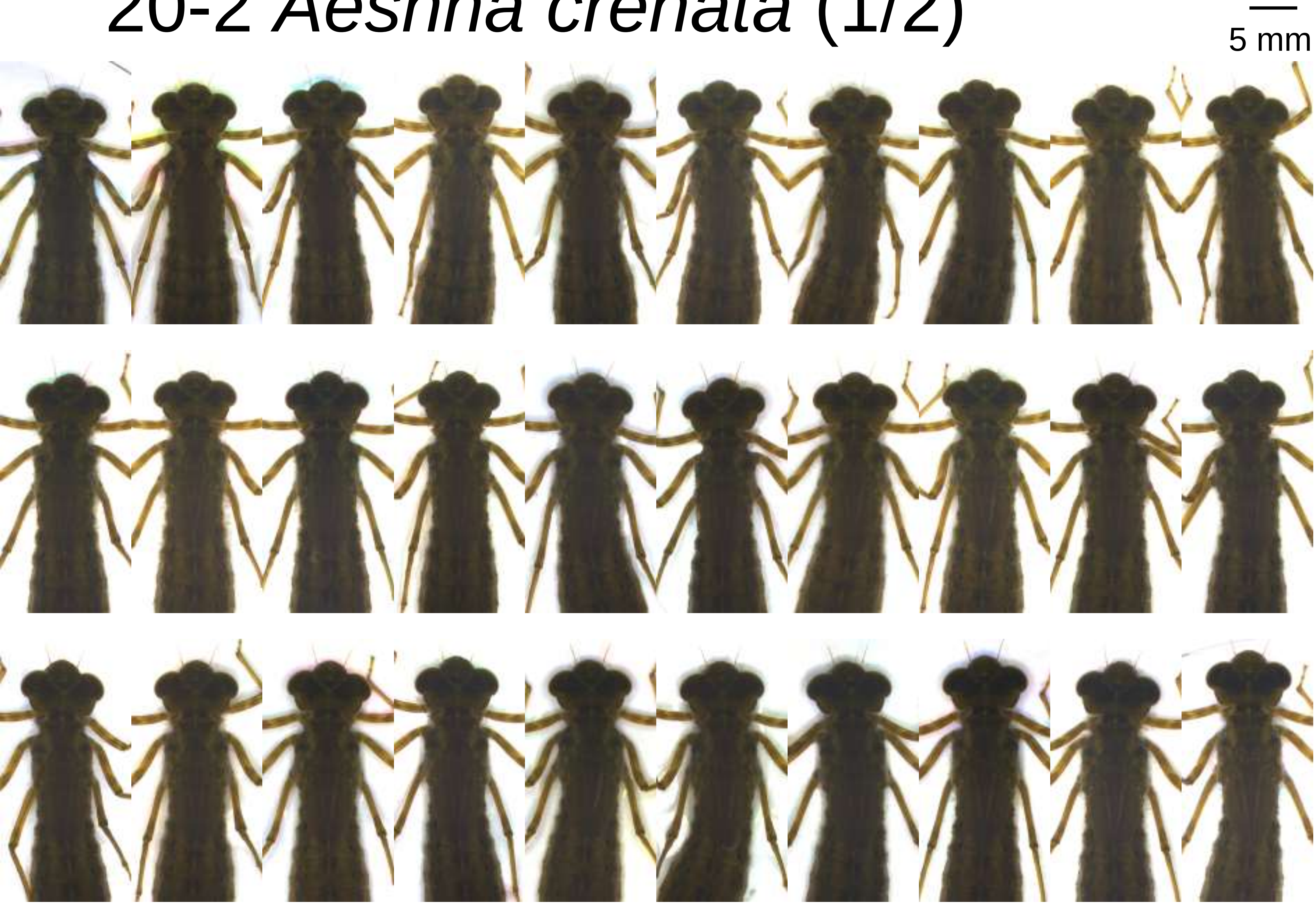

20-2 *Aeshna crenata* (2/2)

5 mm

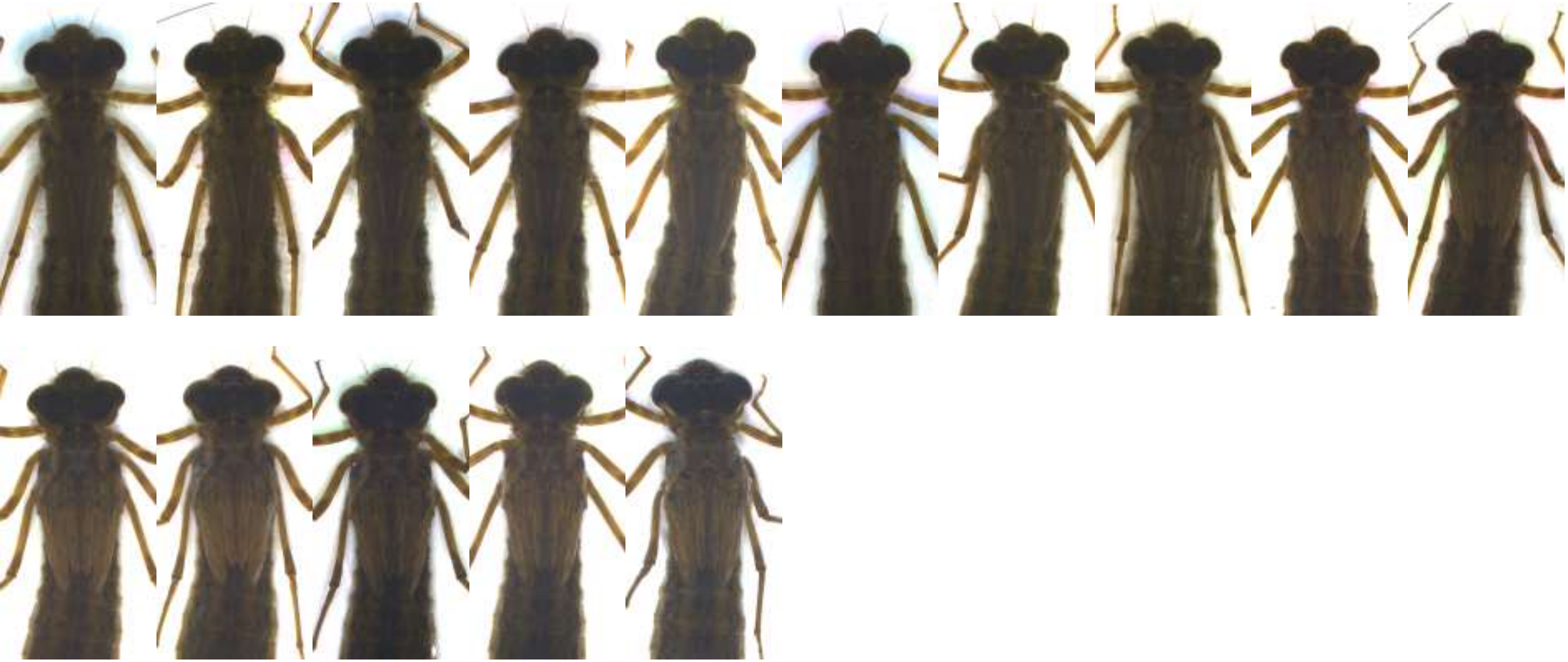

20-3 *Aeshna crenata* (1/1)

5 mm

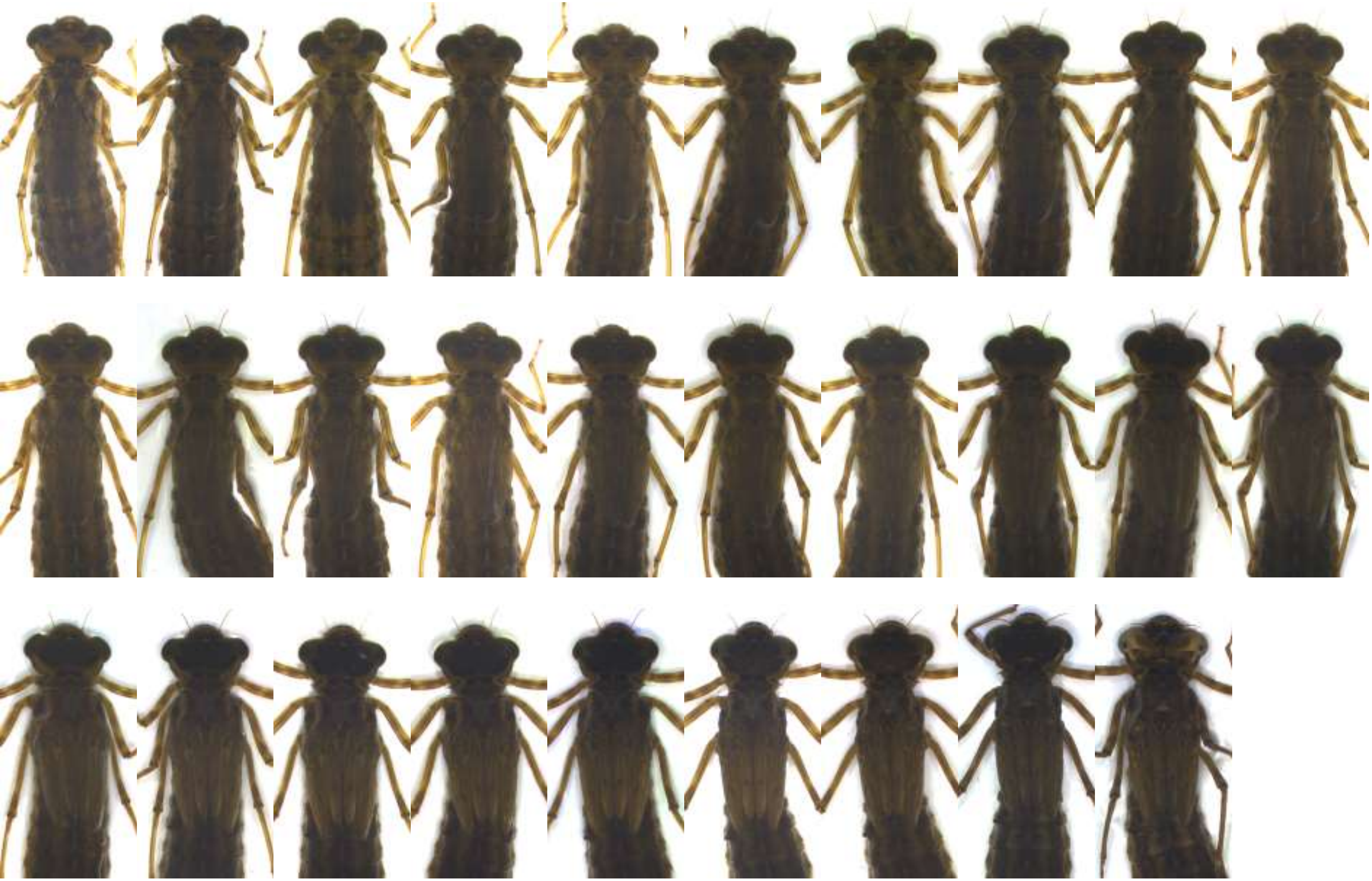

20-4 *Aeshna crenata* (1/1)

5 mm

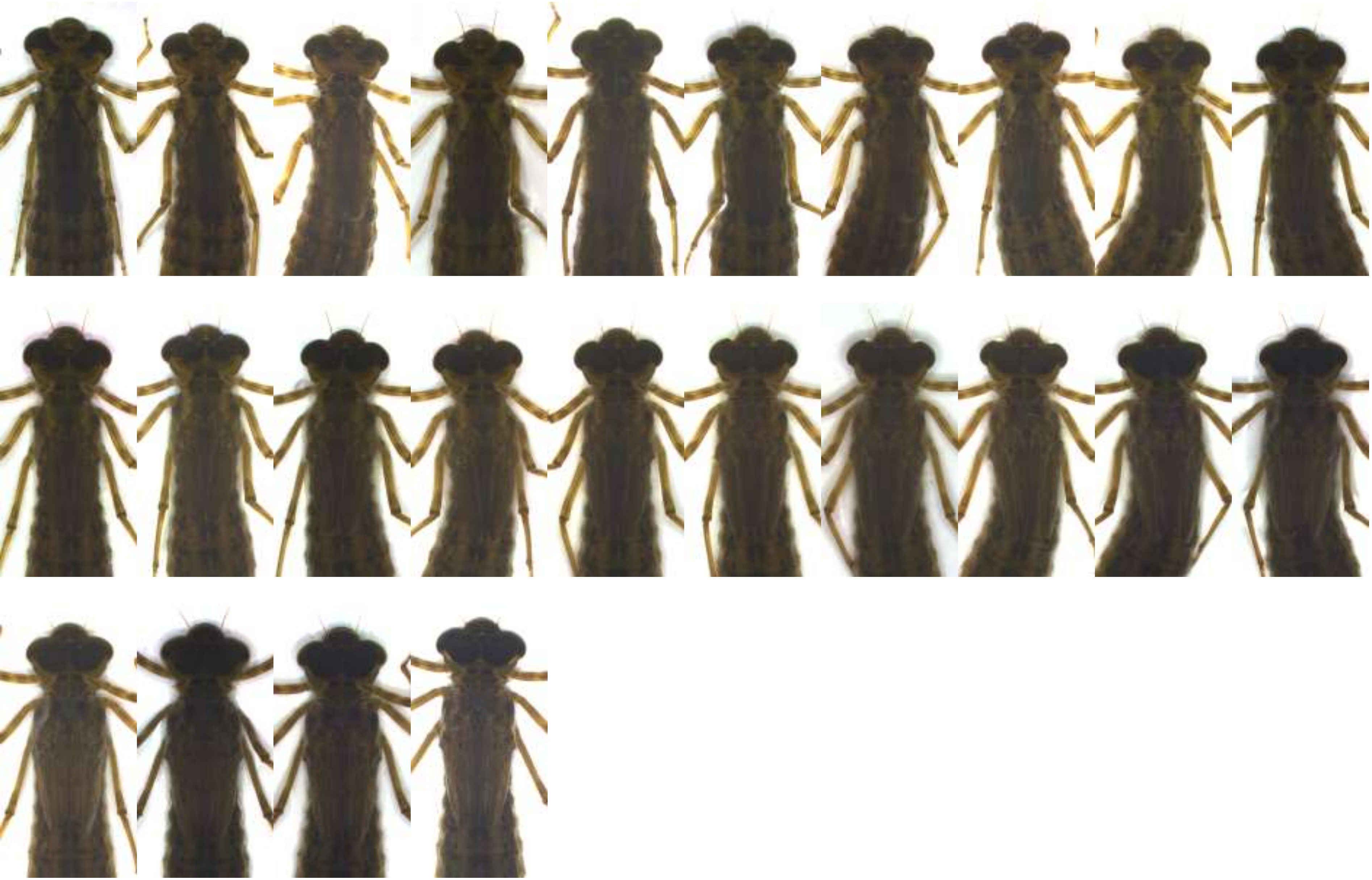

21-1 *Aeshna juncea* (1/1)

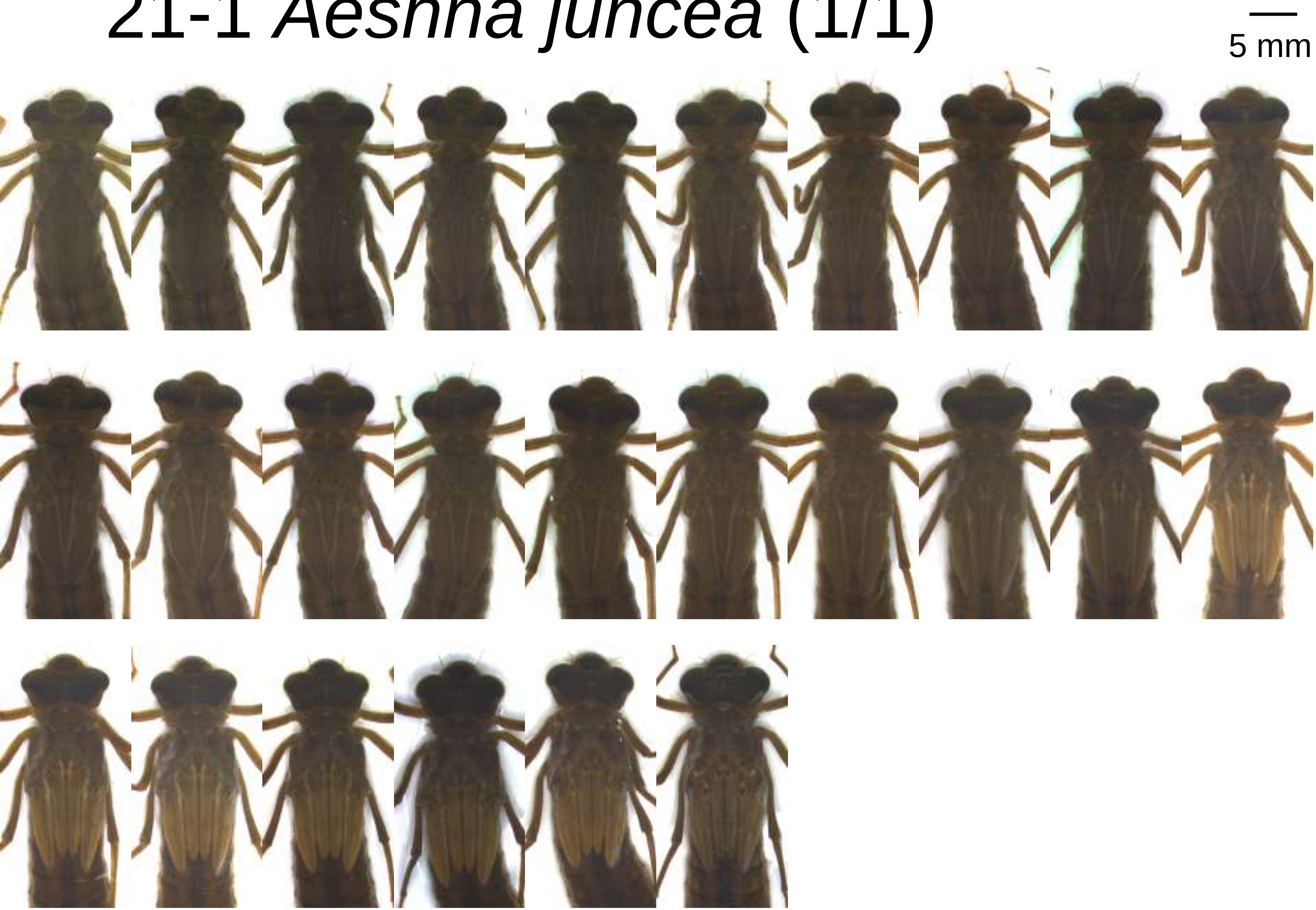

22-4 *Anax ephippiger* (1/1)

–
2 mm

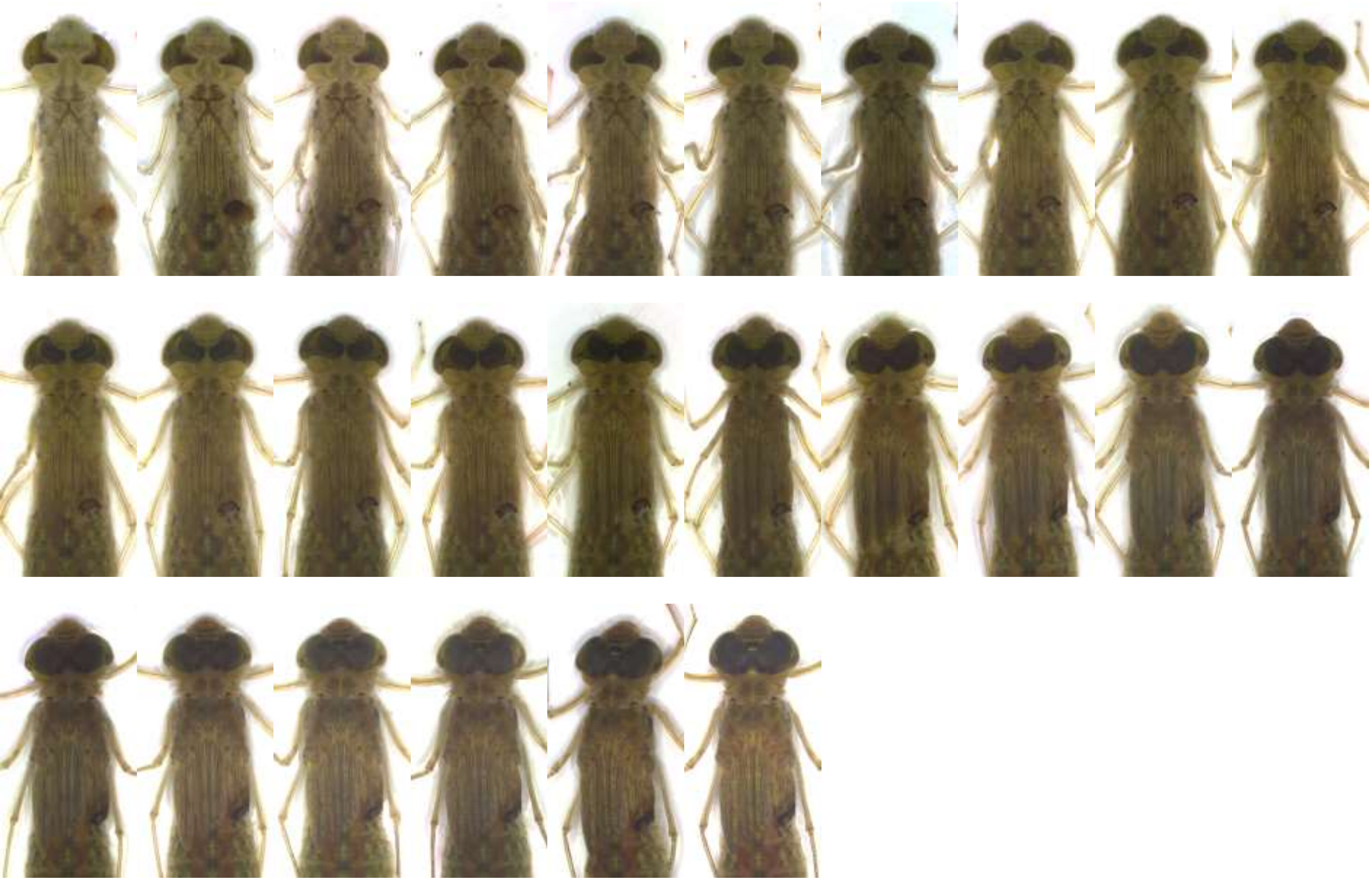

22-5 *Anax ephippiger* (1/1)

2 mm

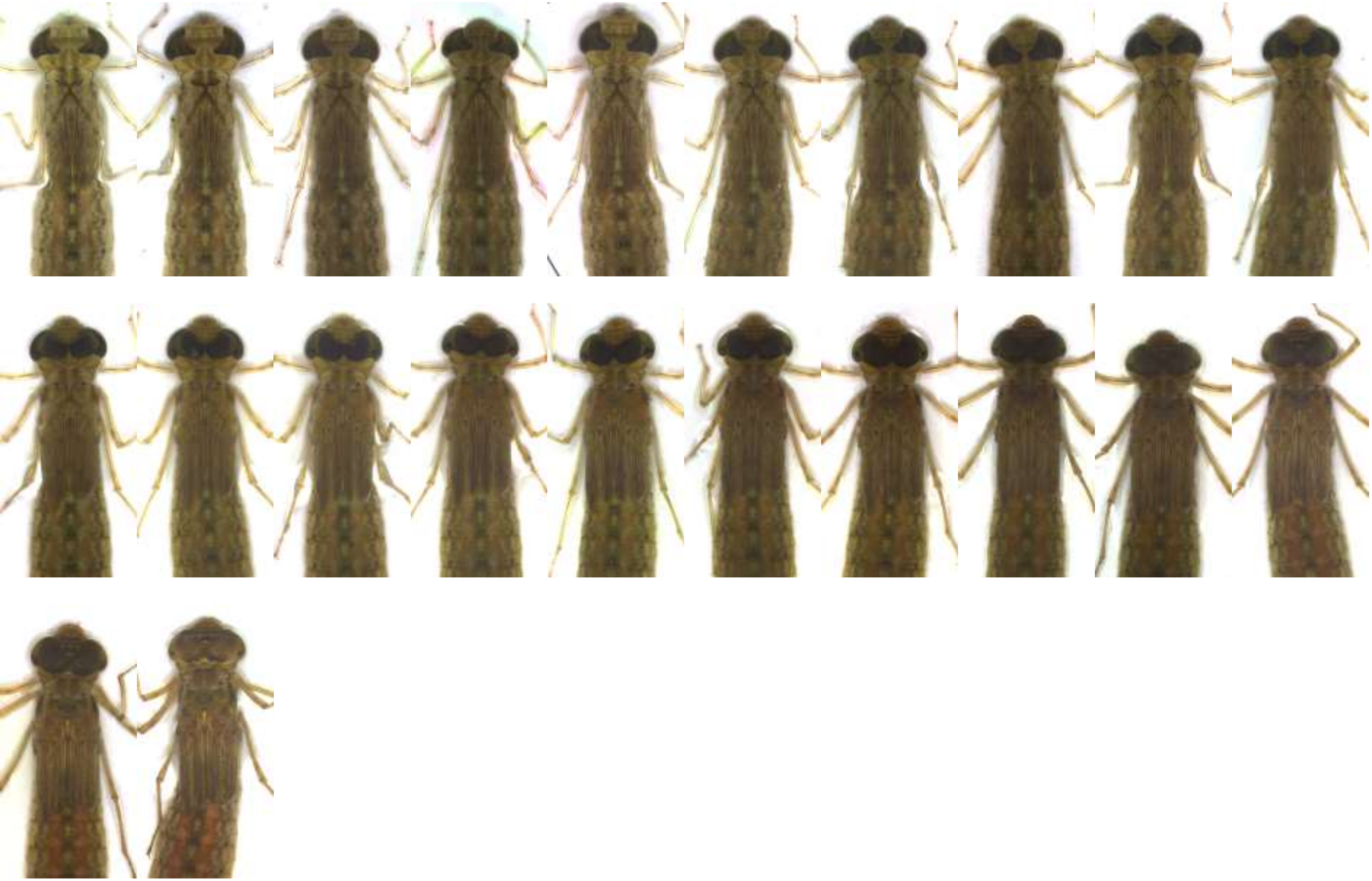

22-6 *Anax ephippiger* (1/1)

2 mm

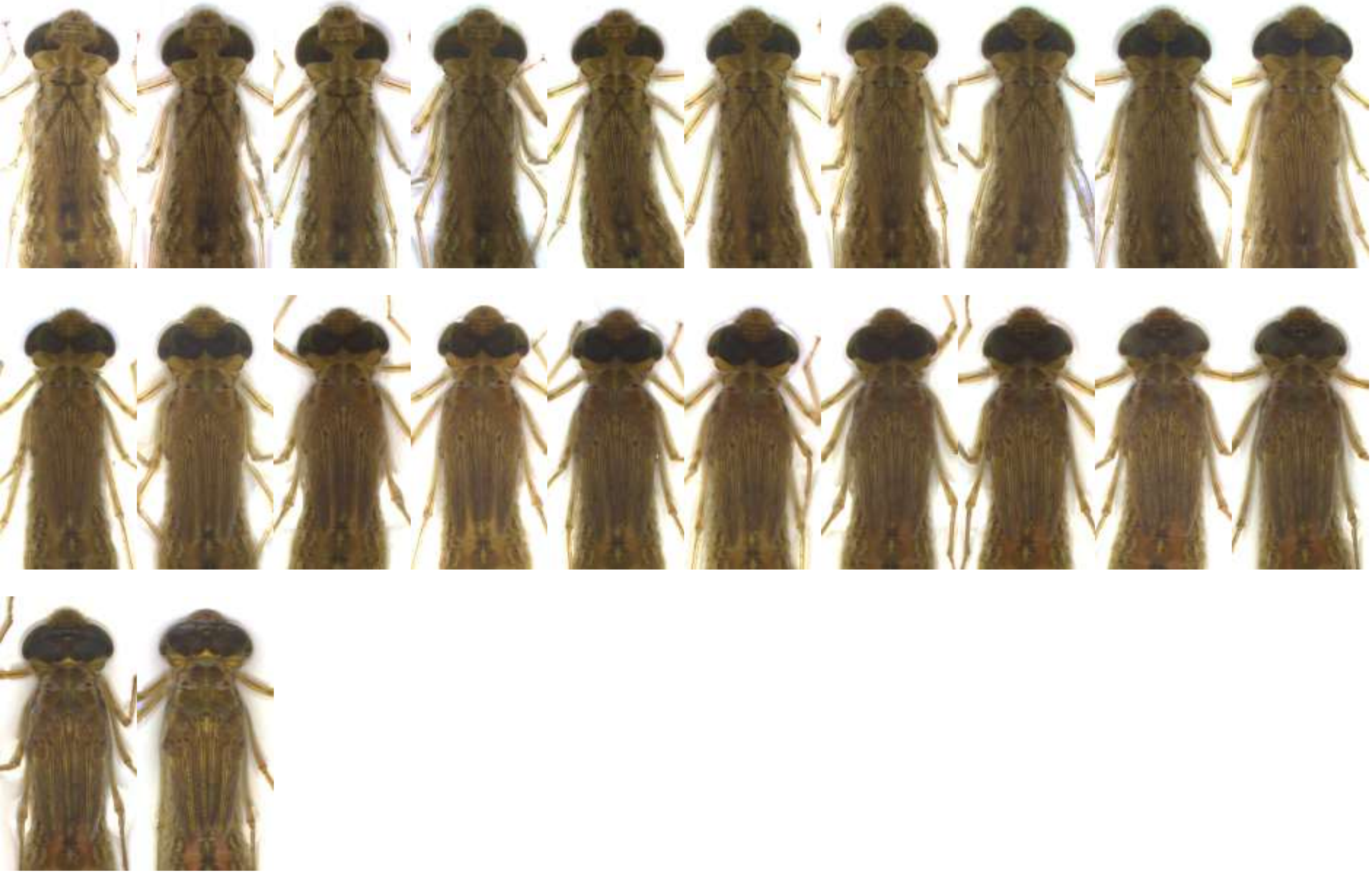

22-7 *Anax ephippiger* (1/1)

2 mm

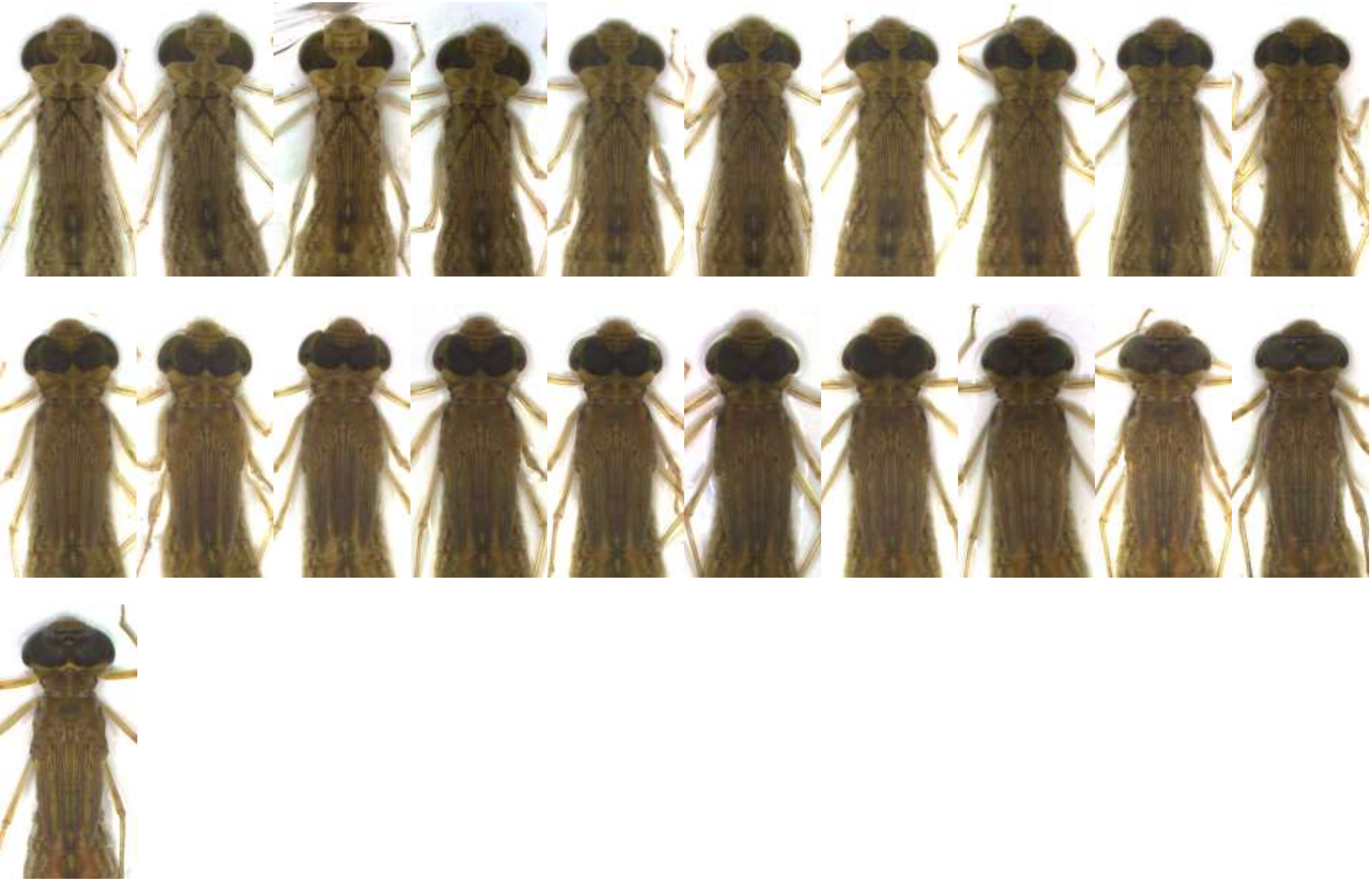

22-8 *Anax ephippiger* (1/1)

2 mm

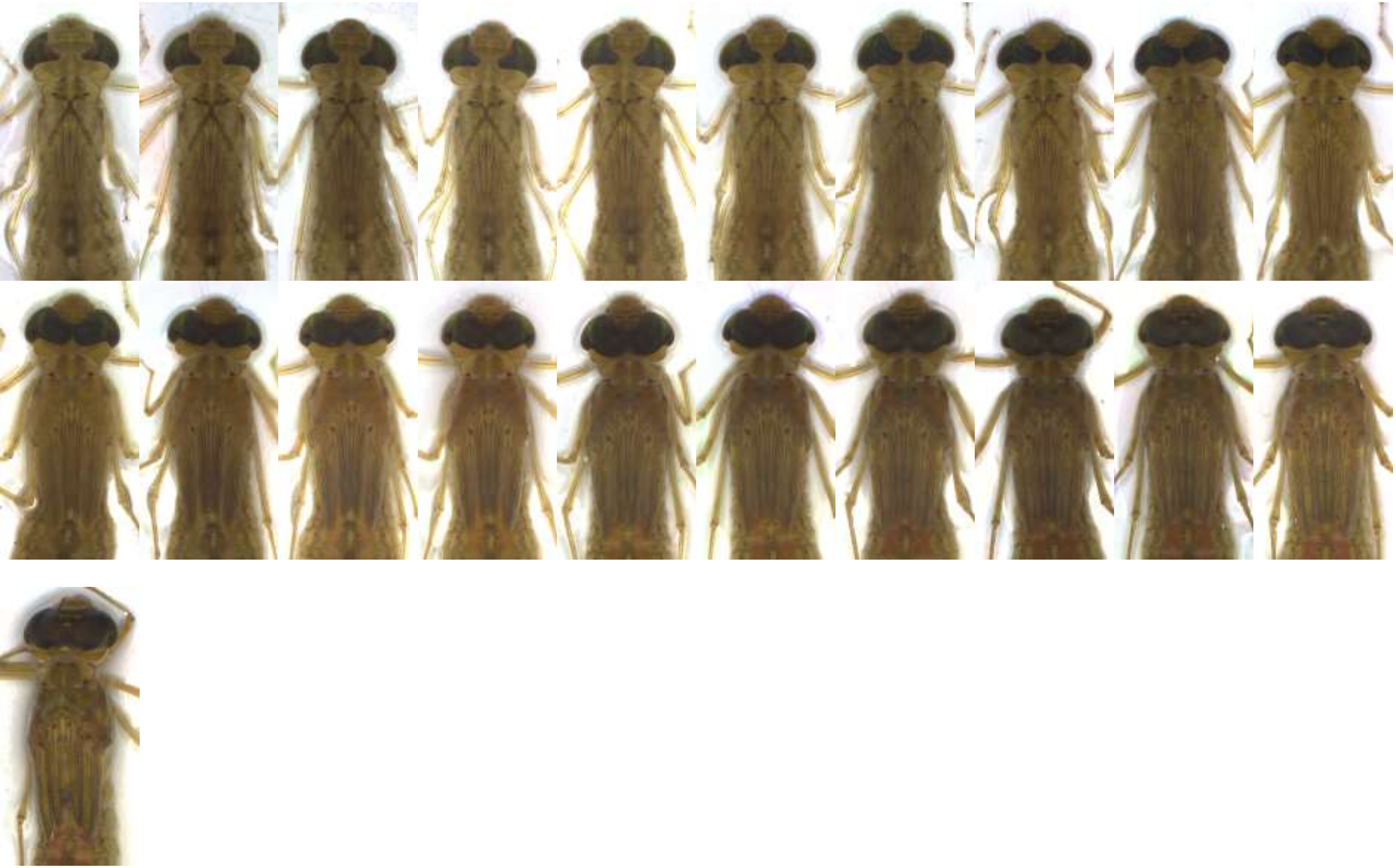

23-1 *Anax parthenope* (1/2)

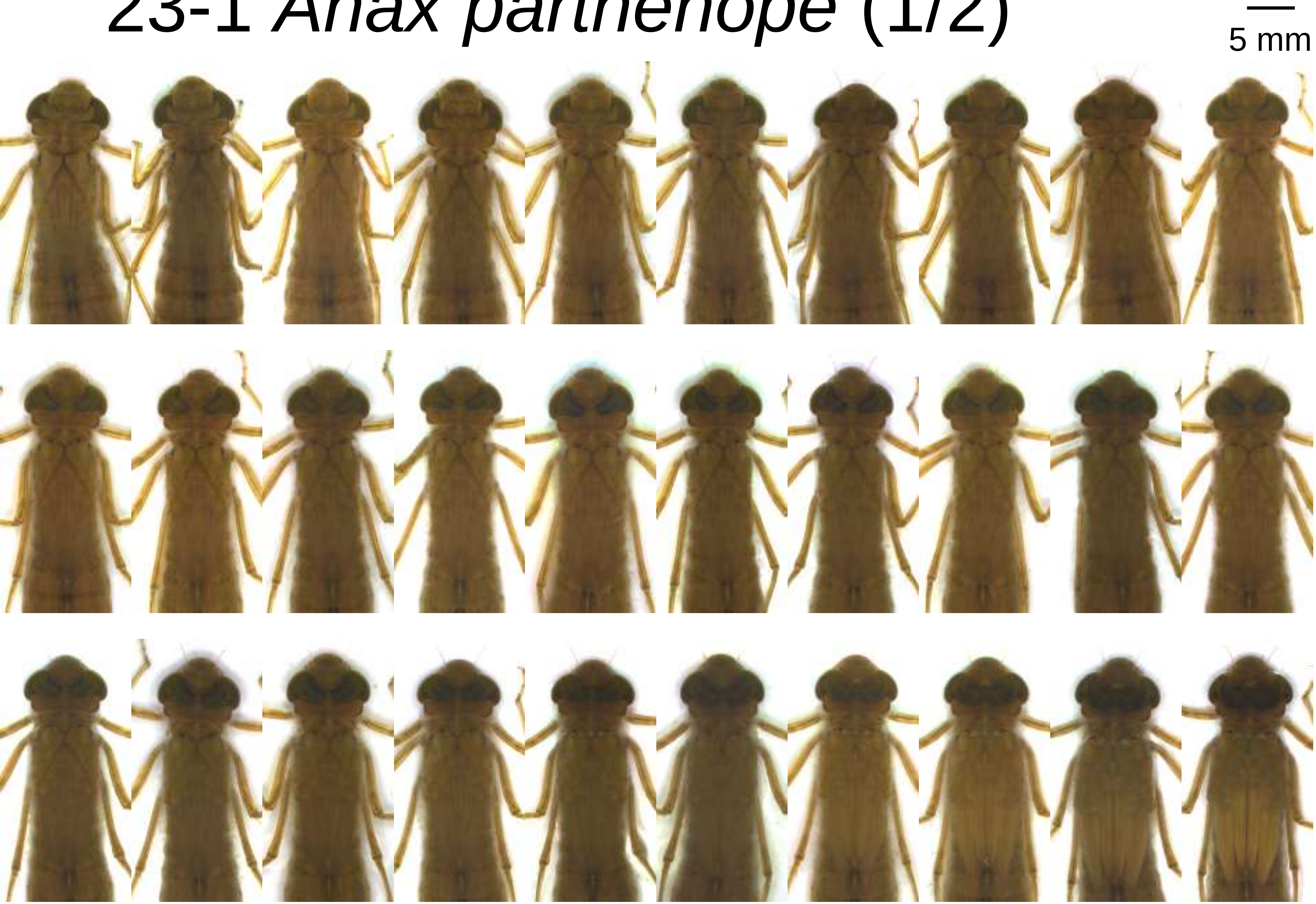

23-1 *Anax parthenope* (2/2)

5 mm

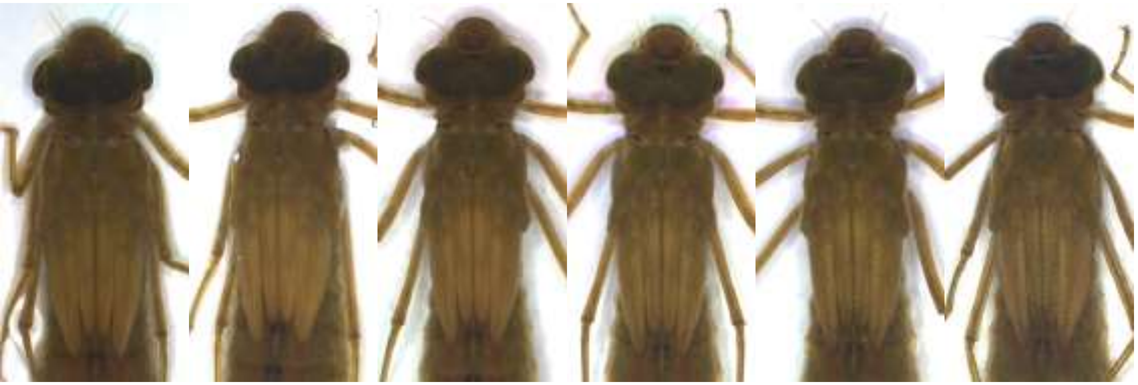

24-1 *Anax nigrofasciatus* (1/2)

5 mm

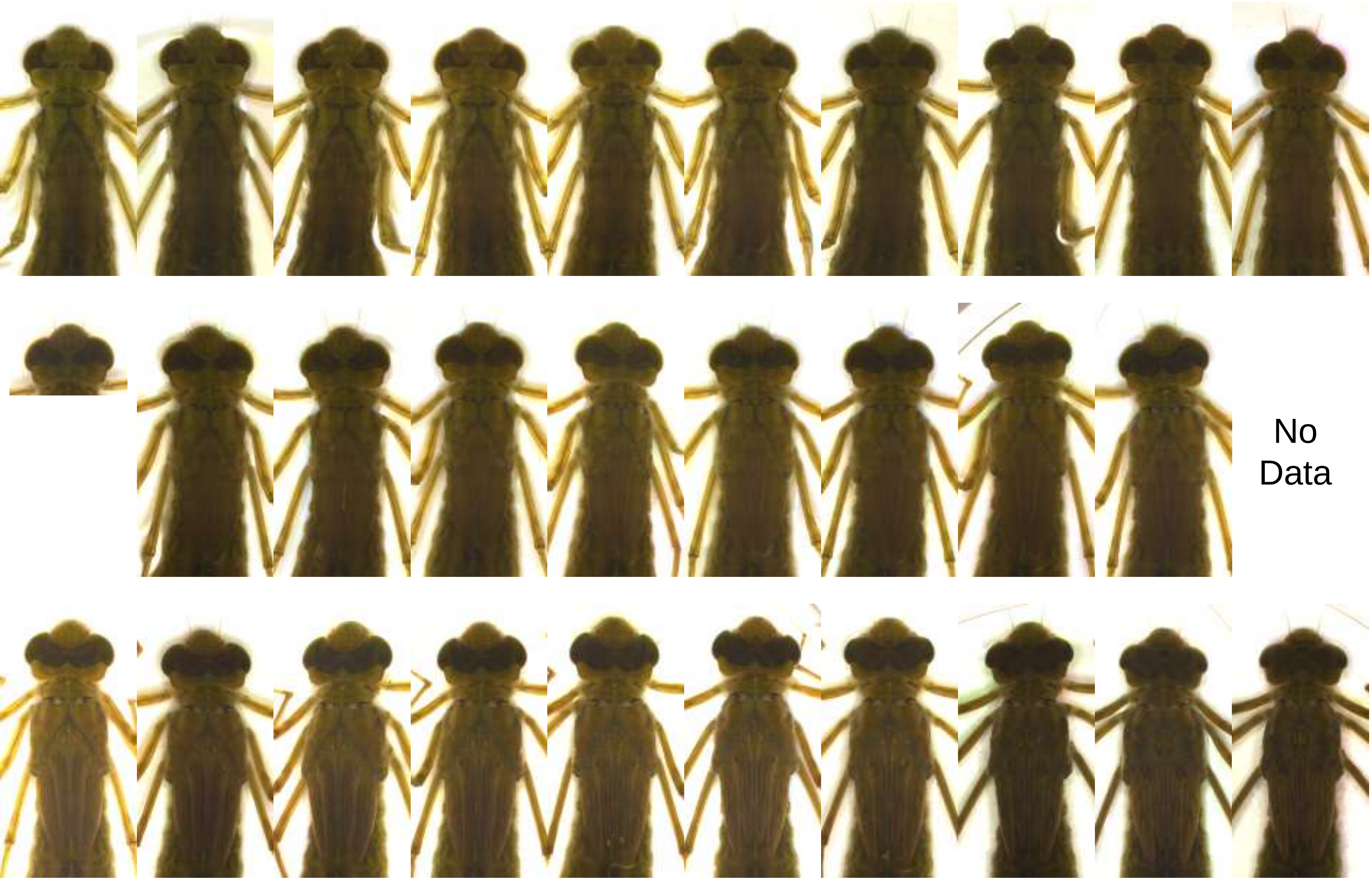

24-1 *Anax nigrofasciatus* (2/2)

5 mm

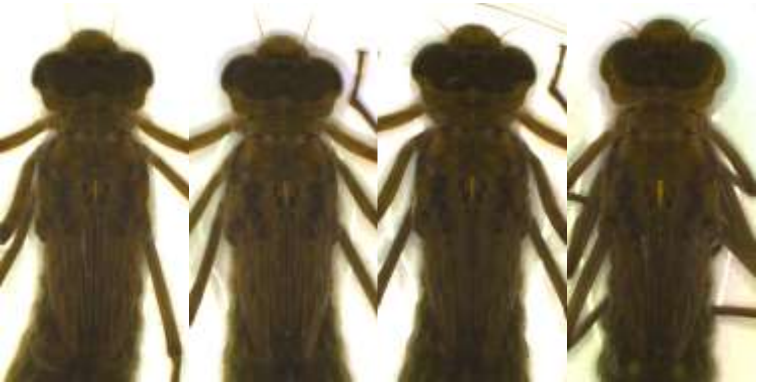

25-1 *Sieboldius albardae* (1/2)

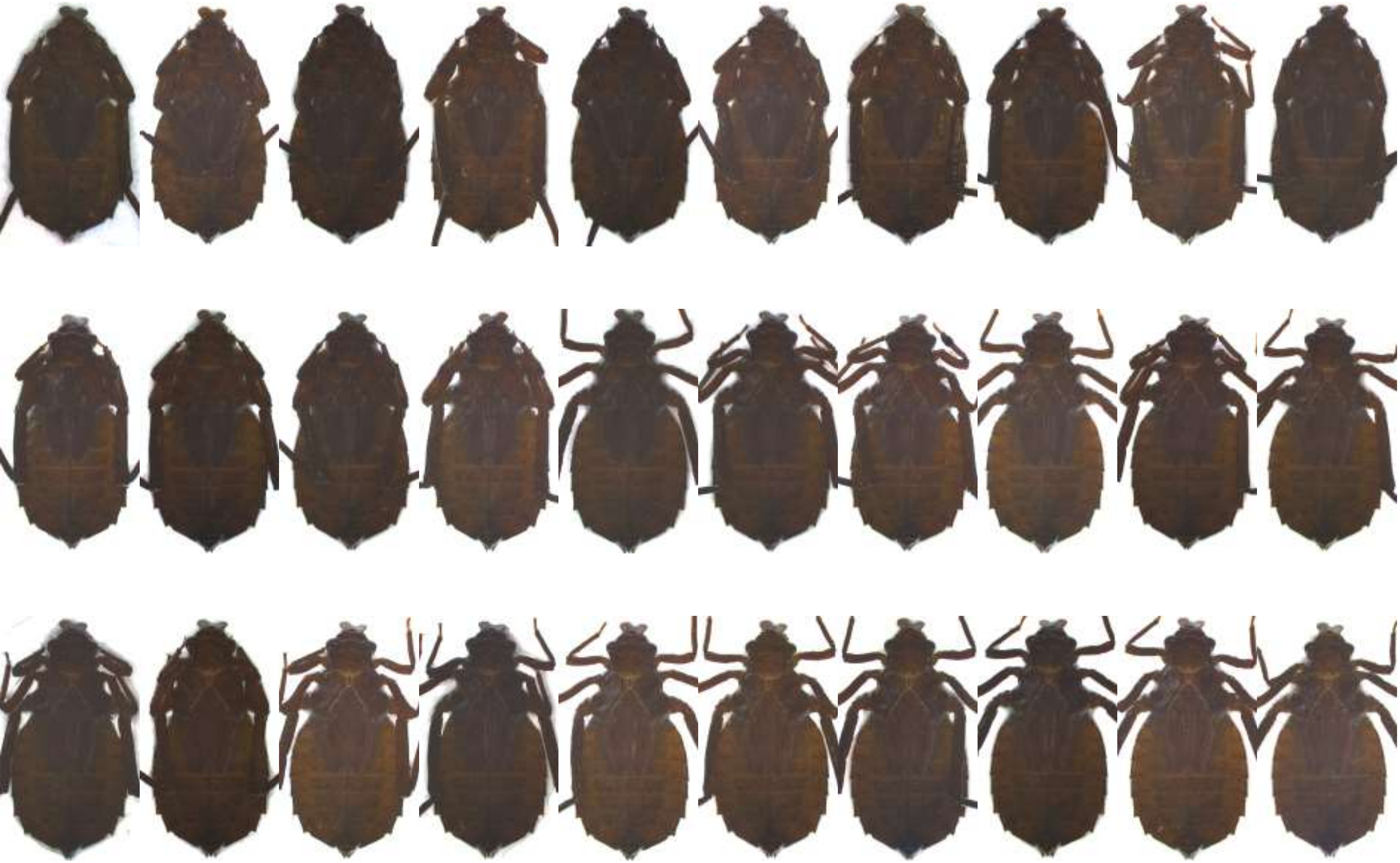

25-1 *Sieboldius albardae* (2/2)

5 mm

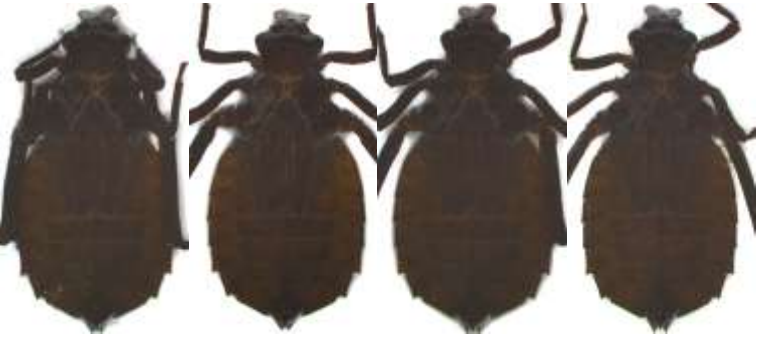

25-2 *Sieboldius albardae* (1/2)

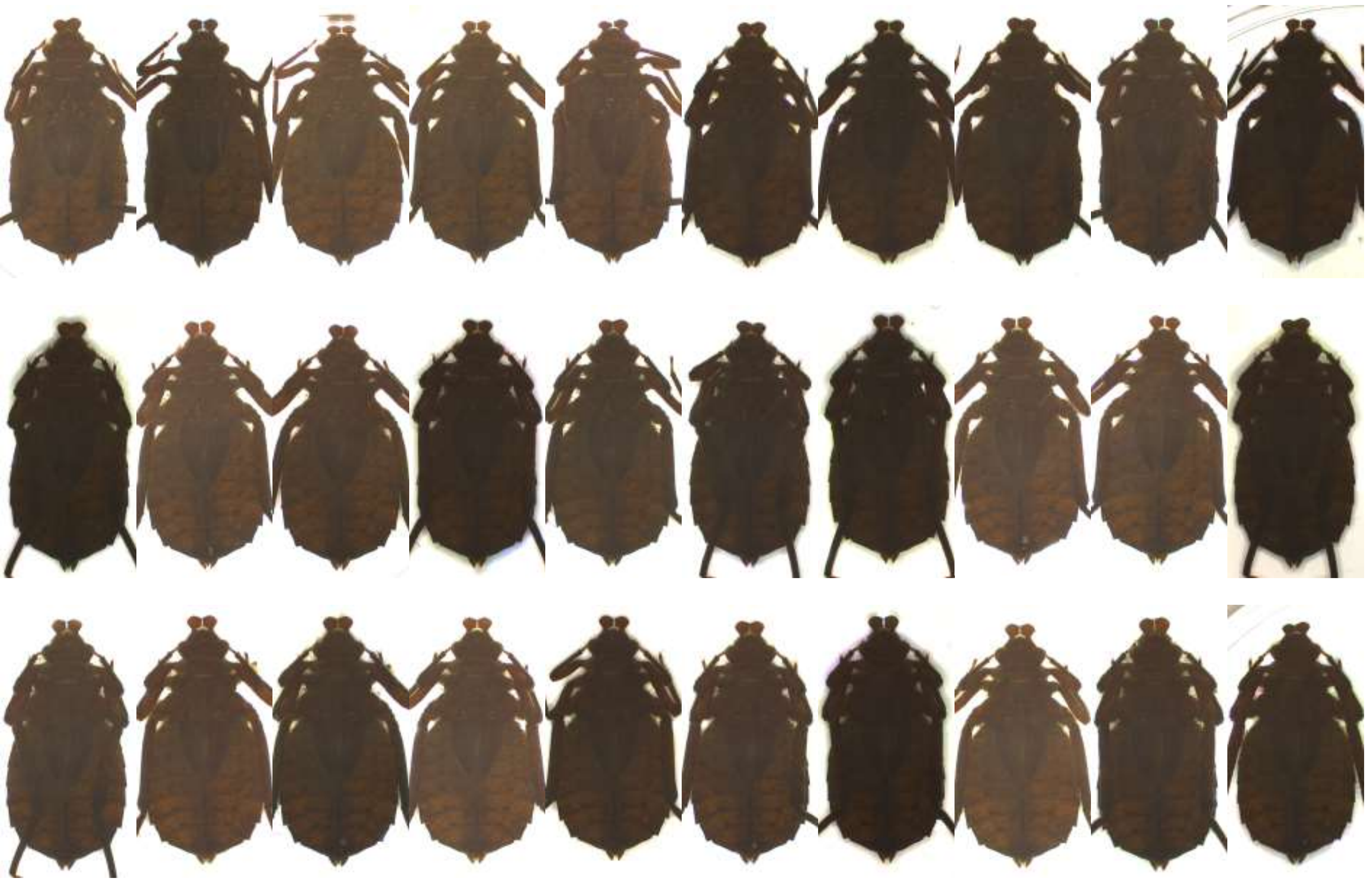

25-2 *Sieboldius albardae* (2/2)

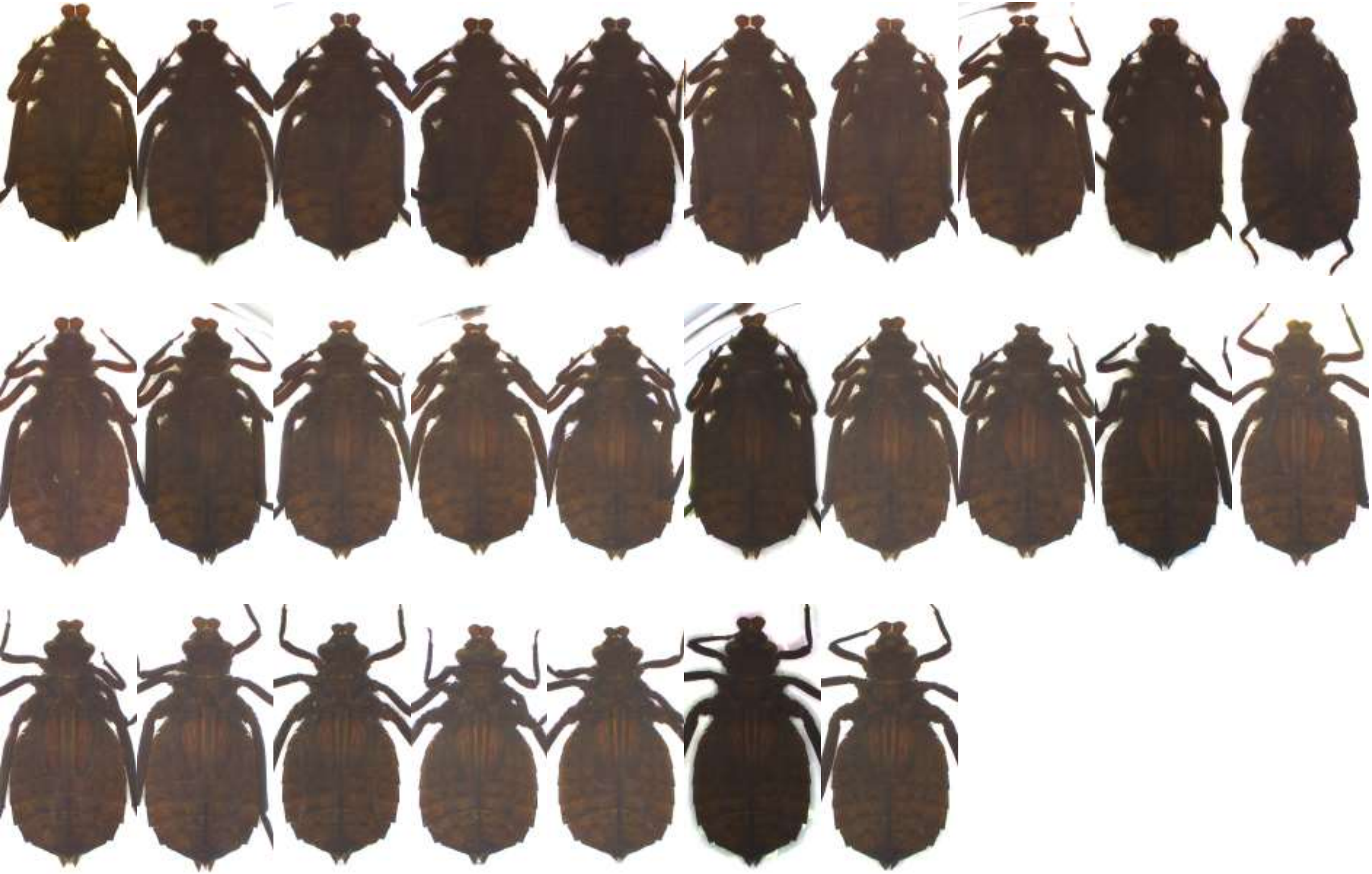

25-3 *Sieboldius albardae* (1/2)

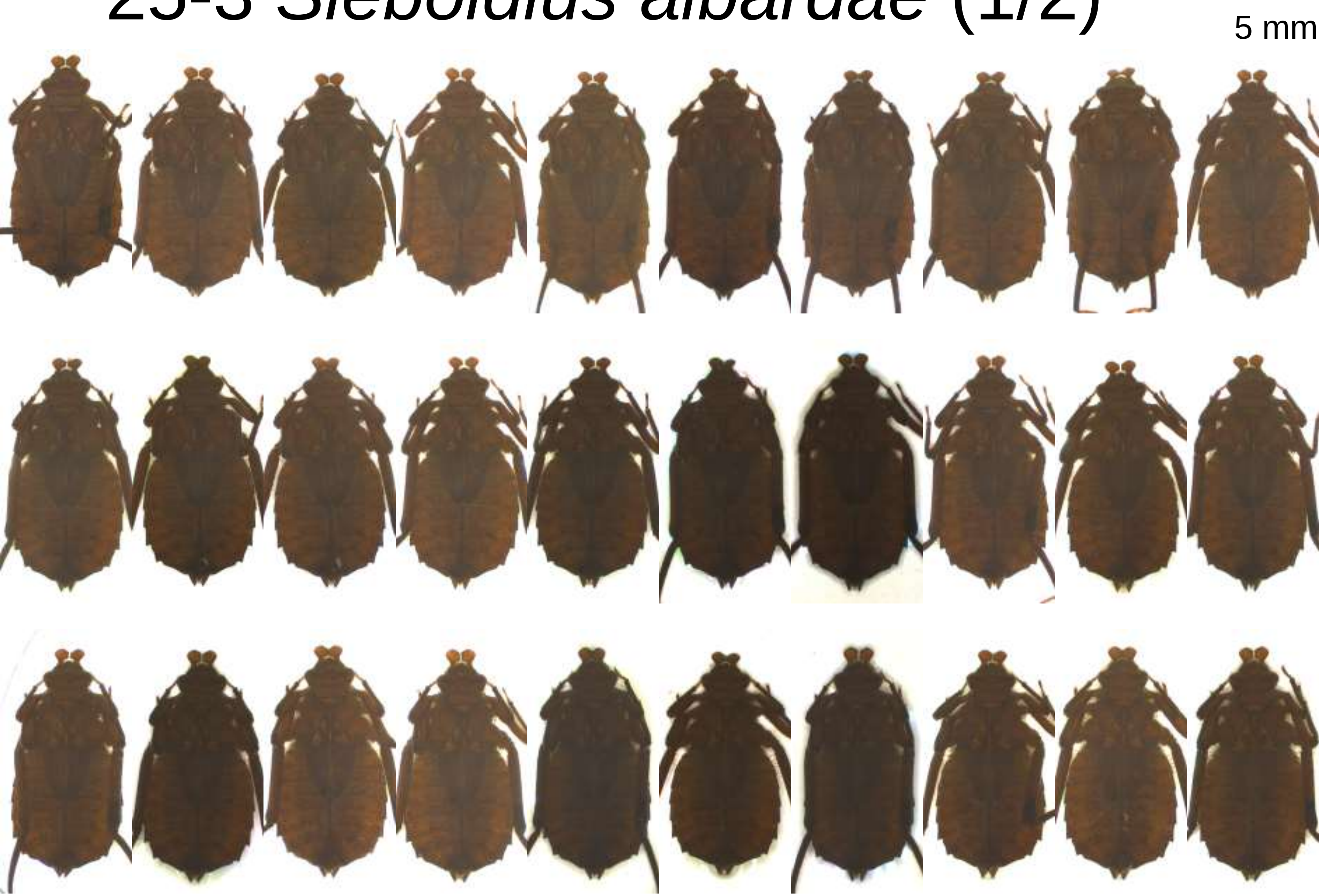

25-3 *Sieboldius albardae* (2/2)

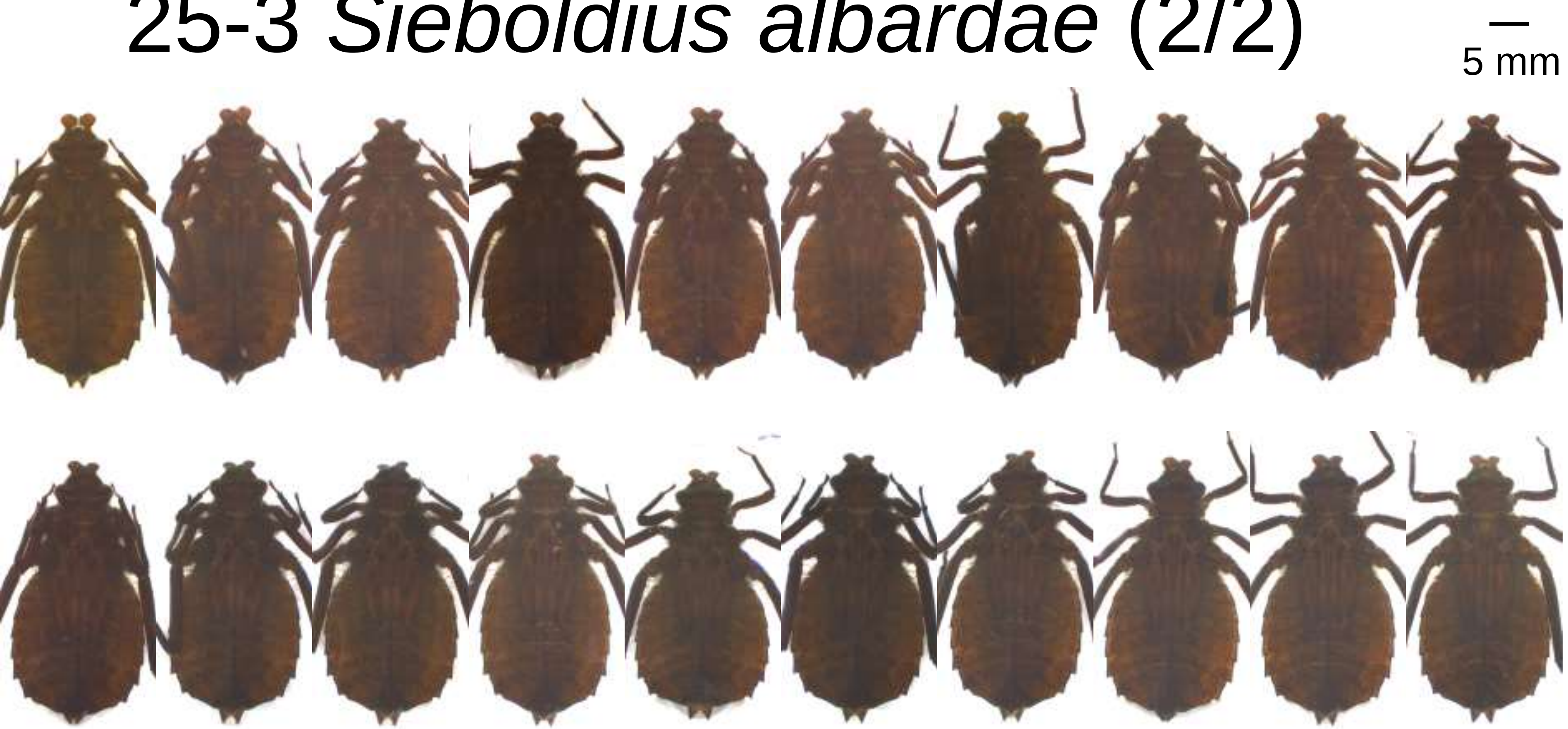

26-1 *Melligomphus viridicostus* (1/2)

5 mm

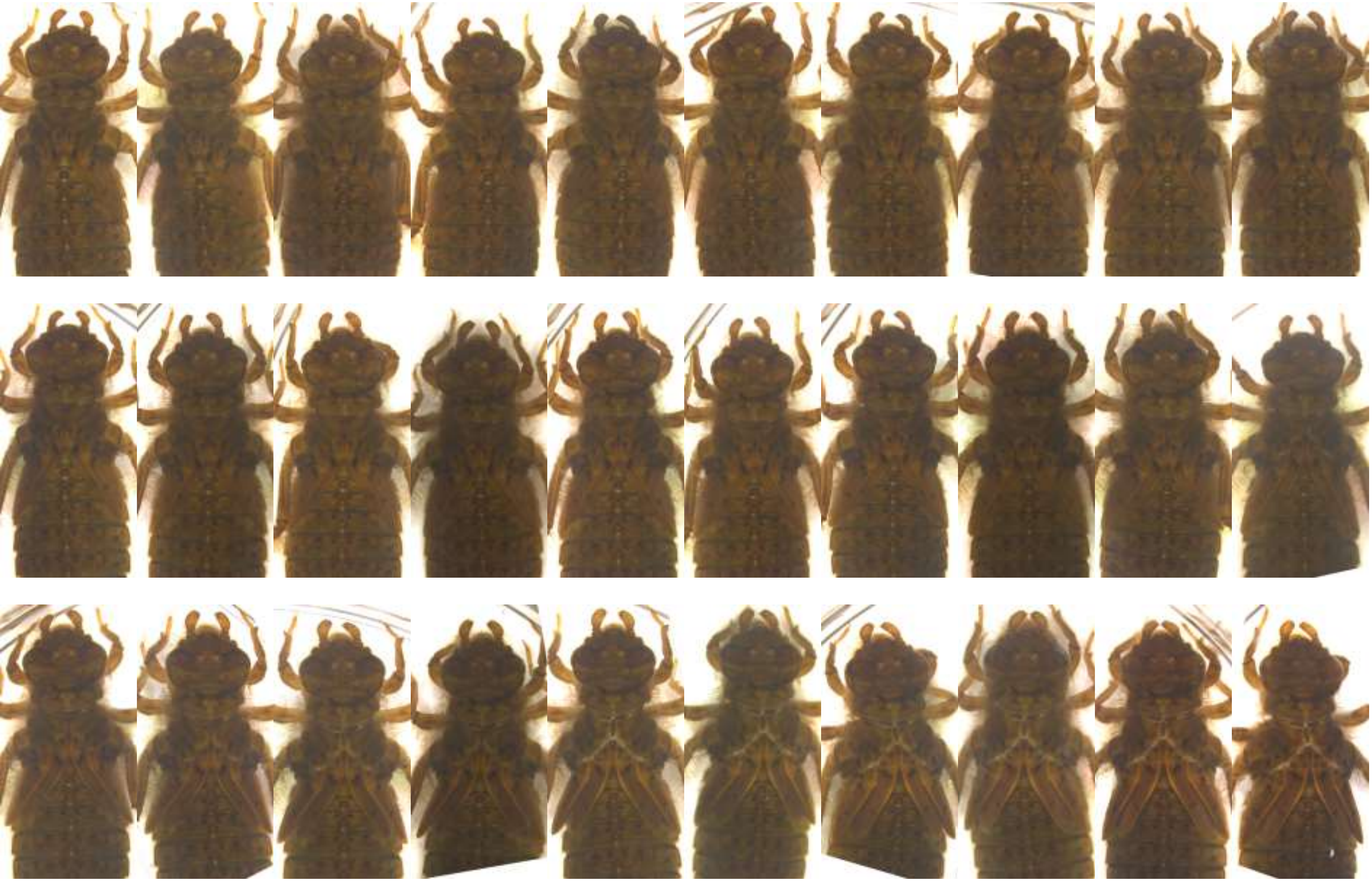

26-1 *Melligomphus viridicostus* (2/2)

5 mm

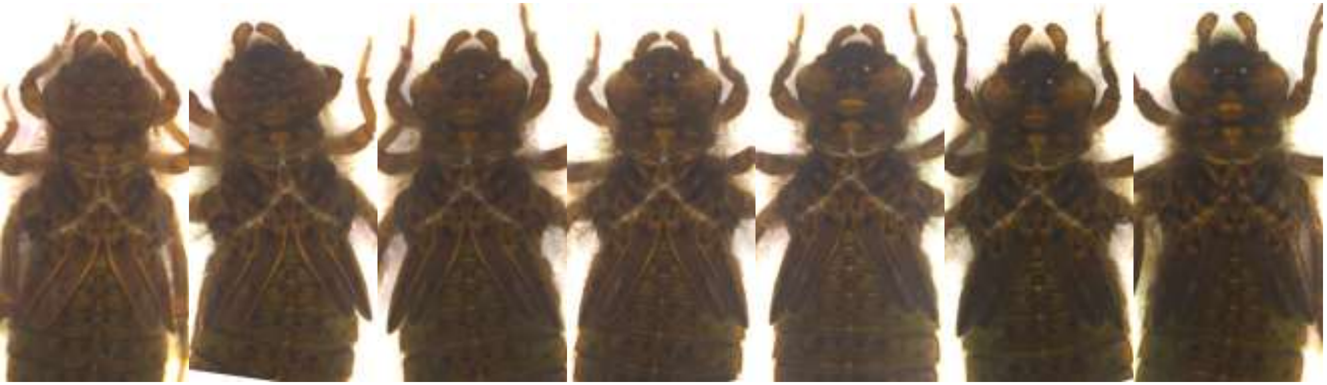

26-2 *Melligomphus viridicostus* (1/2)

5 mm

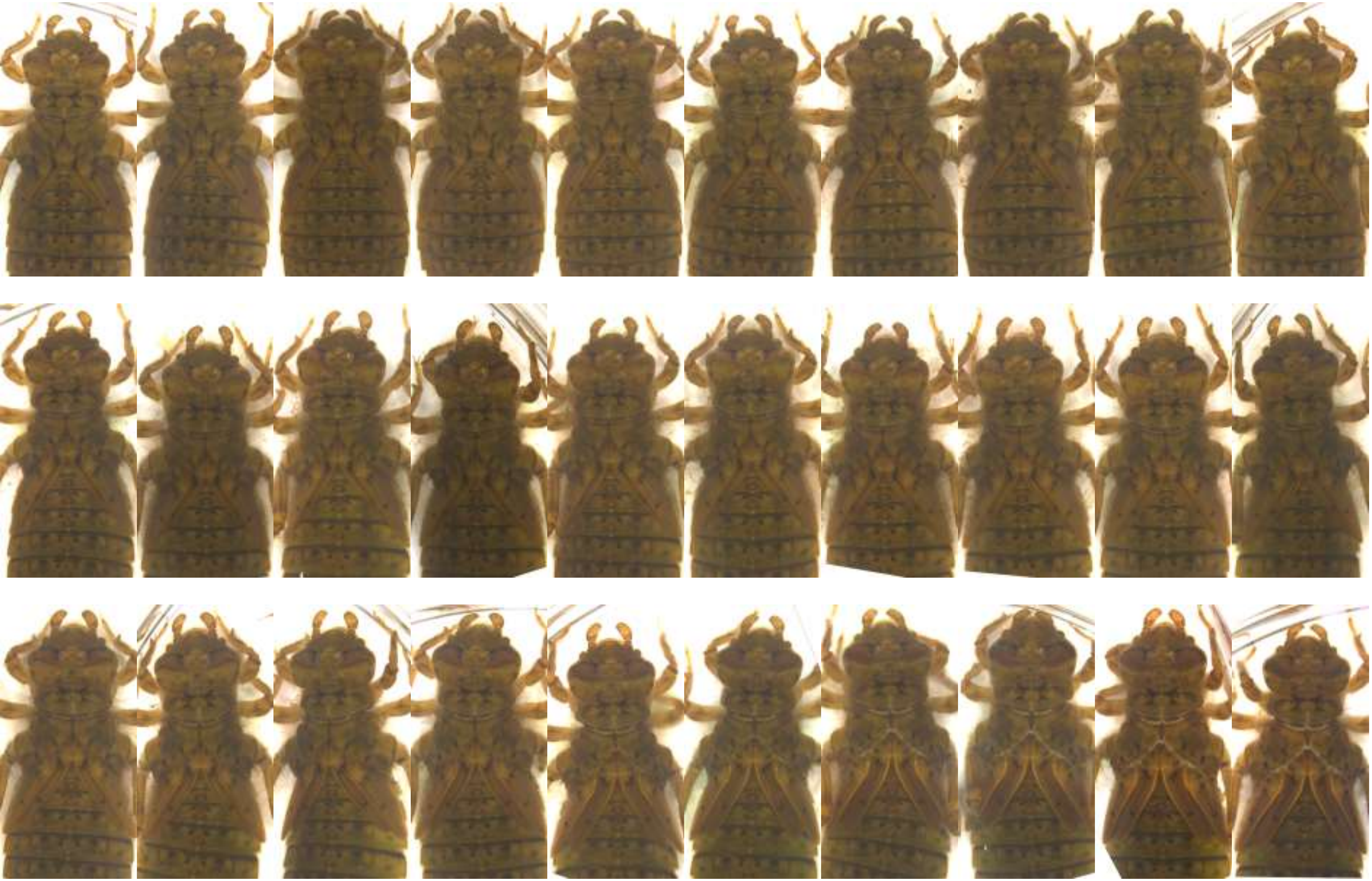

26-2 *Melligomphus viridicostus* (2/2)

5 mm

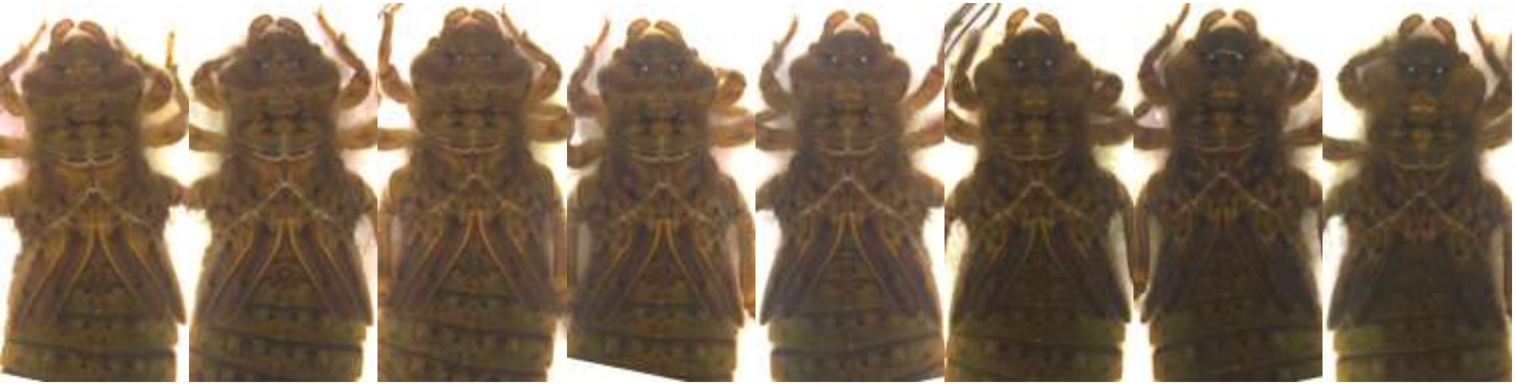

26-3 *Melligomphus viridicostus* (1/2)

5 mm

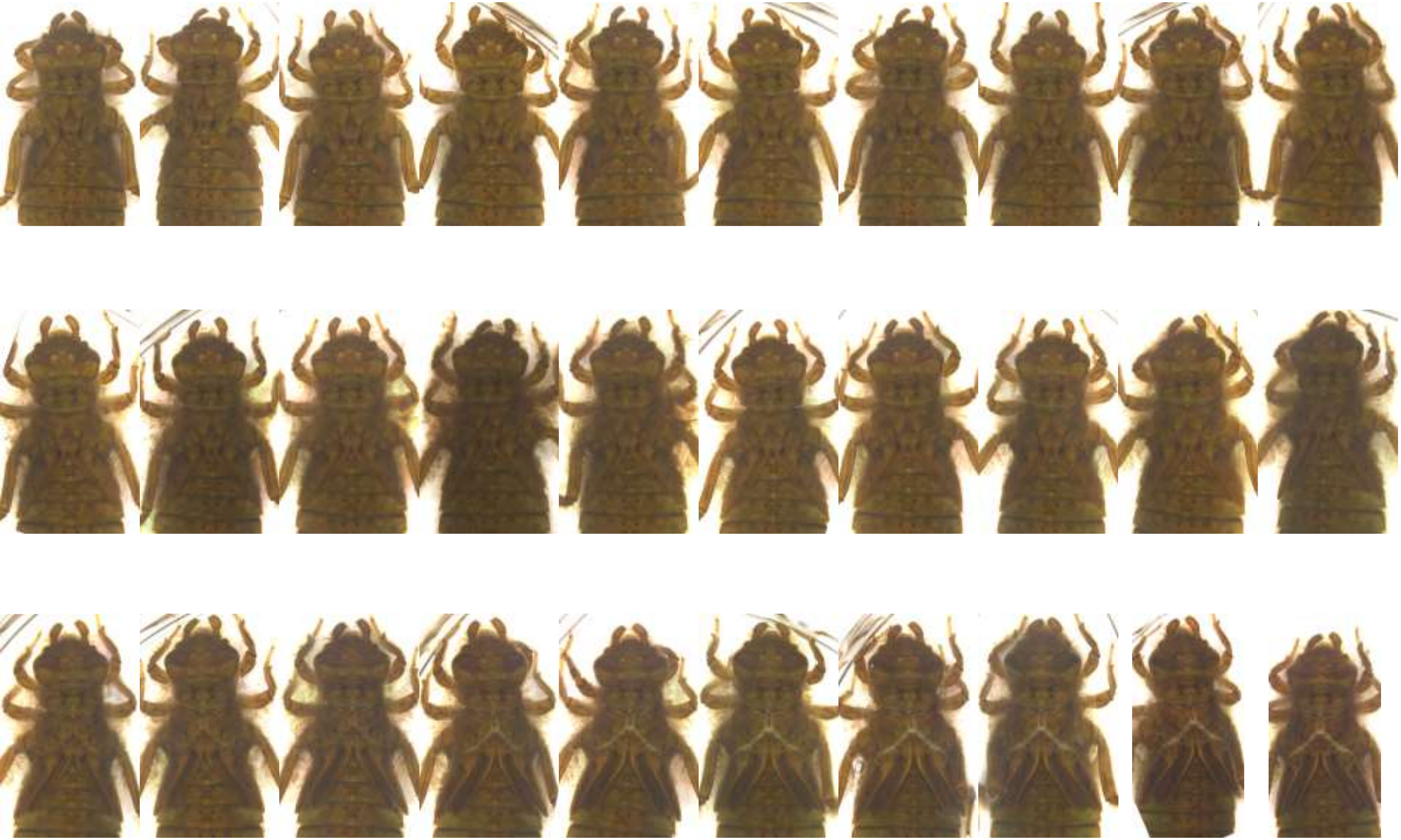

26-3 *Melligomphus viridicostus* (2/2)

5 mm

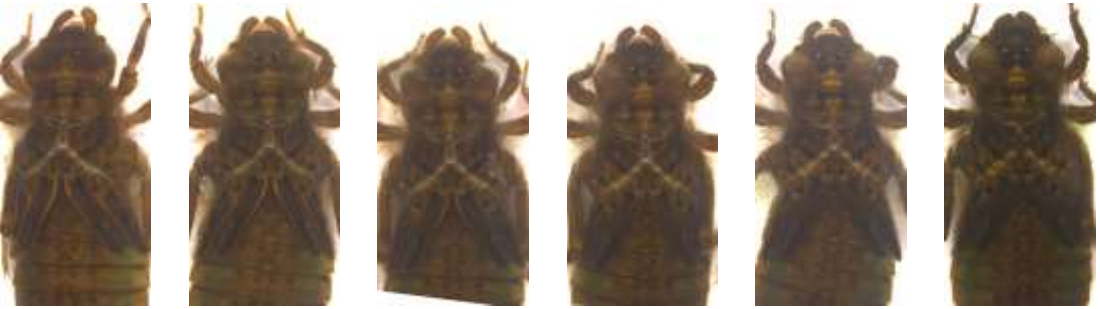

27-1 *Nihonogomphus viridis* (1/1)

5 mm

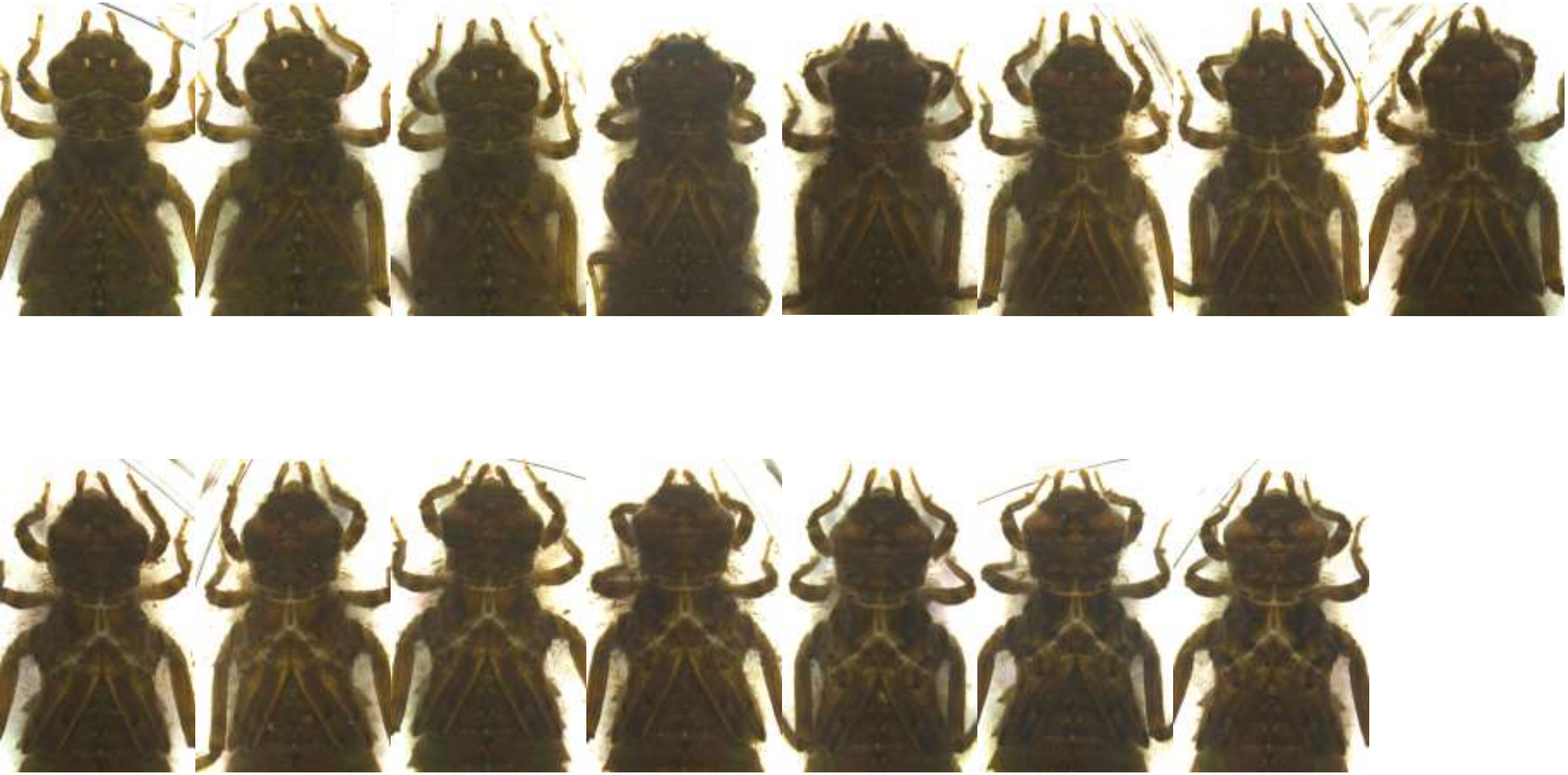

28-1 *Davidius nanus* (1/1)

5 mm

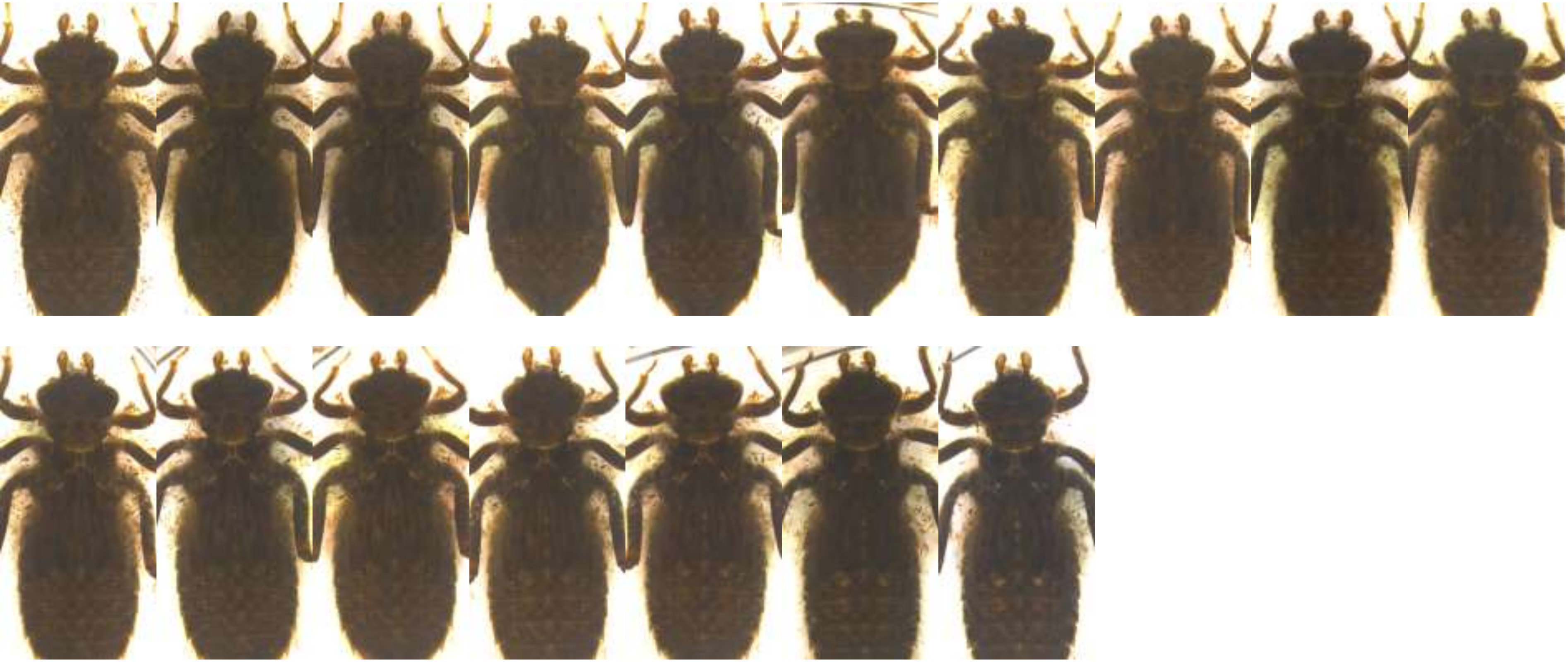

28-2 *Davidius nanus* (1/1)

5 mm

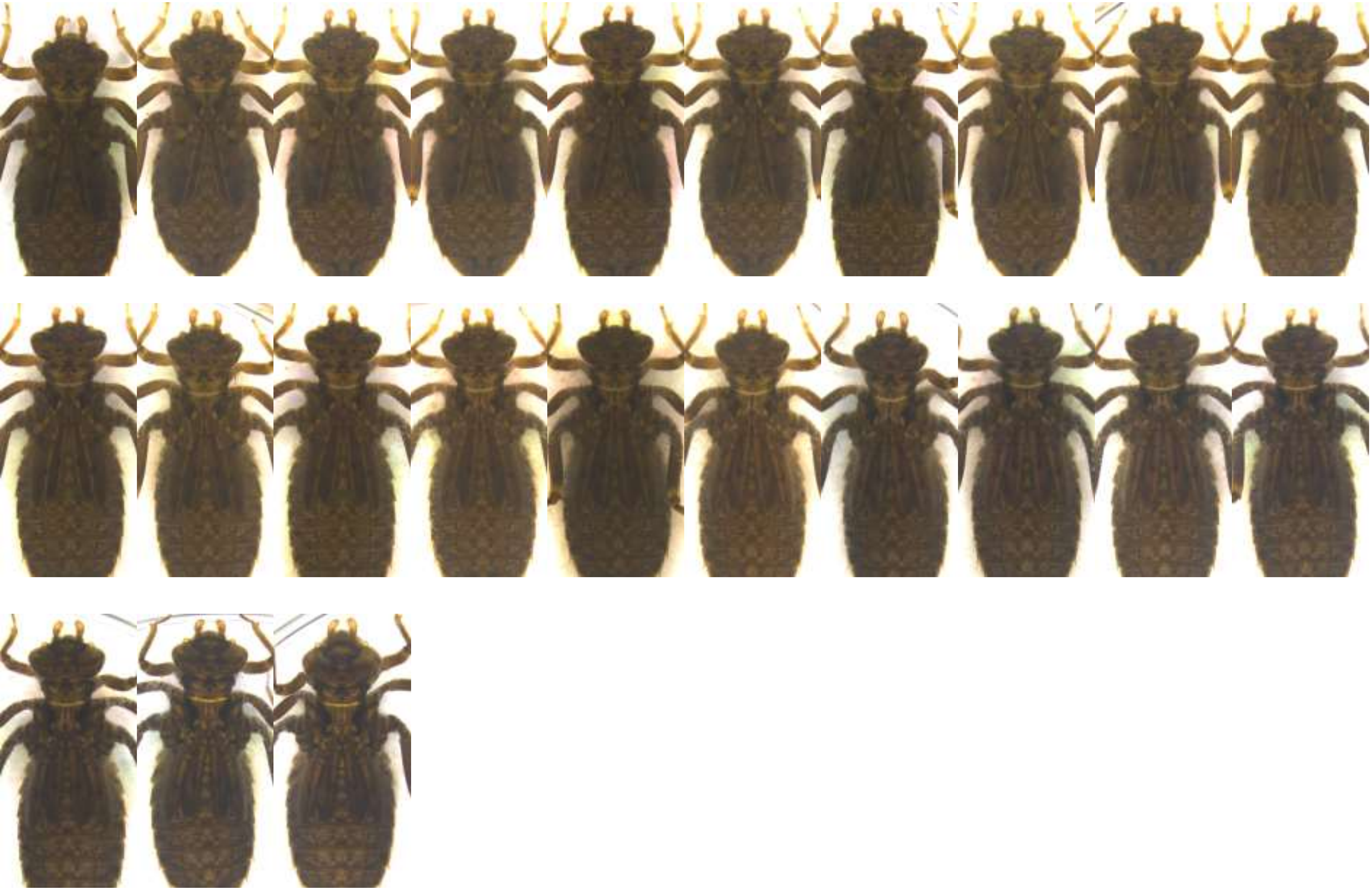

28-3 *Davidius nanus* (1/1)

5 mm

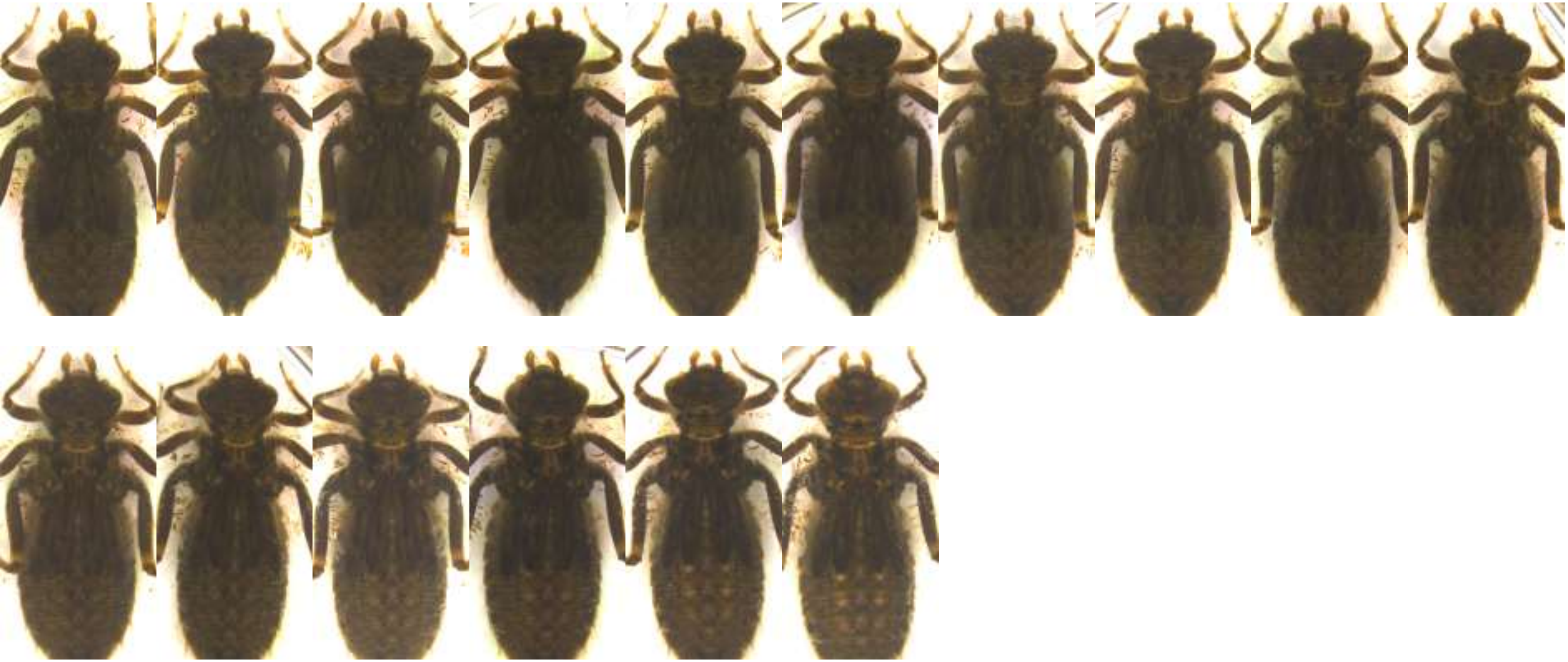

28-4 *Davidius nanus* (1/1)

5 mm

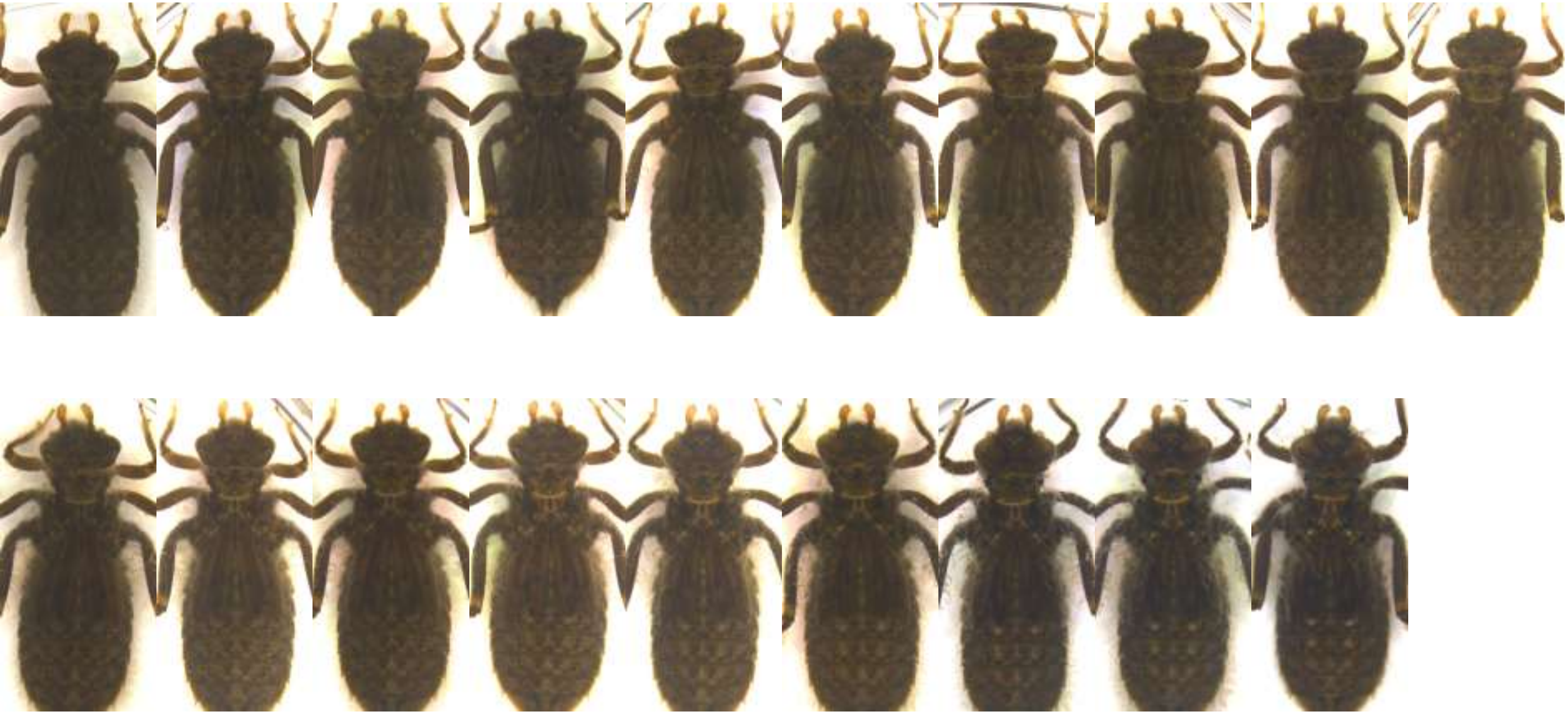

28-5 *Davidius nanus* (1/1)

5 mm

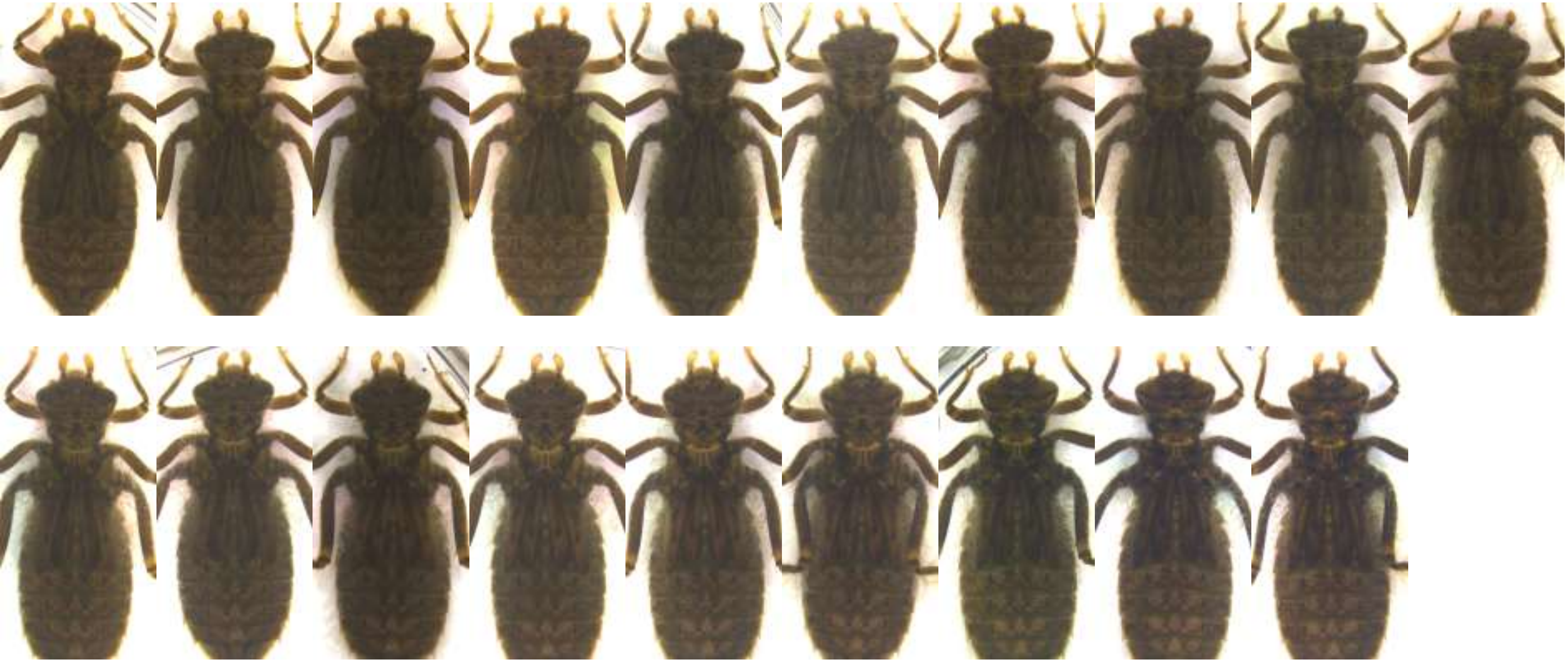

29-1 *Sinogomphus flavolimbatus* (1/2)

5 mm

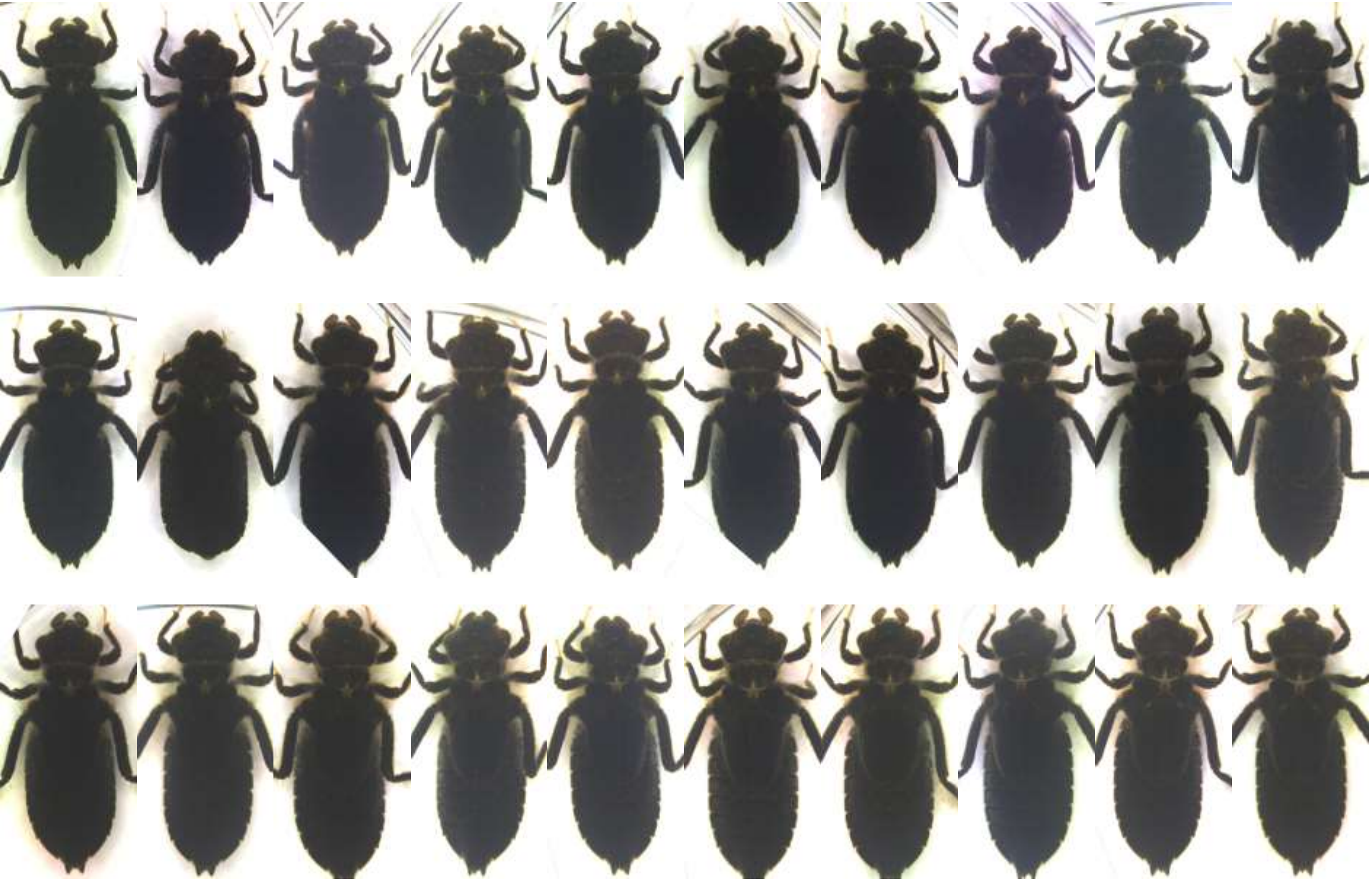

29-1 *Sinogomphus flavolimbatus* (2/2)

5 mm

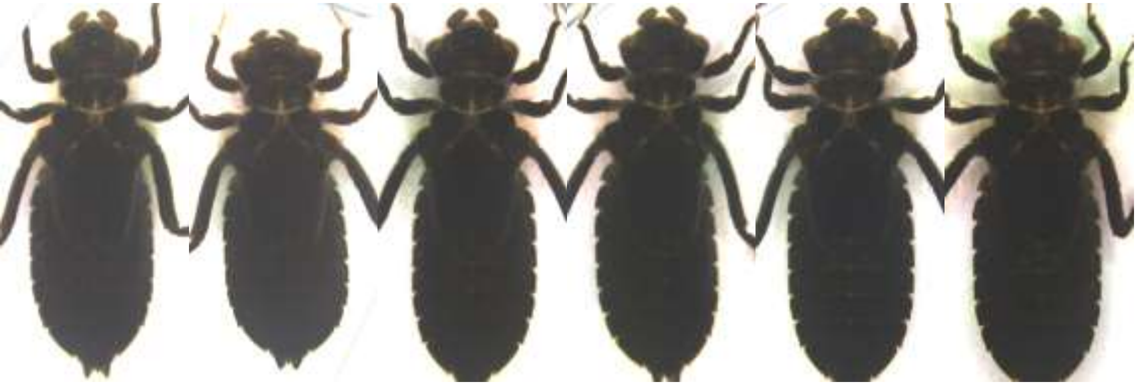

29-2 *Sinogomphus flavolimbatus* (1/2)

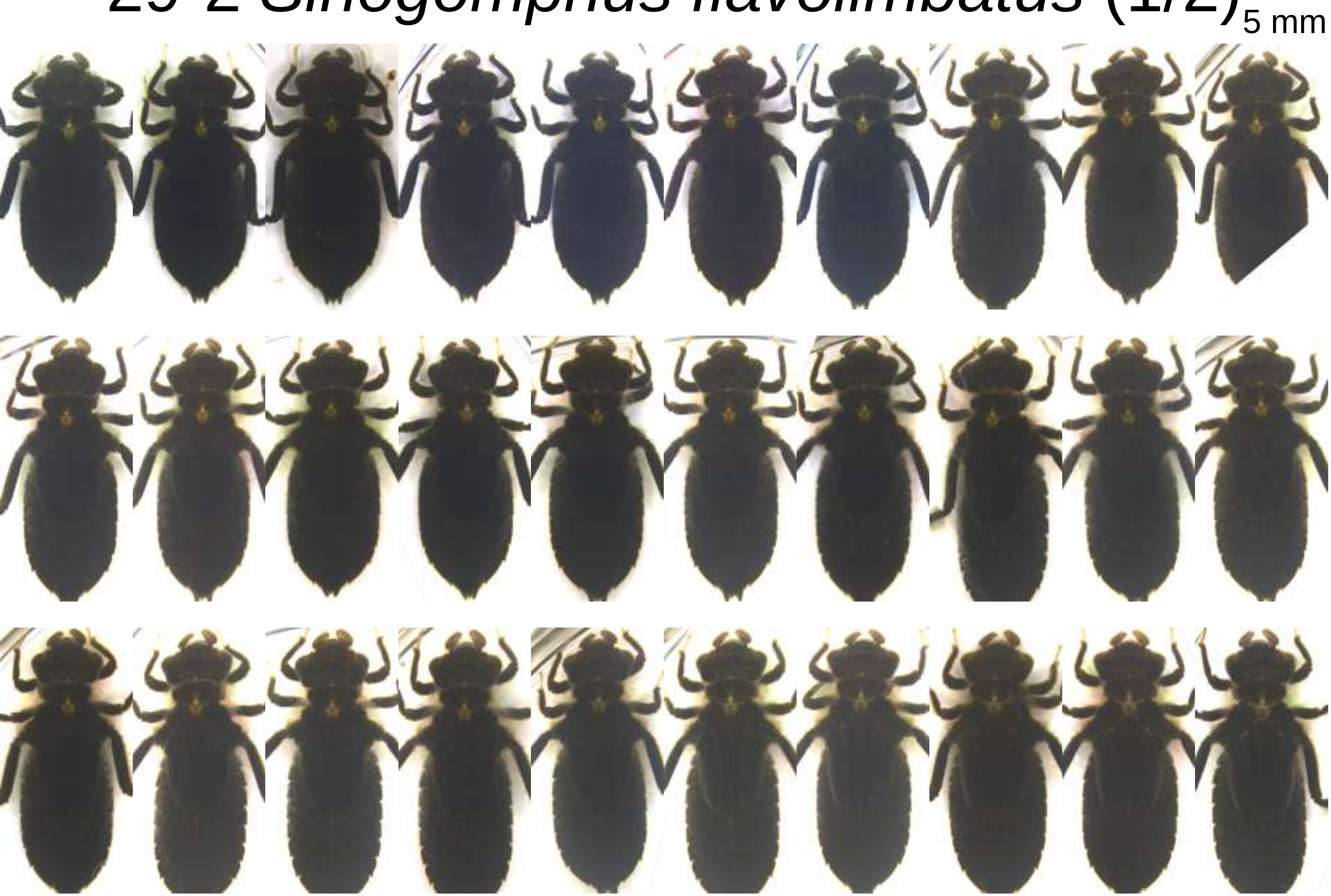

29-2 *Sinogomphus flavolimbatus* (2/2)

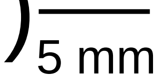

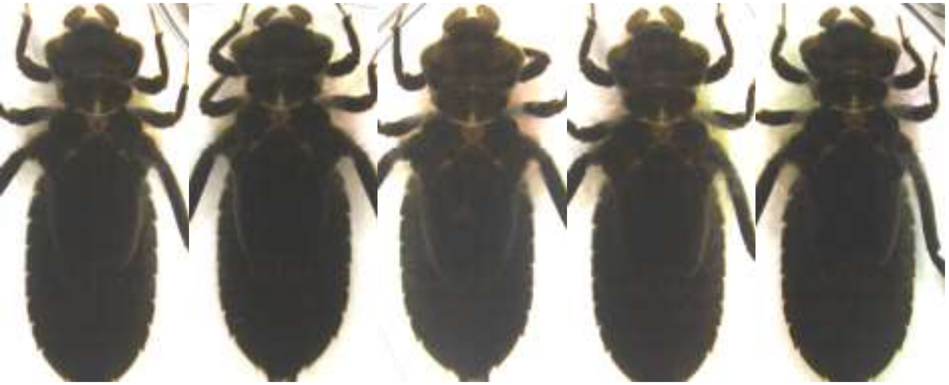

30-1 *Stylogomphus suzukii* (1/2)

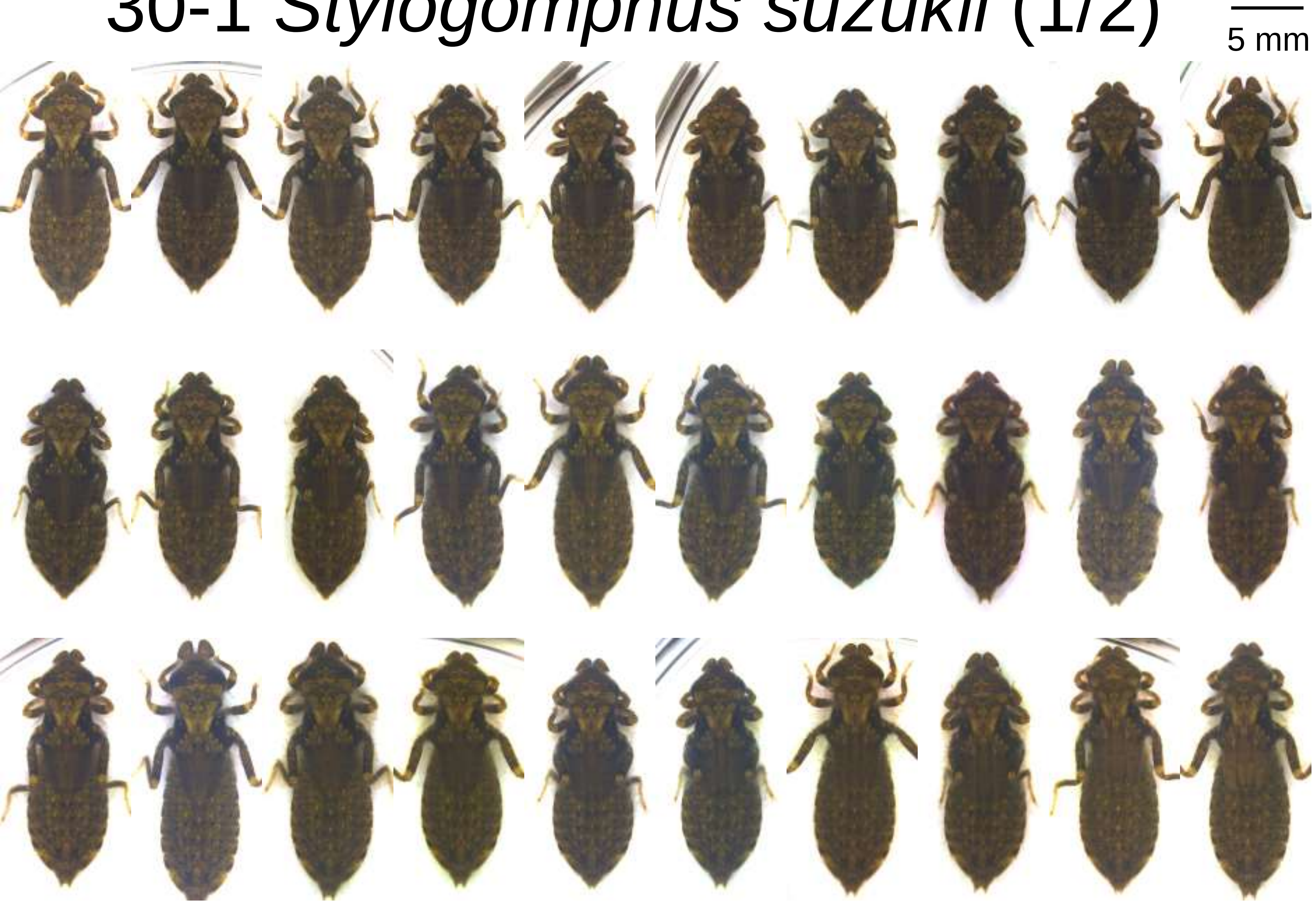

30-1 *Stylogomphus suzukii* (2/2)

5 mm

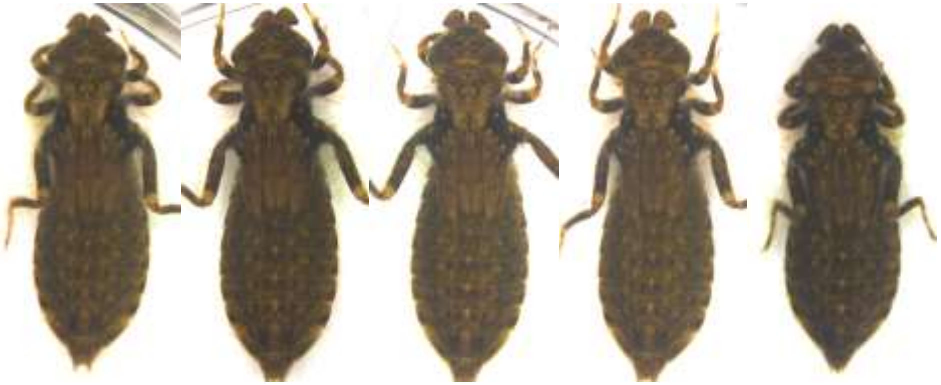

30-2 *Stylogomphus suzukii* (1/2)

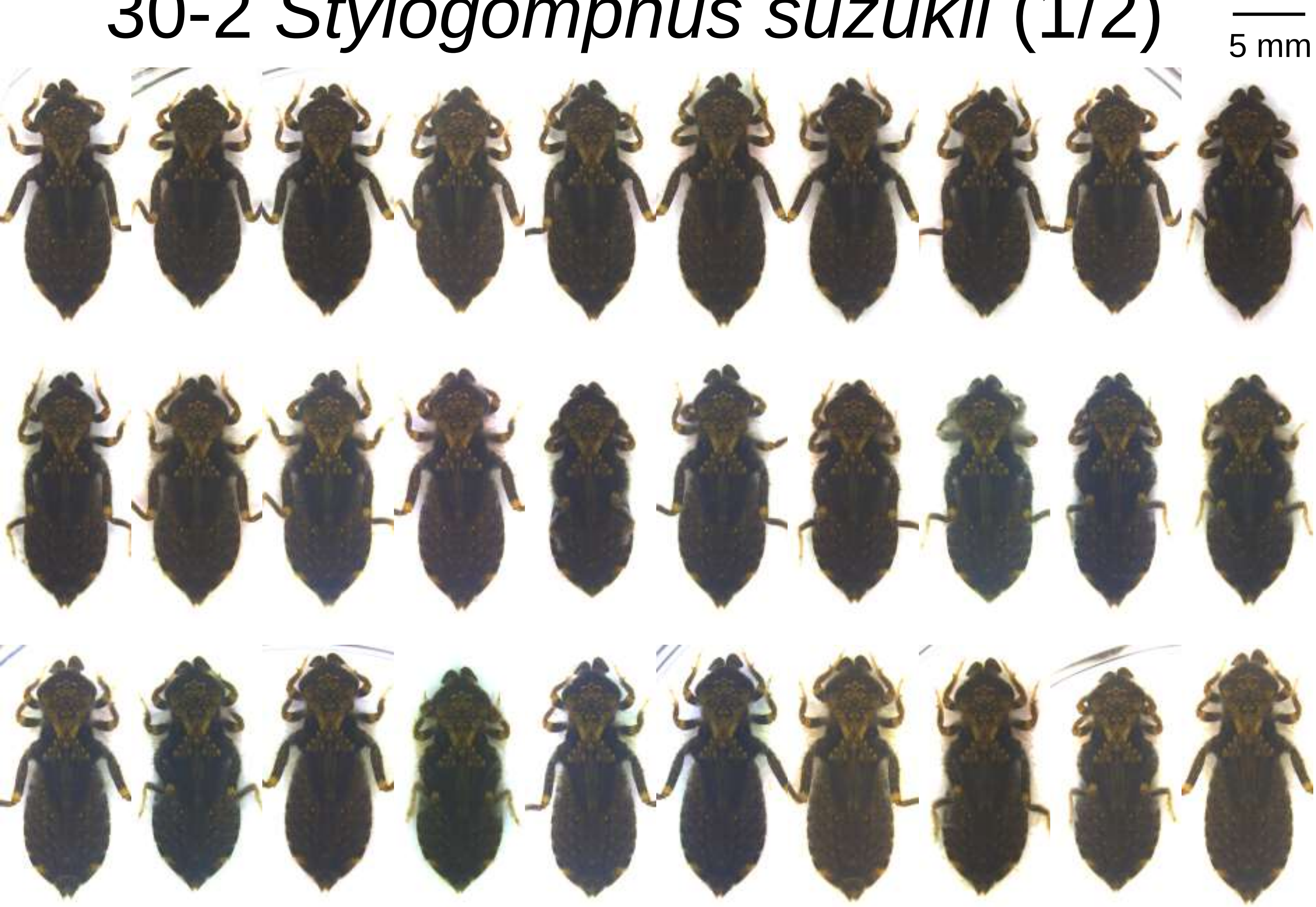

30-2 *Stylogomphus suzukii* (2/2)

5 mm

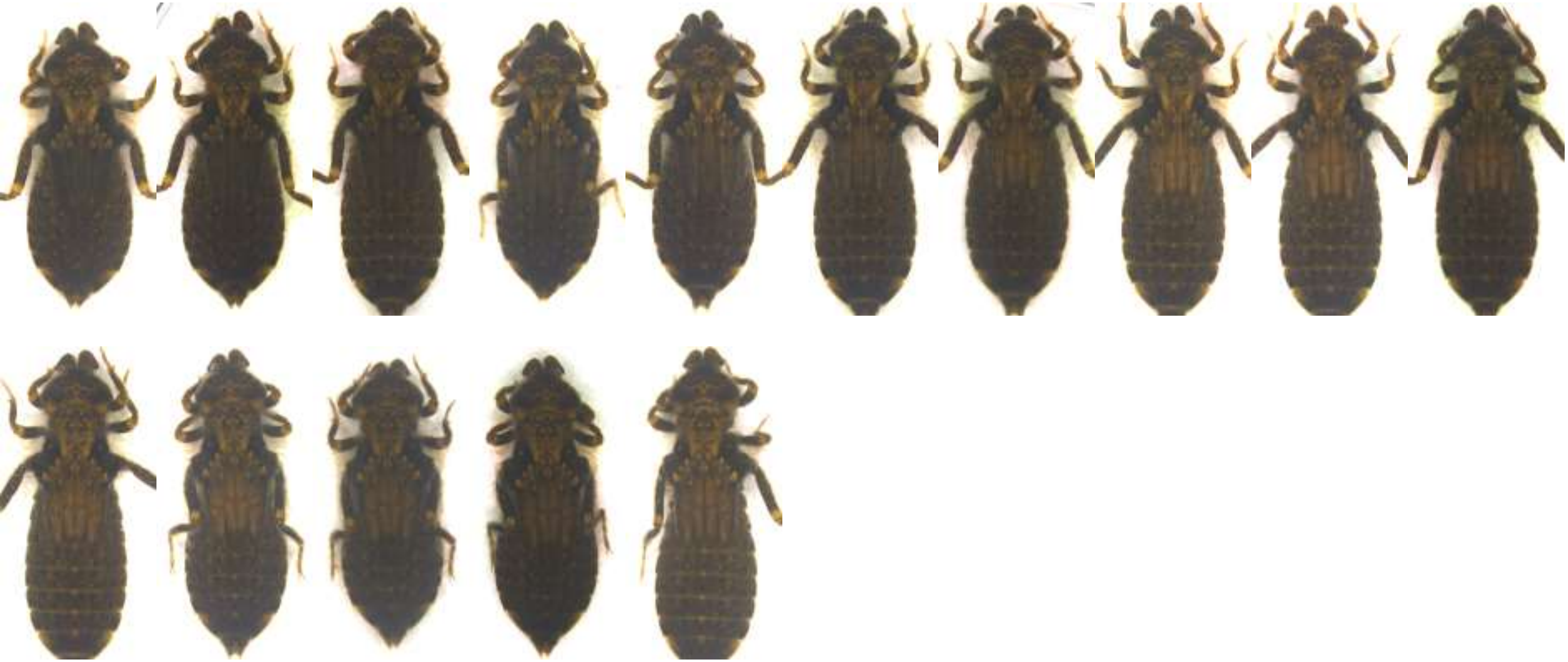

30-3 *Stylogomphus suzukii* (1/2)

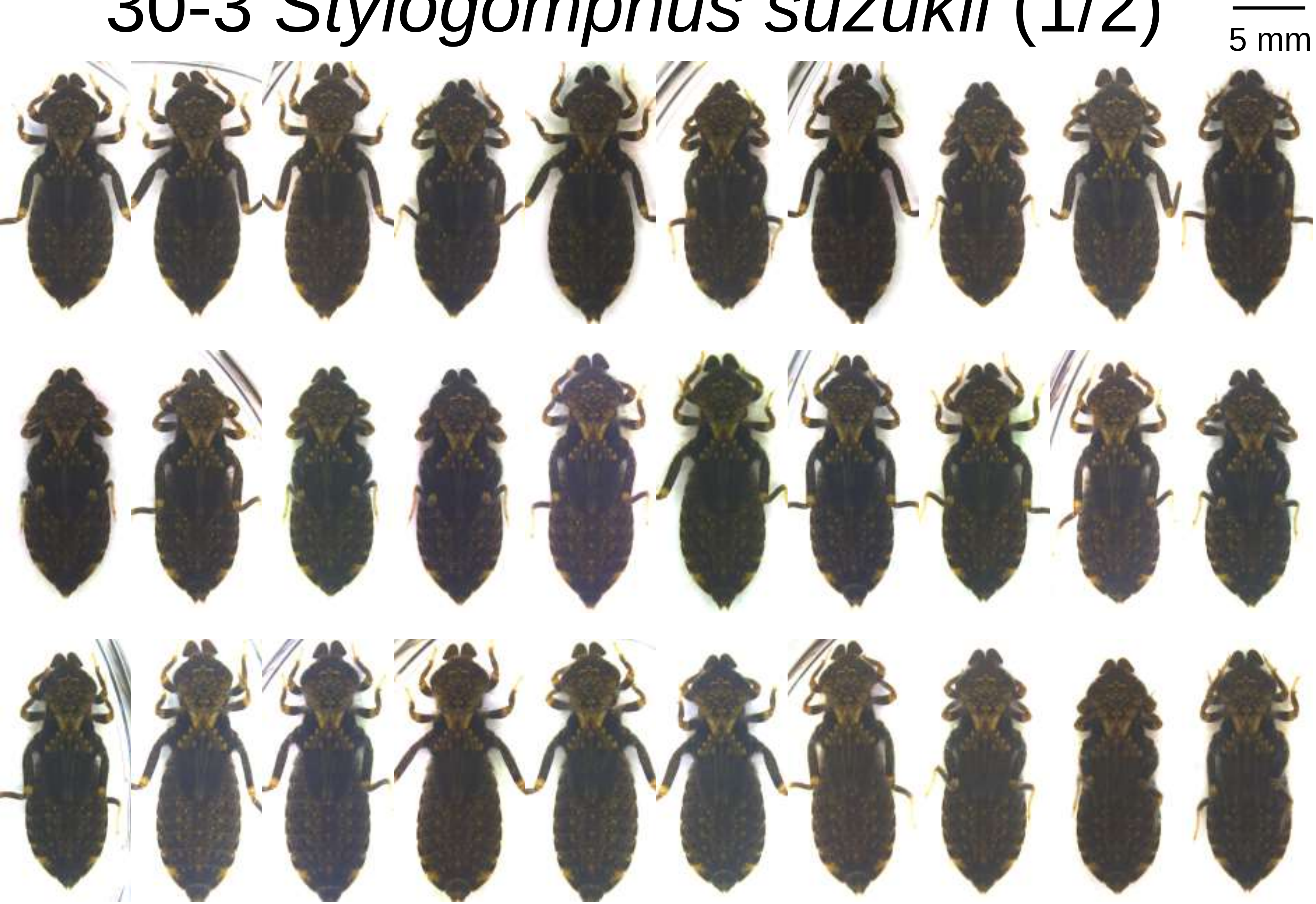

30-3 *Stylogomphus suzukii* (2/2)

5 mm

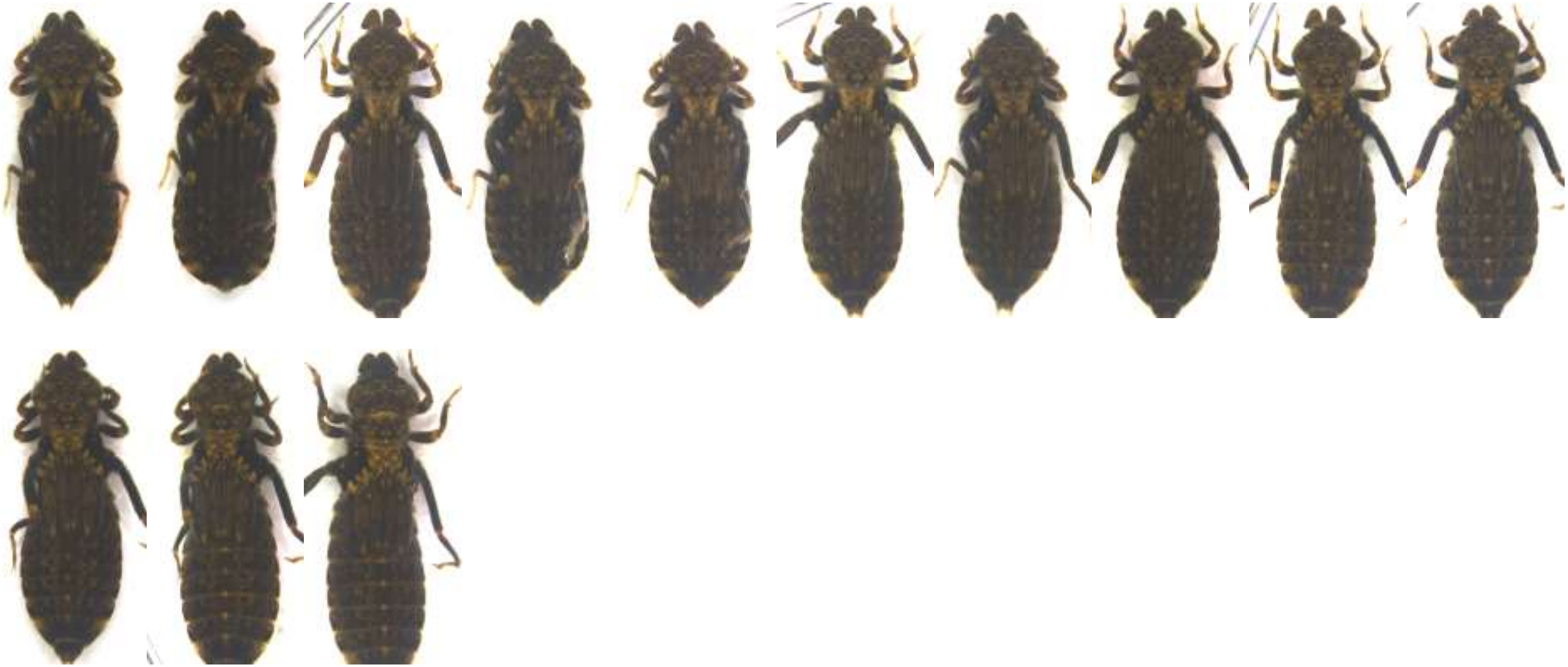

30-4 *Stylogomphus suzukii* (1/2)

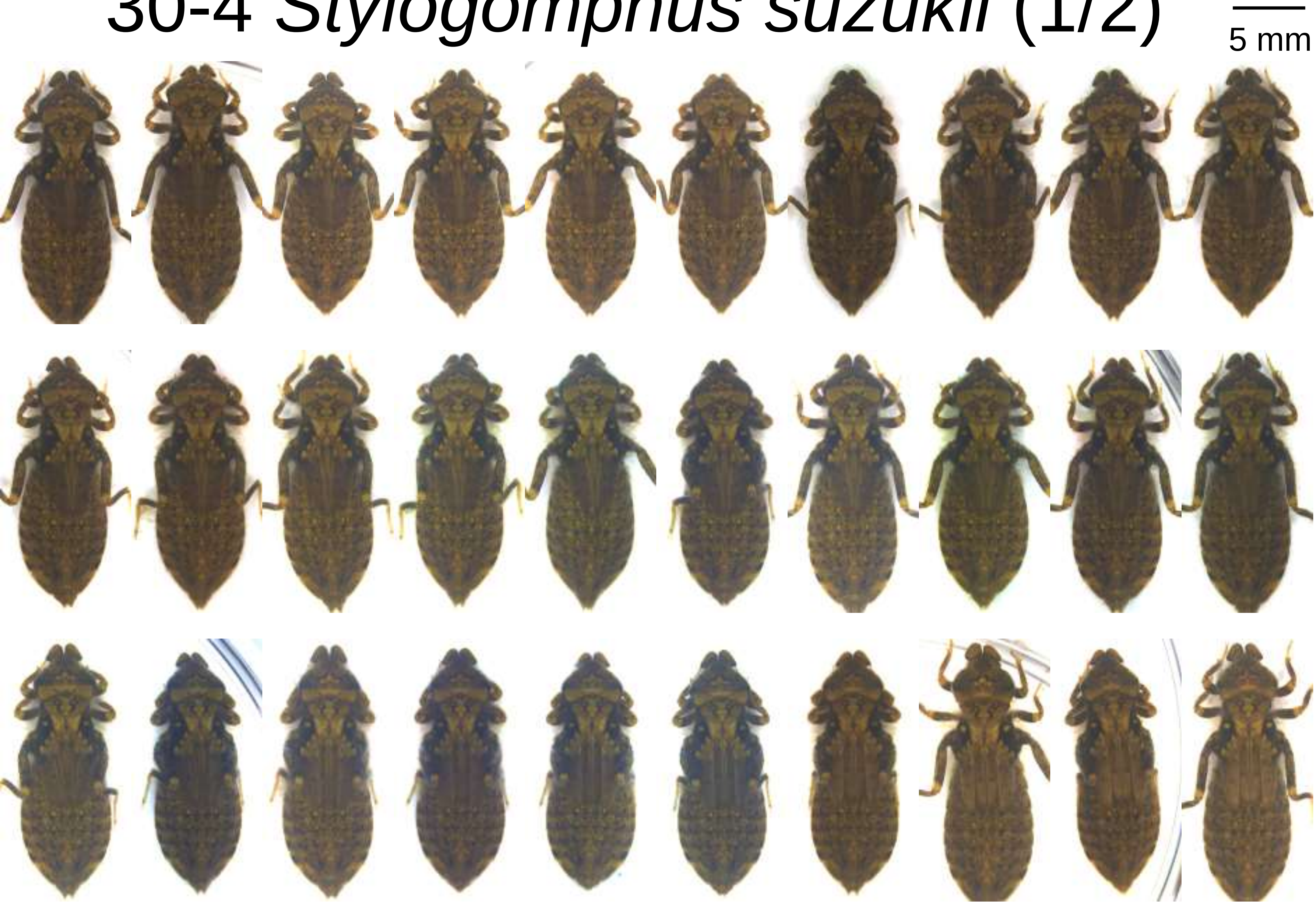

30-4 *Stylogomphus suzukii* (2/2)

5 mm

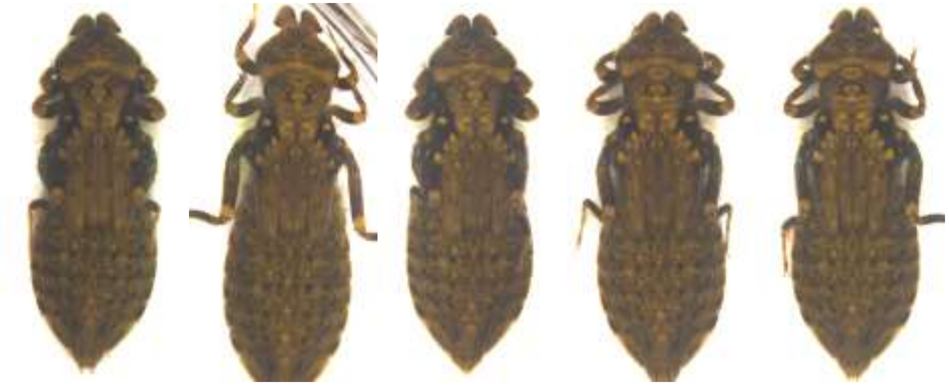

31-1 *Asiagomphus melaenops* (1/1)

5 mm

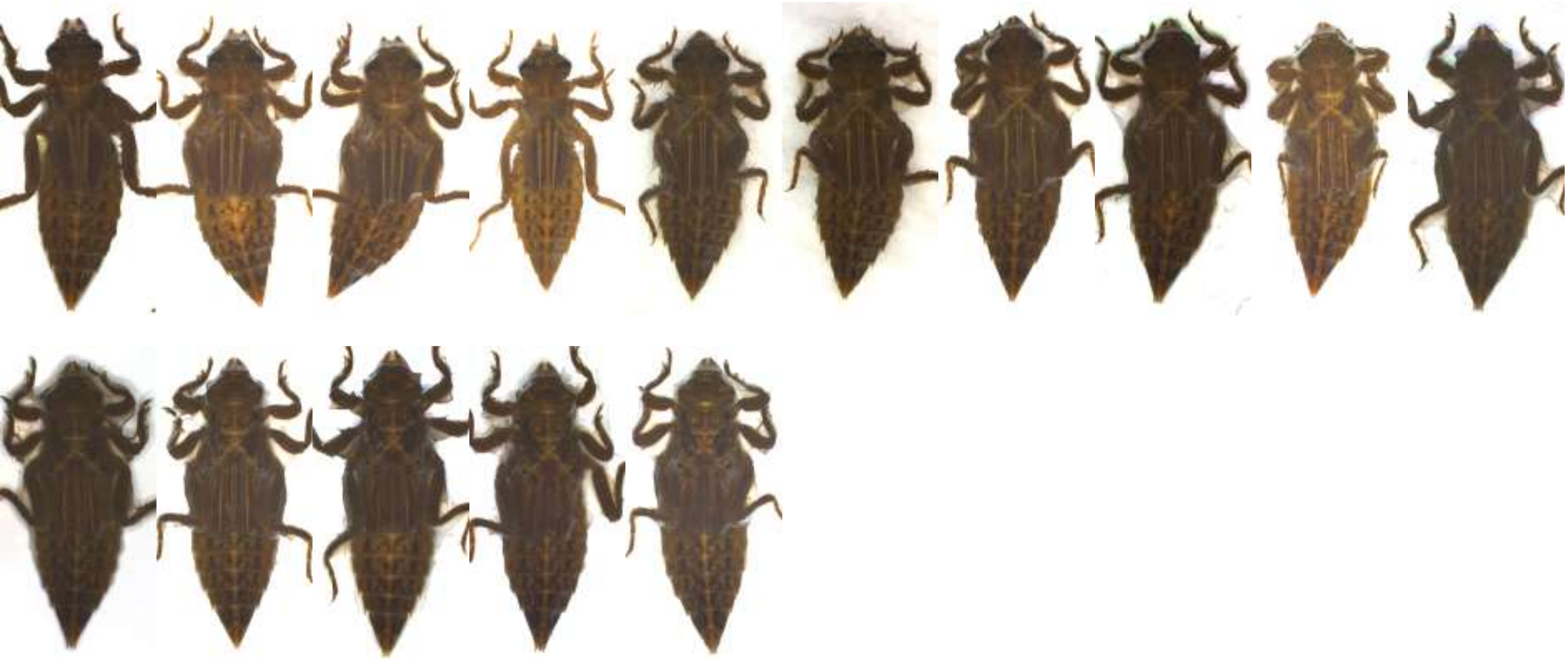

31-2 *Asiagomphus melaenops* (1/1)

5 mm

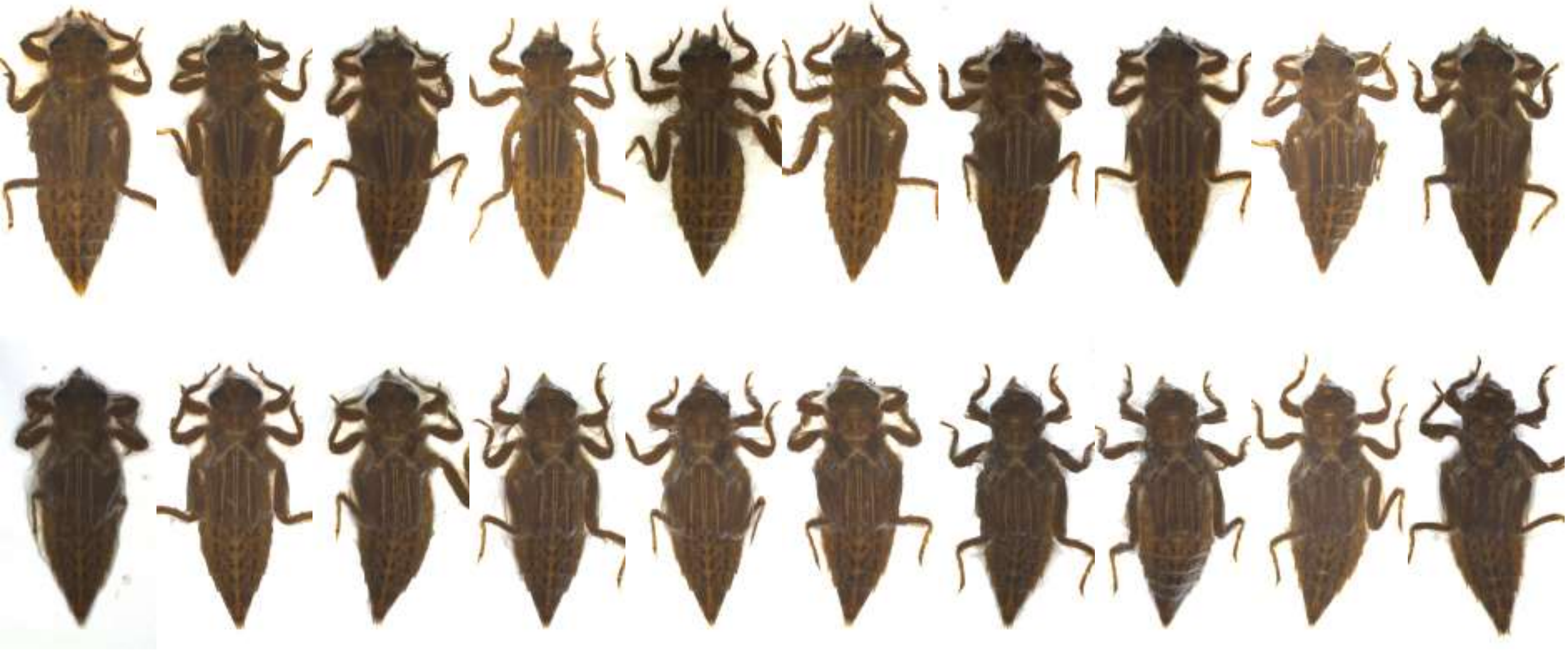

31-3 *Asiagomphus melaenops* (1/1)

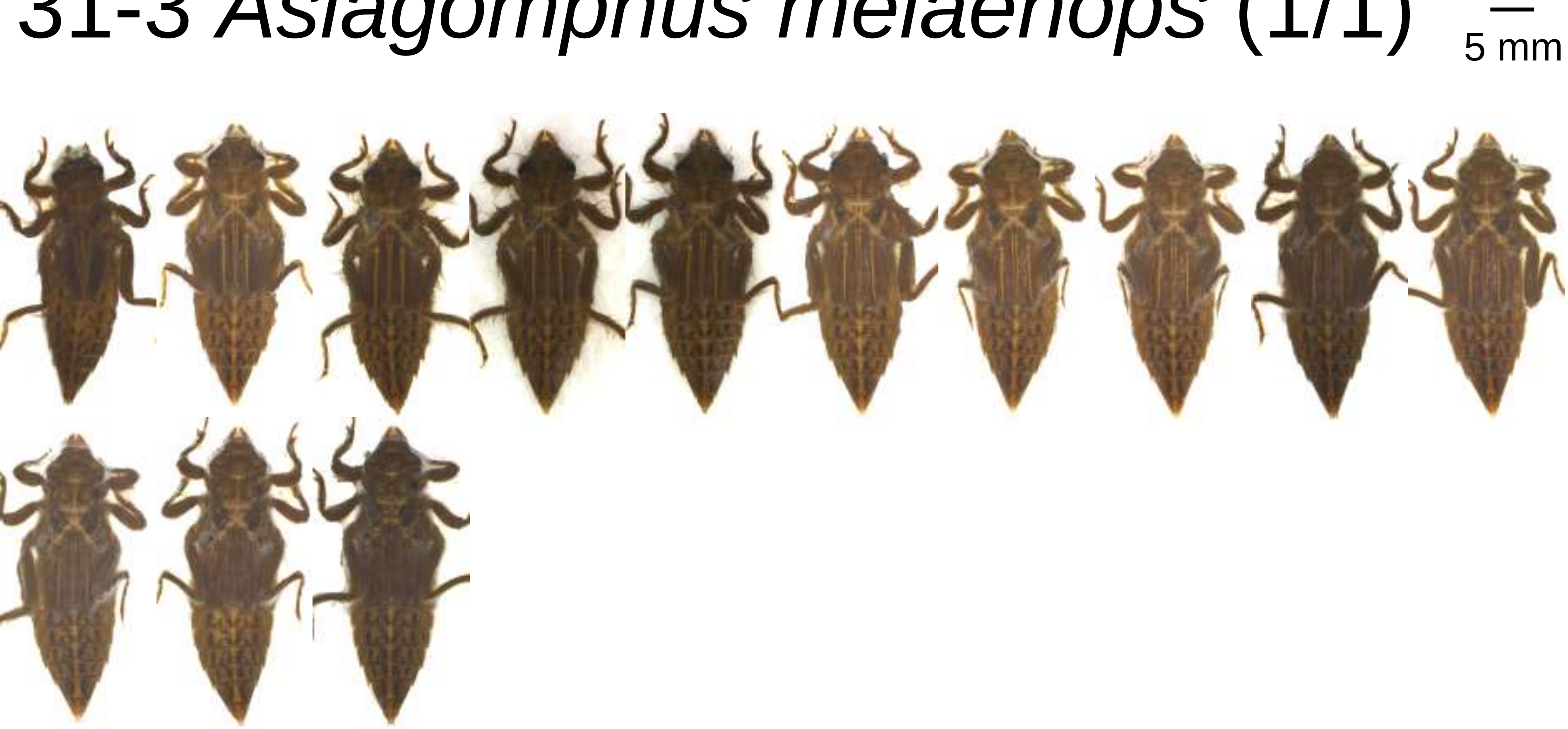

31-4 *Asiagomphus melaenops* (1/1)

5 mm

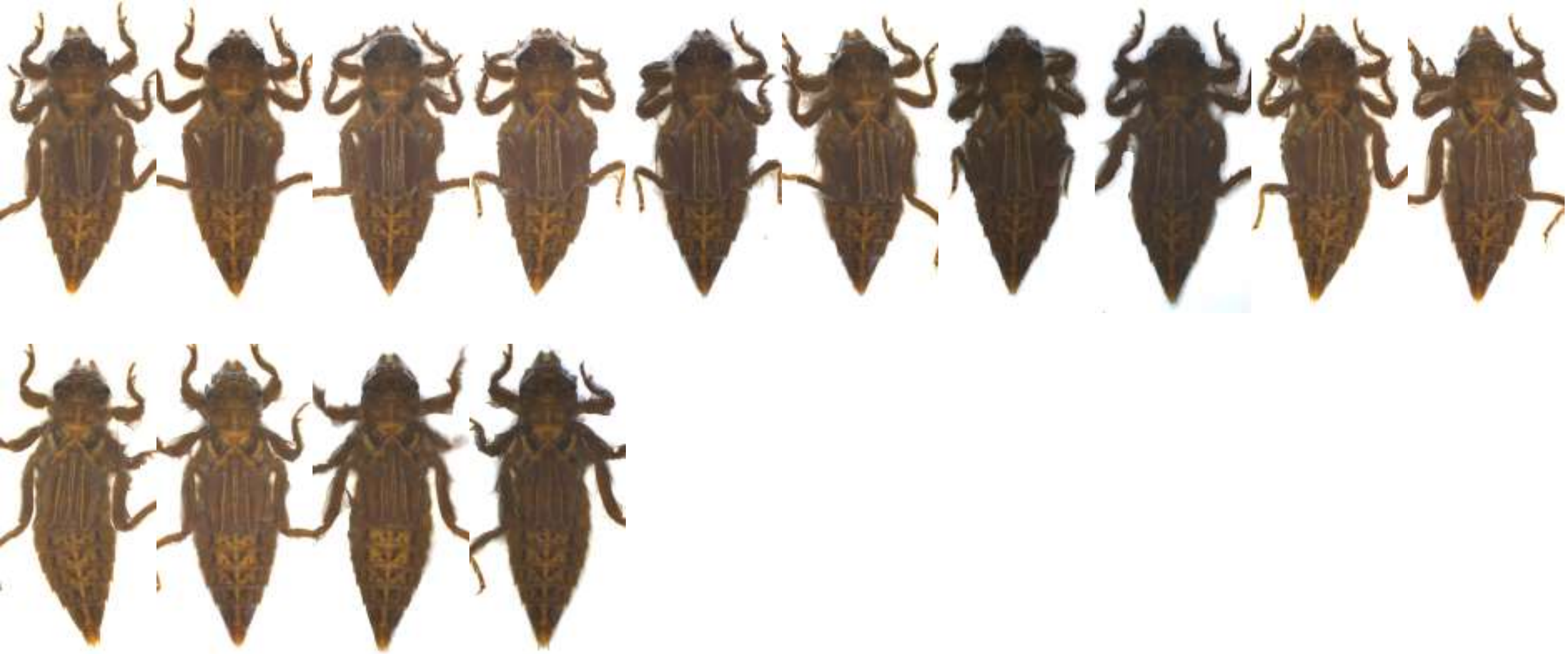

32-1 *Tanypteryx pryeri* (1/1)

5 mm

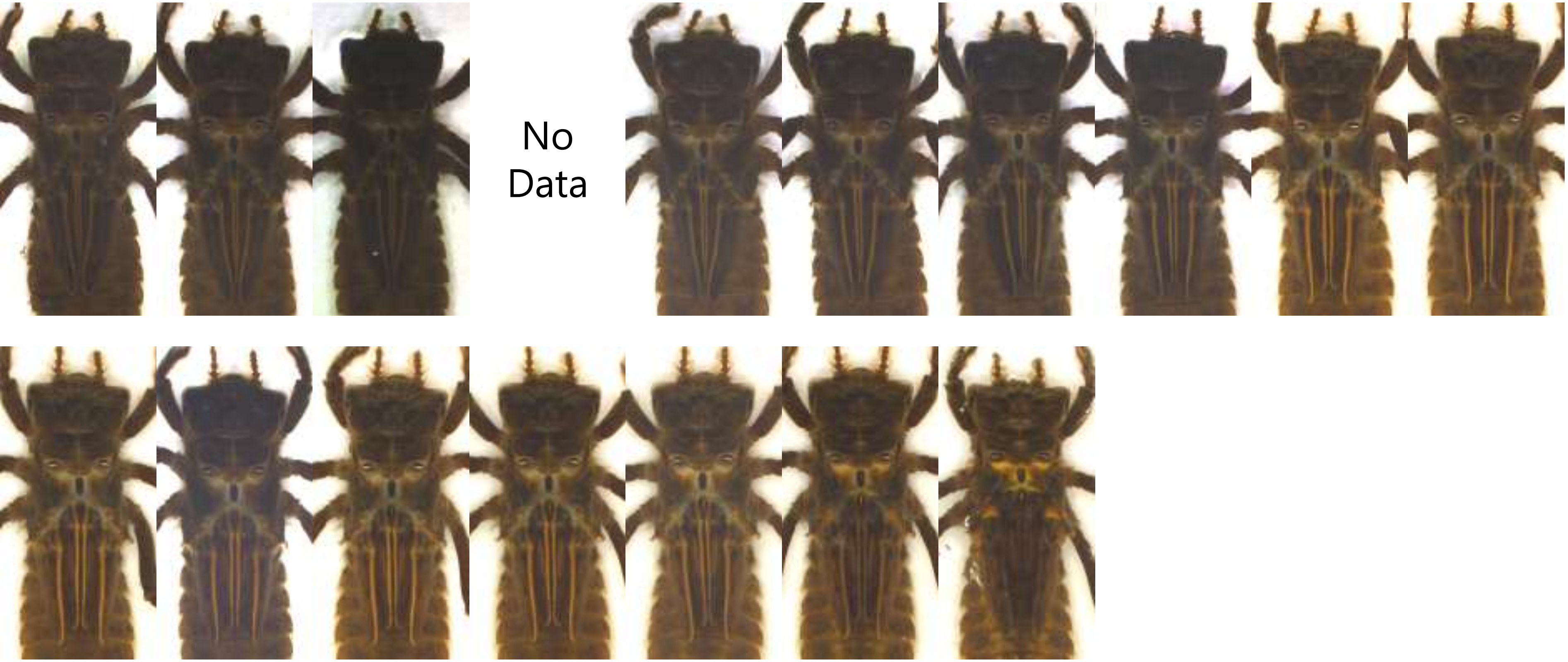

32-2 *Tanypteryx pryeri* (1/1)

5 mm

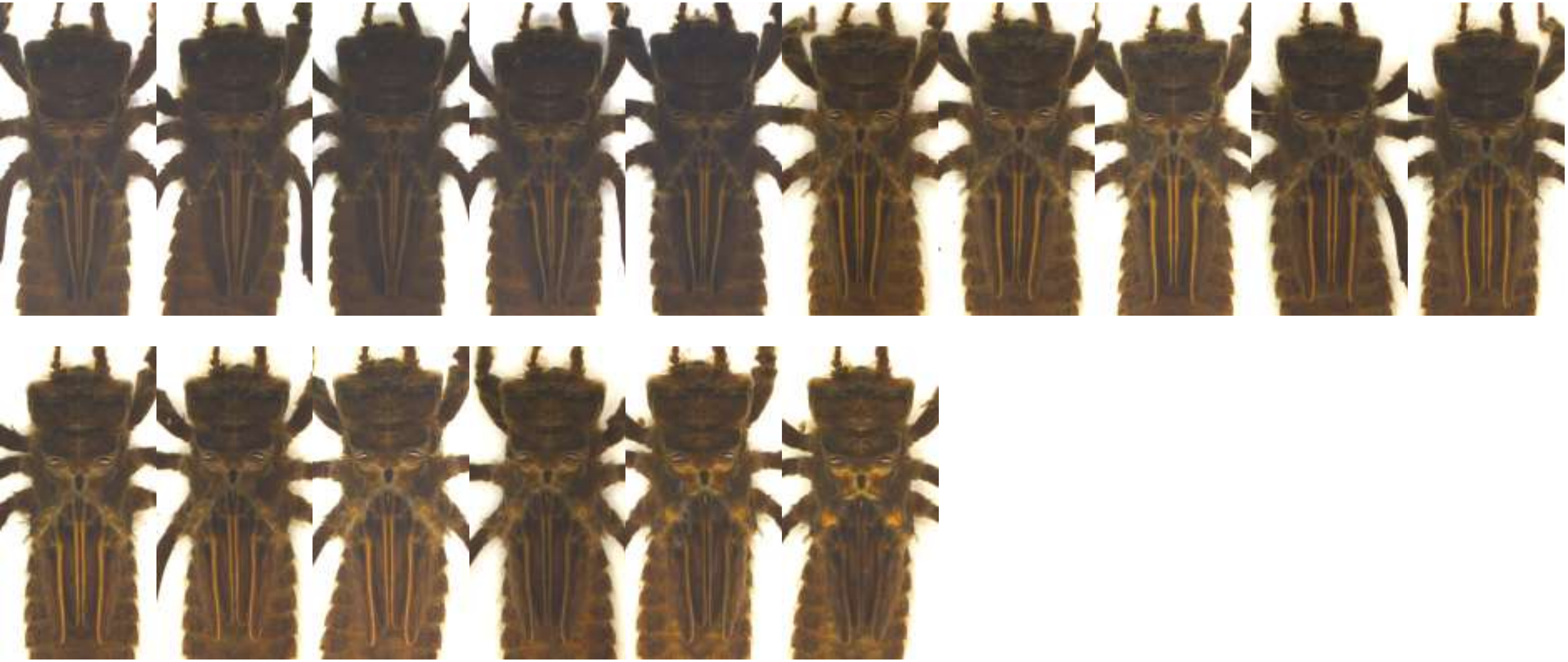

32-3 *Tanypteryx pryeri* (1/1)

5 mm

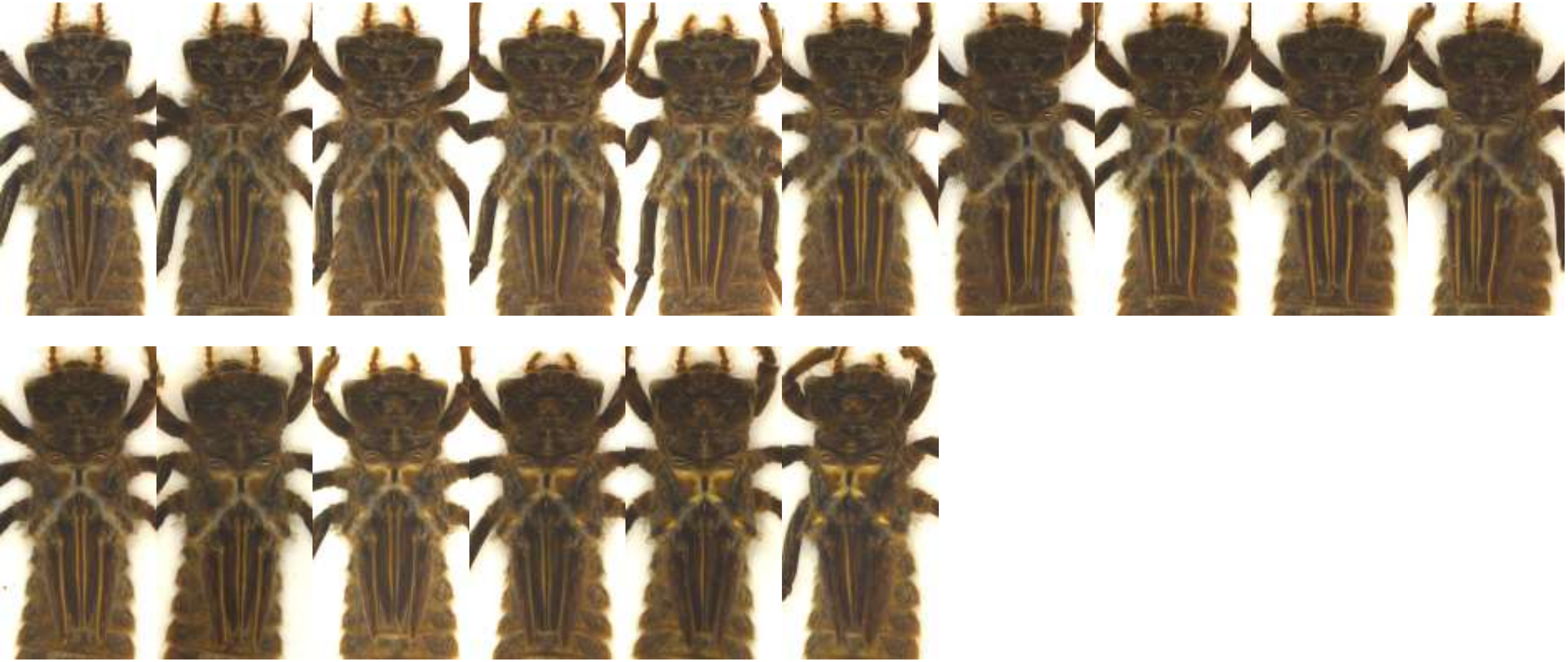

32-4 *Tanypteryx pryeri* (1/1)

5 mm

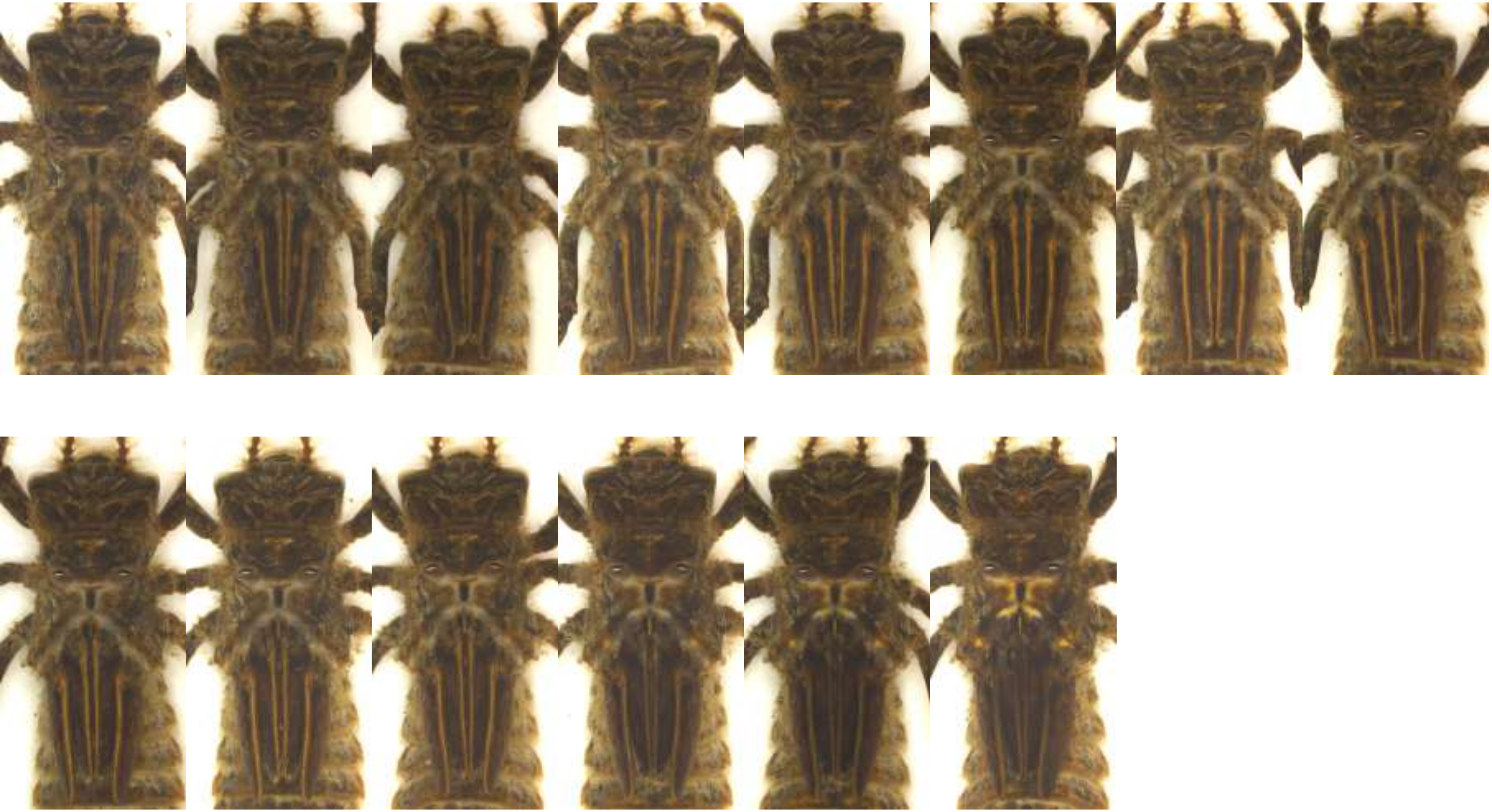

33-1 *Chlorogomphus brunneus* (1/2)

5 mm

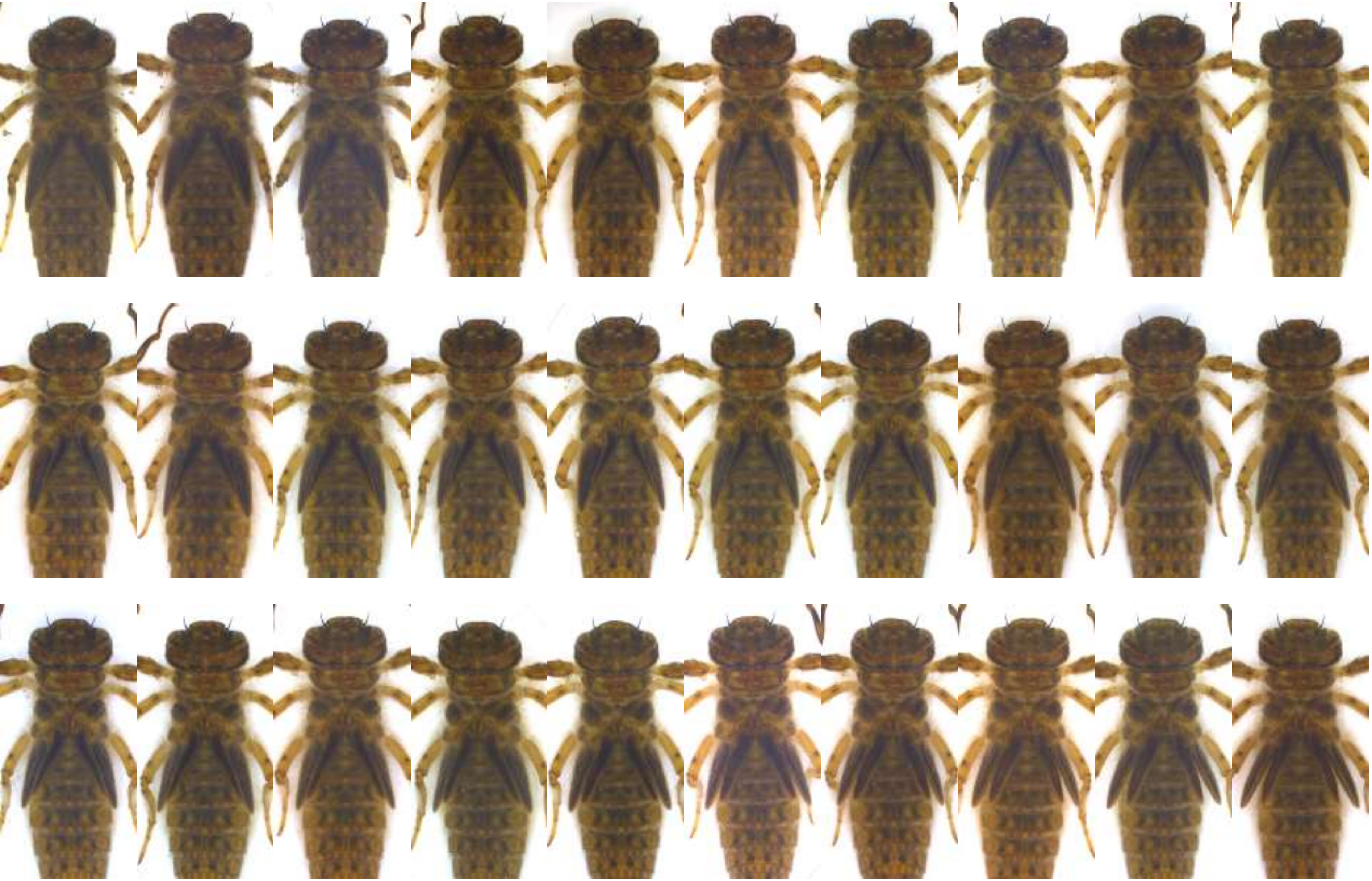

33-1 *Chlorogomphus brunneus* (2/2)

5 mm

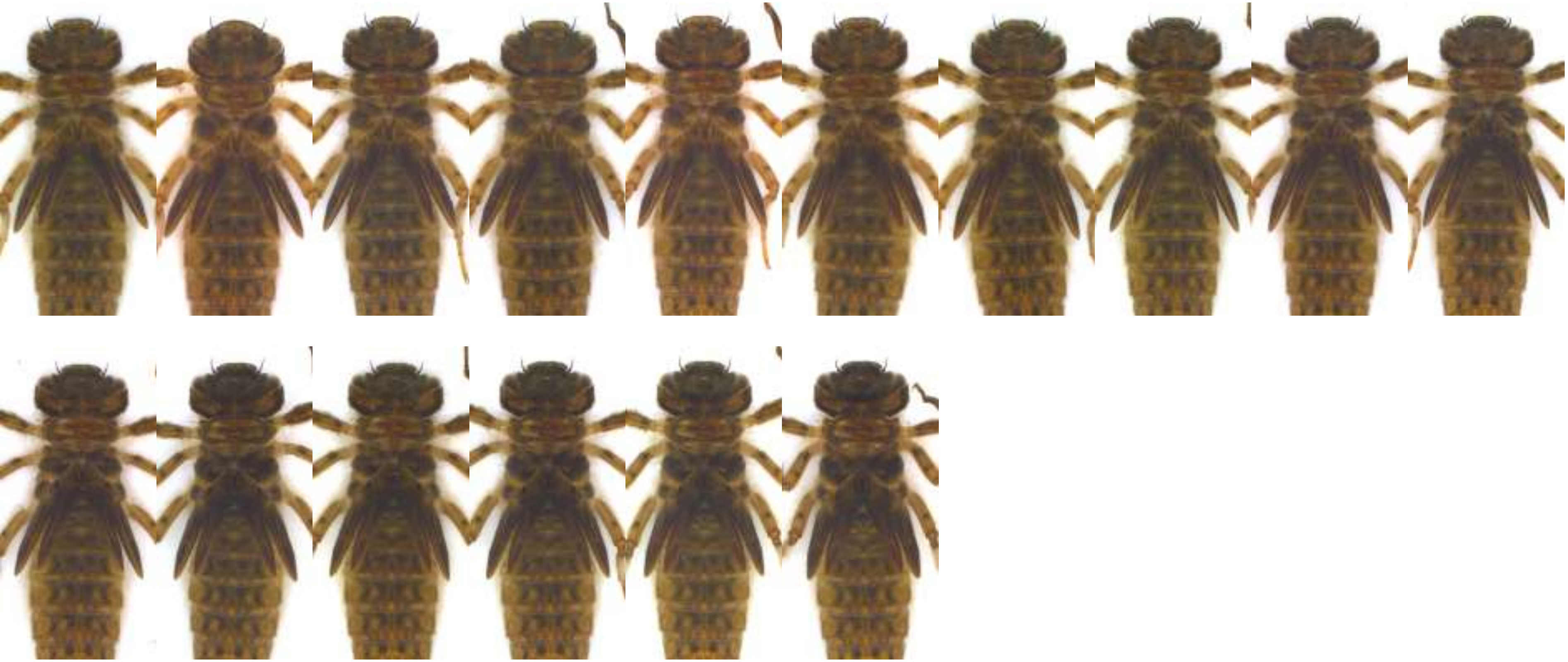

34-1 *Anotogaster sieboldii* (1/2)

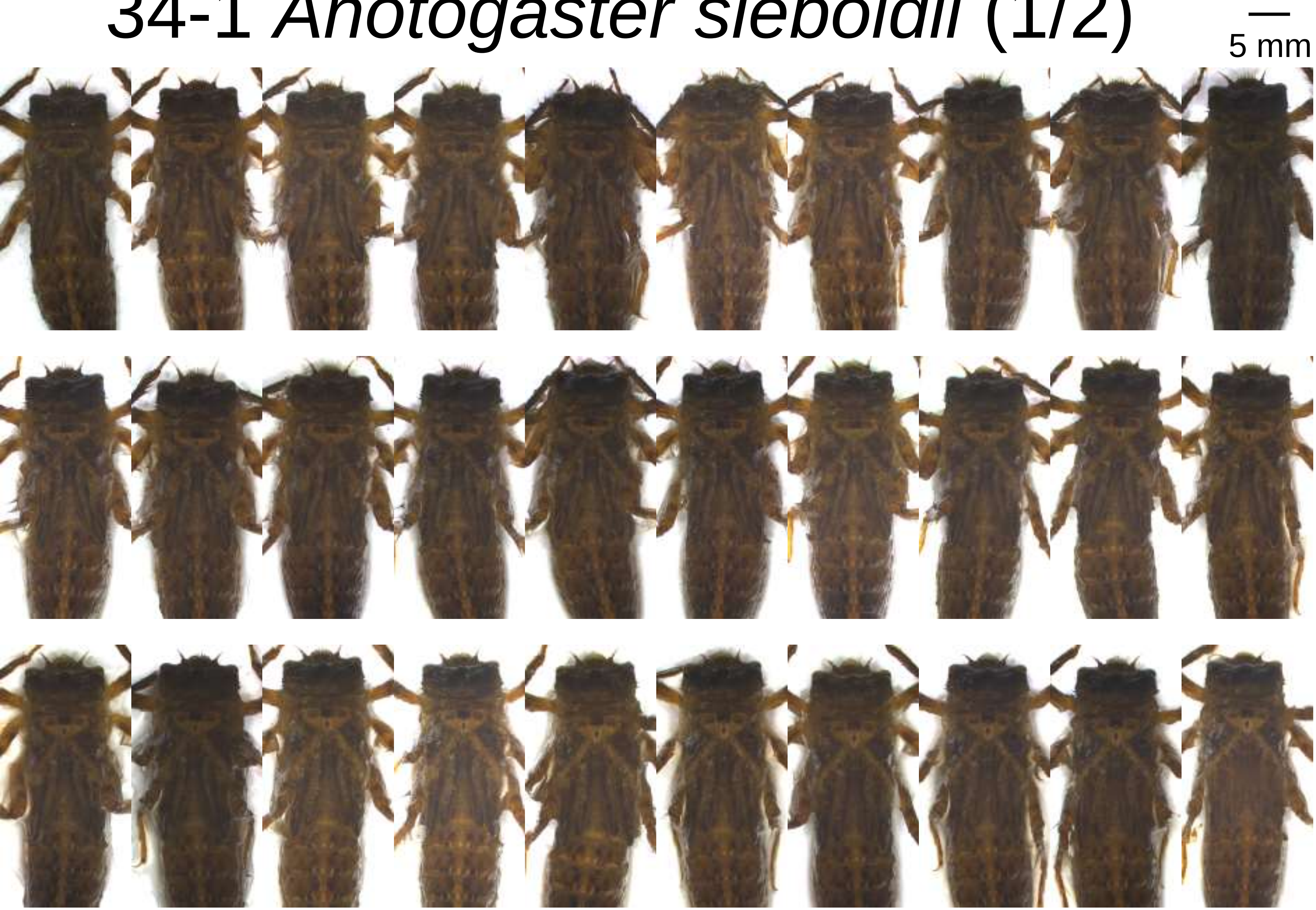

34-1 *Anotogaster sieboldii* (2/2)

5 mm

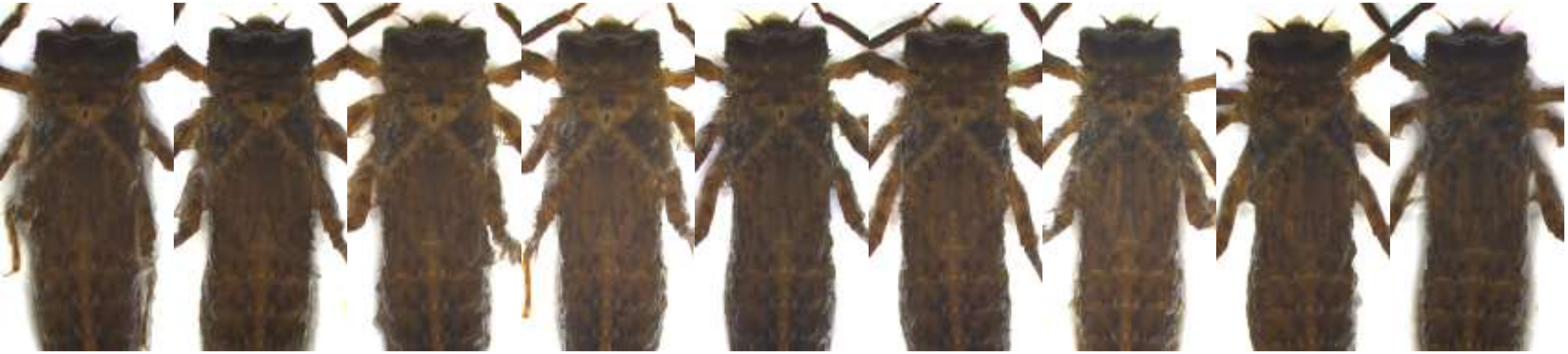

34-2 *Anotogaster sieboldii* (1/3)

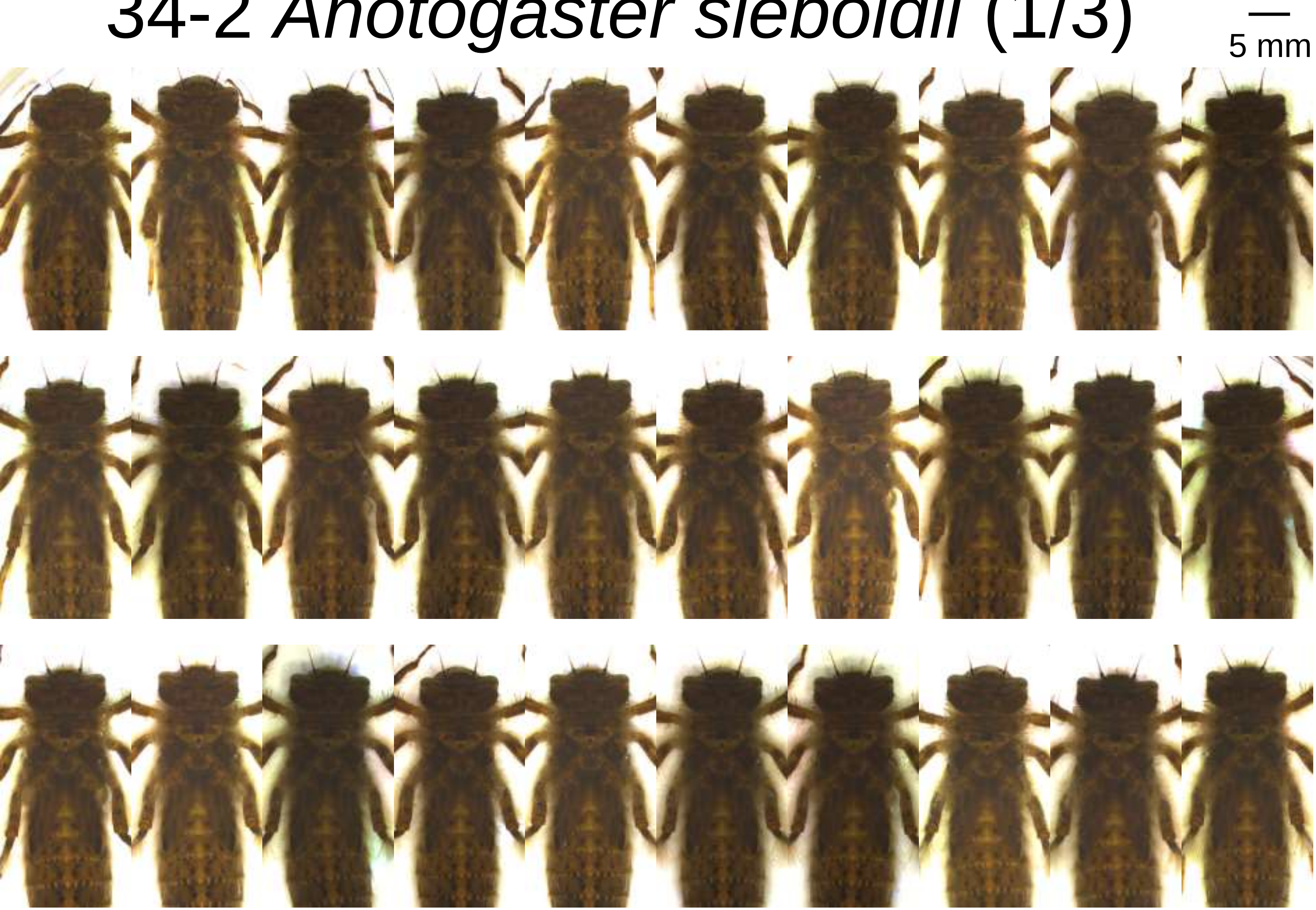

34-2 *Anotogaster sieboldii* (2/3)

5 mm

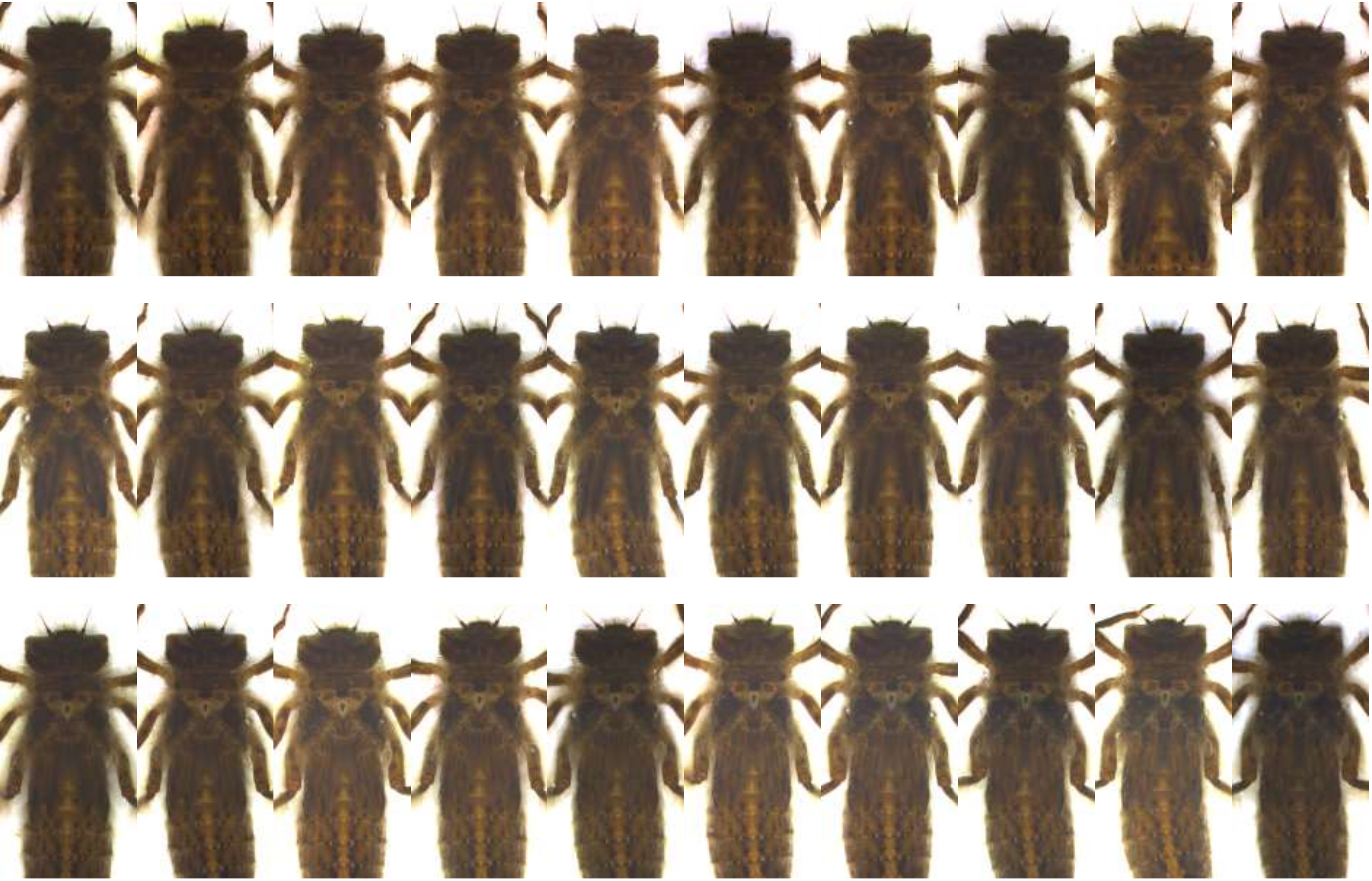

34-2 *Anotogaster sieboldii* (3/3)

5 mm

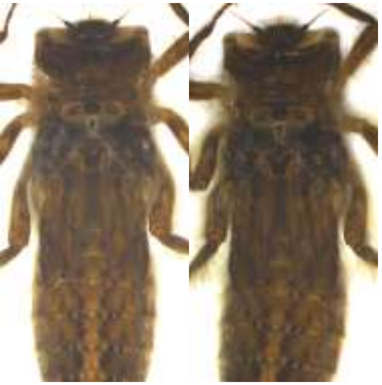

34-3 *Anotogaster sieboldii* (1/3)

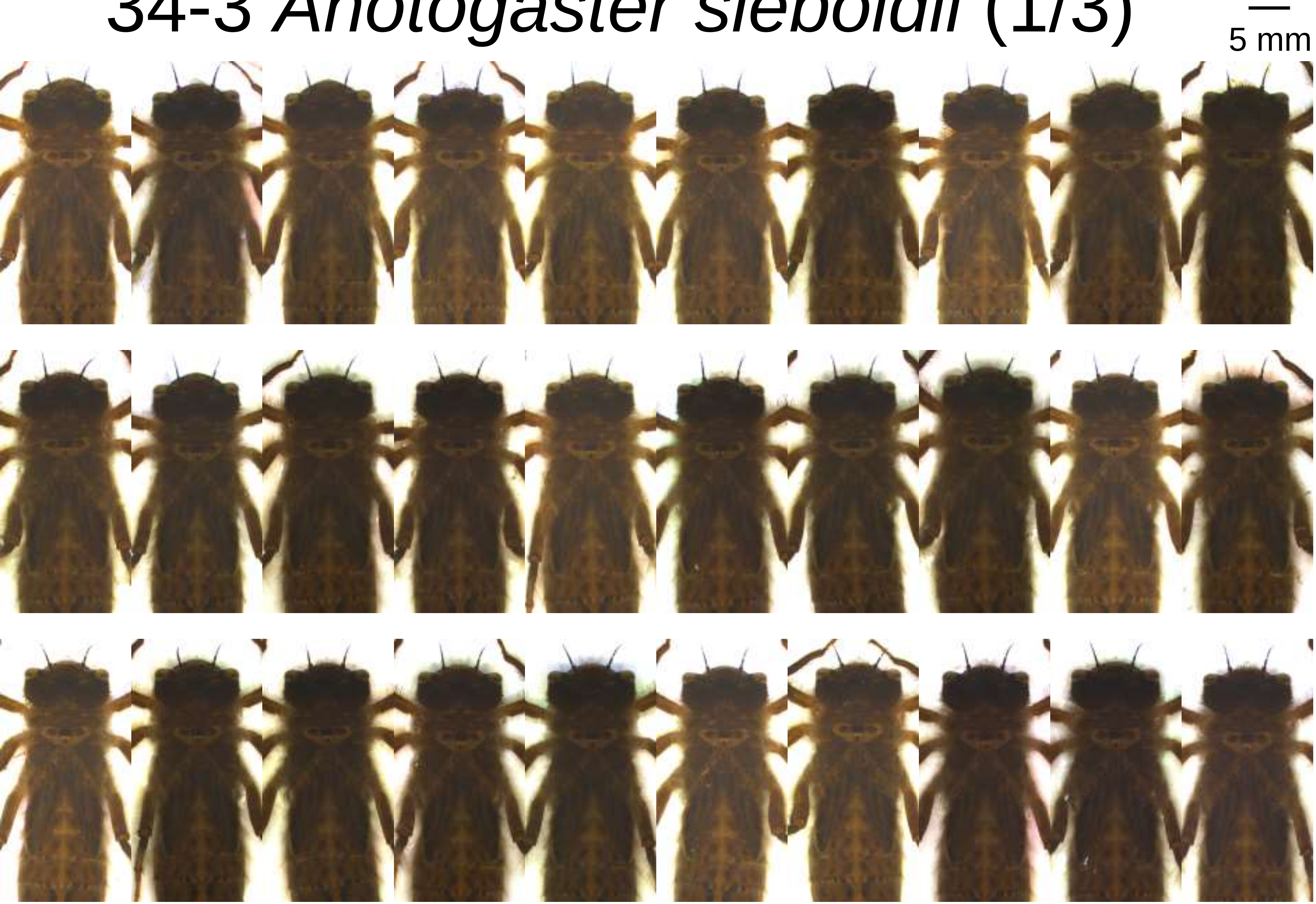

34-3 *Anotogaster sieboldii* (2/3)

5 mm

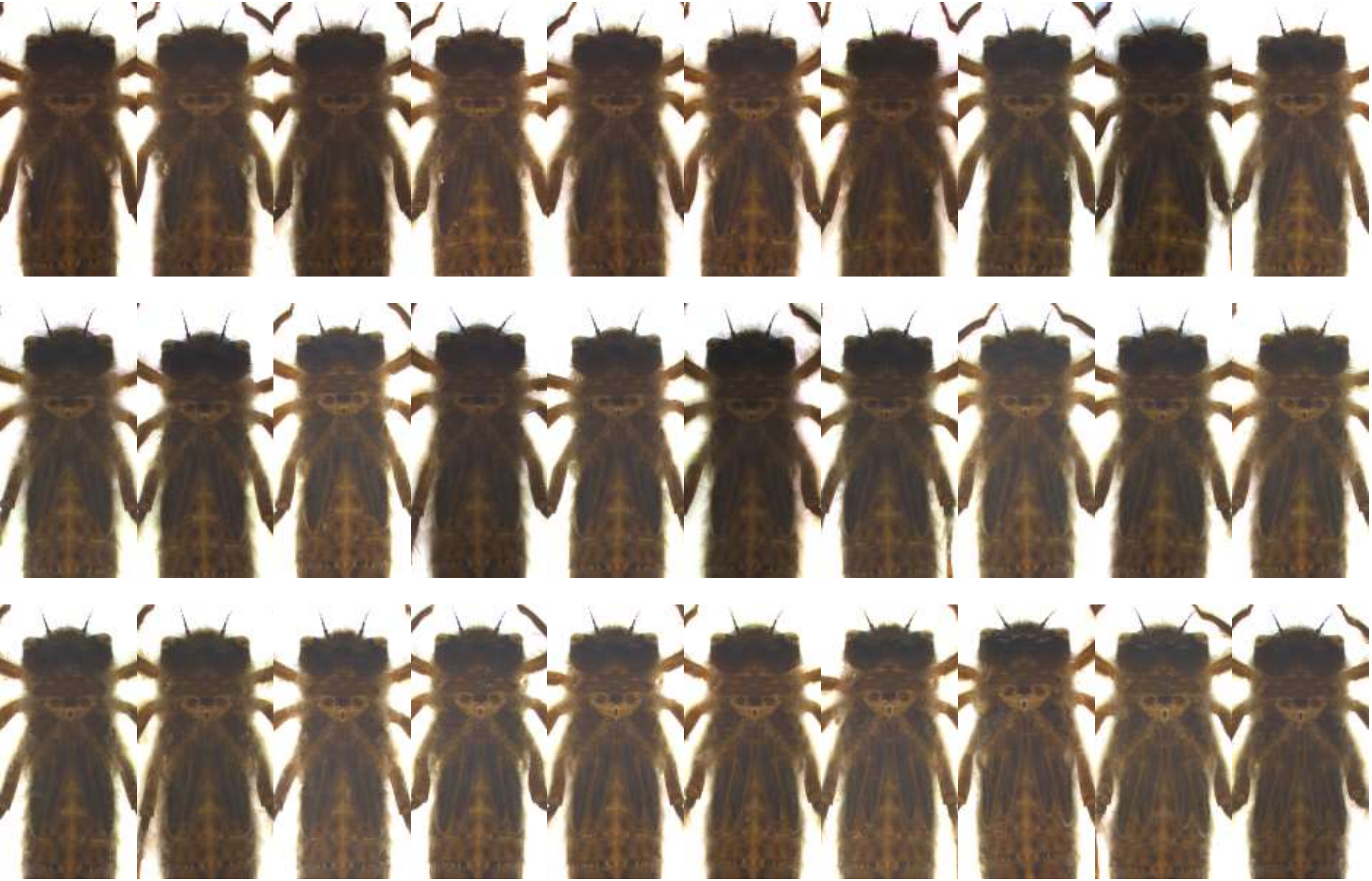

34-3 *Anotogaster sieboldii* (3/3)

5 mm

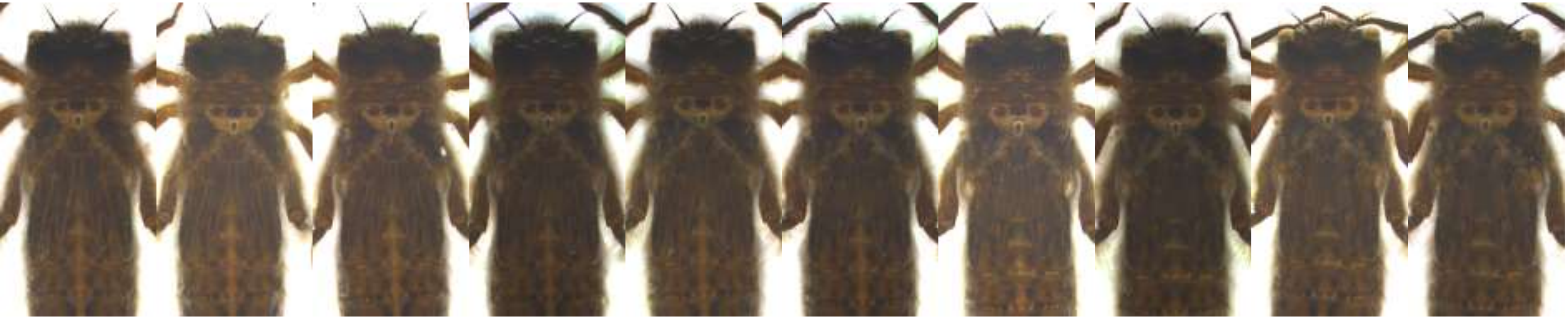

34-4 *Anotogaster sieboldii* (1/2)

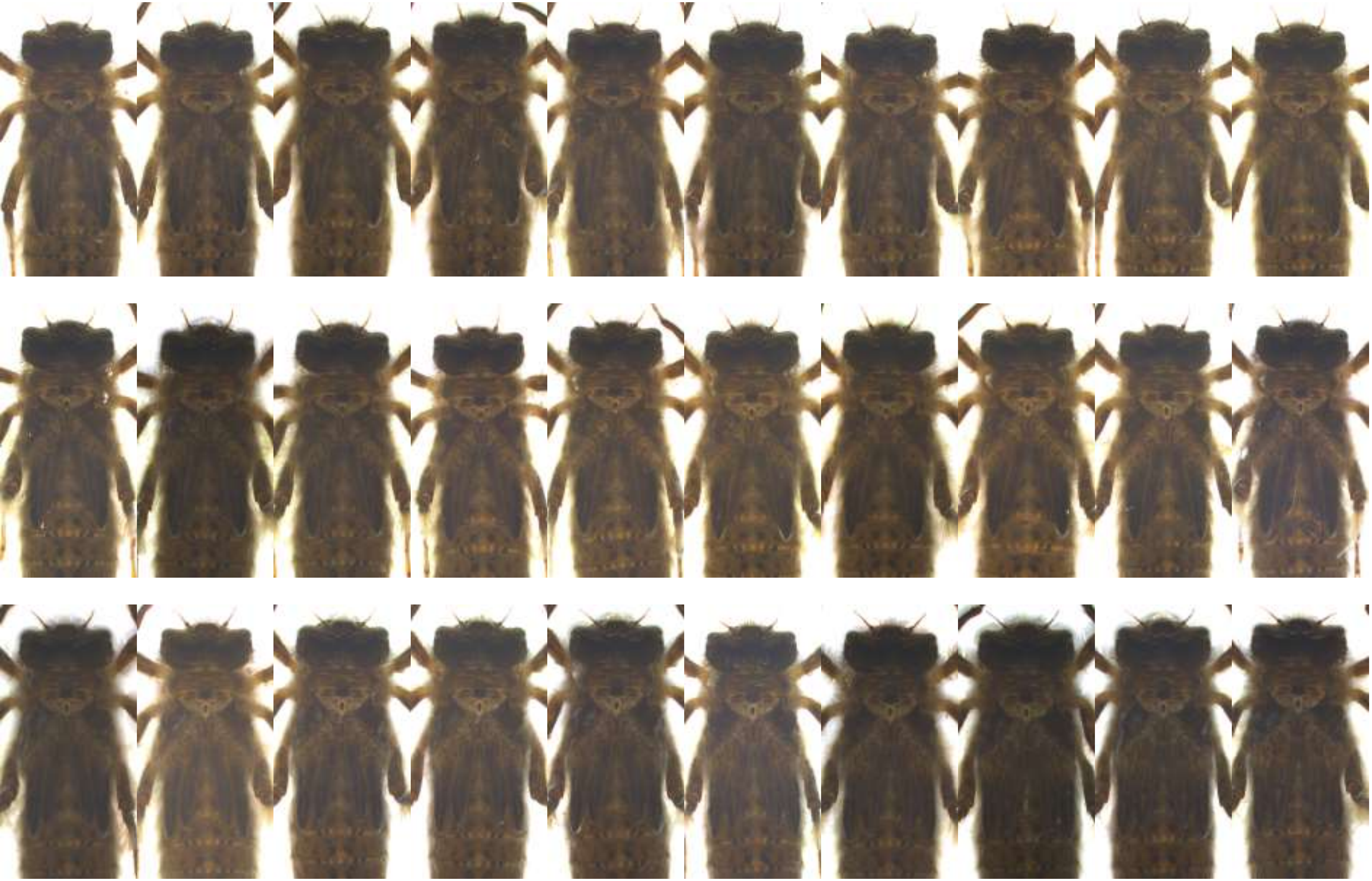

34-4 *Anotogaster sieboldii* (2/2)

5 mm

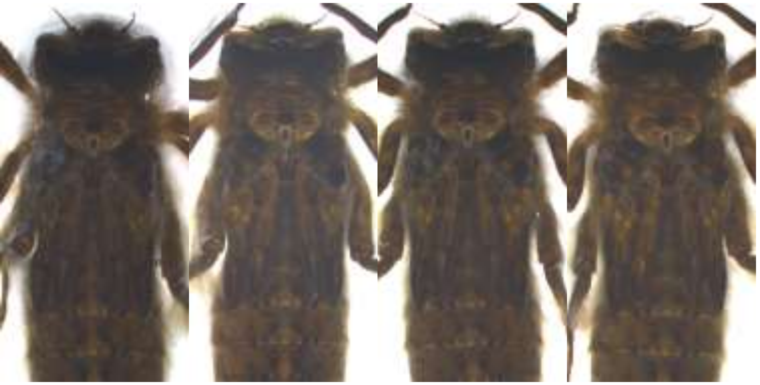

35-1 *Macromia daimoji* (1/3)

5 mm

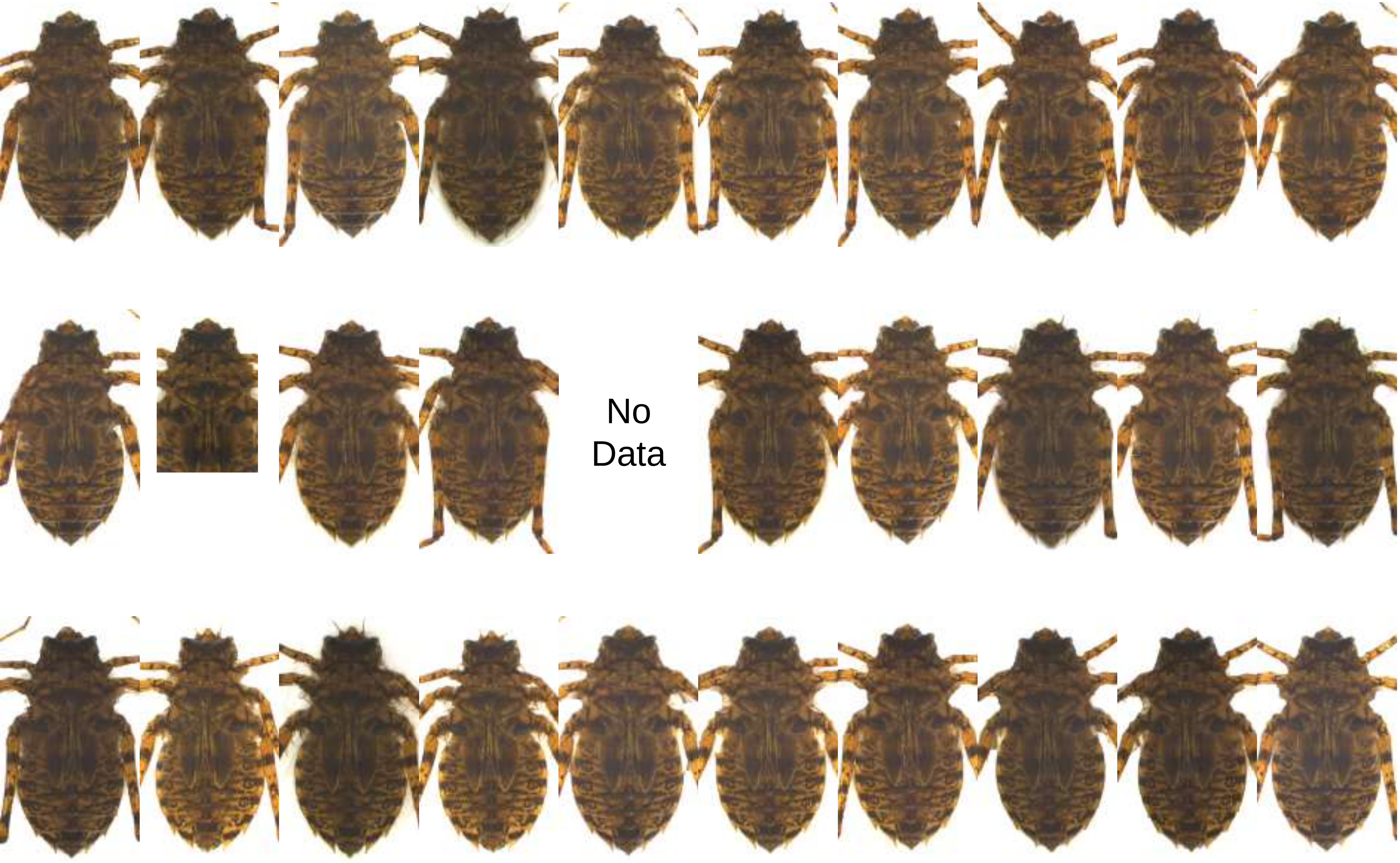

35-1 *Macromia daimoji* (2/3)

– 5 mm

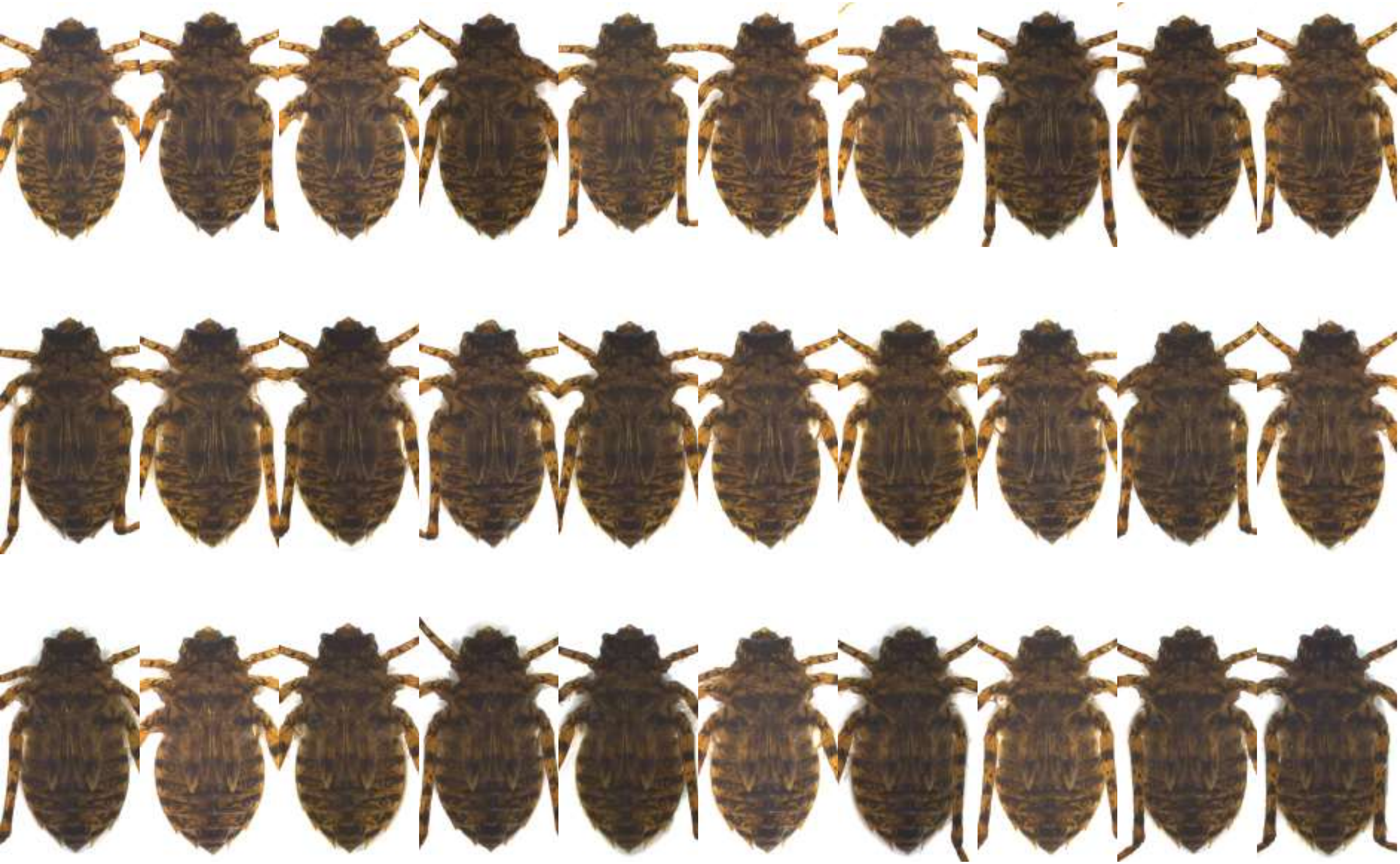

35-1 *Macromia daimoji* (3/3)

–
5 mm

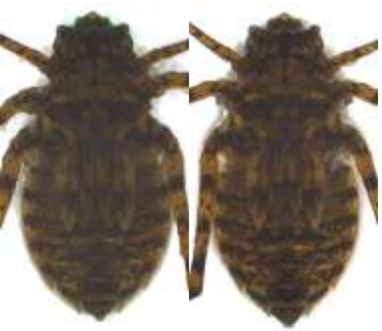

35-2 *Macromia daimoji* (1/4)

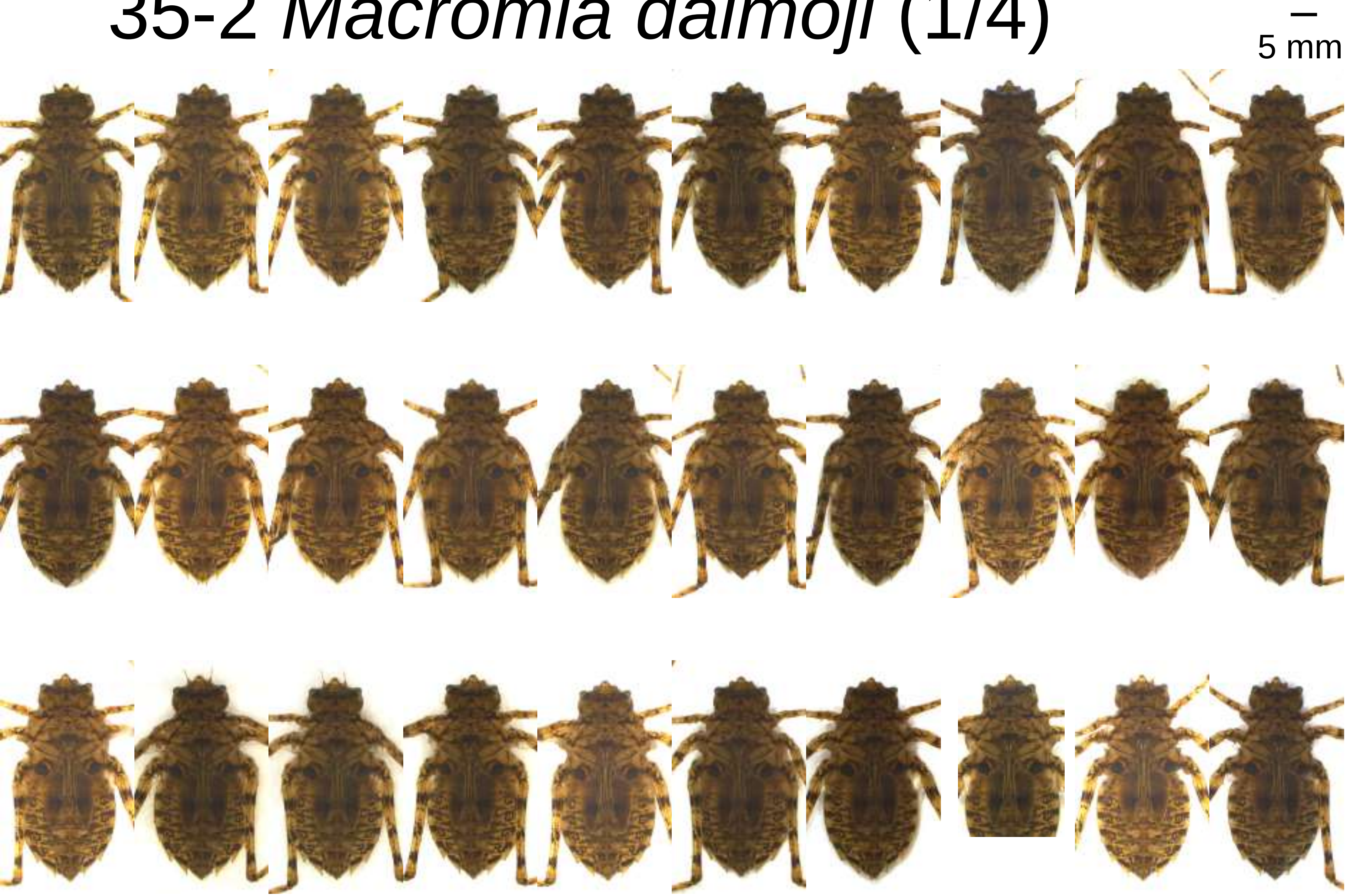

35-2 *Macromia daimoji* (2/4)

5 mm

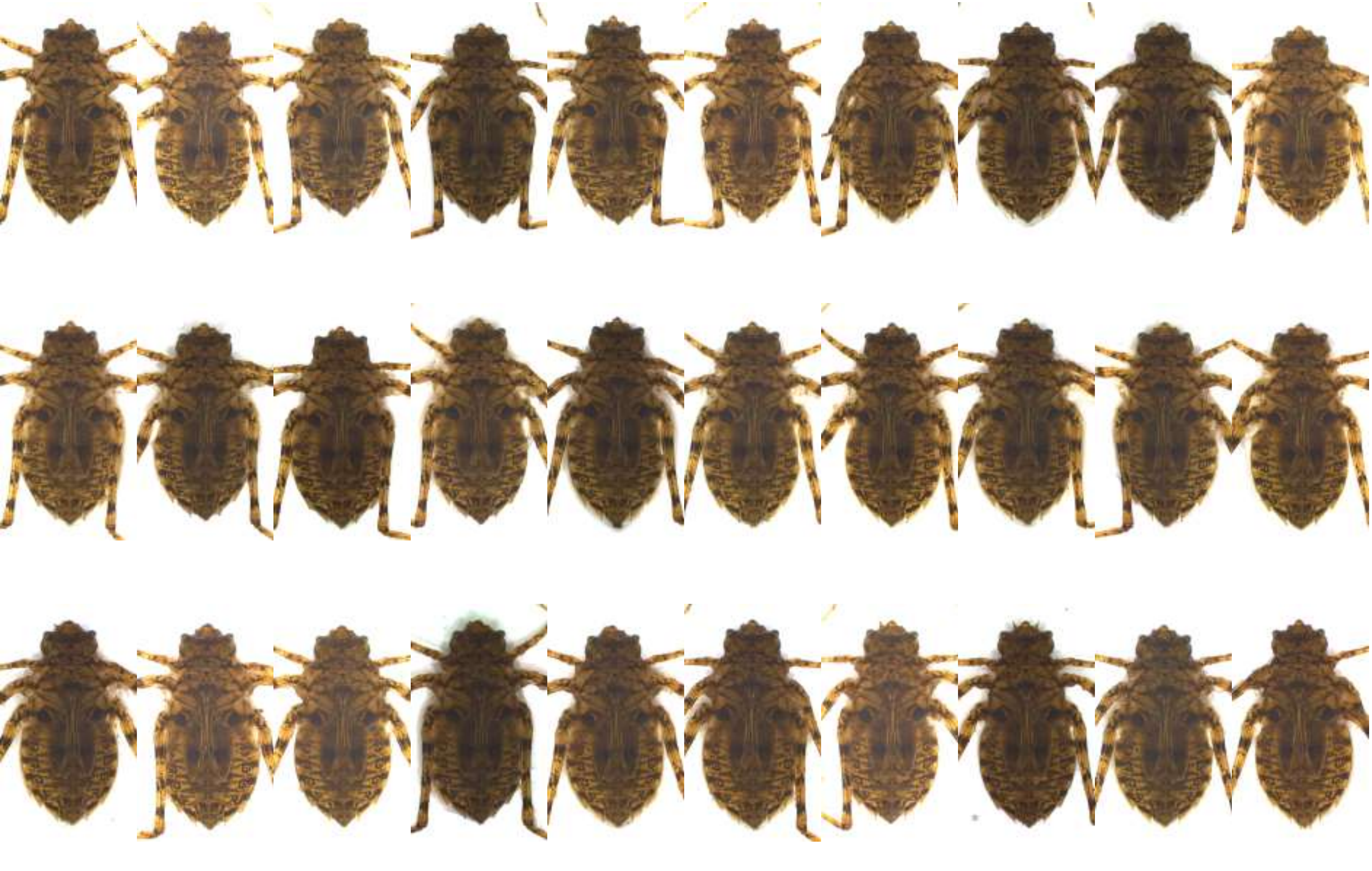

35-2 *Macromia daimoji* (3/4)

5 mm

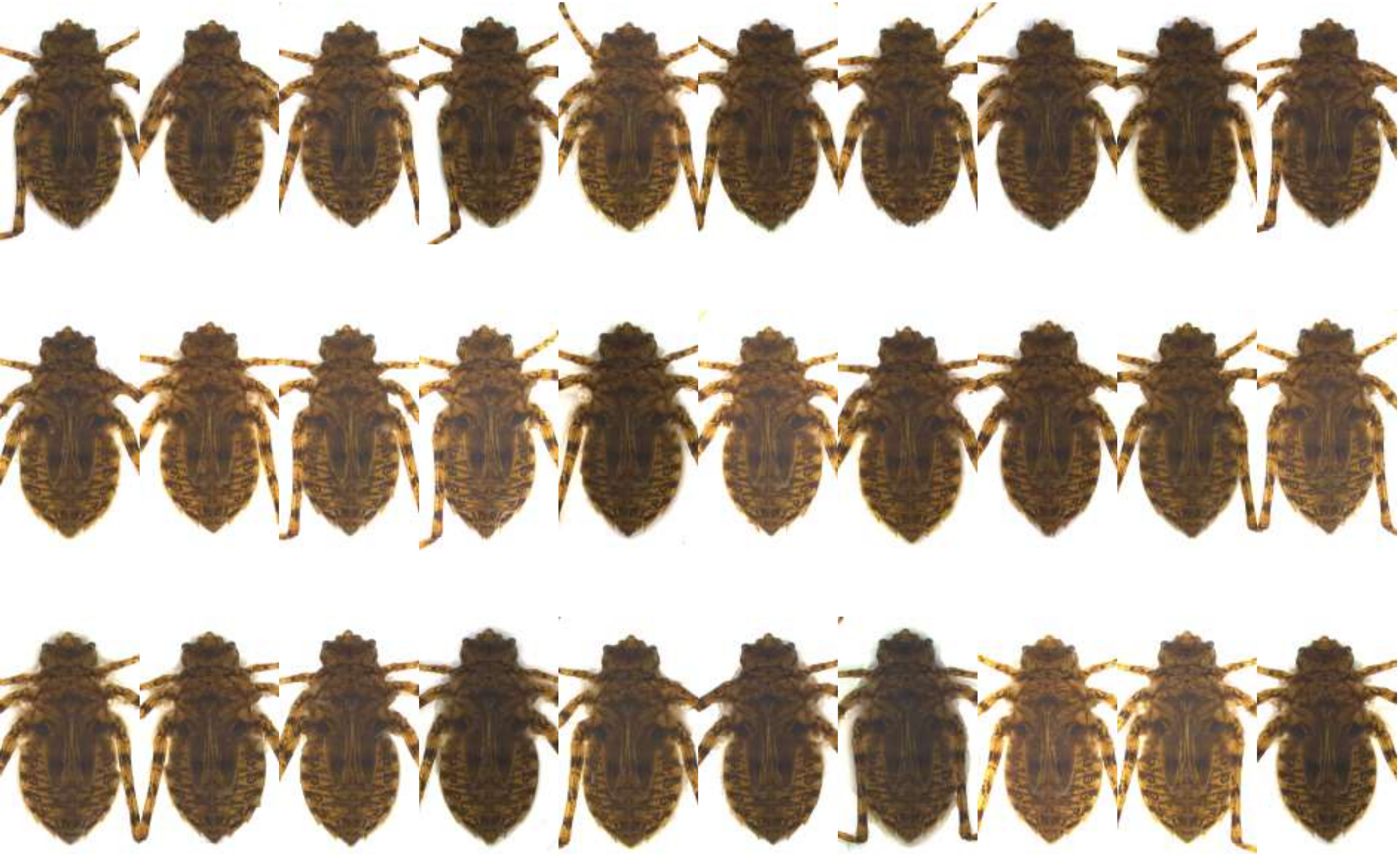

35-2 *Macromia daimoji* (4/4)

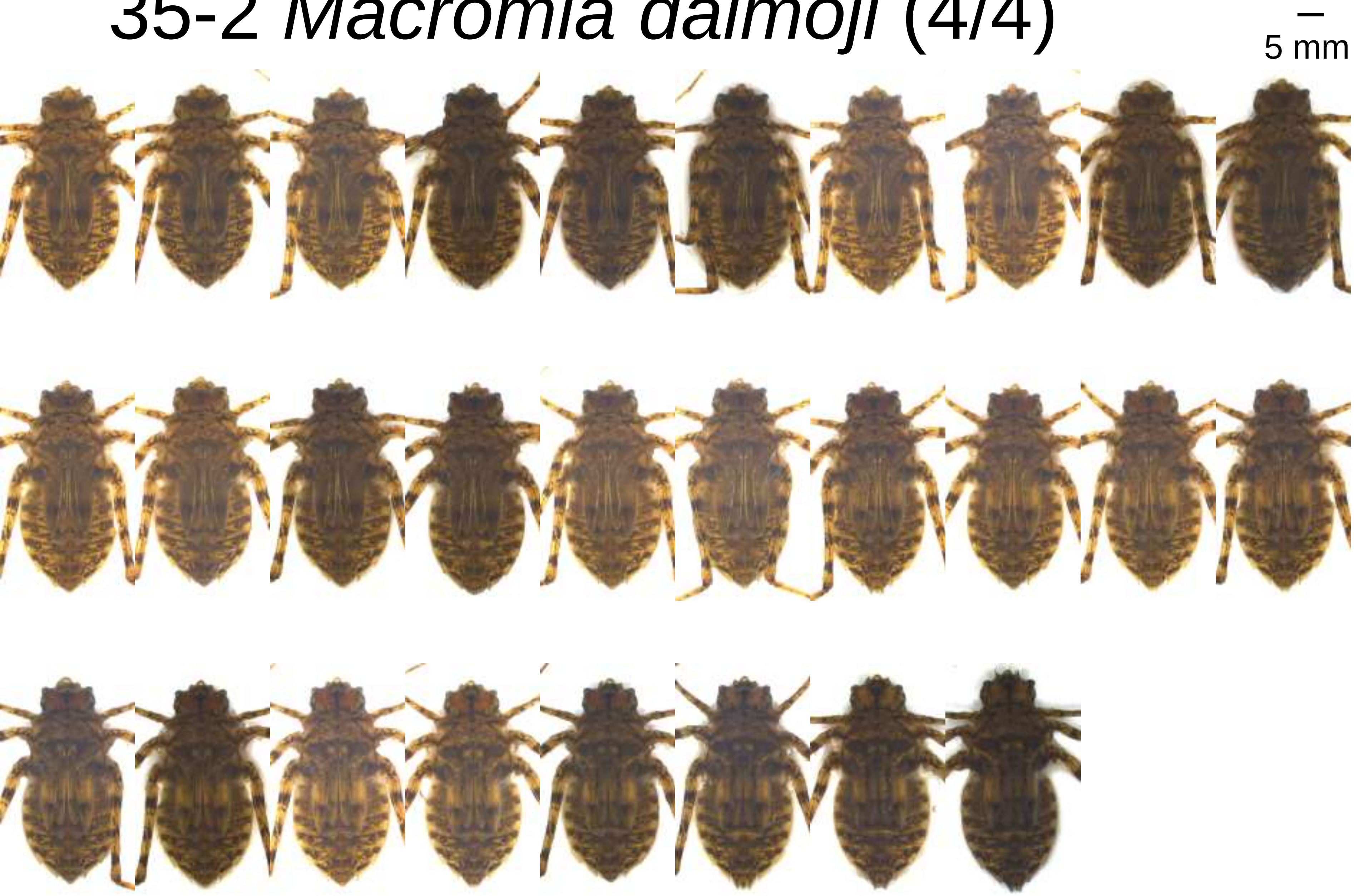

35-3 *Macromia daimoji* (1/3)

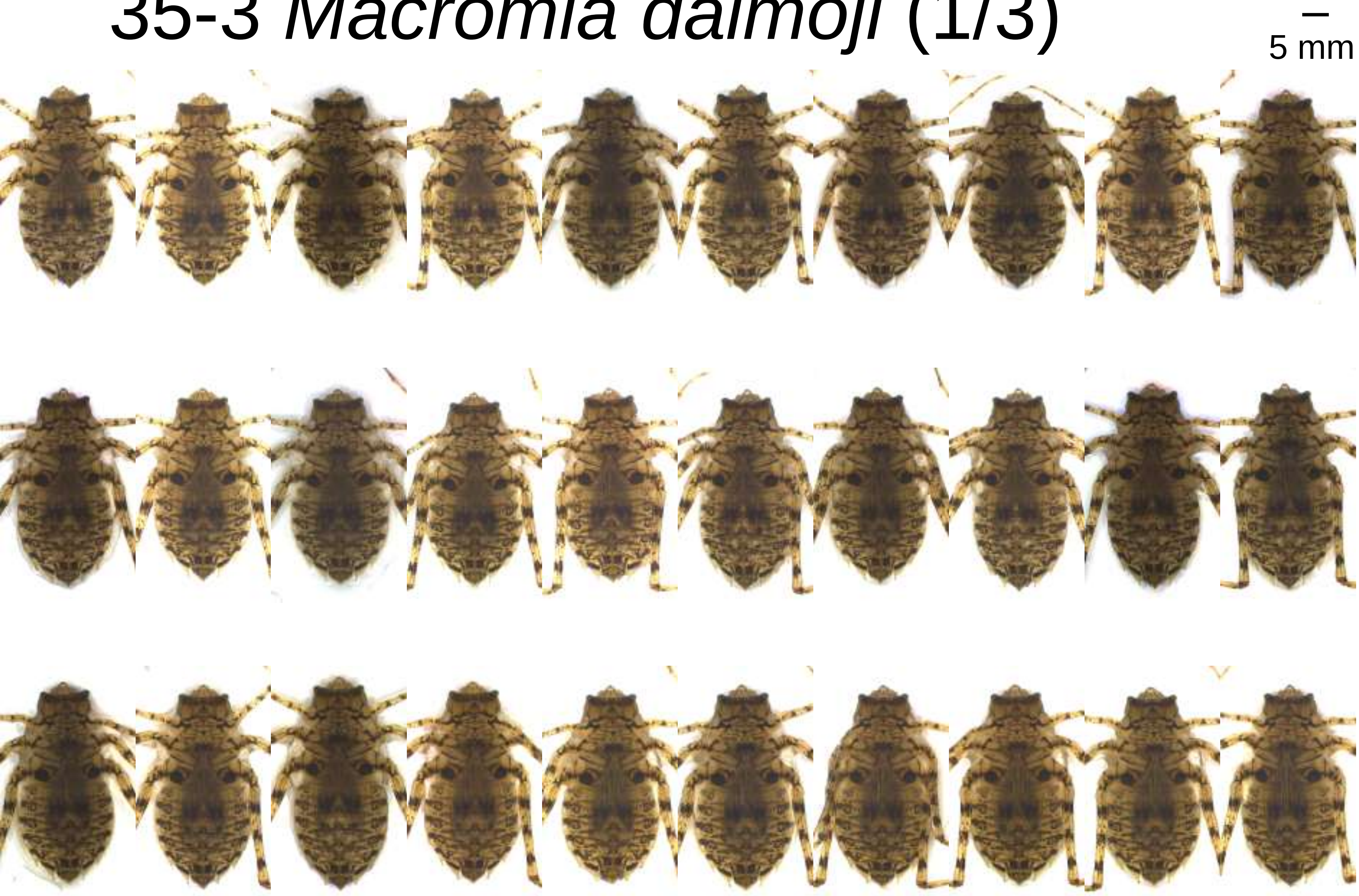

35-3 *Macromia daimoji* (2/3)

5 mm

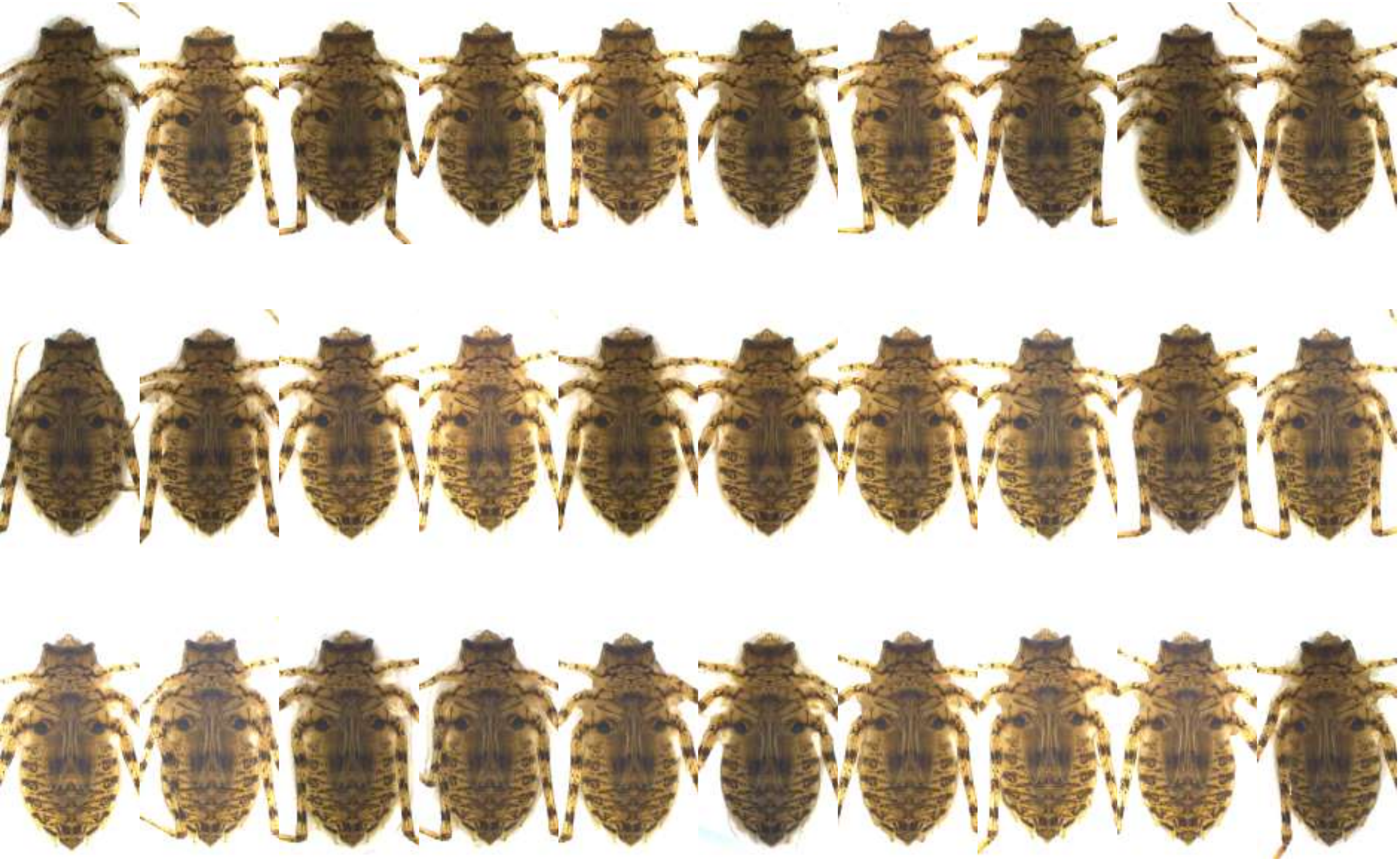

35-3 *Macromia daimoji* (3/3)

5 mm

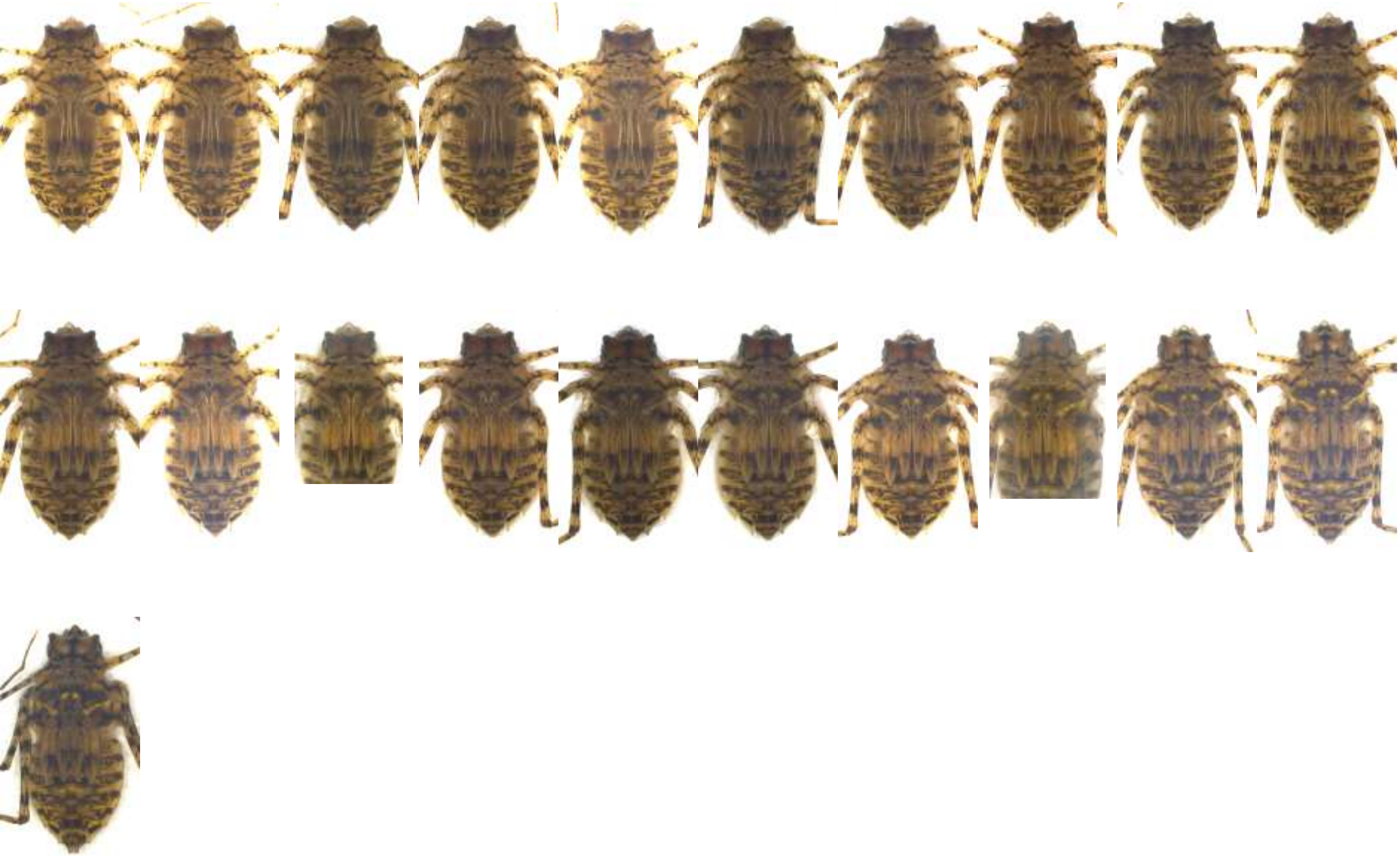

36-1 *Epitheca bimaculata* (1/1)

2 mm

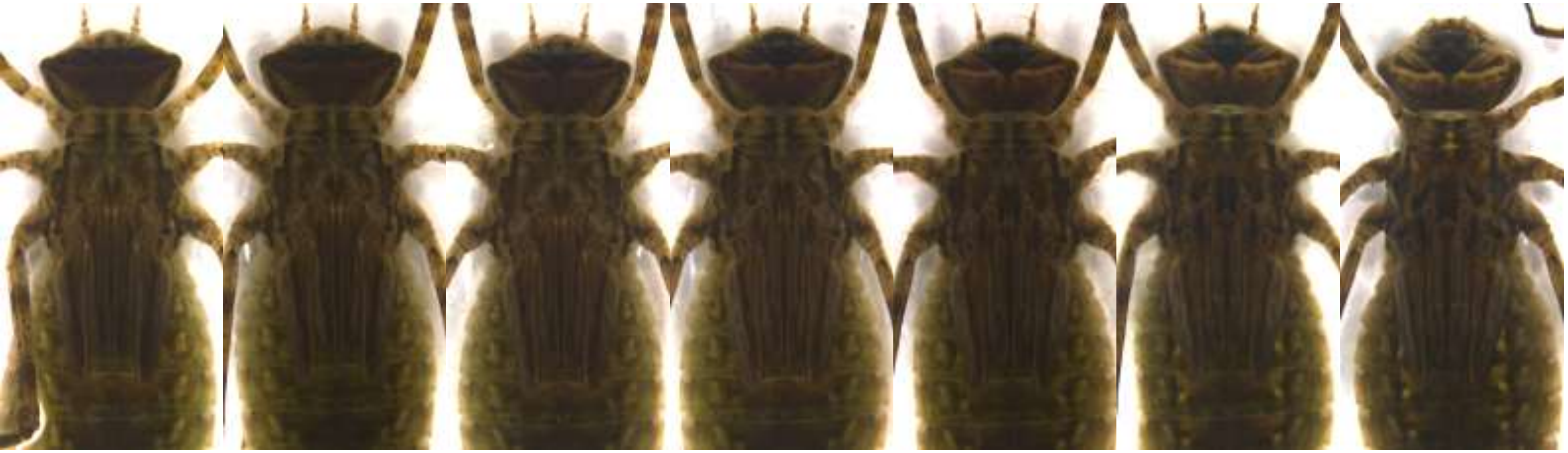

37-1 *Somatochlora uchidai* (1/1)

5 mm

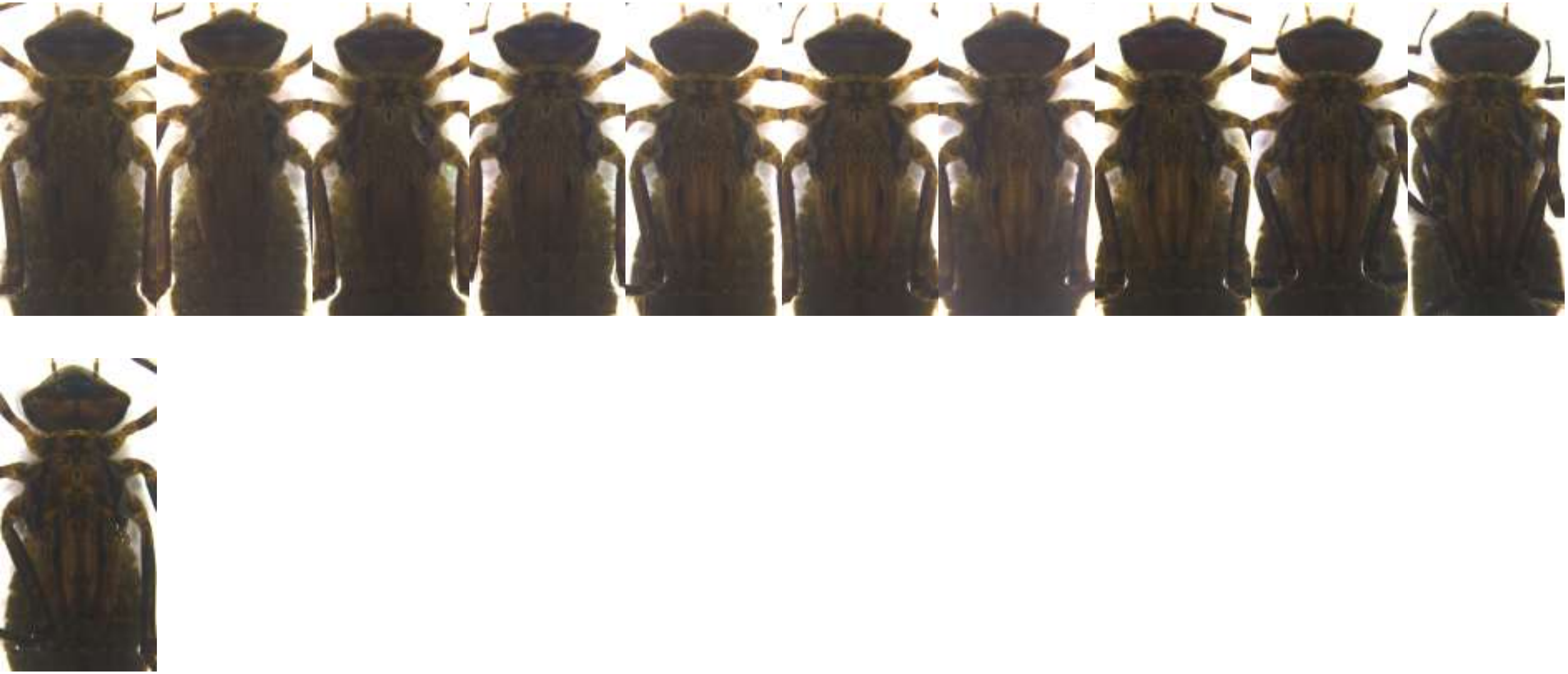

37-2 *Somatochlora uchidai* (1/1)

5 mm

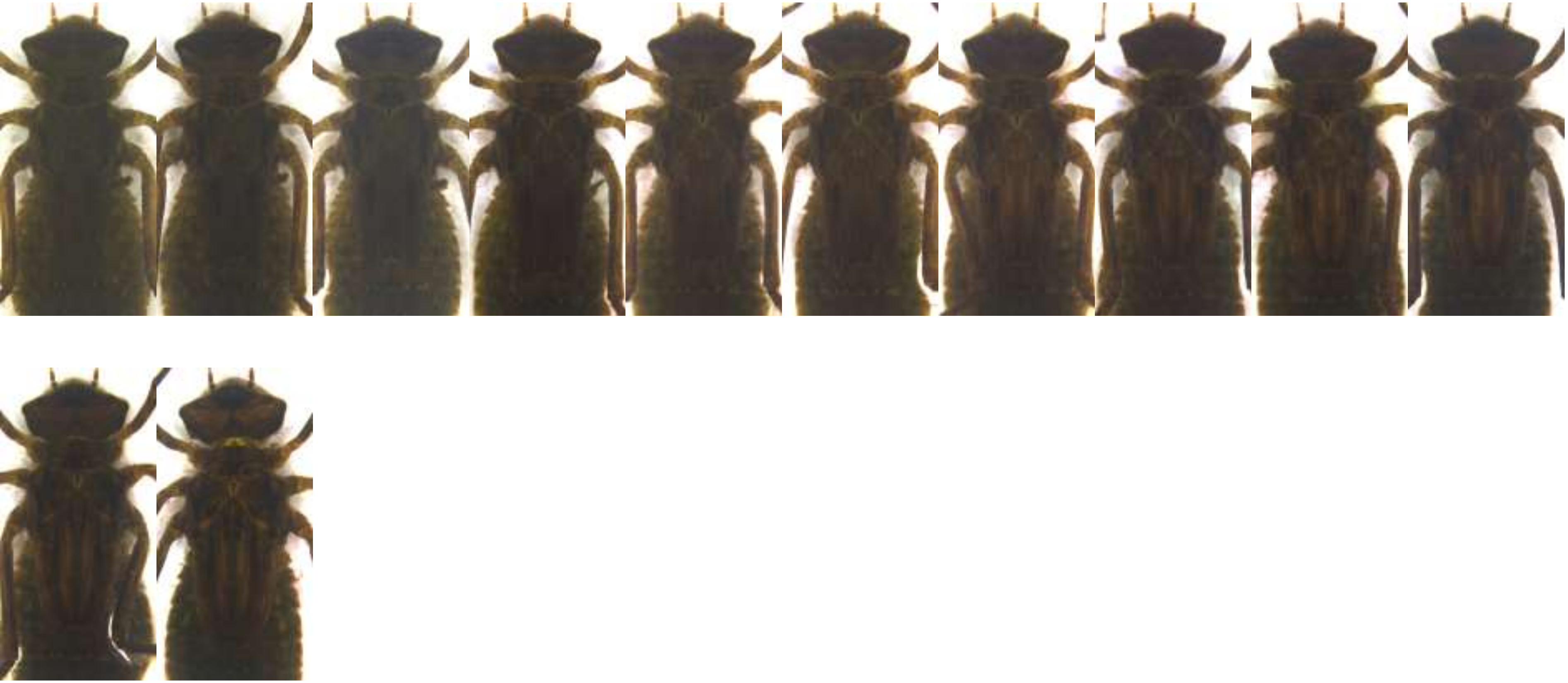

37-3 *Somatochlora uchidai* (1/1)

5 mm

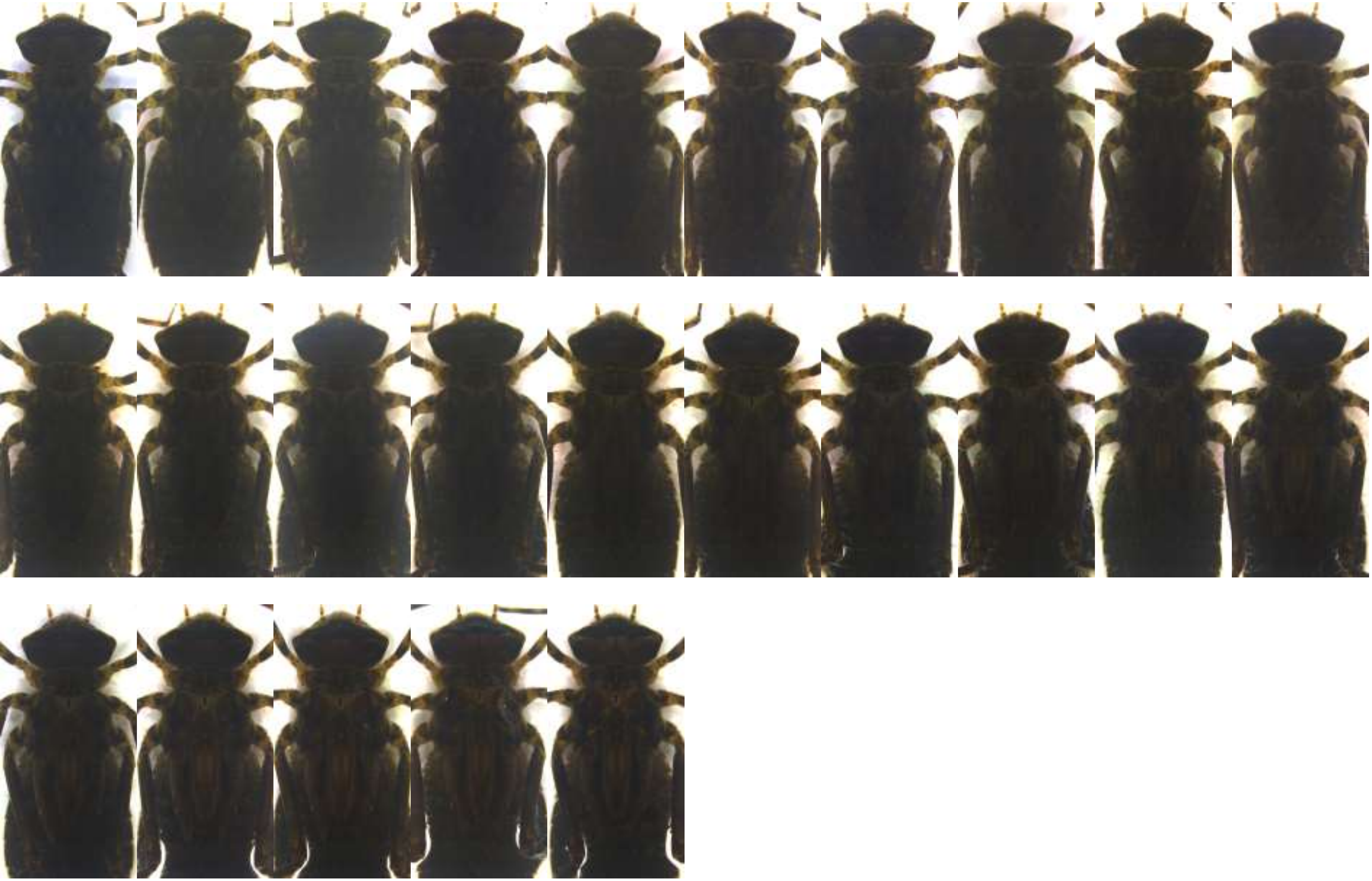

37-4 *Somatochlora uchidai* (1/1)

5 mm

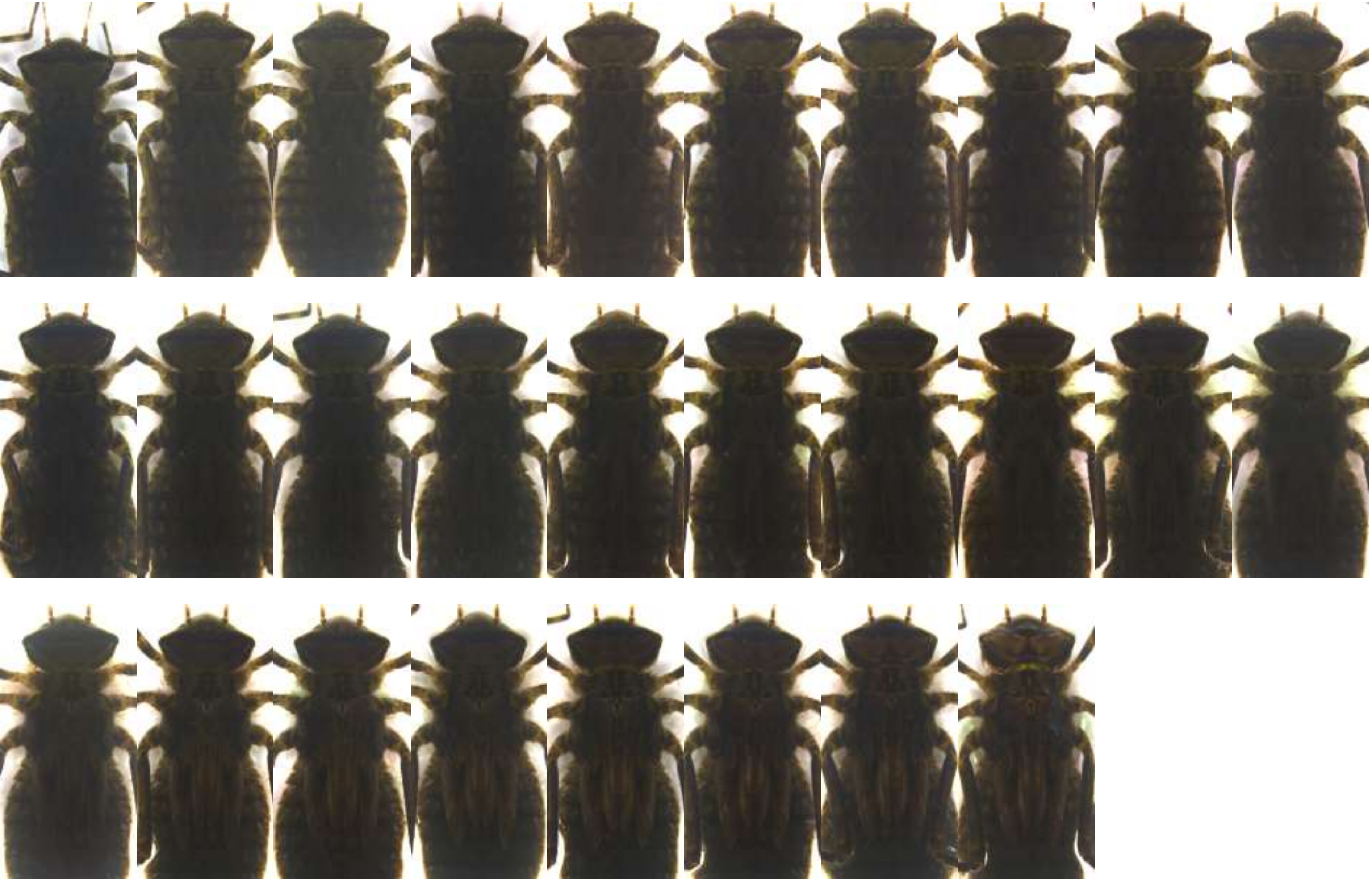

37-5 *Somatochlora uchidai* (1/1)

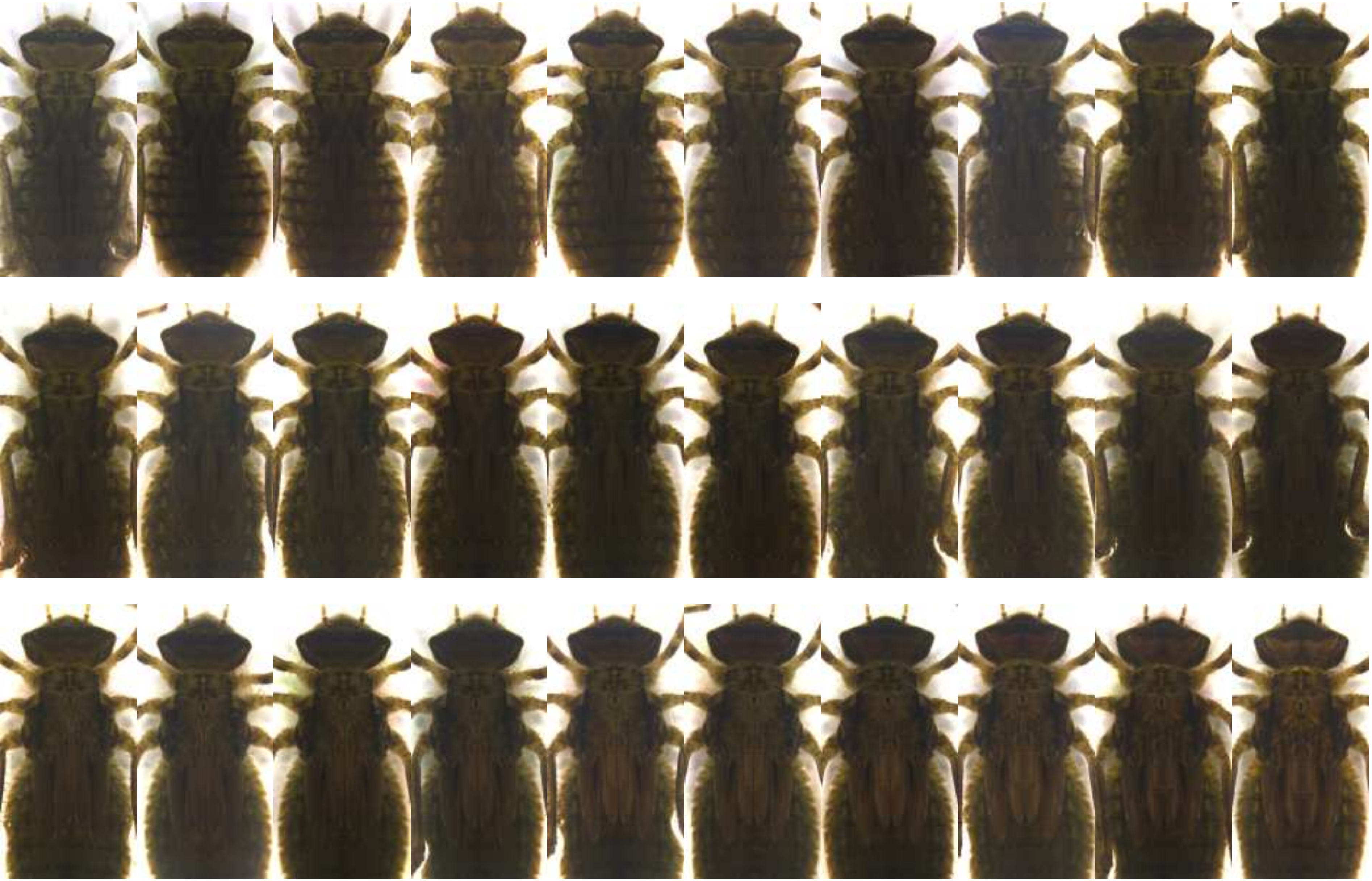

37-6 *Somatochlora uchidai* (1/1)

5 mm

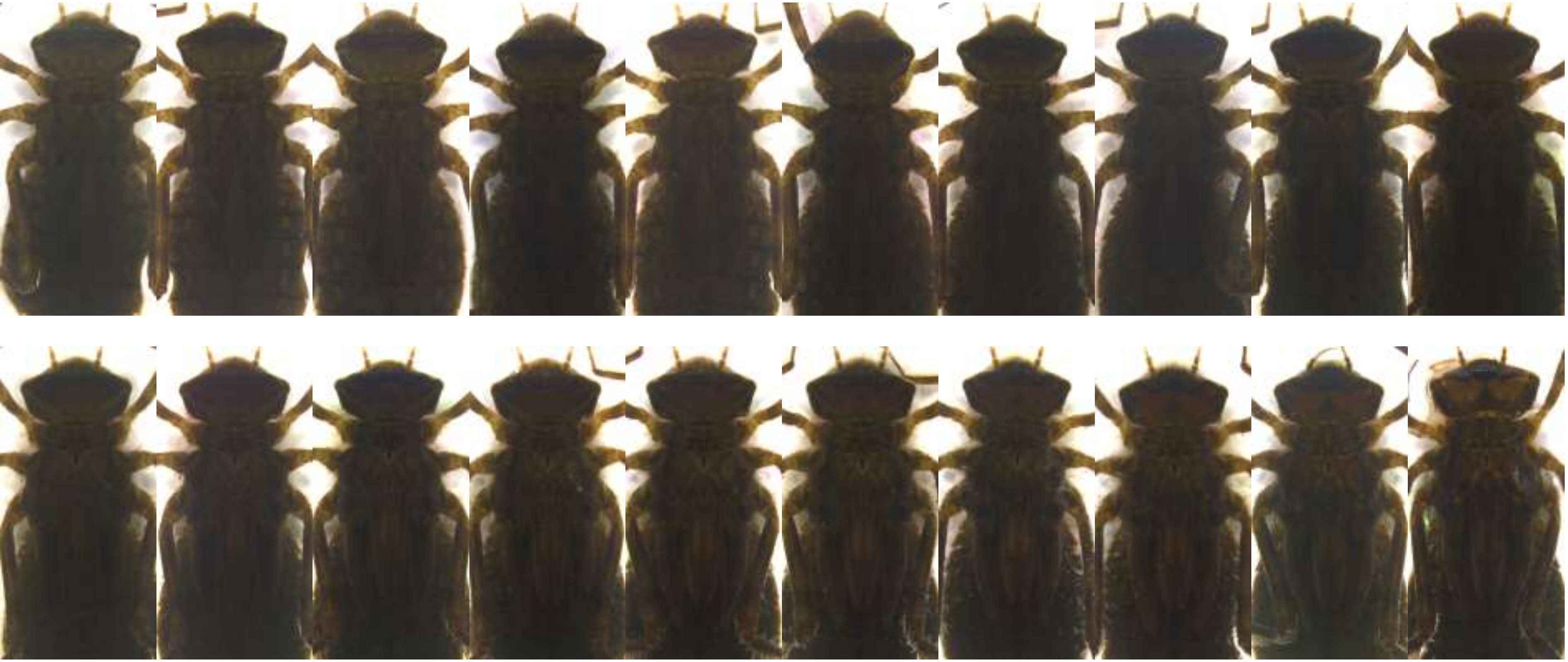

38-1 *Rhyothemis fuliginosa* (1/2)

5 mm

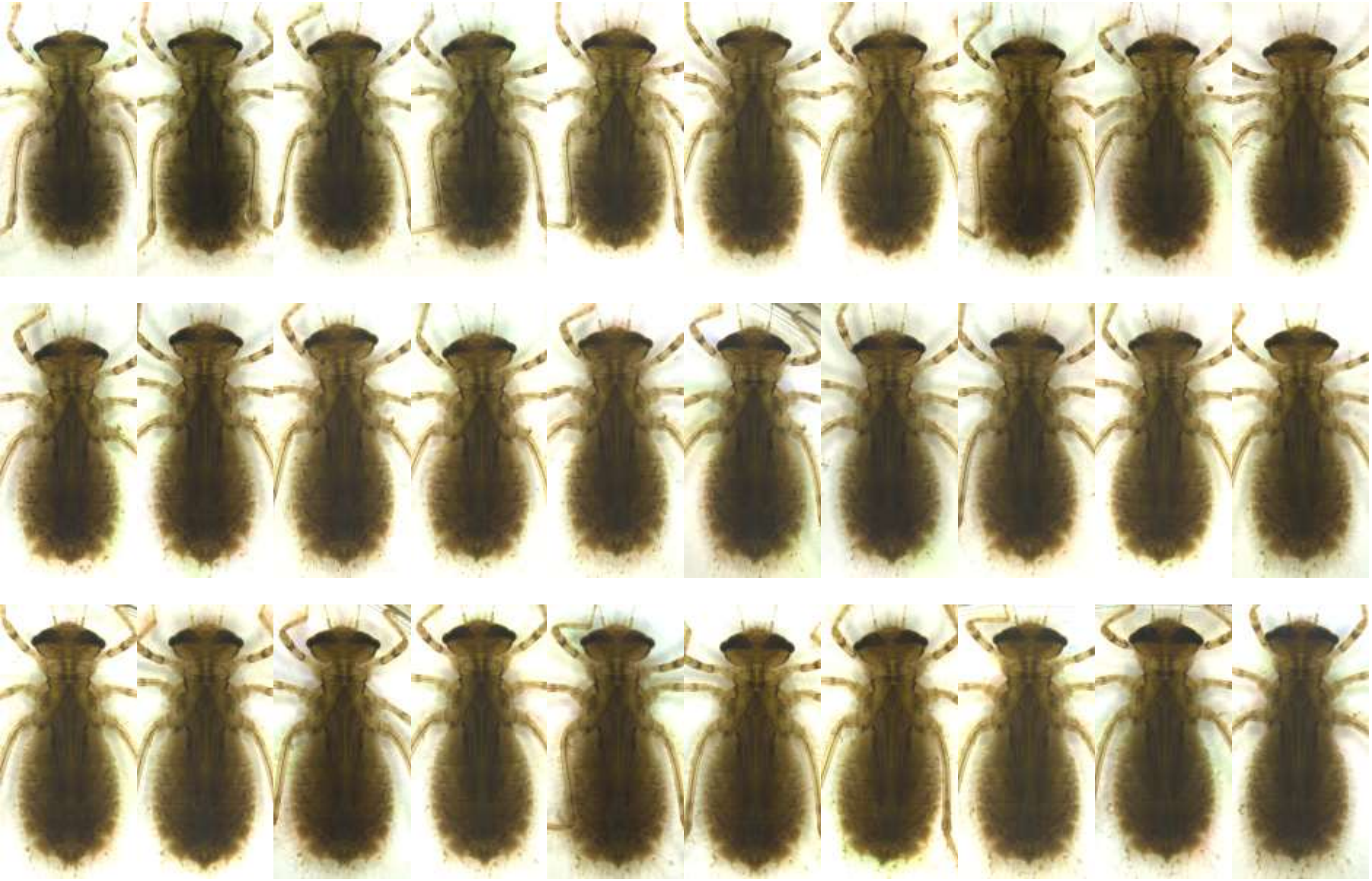

38-1 *Rhyothemis fuliginosa* (2/2)

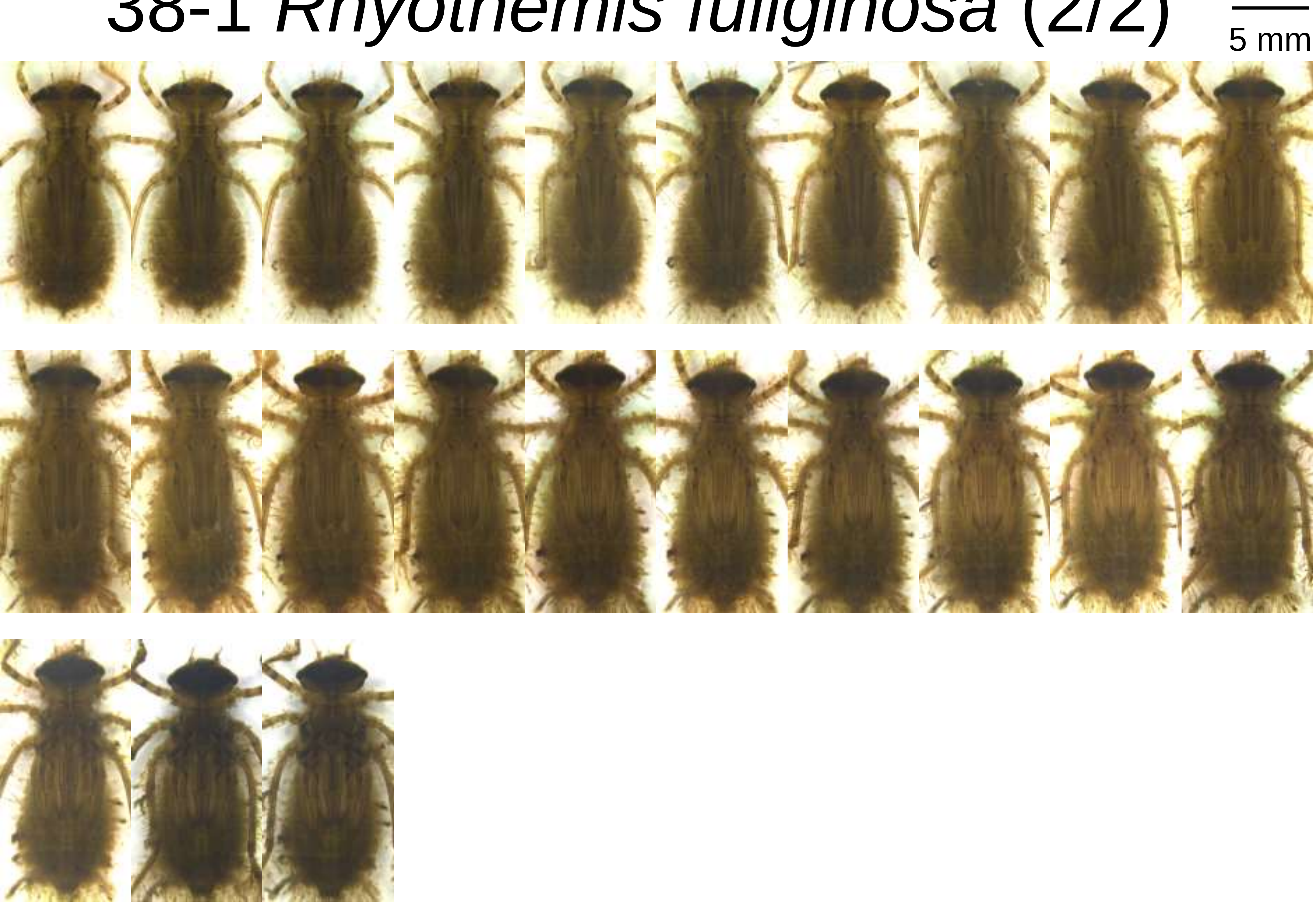

38-2 *Rhyothemis fuliginosa* (1/2)

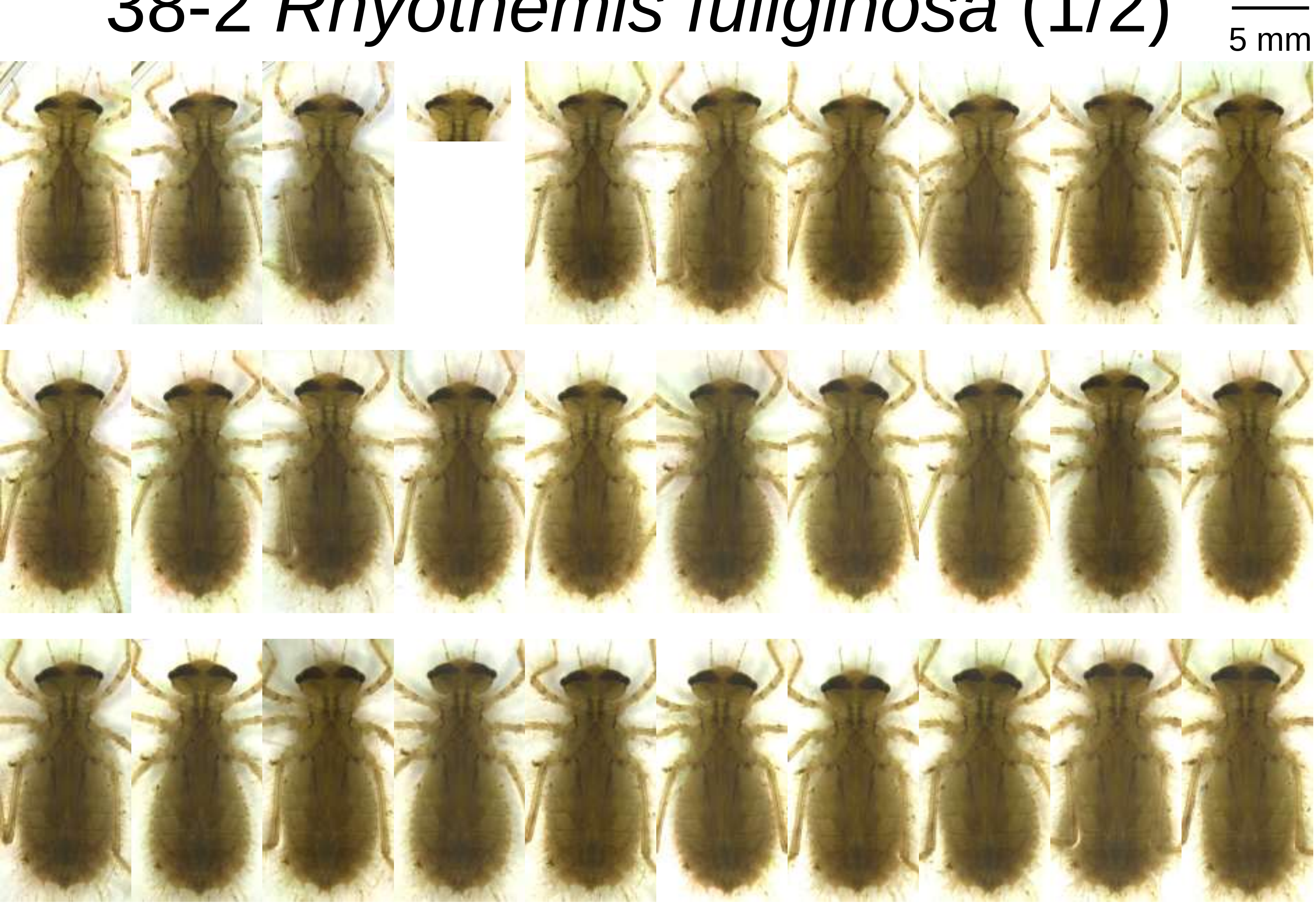

38-2 *Rhyothemis fuliginosa* (2/2)

5 mm

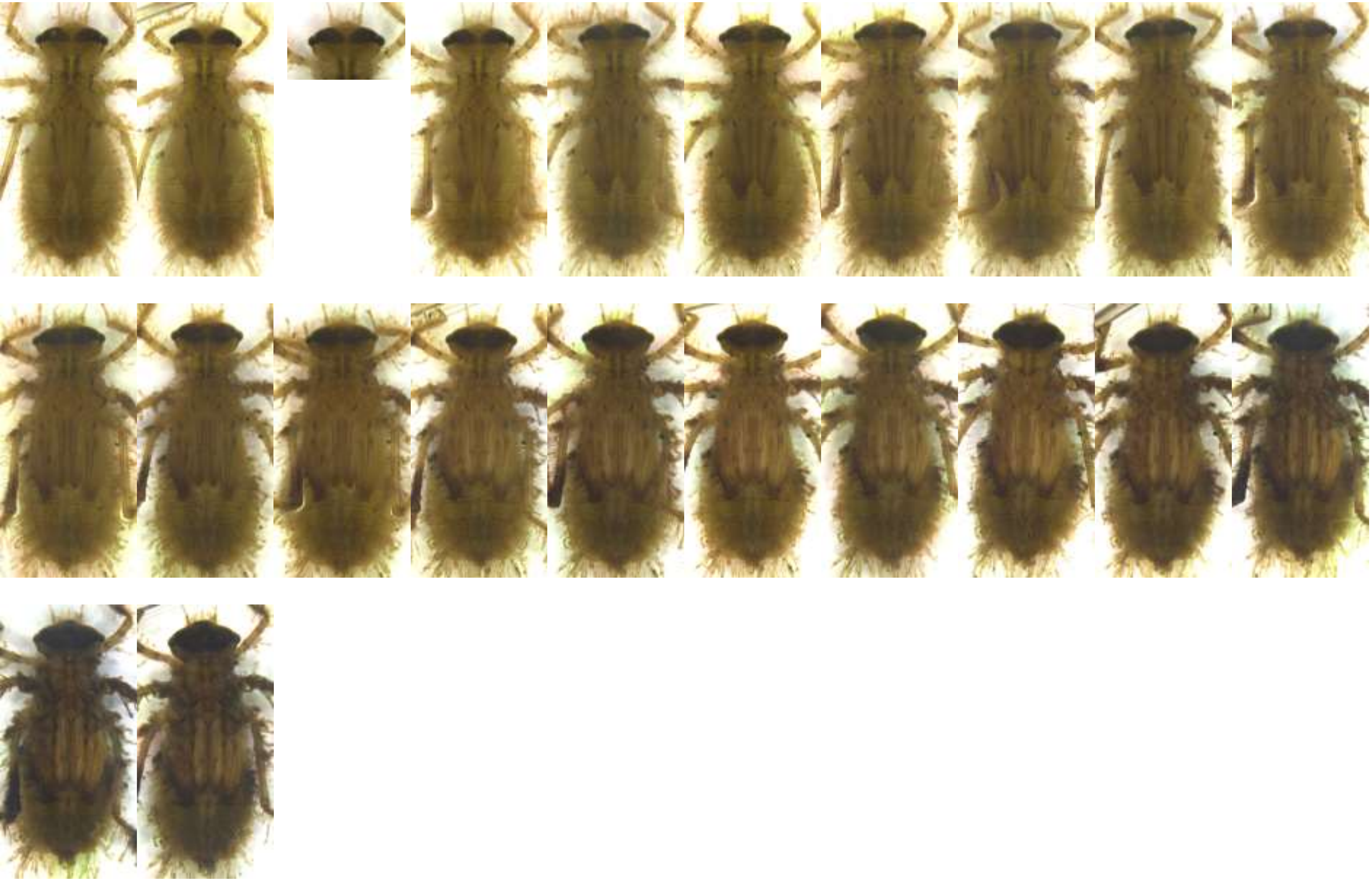

38-3 *Rhyothemis fuliginosa* (1/2)

5 mm

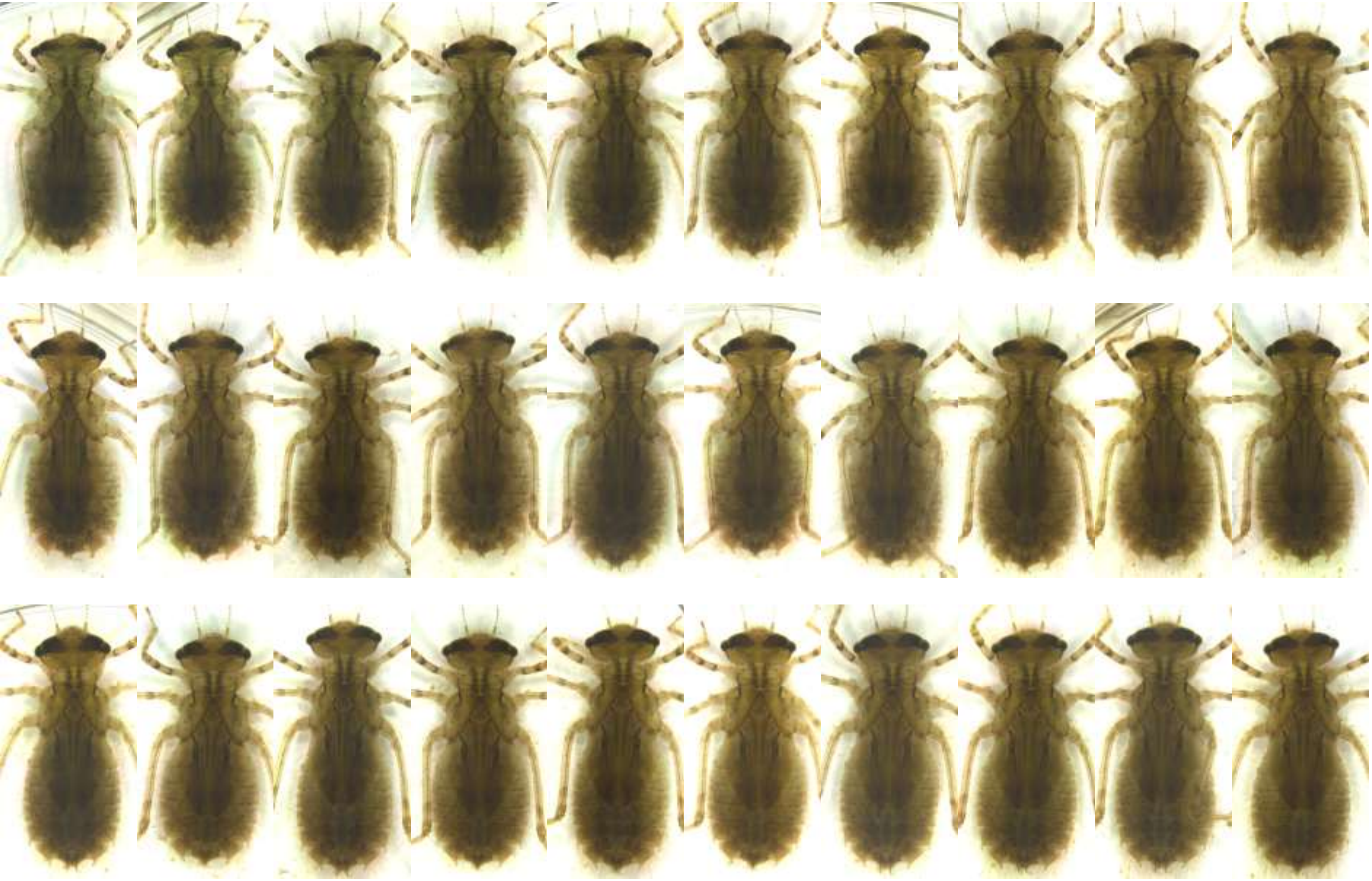

38-3 *Rhyothemis fuliginosa* (2/2)

5 mm

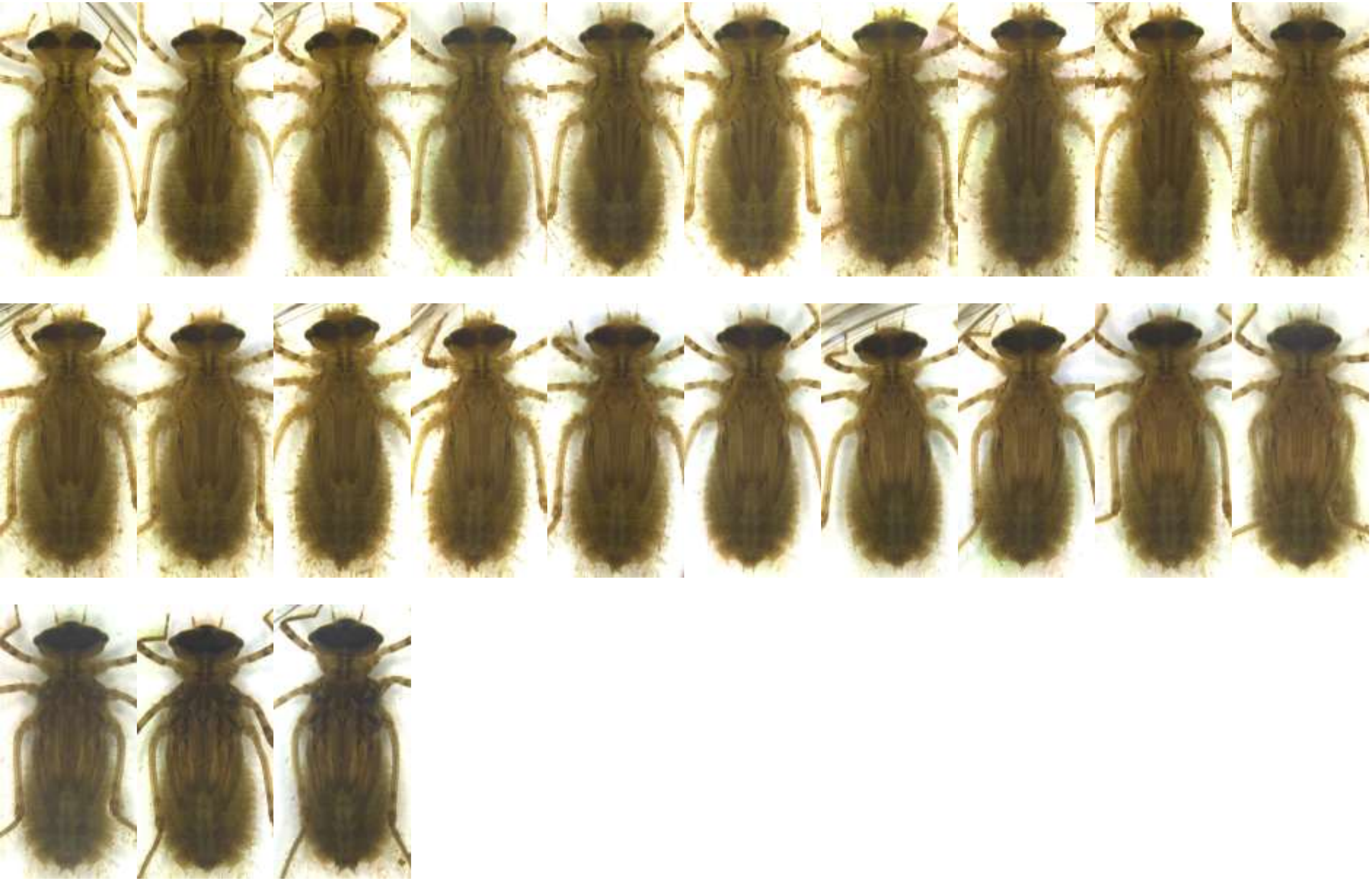

38-4 *Rhyothemis fuliginosa* (1/2)

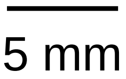

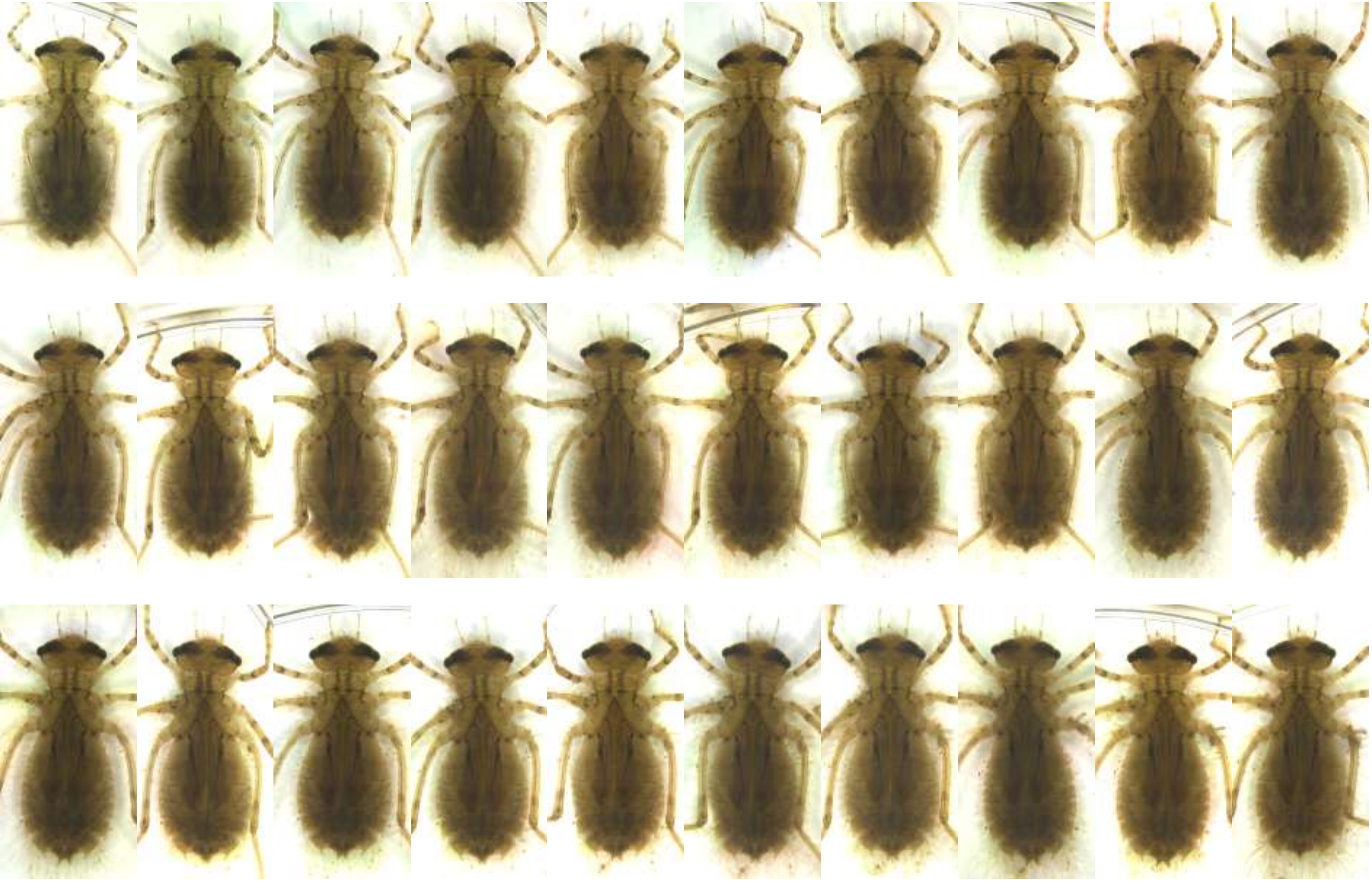

38-4 *Rhyothemis fuliginosa* (2/2)

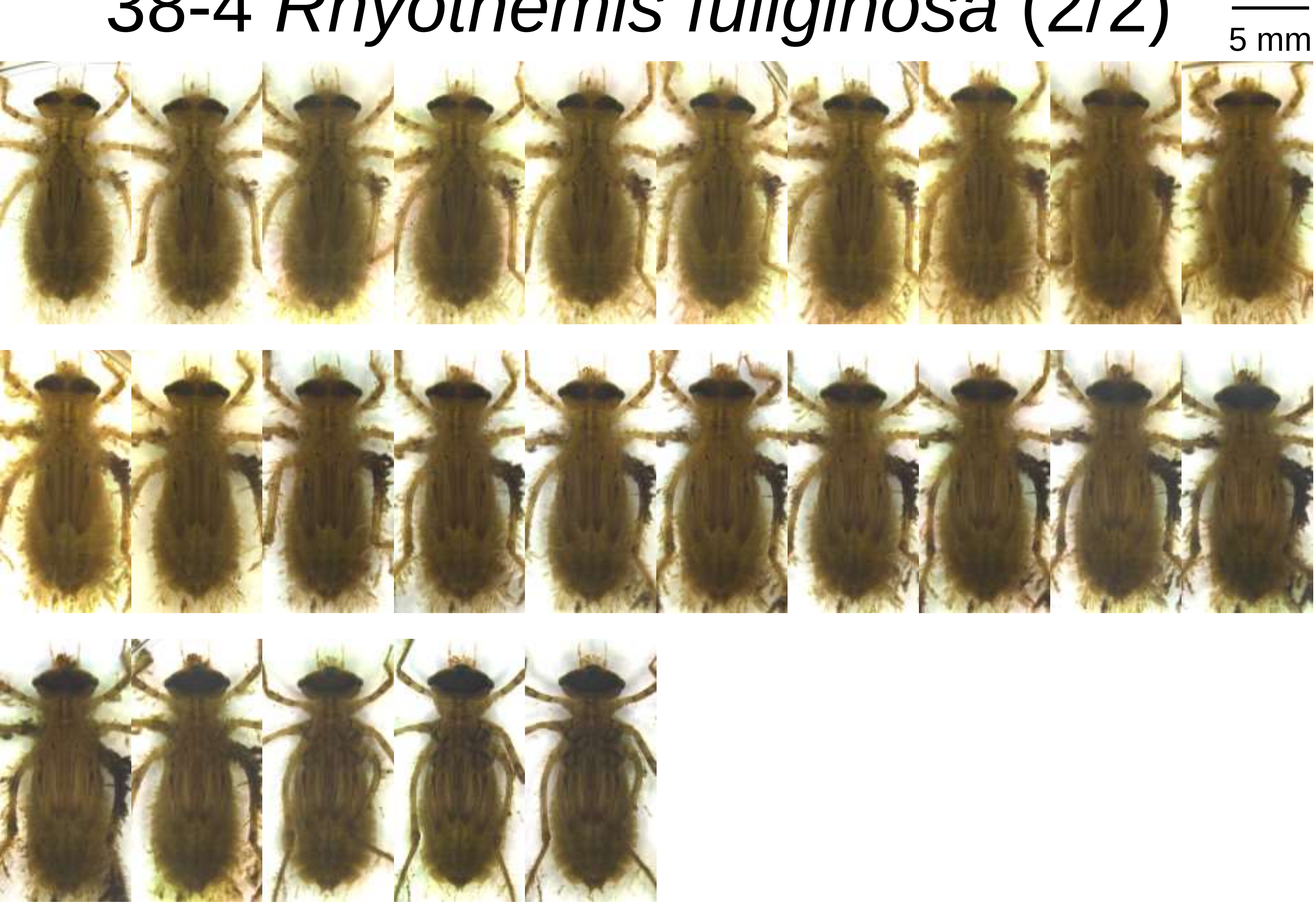

38-5 *Rhyothemis fuliginosa* (1/2)

5 mm

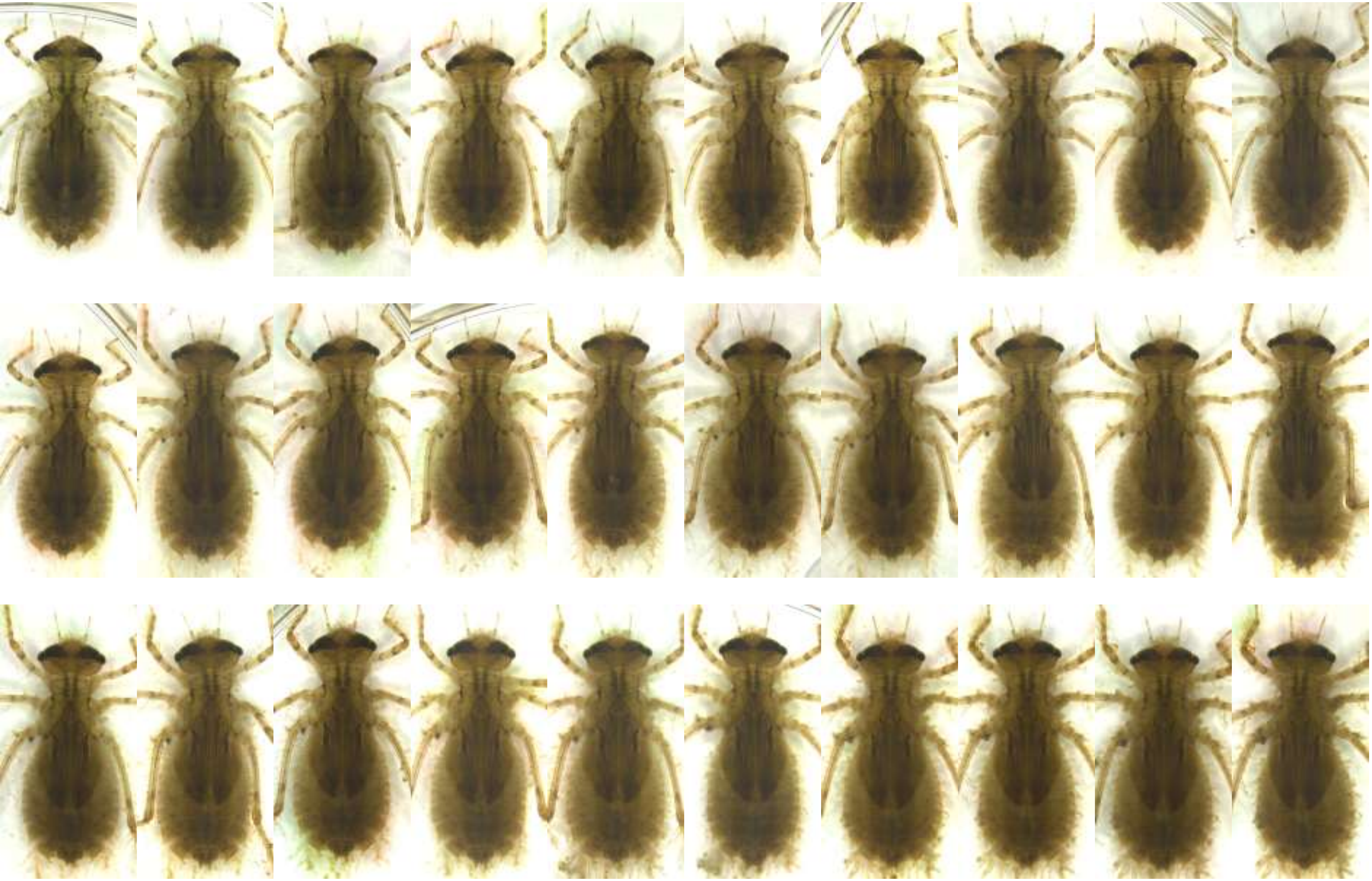

38-5 *Rhyothemis fuliginosa* (2/2)

5 mm

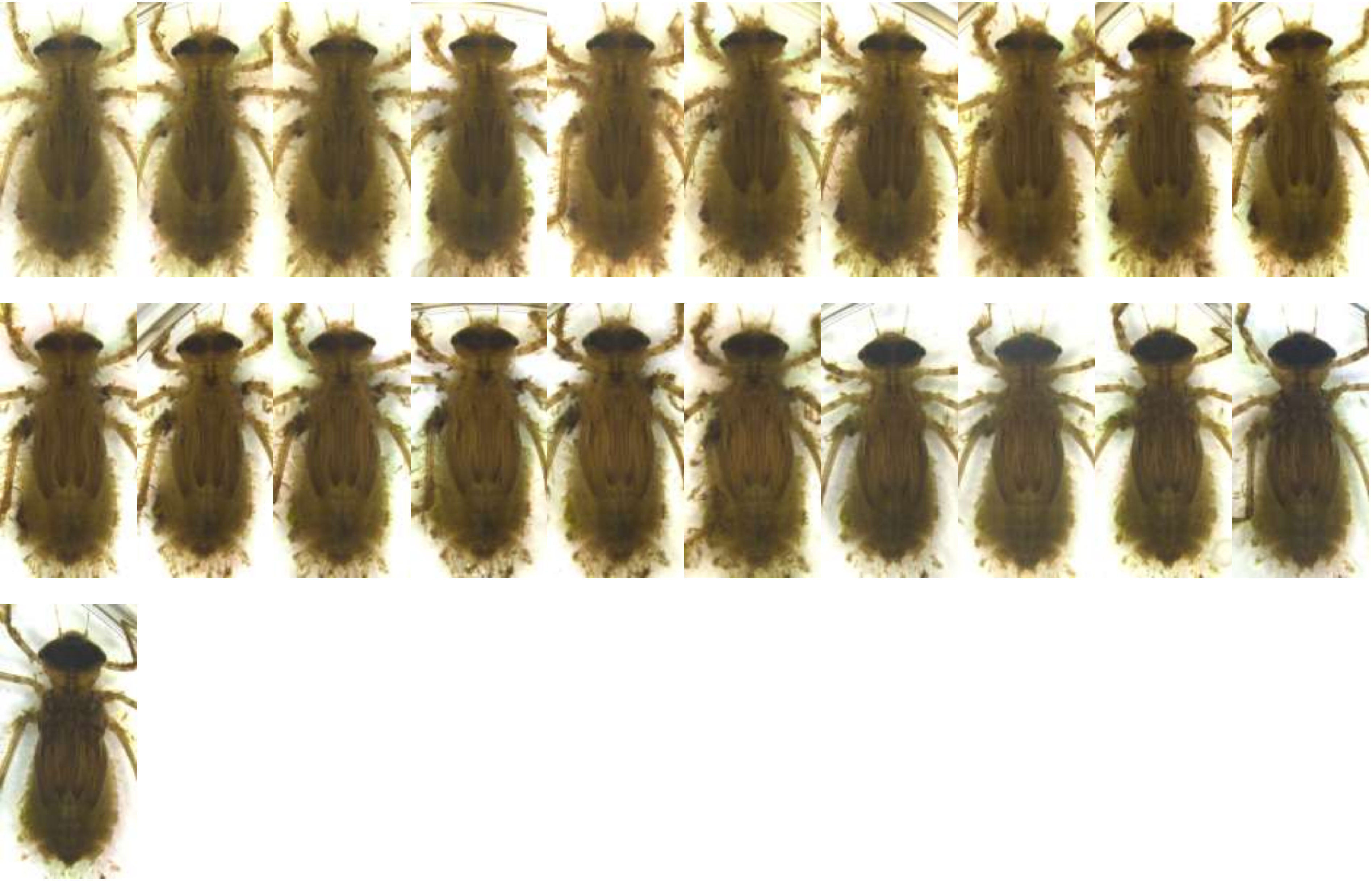

38-6 *Rhyothemis fuliginosa* (1/3)

5 mm

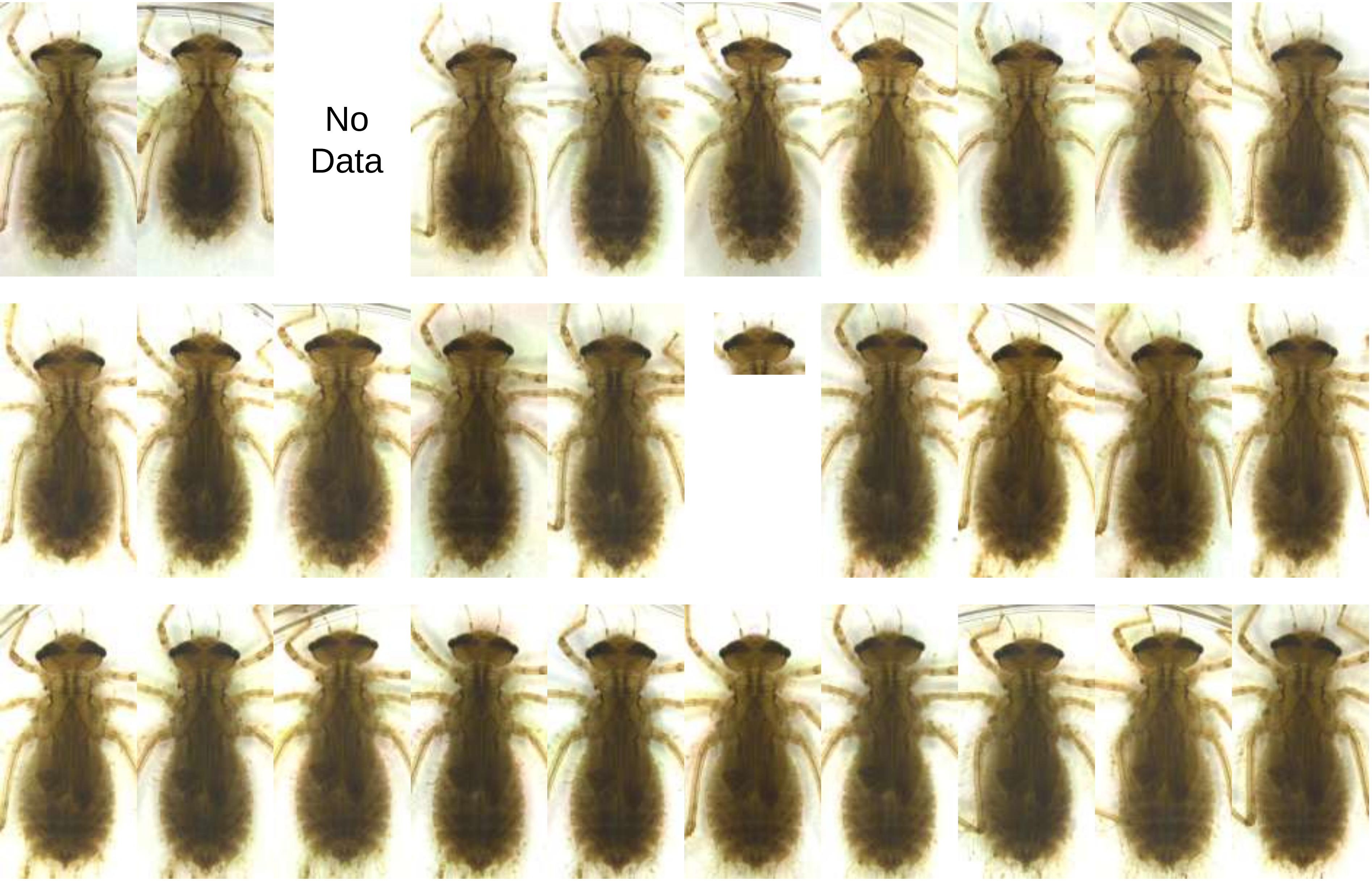

38-6 *Rhyothemis fuliginosa* (2/3)

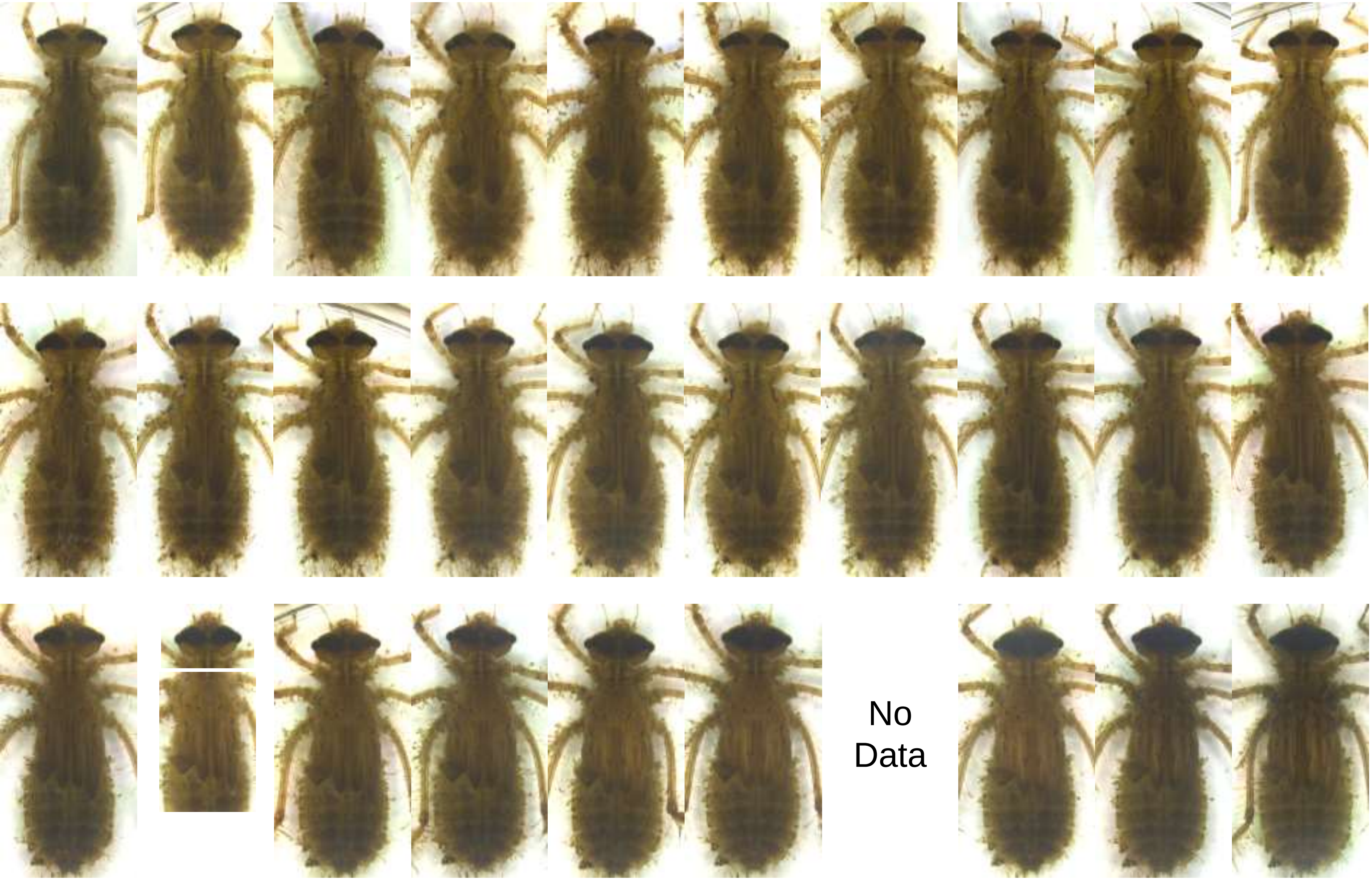

33-6 *Rhyothemis fuliginosa* (2/3)

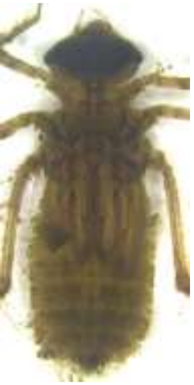

38-7 *Rhyothemis fuliginosa* (1/3)

5 mm

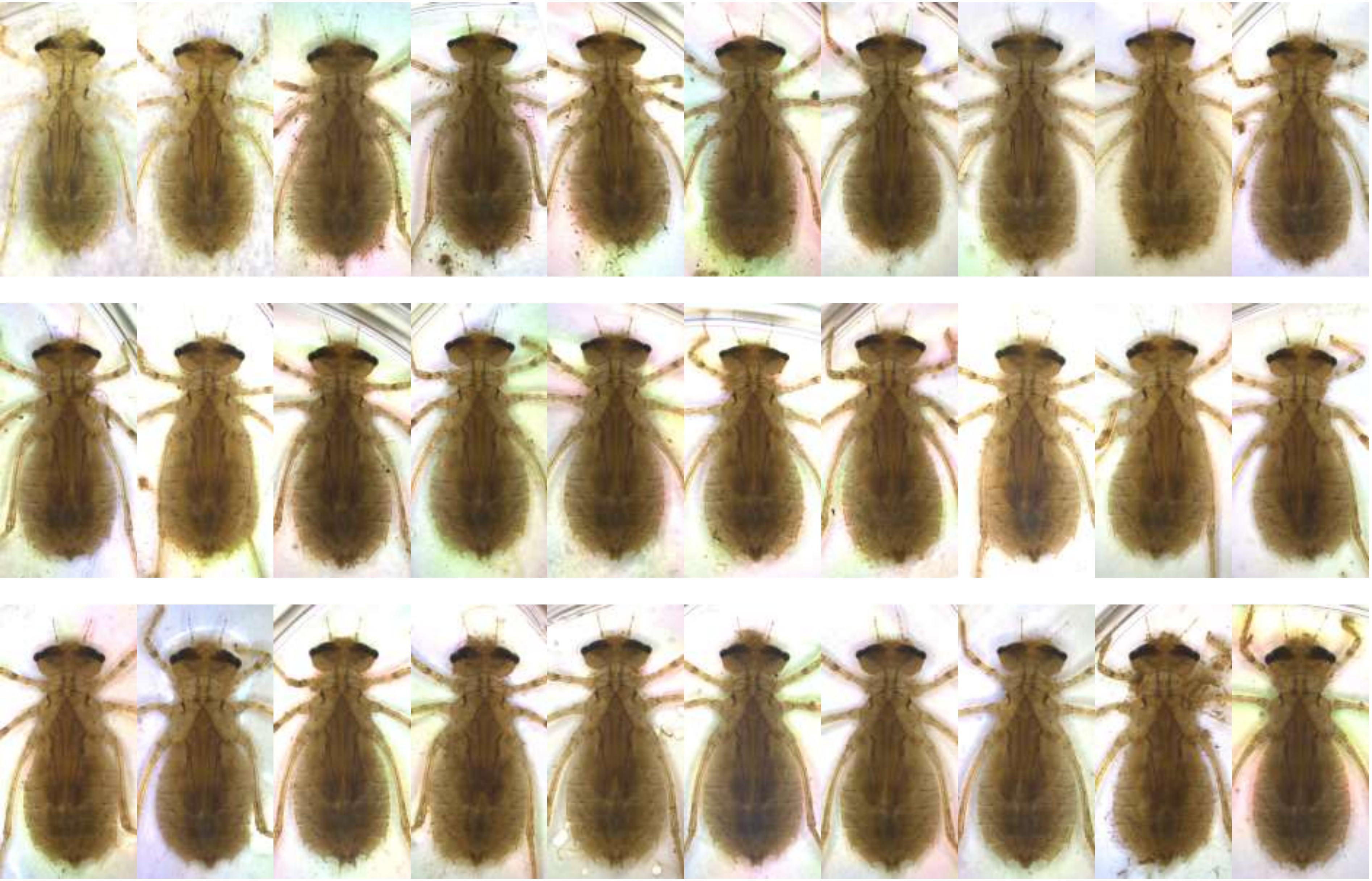

38-7 *Rhyothemis fuliginosa* (2/3)

5 mm

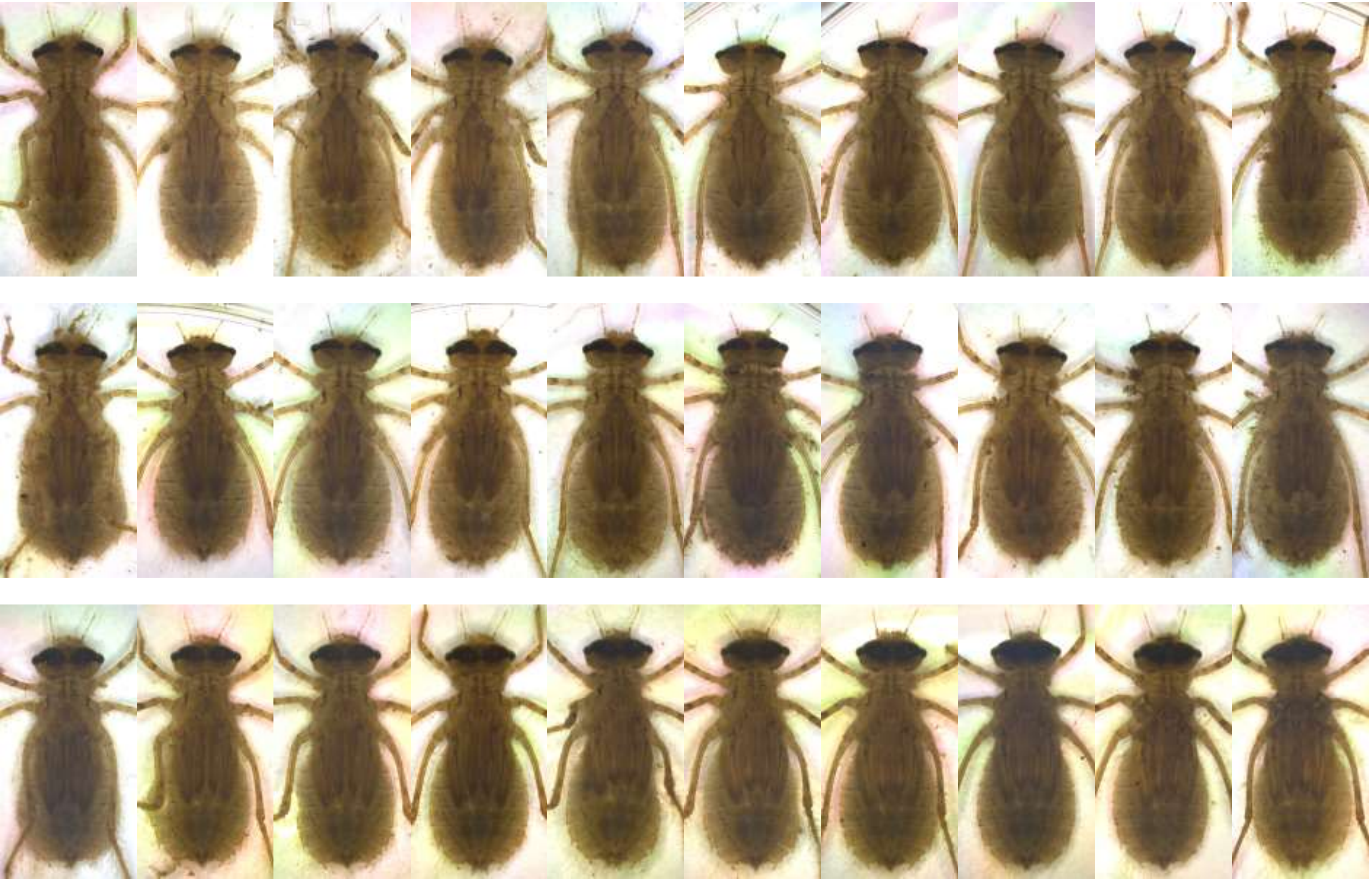

38-7 *Rhyothemis fuliginosa* (3/3)

5 mm

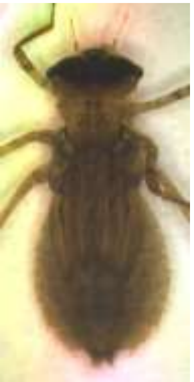

39-1 *Sympetrum darwinianum* (1/1)

5 mm

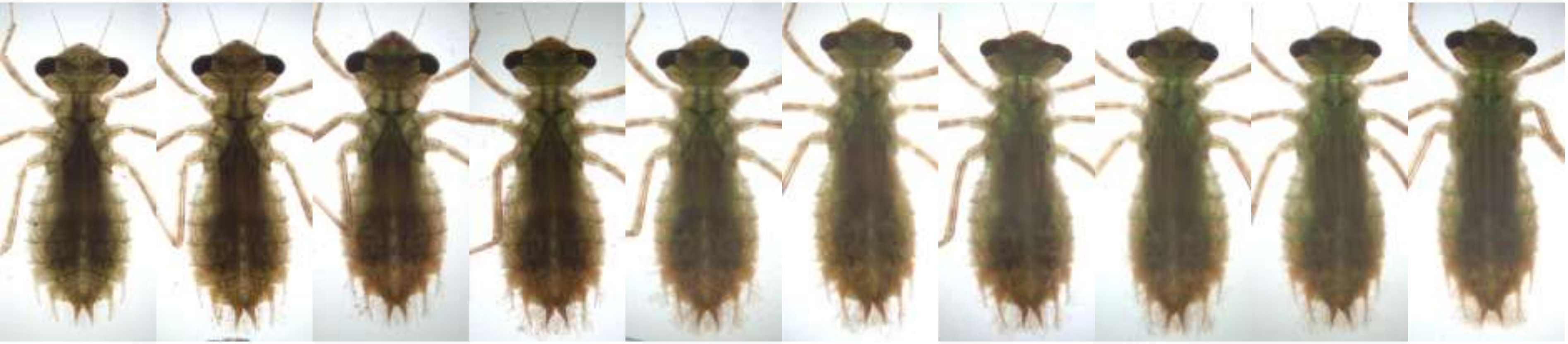

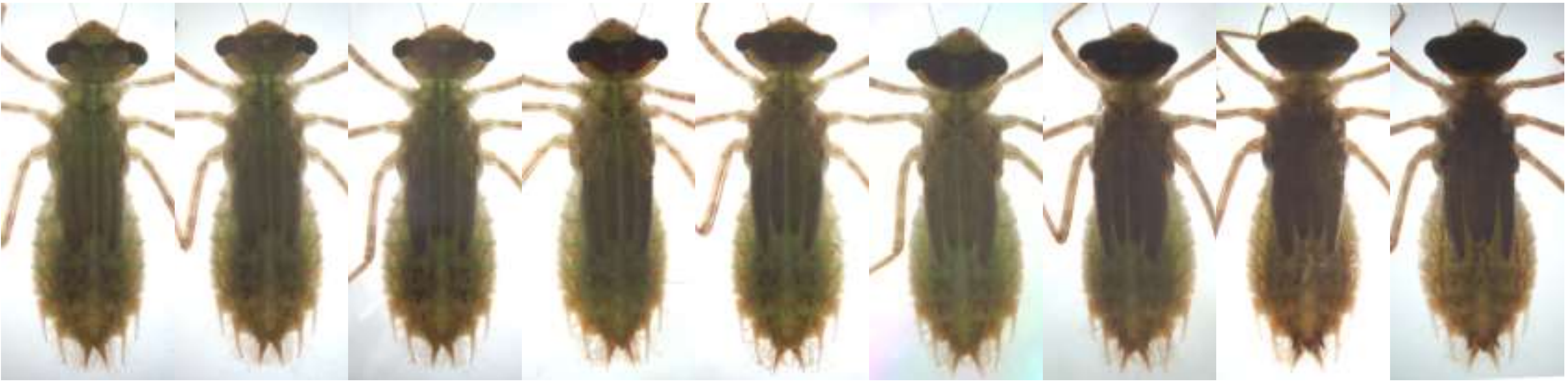

39-2 *Sympetrum darwinianum* (1/1)

5 mm

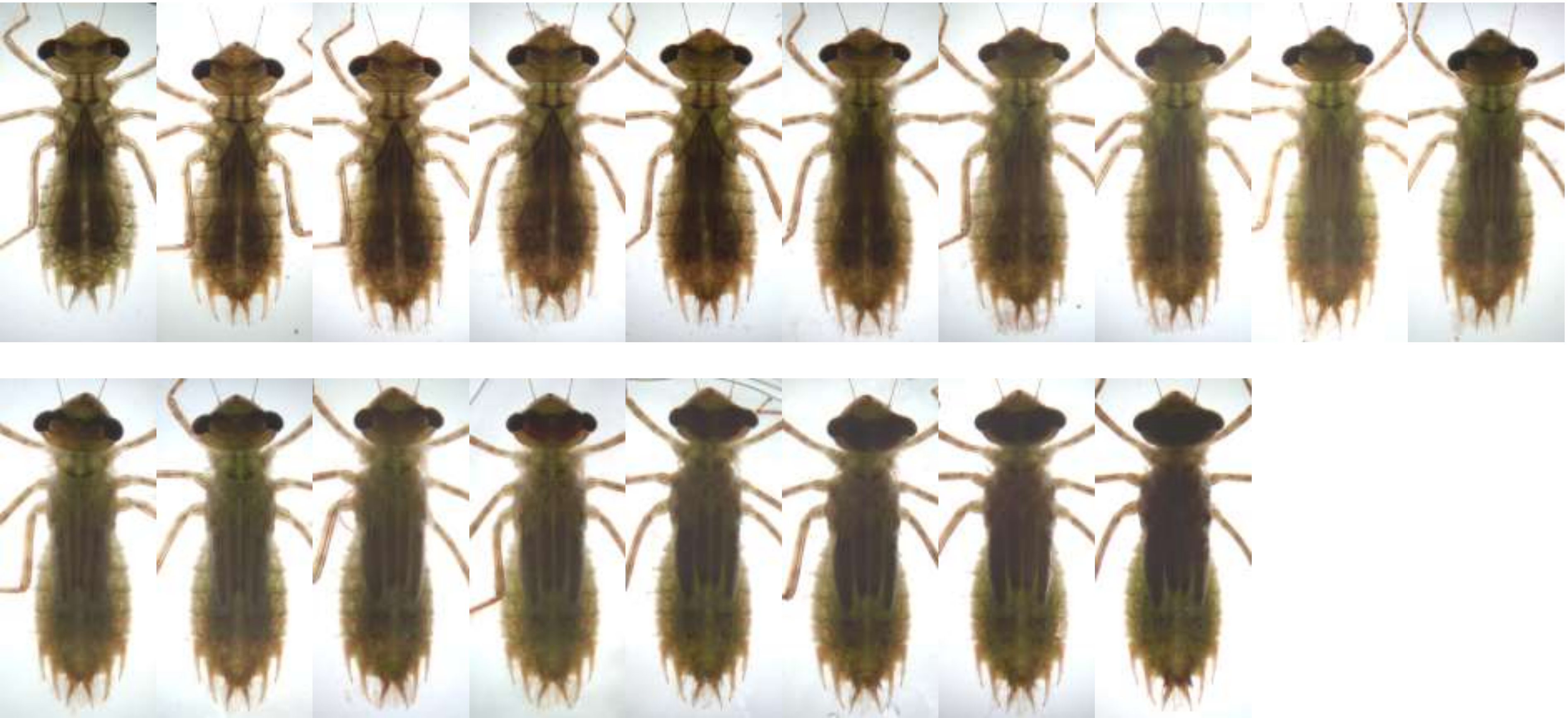

39-3 *Sympetrum darwinianum* (1/1)

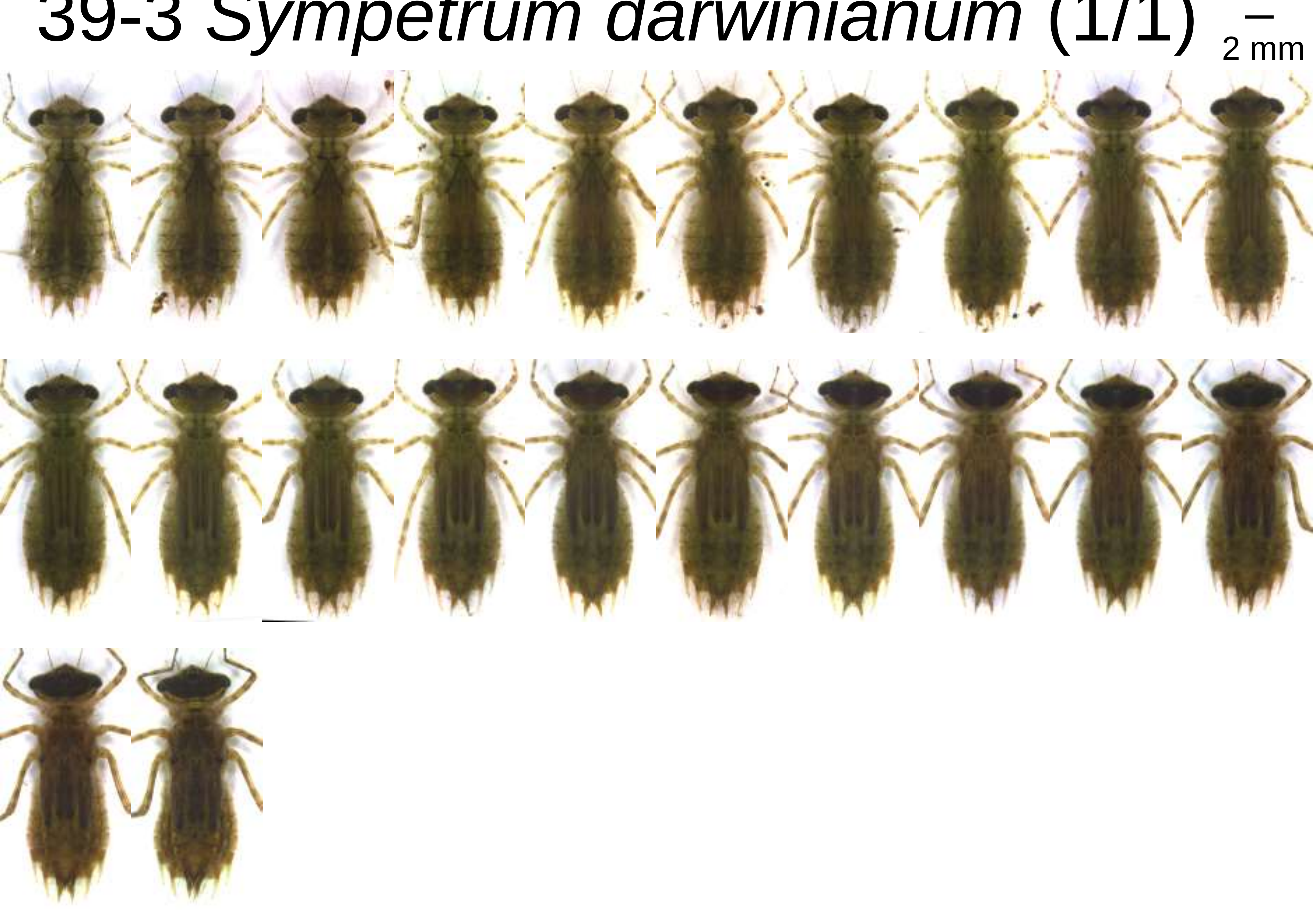

40-1 *Sympetrum maculatum* (1/1)

2 mm

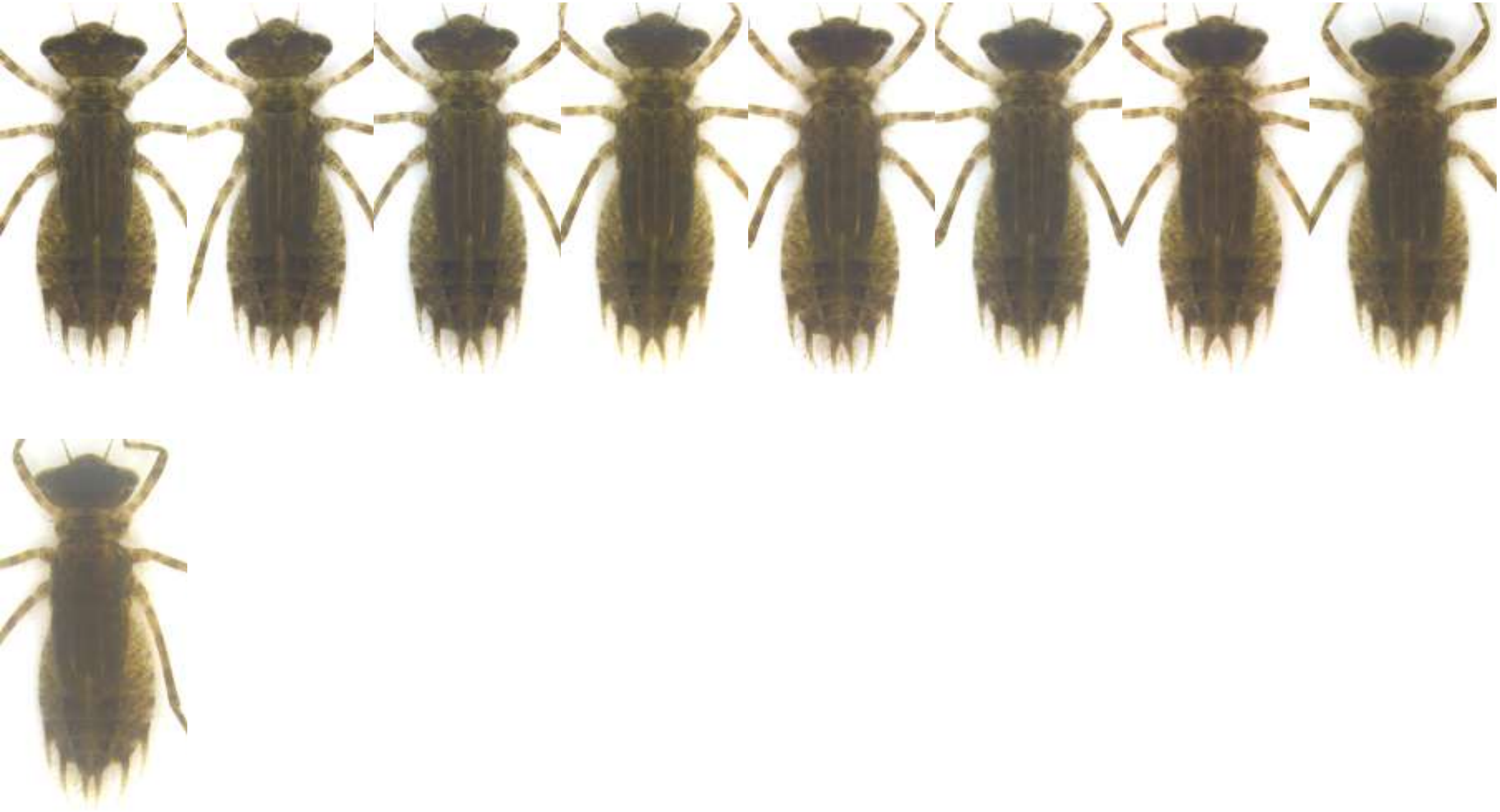

40-2 *Sympetrum maculatum* (1/1)

2 mm

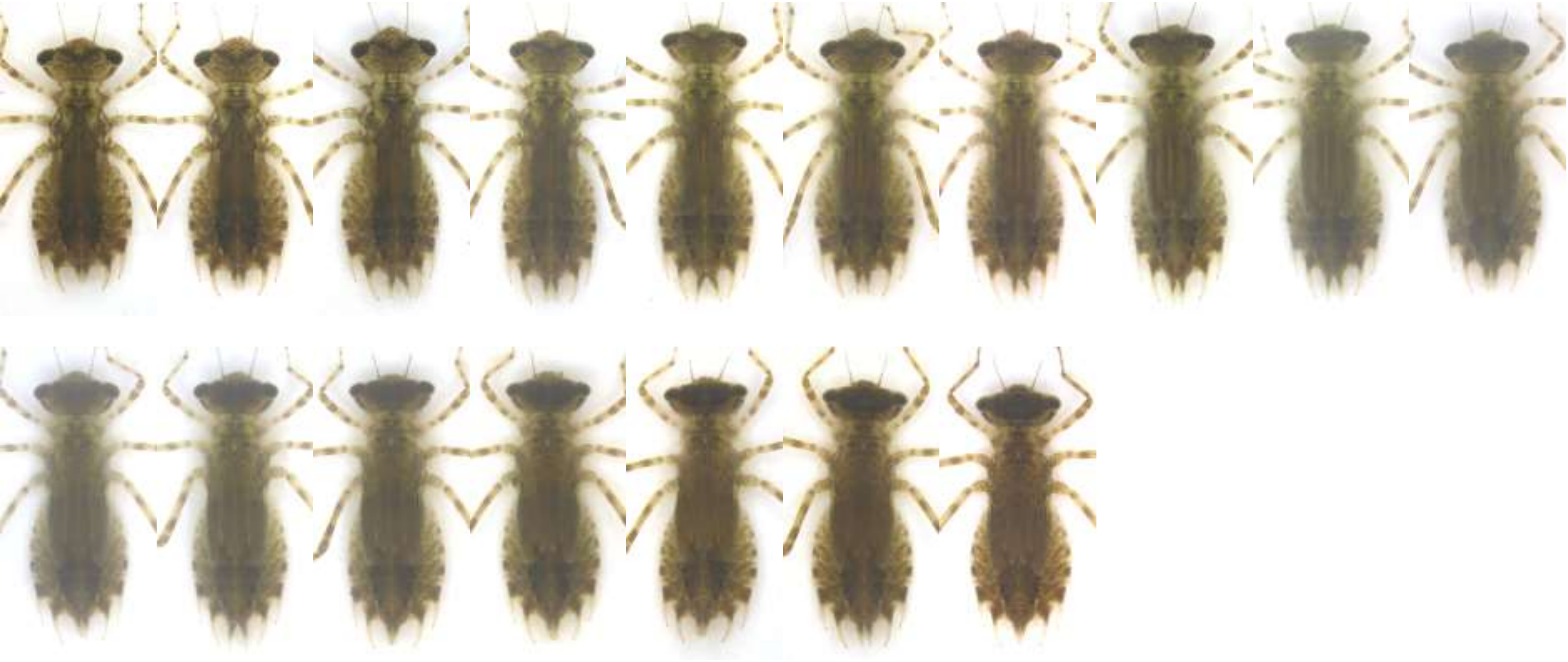

40-3 *Sympetrum maculatum* (1/1)

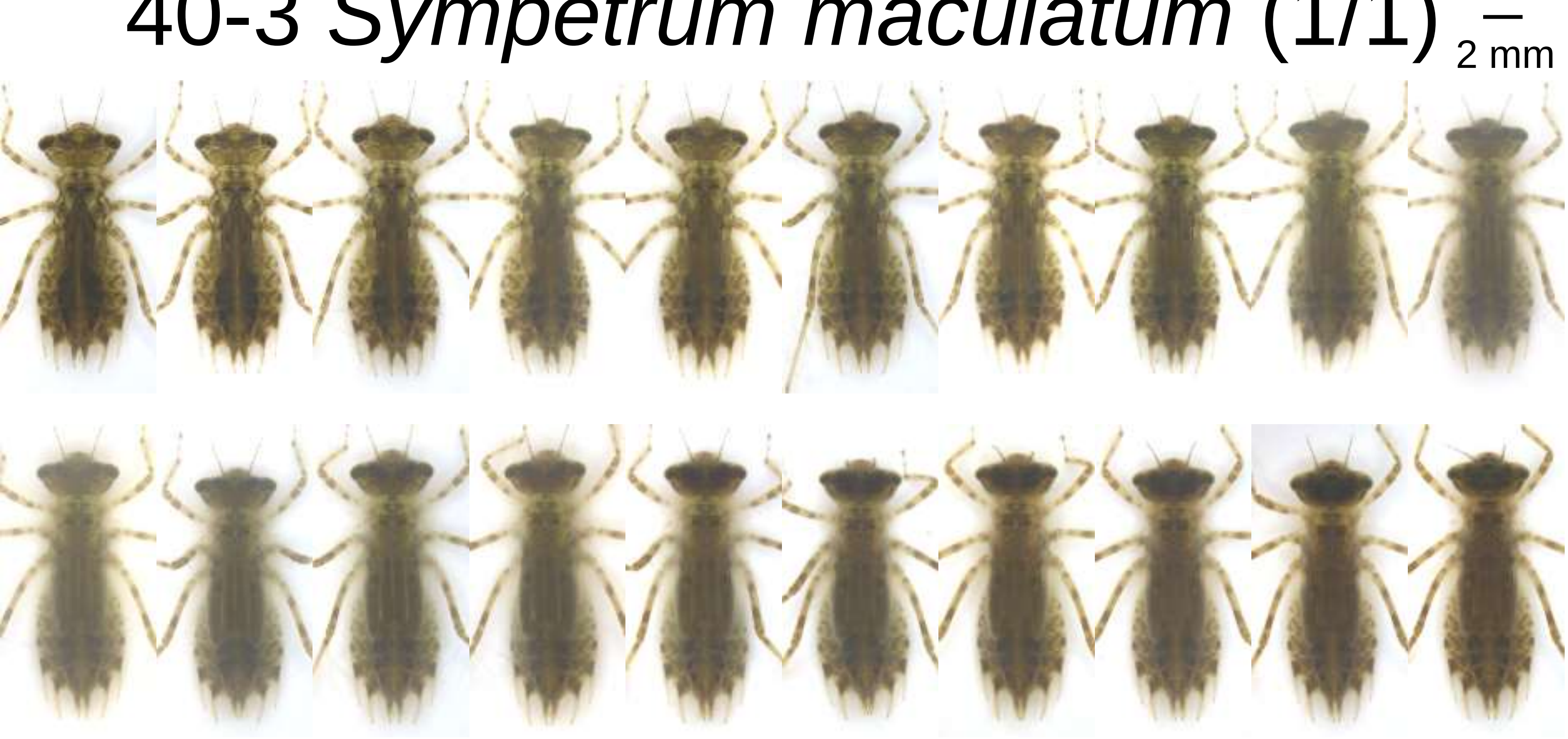

40-4 *Sympetrum maculatum* (1/1)

2 mm

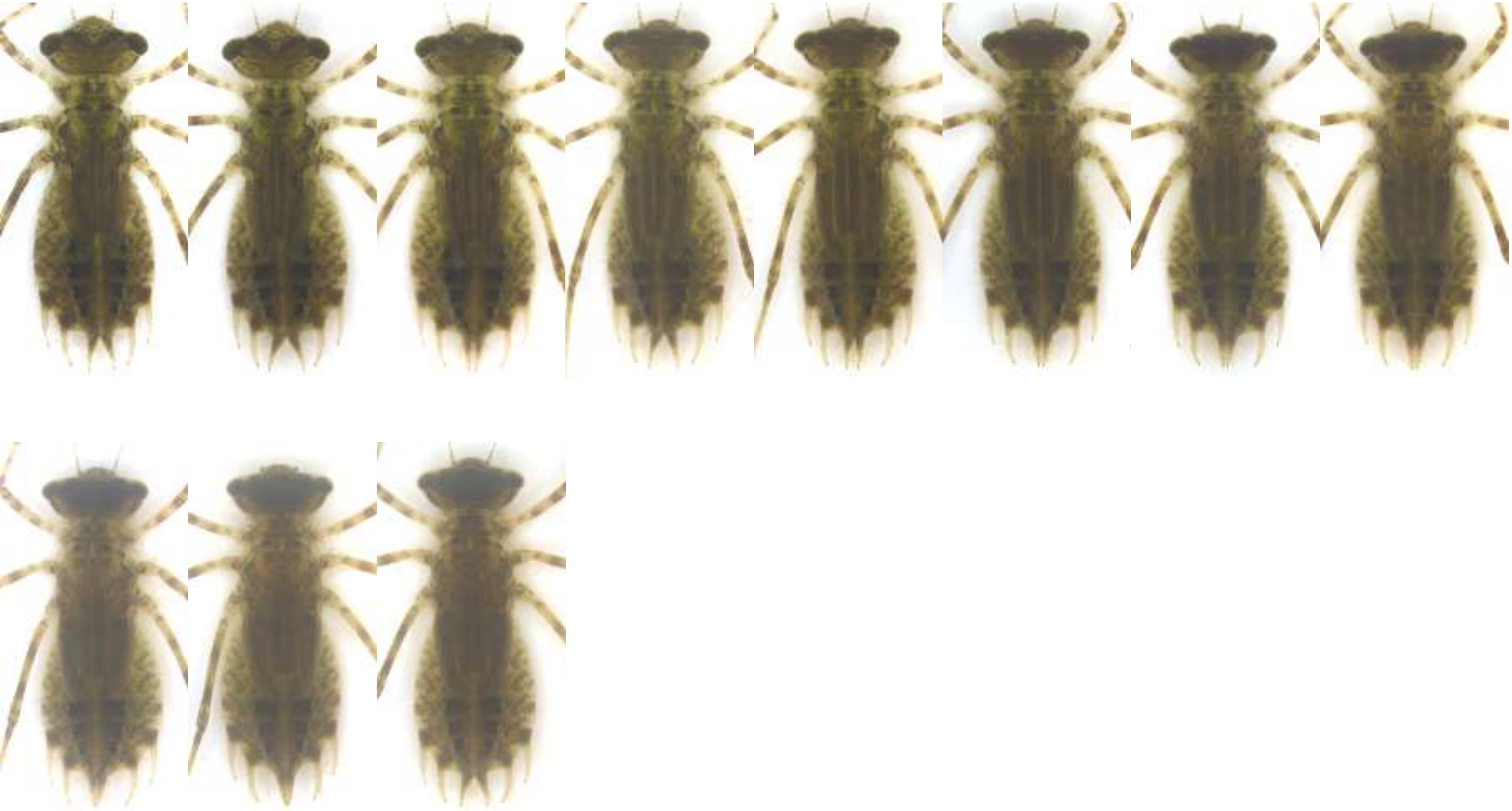

41-1 *Sympetrum infuscatum* (1/1)

5 mm

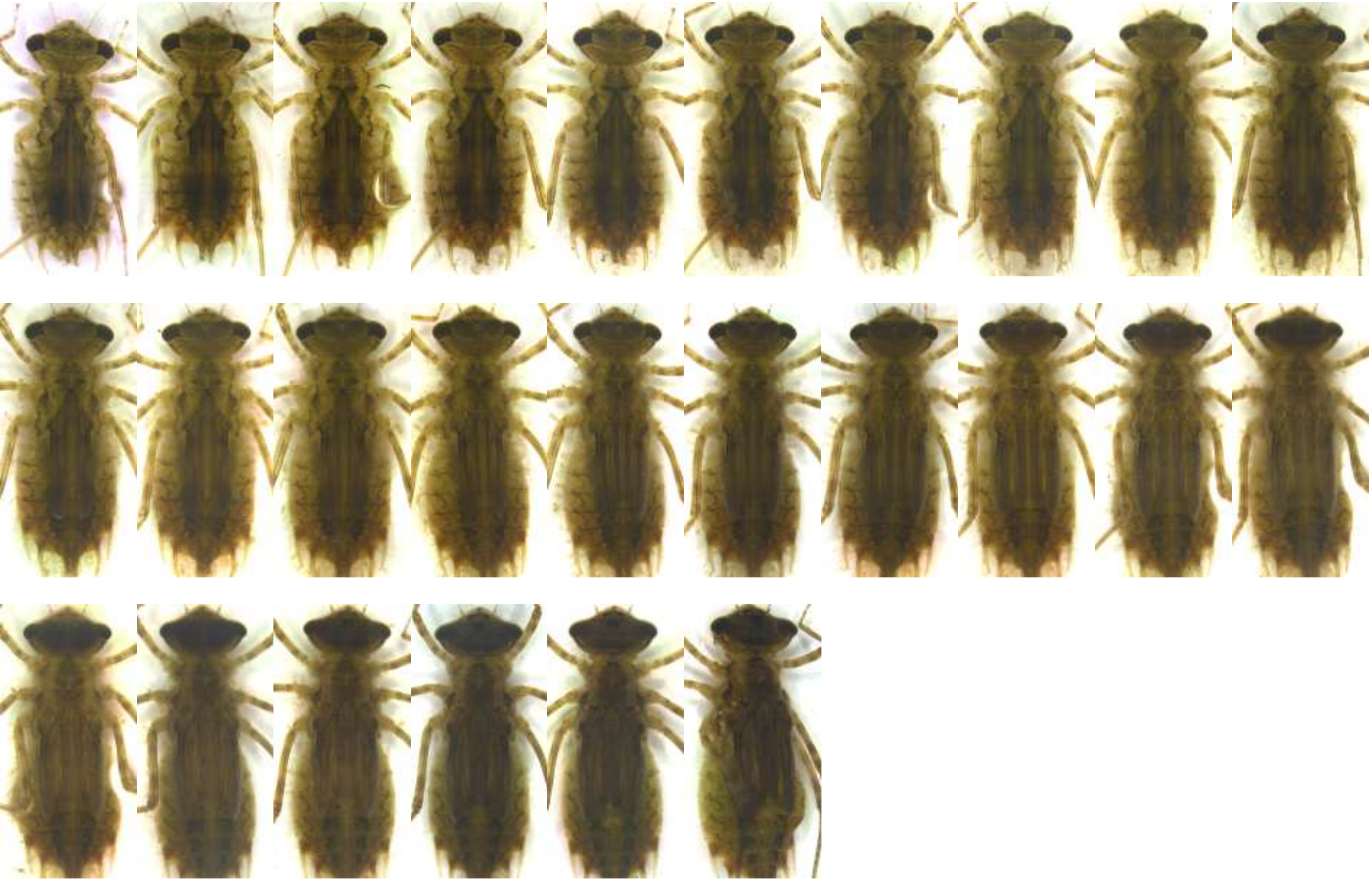

41-2 *Sympetrum infuscatum* (1/1)

5 mm

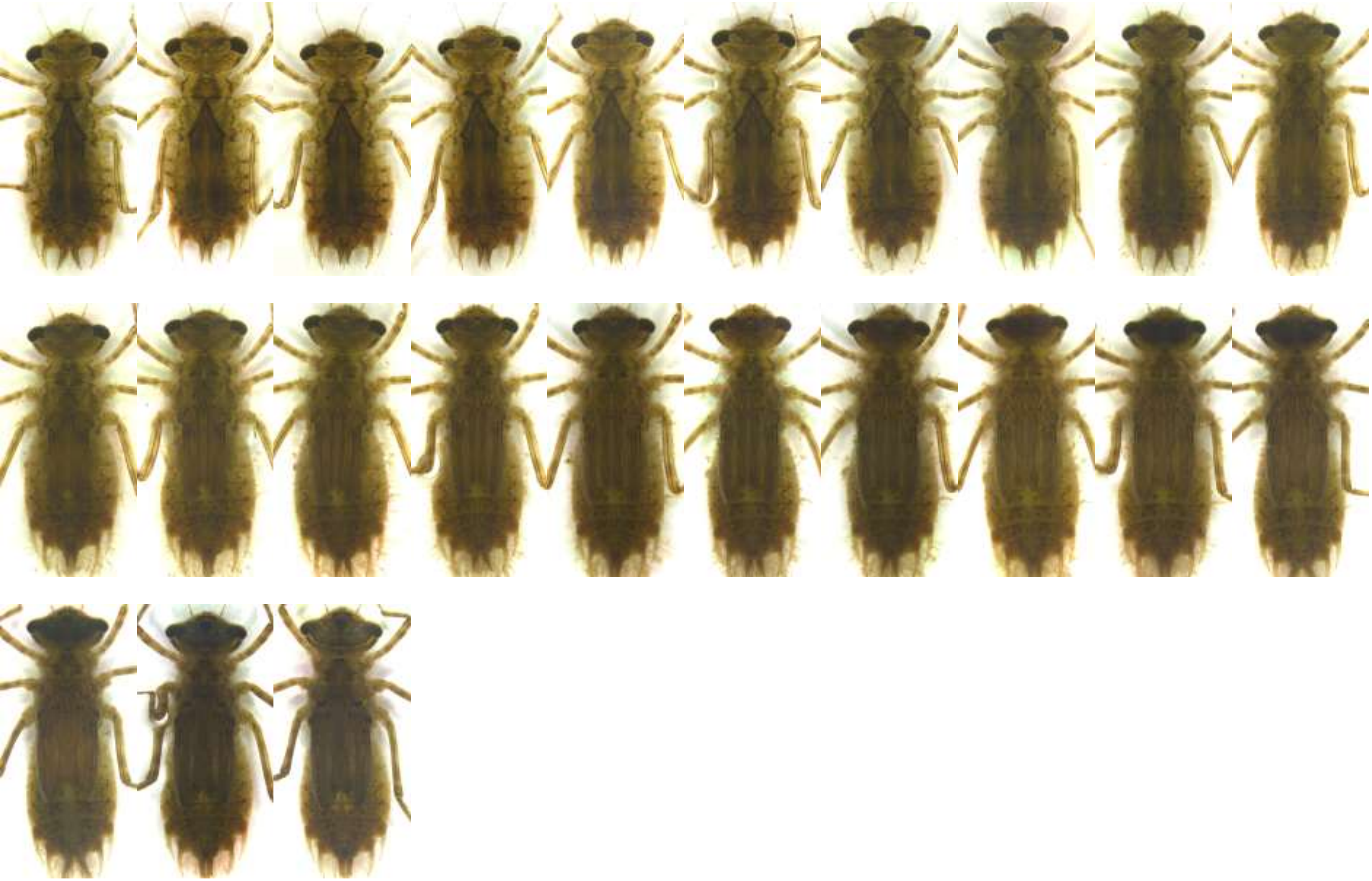

41-3 *Sympetrum infuscatum* (1/1)

5 mm

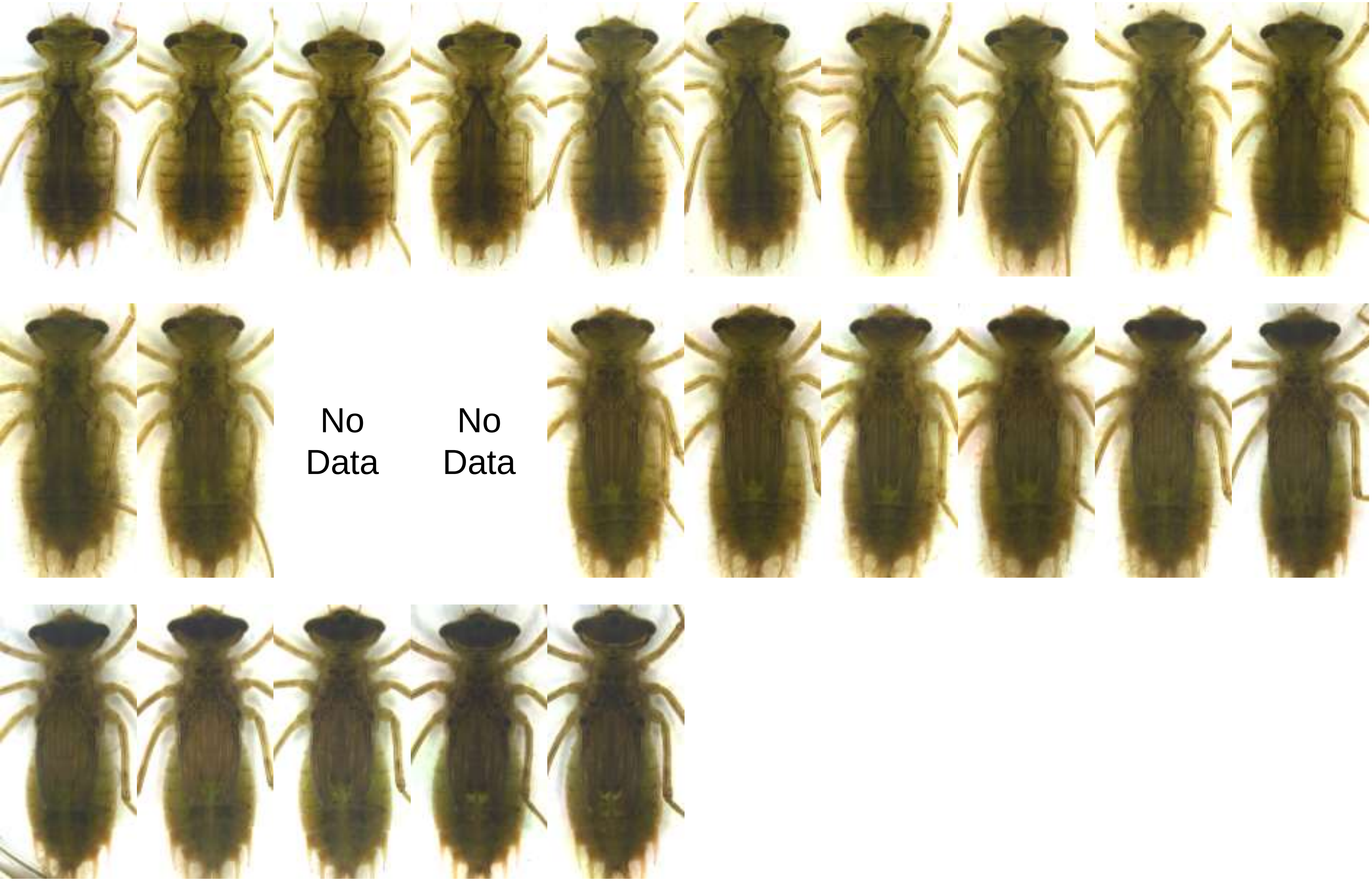

42-1 *Sympetrum frequens* (1/1)

5 mm

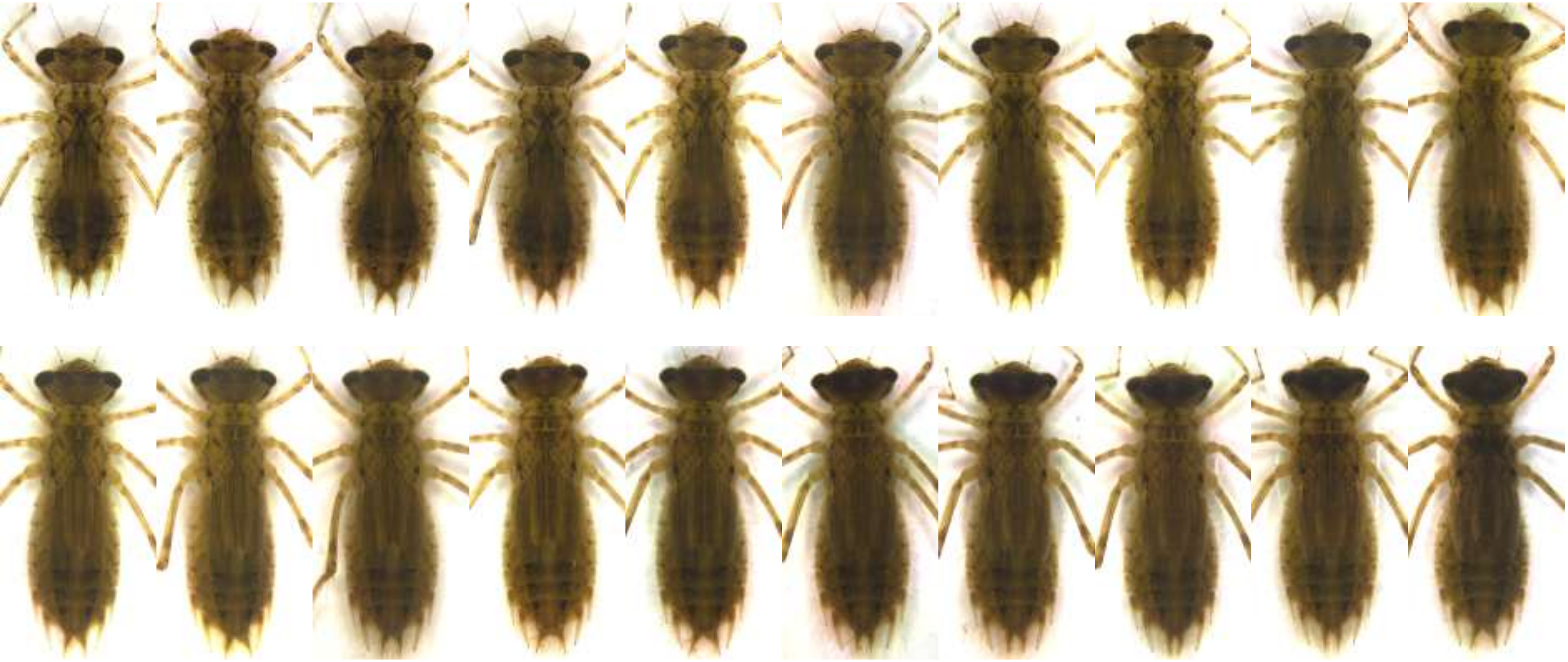

43-1 *Sympetrum kunckeli* (1/1)

5 mm

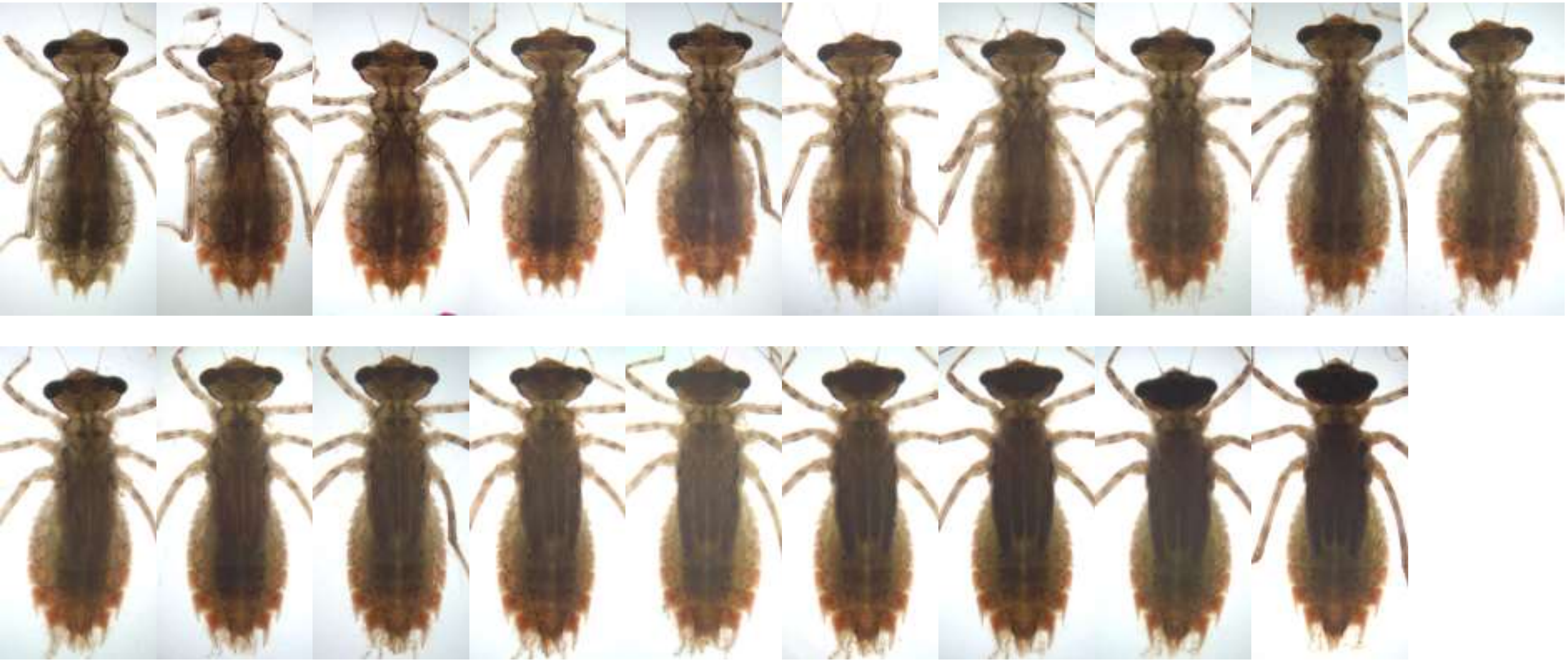

43-2 *Sympetrum kunckeli* (1/1)

5 mm

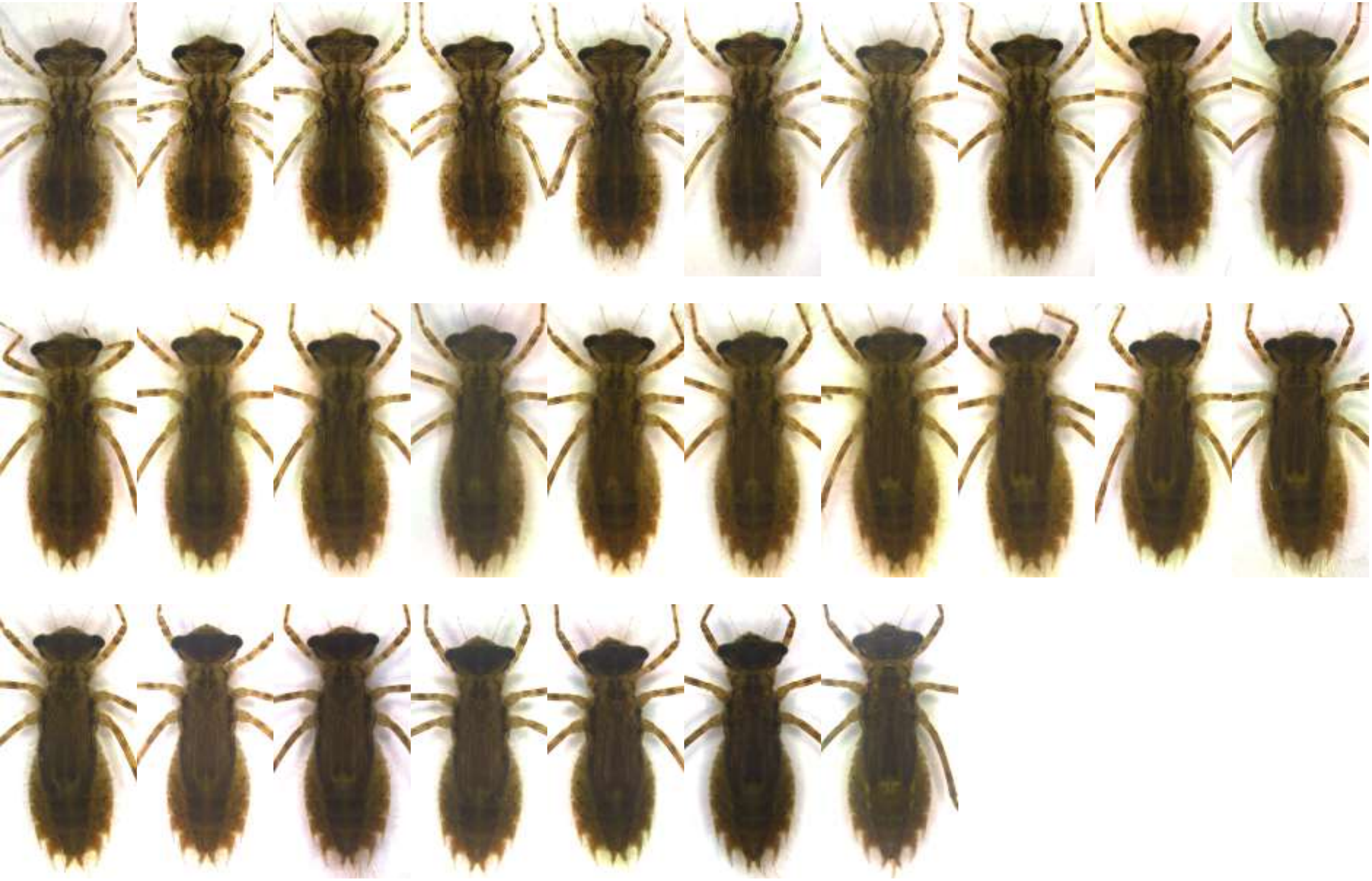

43-3 *Sympetrum kunckeli* (1/1)

5 mm

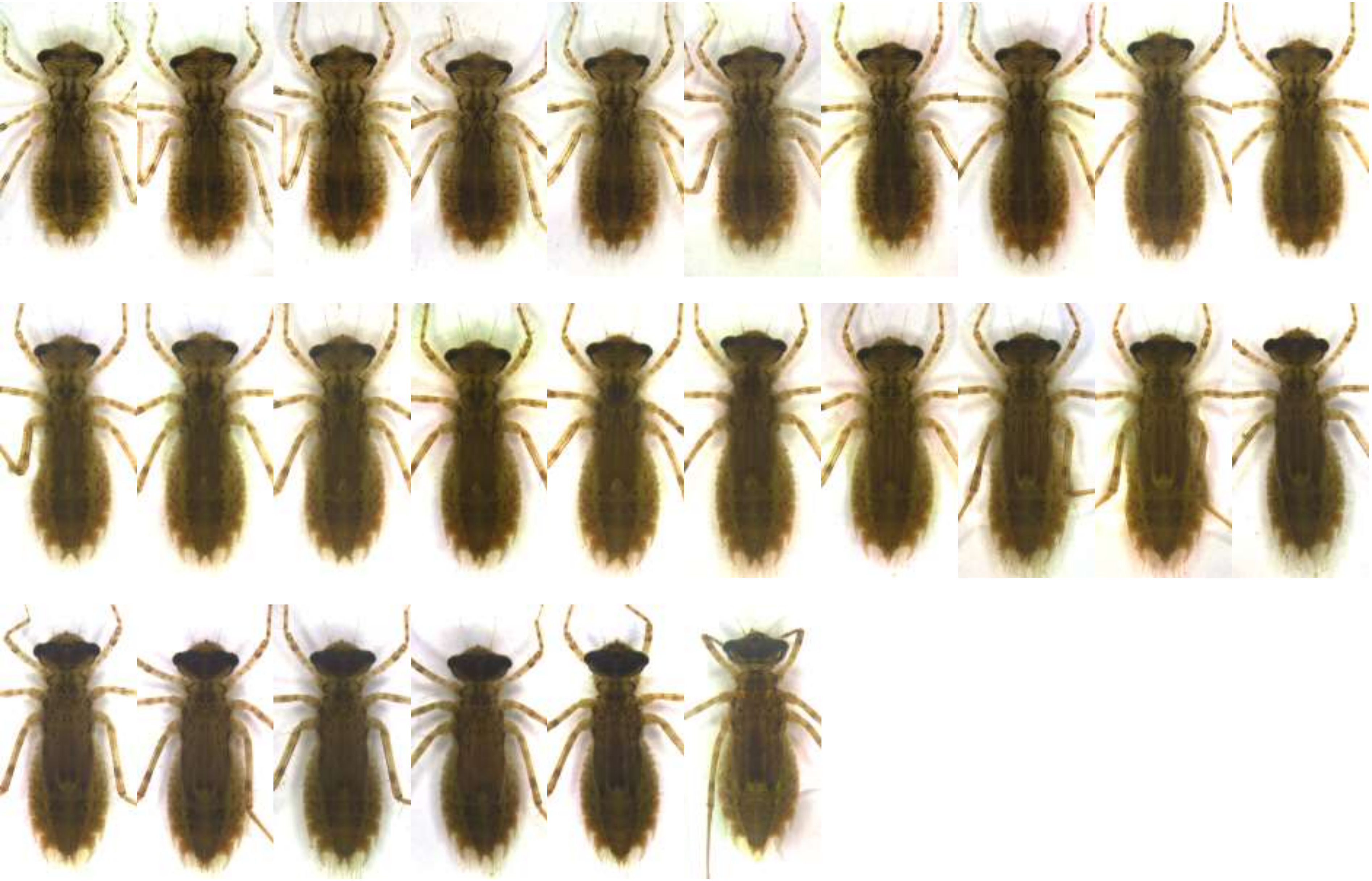

44-1 *Sympetrum uniforme* (1/1)

5 mm

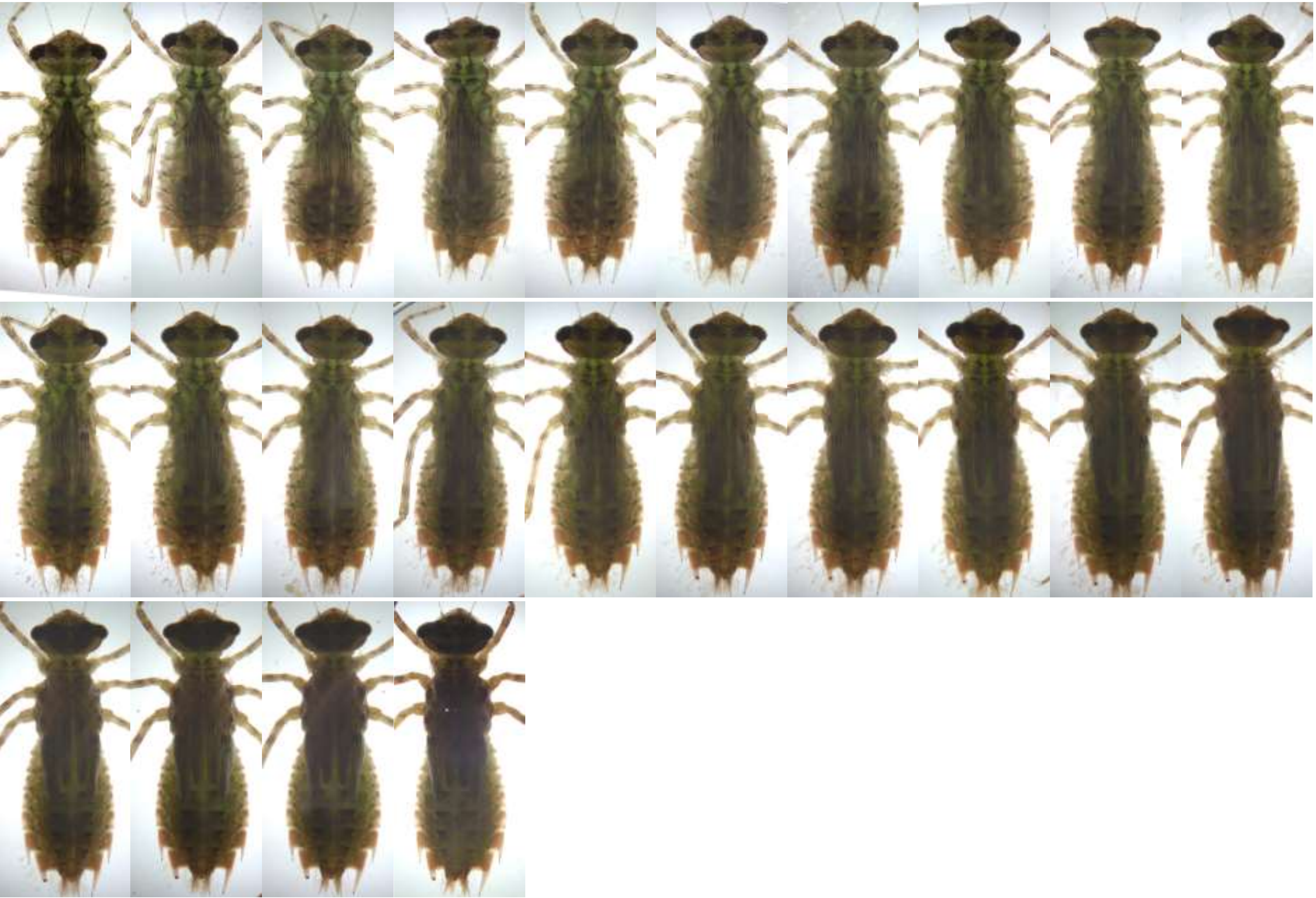

44-2 *Sympetrum uniforme* (1/1)

5 mm

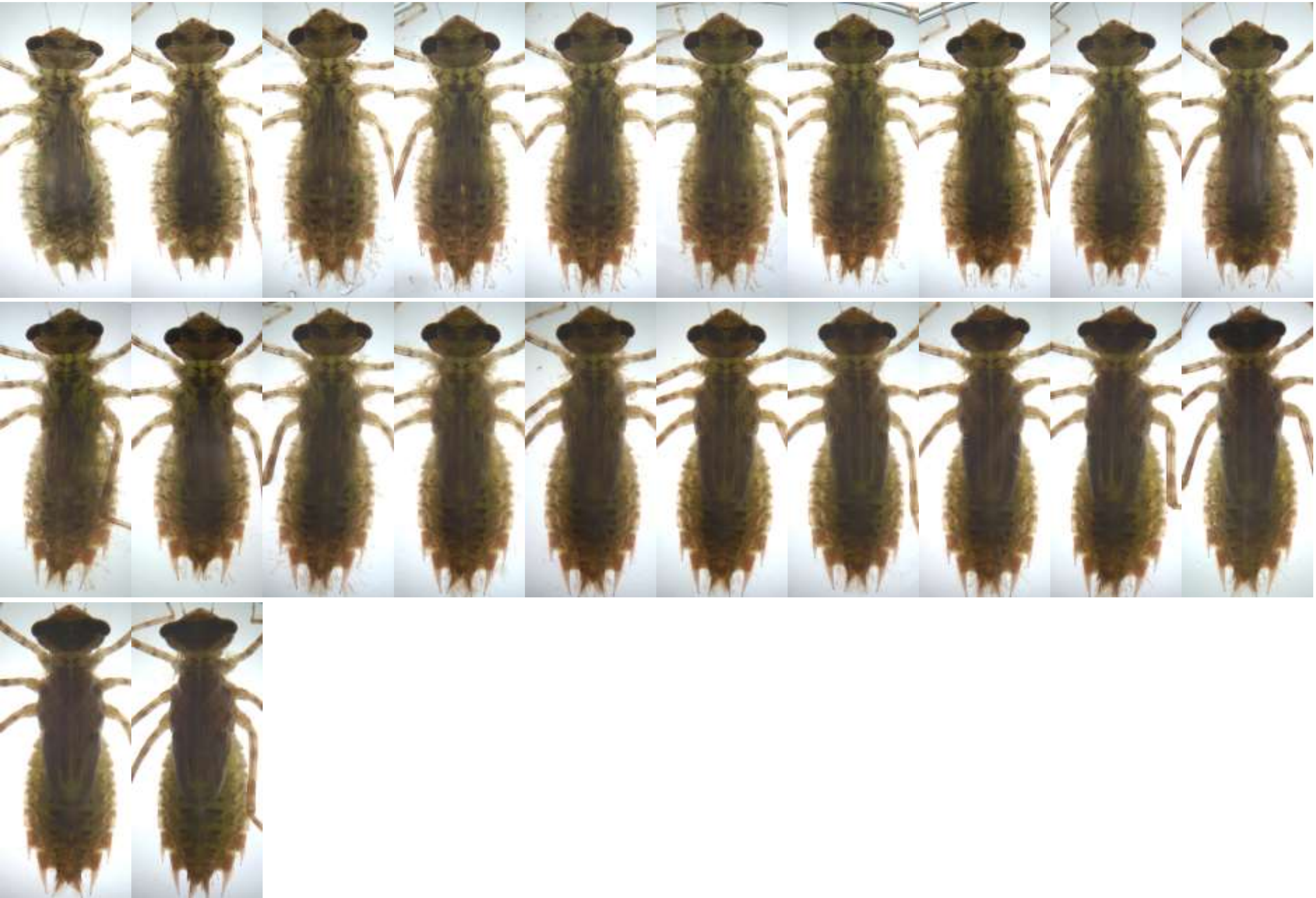

44-3 *Sympetrum uniforme* (1/1)

5 mm

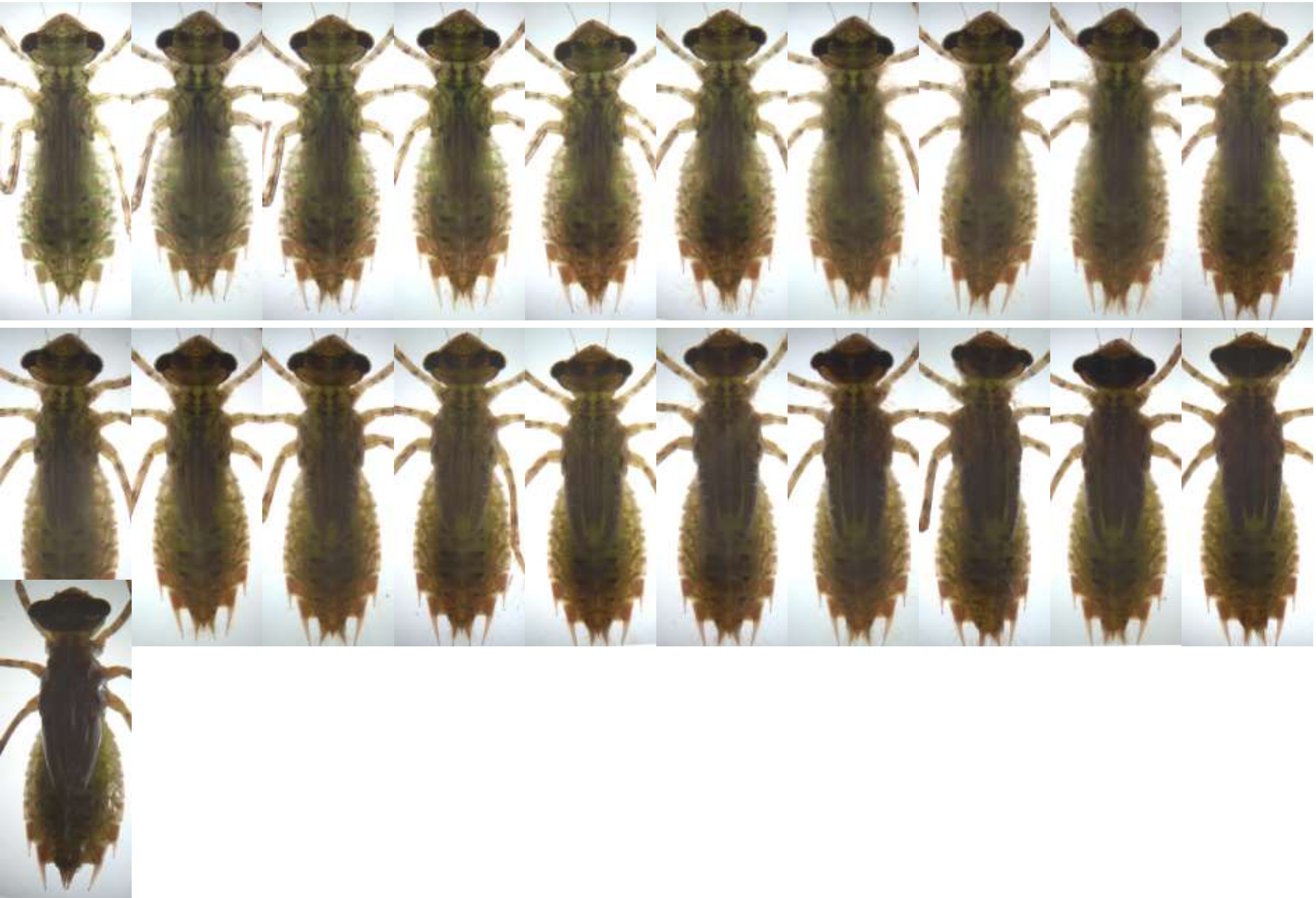

45-1 *Pseudothemis zonata* (1/2)

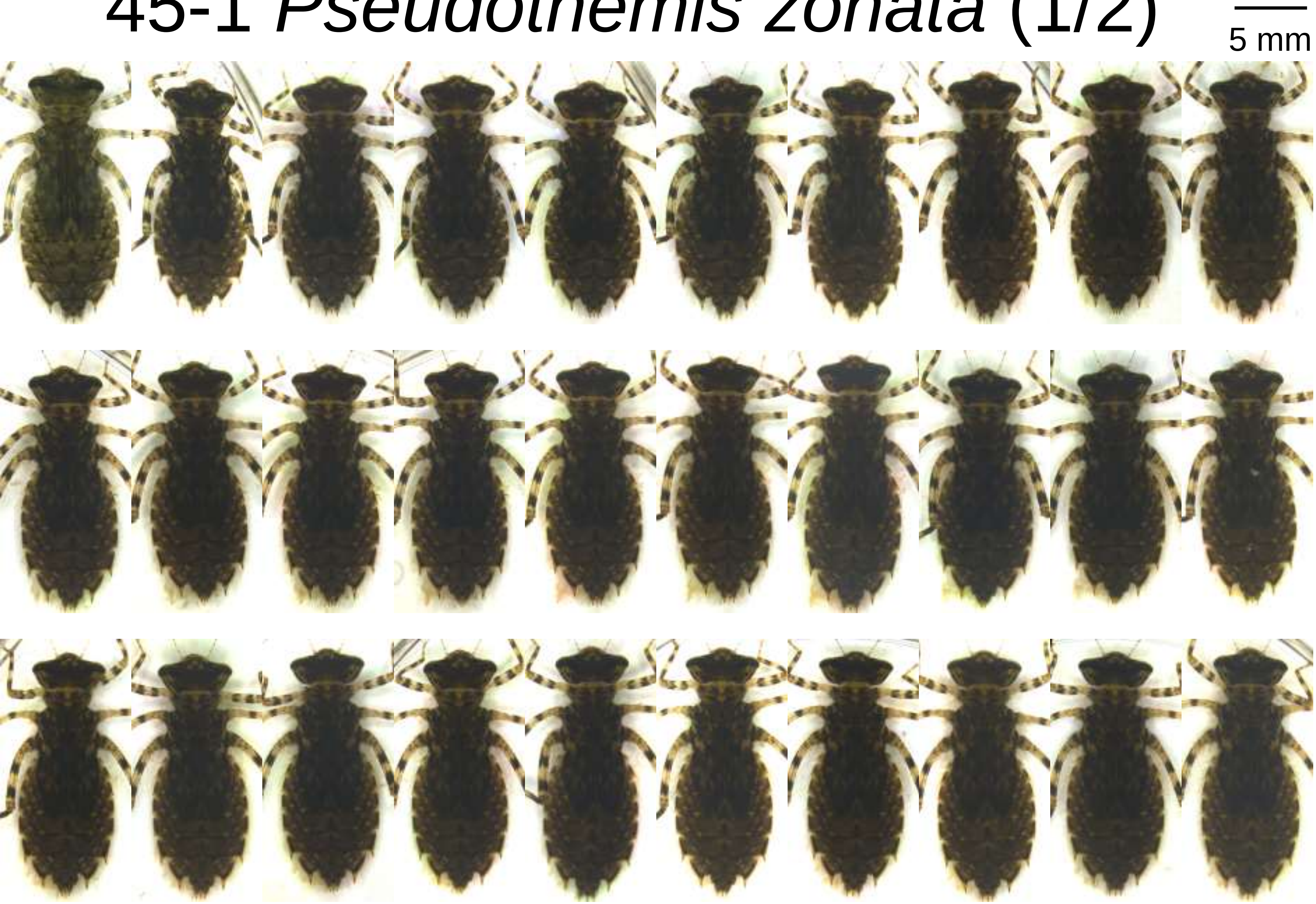

45-1 *Pseudothemis zonata* (2/2)

5 mm

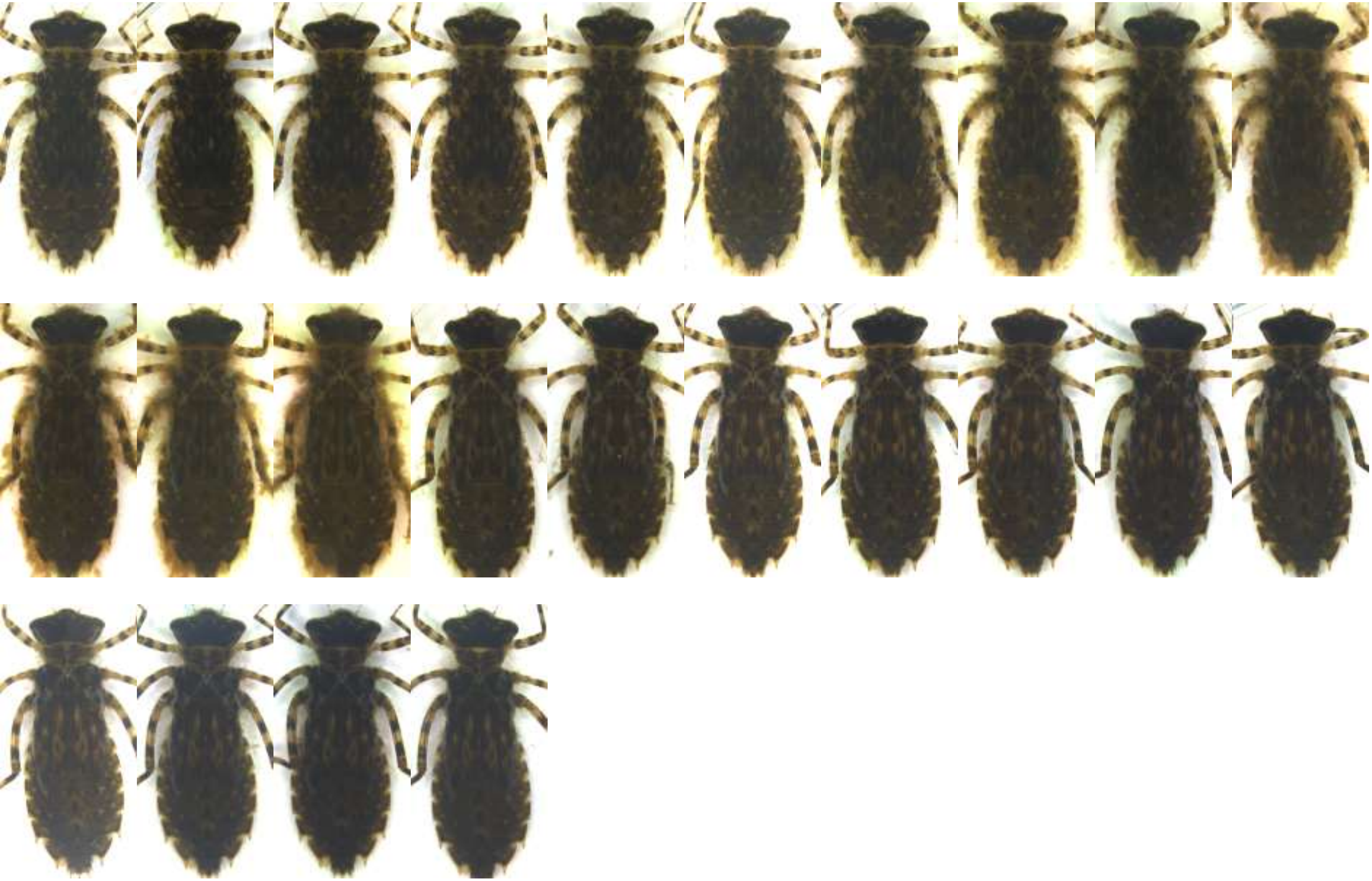

45-2 *Pseudothemis zonata* (1/2)

5 mm

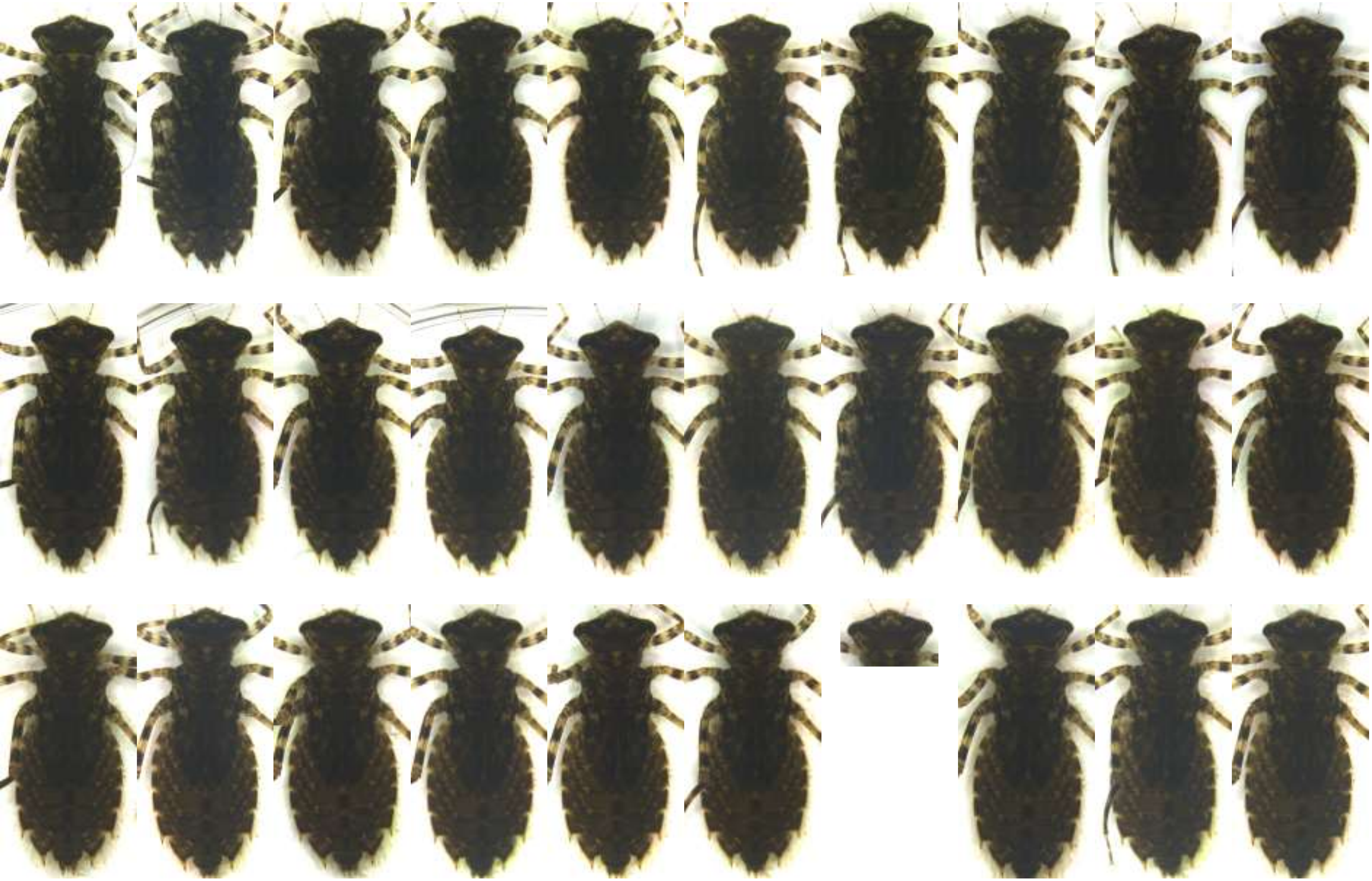

45-2 *Pseudothemis zonata* (2/2)

5 mm

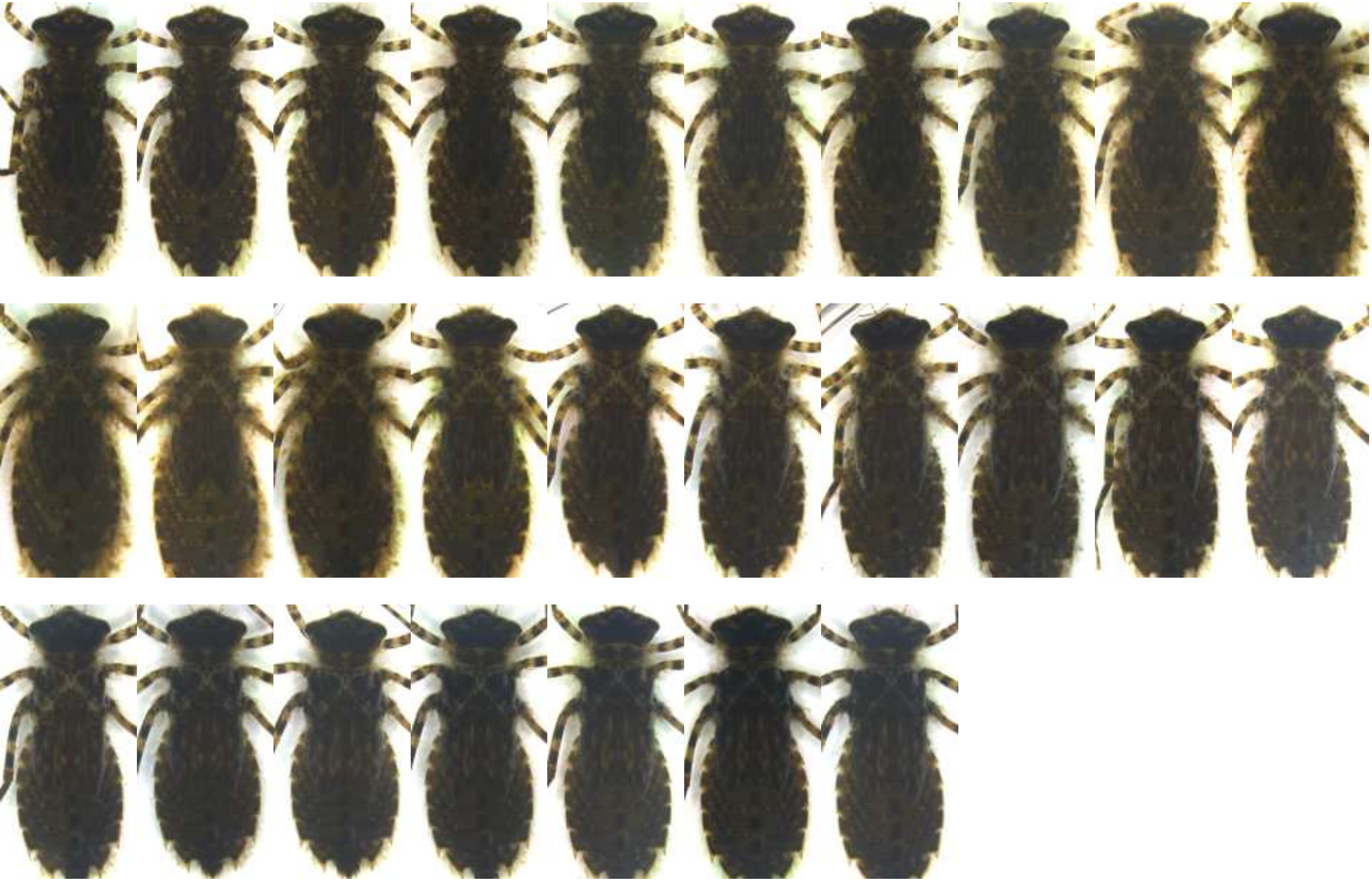

45-3 *Pseudothemis zonata* (1/2)

5 mm

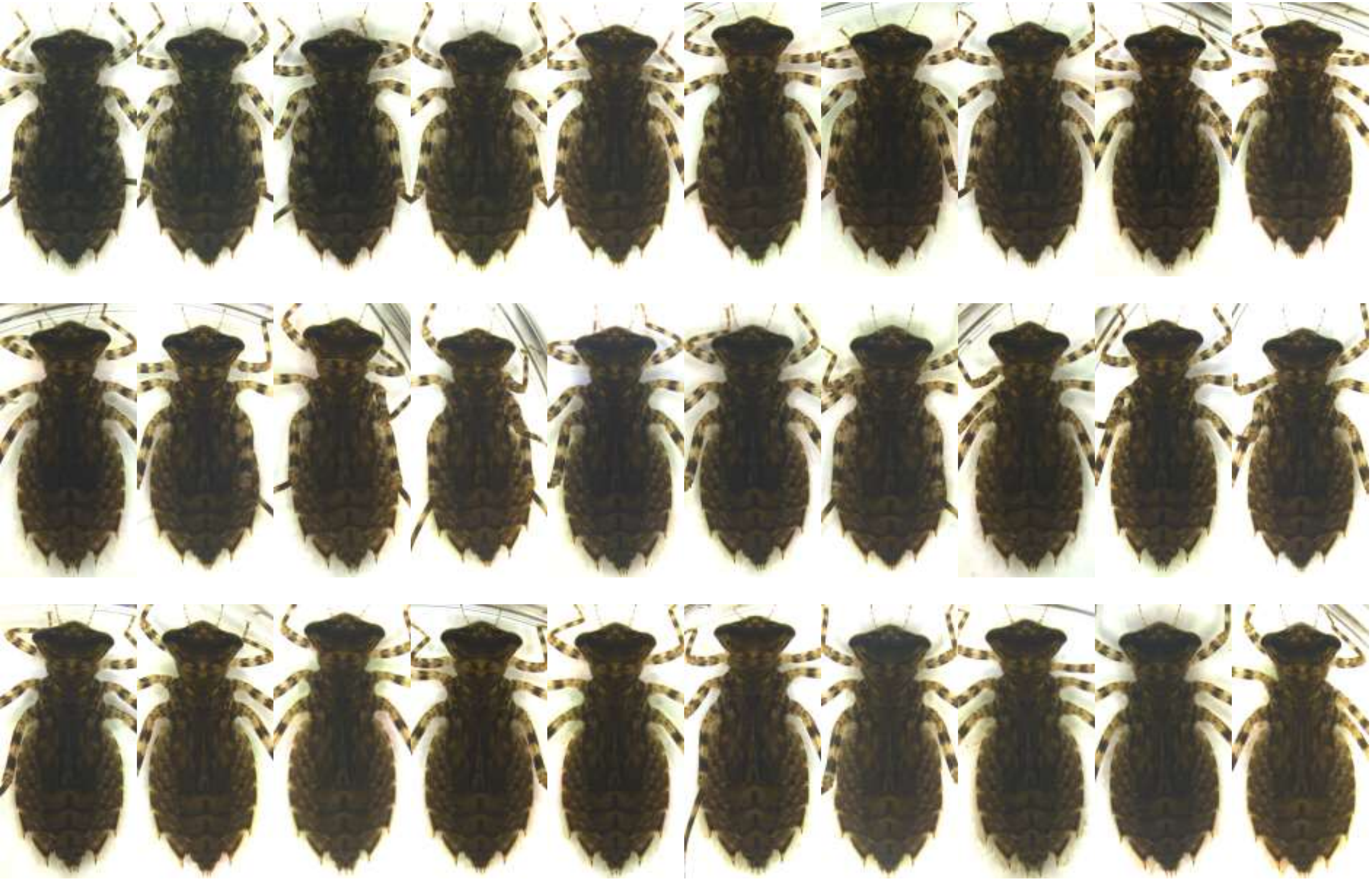

45-3 *Pseudothemis zonata* (2/2)

5 mm

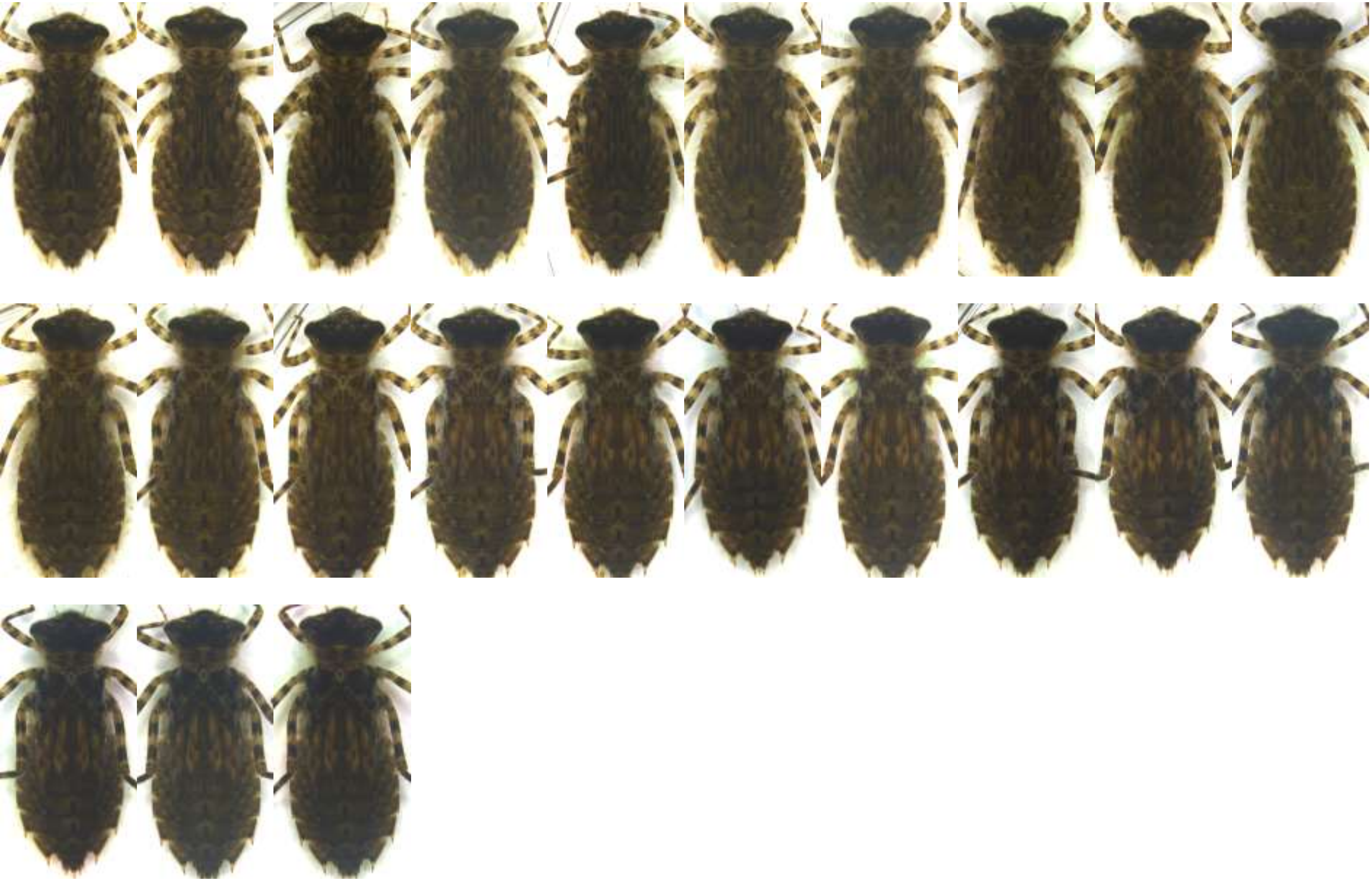

45-4 *Pseudothemis zonata* (1/2)

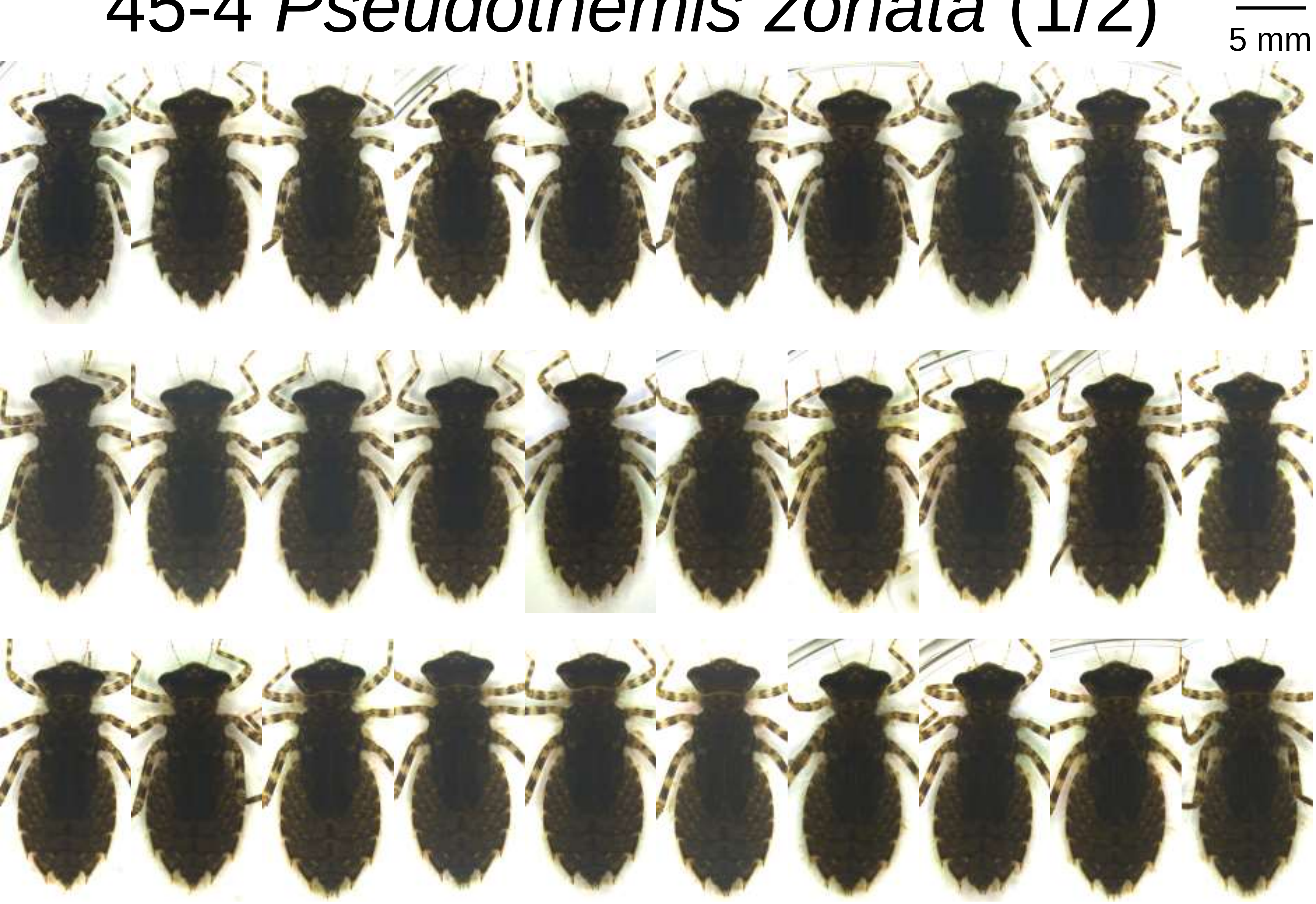

45-4 *Pseudothemis zonata* (2/2)

5 mm

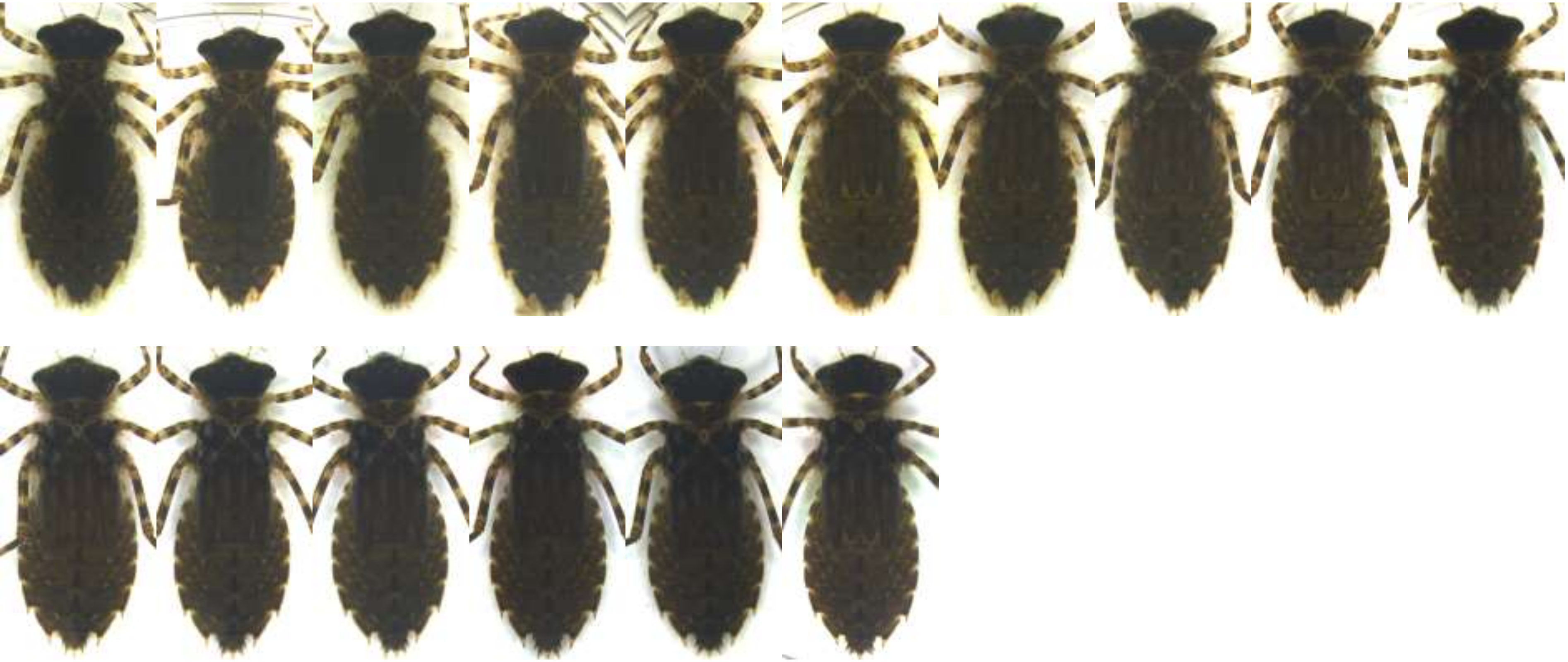

46-1 *Deielia phaon* (1/2)

5 mm

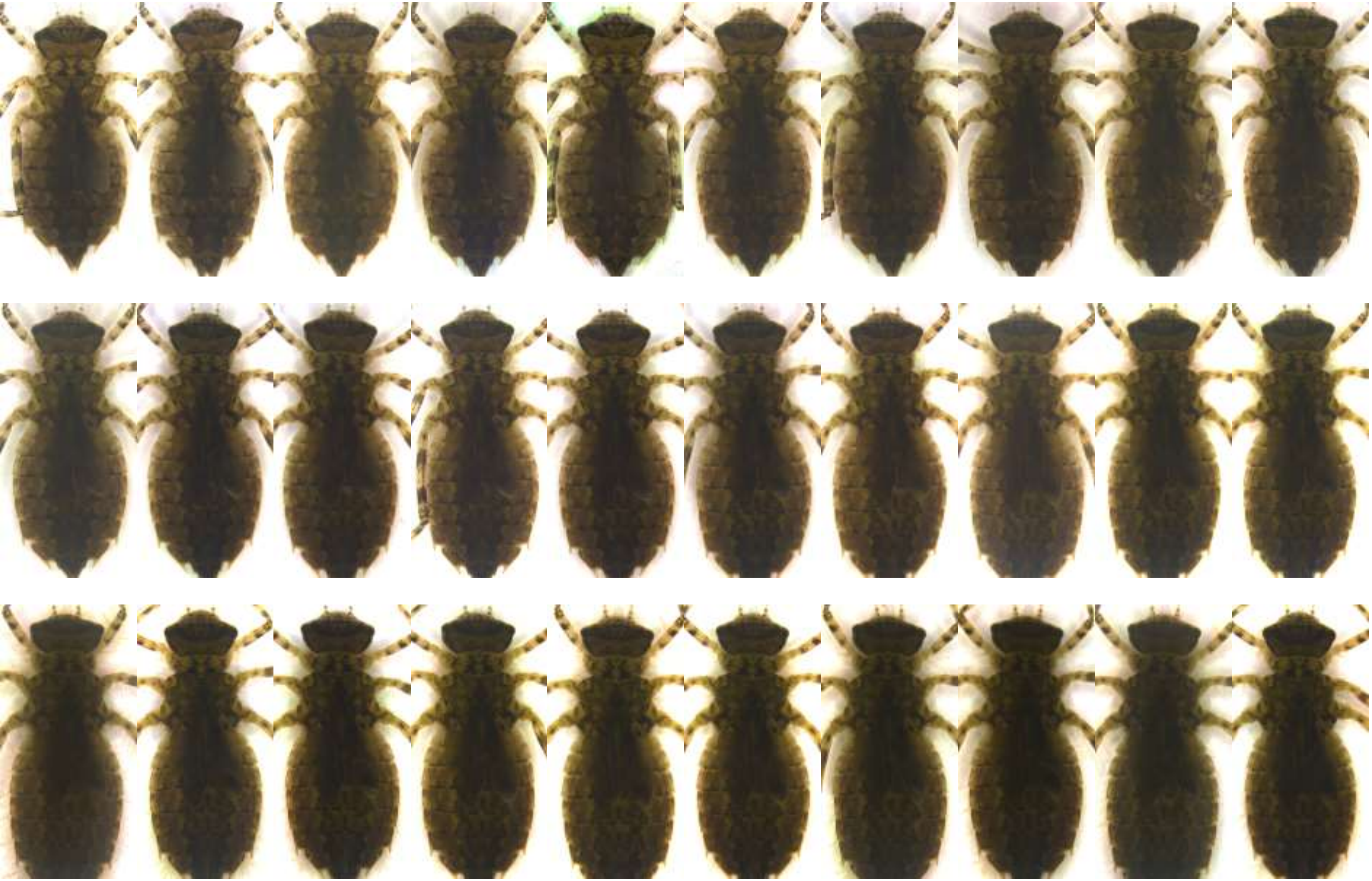

46-1 *Deielia phaon* (2/2)

5 mm

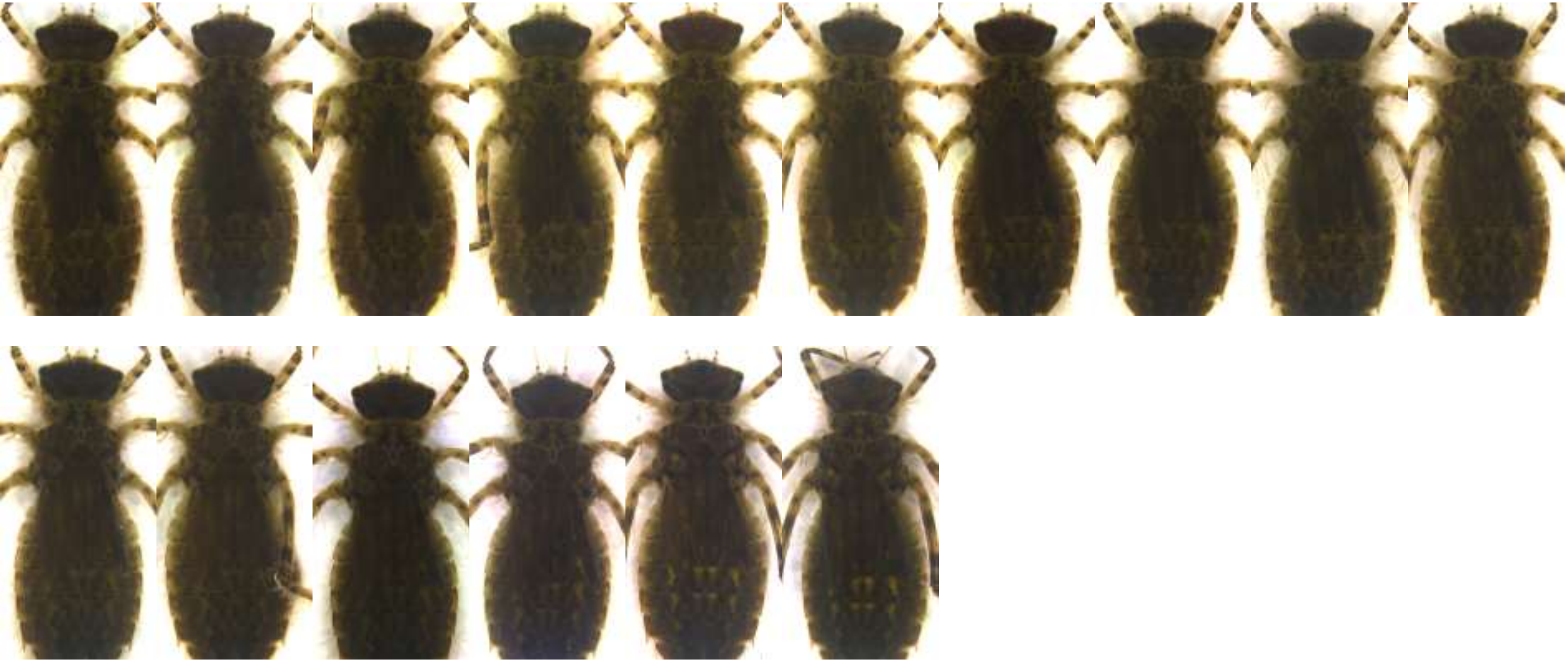

47-1 *Acisoma panorpoides* (1/2)

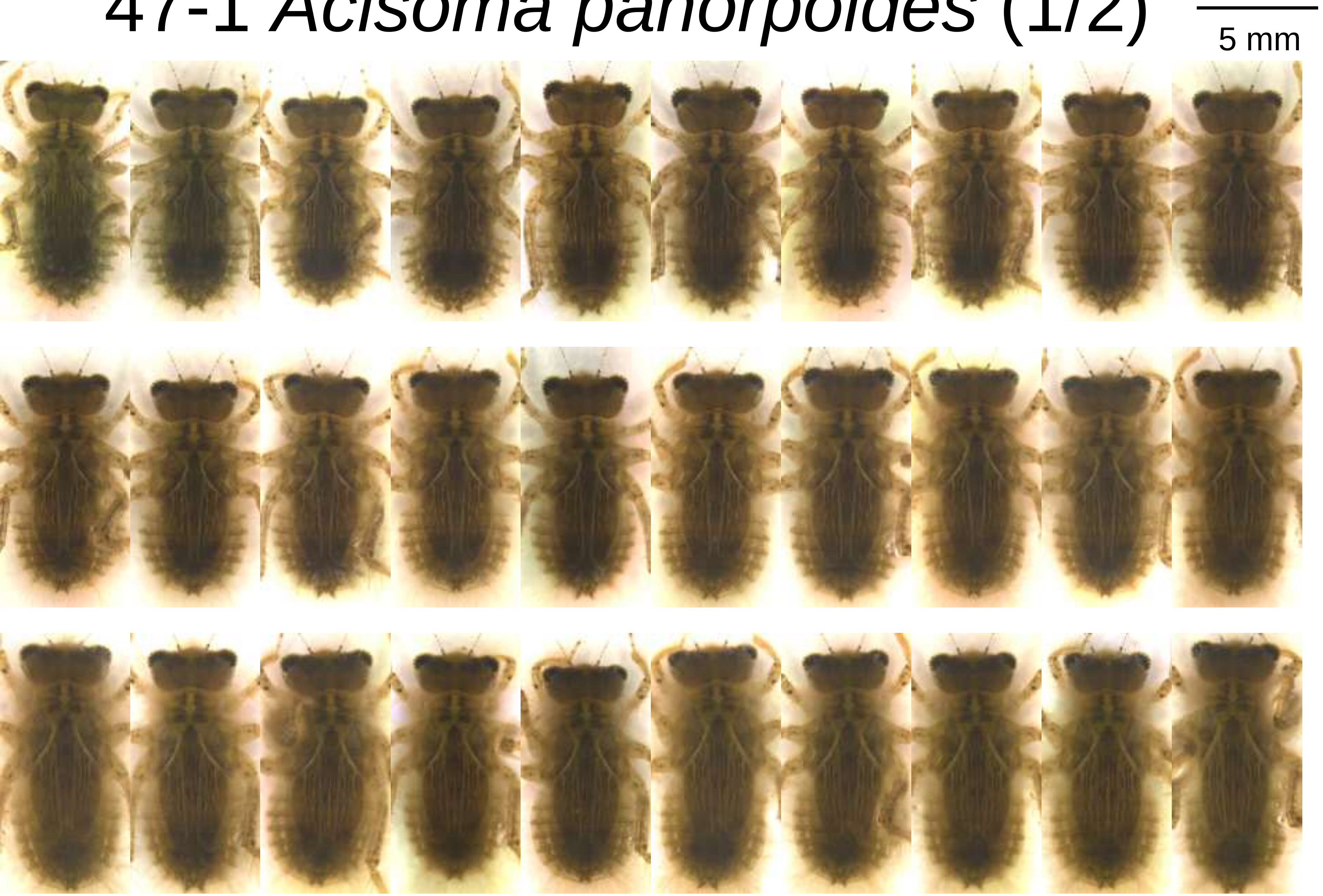

47-1 *Acisoma panorpoides* (2/2)

5 mm

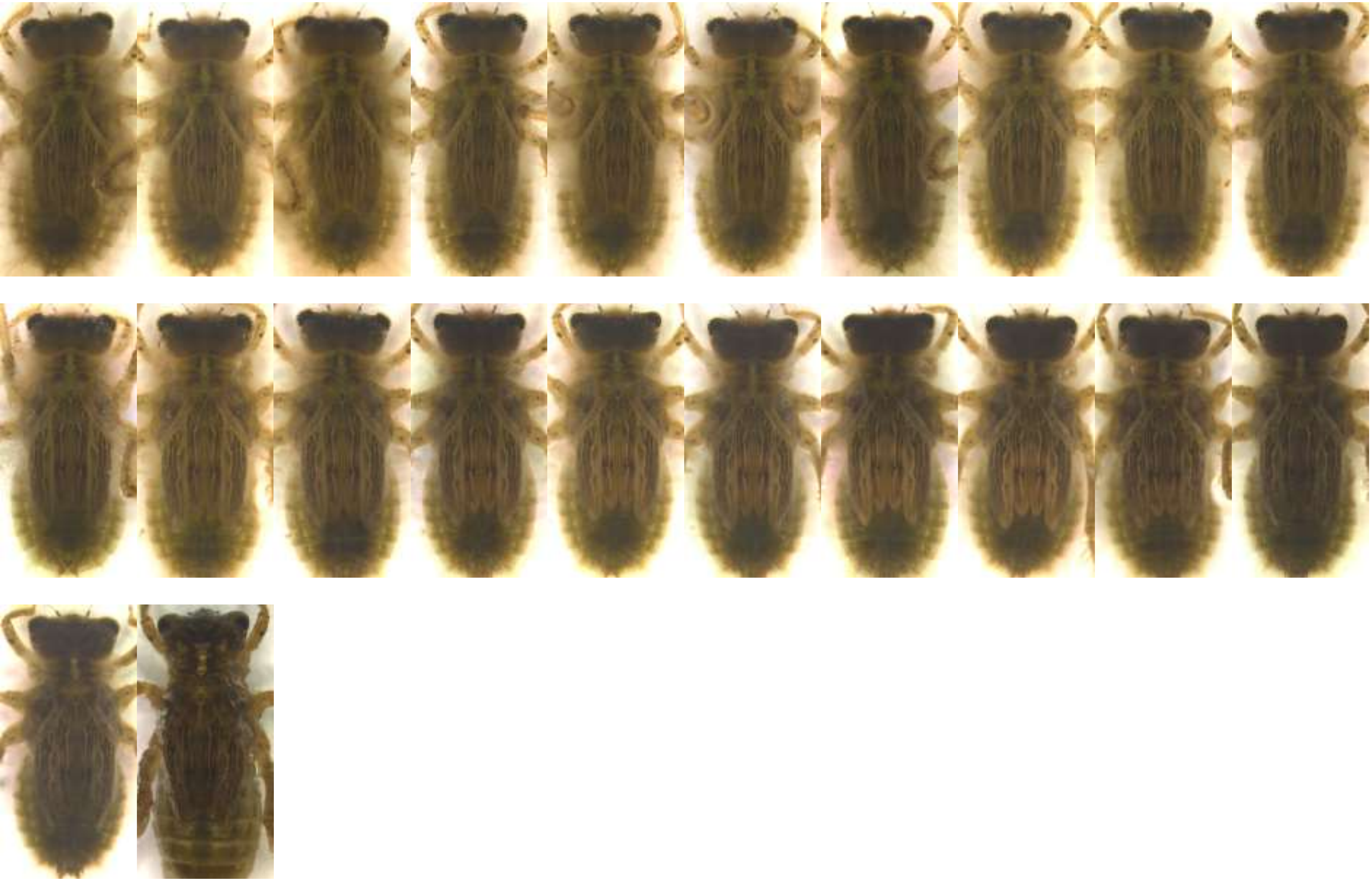

48-1 *Crocothemis servilia* (1/1)

5 mm

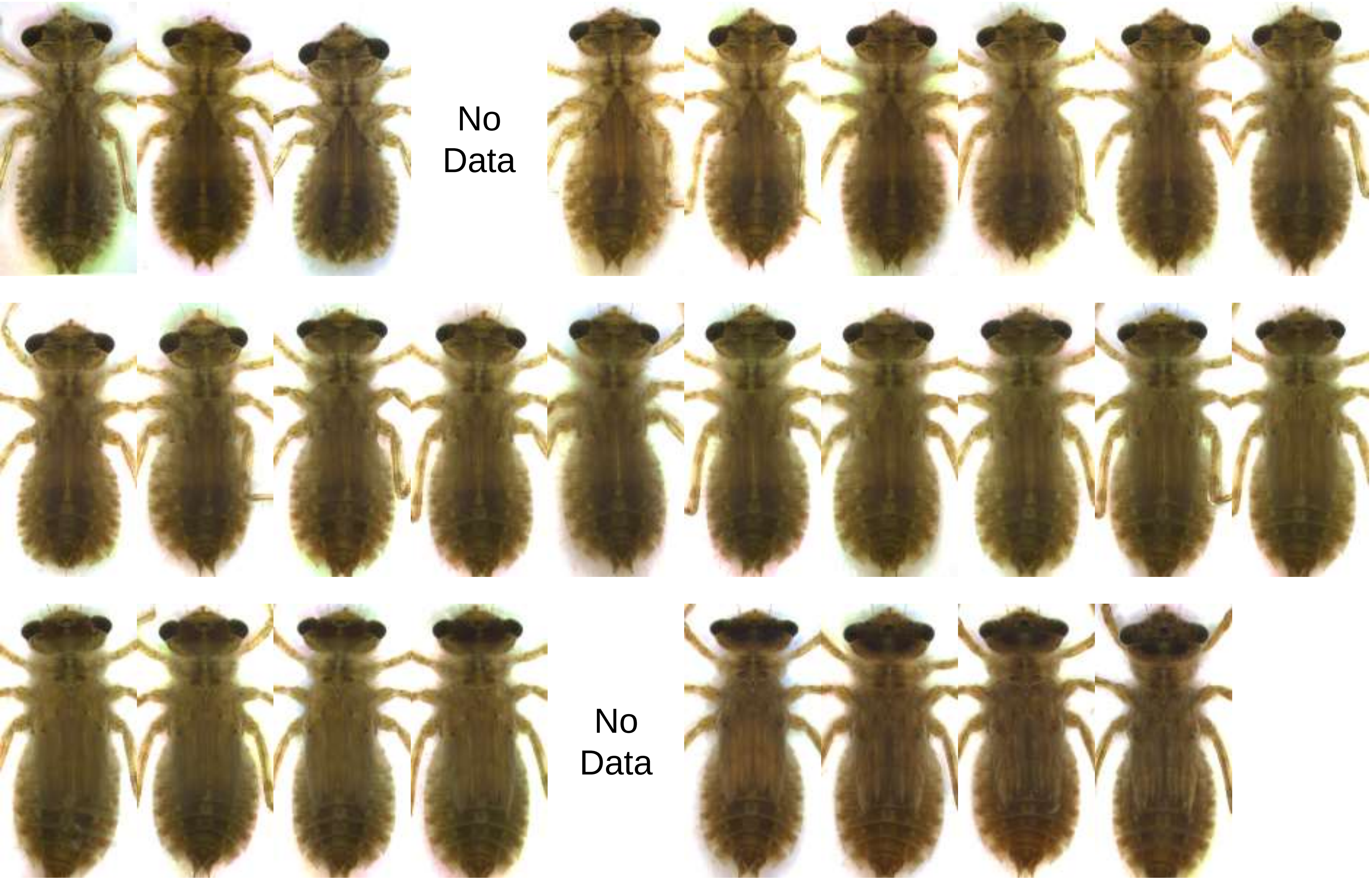

48-2 *Crocothemis servilia* (1/2)

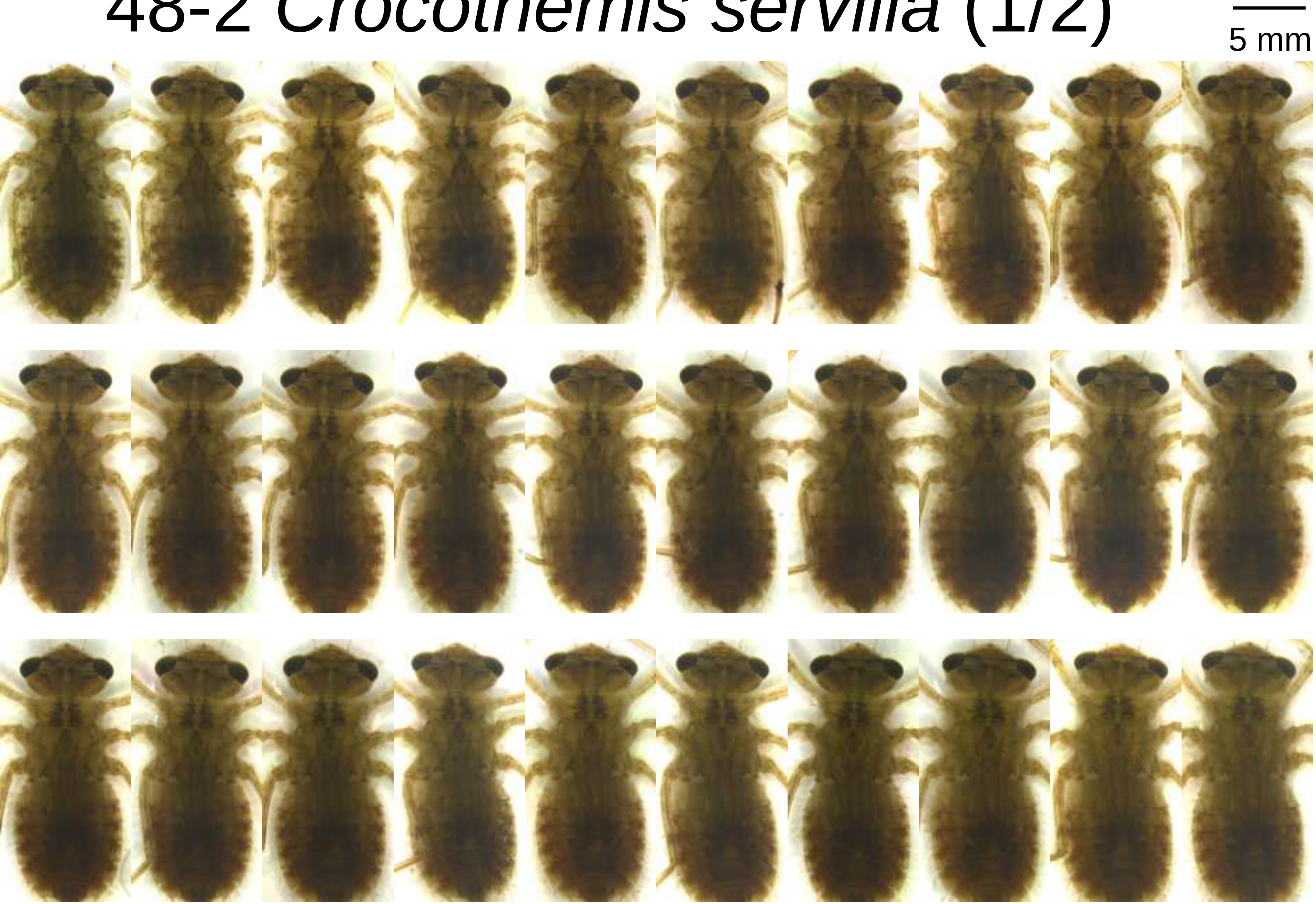

48-2 *Crocothemis servilia* (2/2)

5 mm

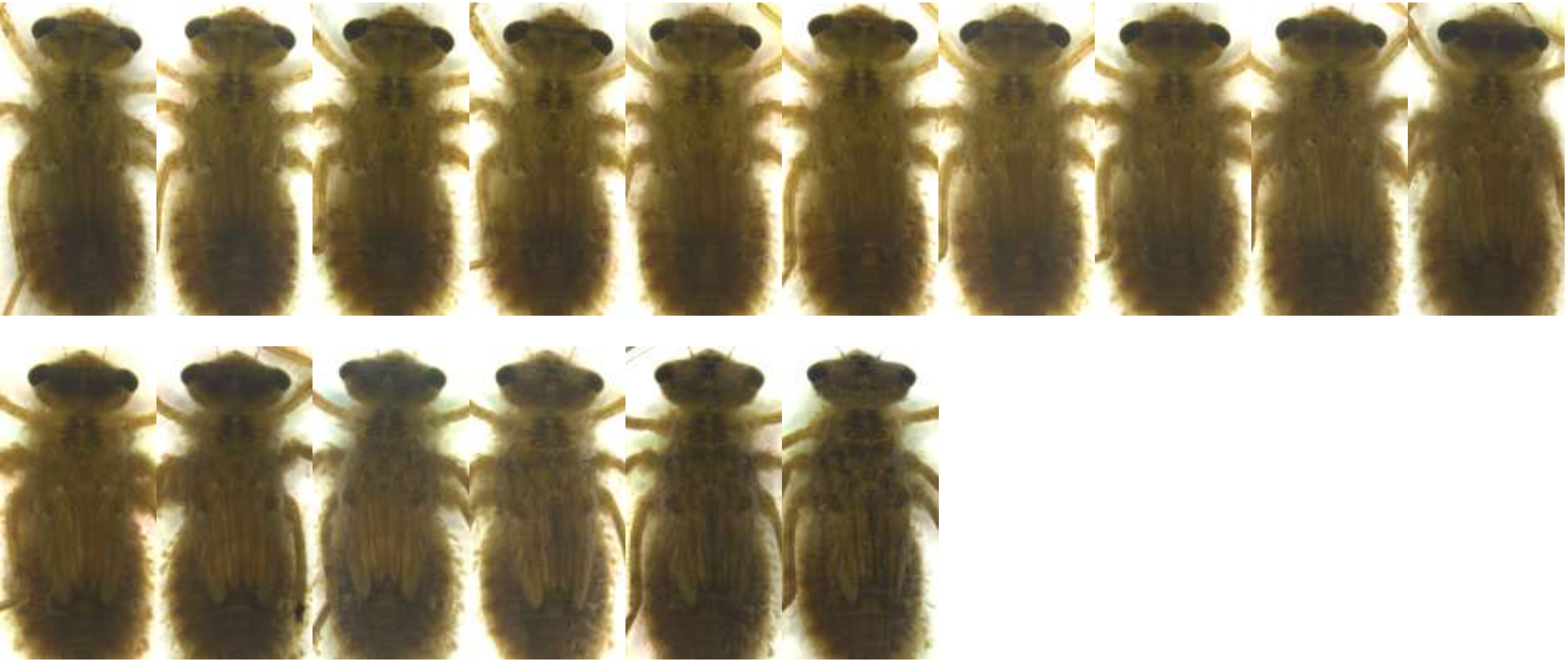

48-4 *Crocothemis servilia* (1/2)

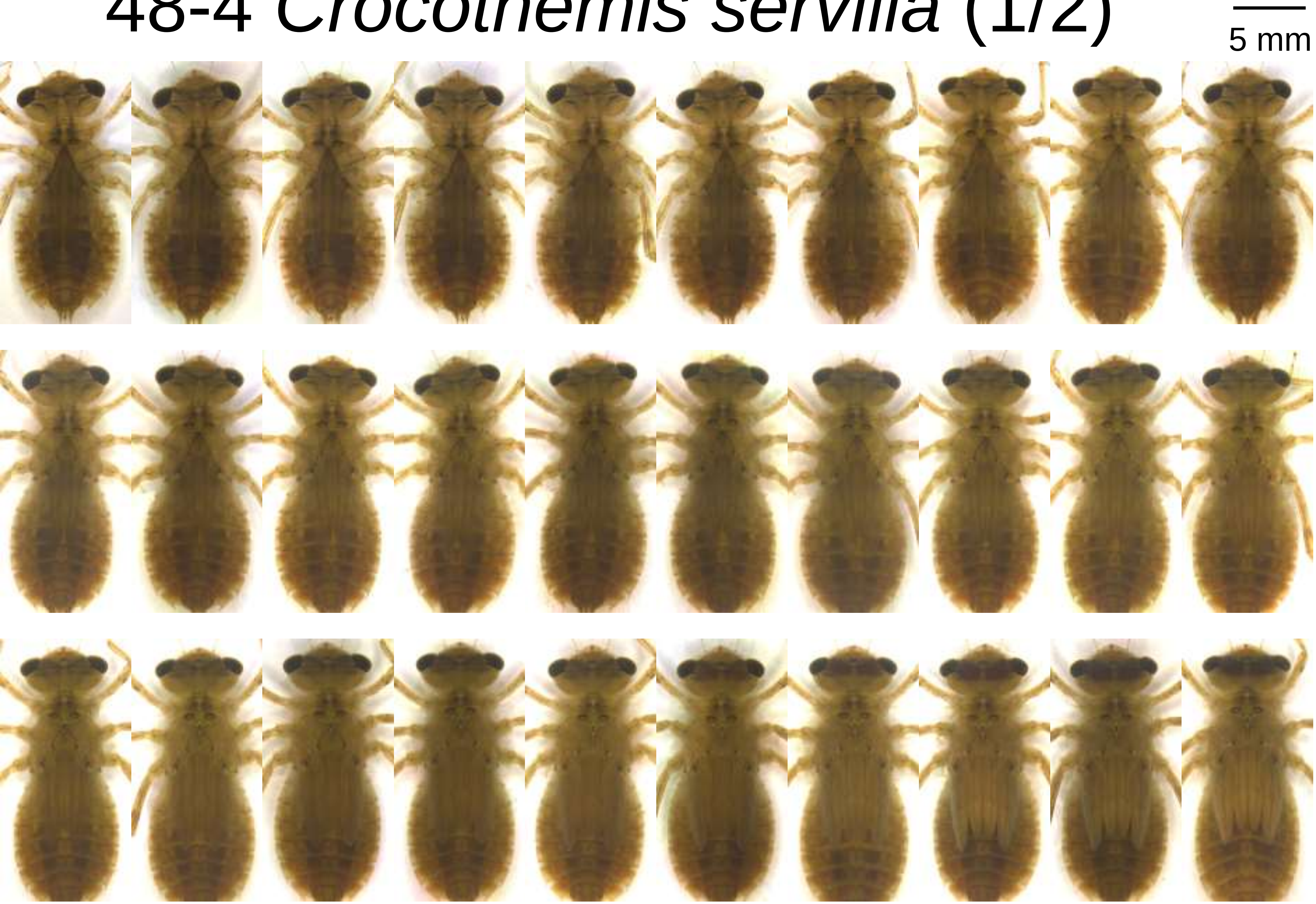

48-4 *Crocothemis servilia* (2/2)

5 mm

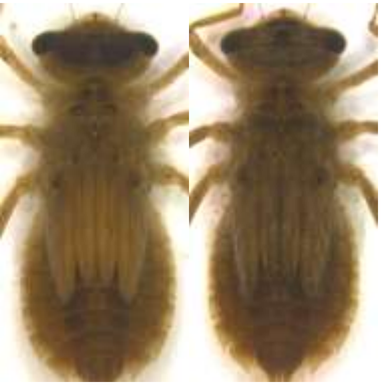

48-5 *Crocothemis servilia* (1/1)

5 mm

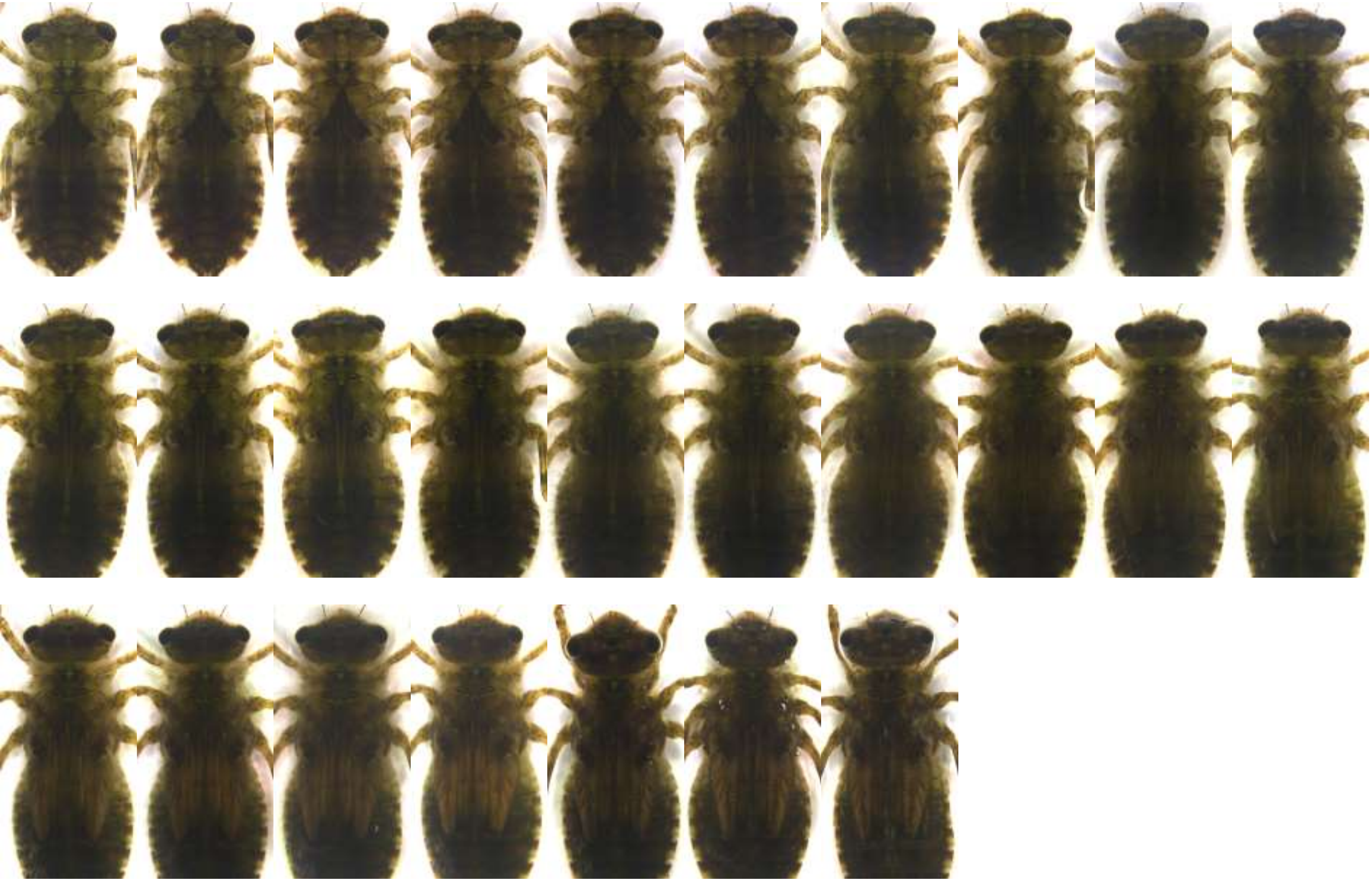

48-6 *Crocothemis servilia* (1/2)

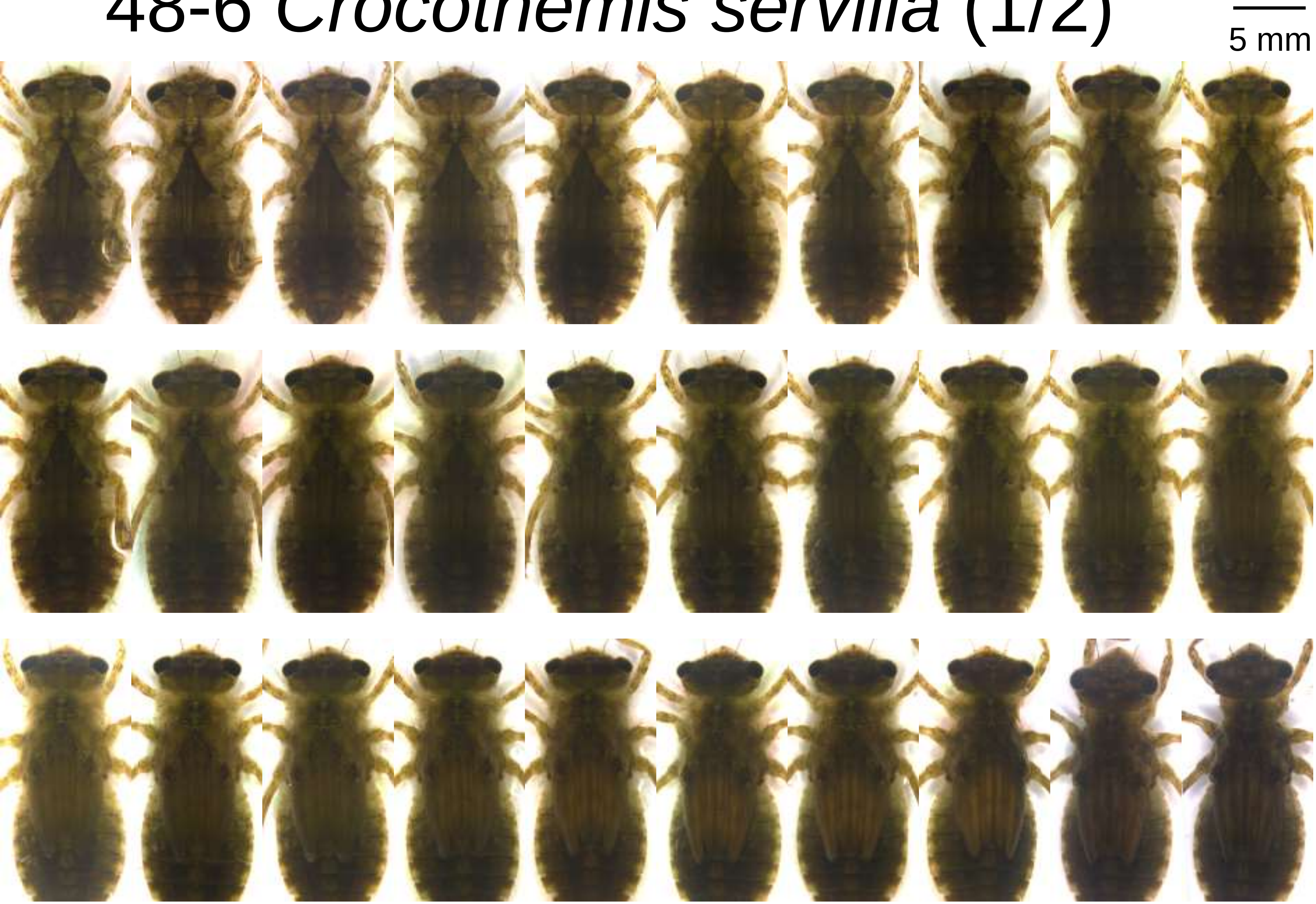

48-6 *Crocothemis servilia* (2/2)

5 mm

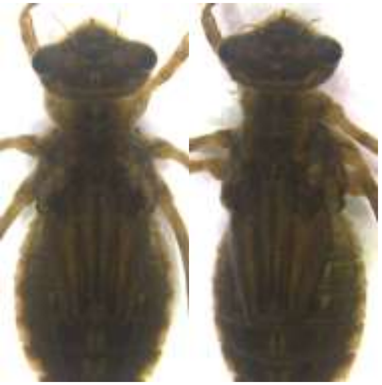

49-1 *Orthetrum albistylum* (1/2)

5 mm

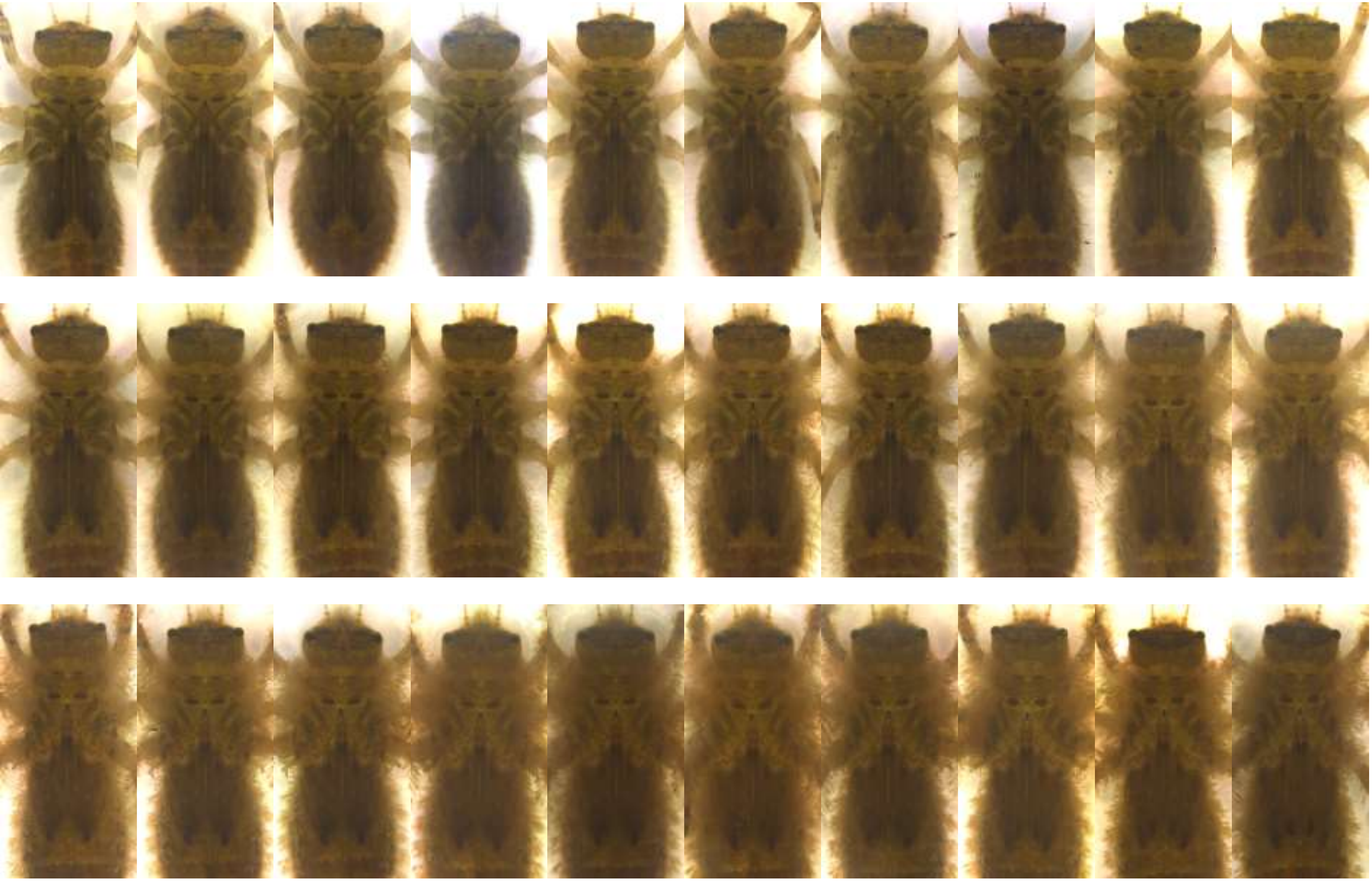

49-1 *Orthetrum albistylum* (2/2)

5 mm

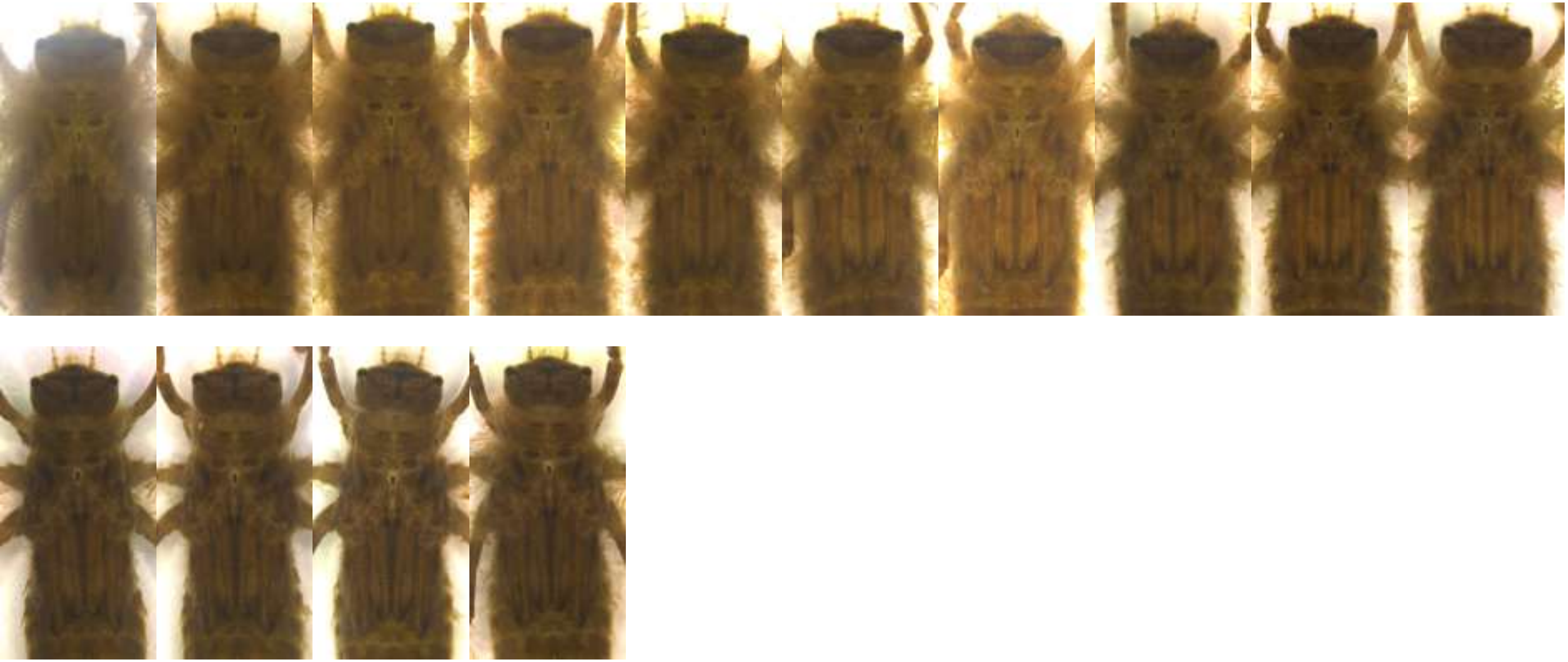

49-3 *Orthetrum albistylum* (1/2)

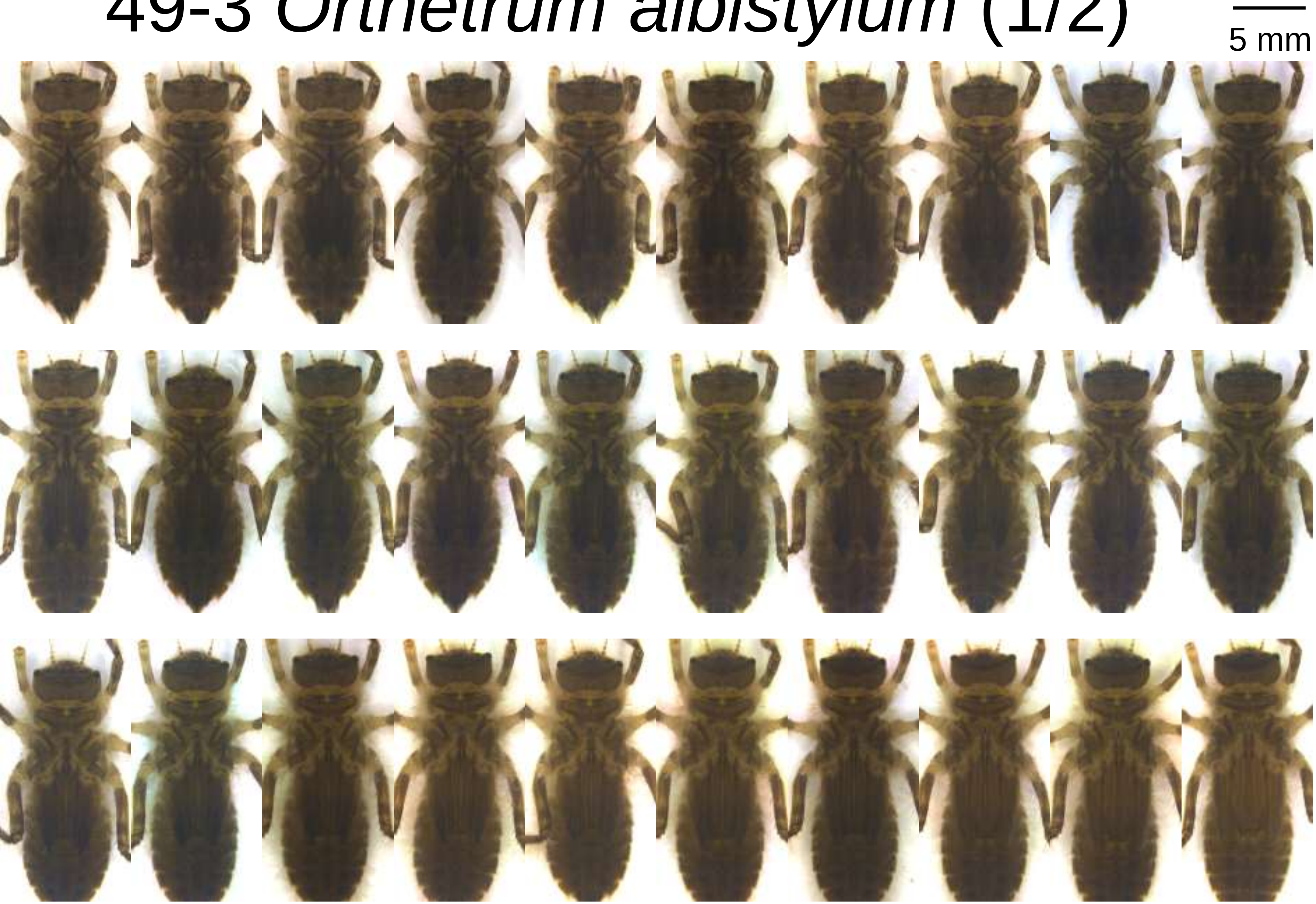

49-3 *Orthetrum albistylum* (2/2)

5 mm

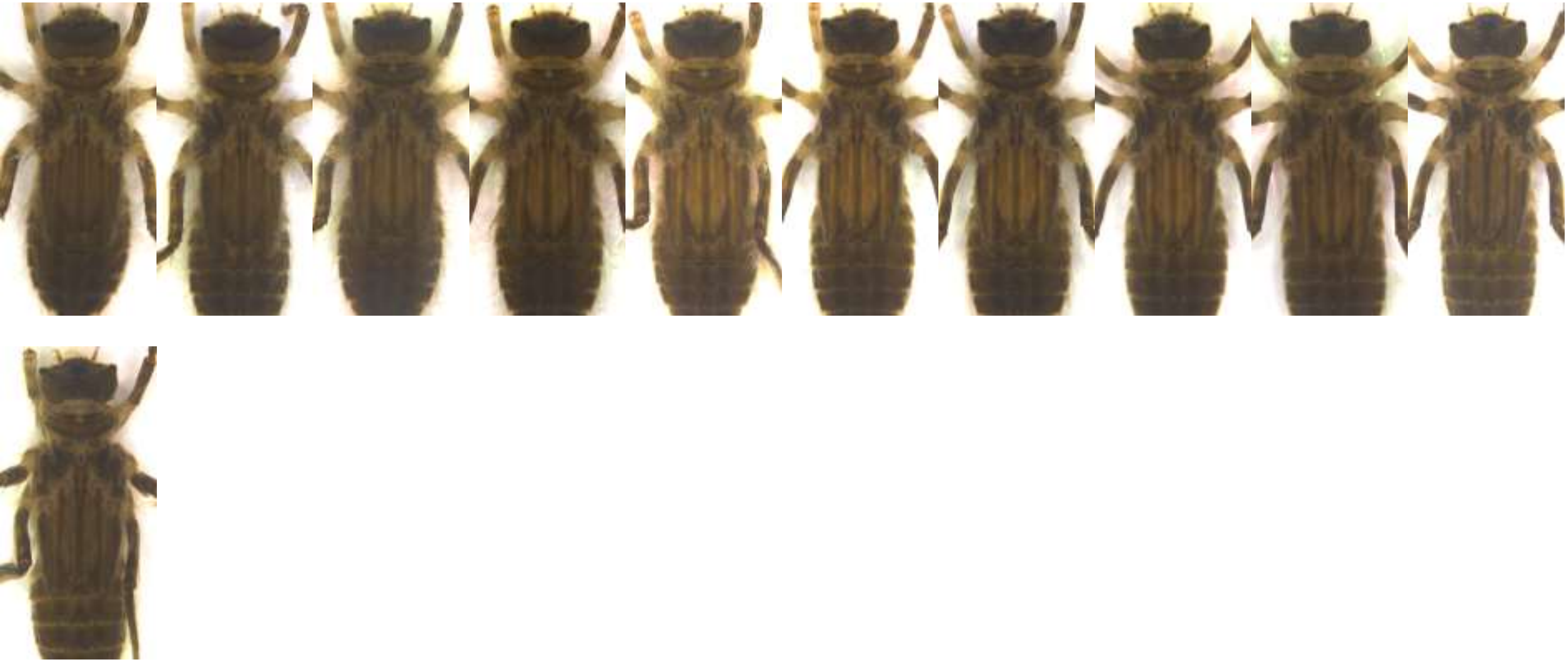

49-4 *Orthetrum albistylum* (1/2)

5 mm

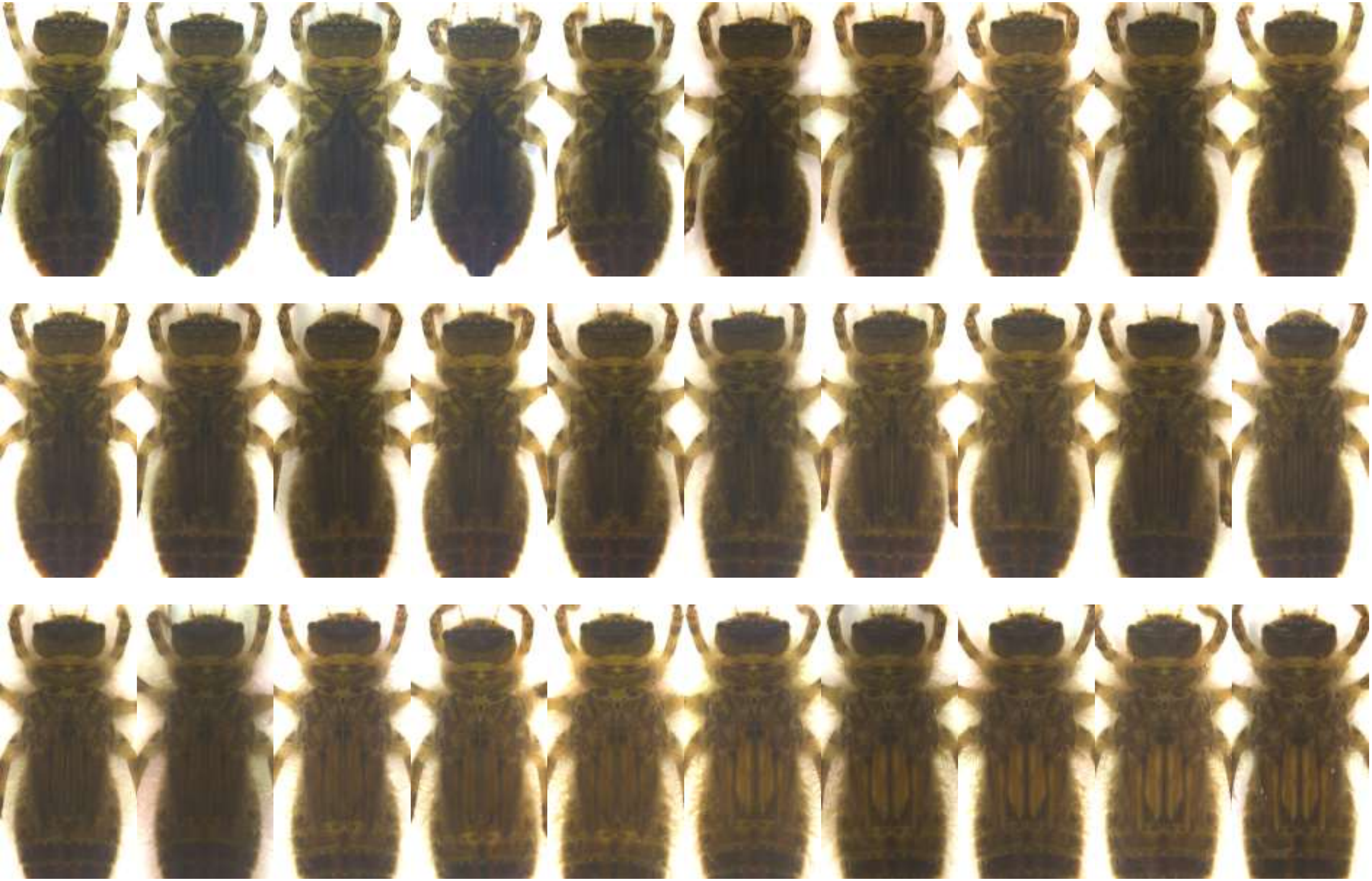

49-4 *Orthetrum albistylum* (2/2)

5 mm

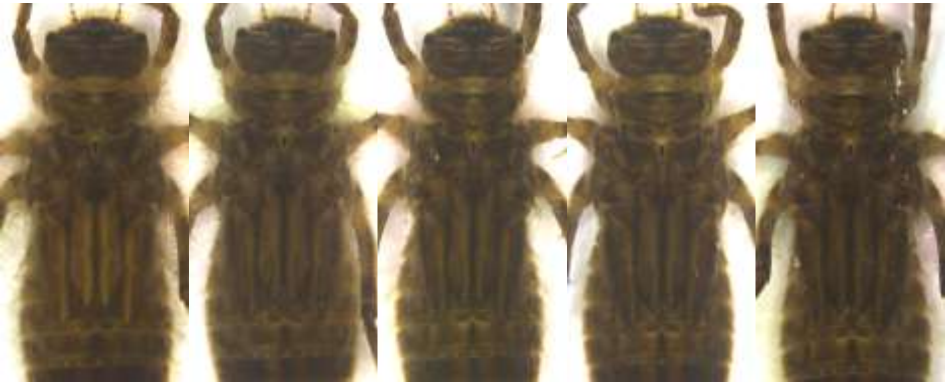

49-5 *Orthetrum albistylum* (1/2)

5 mm

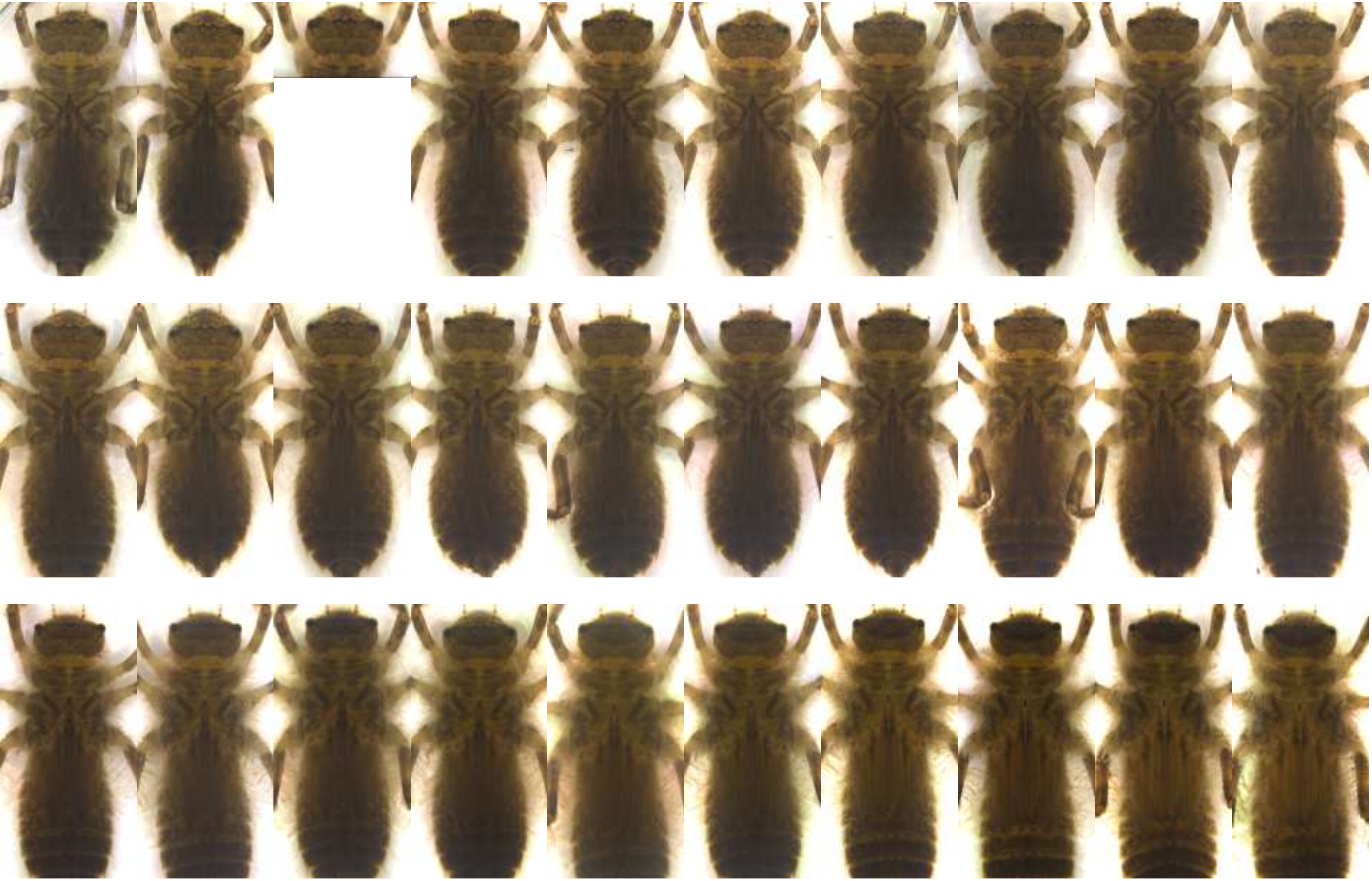

49-5 *Orthetrum albistylum* (2/2)

5 mm

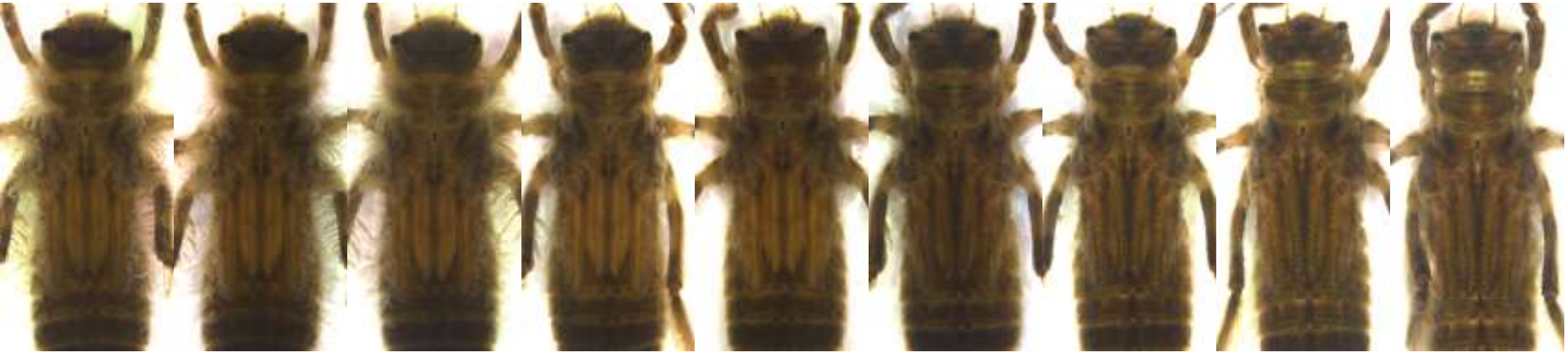

49-6 *Orthetrum albistylum* (1/2)

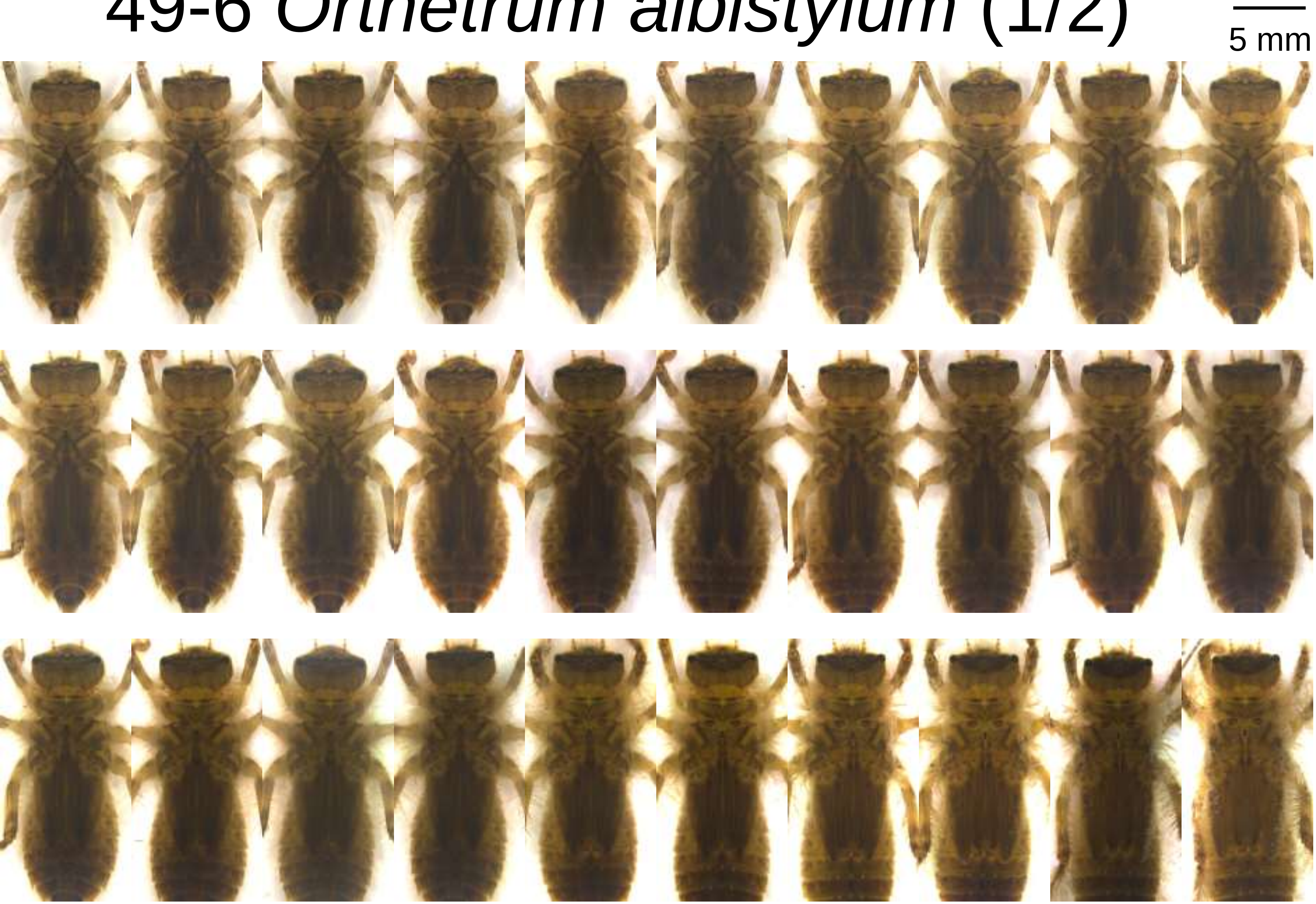

49-6 *Orthetrum albistylum* (2/2)

5 mm

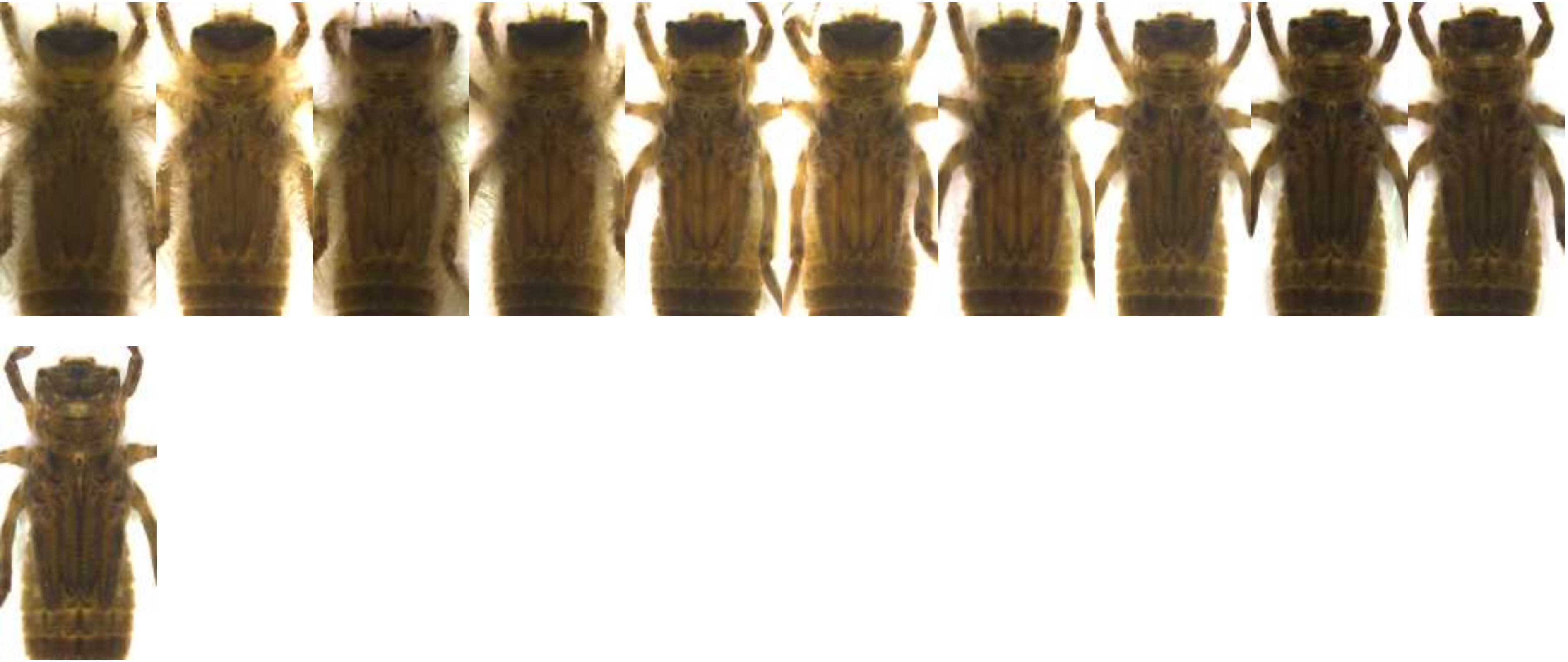

Supplement: Supplementary file 2 — Supplementary Figure S1. [file 41598_2021_84639_MOESM2_ESM.pdf]
