# Supplementary material for: Comprehensive comparative morphology and developmental staging of final instar larvae toward metamorphosis in the insect order Odonata
Source: Sci Rep. 2021 Mar 4;11:5164. doi: 10.1038/s41598-021-84639-2 (PMC7970851; doi:10.1038/s41598-021-84639-2)

Comprehensive comparative morphology and developmental staging of final instar larvae toward metamorphosis in the insect order Odonata

Genta Okude, Takema Fukatsu, Ryo Futahashi

Figure S2

All the adjusted photos of F-1 instar larvae taken in this study.
Individual data are shown in Table S1.

1-1 *Lestes sponsa* (1/1)

2 mm

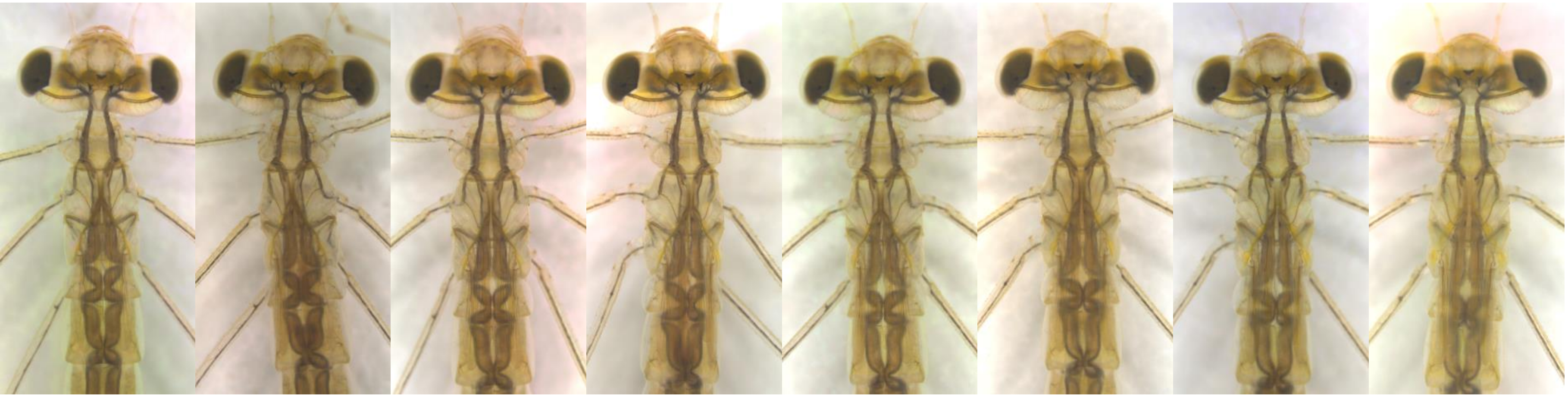

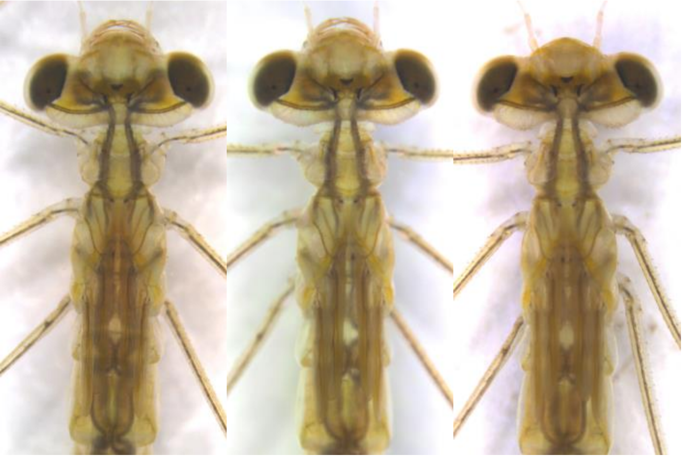

2-5 *Lestes temporalis* (1/1)

2 mm

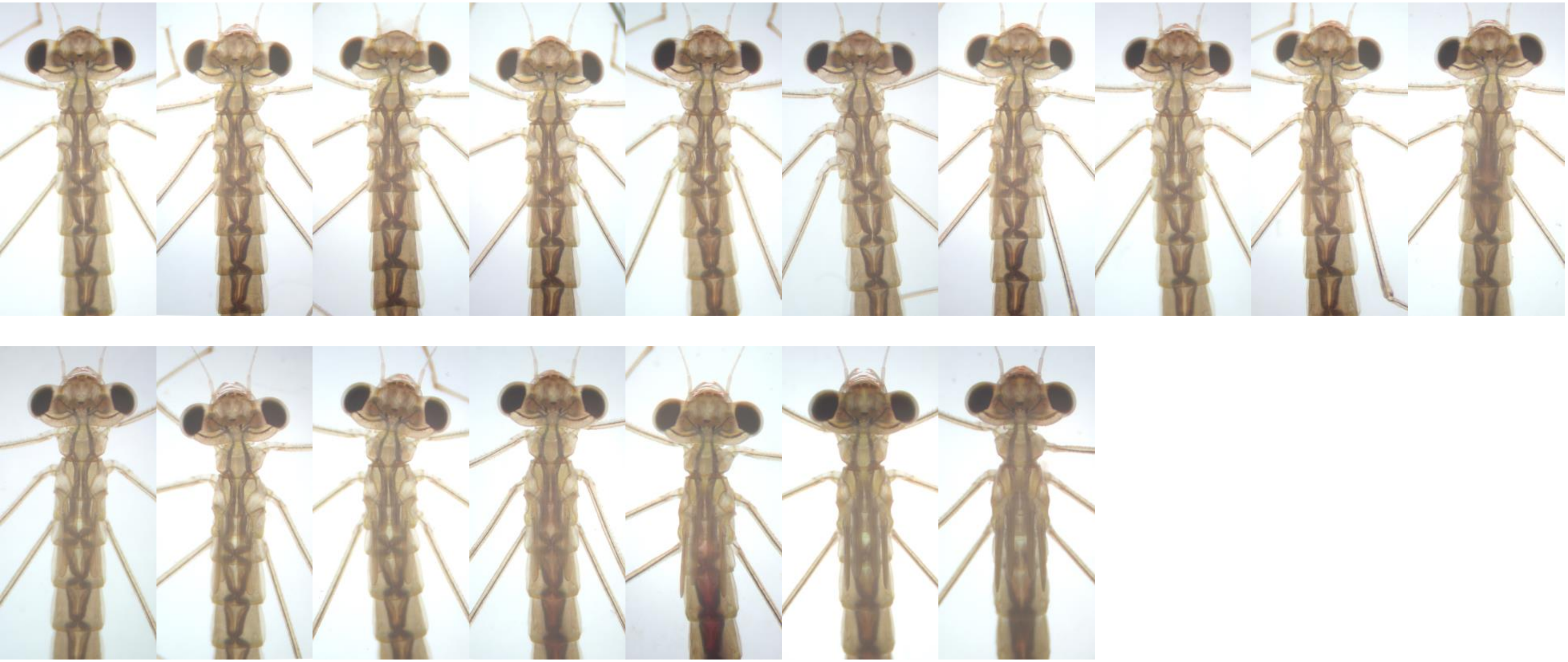

7-1 *Paracercion calamorum* (1/1)

2 mm

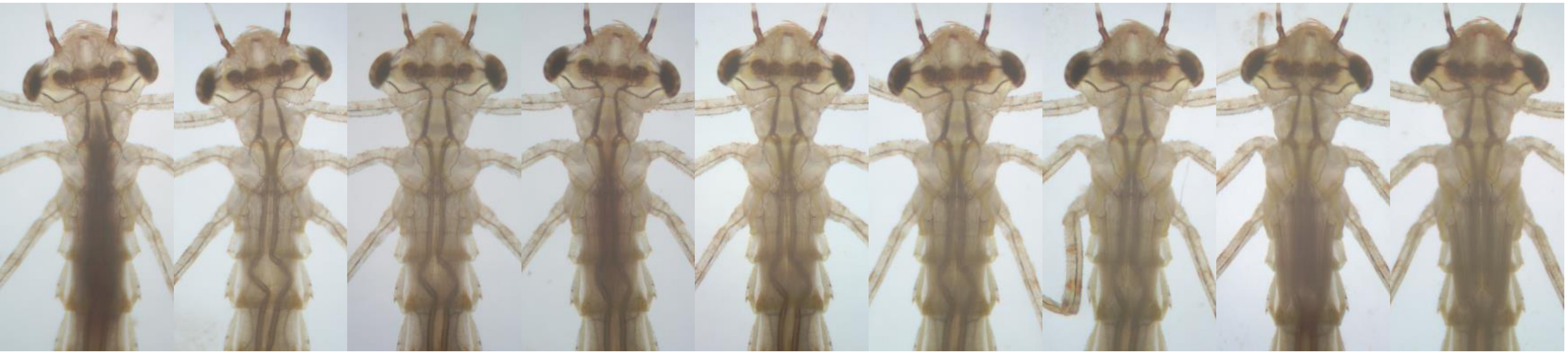

7-2 *Paracercion calamorum* (1/1)

2 mm

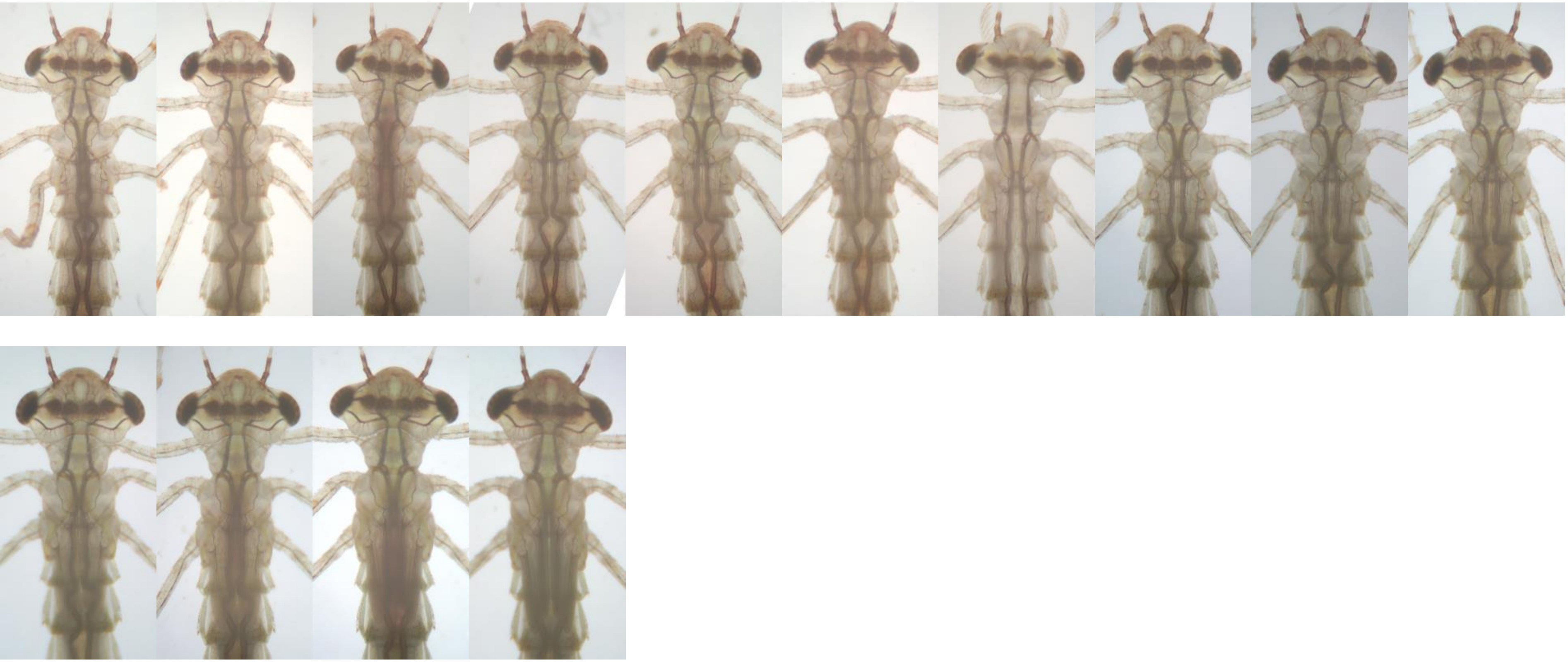

7-3 *Paracercion calamorum* (1/1)

2 mm

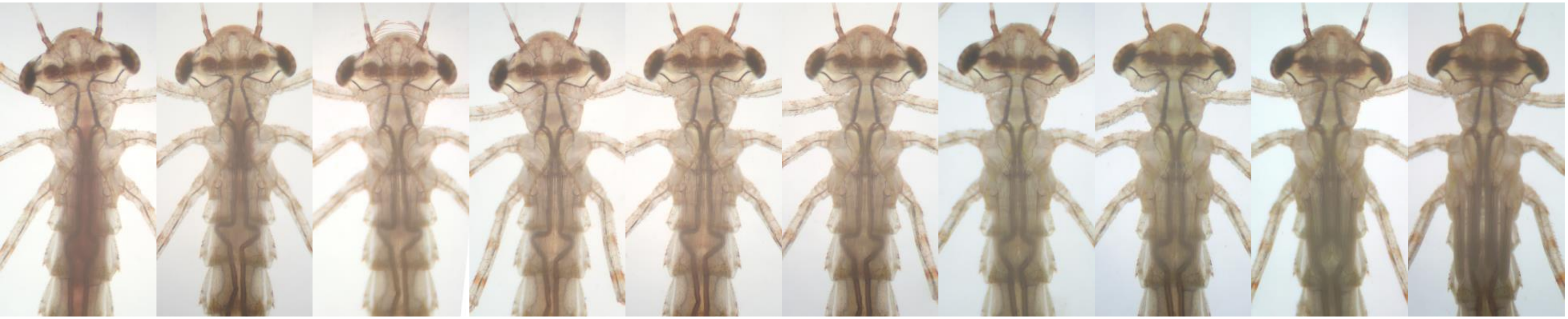

7-4 *Paracercion calamorum* (1/1)

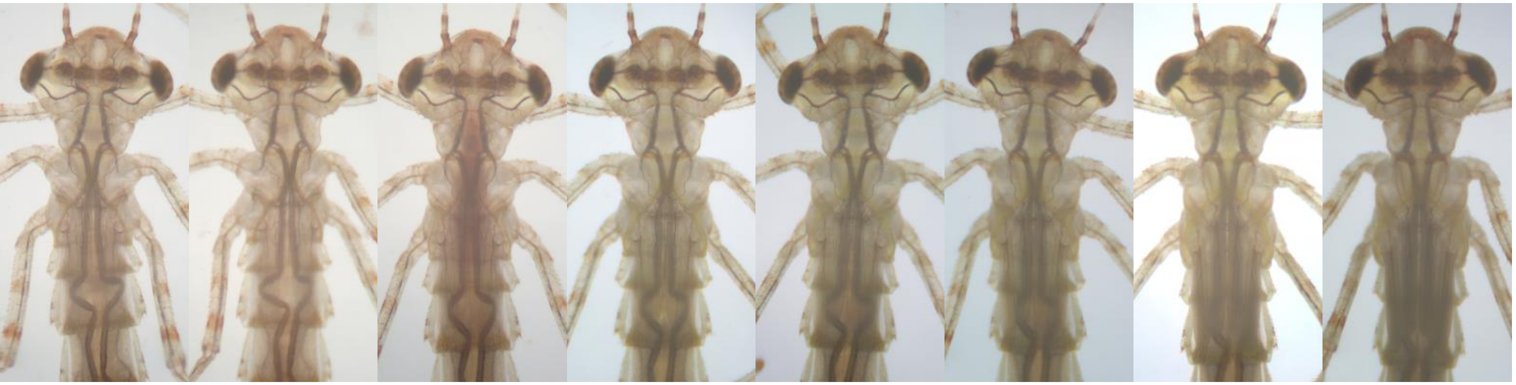

9-1 *Paracercion sieboldii* (1/1)

2 mm

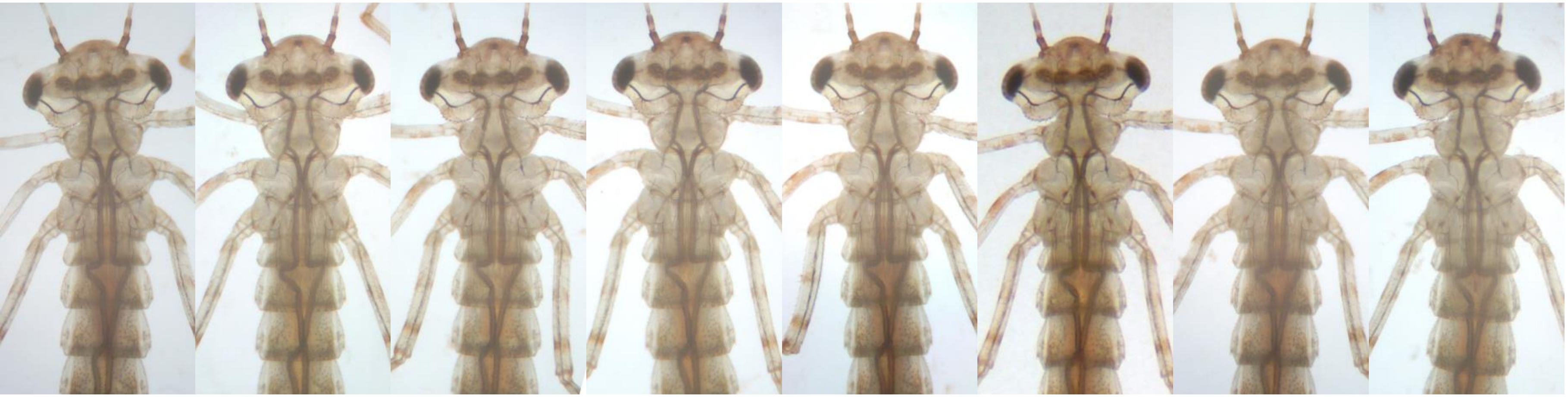

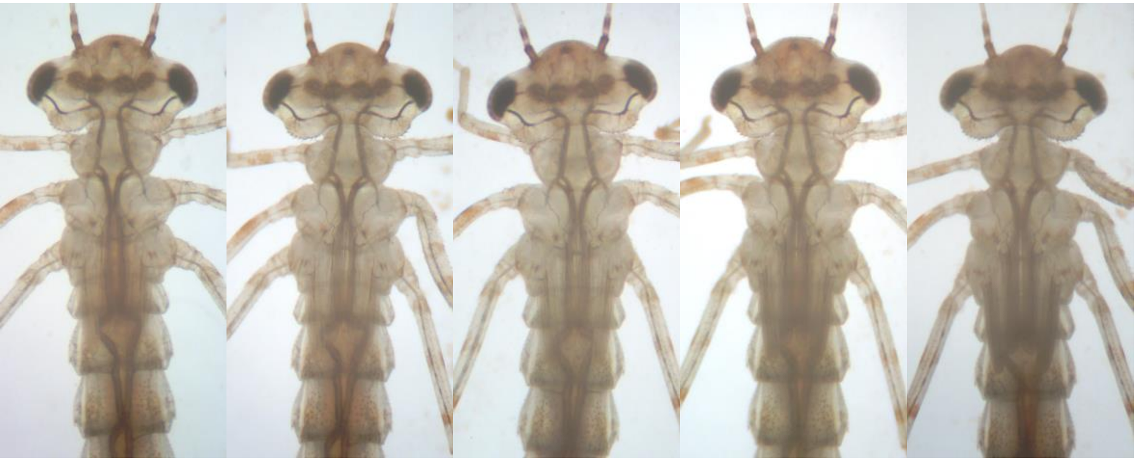

13-1 *Enallagma circulatum* (1/1)

2 mm

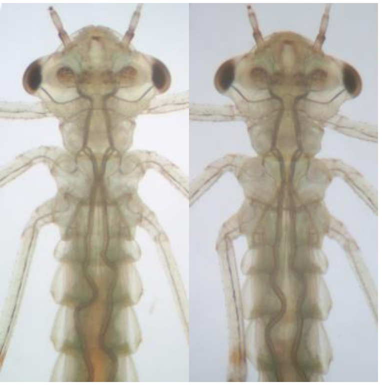

No
Data

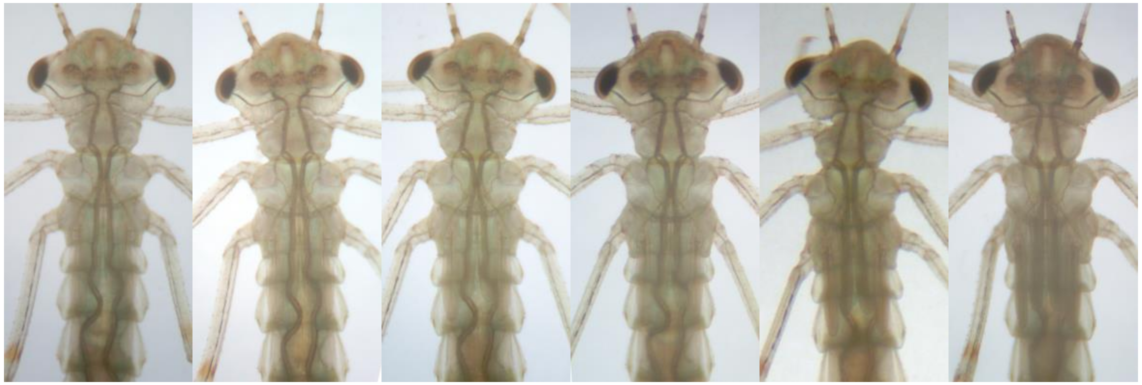

19-3 *Gynacantha japonica* (1/1)

2 mm

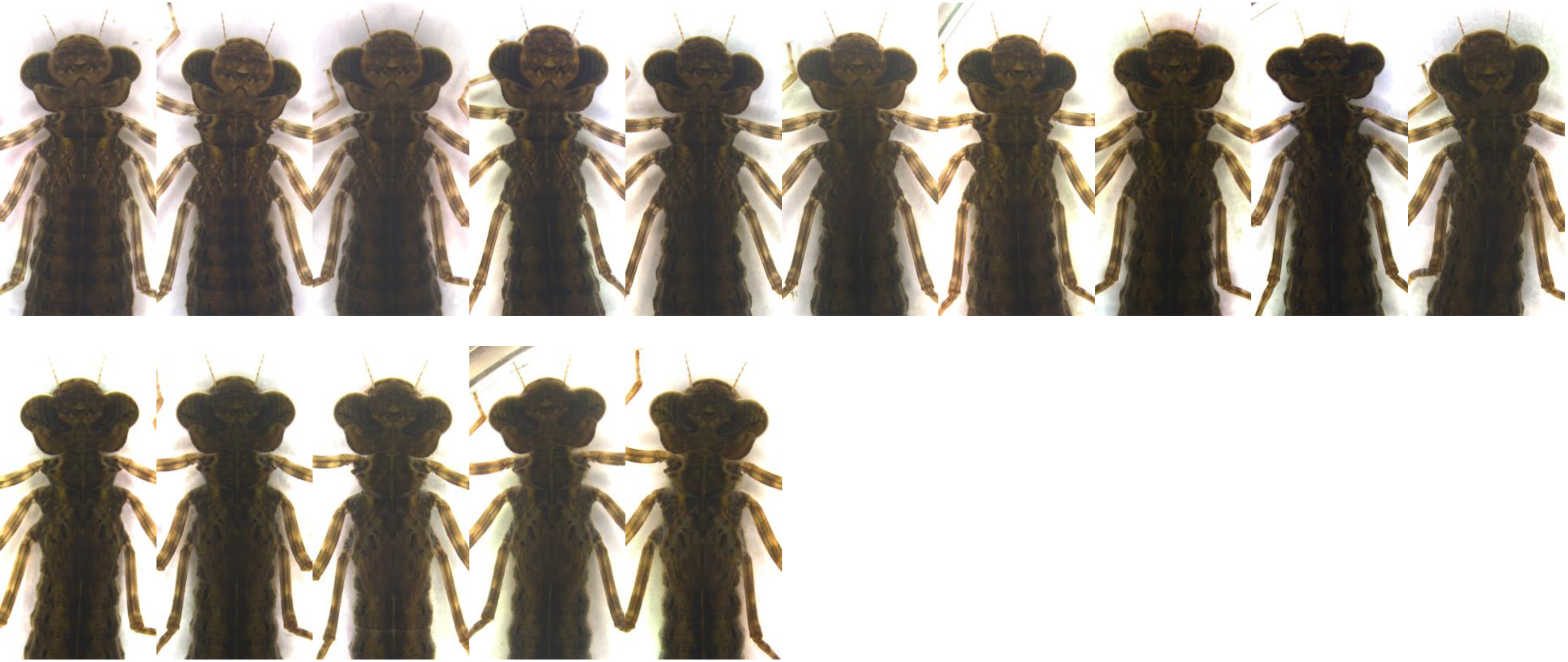

22-1 *Anax ephippiger* (1/1)

2 mm

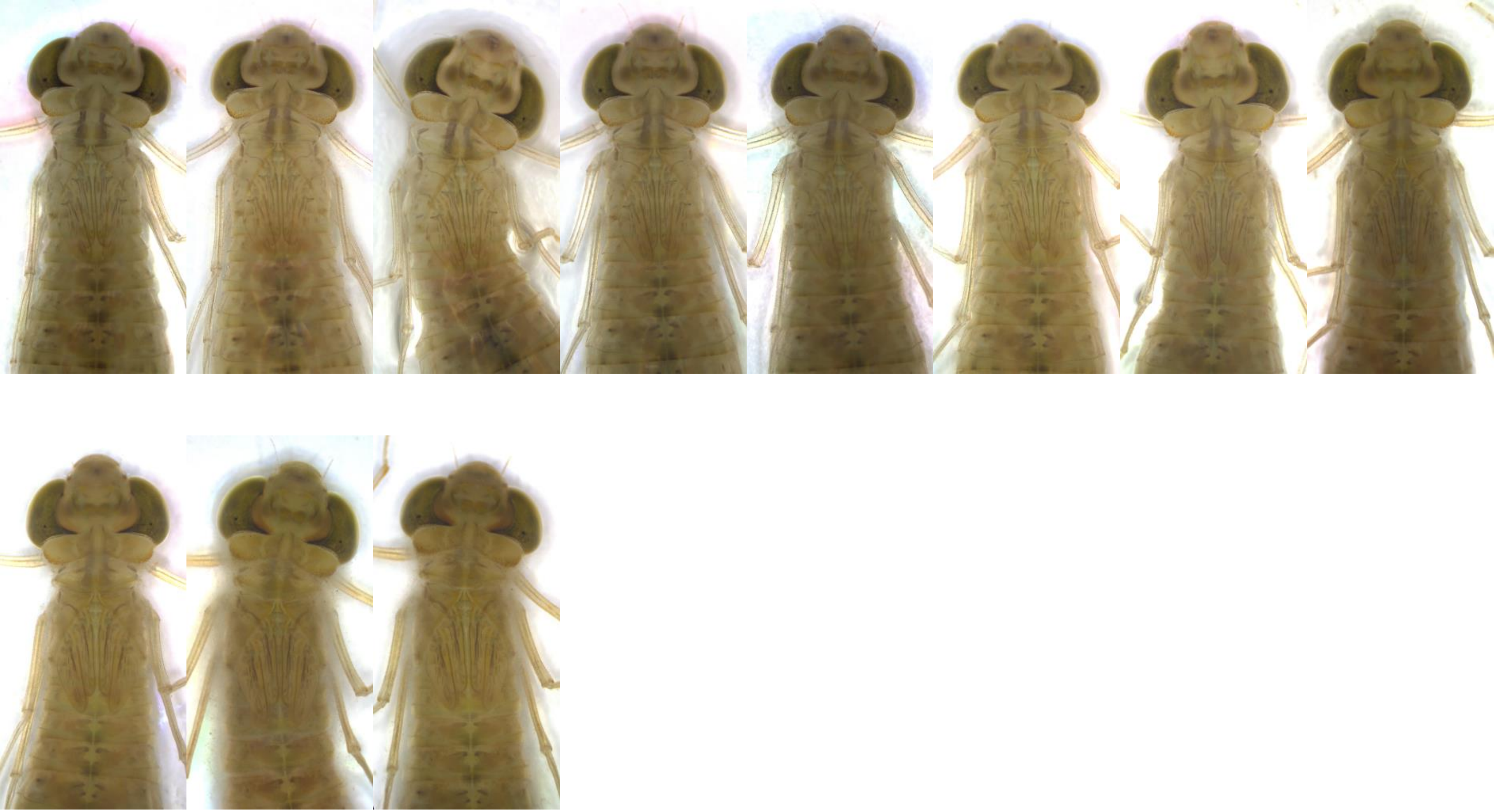

22-2 *Anax ephippiger* (1/1)

2 mm

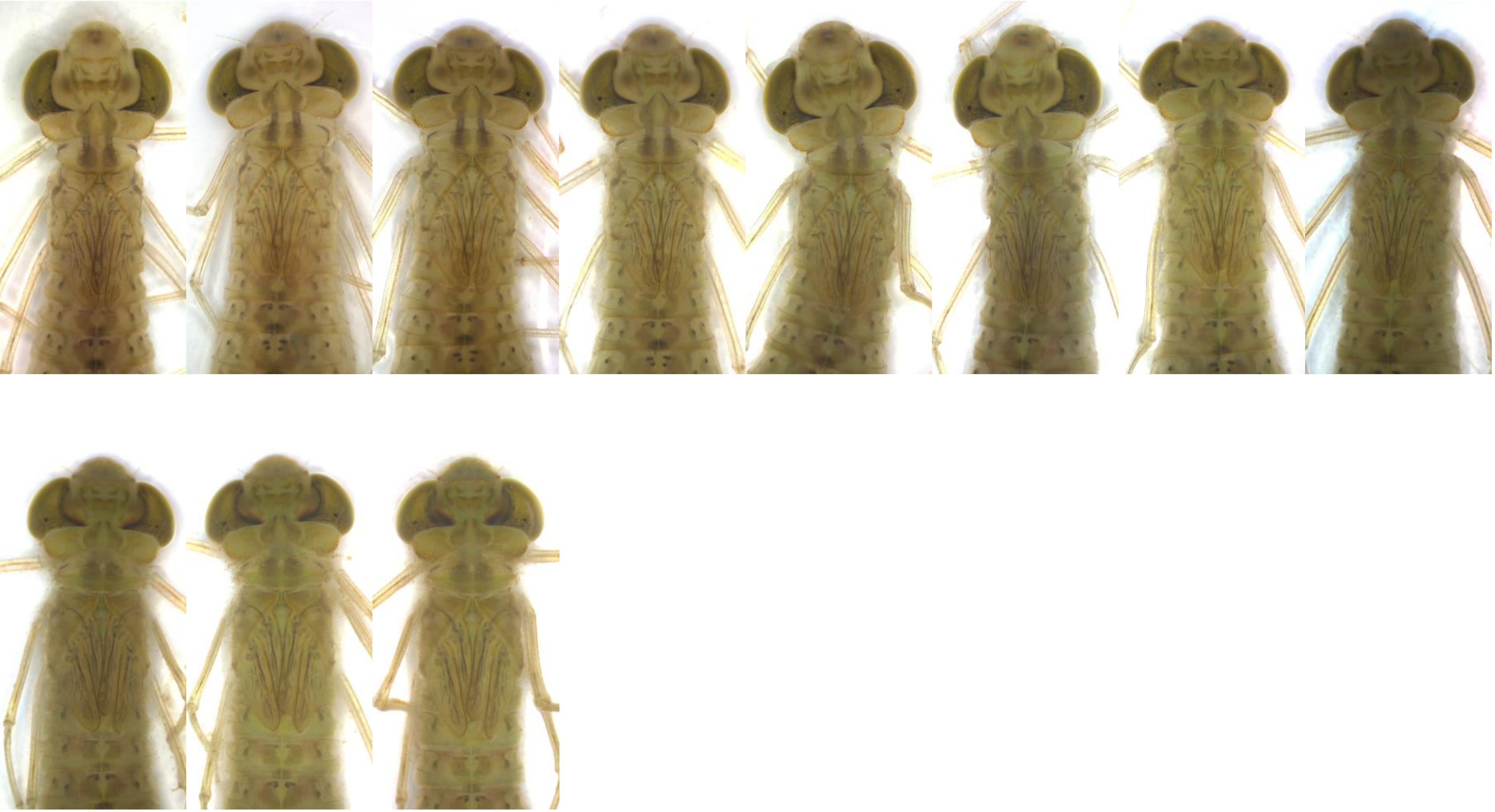

22-3 *Anax ephippiger* (1/1)

2 mm

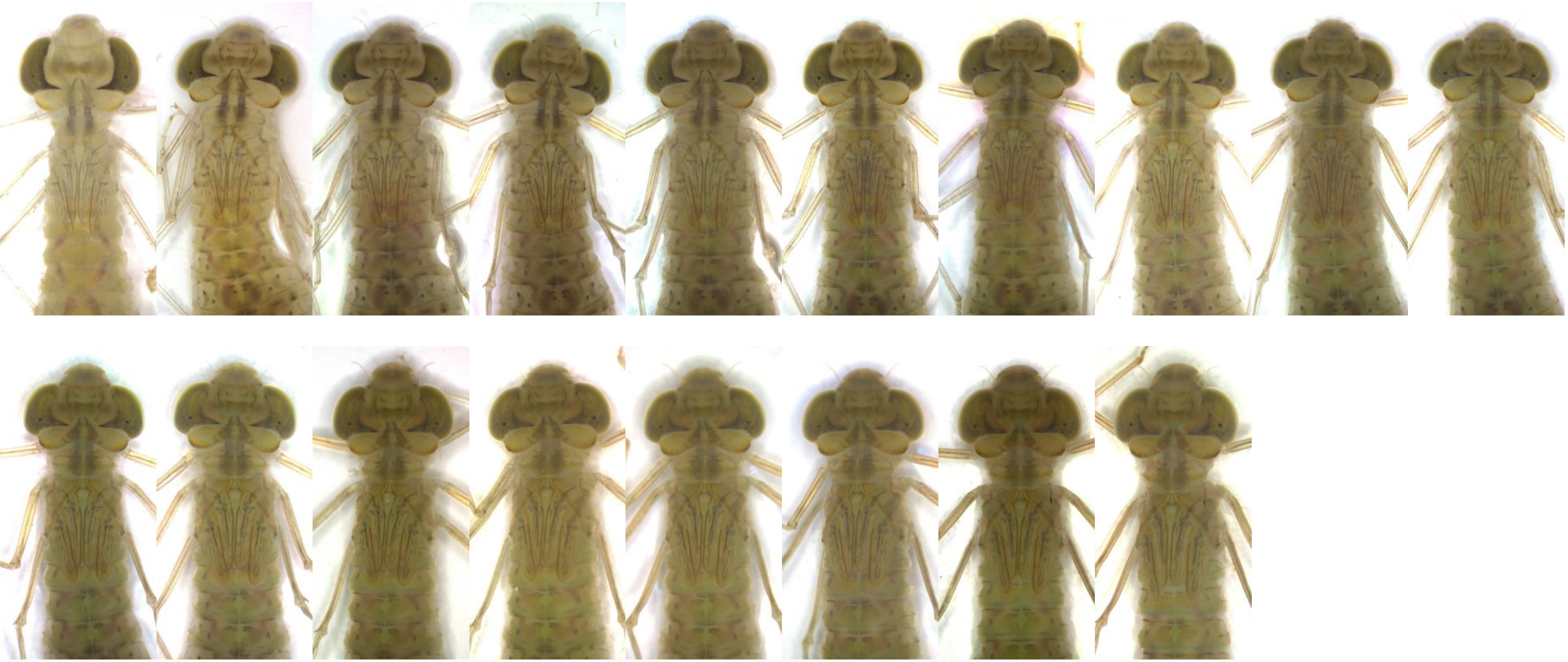

22-4 *Anax ephippiger* (1/1)

2 mm

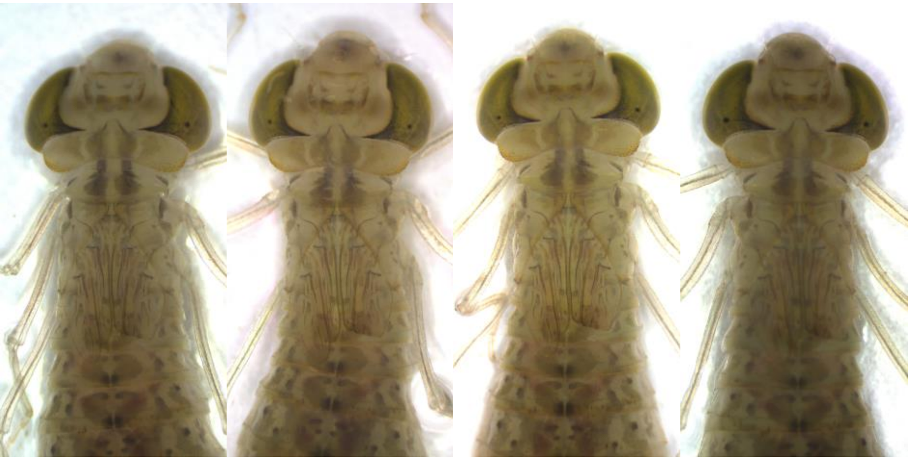

No
Data

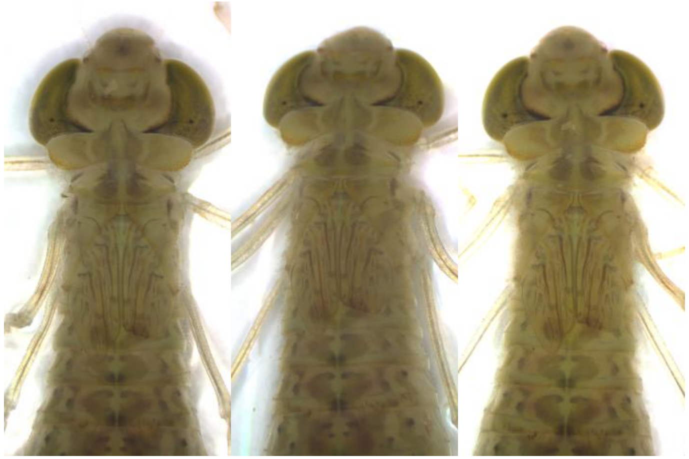

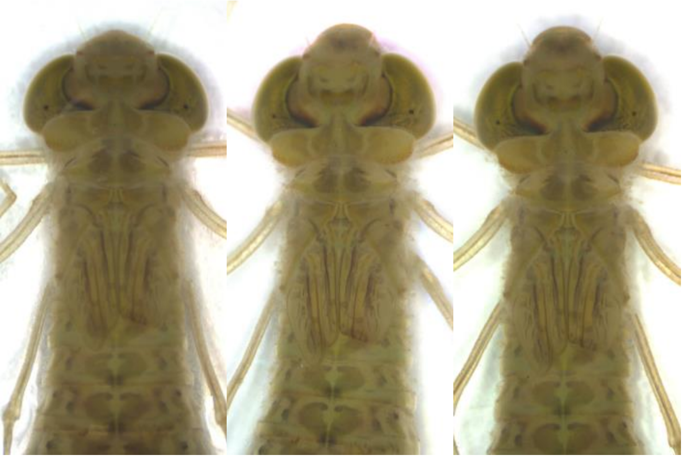

23-1 *Anax parthenope* (1/1)

2 mm

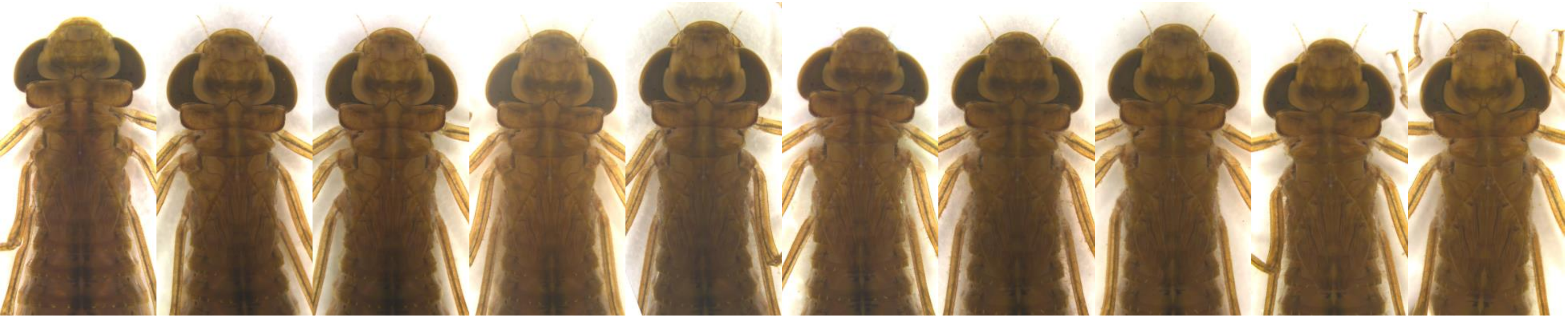

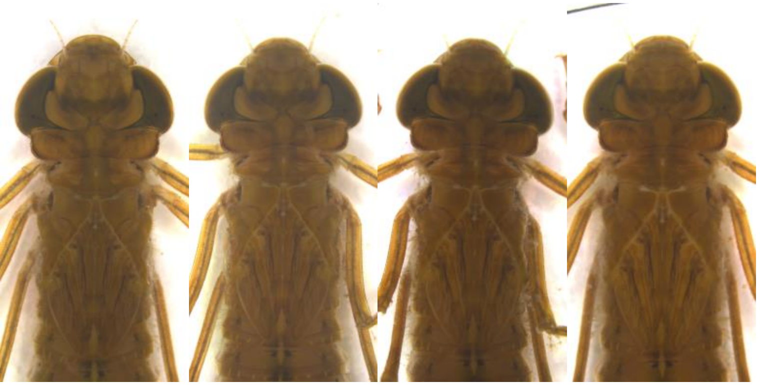

No Data

24-1 *Anax nigrofasciatus* (1/1)

2 mm

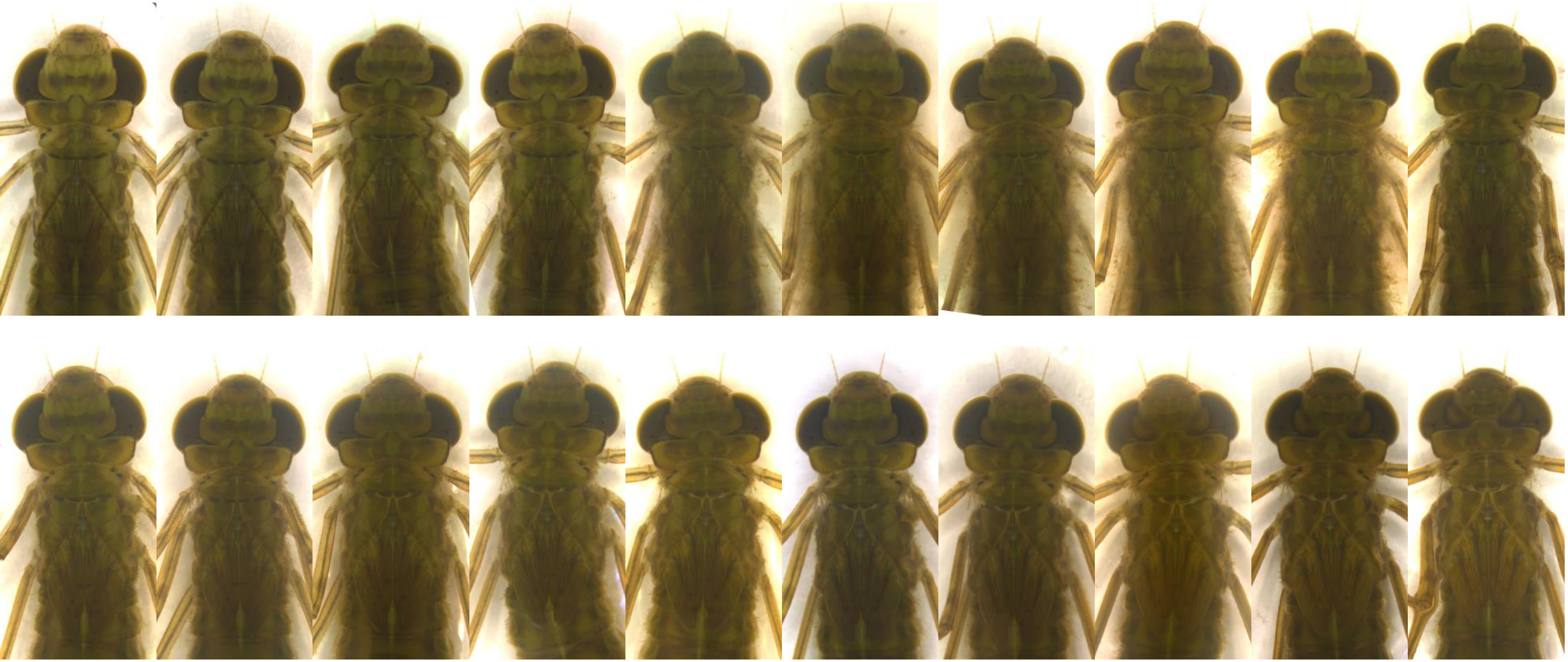

44-1 *Sympetrum uniforme* (1/1)

5 mm

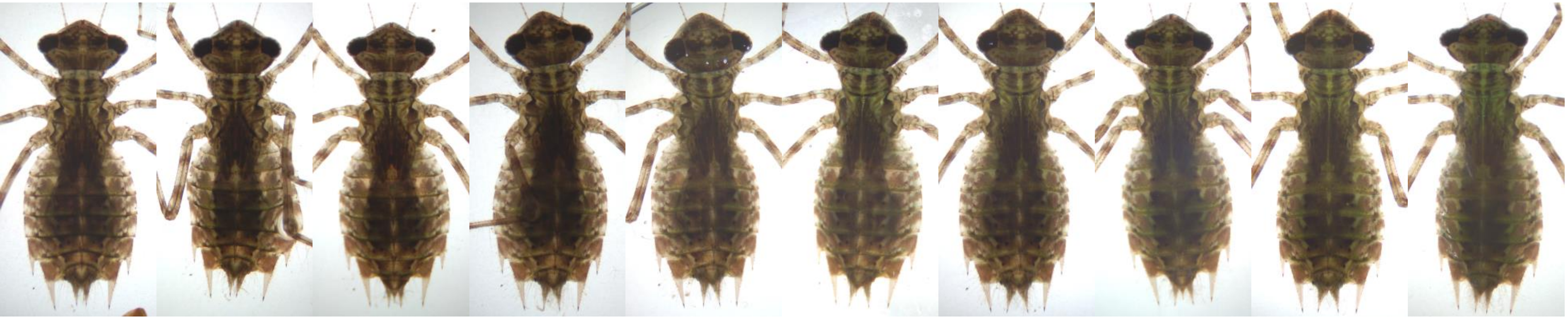

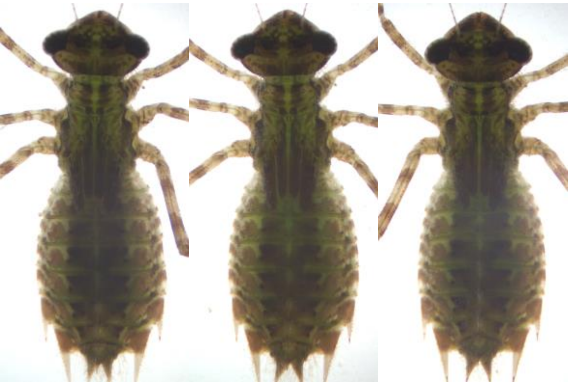

44-2 *Sympetrum uniforme* (1/1)

5 mm

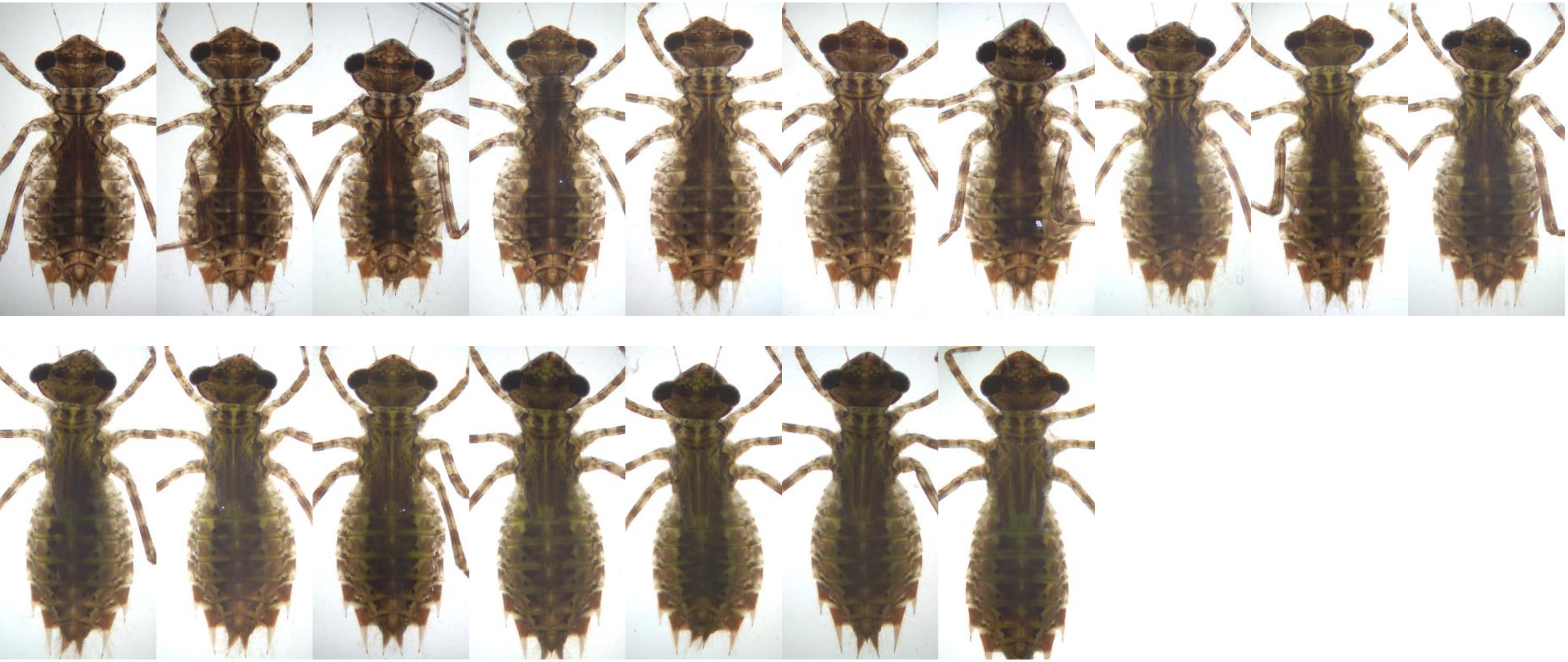

44-3 *Sympetrum uniforme* (1/1)

5 mm

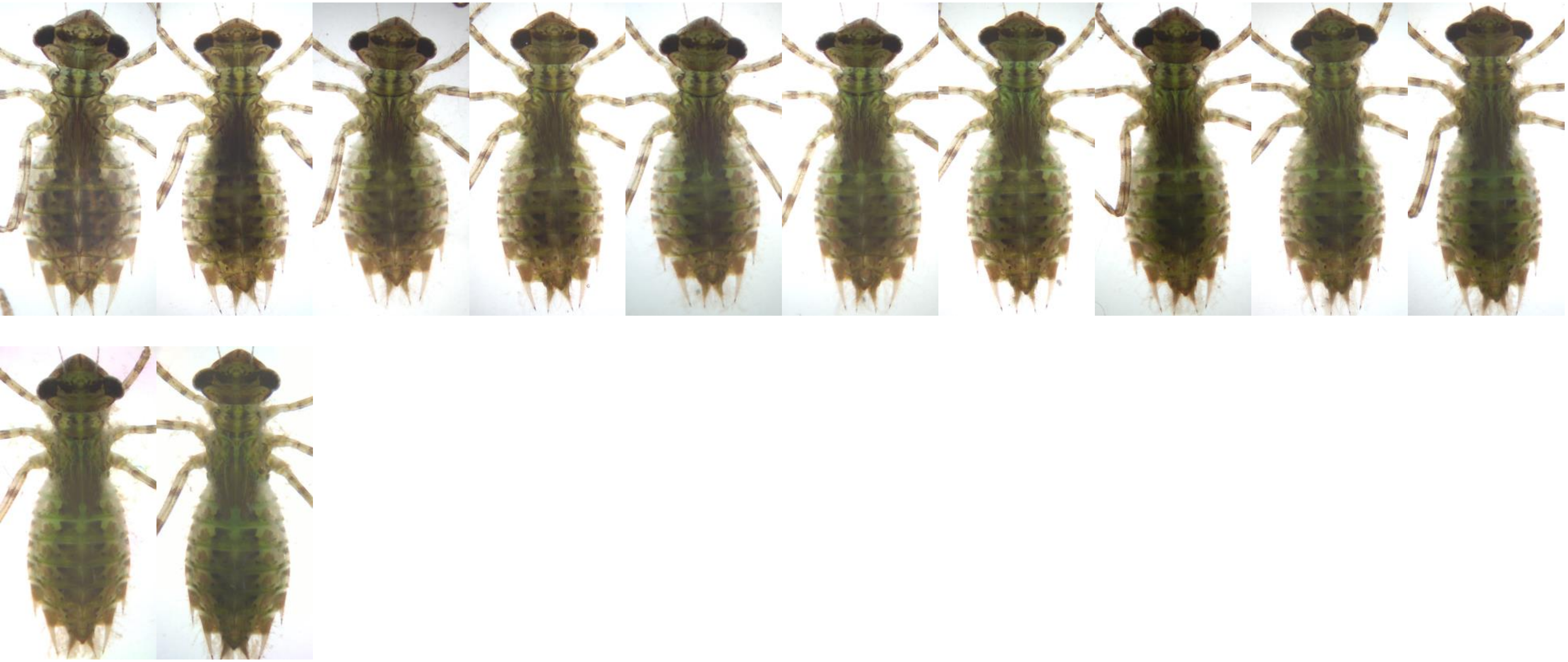

48-3 *Crocothemis servilia* (1/1)

2 mm

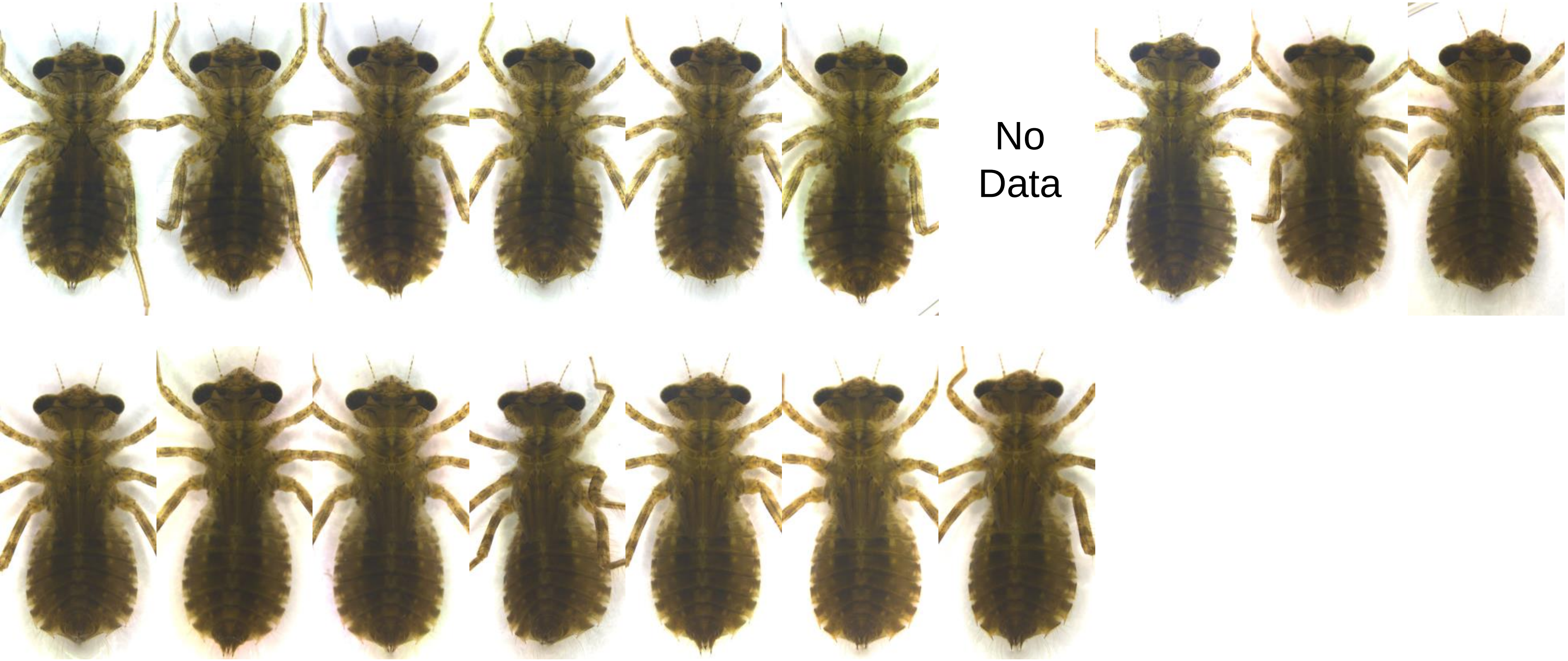

49-1 *Orthetrum albistylum* (1/1)

2 mm

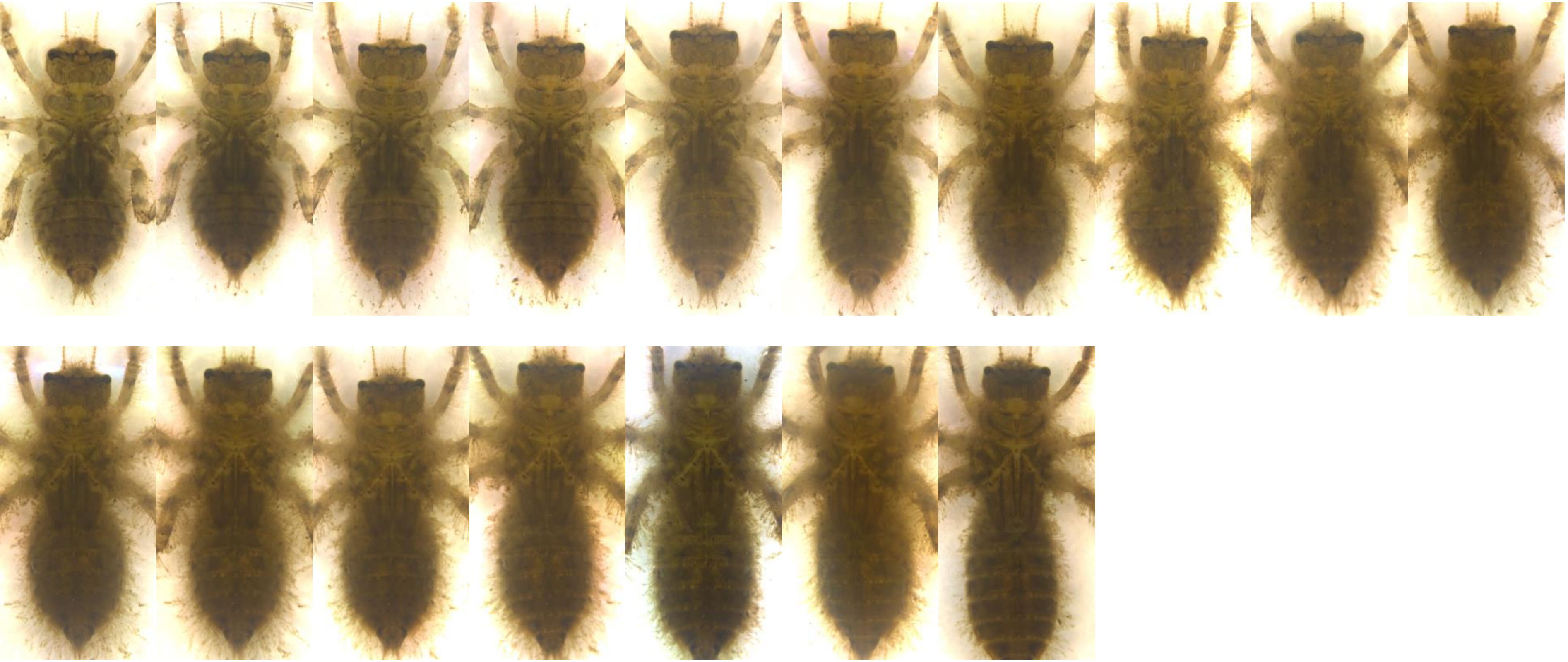

49-2 *Orthetrum albistylum* (1/1)

2 mm

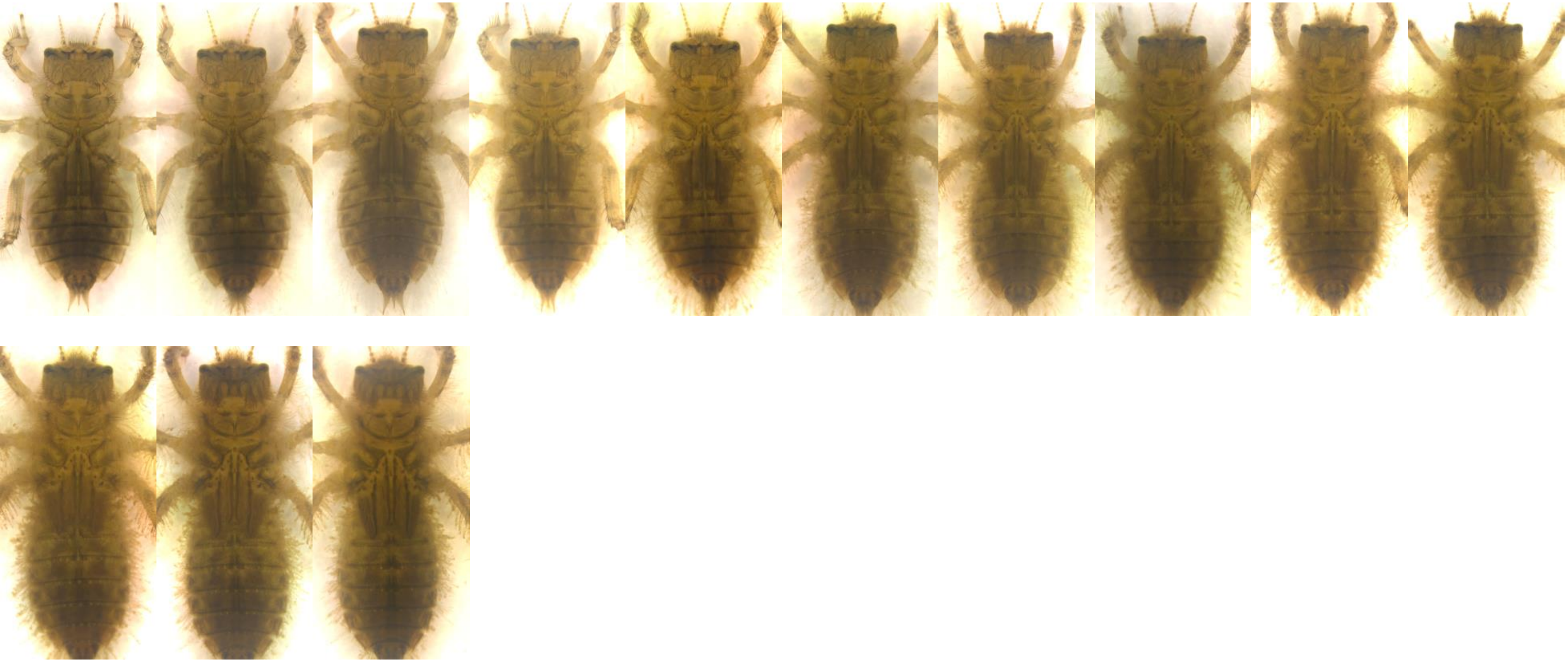

49-7 *Orthetrum albistylum* (1/1)

2 mm

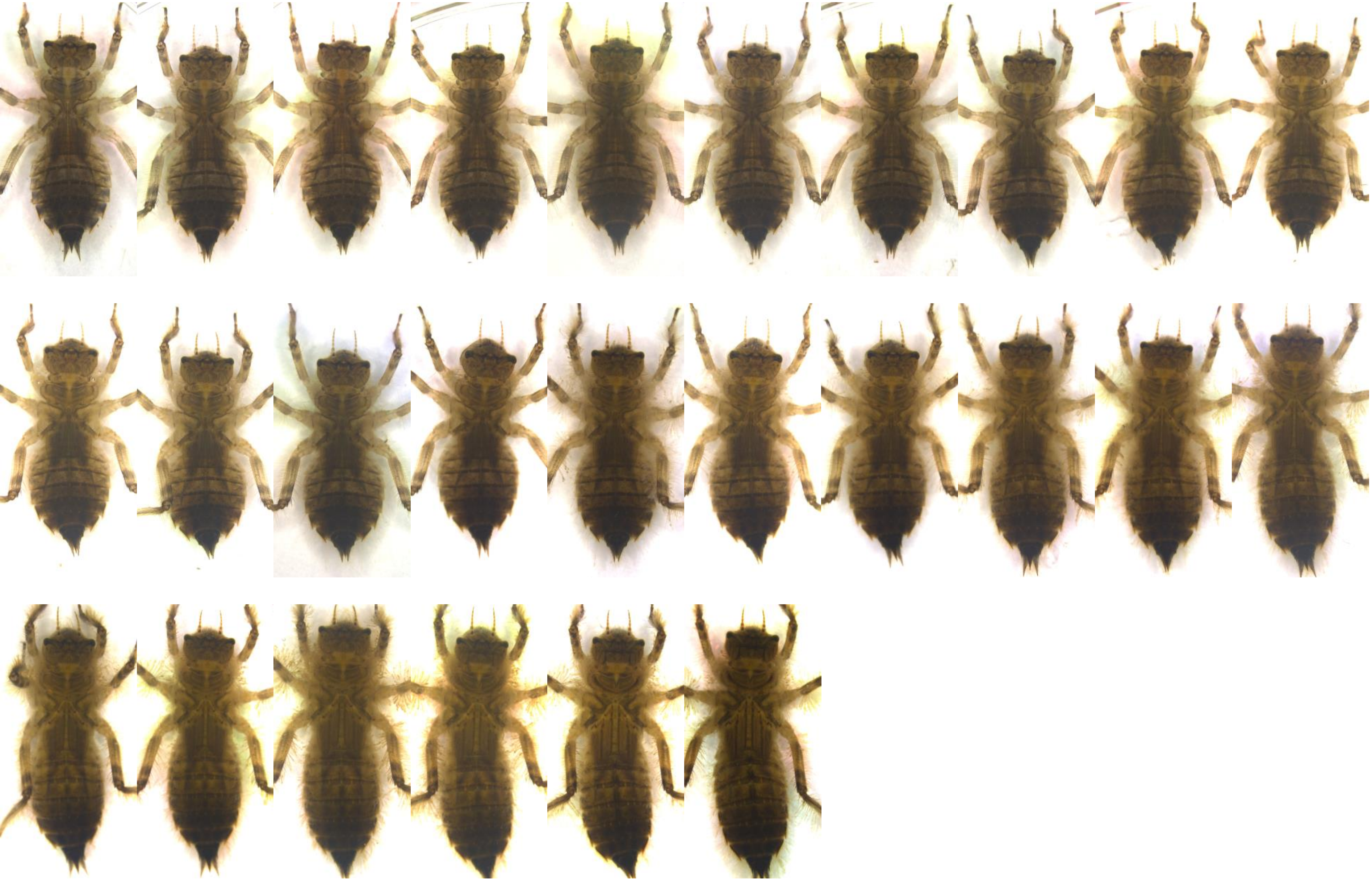

Supplement: Supplementary file 3 — Supplementary Figure S2. [file 41598_2021_84639_MOESM3_ESM.pdf]
