# Supplementary material for: Comprehensive comparative morphology and developmental staging of final instar larvae toward metamorphosis in the insect order Odonata
Source: Sci Rep. 2021 Mar 4;11:5164. doi: 10.1038/s41598-021-84639-2 (PMC7970851; doi:10.1038/s41598-021-84639-2)
Supplement: Supplementary file 4 — Supplementary Figure S3. [file 41598_2021_84639_MOESM4_ESM.pdf]

Comprehensive comparative morphology and developmental staging of final instar larvae toward metamorphosis in the insect order Odonata

Genta Okude, Takema Fukatsu, Ryo Futahashi

Figure S3

All the adjusted photos of the ventral heads focusing on the shrinking larval labium.

10-1 *Paracercion melanotum* (1/1)

2 mm

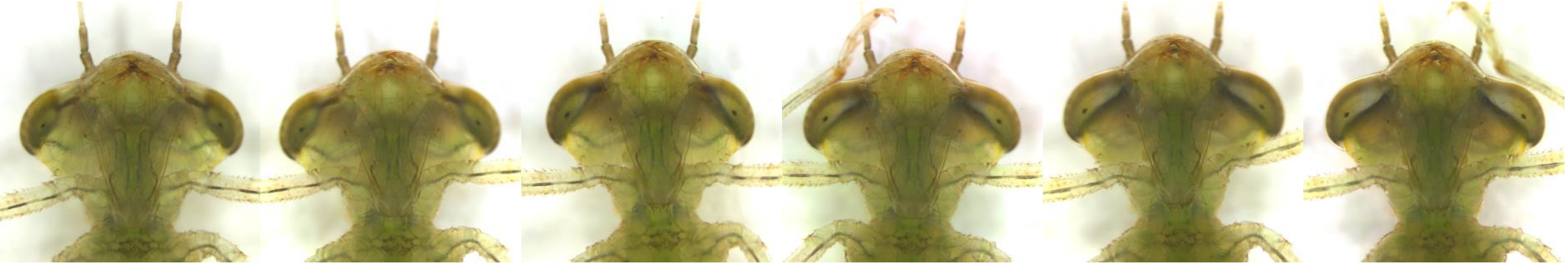

stage 2

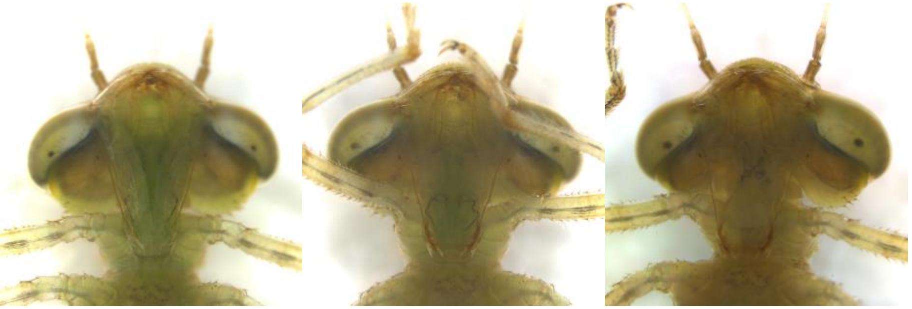

stage 3

10-2 *Paracercion melanotum* (1/1)

2 mm

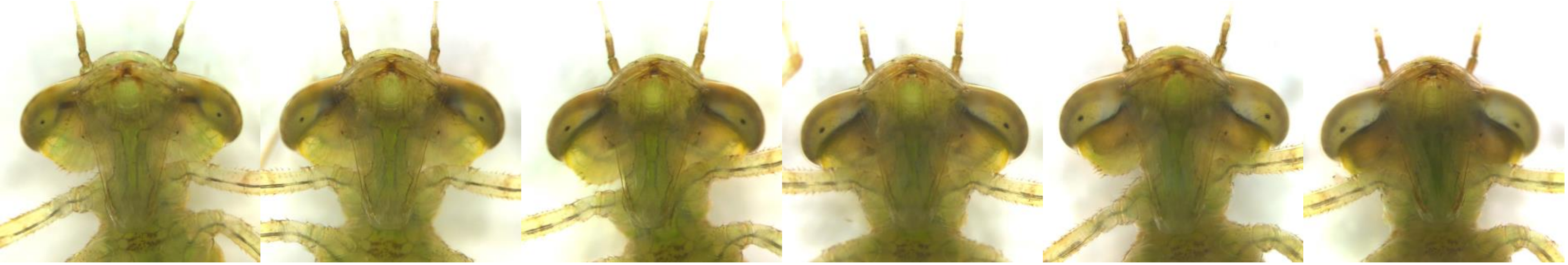

stage 2

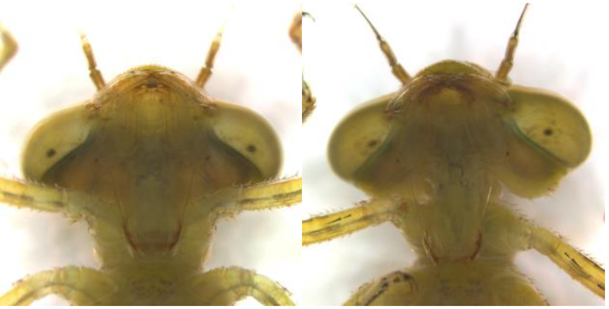

stage 3

14-1 *Ischnura senegalensis* (1/2)

1 mm

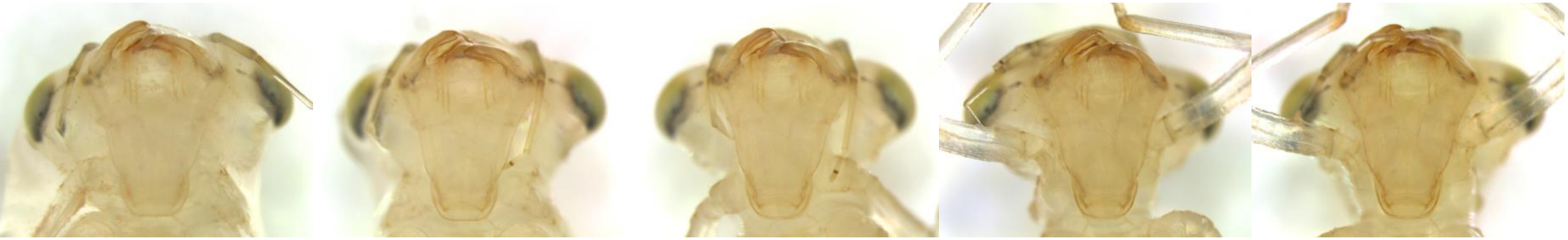

stage 1

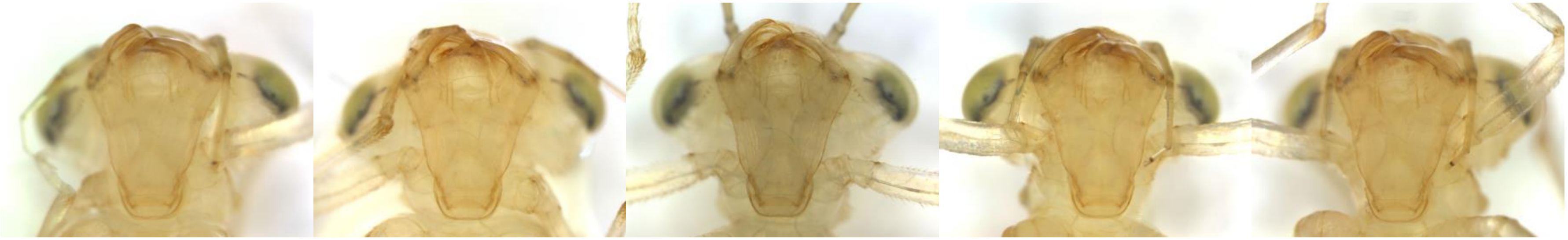

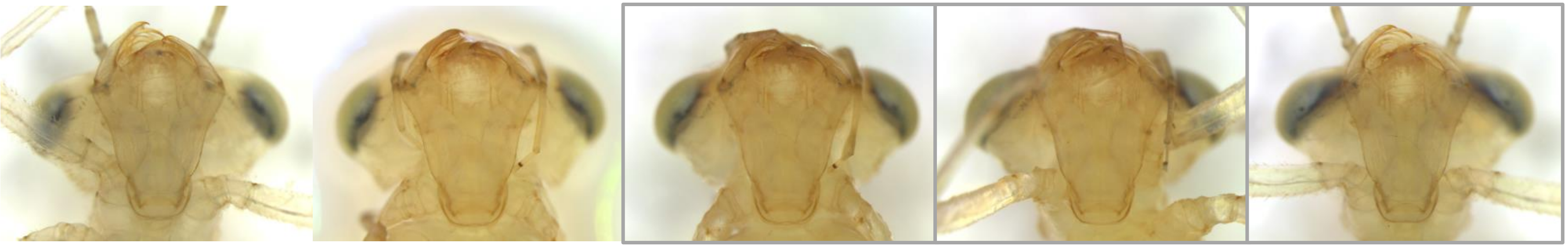

stage 2

14-1 *Ischnura senegalensis* (2/2)

1 mm

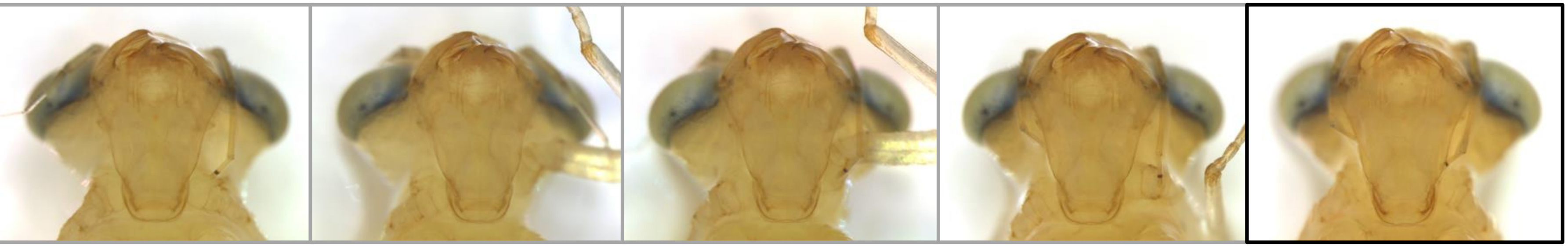

stage 3

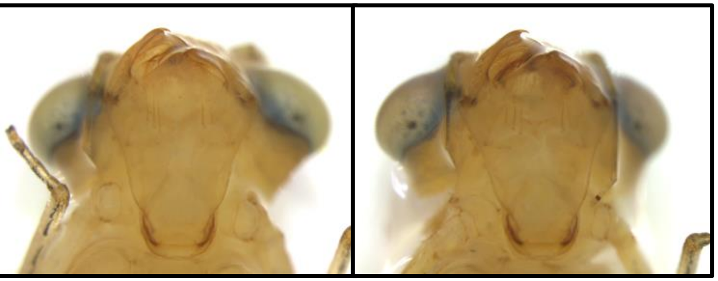

14-2 *Ischnura senegalensis* (1/2)

1 mm

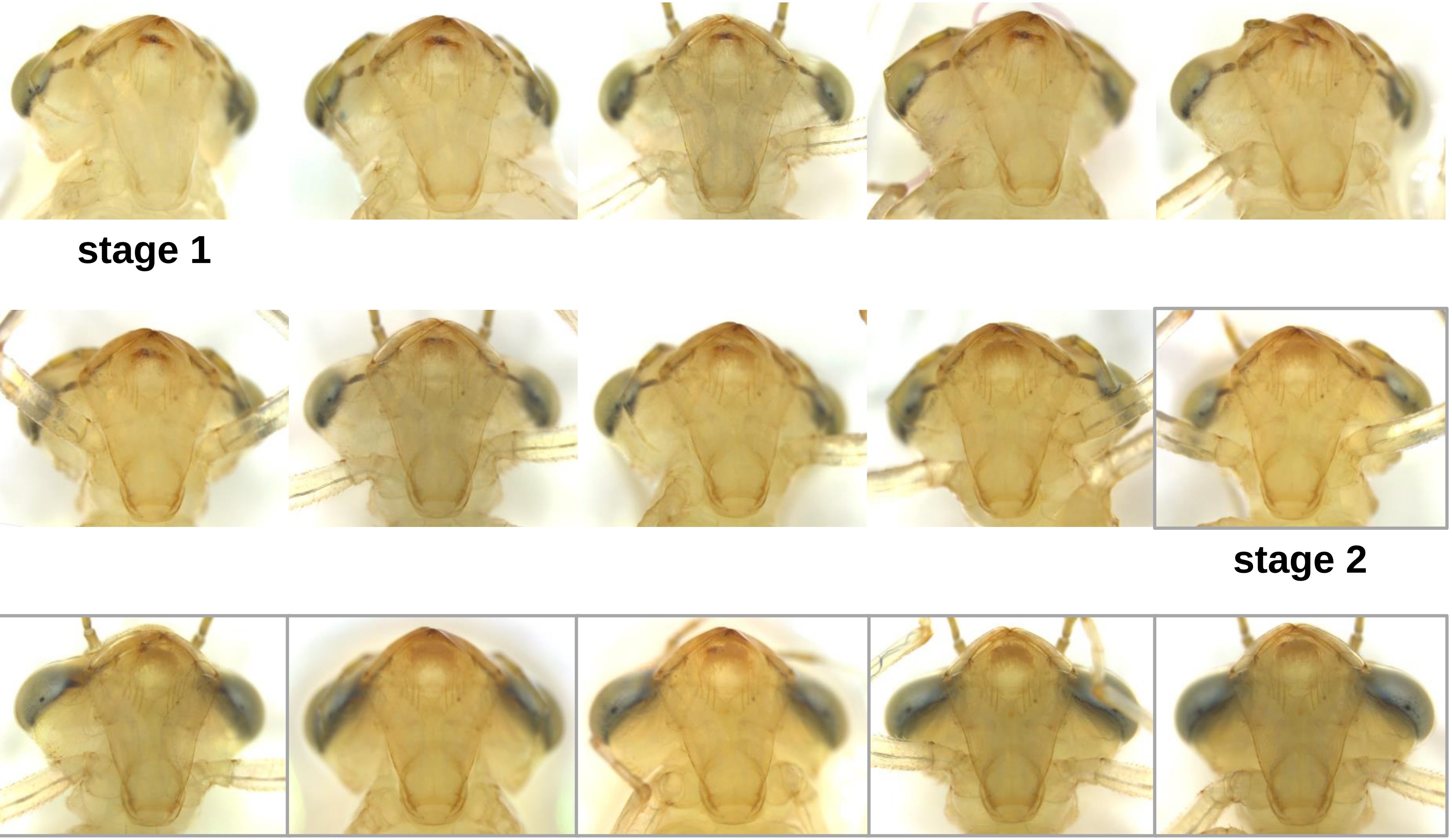

14-2 *Ischnura senegalensis* (2/2)

1 mm

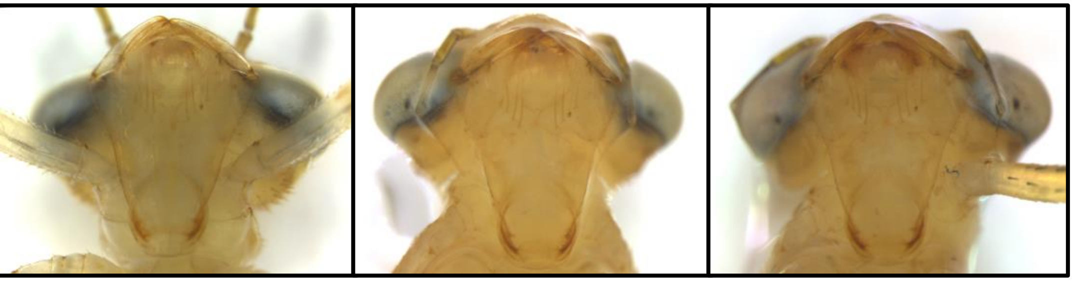

stage 3

22-4 *Anax ephippiger* (1/2)

2 mm

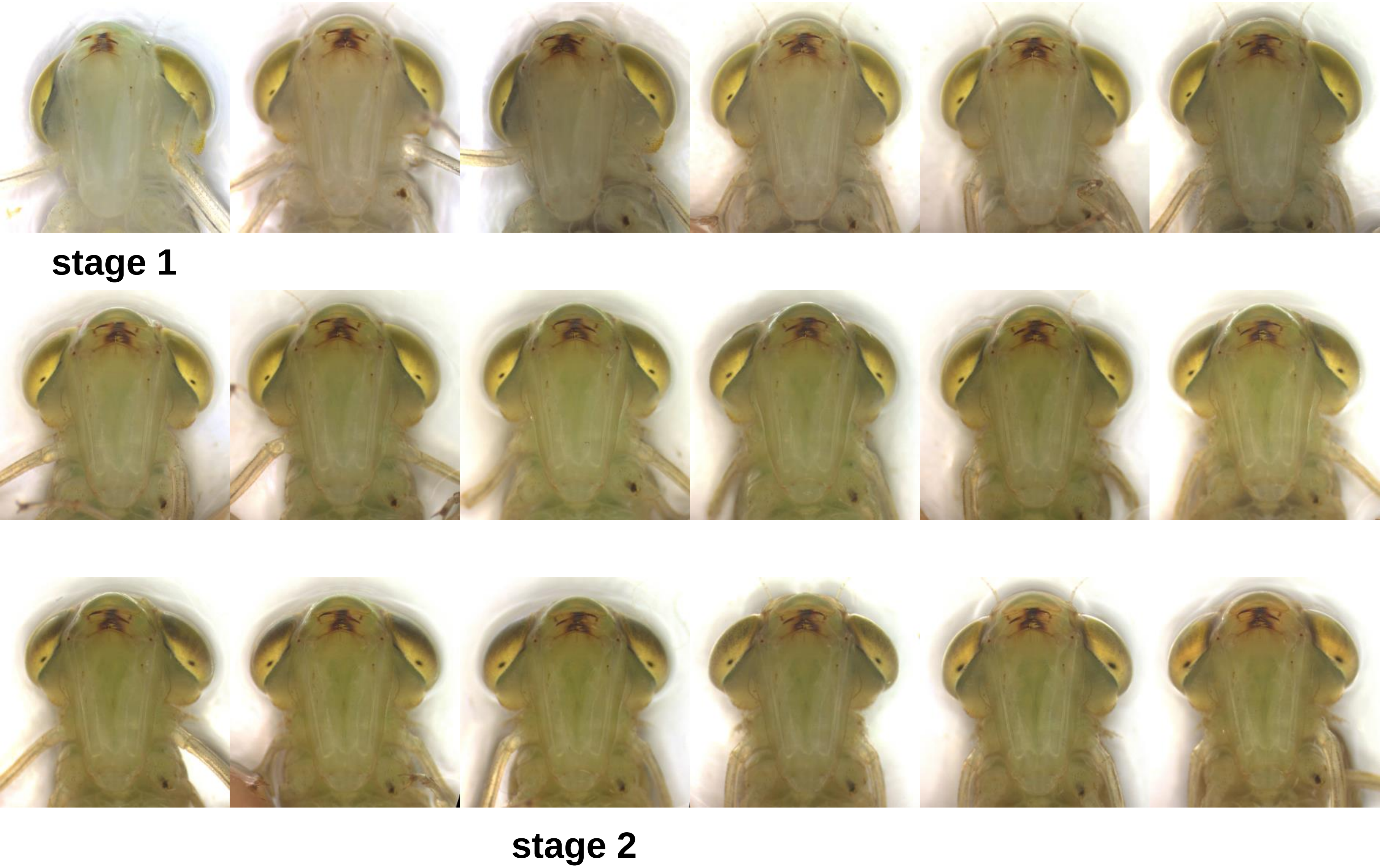

22-4 *Anax ephippiger* (2/2)

2 mm

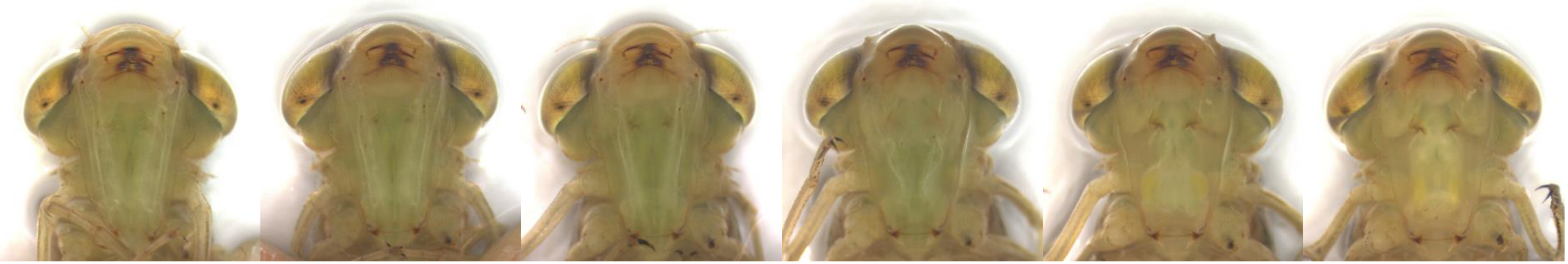

stage 3

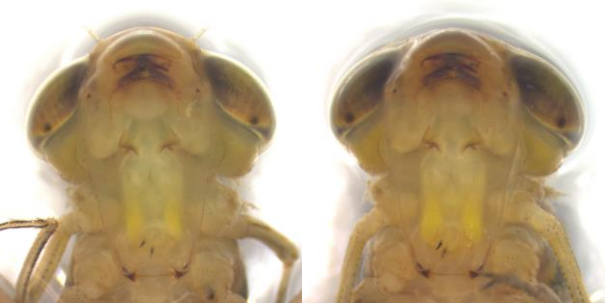

22-5 *Anax ephippiger* (1/2)

2 mm

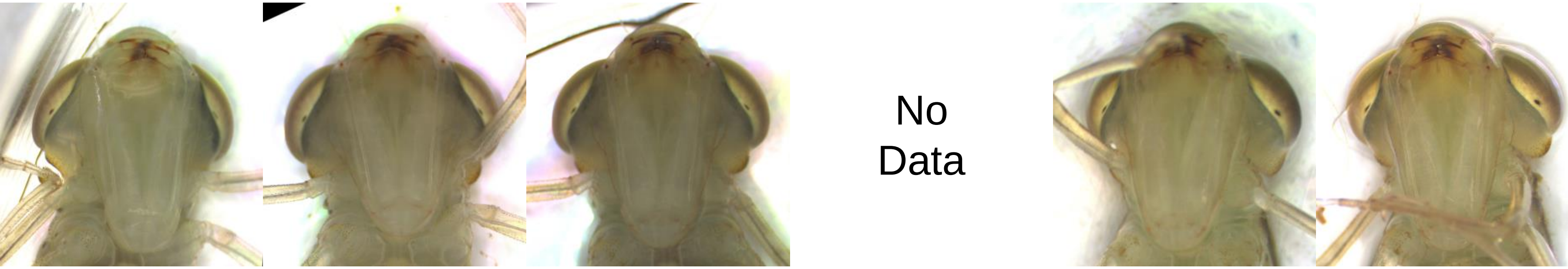

stage 1

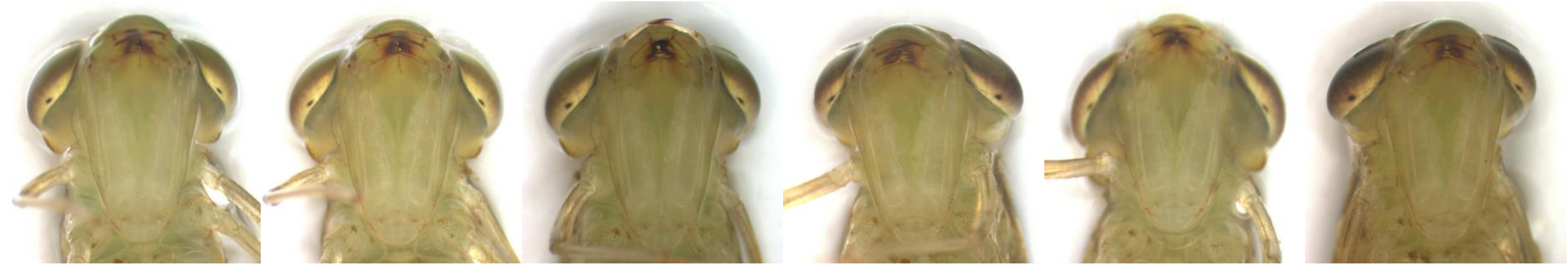

stage 2

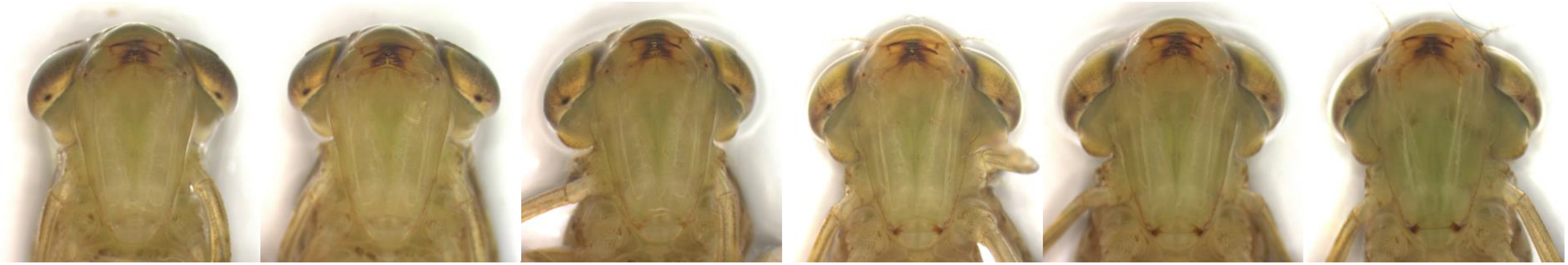

22-5 *Anax ephippiger* (2/2)

2 mm

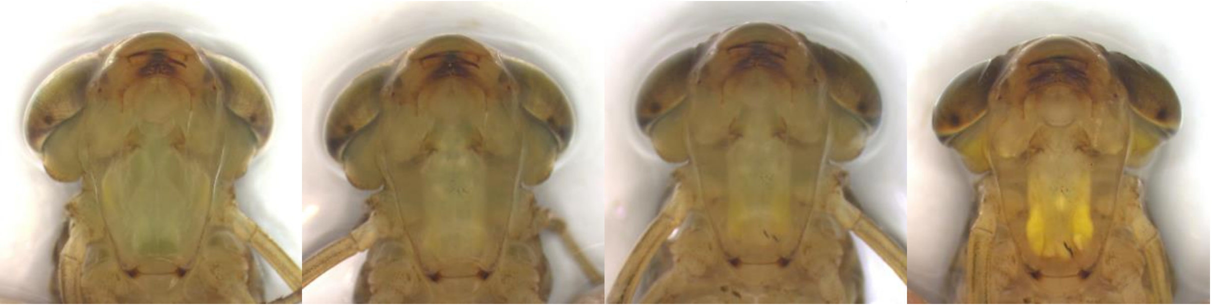

stage 3

22-6 *Anax ephippiger* (1/2)

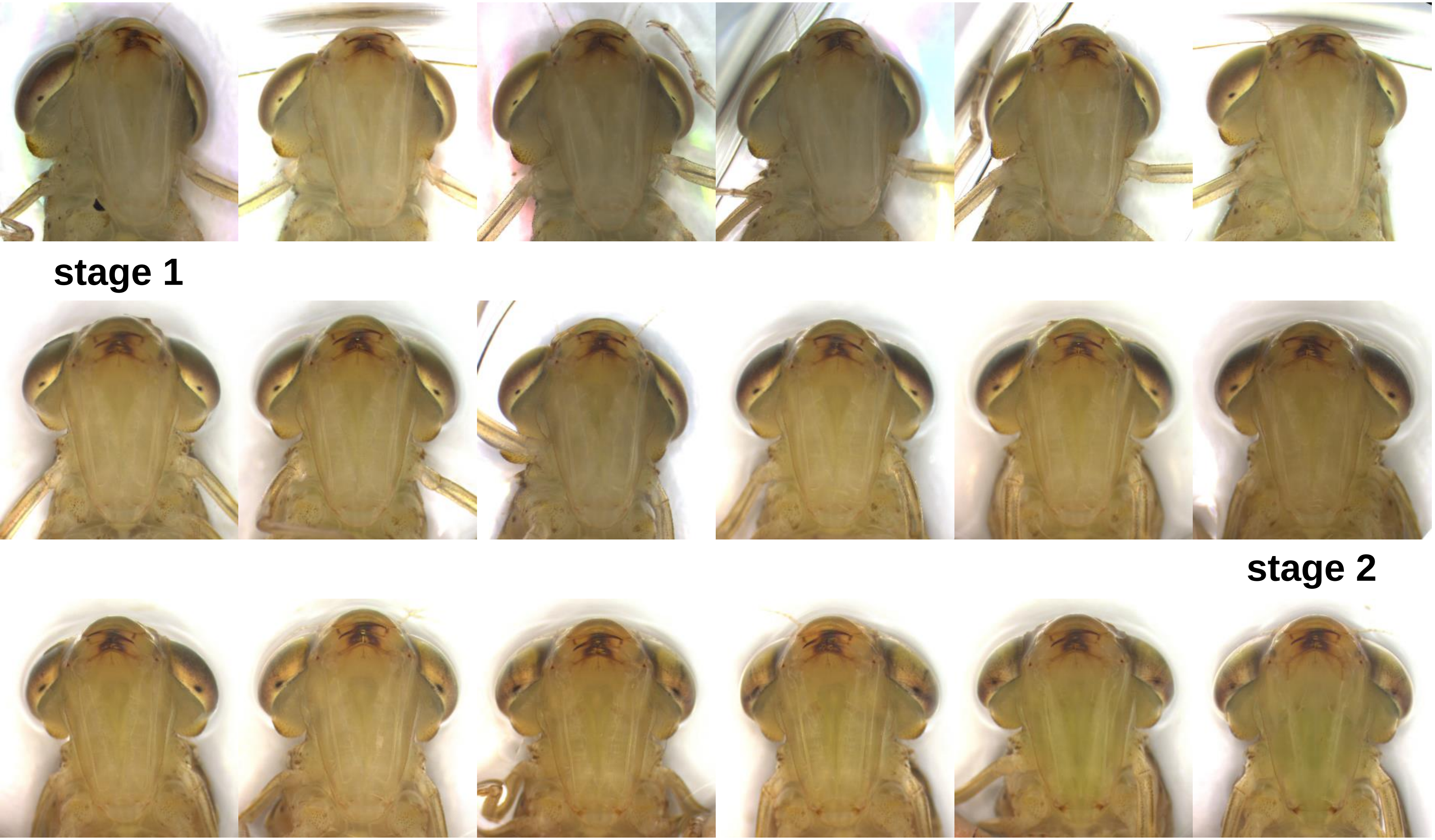

22-6 *Anax ephippiger* (2/2)

2 mm

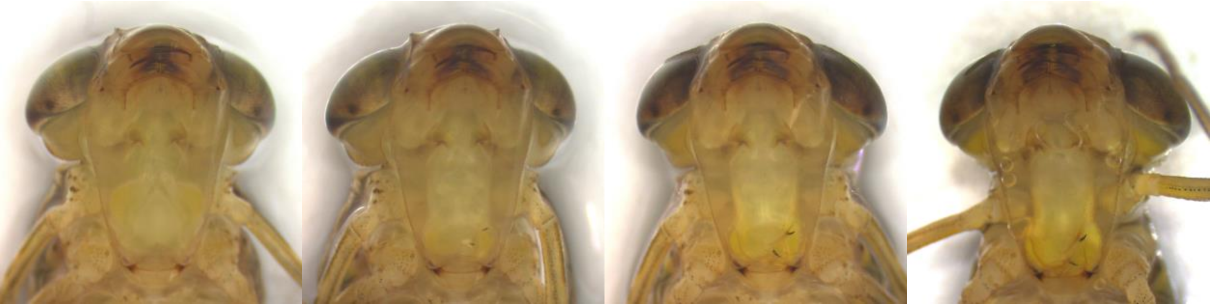

stage 3

22-7 *Anax ephippiger* (1/2)

2 mm

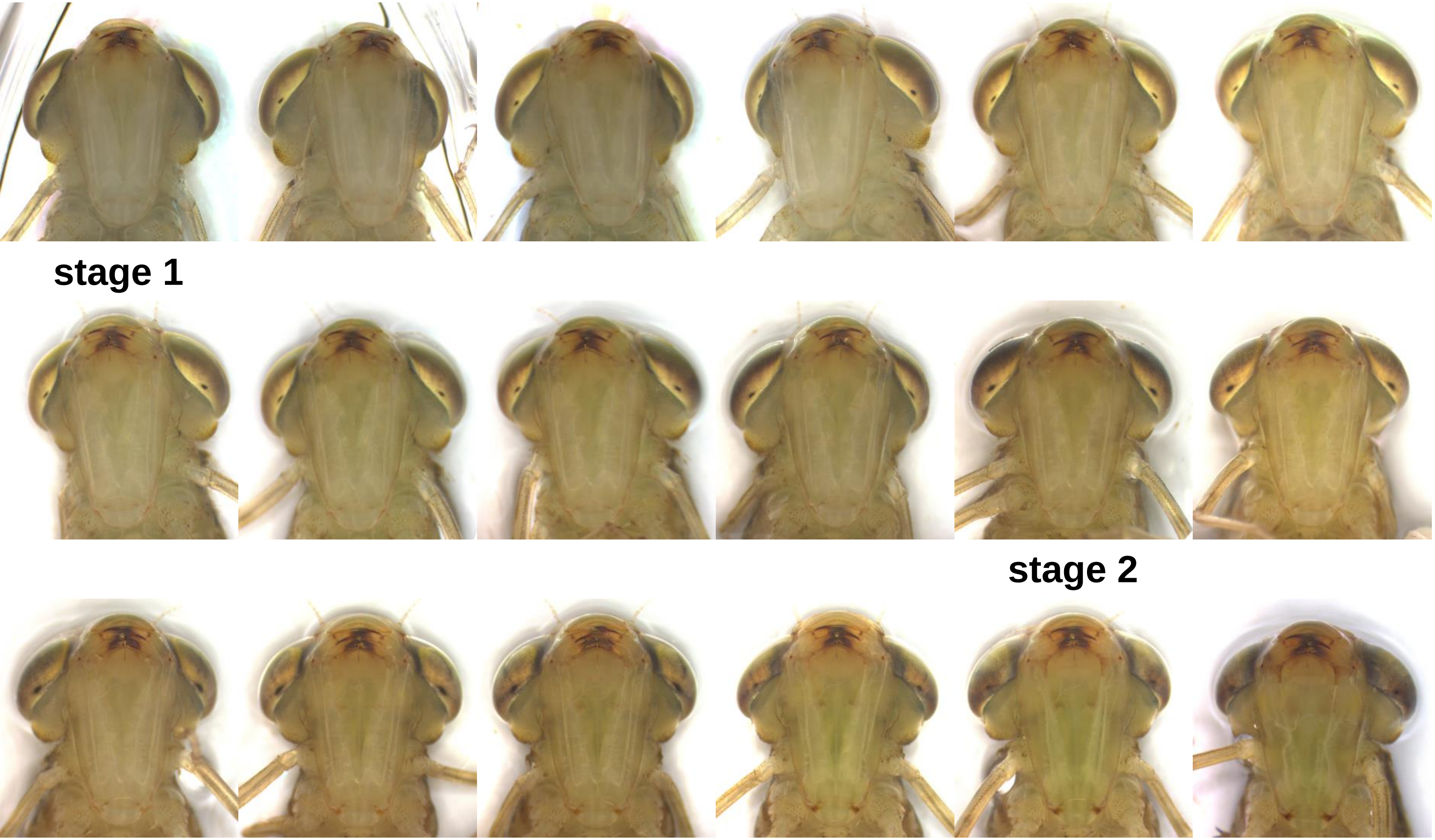

22-7 *Anax ephippiger* (2/2)

2 mm

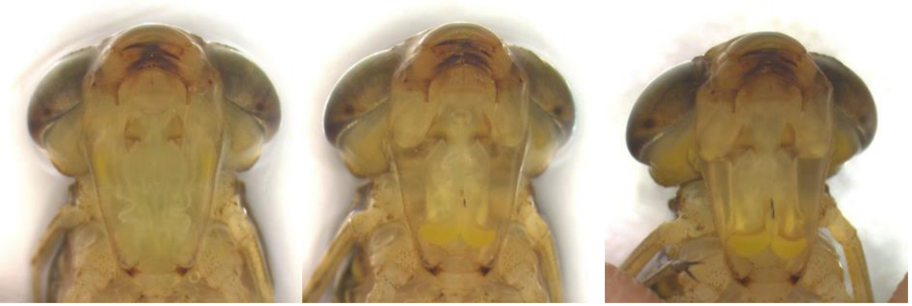

stage 3

22-8 *Anax ephippiger* (1/2)

2 mm

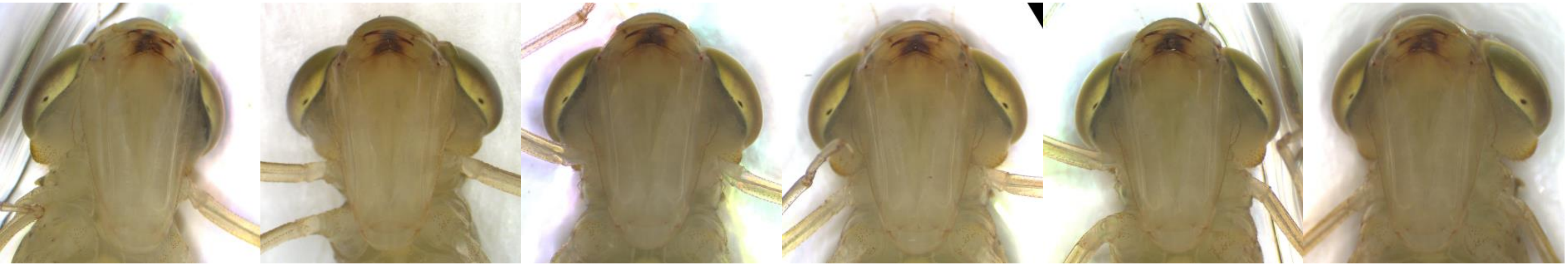

stage 1

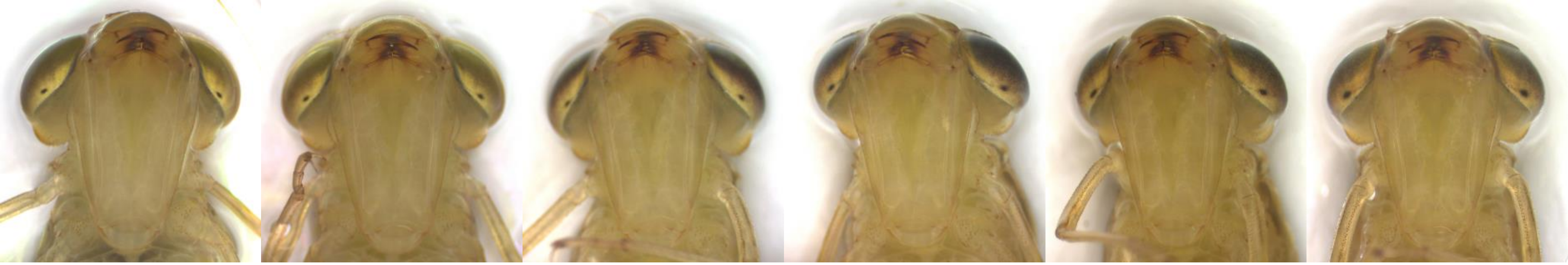

stage 2

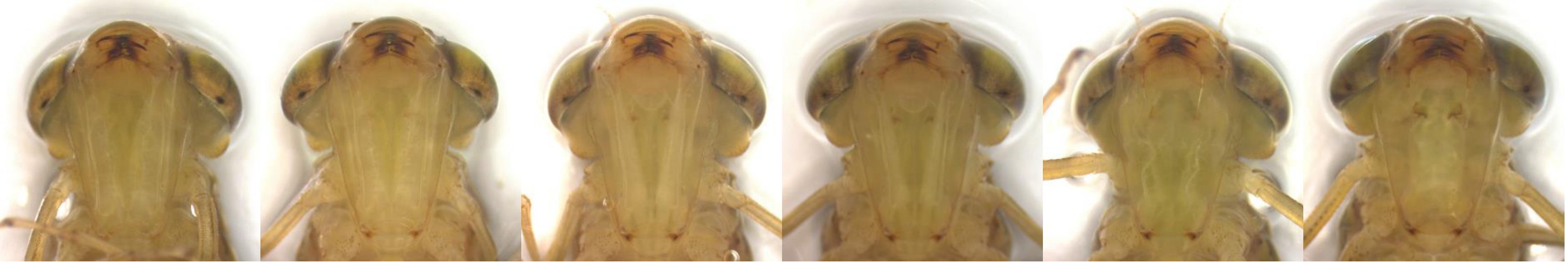

stage 3

22-8 *Anax ephippiger* (2/2)

2 mm

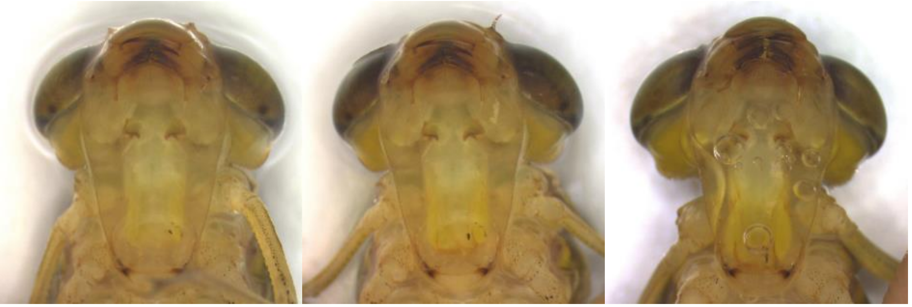

32-1 *Tanypteryx pryeri* (1/1)

2 mm

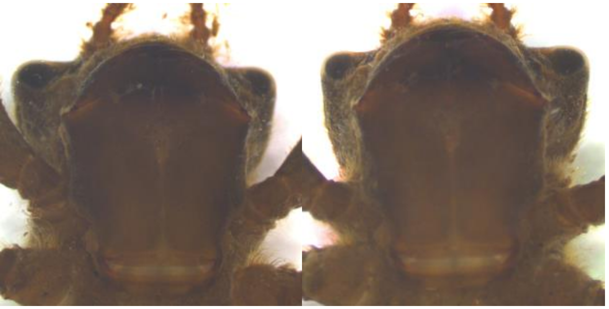

No Data

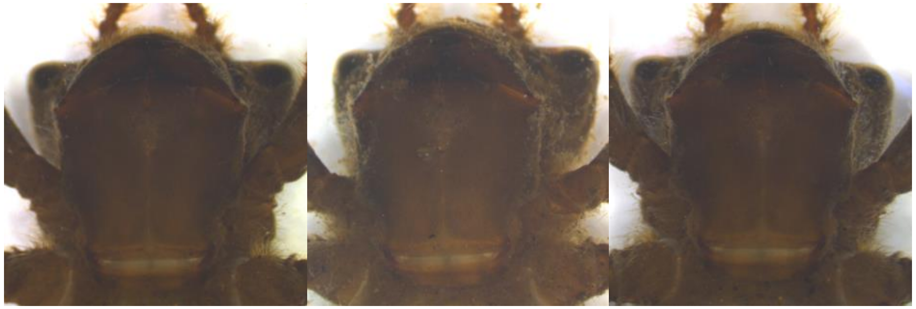

stage 2

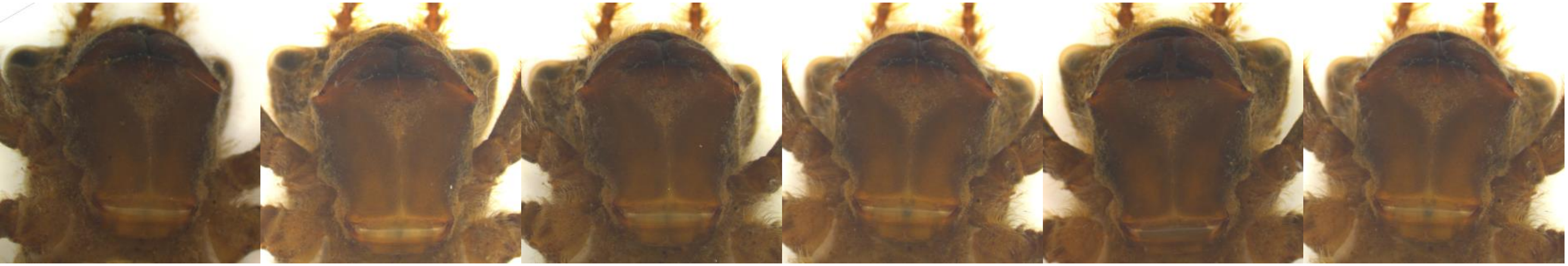

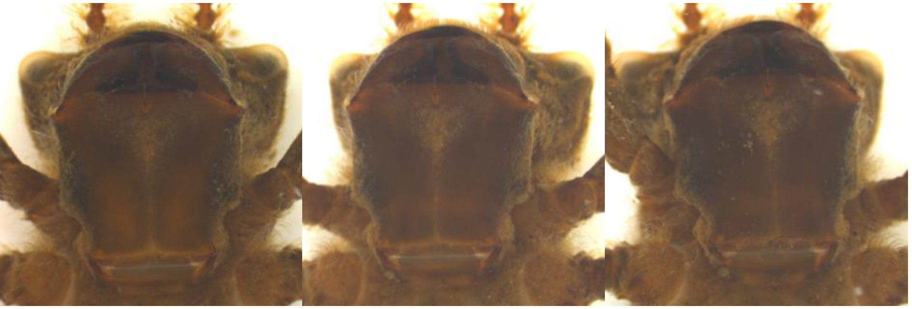

No Data

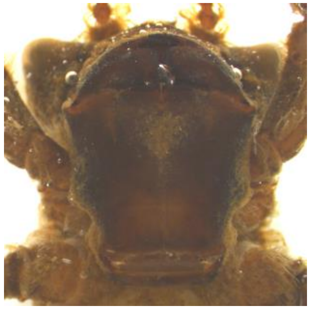

stage 3

32-2 *Tanypteryx pryeri* (1/1)

2 mm

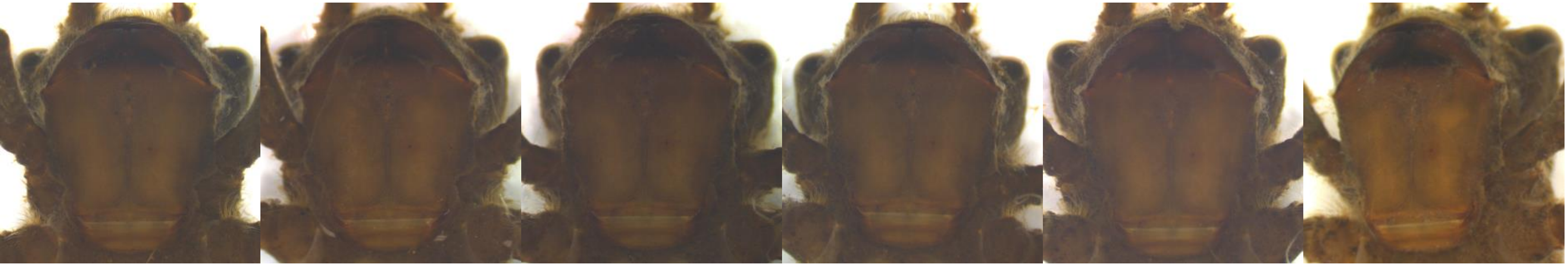

stage 2

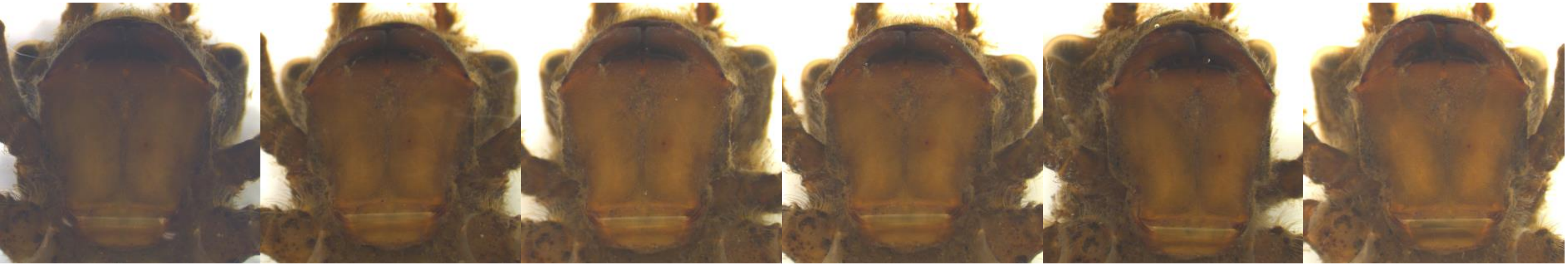

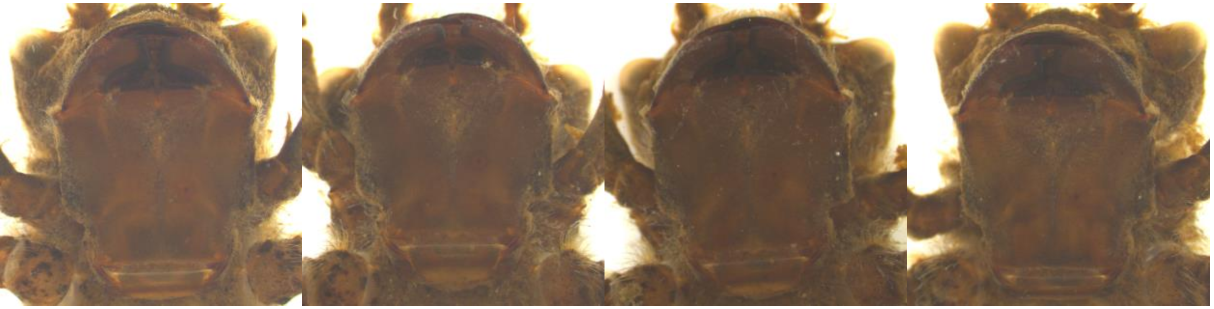

stage 3

32-3 *Tanypteryx pryeri* (1/1)

2 mm

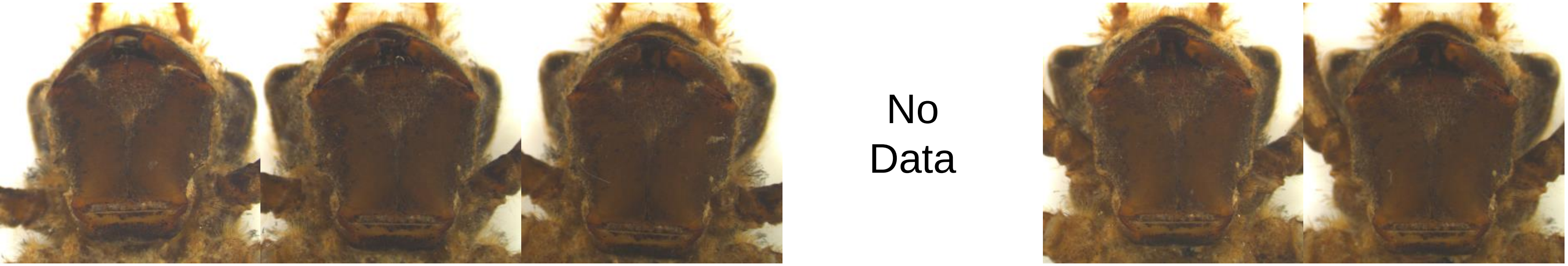

stage 2

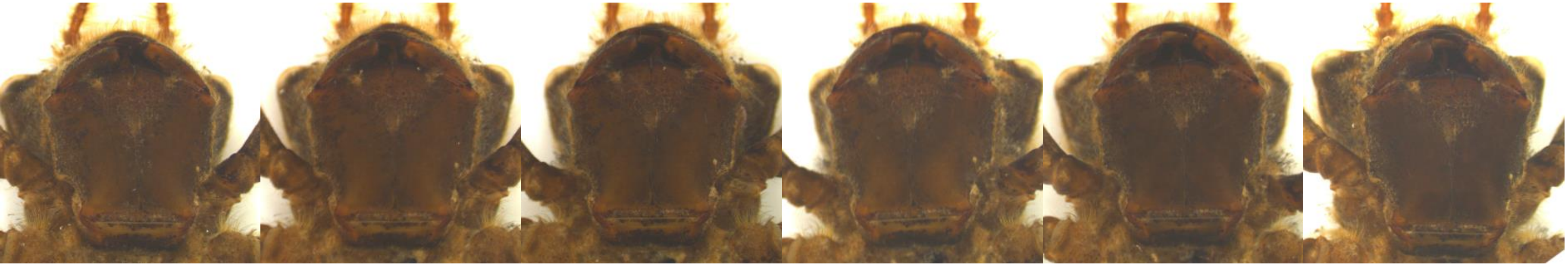

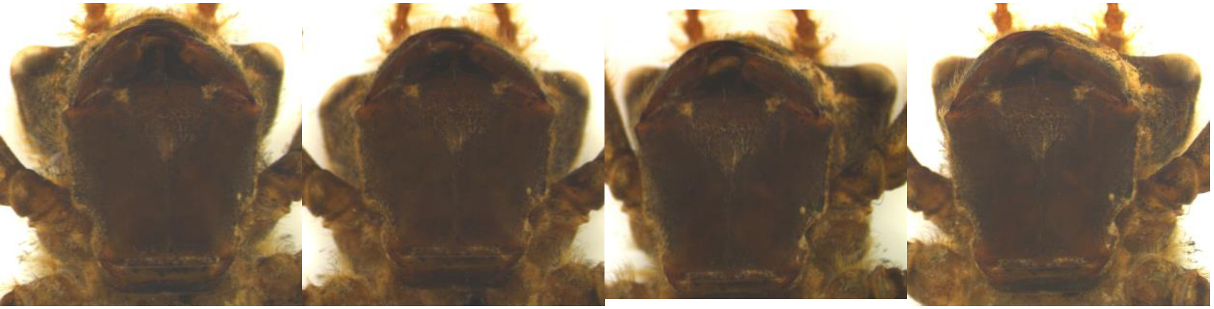

stage 3

33-1 *Chlorogomphus brunneus* (1/1)

2 mm

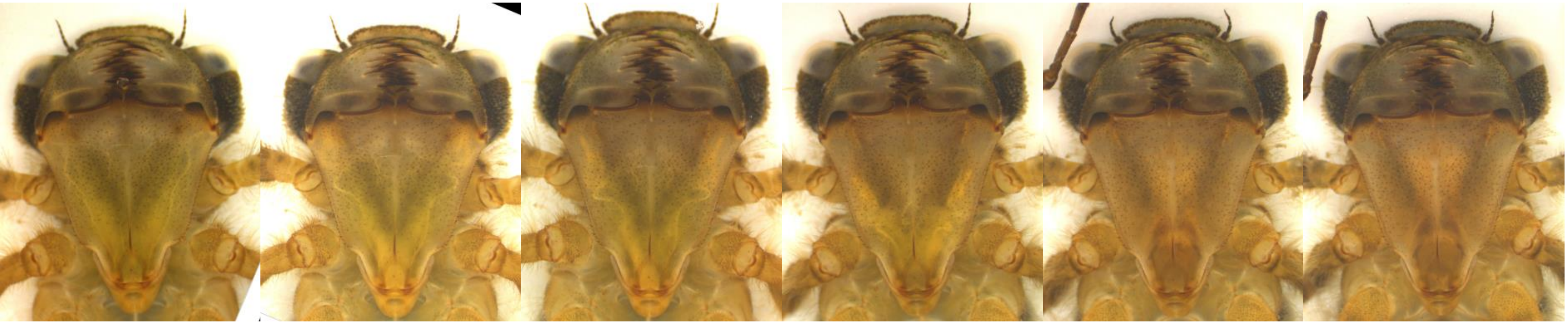

stage 3

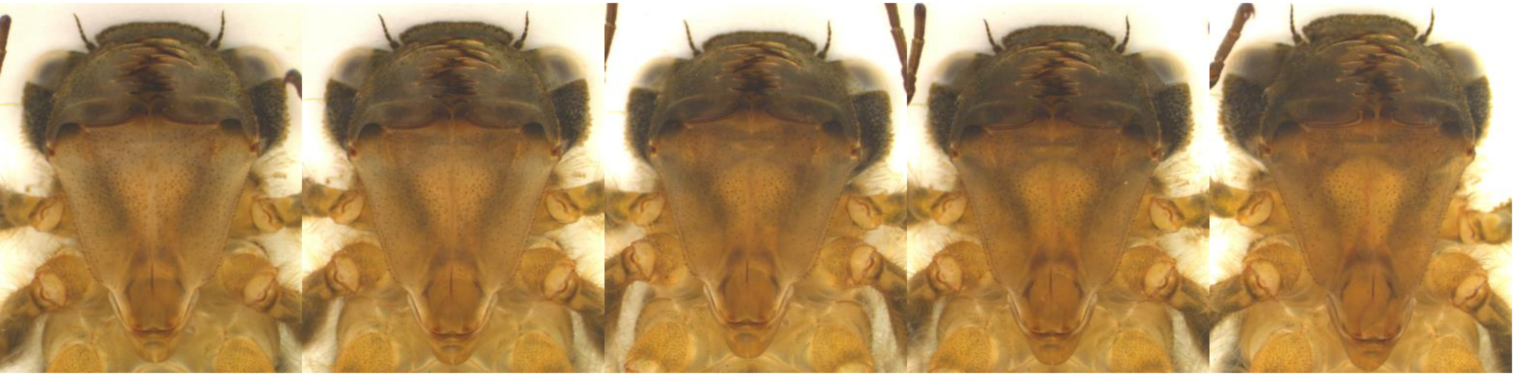

39-3 *Sympetrum darwinianum* (1/1)

2 mm

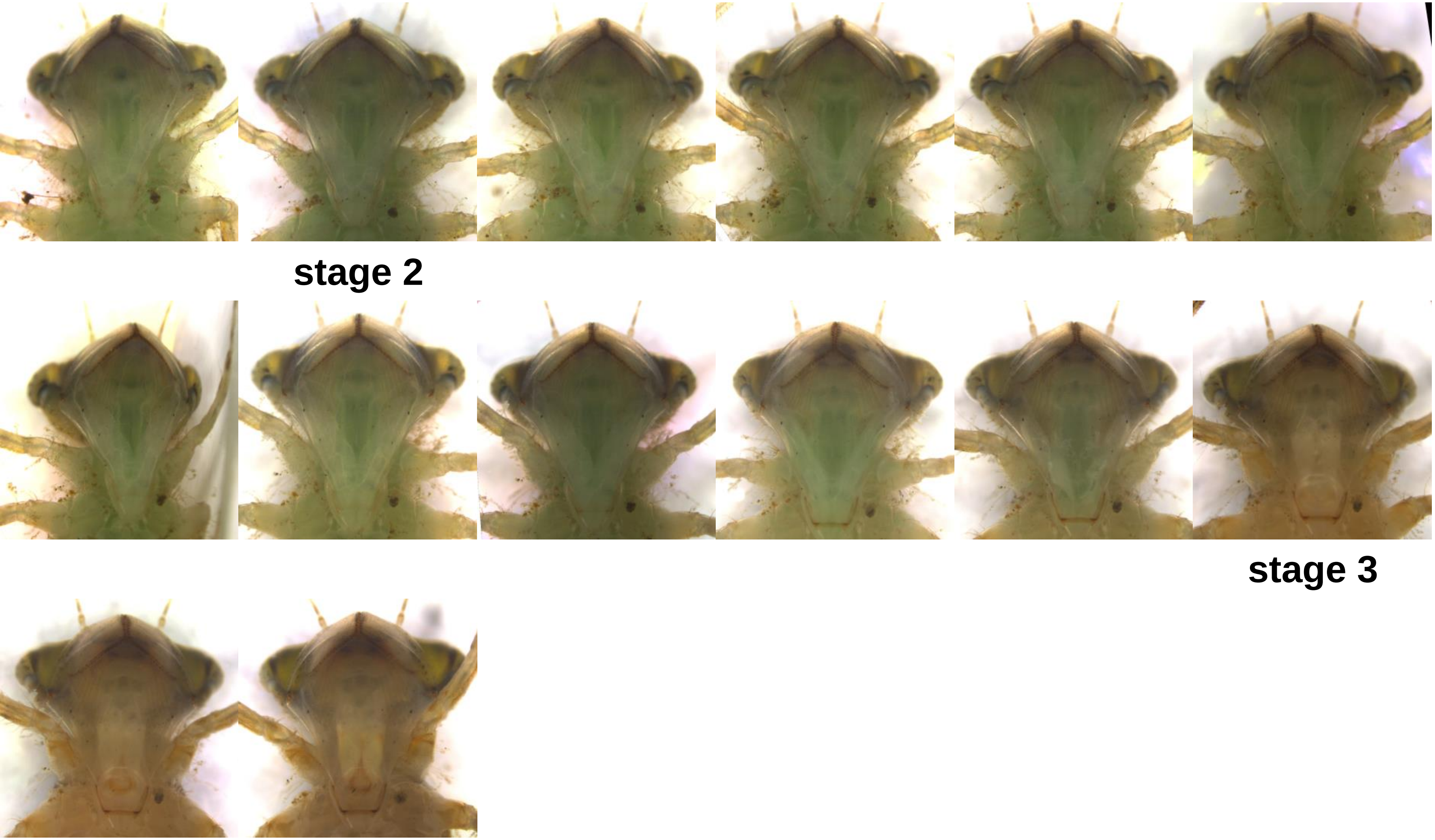

40-1 *Sympetrum maculatum* (1/1)

2 mm

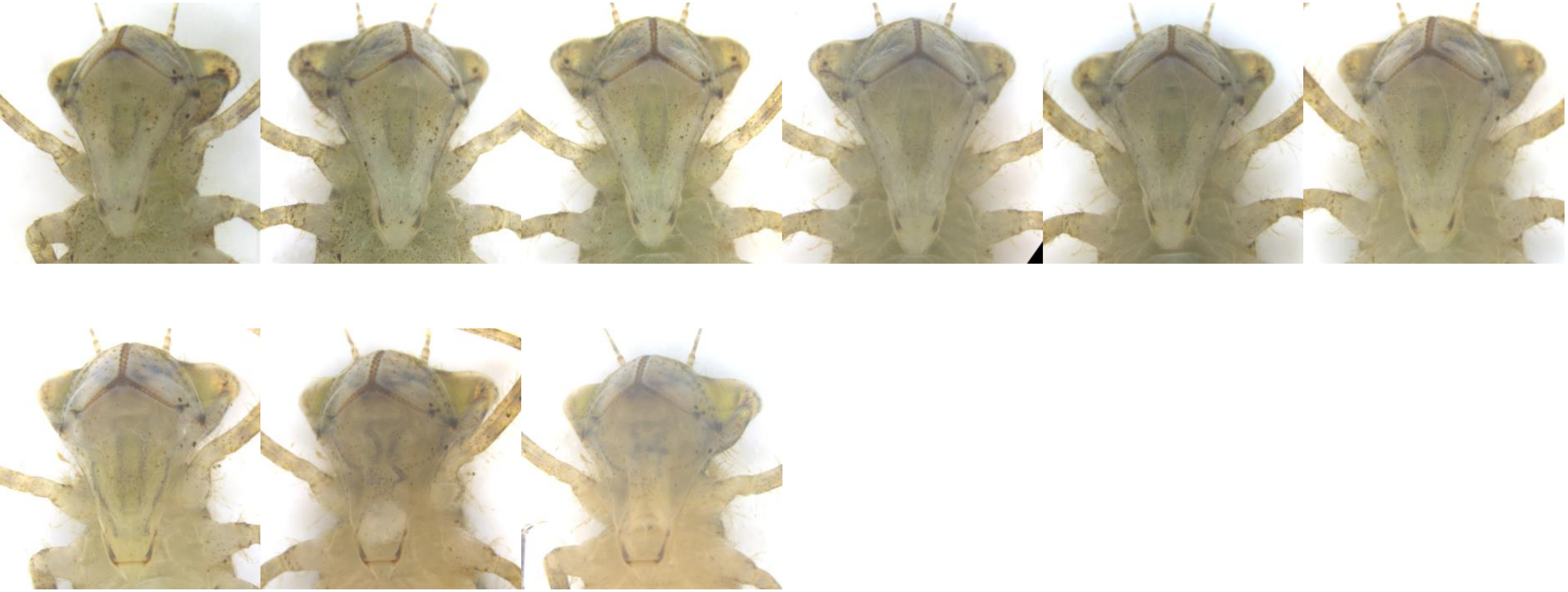

stage 3

40-2 *Sympetrum maculatum* (1/1)

2 mm

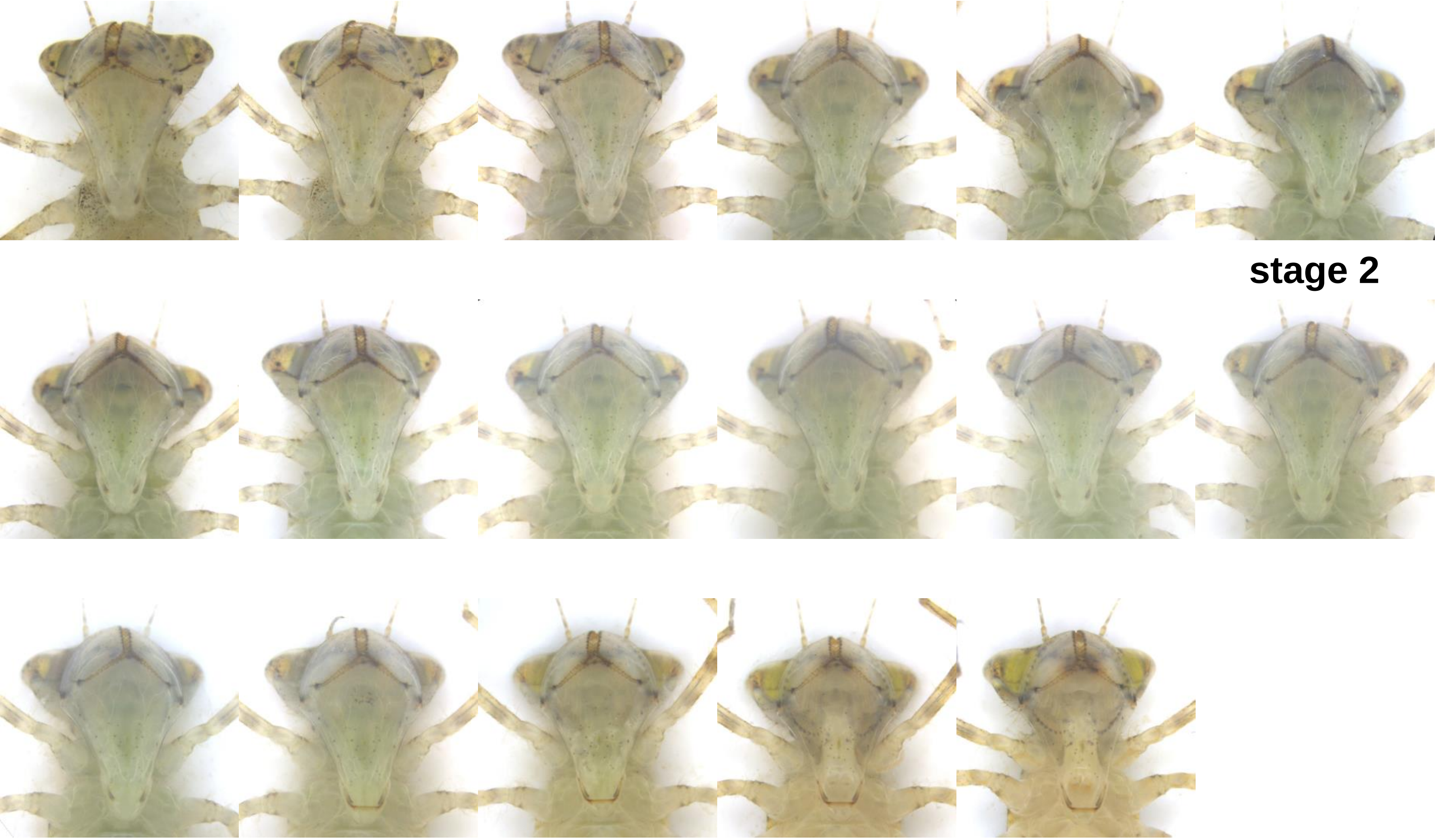

stage 3

40-3 *Sympetrum maculatum* (1/2)

2 mm

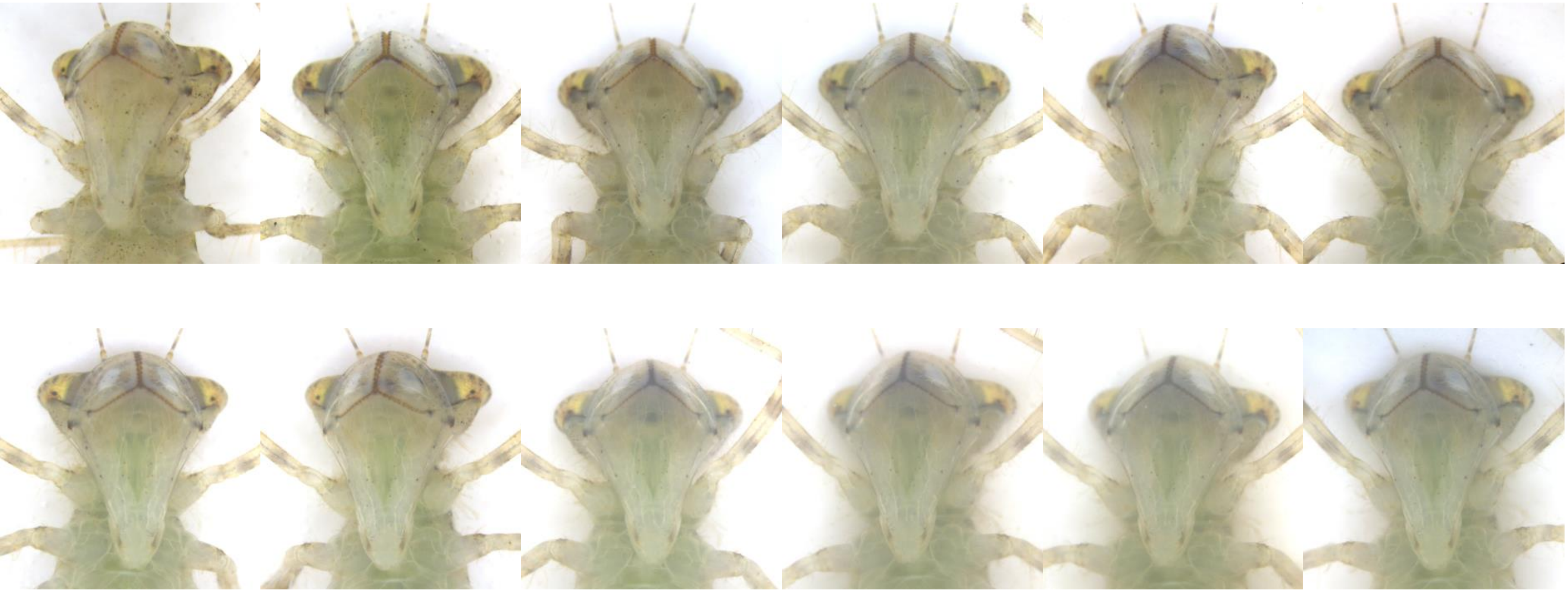

stage 2

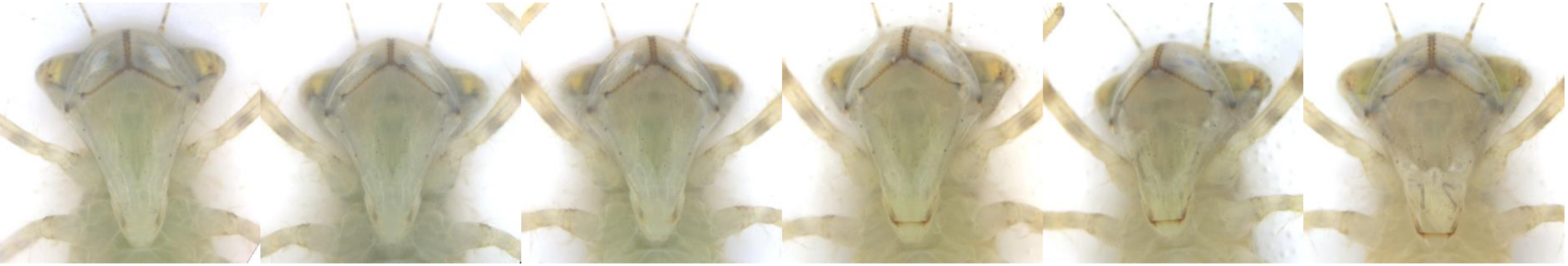

40-3 *Sympetrum maculatum* (2/2)

2 mm

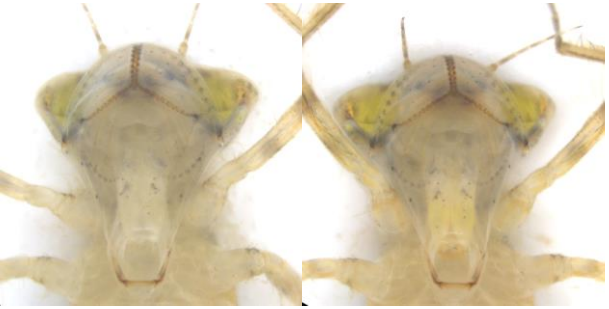

stage 3

40-4 *Sympetrum maculatum* (1/1)

2 mm

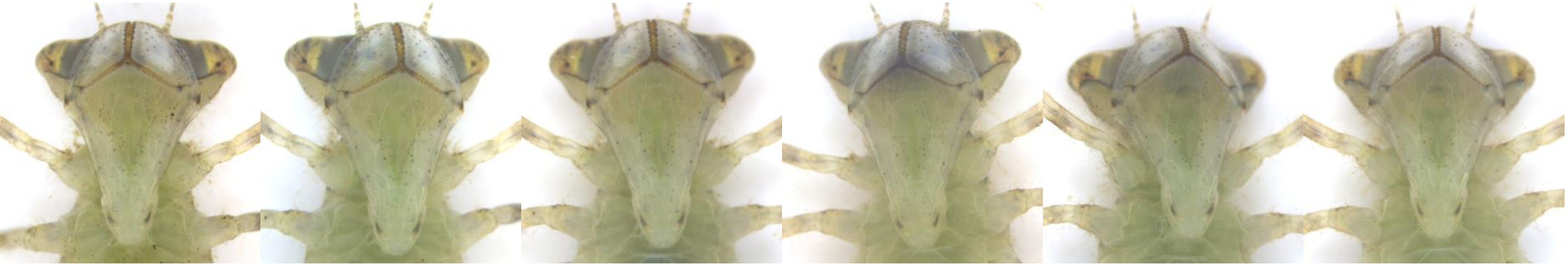

stage 2

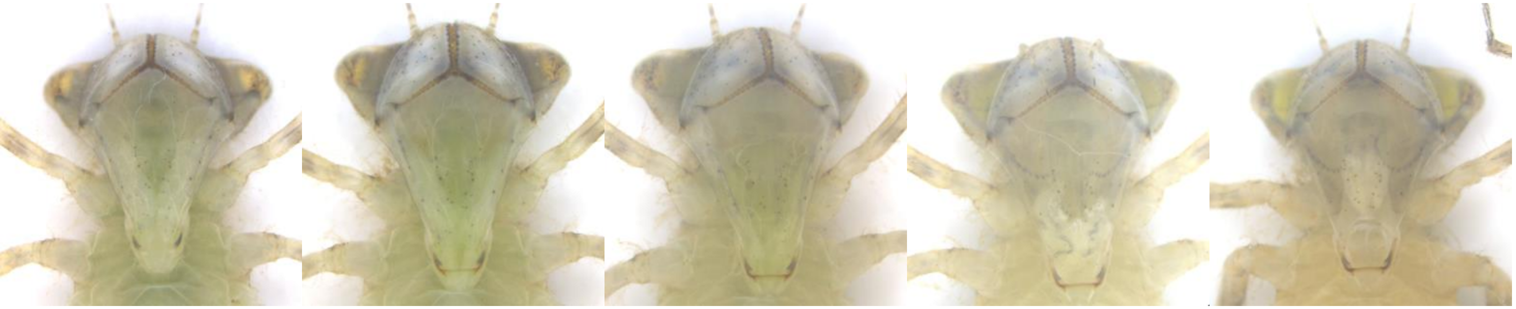

stage 3
