# Supplementary material for: Comprehensive comparative morphology and developmental staging of final instar larvae toward metamorphosis in the insect order Odonata
Source: Sci Rep. 2021 Mar 4;11:5164. doi: 10.1038/s41598-021-84639-2 (PMC7970851; doi:10.1038/s41598-021-84639-2)
Supplement: Supplementary file 5 — Supplementary Figure S4. [file 41598_2021_84639_MOESM5_ESM.pdf]

Comprehensive comparative morphology and developmental staging of final instar larvae toward metamorphosis in the insect order Odonata

Genta Okude, Takema Fukatsu, Ryo Futahashi

Figure S4

Examples of enlarged views before and after entering each stage. (A) *Calopteryx japonica* (No. 4-1). (B) *Anax parthenope* (No. 23-1).

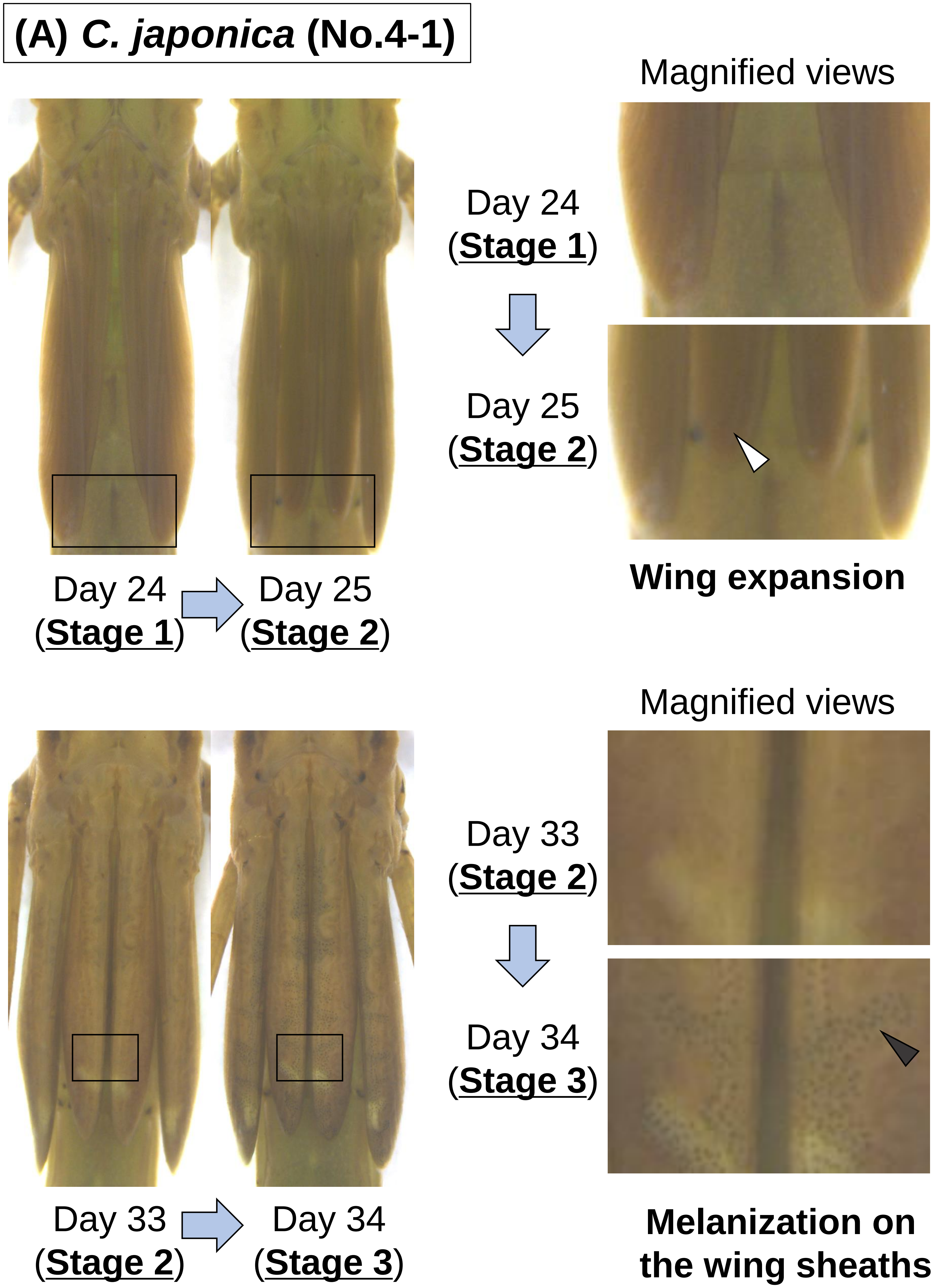
(A) C. japonica (No.4-1)
Magnified views
Day 24
(Stage 1)
Day 25
(Stage 2)
Day 24
(Stage 1)
Day 25
(Stage 2)
Wing expansion
Magnified views
Day 33
(Stage 2)
Day 34
(Stage 3)
Day 33
(Stage 2)
Day 34
(Stage 3)
Melanization on
the wing sheaths

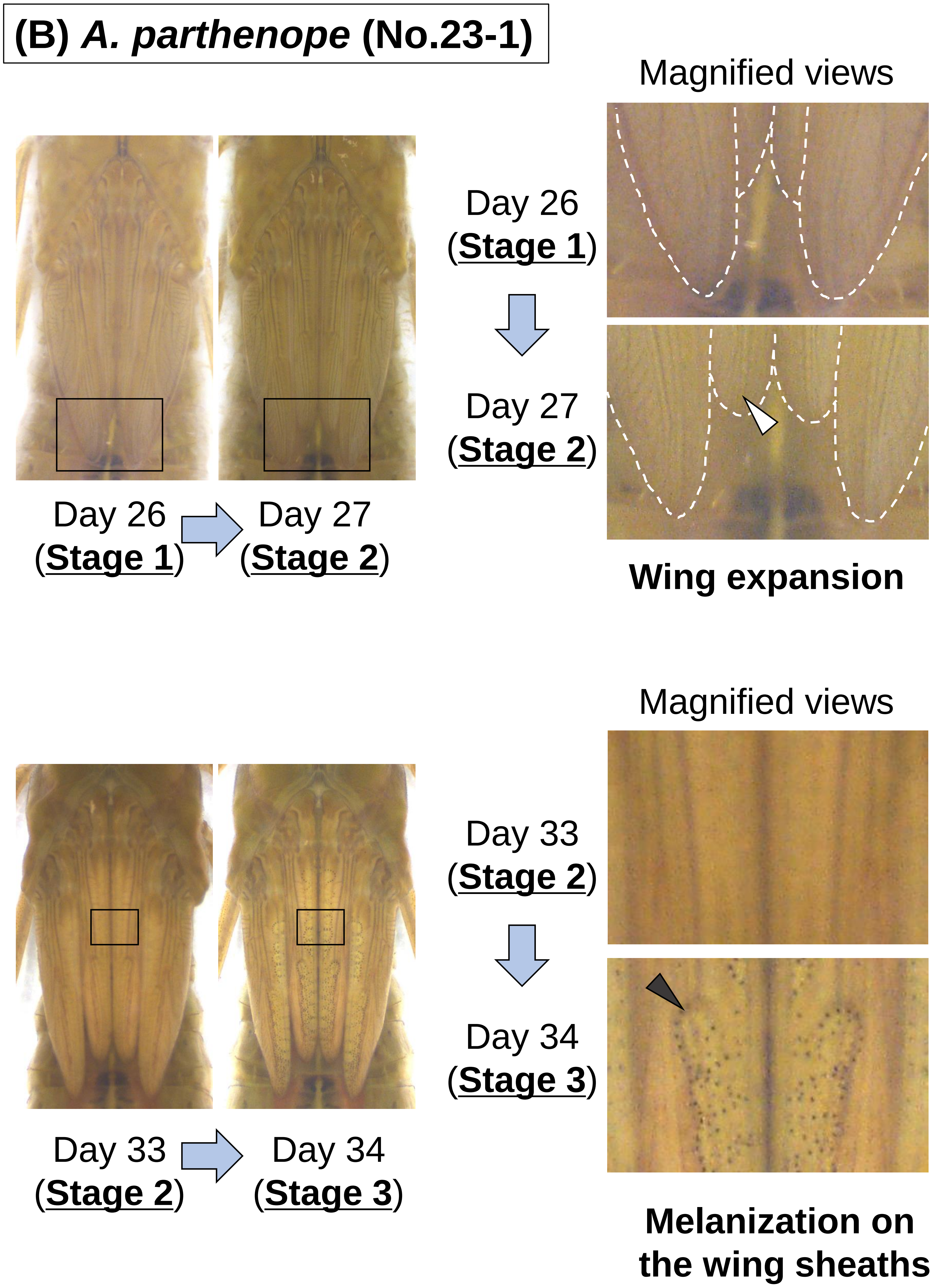
(B) A. parthenope (No.23-1)
Magnified views
Day 26
(Stage 1)
Day 27
(Stage 2)
Day 26
(Stage 1)
Day 27
(Stage 2)
Wing expansion
Magnified views
Day 33
(Stage 2)
Day 34
(Stage 3)
Day 33
(Stage 2)
Day 34
(Stage 3)
Melanization on
the wing sheaths
