# Supplementary material for: Comprehensive comparative morphology and developmental staging of final instar larvae toward metamorphosis in the insect order Odonata
Source: Sci Rep. 2021 Mar 4;11:5164. doi: 10.1038/s41598-021-84639-2 (PMC7970851; doi:10.1038/s41598-021-84639-2)
Supplement: Supplementary file 6 — Supplementary Figure S5. [file 41598_2021_84639_MOESM6_ESM.pdf]

Comprehensive comparative morphology and developmental staging of final instar larvae toward metamorphosis in the insect order Odonata

Genta Okude, Takema Fukatsu, Ryo Futahashi

Figure S5

Durations of stage 2 and stage 3 of observed larvae. Duration of stage 1 is not included because we started to photograph in the middle of stage 1 for most individuals of river dwelling species. Error bars show standard deviations. Abbreviations: Lest, Lestidae; Calo, Calopterygidae; Plat, Platycnemidididae; Coen, Coenagrionidae; Aesh, Aeshnidae; Gomp, Gomphidae; Peta, Petaluridae; Chlo, Chlorogomphidae; Cordule, Cordulegastridae; Macr, Macromiidae; Corduli: Corduliidae; Libe, Libellulidae.

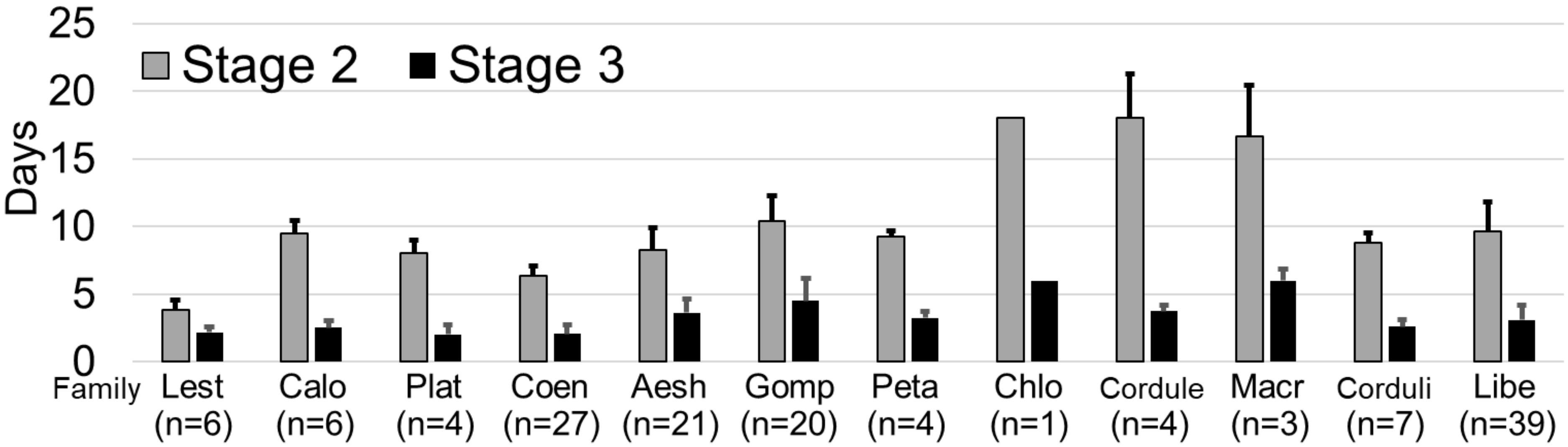
Stage 2
Stage 3
Days
25
20
15
10
5
0
Family
Lest (n=6)
Calo (n=6)
Plat (n=4)
Coen (n=27)
Aesh (n=21)
Gomp (n=20)
Peta (n=4)
Chlo (n=1)
Cordule (n=4)
Macr (n=3)
Corduli (n=7)
Libe (n=39)
